# Genome-wide identification and analysis of mitogen activated protein kinase kinase kinase gene family in grapevine (*Vitis vinifera*)

**DOI:** 10.1186/s12870-014-0219-1

**Published:** 2014-08-27

**Authors:** Gang Wang, Arianna Lovato, Annalisa Polverari, Min Wang, Ying-Hai Liang, Yuan-Chun Ma, Zong-Ming Cheng

**Affiliations:** College of Horticulture, Nanjing Agricultural University, Nanjing, Jiangsu 210095 China; Institute of Botany, Jiangsu Province and Chinese Academy of Sciences, Nanjing, Jiangsu 210014 China; Department of Biotechnology, University of Verona, Strada Le Grazie 15, 37134 Verona, Italy; Department of Plant Sciences, University of Tennessee, Knoxville, TN 37996 USA

**Keywords:** Grapevine, Mitogen-activated protein kinase kinase kinase (MAPKKK), Gene family, Phylogenetic analysis, Expression analysis, Stresses

## Abstract

**Background:**

Mitogen-activated protein kinase kinase kinases (MAPKKKs; MAP3Ks) are important components of MAPK cascades, which are highly conserved signal transduction pathways in animals, yeast and plants, play important roles in plant growth and development. MAPKKKs have been investigated on their evolution and expression patterns in limited plants including *Arabidopsis*, rice and maize.

**Results:**

In this study, we performed a genome-wide survey and identified 45 MAPKKK genes in the grapevine genome. Chromosome location, phylogeny, gene structure and conserved protein motifs of MAPKKK family in grapevine have been analyzed to support the prediction of these genes. In the phylogenetic analysis, MAPKKK genes of grapevine have been classified into three subgroups as described for *Arabidopsis*, named MEKK, ZIK and RAF, also confirmed in grapevine by the analysis of conserved motifs and exon-intron organizations. By analyzing expression profiles of MAPKKK genes in grapevine microarray databases, we highlighted the modulation of different MAPKKKs in different organs and distinct developmental stages. Furthermore, we experimentally investigated the expression profiles of 45 grape MAPKKK genes in response to biotic (powdery mildew) and abiotic stress (drought), as well as to hormone (salicylic acid, ethylene) and hydrogen peroxide treatments, and identified several candidate MAPKKK genes that might play an important role in biotic and abiotic responses in grapevine, for further functional characterization.

**Conclusions:**

This is the first comprehensive experimental survey of the grapevine MAPKKK gene family, which provides insights into their potential roles in regulating responses to biotic and abiotic stresses, and the evolutionary expansion of MAPKKKs is associated with the diverse requirement in transducing external and internal signals into intracellular actions in MAPK cascade in grapevine.

**Electronic supplementary material:**

The online version of this article (doi:10.1186/s12870-014-0219-1) contains supplementary material, which is available to authorized users.

## Background

Plants are constantly confronted by various pathogenic and environmental stresses that challenge their survival. To deal with stresses, plants have evolved a variety of biochemical and physiological mechanisms. Stress-activated molecular pathways include multiple inter-linked regulatory networks such as protein kinase signaling cascades that can efficiently transduce input signals into suitable outputs [1]. The best characterized protein-kinase-based amplification cascades rely on the mitogen activated protein kinases (MAPKs), which are conserved components of signal transduction in all eukaryotic organisms [2]. The MAPK cascades rapidly transduce stress signals into various appropriate intracellular responses [3]. The basic MAPK cascades are composed of three classes of protein kinases: MAPK kinase kinase (MAPKKK/MAP3K), MAP kinase (MAPKK/MKK) and MAPK (MAPK/MPK). MAPKKKs are the first component of the cascades that activate MAPKKs by phosphorylating two amino acids in the S/T-XXXXX-S/T (x represents any amino acid) motif of the MAPKK activation loop, and then MAPKKs become dual-specificity kinases that activate the downstream MAPK through double phosphorylation of the T-X-Y motif in the activation loop (T-loop) [4,5]. The activated MAPK leads to the phosphorylation of transcription factors and other signaling components that regulate the expression of downstream target genes [6].

So far, different members in MAPK cascades have been identified and characterized by functional genomics approach in a variety of plant species, including *Arabidopsis*, tobacco, rice, alfalfa and poplar. *Arabidopsis thaliana* genome contains 80 MAPKKKs, 10 MAPKKs and 20 MAPKs [6,7], whereas the rice genome contains 75 MAPKKKs, 8 MAPKKs and 17 MAPKs [8,9]. Compared with MAPKs and MAPKKs, MAPKKKs act at the top of MAPK cascades with much greater numbers and show more complexity and sequence diversity. According to characteristic sequence motifs, MAPKKKs are divided into three groups in higher plants: the MEKK-like subfamily, ZIK subfamily and Raf-like subfamily. Compared to ZIK subfamily and RAF-like subfamily, MEKK subfamily members have a less conserved protein structure [8]. The RAF and ZIK subfamily proteins have a C-terminal kinase domain (KD) and a long N-terminal regulatory domain (RD) that might function in scaffolding to recruit MAPKKs and MAPKs [3,4].

In plants, MAPK cascades have been implicated in the signaling pathways related to various stresses, ethylene signaling, innate immunity and defense responses [10-12]. In *Arabidopsis*, the cascade MEKK1-MKK4/5-MPK3/6-WRKY22/WRKY29 plays an important role in plant innate immunity [11]. Investigations in alfalfa (*Medicago sativa*) have indicated that OMTK1, a MAPKKK, was activated by hydrogen peroxide (H_2_O_2_) [13]. Two well-studied MAPKKK are CTR1 (Constitutive Triple Response 1) and EDR1 (Enhanced Disease Resistance 1) of *A. thaliana*, both belonging to the RAF-like subfamily. The CTR1 multigene family encodes an essential negative regulator for ethylene-induced gene expression in *Arabidopsis* [14], while EDR1 was shown to be a negative regulator in salicylic acid-inducible defense responses [15] with *edr1* mutants showing increased resistance to powdery mildew [16]. In addition, it was reported that *AtRaf5* mutant exhibited an enhanced tolerance to salt in *Arabidopsis* [10]. Over-expression of *Os-MAPKKK6* increased the tolerance to dehydration stress through ROS scavenging in rice [17]. In contrast to several reports on MAPKKKs in *Arabidopsis* and rice, research on MAPKKKs in grapevine is still very limited.

Grapevine (*Vitis vinifera* L.) is one of the most economically valuable and most widely grown fruit crops in the world. Sequencing of the highly homozygous grapevine PN40024 genome [18] provides a great opportunity for analysis of the grapevine genome and gene family evolution. Previously we have validated 12 grapevine MAPK gene by gene isolation and expression [19]. To further understand how the MAPK cascade operates in grapevine and their internal relationships, we surveyed the gene family of MAPKKKs, the top of the MAPK cascade in the grapevine genome. Fourty-five grapevine MAPKKK genes were identified by a detailed bioinformatics analysis, annotated and named according to their sequence similarity with Arabidopsis genes, as established by the grapevine scientific community ([20] personal communication) and their chromosomal position and gene structure were determined. In addition, we analyzed their transcript profiles in different organs and developmental stages using published microarray data. Finally, we examined their expression patterns in response to different stresses using quantitative real time polymerase chain reaction (qRT-PCR). These results indicate that the evolutionary expansion of MAPKKKs is associated with the diverse requirement in transducing external and internal signals into intracellular actions in MAPKKK-MAPKK-MAPK cascade in grapevine.

## Results and discussion

### Identification of MAPKKK family in grapevine and construction of a phylogenetic tree

**Figure 1:**
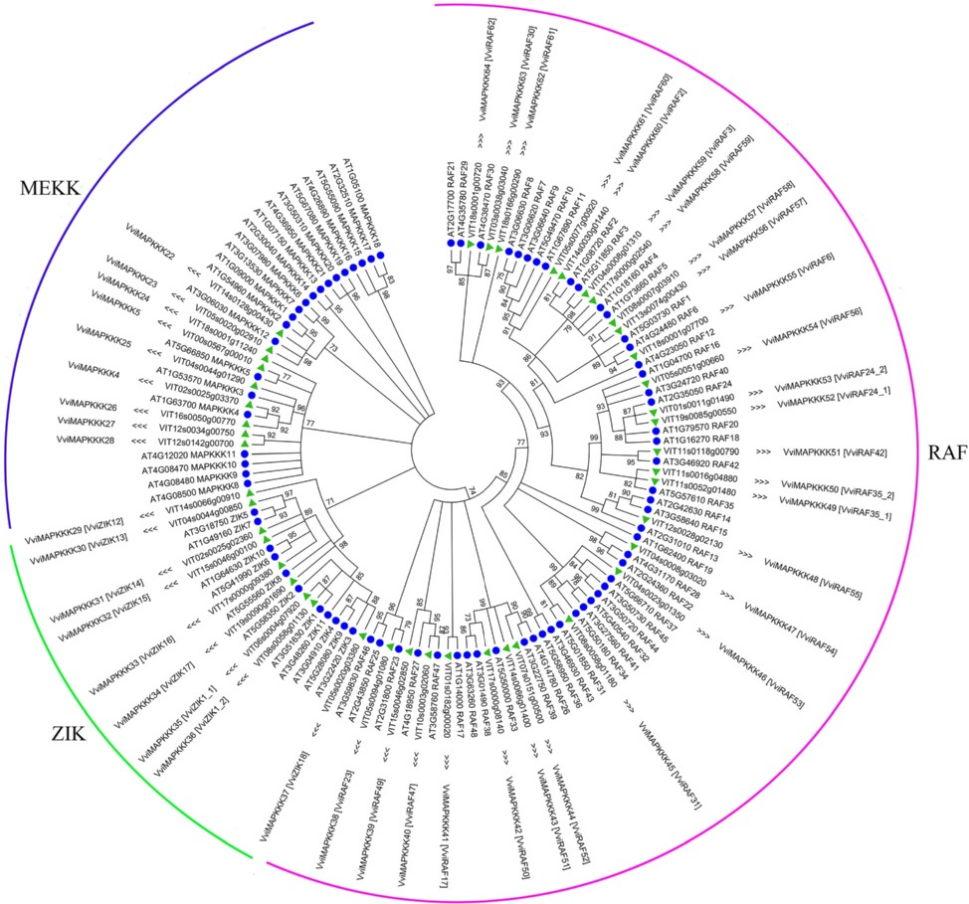
**Phylogenetic relationships of MAPKKK in** ***A. thaliana*** **and** ***V. vinifera*** **(sequenced genotype PN40024).** The phylogenetic tree was created using MEGA5 program with the neighbor-joining (NJ) method using full length sequences of 45 grapevine and 80 *Arabidopsis* MAPKKK proteins. Bootstrap values for 2000 replicates are indicated at each branch. To identify the species of origin for each MAPKKK, a species acronym is included before the protein name: AtMEKK, AtRAF, AtZIK for MAP3K from *A. thaliana*; VviMAPKKKs for MAPKKK from *V. vinifera*.

Availability of the complete grapevine genome sequence has made it possible for the first time to identify all the MAPKKK family members in this plant species. With this aim, we performed HMMER searches using 80 *Arabidopsis* MAPKKK sequences as query and identified a total of 45 MAPKKK genes from the grapevine genome. These genes were named according to the rules recently established by the grapevine scientific community ([20]; Grimplet J., personal communication). Functional gene names were assigned according to their sequence similarity to *Arabidopsis* genes, and following the nomenclature reported in the TAIR database (Table 1). In all cases the Locus ID reported on the V1 grapevine genome browser (http://genomes.cribi.unipd.it/grape/) is also reported, to provide a unique identifier and avoid mistakes during future conversion from different sources. The phylogenetic tree described above was constructed with the Phylogeny.fr web service [21], to provide a repeatable phylogenetic tree. All genes received a functional name (MAPKKK) followed by a number higher than the highest number used for *Arabidopsis*. Therefore, the progressive numbering of grapevine gene names procedes along the phylogenetic tree in Figure 1 from left to right. Only when a one-to-one orthology was present in the Arabidopsis MEKK subfamily, the grapevine gene was given the corresponding Arabidopsis-like name (example: AtMAPKKK4 and VviMAPKKK4). In the other subfamilies, the RAF or ZIK names were used as synonyms, derived from the Arabidopsis orthologous (example: AtRAF17 and VviMAPKKK41 [VviRAF17]). If two or more grapevine genes had the same phylogenetic distance from a single homologue in Arabidopsis, they were differentiated by a number (example: AtRAF24, VviMAPKKK52 [VviRAF24_1] and VviMAPKKK53 [VviRAF24_2]).

**Table 1:** **Characteristics of MAPKKKs of grapevine**

**Name**	**ID**	**Chromosomal localization**	**Gene length (bp)**	**Aminoacid length (AA)**	**PI**	**MW (KD)**
VviMAPKKK4	VIT02s0025g03370	chr2:2874846-2883582	8737	901	9.16	97.80
VviMAPKKK5	VIT00s0567g00010	chrUn:31987912-3199568	7770	707	10.32	77.59
VviMAPKKK22	VIT14s0128g00430	chr14:3063713-3072185	8473	670	5.13	73.67
VviMAPKKK23	VIT05s0020g02910	chr5:4619268-4633136	13869	686	7.18	75.83
VviMAPKKK24	VIT18s0001g11240	chr18:9550551-9559415	8865	520	8.41	57.26
VviMAPKKK25	VIT04s0044g01290	chr4:22814326-22820168	5843	623	9.18	67.71
VviMAPKKK26	VIT16s0050g00770	chr16:17708276-17713661	5386	892	9.84	95.83
VviMAPKKK27	VIT12s0034g00750	chr12:16523826-16552015	28190	313	7.14	35.28
VviMAPKKK28	VIT12s0142g00700	chr12:615727-641062	25336	264	7.05	30.11
VviMAPKKK29 [VviZIK12]	VIT14s0066g00910	chr14:27366994-27370901	3908	676	5.32	76.00
VviMAPKKK30 [VviZIK13]	VIT04s0044g00850	chr4:21993993-21997860	3868	677	4.80	75.75
VviMAPKKK31 [VviZIK14]	VIT02s0025g02360	chr2:2095996-2099570	3575	645	4.76	72.78
VviMAPKKK32 [VviZIK15]	VIT15s0046g00100	chr15:17151522-17154829	3.308	625	5.66	70.53
VviMAPKKK33 [VviZIK16]	VIT17s0000g09380	chr17:10966252-10980394	14143	669	5.18	74.92
VviMAPKKK34 [VviZIK17]	VIT19s0090g01690	chr19:7729733-7731410	1678	297	6.60	34.10
VviMAPKKK35 [VviZIK1_1]	VIT06s0004g07920	chr6:8665166-8669149	3984	631	7.27	71.63
VviMAPKKK36 [VviZIK1_2]	VIT08s0058g01130	chr8:10519601-10524077	4477	626	5.15	71.72
VviMAPKKK37 [VviZIK18]	VIT05s0020g03380	chr5:5145523-5148774	3252	729	4.83	83.53
VviMAPKKK38 [VviRAF23]	VIT05s0094g01080	chr5:24408625-24418281	9657	472	9.47	53.27
VviMAPKKK39 [VviRAF49]	VIT15s0046g02850	chr15:19576430-19587223	10794	540	7.52	61.84
VviMAPKKK40 [VviRAF47]	VIT10s0003g02060	chr10:3697834-3713475	15642	462	6.80	52.52
VviMAPKKK41 [VviRAF17]	VIT01s0182g00020	chr1:13949463-13973640	24178	435	8.00	49.13
VviMAPKKK42 [VviRAF50]	VIT17s0000g08140	chr17:9166532-9172436	5905	381	6.91	42.34
VviMAPKKK43 [VviRAF51]	VIT14s0066g01400	chr14:27812915-27819523	6609	360	6.77	40.53
VviMAPKKK44 [VviRAF52]	VIT07s0151g00500	chr7:944982-950180	5199	404	7.37	44.93
VviMAPKKK45 [VviRAF31]	VIT08s0058g01180	chr8:10650470-10661269	10800	337	9.04	37.97
VviMAPKKK46 [VviRAF53]	VIT04s0023g01350	chr4:17821355-17824440	3086	211	9.74	23.84
VviMAPKKK47 [VviRAF54]	VIT04s0008g03020	chr4:2501035-2518539	17505	417	7.33	46.58
VviMAPKKK48 [VviRAF55]	VIT12s0028g02130	chr12:2837360-2886960	49601	817	6.34	91.58
VviMAPKKK49 [VviRAF35_1]	VIT11s0052g01480	chr11:19187047-19205338	18292	1136	5.52	126.16
VviMAPKKK50 [VviRAF35_2]	VIT11s0016g04880	chr11:4194812-4202353	7542	1021	8.12	114.11
VviMAPKKK51 [VviRAF42]	VIT11s0118g00790	chr11:6573516-6581639	8124	1425	4.97	155.43
VviMAPKKK52 [VviRAF24_1]	VIT19s0085g00550	chr19:22924891-22931831	6941	1169	4.90	129.39
VviMAPKKK53 [VviRAF24_2]	VIT01s0011g01490	chr1:130365-1315467	11817	1238	5.21	136.63
VviMAPKKK54 [VviRAF56]	VIT05s0051g00660	chr5:11545076-11551320	6245	1217	5.16	135.17
VviMAPKKK55 [VviRAF6]	VIT18s0001g07700	chr18:5939219-5949276	10058	905	8.20	101.38
VviMAPKKK56 [VviRAF57]	VIT13s0074g00430	chr13:8150122-8202670	52549	758	7.89	85.13
VviMAPKKK57 [VviRAF58]	VIT08s0007g03910	chr8:17882576-17901834	19259	856	6.30	94.50
VviMAPKKK58 [VviRAF59]	VIT17s0000g02540	chr17:2321847-2341848	20002	1033	5.11	112.44
VviMAPKKK59 [VviRAF3]	VIT04s0008g01310	chr4:1079716-1090458	10743	914	5.12	101.12
VviMAPKKK60 [VviRAF2]	VIT14s0030g01440	chr14:6026362-6048716	22355	986	6.15	109.38
VviMAPKKK61 [VviRAF60]	VIT05s0077g00920	chr5:694789-701729	6941	771	7.56	85.74
VviMAPKKK62 [VviRAF61]	VIT18s0166g00290	chr18:14296029-14329589	33561	550	4.87	61.82
VviMAPKKK63 [VviRAF30]	VIT03s0038g03040	chr3:2141254-2162029	20776	580	5.75	64.54
VviMAPKKK64 [VviRAF62]	VIT18s0001g00720	chr18:1477309-1491529	14221	522	7.17	59.35

When one or more genes in grapevine matched more than one gene in Arabidopsis, a new name was attributed consisting of the common MAPKKK term and an increasing numbering. The detailed information on the VviMAPKKK genes identified in the present study is listed in Table 1 and Additional file 1, including nomenclature, accession numbers, chromosomal localizations, gene length, number of amino acid in the protein, isoelectric point (PI) and molecular weight (MW). These genes were distributed over almost all chromosomes, except chromosome 9. The gene length ranged from 1,678 bp (VviMAPKKK34) to 52,549 bp (VviMAPKKK6). The open reading frames (ORFs) encoded polypeptides ranging from 211 AA (VviMAPKKK46) to 1425 AA (VviMAPKKK51). The predicted molecular masses ranged from 23.84 to 155.43 kD and isoelectric point value ranged 4.76-10.32 (Table 1). According to the present study, the number of grapevine MAPKKK genes is significantly smaller than those of *Arabidopsis* MAPKKKs (80) [6] and rice MAPKKKs (75) [8].

### Phylogenetic analysis of VviMAPKKK genes

To investigate the evolutionary relationships between MAPKKK members in grapevine and *Arabidopsis*, and also to assign a name to grapevine MAPKKK genes (see below) a phylogenetic tree was constructed from alignments of the full coding sequences of all 125 MAPKKK genes (45 from grapevine and 80 from *Arabidopsis*, Additional file 2) with the procedure and parameters described in Materials and Methods (Figure 1). Based on the phylogenetic tree, grapevine MAPKKK were classified into the same corresponding categories in *Arabidopsis*, which include MEKK-like, RAF and ZIK subfamilies. There were 9 VviMAPKKKs and 21 AtMAPKKKs in the MEKK subfamily, only 9 VviMAPKKKs and 11 AtZIKs in ZIK subfamily, while 27 VviMAPKKKs and 48 AtRAFs grouped in the RAF subfamily (Figure 1).

In the three clades, there were many grapevine MAPKKKs clustering together, suggesting that these homologous genes may have derived from multiple duplications after the speciation of grape during the evolution. Moreover, many grapevine MAPKKK genes have their clear orthologues in the *Arabidopsis* genome, which suggests that these genes might be conserved for some specific functions in the two species. Interestingly, one grapevine gene, VviMAPKKK29, stands outside the main branches and was included in the ZIK family with a bootstrap values of 71%, just above the threshold of 70% established for the phylogenetic analysis (Figure 1).

### Chromosomal location of VviMAPKKK genes

**Figure 2:**
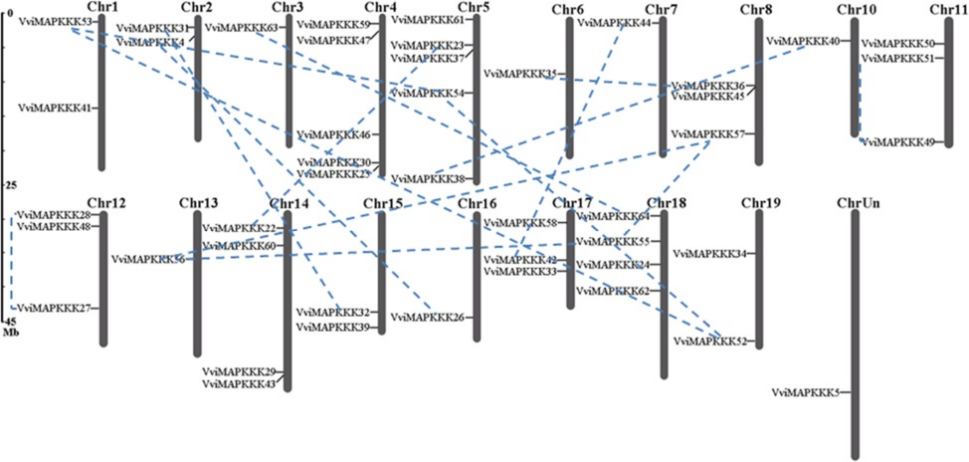
**Chromosomal locations of MAPKKK genes in grapevine genome.** Scale represents chromosomal distance. Chromosomes 1–19 (Chr1-19) are depicted as gray bars. VviMAPKKK genes are indicated by vertical black lines. Chromosome 9, in which no VviMAPKKK gene was located, is not shown. The blue dotted lines connecting VviMAPKKK genes represent duplicate chromosomal segments.

Based on the gene prediction of the grapevine genome, the physical locations of the MAPKKK genes on grape chromosomes are depicted in Figure 2. Fourty-five VviMAPKKK genes mapped on all grapevine chromosomes except chromosome 9, and one MAPKKK (VviMAPKKK5) was situated on the undetermined chromosome (ChrUn). The VviMAPKKK genes were unevenly distributed, with a number of genes per chromosome ranging from one to five (Table 1). We identified 18 paralogs among the 45 grapevine MAPKKKs, 16 of which appeared to result from genome fusion events [18], and the other 2 paralogs within the same chromosome (VviMAPKKK50/VviMAPKKK51, VviMAPKKK27/VviMAPKKK28) were likely generated through tandem duplications (Figure 2). Gene duplication events resulted in gene family members’ amplification in the genome. Although several paralogs such as VviMAPKKK23 and VviMAPKKK22, VviMAPKKK4 and VviMAPKKK26 shared high similarity of amino acid sequences, they were far from each other on different chromosomes.

### Gene structural analysis of VviMAPKKK genes

**Figure 3:**
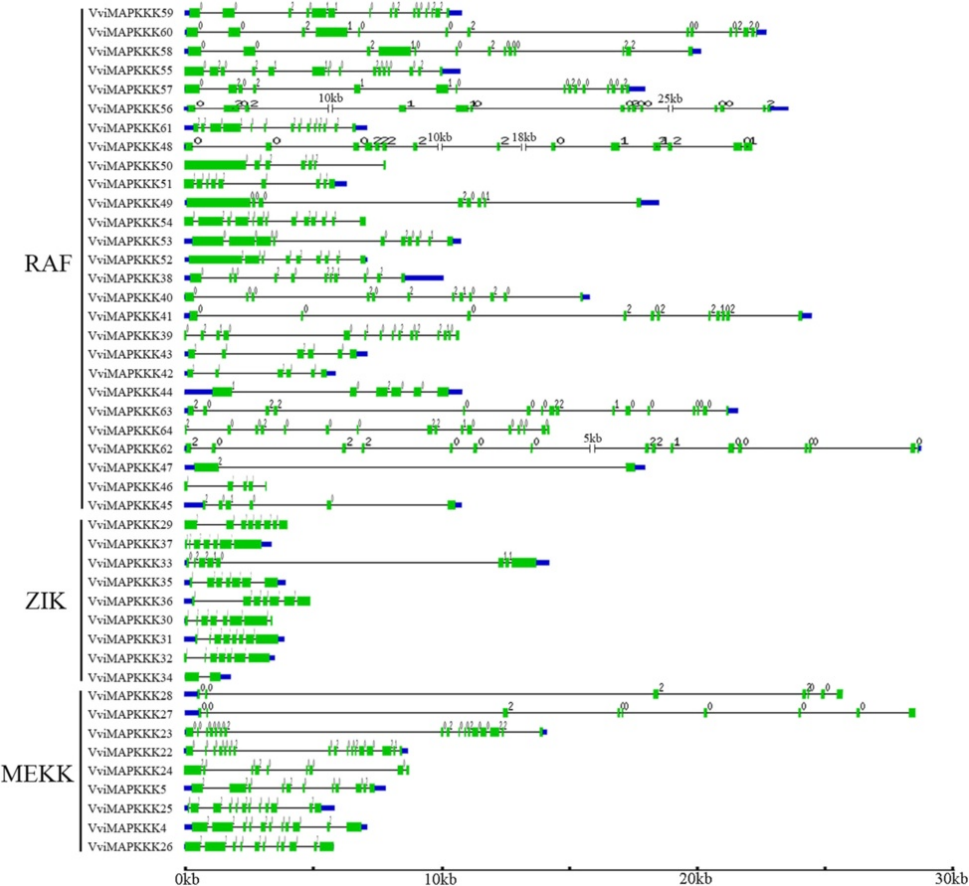
**Schematic diagrams for intron/exon structures of MAPKKK genes in grapevine.** The green boxes indicate the exons while the single lines indicate introns. UTRs are displayed by thick blue lines at both ends. 0, 1 and 2 represent different intron phases. Gene models were drawn to scale as indicated at the bottom.

Exon/intron structure can provide additional evidence to support phylogenetic groupings [22] as exon/intron structure divergence often plays a key role in the evolution of gene families [23]. Moreover, the conservation of gene structure in paralogous genes is usually strong and sufficient to reveal evolutionary relationships [24]. The exon/intron structures of the VviMAPKKK genes were investigated by using the prediction of the grapevine genome (Figure 3).

As shown in Figure 3 and Table S2 (Additional file 3), the number of introns in VviMAPKKK genes was highly variable, ranging from 4 to 16 introns, whereas two genes (VviMAPKKK47 and VviMAPKKK34) had only one intron. The large variation in structures of VviMAPKKK genes suggests that the grapevine genome has changed significantly during its long evolutionary history. However, a certain degree of similarity could be observed among subgroups, supporting evolutionary relationships among members of each clade. The majority of genes in the ZIK subfamily contain 6–7 introns, genes in the MEKK subfamily mostly ranged between 8 and 10 introns, the RAF subfamily showed a variable exon number but with a majority of genes ranging between 12 and 15 introns, often with very long introns. Within this frame, paralogous gene pairs generally shared highly similar exon-intron structures (Figure 3). Collectively, the divergent gene structures between the different phylogenetic subgroups suggest that duplication events of MAPKKK genes might have occurred in ancient times and that offspring genes evolved into diverse exon/intron structures, possibly to accomplish different functions in the grapevine genome.

### Analysis of conserved domains among VviMAPKKKs

The pattern of amino acid residues found in many subdomains is conserved among the family members [8]. All VviMAPKKK genes grouped under MEKK, ZIK and RAF subfamilies were further analyzed for the presence of specific signatures. Nine VviMAPKKKs and 21 AtMAPKKKs which belong to MEKK subfamily share the conserved signature motif G (T/S) Px (W/Y/F) MAPEV, as revealed by the amino acid sequence analysis of the protein kinase domain (Additional file 4: Figure S1A). Presence of this signature in 8 out of 9 VviMAPKKK further confirmed their grouping into the MEKK subfamily, while VviMAPKKK28 showed a substitution of the methionine residue with a threonine. The ZIK subfamily consists of 9 VviMAPKKKs and 11 AtZIKs. The characteristic feature of this subfamily consists of a conserved signature GTPEFMAPE (L/V) Y across all grapevine members (Additional file 4: Figure S1B). No additional kinase domains were identified in the grapevine MEKK or ZIK subfamilies, except for VviMAPKKK27 (Additional file 5: Table S3). One exception is VviMAPKKK29, which clusters together with ZIK-encoding genes at the nucleotide level (Figure 1) with a bootstrap values just above the threshold of 70%, but the alignment of the corresponding predicted protein with other grapevine MAPKKKs at the aminoacid level revealed the presence of a slightly modified RAF domain instead of the typical ZIK domain. For this reason it was simply named VviMAPKKK29, without any reference to subfamily.

The RAF subfamily is the largest of the 3 clades of MAPKKKs. Twenty-seven and 48 MAPKKKs were grouped in the RAF subfamily in grapevine and *Arabidopsis*, respectively. Multiple alignments of the kinase domains revealed the presence of the RAF specific signature GTxx (W/Y) MAPE in almost all grapevine MAPKKK proteins, with only slight variations in VviMAPKKK38 and 40 (Additional file 6: Figure S2). Moreover, the majority of proteins in the RAF subfamily contained additional protein domains (Additional file 2: Table S3), the most frequent one being the EDR1 domain (7 proteins) followed by the PB1 domain (5 proteins), and other additional domains with lower frequencies. Interestingly, two “stress/fungal response” domains were detected in the sequence of VviMPKKK29, which shows a relevant divergence from other members of both clades as already mentioned.

Among the components of the kinase cascade in plants, only a few MAPKKK genes have been characterized. It was shown that AtMAPKKK1 and AtMAPKKK2 played important roles in plant innate immunity [25,26]. MAPKKK1 in *Arabidopsis* was found to be responsible for oxidative stress and to be involved in negative regulation of hormone signaling [27]. It was reported that OMTK1, a MAPKKK from *M. sativa,* regulates oxidative stress signaling [13]. Recently, the *Arabidopsis* AtZIK4 protein WNK1 (At3g04910) was demonstrated to phosphorylate the putative circadian clock component APRR3 in vitro and might be involved in the control of circadian rhythms by regulating its biological activity, suggesting a different function from that of other MAPKKKs [28]. Two of the best-studied RAF-like MAPKKKs in *Arabidopsis*, CTR1 [AtRAF1] and EDR1 [AtRAF2], act as negative regulators in ethylene-induced gene expression [14,29] and in response to powdery mildew attack [16], respectively. However, neither CTR1 nor EDR1 have been confirmed to participate in a classic MAPK cascade [30]. Among those genes, only EDR1 has a clear orthologue in grapevine (VviMAPKKK60 [VviRAF2]) and can be an interesting candidate to ascertain its possible analogous functions in this species, while other characterized *Arabidopsis* MAP3Ks show different degrees of similarity with several grapevine genes.

### Expression profiles of VviMAPKKK genes in different developmental stages and tissues

**Figure 4:**
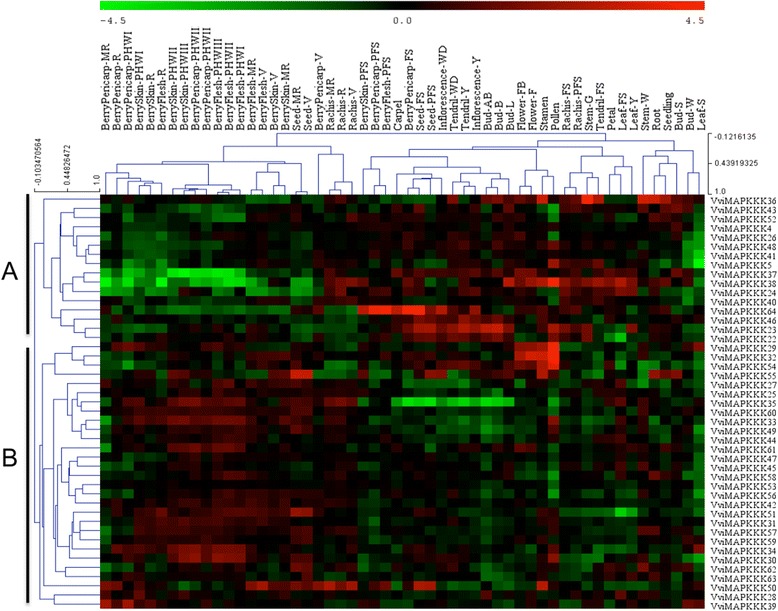
**Hierarchical clustering of the expression profiles of all 45 VviMAPKKK genes in different grapevine developmental stages and tissues.** A total of 54 grapevine samples (flower, berry, bud, leaf, rachis, root, seed, seedling, stem, and tendril) covering most organs at several developmental stages were analyzed. Log2-transformed expression values were used to create the heat map. The red or green colors represent the higher or lower relative abundance of each transcript in each sample, compared to the median expression value of that gene in the whole sample set. Genes and organs were clustered **(A and B)** according to their expression profiles. Developmental stages are abbreviated according to Fasoli et al. [31].

To determine the putative involvement of VviMAPKKK genes in grapevine growth and development during the life cycle, we analyzed their transcript levels in 54 different grapevine tissues corresponding to various developmental stages (including flower, berry, bud, leaf, rachis, root, seed, seedling, stem, and tendril) by performing a hierarchical clustering of a high-throughput microarray dataset from recent research [31]. All 45 VviMAPKKK genes were represented by probes on the array. The heatmap in Figure 4 represents the abundance of each transcript in each sample, normalized on the median expression value of that gene in all samples (Additional file 7), and clustered according to the expression profile in different grapevine tissues and developmental stages. All VviMAPKKK members were expressed in at least one developmental stage of grape organs and most of them did not show striking difference in expression between samples, suggesting these genes may have house-keeping roles in the organ development.

The most peculiar expression of VviMAPKKK was in pollen samples where most genes showed a up- or down-regulation, in comparison to other organs. The clustering of VviMAPKKK s according to their expression profile (Figure 4) revealed that the expression of a group of genes was much higher in young tissues and organs than in ripening or senescing ones, suggesting that these VviMAPKKK are mostly related to signal transduction during development in metabolically active tissues. The decreased transcript levels of VviMAPKKK genes in Cluster A were especially evident in post-withering stages, in which berries are left to natural dehydration for about 3 months. On the opposite, VviMAPKKK transcripts in Cluster B showed a higher level in later stages of grape development and during withering, suggesting that this set of genes may be responsive to dehydration and putatively involved in the deep transcriptomic and metabolic changes controlling biosynthesis of secondary metabolites responsible for the typical aromas of wines. This information can be important for further dissection of the signal transduction pathways operating in the transition from vegetative to reproductive stages [31] and in the regulation of the biosynthesis of aromatic compounds in the berry, in which different groups of MAPKKK may be involved.

It should be noted that the clustering of expression profiles does not reflect phylogenetic similarities. We only found similar expression profiles for the couples of MAPKKKs 22/23, 4/26, 56/57, and 42/47. In general, genes within the 3 clades of MEKK, ZIK and RAF or even paralogous genes may have very different expression profiles and possibly serve different functions in each organ and stage. This could have resulted from post-duplication diversifications, including subfunctionalization, neofunctionalization, or sub-neofunctionalization [32]. These results provide a basis for further investigations on the function of VviMAPKKK genes in grapevine developmental biology.

### Expression profiles of VviMAPKKK genes in response to biotic and abiotic stresses

Only a limited number of genes in MAPKKK family have been functionally characterized in *Arabidopsis* [7] and even less in other species [8]. Among those characterized genes, some were shown to be involved in the response to biotic and abiotic stresses [16,33-35]. In particular, two members of *Arabidopsis* RAF-like MAPKKKs with a function in plant defense were characterized: CTR1 [AtRAF1], negatively regulating ethylene responses [29], and EDR1 [AtRAF2], acting as a negative regulator of disease resistance and ethylene-induced senescence in *Arabidopsis* [16]. Gene expression patterns usually act as indicators of gene function. In the present study, we investigated the expression patterns of all VviMAPKKK genes by semi-quantitative real-time RT-PCR in response to biotic (powdery mildew) and abiotic (drought) stress conditions, as well as in response to hormones (SA, ETH) and H_2_O_2_ treatments. Powdery mildew caused by the biotrophic ascomycete *Erysiphe necator* Schw. adversely affects vine growth, berry quality and grape production worldwide [28]. Salicylic acid (SA), ethylene (ETH) and hydrogen peroxide (H_2_O_2_) play central roles in biotic stress signaling upon pathogen infection. SA and ETH are signal molecules implicated in plant defense responses to pathogens [36,37]. H_2_O_2_ is an important ROS and a critical signaling molecule in cascades leading to plant responses to pathogens and abiotic stress factors [38]. Treated samples were collected in all cases at 6 time points, that is 4, 8, 12, 24, 48 and 72 hours post-treatment (hpt), except for samples subjected to drought stress, which were collected after 4, 8 and 12 days (dpt).

**Figure 5:**
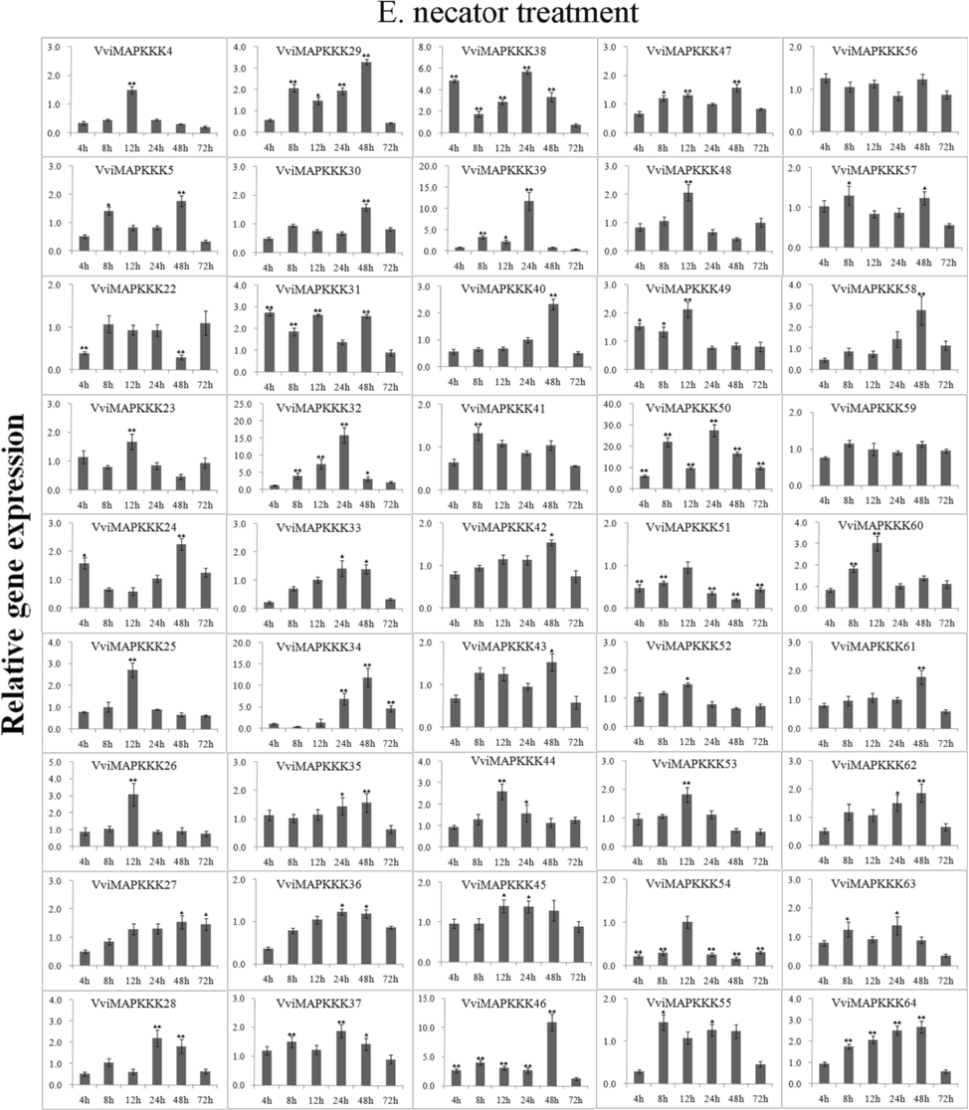
**Expression profiles of VviMAPKKK genes in grapevine leaves in response to powdery mildew infection.** Detached leaves were heavily inoculated with *E. necator* and sampled after 4, 8, 12, 24, 48 and 72 h. To visualize the relative expression levels data are presented as the mean fold changes between treated and control samples at each time point ± standard deviations (SDs). ** and * indicate significant differences in comparison with the control at P < 0.01 and P < 0.05, respectively.

**Figure 6:**
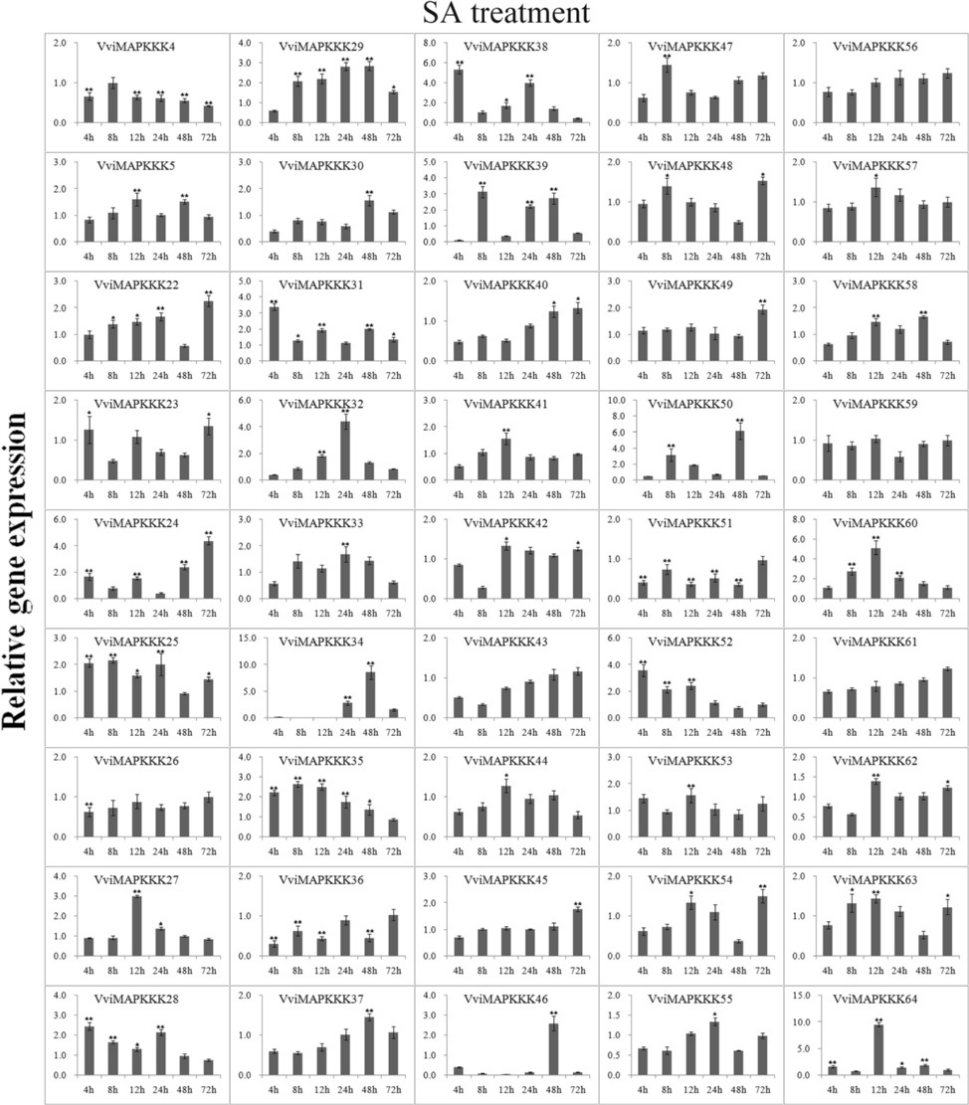
**Expression profiles of VviMAPKKK genes in grapevine leaves in response to SA treatment.** Detached leaves were placed into 5 mM SA and sampled after 0, 4, 8, 12, 24, 48 and 72 h. To visualize the relative expression levels data are presented as the mean fold changes between treated and control samples at each time point ± standard deviations (SDs). ** and * indicate significant differences in comparison with the control at P < 0.01 and P < 0.05, respectively.

**Figure 7:**
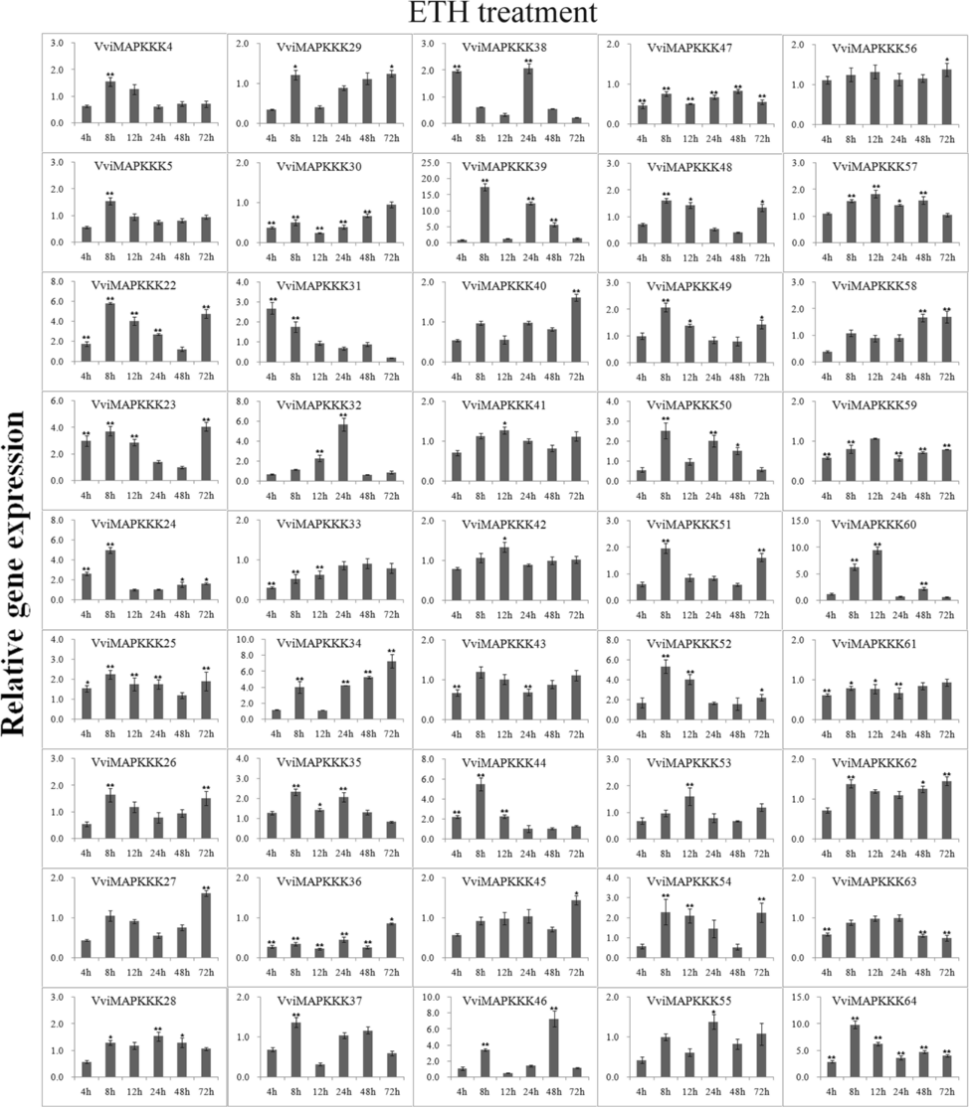
**Expression profiles of VviMAPKKK genes in grapevine leaves in response to ETH treatment.** Detached leaves were placed into 5 mM ethylene (ETH) and sampled after 0, 4, 8, 12, 24, 48 and 72 h. To visualize the relative expression levels data are presented as the mean fold changes between treated and control samples at each time point ± standard deviations (SDs). ** and * indicate significant differences in comparison with the control at P < 0.01 and P < 0.05, respectively.

**Figure 8:**
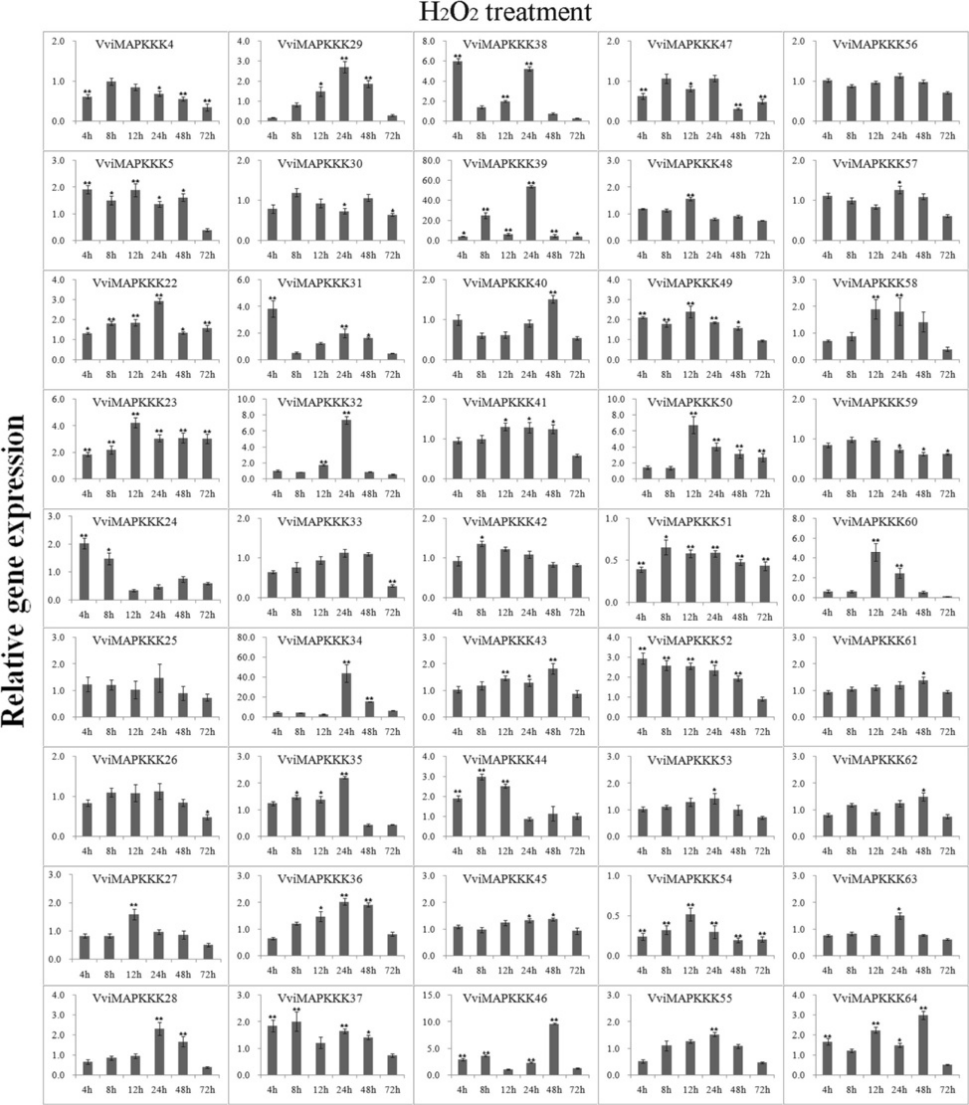
**Expression profiles of VviMAPKKK genes in grapevine leaves in response to H**_**2**_ **O**_**2**_ **treatment.** Detached leaves were placed into 10 mM H_2_O_2_ and sampled after 0, 4, 8, 12, 24, 48 and 72 h. To visualize the relative expression levels data are presented as the mean fold changes between treated and control samples at each time point ± standard deviations (SDs). ** and * indicate significant differences in comparison with the control at P < 0.01 and P < 0.05, respectively.

**Figure 9:**
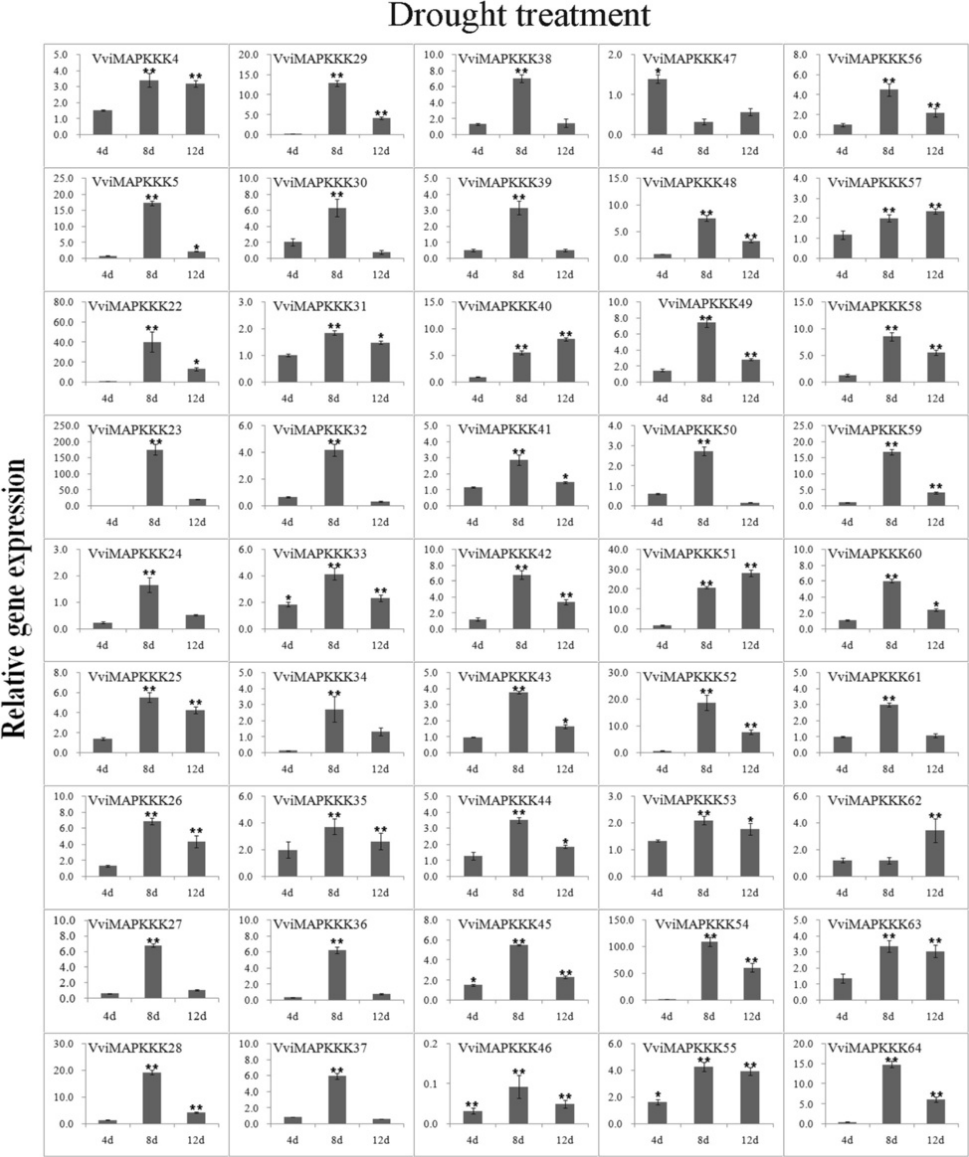
**Expression profiles of VviMAPKKK genes in grapevine leaves in response to drought stress.** Leaves were collected at 4, 8 and 12 d post-drought. To visualize the relative expression levels data are presented as the mean fold changes between treated and control samples at each time point ± standard deviations (SDs). ** and * indicate significant differences in comparison with the control at P < 0.01 and P < 0.05, respectively.

**Figure 10:**
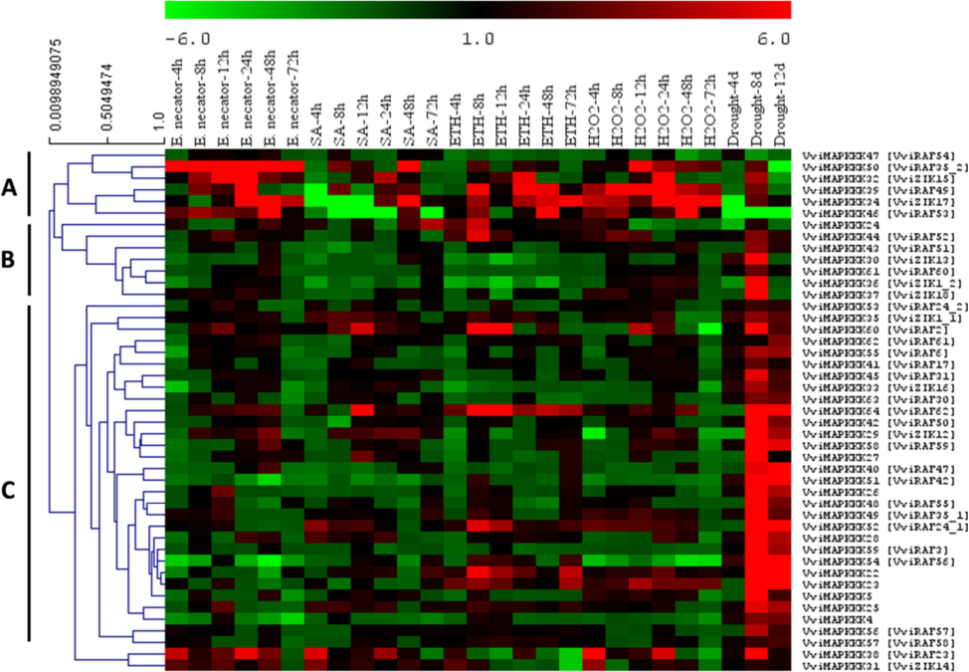
**Hierarchical clustering of the expression profiles of VviMAPKKK genes obtained by quantitative RT-PCR analysis in grapevine leaves in response to different biotic and abiotic stimuli.** *E. necator*: powdery mildew infection; SA: treatments with salicylic acid; ETH: treatments with ethylene, H_2_O_2_: treatments with hydrogen peroxide; Drought: drought treatments. The heat-map reports the fold-change of relative expression for all VviMAPKKK genes in response to the different treatments, in comparison to their respective controls. Red and green colors represent increased or decreased expression levels, respectively, in comparison to controls, as reported by the scale. Genes were clustered **(A, B and C)** according to similarities in expression profiles. Details of the treatments are reported in Materials and Methods. Relative expression values for each gene and each treatment are provided in Figures 5, 6, 7, 8, 9 and Additional file 8.

Expression data of individual genes under each treatment are reported in Figures 5, 6, 7, 8, 9 and Additional file 8. Expression changes less than two-fold were not considered significant under these stresses. A comprehensive view of the expression profiles for all genes and all treatments is provided in Figure 10. Red or green colors represent the increase or decrease of transcript levels (fold-change) between treated and control samples, while black boxes represent non-modulated genes. The heat-map graphic output allows a glance of differences and similarities for a comparison of the effects of different treatments on a given gene. On the whole it is apparent that expression profiles can be grouped in 3 main clusters: cluster A) is a small group of VviMAPKKK genes with a prevalent trend of up regulation in most treatments, although with some notable exceptions following SA and drought treatment; the small cluster B) contains genes mostly down regulated by all treatments except drought stress, and C) a third cluster with a variable expression pattern in different treatments or time points, which have in common a strong up regulation of transcript levels in response to drought. Thus, from this general overview in can be suggested that water deprivation induces a peculiar expression profile of all MAPKKK genes, different from all other treatments considered.

Only a few genes diverge from these 3 main groups: VviMAPKKK47, which is almost invariably repressed, and VviMAPKKK38, which is strongly induced by *E. necator*, by SA and by H_2_O_2_ at the same time points of 4 and 24 h post-treatment.

Examining each stress condition separately, it can be observed that *E. necator* caused a strong increase of transcripts of most genes in cluster A (VviMAPKKK46,50, 32, 39, 34) and additionally of VviMAPKKK31 and 38; in particular, VviMAPKKK50 showed the highest transcript abundance, between 6 and 27-fold the control (Figures 5 and 10). A few genes (VviMAPKKK4, 54 and 51) are significantly down regulated by powdery mildew infection, especially VviMAPKKK54, while other genes are variably but slightly modulated. It can be observed however that many VviMAPKKK transcripts showed a decreased abundance at the very early time point (4hpt), a trend to a more or less pronounced increase afterwards, and a new decrease at the last collection time (72 hpt). This observation might correlate with the full establishment of infection and a possible down-regulation of defense responses, as it was reported in barley that powdery mildew can induce susceptibility in infected cells [39]. Although elucidating the exact roles of these VviMAPKKK genes in pathogen interactions requires further functional analysis, our findings provide the first gene-family-wide survey on the expression patterns of specific grapevine MAPKKK in pathological conditions, and these highly up- and down-regulated genes can be candidate genes for future investigations.

Salicylic acid and ethylene were chosen to investigate transcriptional responses of VviMAPKKKs to hormone treatments. Regarding the response to SA, Figures 6 and 10 shows a general picture of VviMAPKKKs down regulation for most genes at most time points, especially in Cluster A, in which VviMAPKKK34 and 46 show a decreased transcript abundance of more than 20 fold at 12 hpt, but increased afterwards, especially at 48 hpt. Clear increases could be detected for VviMAPKKK60 and 64 at 12 hpt and for VviMAPKKK34 and 50 at 48 hpt. Response to ethylene was striking for some VviMAPKKK genes (examples: VviMAPKKK39, with an increase above 10-fold at 8 hpt; VviMAPKKK60, induced by 9-fold at 12 hpt; VviMAPKKK34 and 46 with fold change values between 3 and 7 (Figure 7). The same VviMAPKKK60 and 64, responsive to SA at 12hpt, were also responsive to ethylene, especially at early time points, as well as VviMAPKKK52 and the paralogous couple VviMAPKKK 22/23. VviMAPKKK60 is the grape orthologue of *Arabidopsis* EDR1 gene. EDR1 exerts its negative control at a point of cross talk between ethylene and salicylic acid signaling [40]. Therefore it seems interesting that treatments with both SA and ETH may induce an increase of VviMAPKKKK60 at early time points, possibly as a regulatory mechanism to keep a balance between the two pathways. SA and ETH are involved in different signal transduction pathways and their action is often considered antagonistic, but may also cooperate in regulating defense responses [41]. Other MAPKKK genes were mostly down regulated by ethylene along the whole time course, such as VviMAPKKK30 and 36 (Figure 7). In some cases we observed a very similar expression profile in response to these two treatments, such as for VviMAPKKK30, 36 and 61, all down regulated and for VviMAPKKK27, 40 and 52 with variable but similar profiles along the time course of the experiments.

To further investigate the roles of VviMAPKKK genes, their expression levels were measured in response to the defense signaling compound H_2_O_2_. Several transcripts reacted strongly to this treatment along the whole experiment (Figures 8 and 10), especially VviMAPKKK39, which increased up to 50 fold at 24 hpt, and VviMAPKKK34, 46 and 50, with relevant increases at several collection times. A slight increase, of about 3-fold (24 hpt) was also observed for VviMAPKKK22, which shows a high degree of sequence similarity with *Arabidopsis* AtMAPKKK1 [MEKK1], involved in the regulation of reactive oxygen species (ROS) homeostasis [36]. The level of this grapevine transcript was down regulated by *E. necator* infection and induced by ethylene. Since plant genomes generally contain a relatively large number of MAPKKK genes, this differential regulation of MAPKKK gene expression may be a mechanism by which stress responses are fine tuned, although additional work is needed to confirm this hypothesis.

To analyze the responsiveness of VviMAPKKK to abiotic stress, drought treatments were performed. Drought is a major environmental factor limiting productivity and distribution of plants [42]. When grapevine plants were subjected to drought stress, almost all VviMAPKKK genes displayed significantly increased expression levels especially at 8 days post-treatment (Figures 9 and 10). Among all performed treatments, drought is the one which caused the strongest effect, with many VviMAPKKK transcripts increasing more than 10 fold, and 4 transcripts (VviMAPKKK22, 23, 51, and 54) showing a more than 20-fold increased expression (Figure 9). Down regulation, although rare, was also very strong in the case VviMAPKKK46, (up to 30-fold less than control). For several unregulated genes, the increase was sustained also in the last collection time of 12 dpt. The first collection time (4 days) did not reveal a significant modulation, suggesting that plants could maintain more or less stable mRNA levels in the first days after watering suspension, except for VviMAPKKK24, 34 and 46, strongly and early down regulated. Therefore, in comparison to other stresses, drought determines a peculiar response of strong transcriptional activation on most VviMAPKKKs, especially at 8 d after drought treatments, suggesting that grapevine MAPKKKs are very likely playing roles in response to drought stress. The involvement of MAPKKK in drought resistance was seldom investigated. Among the few examples there are a RAF-Like MAPKKK gene DSM1 with a function in drought and oxidative stresses signaling in rice [17] and a tobacco MAPKKK (NPK1) which enhanced drought tolerance in transgenic maize [43]. Our data provide the first insight into the possible involvement of grapevine MAPKKKs in this type of stress. However, more research is needed to determine the specific functions of the MAPKKK family by additional biological experiments, particularly to investigate why such a high number of VviMAPKKKs genes are responsive to drought.

## Conclusions

So far, MAPKKKs have only been investigated in some plant species including *Arabidopsis*, rice and maize, while no systematic analysis has been conducted in grapevine. In the present study we identified 45 MAPKKK coding genes in the *V. vinifera* genome, which were grouped into three subfamilies - MEKK, ZIK and RAF- and named according to their sequence similarity to *Arabidopsis* genes. The exon/intron structure, phylogeny and conserved domains strongly supported their identity as members of each subfamily. Furthermore, by exploring a previously published microarray analysis, we provided information about the expression profiles of all VviMAPKKK genes across different tissues and developmental stages. Finally, we experimentally determined the expression profiles of all grapevine MAPKKK genes in response to biotic and abiotic stress conditions, as well as hormone and H_2_O_2_ treatments. In conclusion, our work provides an inventory of VviMAPKKK genes potentially involved in environmental stresses, an initial insight into this important gene family and a number of possibly stress-related candidates for future functional analysis. This information provides a framework to unravel the biological roles of the VviMAPKKK genes family in grape and their regulatory mechanism, particularly their apparently wide implication in drought responses.

## Methods

### Identification of MAPKKK gene family in grapevine

MAPKKK protein sequences of *Arabidopsis* were used as query to search against the Proteome databases of *V. vinifera* (12X, V1) (http://genomes.cribi.unipd.it/grape) [44], Vitis-URGI (http://urgi.versailles.inra.fr/Species/Vitis) and NCBI databases (http://www.ncbi.nlm.nih.gov/) using the BLASTP program with e-values > 1E-5. The Protein family (Pfam) database (http://pfam.sanger.ac.uk/) was used to identify their protein domains using HMMER3.0. MAPKKK gene models were only accepted if they displayed the consensus sequences of dual-specificity protein kinases. Then, the online software SMART (http://smart.embl-heidelberg.de/) was used to further confirm the predicted MAPKKK protein sequences [45]. To increase efficiency of the search, we also investigated the results of a recently published paper [44] reporting a comparison of gene predictions between the 8× and the 12X genome coverages. Three additional MAPKKK genes were identified in the 8X genome, which are not present in the 12X version, but we could not find evidence that these 3 genes are actually expresses in the ESTs databases, and therefore they were not included in the family.

### Phylogenetic and motif analysis of the MAPKKK gene family in grapevine

The phylogenetic tree was constructed with the web service Phylogeny.fr (http://www.phylogeny.fr/) following the rules defined by the Grapevine Super Nomenclature Committee ([20]; Grimplet J. personal communication); bootstrap values below 70% were collapsed. The Arabidopsis nomenclature used following the MAPKKK nomenclature reported in the TAIR database (https://www.arabidopsis.org/) in which MEKK genes were named MAPKKK while RAF and ZIK genes were annotated maintaining the subfamily name. Multiple-sequence alignments of computationally predicted MAPKKK proteins belonging to each group of both *Arabidopsis* and grapevine (including characteristic sequence motifs) were performed using ClustalX program (version 1.83) [46] and GeneDoc (http://www.nrbsc.org/gfx/genedoc/). The PFAM database (http://pfam.sanger.ac.uk/) was used for identification of additional conserved motifs outside the MAPKKK domain. The protein sequences of *Arabidopsis* MAPKKKs were obtained from the TAIR (http://www.arabidopsis.org/) database.

### Gene structure, chromosomal location and gene duplication of grapevine MAPKKK genes

The information on MAPKKK genes in the grapevine genome, including accession number, chromosomal location, open reading frame (ORF) length, molecular masses, isoelectric point value (pI) and exon-intron structure were retrieved from the grapevine database (http://genomes.cribi.unipd.it/grape/). Gene duplication events of MAPKKK genes in grapevine were investigated based on three criteria: 1) The alignment length covered >90% of the longer gene; 2) The aligned region had an identity >90%; 3) Only one duplication event was counted for tightly linked genes [47]. GSDS (Gene Structure Display Server, http://gsds.cbi.pku.edu.cn/) was exploited to illustrate exon-intron organization of MAPKKK genes [48].

### Microarray data analysis of VviMAPKKK genes

To understand the spatial and temporal expression patterns of MAPKKK genes during the grapevine life cycle, the expression profiles of the MAPKKK genes was analyzed based on published high-throughput microarray data [31]. In the data sets, a total of 54 grapevine samples were included (bud, inflorescence, carpel, petal, pollen, berry, withering berry, leaf, root, seed, seedling, rachis, stem, and tendril), covering most organs at several developmental stages. The expression data were transformed in log_2_ values. The heat map was made with software MeV4.8 (http://www.tm4.org/mev/).

### Plant materials and stress treatments

PN40024 plants (*V. vinifera* inbred line of Pinot noir, sequenced genotype) were kindly provided by Dr. Anne-Françoise Adam-Blondon, INRA, France, and maintained in vitro on ^1^/_2_ MS medium supplied with 0.3 mg/L Indole 3-butyric acid (IBA, Sigma, USA), under a 16/8 h photoperiod (100 μmol m^−2^ s^−1^) at 25°C in the growing chamber. Five-week-old plants were used in all treatments.

For treatments with salicylic acid (SA, Sigma, USA), ethylene (ETH) (Ethephon, Sigma, USA) and hydrogen peroxide (H_2_O_2_, Sigma, USA), plants with fully expanded six to eight leaves per tissue-culture container (240 mL) were sprayed with 5 mM SA, 5 mM ethephon (as an ethylene donor) and 10 mM H_2_O_2._ All the chemicals were purchased from Sigma and dissolved in sterile distilled water. The samples (the second to fourth leaf counted from the top) were harvested at 4, 8, 12, 24, 48 and 72 h post-treatment. Each collected sample contained independent biological replicates (three independent treated plants) and three corresponding controls. For powdery mildew infection, a local strain of *Erysiphe necator* Schw. was maintained on PN40024 in a greenhouse. Young leaves of similar developmental stages were inoculated with *E. necator* by gently pressing and tapping conidia from infected leaves on healthy ones. The second to fourth leaves were sampled at 4, 8, 12, 24, 48 and 72 h post-inoculation. Each collected sample contained independent biological replicates (three independent treated plants) and three corresponding controls. For drought treatments, in vitro plants were acclimated to pots filled with a mixture of soil and sand (1:1) in the greenhouse until they grew to a length of about 40 cm with 14 leaves. The plants were watered thoroughly first and then not watered. The sixth leaves were collected at 4, 8 and 12 days after watering interruption, immediately frozen in liquid nitrogen and stored at −70°C until analysis. Every treated sample had a corresponding regularly-watered control. For each point, three independent biological replications (three independent plants) were sampled.

### Total RNA isolation and qRT-PCR expression analysis

Total RNA was extracted from the collected samples according to described previously with some modifications [19]. The concentration and purity of RNAs were examined by measuring optical density (OD) absorption ratio at 260 and 280 nm in a One Drop™ OD-1000 spectrophotometer (Thermo Fisher Scientific, USA). The RNA integrity was checked by electrophoresis on 1.0% agarose gels stained with ethidium bromide (EB). The first-strand cDNA templates were synthesized from1 μg total RNA using PrimeScritpt RT reagent Kit (TaKaRa, Japan) following the manufacturer’s instructions.

The expressions of VviMAPKKKs were examined by qRT-PCR using a SYBR Green method on an ABI 7300 Real-time PCR System (Applied Biosystems). Primers were designed by Beacon Designer 7.0 software (Premier Biosoft International, USA), based on the 3′-untranslated region and the 3′ terminal sequences of the coding region according to the predicted mRNA sequence. The amplification product of each reaction was about 200 bp. The reaction mix (total volume of 20 μL) contained: 10 μL SYBR® Premix Ex Taq™, 0.2 μL of each primer, 1 μL of template and 8.6 μL ddH_2_O. The PCR conditions were: pre-denaturation at 95°C for 30 s, followed by 40 cycles at 95°C for 20 s, 60°C for 20 s, and 72°C for 43 s. Grapevine actin gene (actin-101- like, VIT_12s0178g00200) was used as the internal normalize, which was previously shown to be a suitable internal standard [19]. The relative gene expression level was calculated according to the 2^-ΔΔCt^ method, where ^ΔΔ^Ct = (Ct_target gene_ - Ct_actin_) _treatment_ - (Ct_target gene_ - Ct_actin_) _control_ [49,50]. To visualize the relative expression levels data are presented as the mean fold changes between treated and control samples at each time point ± standard deviations (SDs). Mean values and standard deviations (SDs) were obtained from three biological replicates, each with three technical replicates. The gene specific primers are listed in Table S1 (Additional file 9).

### Statistical analysis

Statistical analyses were performed using the software SPSS version 13.0 (Chicago, IL) and Excel. All results were indicated as means ± standard deviations (SDs) based on Duncan’s multiple range test. P < 0.05 and P < 0.01 were taken as statistically significant or highly significant, respectively.

### Availability of supporting data

Here we are with the supporting data (including sequence data, microarray data and expression data) as additional files.

## Additional files

Additional file 1:
**MAPKKK amino acids sequence from V. vinifera and A. thaliana.**
Additional file 2:
**MAPKKK nucleotide coding region from V. vinifera and A. thaliana.**
Additional file 3: Table S2. The number of introns of VviMAPKKK genes. Additional file 4: Figure S1. Alignment of MAPKKK family from grapevine and Arabidopsis. The highlighted part shows the conserved signature motif. A: MEKK subfamily; B: ZIK subfamily. Additional file 5: Table S3. The additional domains of VviMAPKKKs. Additional file 6: Figure S2. Alignment of RAF subfamily from grapevine and Arabidopsis. The highlighted part shows the conserved signature motif. Additional file 7:
**VviMAPKKK genes fluorescence values.** All microarray expression data are available in the Gene Expression Omnibus under the series entry GSE36128 (http://www.ncbi.nlm.nih.gov/geo/query/acc.cgi?token=lfcrxesyciqgsjoandacc=GSE36128) and statistical analysis was applied according to Fasoli et al. [31]. Additional file 8:
**VviMAPKKK genes expression values under different biotic and abiotic stimuli.**
Additional file 9: Table S1. The primer sequences of the MAPKKK genes in grapevine for quantitative RT-PCR.

## References

[1] Hamel LP, Nicole MC, Sritubtim S, Morency MJ, Ellis M, Ehlting J, Beaudoin N, Barbazuk B, Klessig D, Lee J, Martin G, Mundy J, Ohashi Y, Scheel D, Sheen J, Xing T, Zhang S, Seguin A, Ellis BE (2006). Ancient signals: comparative genomics of plant MAPK and MAPKK gene families. Trends Plant Sci.

[2] Doczi R, Okresz L, Romero AE, Paccanaro A, Bogre L (2012). Exploring the evolutionary path of plant MAPK networks. Trends Plant Sci.

[3] Ichimura K, Shinozaki K, Tena G, Sheen J, Henry Y, Champion A, Kreis M, Zhang SQ, Hirt H, Wilson C, Heberle-Bors E, Ellis BE, Morris PC, Innes RW, Ecker JR, Scheel D, Klessig DF, Machida Y, Mundy J, Ohashi Y, Walker JC, Mapk G (2002). Mitogen-activated protein kinase cascades in plants: a new nomenclature. Trends Plant Sci.

[4] Rodriguez MC, Petersen M, Mundy J (2010). Mitogen-activated protein kinase signaling in plants. Annu Rev Plant Biol.

[5] Fiil BK, Petersen K, Petersen M, Mundy J (2009). Gene regulation by MAP kinase cascades. Curr Opin Plant Biol.

[6] Colcombet J, Hirt H (2008). Arabidopsis MAPKs: a complex signalling network involved in multiple biological processes. Biochem J.

[7] Pitzschke A, Schikora A, Hirt H (2009). MAPK cascade signalling networks in plant defence. Curr Opin Plant Biol.

[8] Rao KP, Richa T, Kumar K, Raghuram B, Sinha AK (2010). In silico analysis reveals 75 members of mitogen-activated protein kinase kinase kinase gene family in rice. DNA Res.

[9] Wankhede DP, Misra M, Singh P, Sinha AK (2013). Rice mitogen activated protein kinase kinase and mitogen activated protein kinase interaction network revealed by in-silico docking and yeast two-hybrid approaches. PLoS One.

[10] Gao L, Xiang CB (2008). The genetic locus At1g73660 encodes a putative MAPKKK and negatively regulates salt tolerance in Arabidopsis. Plant Mol Biol.

[11] Asai T, Tena G, Plotnikova J, Willmann MR, Chiu WL, Gomez-Gomez L, Boller T, Ausubel FM, Sheen J (2002). MAP kinase signalling cascade in Arabidopsis innate immunity. Nature.

[12] Zhang S, Klessig DF (2001). MAPK cascades in plant defense signaling. Trends Plant Sci.

[13] Nakagami H, Kiegerl S, Hirt H (2004). OMTK1, a novel MAPKKK, channels oxidative stress signaling through direct MAPK interaction. J Biol Chem.

[14] Adams-Phillips L, Barry C, Kannan P, Leclercq J, Bouzayen M, Giovannoni J (2004). Evidence that CTR1-mediated ethylene signal transduction in tomato is encoded by a multigene family whose members display distinct regulatory features. Plant Mol Biol.

[15] Frye CA, Innes RW (1998). An Arabidopsis mutant with enhanced resistance to powdery mildew. Plant Cell.

[16] Frye CA, Tang D, Innes RW (2001). Negative regulation of defense responses in plants by a conserved MAPKK kinase. Proc Natl Acad Sci U S A.

[17] Ning J, Li X, Hicks LM, Xiong L (2010). A Raf-like MAPKKK gene DSM1 mediates drought resistance through reactive oxygen species scavenging in rice. Plant Physiol.

[18] Jaillon O, Aury JM, Noel B, Policriti A, Clepet C, Casagrande A, Choisne N, Aubourg S, Vitulo N, Jubin C, Vezzi A, Legeai F, Hugueney P, Dasilva C, Horner D, Mica E, Jublot D, Poulain J, Bruyere C, Billault A, Segurens B, Gouyvenoux M, Ugarte E, Cattonaro F, Anthouard V, Vico V, Del Fabbro C, Alaux M, Di Gaspero G, Dumas V (2007). The grapevine genome sequence suggests ancestral hexaploidization in major angiosperm phyla. Nature.

[19] Wang G, Lovato A, Liang YH, Wang M, Chen F, Tornielli GB, Polverari A, Pezzotti M, Cheng ZM (2014). Validation by isolation and expression analyses of MAPK gene family in grapevine (Vitis vinifera). Aust J Grape Wine Res.

[20] Adam-Blondon F, Grimplet J, Adam-Blondon AF, Bert PF, Bitz O, Cantu D, Cramer G, Pezzotti M, Rombauts S (2014). Towards the Improvement of the Gene Centered Information in Grapevine Genomics.

[21] Dereeper A, Guignon V, Blanc G, Audic S, Buffet S, Chevenet F, Dufayard JF, Guindon S, Lefort V, Lescot M, Claverie JM, Gascuel O (2008). Phylogeny.fr: robust phylogenetic analysis for the non-specialist. Nucleic Acids Res.

[22] Shiu SH, Bleecker AB (2003). Expansion of the receptor-like kinase/Pelle gene family and receptor-like proteins in Arabidopsis. Plant Physiol.

[23] Zhang Y, Gao M, Singer SD, Fei Z, Wang H, Wang X (2012). Genome-wide identification and analysis of the TIFY gene family in grape. PLoS One.

[24] Cao J, Huang J, Yang Y, Hu X (2011). Analyses of the oligopeptide transporter gene family in poplar and grape. BMC Genomics.

[25] Kong Q, Qu N, Gao M, Zhang Z, Ding X, Yang F, Li Y, Dong OX, Chen S, Li X, Zhang Y (2012). The MEKK1-MKK1/MKK2-MPK4 kinase cascade negatively regulates immunity mediated by a mitogen-activated protein kinase kinase kinase in Arabidopsis. Plant Cell.

[26] Pitzschke A, Djamei A, Bitton F, Hirt H (2009). A major role of the MEKK1-MKK1/2-MPK4 pathway in ROS signalling. Mol Plant.

[27] Nakagami H, Soukupova H, Schikora A, Zarsky V, Hirt H (2006). A Mitogen-activated protein kinase kinase kinase mediates reactive oxygen species homeostasis in Arabidopsis. J Biol Chem.

[28] Fung RW, Gonzalo M, Fekete C, Kovacs LG, He Y, Marsh E, McIntyre LM, Schachtman DP, Qiu W (2008). Powdery mildew induces defense-oriented reprogramming of the transcriptome in a susceptible but not in a resistant grapevine. Plant Physiol.

[29] Lin Z, Alexander L, Hackett R, Grierson D (2008). LeCTR2, a CTR1-like protein kinase from tomato, plays a role in ethylene signalling, development and defence. Plant J.

[30] Kong X, Lv W, Zhang D, Jiang S, Zhang S, Li D (2013). Genome-wide identification and analysis of expression profiles of maize mitogen-activated protein kinase kinase kinase. PLoS One.

[31] Fasoli M, Dal Santo S, Zenoni S, Tornielli GB, Farina L, Zamboni A, Porceddu A, Venturini L, Bicego M, Murino V, Ferrarini A, Delledonne M, Pezzotti M (2012). The grapevine expression atlas reveals a deep transcriptome shift driving the entire plant into a maturation program. Plant Cell.

[32] Yang X, Tuskan GA, Cheng MZ (2006). Divergence of the Dof gene families in poplar, Arabidopsis, and rice suggests multiple modes of gene evolution after duplication. Plant Physiol.

[33] Ichimura K, Casais C, Peck SC, Shinozaki K, Shirasu K (2006). MEKK1 is required for MPK4 activation and regulates tissue-specific and temperature dependent cell death in Arabidopsis. J Biol Chem.

[34] Suarez-Rodriguez MC, Adams-Phillips L, Liu Y, Wang H, Su SH, Jester PJ, Zhang S, Bent AF, Krysan PJ (2007). MEKK1 is required for flg22-induced MPK4 activation in Arabidopsis plants. Plant Physiol.

[35] Clark KL, Larsen PB, Wang X, Chang C (1998). Association of the Arabidopsis CTR1 Raf-like kinase with the ETR1 and ERS ethylene receptors. Proc Natl Acad Sci U S A.

[36] Zhao Y, Wei T, Yin KQ, Chen Z, Gu H, Qu LJ, Qin G (2012). Arabidopsis RAP2.2 plays an important role in plant resistance to Botrytis cinerea and ethylene responses. New Phytol.

[37] Hamel LP, Miles GP, Samuel MA, Ellis BE, Seguin A, Beaudoin N (2005). Activation of stress-responsive mitogen-activated protein kinase pathways in hybrid poplar (Populus trichocarpa x Populus deltoides). Tree Physiol.

[38] Overmyer K, Brosche M, Kangasjarvi J (2003). Reactive oxygen species and hormonal control of cell death. Trends Plant Sci.

[39] Yahata E, Sugai K, Penjore K, Hanboonsong Y, Takada Y, Nishiguchi M, Yamaoka N (2009). Susceptibility-inducing factor (suppressor) from Blumeria graminis f. sp hordei has no effect on the primary infection of the fungus. Physiol Mol Plant P.

[40] Wang KL, Li H, Ecker JR (2002). Ethylene biosynthesis and signaling networks. Plant Cell.

[41] Tang D, Christiansen KM, Innes RW (2005). Regulation of plant disease resistance, stress responses, cell death, and ethylene signaling in Arabidopsis by the EDR1 protein kinase. Plant Physiol.

[42] Shi J, Zhang L, An H, Wu C, Guo X (2011). GhMPK16, a novel stress-responsive group D MAPK gene from cotton, is involved in disease resistance and drought sensitivity. BMC Mol Biol.

[43] Shou H, Bordallo P, Wang K (2004). Expression of the Nicotiana protein kinase (NPK1) enhanced drought tolerance in transgenic maize. J Exp Bot.

[44] Grimplet J, Van Hemert J, Carbonell-Bejerano P, Diaz-Riquelme J, Dickerson J, Fennell A, Pezzotti M, Martinez-Zapater JM (2012). Comparative analysis of grapevine whole-genome gene predictions, functional annotation, categorization and integration of the predicted gene sequences. BMC Res Notes.

[45] Letunic I, Doerks T, Bork P (2012). SMART 7: recent updates to the protein domain annotation resource. Nucleic Acids Res.

[46] Thompson JD, Gibson TJ, Plewniak F, Jeanmougin F, Higgins DG (1997). The CLUSTAL_X windows interface: flexible strategies for multiple sequence alignment aided by quality analysis tools. Nucleic Acids Res.

[47] Gu Z, Cavalcanti A, Chen FC, Bouman P, Li WH (2002). Extent of gene duplication in the genomes of Drosophila, nematode, and yeast. Mol Biol Evol.

[48] Guo AY, Zhu QH, Chen X, Luo JC (2007). GSDS: a gene structure display server. Yi Chuan.

[49] Ye X, Kang BG, Osburn LD, Li Y, Zong-Ming C (2009). Identification of the flavin-dependent monooxygenase-encoding YUCCA gene family in Populus trichocarpa and their expression in vegetative tissues and in response to hormone and environmental stresses. Plant Cell Tiss Org.

[50] Udvardi MK, Czechowski T, Scheible WR (2008). Eleven golden rules of quantitative RT-PCR. Plant Cell.

