# Supplementary material for: Genome-wide identification and analysis of mitogen activated protein kinase kinase kinase gene family in grapevine (Vitis vinifera)
Source: BMC Plant Biol. 2014 Aug 27;14:219. doi: 10.1186/s12870-014-0219-1 (PMC4243721; doi:10.1186/s12870-014-0219-1)
Supplement: Additional file 3: Table S2. — The number of introns of VviMAPKKK genes. [file 12870_2014_219_MOESM3_ESM.pdf]

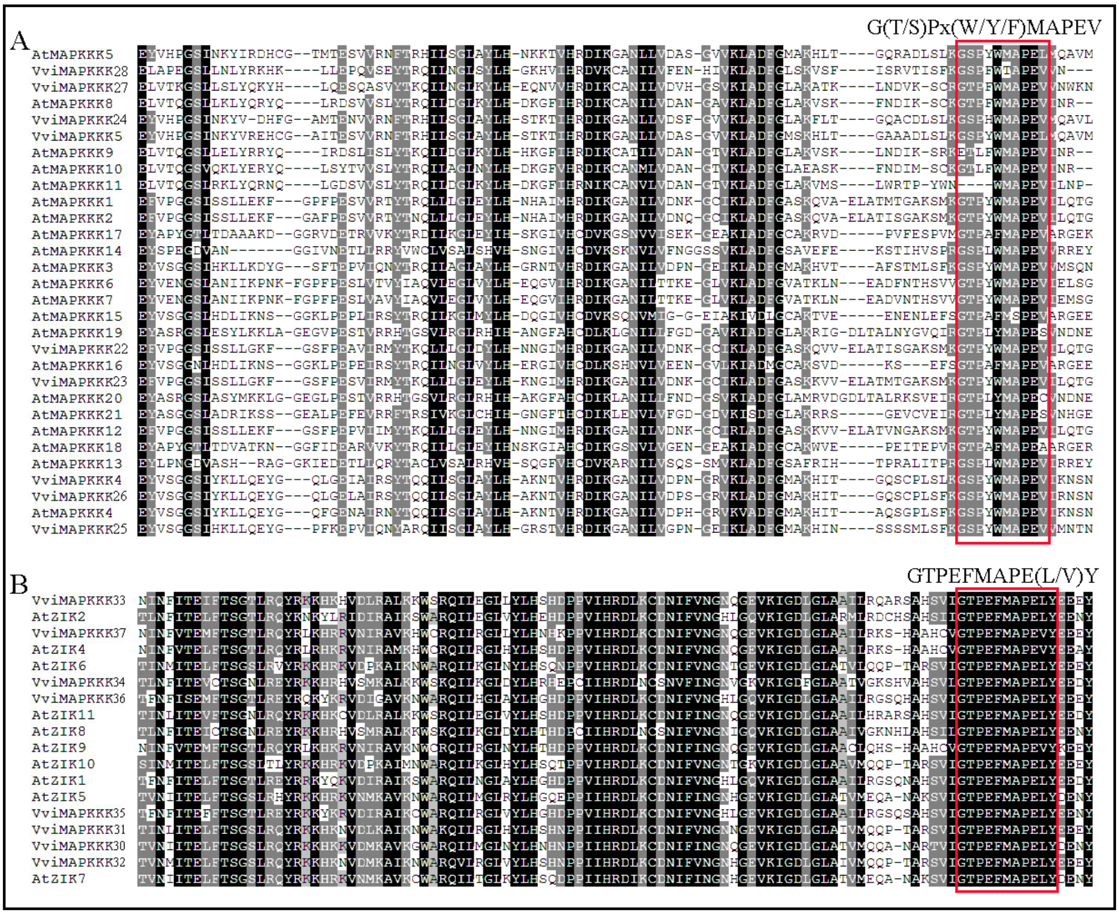
A
G(T/S)Px(W/Y/F)MAPEV
B
GTPEFMAPE(L/V)Y
