# Supplementary material for: Genome-wide identification and analysis of mitogen activated protein kinase kinase kinase gene family in grapevine (Vitis vinifera)
Source: BMC Plant Biol. 2014 Aug 27;14:219. doi: 10.1186/s12870-014-0219-1 (PMC4243721; doi:10.1186/s12870-014-0219-1)
Supplement: Additional file 4: Figure S1. — Alignment of MAPKKK family from grapevine and Arabidopsis. The highlighted part shows the conserved signature motif. A: MEKK subfamily; B: ZIK subfamily. [file 12870_2014_219_MOESM4_ESM.pdf]

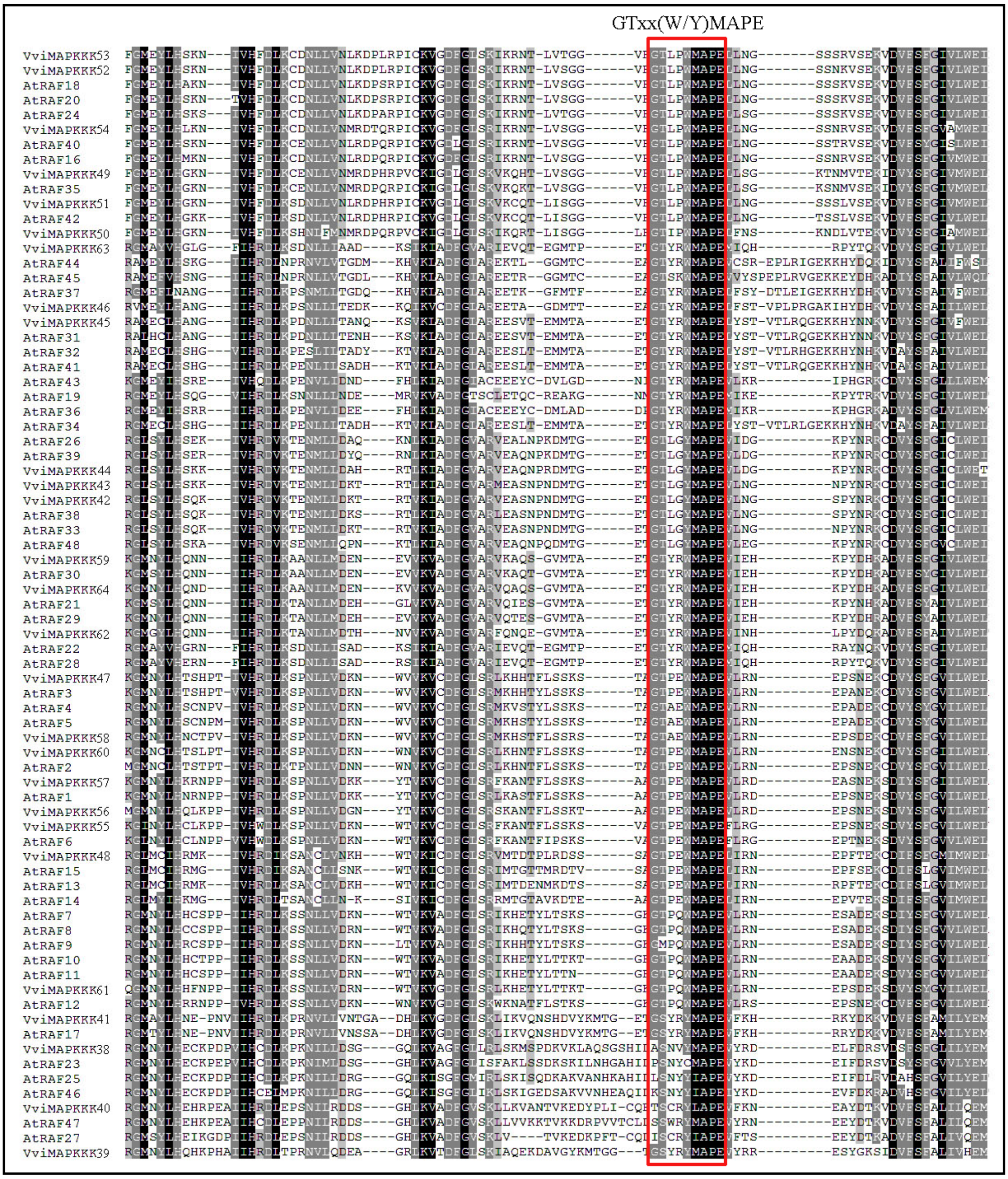
GTxx(W/Y)MAPE
VviMAPKKK53
VviMAPKKK52
AtRAF18
AtRAF20
AtRAF24
VviMAPKKK54
AtRAF40
AtRAF16
VviMAPKKK49
AtRAF35
VviMAPKKK51
AtRAF42
VviMAPKKK50
VviMAPKKK63
AtRAF44
AtRAF45
AtRAF37
VviMAPKKK46
VviMAPKKK45
AtRAF31
AtRAF32
AtRAF41
AtRAF43
AtRAF19
AtRAF36
AtRAF34
AtRAF26
AtRAF39
VviMAPKKK44
VviMAPKKK43
VviMAPKKK42
AtRAF38
AtRAF33
AtRAF48
VviMAPKKK59
AtRAF30
VviMAPKKK64
AtRAF21
AtRAF29
VviMAPKKK62
AtRAF22
AtRAF28
VviMAPKKK47
AtRAF3
AtRAF4
AtRAF5
VviMAPKKK58
VviMAPKKK60
AtRAF2
VviMAPKKK57
AtRAF1
VviMAPKKK56
VviMAPKKK55
AtRAF6
VviMAPKKK48
AtRAF15
AtRAF13
AtRAF14
AtRAF7
AtRAF8
AtRAF9
AtRAF10
AtRAF11
VviMAPKKK61
AtRAF12
VviMAPKKK41
AtRAF17
VviMAPKKK38
AtRAF23
AtRAF25
AtRAF46
VviMAPKKK40
AtRAF47
AtRAF27
VviMAPKKK39
