# Supplementary material for: Genome-wide identification and analysis of mitogen activated protein kinase kinase kinase gene family in grapevine (Vitis vinifera)
Source: BMC Plant Biol. 2014 Aug 27;14:219. doi: 10.1186/s12870-014-0219-1 (PMC4243721; doi:10.1186/s12870-014-0219-1)
Supplement: Additional file 5: Table S3. — The additional domains of VviMAPKKKs. [file 12870_2014_219_MOESM5_ESM.pdf]

>VviMAPKKK4 VIT_02s0025g03370

ATGCCTTCATGGTGGGGGAAATCTTCATCCAAAGAAGTAAAGAAGAAGGAAAACCGGGAAAGTTTCATTGATTCAATACAC
CGGAAATTCAGGACTGTATCTGAAGAGAAGTGCAACAATAGATCAGGAGCTTCCCAAAGACACTGTGGTGACACTGTCTCA
GAGAAGGAATCTCGATCCAGAGCACAATCAAGATCACCATCTCCCTCCACAAAAGTATCACGCTGTCAAAGTTTTGCAGAA
AGGCCTCATGCCCAACCACTTCCACTCCCTGGACCGCACCTTACGAGTGTAGTGCGTACTGACTCTGGAATCAATGCATCAA
AGAAACAAGGATTGGTTGAAGGCTCCAAGACACAGATGGTTTTGCCCCTGCCAAGACCTGGATATGTTGCAAACAGGCTAG
ATCCTACAGATGCTGAGGGGGATCTAGCCACTGCTTCTGTGTTTAGTTATAGTTCCATTGATAGTGAAGATCCATCTGAGTCAC
GCCTTCTCAGCCCCCAGGCATCTGATTATGAGAATGGAAACAGAACCACTATGAACAGCCCTTCCAGCGTAATGCACAAGGA
TCAGTCTCCTGTTCTCACCCCAAGGAAGCCAAGAGAAGCATTGAGGCCAGCTAATCTTTTGTTAAACAATCAGATTCACTCT
ACATCACCTAAATGGGTACCTTTAAGCACTCATGTGCCAAATTTTCCAGTTCCTCAGAATGGCGCTTTCTGTAGTGCTCCAGA
CAGCTCGATGTCAAGTCCTTCTAGAAGTCCAATGAGATTATTTAGCCCTGAGCAAGTCATGAATTCTAGTTTCTGGACGGGAA
AGCCTTATGCTGATATAGCTTTGCTTGGATCTGGACACTGTTCTAGTCCAGGTTCAGGTCACAATTCTGGACATAATTCAATAG
GAGGAGATATGTCAGGACAGCTGTTTTGGCCGCACAGCAGGTGTAGCCCTGAGTGTTCTCCAATACCAAGTCCCAGAATGA
CAAGCCCTGGTCCCAGCTCCAGAATACAGAGTGGTGCTGTCACCCCTCTGCATCCACGAGCTGGAGCAGCTGCTGCAGAGT
CTCCTACCAACCGACCTGATGATGGAAAGCAACAAAGCCACCGATTGCCCCTTCCACCGATAACAATTTCTAATTCTTGTCCT
TTTTCTCCTACATATTCTACATCAACAACTCCCTCAGTACCACGAAGTCCTGGTAGGGCGGAAAACCCAATCAGCCCTGGATC
ACGCTGGAAGAAGGGTCGGCTCTTAGGGAGAGGCACATTTGGGCATGTATATCTTGGGTTTAATAGTGAAAGTGGGGAGATG
TGTGCAATGAAGGAGGTTACATTATTTTCAGATGATGCGAAGTCAAAGGAAAGTGCACAGCAGCTGGGCCAAGAAATTTCG
CTCCTCAGTCGCTTACGTCATCCAAATATAGTGCAATACTATGGATCTGAGACAGTGGATGACAAACTCTACATATATTTGGAA
TATGTATCTGGTGGTTCCATATATAAACTTCTTCAAGAGTATGGCCAGCTTGGTGAAATAGCTATTCGTAGCTATACTCAACAA
ATTCTGTCAGGGCTTGCATATTTGCATGCTAAAAACACTGTCCATAGGGACATCAAAGGAGCAAATATACTGGTGGATCCCAA
TGGCCGTGTGAAATTGGCAGATTTTGGAATGGCAAAGCATATTACTGGACAATCTTGTCCGTTATCCTTAAAGGGGAGCCCTT
ACTGGATGGCACCTGAGGTCATAAAGAACTCAAATGGCTGTAATCTTGCAGTCGATTTATGGAGTCTTGGGTGCACAGTTTT
GGAGATGGCTACAACAAAACCACCTTGGAGCCAGTATGAAGGGGTTGCTGCTATGTTTAAGATTGGTAACAGTAAAGAACTT
CCCACAATACCTGATCATCTCTCAGAGGAGGGCAAGGACTTTGTAAGGCAGTGCTTGCAACGCAACCCATTGCATCGTCCCA
CAGCTGCTTGGCTATTGGAGCACCCTTTTGTTAGAAATGCTGCGCCTCTGGAAAGACCTAGTCTTAGTTCTGAGCTTGAACC
ACCACCTGCAGTTACAAATGCTGTCAGATCCATGGCCATTGGACACACAAGAAATGTTTTAGAGTCTGAAGGAGTGGCTATC
CATCAGTCTAGATGTTCAAAAACTGGTTCAGGATCCAGTGATACCCATACCCCAAGGAACTTATCAAGCCCTGTTTCTCCTAT
TGGGAGCCCTCTTCTGCATTCAAGATCACCACAACATATGAGTGGGAGGATGTCTCCCTCTCCCATATCCAGTCCTCGCACCA
CATCTGGTTCATCCACGCCTCTCAGTGGGGGTAGTGGTGCCATACCTTTTCATCATCCAAAGCCAATAAACTACATGCATGAA
GGCATCGGAATCATCCCAAGATCCCAGAGCAGTCTCTATGCTAATGGCAGCAGCTCCTACCAAGATCCCCAGCCTGATCTTTT
TCGAGGGATGCCACAAGTATCTCATGTTTTCCGGGAGATGATTTCATCTGAAAGTGGCAGTTTTGGAAATCAGTTTGGACGA
CCTGTCCATGGAGACCCCAGGGACCTGTGTGATGCACAATCAGTCTTGTCTGATCGCGTAGCTCAGCAGCTTTTGAGGGACC
ATACAAACTTGCACCTTTCTCTGGACCTAAATCCTGGTTCTCCTATGCTCACGCGCACTAATGGAATTTAA

>VviMAPKKK5 VIT_00s0567g00010

ATGCCTTGGTGGCAGAACATTGCCTTCTCTTCTCCCTCTTCCTCCTCTTCCACGTCTTCGTCGCGGCGCCGTCTCACCCGTGC
CAGGAAGCTCCGTCATGTGCGGGGCAATCACATTGACGCACTTGTGAGATCGCGAAGTTCAGCGGAGCCCAAGGATCTGTT
GCCGTTGCCCTCTTCGTCTTCCAGTTTCGACCATTCGTCTGAGCGCCTGTCGTCCGTGGTGCCGCATCCGCTGCCGCGCCCG
GAATTGGCTCTGCTTTTTCGTCGCAACGGCGGCTCCAATTCGAATTTGAATTGTGATCGCCCTCTTCCTTCGCCTAAAGAGGG
CCTGAGCAGAGGCTTTGAGGACCGGGATAAAGGAAATAGTTTTGTAGGAGATGGGAATGGAGAGGTGGCGGTGTCAAGCA
CCGCAACTGGCAGTAAGGTTGGTTACAATGAAGCCCGAAAGAGTATGGAGCAATTTGACACACTGTCATCTAGATACCTGCC
TCAAGGTCAAAATAGTGTGAAAAACAATCGAGTCAACTTCAAGCTCGATGTTCCTACTAGGAGTGCTCCAACTAGCTCCCTC
TCAAGTCCTGCAGTAAGTCCACAAAGAACAAGTCCTGGGAATTTGTTCCTTTCTCAAAATGTGTCTCCTCAAGTATTTCAAG
GTTGGTCAGCACCAGAGATGCCTGTCTTTGATATGGTCACAGGCTTCACTCCTCAAATGTCCCCTGAGCAAACCATGTTTAGT

ACGGATAATTCTCCCCTTCACAGTCCAACAGTAAAAAGTCCCCATGTAAATCCCCGAAGCCCCAGTGGACCTGCATCACCAT
TGCATCCAAAAATATCCCTTGAAACCTCAACAGCACGGCGTGAAAATAATAGCCATGCCAATGTTCATCGGTTGCCTCTTCCT
CCAGGAGTGGTGGCACCTCCACAGGCATCTTCTATTCATCCTGTTATTGCTAAAACAGAGTCATTTCCAATGACAACCCAATG
GCAAAAAGGAAAGCTCATTGGGCGTGGAACATTTGGAAGTGTTTATGTTGCCACCAATCGAGAAACTGGAGCTCTATGTGC
AATGAAAGAAGTTGAATTGCTTCCTGATGACCCAAAATCTGCAGAGAGTATAAAGCAGTTAGAGCAGGAAATTAAAATTCTT
AGCCAACTAAAGCATCCAAACATTGTGCAGTATTTTGGTAGTGAAACAGTTGAAGACCGGCTTTATATATACCTGGAATATGT
TCATCCTGGTTCAATTAATAAATATGTCCGTGAACATTGTGGAGCTATTACAGAATCTGTTGTTCGCAATTTTACTCGCCATATT
CTTTCTGGGCTGGCTTACTTACACAGCACAAAGACAATACACAGAGACATCAAAGGGGCTAATTTGCTTGTTGATGCATCTG
GAGTTGTTAAGCTTGCTGACTTTGGGATGTCTAAACATCTAACTGGAGCAGCAGCTGATCTTTCTCTAAAGGGAAGTCCATA
CTGGATGGCTCCAGAGCTCATGCAAGCTGTGATGCAGAAAGATCATAGTTCTGATCTTGCCTTTGCTGTTGATATATGGAGTT
TGGGTTGTACTATTATTGAAATGCTGAATGGAAAACCTCCTTGGAGTGAGTATGAGGGGGCTGCCGCTATGTTCAAGGTTATG
AGAGAATCCCCACCCATACCTAAAACATTGTCGTCAGAAGGCAAGGATTTCTTACGTTGTTGCTTTCGAAGAAATCCTGCAG
AGAGGCCTCCAGCTATCAAGTTACTGGAACATCGTTTCCTGAAAAACTCAACACAGCTGGATGTGCCACTTCTCACCCAAGC
ATTTAGTGGAATGAAATTGCCGGATAAAGCTAACAAATCAAGAGAAAAGTCGAATGATAGAGTTGATCCGGTGCCAATATCT
CCAAGGAAAAAAACCTCAAAAGGAAAAAAGGCCAGTGGAACTGGCCAACAATCTCATCGTGAAACTTCCGACTTAACAGT
GGCCTCTCATCATTCCCCACGCTCTACCCTCGAGGCCCTTCCTAGTTTATCTCCTCCGCATTCGGGTCAGAGAGCATATCATCT
CAGCCCTCCTGCAAATGTTCCCAGCCCTATTAATTATGGTGCTAAAAAGAAGCGTACGTGGGGGTGA

>VviMAPKKK22    VIT_14s0128g00430

ATGCAAGACATTTTCGGATCAGTTCGCCGGTCGCTCGTCCTCCGGTCACCCGATGGCGACGACACTTCCCCCGGAACCCTAG
TCGACAAAATCAACTCTTGCATTCGCAAATCCAGAGTCTTCTCCAGAGCCTCCCCTCCGCCACTAATTCCCAAAGACGCCAC
TGCGCCTTCGATCCGATGTCGAAAAGGGGAGCTGATCGGCTGTGGCGCCTTCGGTCGTGTCTATATGGGCATGAATCTCGATT
CTGGAGAGCTTATCGCCGTCAAACAGGTTTTGATCACCACAAGCAATGCTACAAAAGAGAAGGCACAGGCTCATATTAGGG
AACTTGAAGAAGAAGTGAAGCTTCTTAAGAATCTCTCTCATCCAAACATTGTTAGGTATTTGGGTATAGTCAGGGAGGAGGA
GACCTTGAATATTTTGTTGGAATTTGTTCCTGGTGGATCCATCTCATCACTTTTGGGGAAATTTGGATCTTTCCCTGAGGCTGT
GATAAGAATGTACACAAAACAATTATTATTGGGACTAGATTATCTTCACAACAATGGAATTATGCACAGGGACATCAAGGGGG
CAAACATCCTCGTCGATAATAAAGGATGCATTAAACTTGCAGATTTTGGTGCTTCCAAGCAGGTTGTGGAGCTGGCTACCATT
TCAGGTGCCAAGTCCATGAAAGGCACCCCGTATTGGATGGCTCCGGAAGTCATTCTCCAGACAGGCCATAGCTTCTCGGCTG
ATATATGGAGTGTTGGATGTACCGTTATTGAGATGGCCACTGGAAAGCCTCCTTGGAGCCAAAAGTACCAAGAGGTTGCTGC
TCTCTTTTATATTGGGACAACAAAGTCTCATCCGCCAATTCCTGCACATCTCTCTGCTGAGGCAAAAGATTTTCTACTGAAGT
GTTTACAAAAGGAACCAGATTTGAGGCCAGCTGCCTATGAGTTGCTGAAGCATCCATTTGTTACTGGAGAGTATAACGAAGC
TCAACTTGCTCTTCAAACTTCCGTTATGGAAAATTCTGAAACTCCTGTATCAACTTCTGAGGAAAACTTGCCAAATCCTCAAA
TGACAAATGACTCTAGCTTGCTGGATGTCTGTGAGATGGGGAATTTGAGCTGCTCAACTGGATATCCTGAGAATTCAGAATC
CAAACCTAATTGGGGAACAAGCAGCAGTATTGACATGTGTCAAATTGATGACAAAGATGATGTTATGATGGTTGGGGAAGTA
AAGTTCAATTCTATCCTGCTGCCTGATAACTTTAATAAGAGTTTCAATCCGATTTCTGAGCCCTCACATGATTGGAGCTGTGAA
ATTGATGGAAATCTACGACCAGAACATAGTGGAATGGACATGGACACCAATCCATCTCTTGATATGCCTGCTGGTAGCTCTGG
CATATCCAATAAGGGAGGCAATGATTTCTCATTTCCTTGTGGGCCATCAGTATCTGAGGATGATGATGAAGTTACTGAGTCAA
AAATCAGAGACTTCCTGGATGAGAAGGCCATTGAACTGAAGAAACTGCAAACGCCATTATATGAGGAGTTCTACAACACTTT
GAATGCTGCATGCTCTCCAAGTGTTGCTGAGCGTACACAGGATGAAAATGTTATAAACTACTTGAAATTGCCTCCTAAAAGC
CGTTCACCAAGTCGGGCTCCAATTGGAACCCCATCTGCGGCTGTTGATTCCACTAGTACTGCGAGCCCTGGGAGTTGTGGCA
GACGAATGTCAAATGTTGGCAATGCAGGTGATCAAACTGCACAGGACACCCCGTCACCTCCACATAATGACTGGAAAGGCC
TTCTTGTTGATTCTCAGCAGGAACCTAATAGCCCAAGTGTGAGCTTTTATGAGATACAGAGGAAGTGGAAGGAGGAGCTTGA
CCAAGAACTAGAGAGAAAACGAGAGATGATGCGCCAAGCAGGGAAAACATCATCACCAAAGGATCGAGCTTTGAATCGGC
CAAGAGAACGTTCAAGGTTTGCATCTCCAGGCAAATGA

>VviMAPKKK23    VIT_05s0020g02910

ATGCAAGACATATTCGGATCAGTTCGCCGATCTCTAGTATTTCGACCAGCATCCGGCGAAGATGGAGAATATGGAGGATATGG
AGGCCCGGTGGAGAAAATATGGTCCAGCATTCGCAAATCTGGAATCGGACTCTTTTCAAGACAGTCTGTTCGTGCGTTGCCT
CCGATCCCCAAAGATGAGGCGCCTTCGATCCGGTGGAGGAAAGGCGAGTTAATCGGGTGTGGCGCATTCGGGCGAGTCTATA
TGGGGATGAATCTTGATTCCGGAGAGCTTCTTGCCATTAAGCAGGTTTCAATTGCTGCGAATAGTGCTTCAAAAGAAAAAAC
GCAGGCTCACATCAGAGAGCTTGAGGAAGAAGTGAAGCTTCTGAAGAATCTCTCACATCCAAACATTGTTAGATATTTGGGA
ACTGCAAGAGAAGATGAGTCATTGAACATTTTGTTGGAGTTTGTGCCGGGAGGGTCCATTTCGTCACTCTTAGGAAAATTTG
GATCCTTCCCTGAGTCTGTTATAAGAATGTATACAAAACAGCTATTATTAGGATTGGAATACCTTCACAAAAATGGTATTATGC
ATAGGGACATAAAGGGGGCAAATATCCTGGTTGATAATAAGGGATGCATTAAACTTGCAGACTTTGGTGCATCTAAGAAAGT
TGTTGAGCTGGCTACTATGACTGGTGCCAAGTCAATGAAGGGTACTCCATATTGGATGGCTCCTGAAGTTATTCTCCAGACTG
GACATAGCTTCTCTGCTGATATATGGAGTGTTGGATGTACTGTTATTGAGATGGCTACAGGAAAACCTCCTTGGAGCCAGCAA
TATCAGGAGGTTGCTGCTCTCTTCCATATAGGGACGACTAAATCTCATCCTCCAATTCCAGAGCATCTTACTGCAGAGGCAAA
AGATTTTCTATTGAAATGTTTACAAAAGGAACCAAACTTGAGGCCTGCTGCTTCAGAATTGCTGCAGCATCCATTTGTATCTG
GGGAGTATCAGGAACCTCATCCAGTGTTTCAAACTTCAGTTATGGAAAATTCTGGAAACATGATGGCAACATCTGAGACAGA
CCTTAAGAGCTTCCCAAACCCAGTAATCAGAAGGTCAAACTGCACAAGCTCAAATAATGTTTGTAACATGGGTAGTGTGAGA
TGCTCAACTGTATACCCTGAGAAGTTTTCAGGAACTGGACCCCTTTGGGGAGCAAACAGTTGTGATGATGACATGTGTCAGA
TTGATGATAAAGACGATTTTATAGTTCATCAATCAGTAAAGTTCGGTTCAGCCATTCTTTCTGATCATCTAAATAAGAGTTTTA
ACCCTATGTGTGAGCCCACTGATGATTGGCCATGCAAATTTGATGAGAGTCCAGAGTTGACTAAGAGCGGAGCAAACTTATC
CTCTCACCAAACCATTAGTAAGCCTGCTGGCAGCCCTAGAGCATCTAATGAGAGGGAGAATGACTTCACATTTCCATGTGGG
CCATTAGTTGGAGATGATGATGATGAAGTTACAGAGTCAAAAATTAGAGCCTTTCTAGATGAAAAGGCCTTAGATTTGAAGA
AGCTTCAAACGCCACTATATGAAGAATTTTACAACAGCACATTGAATGCAGCTGGTCCTCCAAGTGCTGTTGGGAAATCACA
TGACAATGTTACAAATTTTTTGAACTTACCACCTAAAAGCAGGTCACCAAATCAAACCCCTACCAGAAGGTTGTCAGCTGCT
GTTGATGCTGCCTGTATTTCCAACTCTGCAAATCATACAAAACGTGGATTAAATGTTGGCACTATAAGTGATAGAACTTTGAA
GGAAATTCAGTCGCCTCAGCTGAGTGAATGGAAAGAGCTTCTTCTTGATGCCCAGCAGGAACCAGTCAGTTTAAGCACAAA
CTTCTCTGAGAGACGAAGAAAATGGAAAGAAGAGCTTGATCAAGAGCTTGAGAAGAAACGAGAATTGTTGCGCCAGGCAG
GTGTTGGAGGGAAGACAGCATCTCCAAAGGATCAGATTCTAAATCGGCCAAGAGAGCGGTTAAGATTTGCATTCCCAGGAA
AATGA

>VviMAPKKK24    VIT_18s0001g11240

ATGAAATTGTTATGGTGCAGTGTTTATGCTCGTGGATCTACCTGGAAGAACACAGATCATCTTGCCACAAAATCAGCTAATTC
GCCAAGGGGATTCTCTCAAGATCTAAATGATGAAGCTTGTCCTTACAACTTACGGGTGAATATTCCTGCTAGGAGTGCTCCAA
CAAGTGGTTTCTCAAGTCCTGCTCTTAGCCCACAAAGATTTAGCCCTGGGGAACGTTTACCATCCTCCTATGCAGCTATTCAA
GACTTTCAGTCACCCGGCTTTGATAGGCTTCCAGGTTGTTCTTCACAGATGTCACCATTGAAAACTCCGCACACTCCCGATTA
TTCTCCGCTTCATAGCCCAACAGTTCAAAGTCCCTGCATTAGCCCCTGTCTTAGCCCCAAAAGTCCAACTGGTATTGCATTTT
CTTTGTATCCTAAATTGCTCCCAGGGAGTCATGTAACATGGCCTGAAAAGAATGGTCATGTCACTGTCCATCCATTGCCCCTT
CCTCCTATAGCTTTAATGCCATCAGAATTACCCTTGCCTCCAAAAGCTTTAACACCATCAGAGTCAGCTATCAGTCATCACACT
GCAGAGAAACCAAATGTACCATCAATGAAAAGTCAATGGCAGAAAGGGAAACTTATTGGACGGGGCACATTTGGAAGTGTG
TATGTTGCAACAAATCGAGAAACTGGAGCTTTATGTGCAATGAAGGAAGTTGATATCATTCCAGATGACCCTAAATCTAGTGA
GTGTATCAAACAGTTGGAGCAGGAAATTAAAGTTCTCCATCATTTAAAGCATCCAAATATTGTGCAGTACTATGGCAGTGAAA
TTGTTGATGATCATTTTTACATATATTTGGAATATGTTCATCCTGGATCCATAAATAAATATGTTGATCATTTTGGAGCTATGACA
GAAAATGTAGTTCGAAATTTCACCCGCCATATTCTCTCTGGGTTGGCTTACTTGCACAGCACAAAGACAATCCACAGGGATAT
CAAAGGGGCAAATTTACTCGTTGACTCATTTGGTGTTGTTAAGCTTGCAGACTTTGGGTTGGCAAAATTTCTTACAGGACAA
GCATGTGATCTTTCACTGAAGGGCAGTCCACACTGGATGGCTCCAGAGGTCATGCAAGCTGTGCTGCGGAAAGATGCCAAT
CCTGATCTTGCTTTTGCGGTGGACATATGGAGCTTGGGTTGTACCATCATTGAGATGCTGAATGGAAGACCTCCTTGGAGTGA
GTTTGCTGCGCCTGCAGCAATGTTCAAAGTTTTGCATGAGAGCCCACCCTTACCAGAAACATTGTCTTCAGAGGGAAAGGAT
TTCCTCCAGCATTGCTTTCGAAGAAATCCTGCAGAACGGCCATCAGCTGCTATGCTGCTTGATCATTCTTTTGTAAGAAGTTC

ACAAGATCAAAATGTTTCAGGCTTCAGCCAGGCATTTTCAGGAATGCAGTTAGTGGACAAACCACGCAGTCCAGGAGATGC
CATGAAACATAAAATTCATTCCATGCCACTCTCTTCAGGCACACAAACCATGAACAGAAATGTACTAAGGTGA

>VviMAPKKK25    VIT_04s0044g01290

ATGCCTGCTTGGTGGGGGAGAAAGTCGAGCAAGTGCAAAGAAGAAGTGCAGCAGCAGAACCCAGAGAGCACTCTCTATAA
TATATCGAAAATCTCGATCAGAAACGACAAGAAGAATGGCAAGGACAAGCCCAAGAGCTTCGATGAGGGGCTGTTTTCGCG
AAATTCGCCCAGGAGCAGCAAGGACTATGGGGCGTTGACGGTCAGCGGTGGTGGTGGGTCGTCGGGGTTTTCCGGGTTCGA
TTCGGATTGTGGAGACAAGATCAGGGGACACCCACTGCCTCTGCCGTCGGCGGGAATTGAACATGGGGTGGGATCTGGGTC
GGGGTCGGTTTCGAGTGTTAGCTCTTCTGGGTCGTCCGATGATCATCCCAGCCCTCATGATCACGCTCCCTTTGGCGTATATA
GAGGACAGGGGGAAACCAAGTCCAACACAAGATCAAGAAGTCCAGGTCCAGGGTCACGATCTGCTACATCACCGCTTCATC
CTCGGTTTAGTACCTCGAATATAGATTCTCTGACGGGAAAGCAGGAAGAAGGGAGGAGCTGTCATCGGTTGCCCCTTCCGCC
GGGTTCTCCTACCAGCCCTTCTACCTTGTCCAGCACTAGGACTTGCGTAGTGACTGAAAGCACAACTTGTAATATGTCAAAG
TGGAAGAAAGGGAGGCTTCTAGGAAGGGGGACTTTTGGGCATGTTTACGTTGGATTTAACAGTGAAAACGGGCAAATGTGT
GCGATAAAAGAAGTCAAGGTTGTTTCAGATGATCATACATCAAAAGAATGCCTTAAGCAACTGAACCAGGAGATAAATTTAC
TCAGTCAACTTTCACATCCAAATATCGTTCAATACTATGGTAGTGAAATGGGTGAAGAAACACTATCAGTTTATTTGGAGTAT
GTCTCTGGTGGCTCTATCCACAAGTTACTTCAAGAATATGGTCCCTTTAAGGAACCTGTCATTCAAAATTATGCTAGGCAGATT
ATTTCTGGGCTTGCCTACTTACACGGGAGAAGTACTGTGCACAGGGATATCAAAGGGGCAAACATATTAGTGGGACCTAATG
GGGAGATCAAGTTGGCTGATTTTGGCATGGCAAAACATATAAATTCTTCTTCTTCAATGCTCTCTTTCAAGGGAAGTCCCTAT
TGGATGGCGCCTGAGGTTGTAATGAATACAAATGGCTACAGCCTTGCGGTGGATATTTGGAGCTTAGGATGTACAATTCTTGA
AATGGCAACATCAAAACCACCTTGGAGTCAATATGAAGGGGTGGCTGCAATTTTTAAAATTGGAAACAGCAGGGATGTCCCT
GAAATTCCCGATCACCTTTCTAATGATGCAAAAAGTTTTGTAAGGCTATGCTTGCAACGGGATCCATCGGCACGGCCAACAG
CCTTACAACTACTAGATCACTCTTTTGTTCGAGACCAAGCCACAACAAGAATTGCTAATATTGCTATAACAAAGGATGCCTTT
CCTTCCACCTTTGATGGAAGCCGCACACCGACTGCATTAGAGCTTCATTCCAACAGAACGAGTCTTACTTTATTTGATGGAGA
TTATGTGACAAAACCAGTGGGCACGGTTTCGAGAGCTGCAAAGAACTCAAGGGACAGTGTGAGAACGATCACATCTTTGCC
TGTATCTCCATGTTCAAGCCCACTACGGAACTATGGACCAGCACACAAGAGCTGCTTTCTTTCTCCTCCTCATCCATCTTATCC
TATAGTGGGACAAAGCAGTTACAACTCAAACGATTACTCACTATATCCAACAAGAGCAATCACAAAATATACACATGACCCTT
GGTCTGACAACCCCCCATTCAGATCCCTAACACCTAATGGATCCCCAAGAACGAGACCCATTTGA

>VviMAPKKK26    VIT_16s0050g00770

ATGCCTTCATGGTGGGGAAAATCATCATCCAAAGAAGCAAAGAAGAAAACAAACAAGGAAAGTTTCATCGACACATTACAT
CGAAAATTTAAGATTCCATCTGAAGGTAAGGTGAGCAATAGATCAGGAGGGTCTCATAGACGGTGCAGTGATACAATATCAG
AGAAGGGATCTCAATCCCGAGCAGAATCAAGATCACCATCACCTTCCAAACTAGTGTCAAGATGTCAAAGTTTTGTTGAAAG
GCCTAATGCCCAACCACTTCCACTTCCTGGTCGGCACCCTGCAAGTGTGGGTCGTACTGATTCTGGAATCAGTATATCAACAA
AACAAAGACTGGAAAAAGGCTCCAAGTCATCCTTTCTTCCTCTCCCAAGACCCAGATGCATTGGGGGCAGACCCGATCCTA
CAGATTTAGATGGTGATTTTGTTGCTTCAGTTTATAGCGAAGGTTCCACTGATAGTGAAGATGCAGCTGACTCACATCATCGT
AGTCCCCAGGCAACTGACTATGATAATGGGACTAGAACTGCTGCAAGCATATTTTCTAGTGTAATGCTCAAGGATCAGTCACC
TGTTGCTCATGTAAACGCAAGGGAGGCACAAAAACCAGCTAATCTTTTGTTTAGTAATCATATTTCCCCTACATCACCTAAAC
GGAGGCCTTTAAGCAGCCATGTACCAAATTTACAGGTCCCTTATCATGGTGCTTTCGGCAGTGCTCCAGACAGCTCAATGTC
AAGTCCTTCAAGAAGTCCATTGAGAGCATTTGGCACTGACCAAGGTTTGAACTCTGCTTTCTGGGCTGGGAAACCTTATTCA
GATGTTACTTTACTTGGATCTGGCCAATGCTCCAGTCCAGGTTCAGGTCAGAATTCTGGGCACAATTCAATGGGAGGAGATAT
GTCAGGACAGTTGTTTTGGCAACCCAGCAGGGGCAGCCCGGAGTATTCTCCTATACCTAGTCCCAGAATGACAAGCCCTGGT
CCCAGCTCCAGAATTCATAGTGGTGCAGTCACACCTCTTCATCCCAGAGCTGGAGGAGCAGCCTCTGAATCCCAGACAAGC
TGGCCAGATGAGGGGAAACAACAAAGCCACCGGTTGCCCCTTCCTCCTGTAGCAGTTTCTTCTTCTTCACCTTTCTCTCATTC
AAATTCACCAGCAGCATCTCCCTCTGTCCCACGCAGTCCAGGAAGAGCAGAGGCTCCAACAAGCCCAGGCTCTCGCTGGAA
AAAGGGAAAGTTGCTGGGAAGAGGCACATTTGGACATGTTTATGTTGGCTTTAACAGTGAAAGTGGTGAAATGTGTGCAAT
GAAGGAGGTGACACTATTTTCAGATGATGCAAAGTCAAAGGAAAGTGCAAAACAGTTGGGGCAAGAAATTGTTCTGCTTAG

CCGCTTATGCCATCCAAACATTGTGCAGTATTATGGATCTGAAACGGTTGGTGACAAACTTTATATATACTTGGAGTATGTATC
TGGTGGCTCCATCTATAAACTTCTTCAAGAATATGGCCAACTTGGTGAACTAGCAATTCGTAGCTATACCCAACAAATCTTGT
CAGGGCTCGCCTATTTGCATGCTAAAAATACTGTCCATAGGGATATTAAAGGGGCAAATATACTTGTAGACCCAAGTGGTCGA
GTCAAGTTAGCAGATTTTGGAATGGCAAAGCATATCACTGGGCAGTCATGTCCTTTATCATTCAAGGGAAGCCCATACTGGAT
GGCACCTGAGGTTATAAGGAATTCAAATGGTTGCAACCTTGCTGTGGATATTTGGAGTCTAGGGTGCACAGTTTTGGAAATG
GCCACAACAAAACCACCCTGGAGTCAGTTTGAAGGGGTTGCTGCAATGTTCAAGATTGGGAATAGTAAGGACCTCCCAGCA
ATTCCTGATCACCTTTCAGATGAAGGTAAGGATTTTGTAAGGCAGTGCTTGCAACGAAATCCACTACATCGTCCAACAGCTG
CTCAGCTCTTGGAGCATCCTTTCGTGAAAAATGCTGCACCTCTGGAAAGACCTATTTTGTCCCCTGAAACTTCAGATCCACCT
CCTGGAGTTACCAATGGAGTGAAATCTCTGGGAATCGGACATGCTAAAAATCTTTCATCCTTGGATTCAGAAAGACTTGCAG
TTCATTCGTTTAGAGTTTTAAAAACTGGTTCCCATTCAAGTGATCCTCATATTGCGAGGAATATATCATGTCCTGTCTCTCCTAT
TGGGAGCCCTCTTTTGCATTCAAGGTCACCTCAACACCTGAATGGAAGAATGTCTCCTTCTCCTATATCCAGTCCTCGTACCA
CTTCTGGCCCATCCACGCCTTTGACAGGTGGCAGTGGTGCCATTCCATTTCCTCATCTAAAACCATCAGTTTACCTGCAAGAG
GGATTTGGAAACGTTTCTAAGCCCCTAAACAATCCCTATTCCAACGGCCCCTCCTATCATGATCCAAATGCCGACATCTTTCG
AGGGATGCAGCTAGGGTCTCACATATTCCCAGAAAGTGATGCTCTTGGAAAGCAGTTTGGGAGGACTGCTCATGTAGAATTG
TATGATGGGCAGTCAGTCTTAGCTGATCGCGTCTCTCGGCAGCTCTTAAGGGATCAAGTGAAGATGAATCCATCTCTGGATCT
TAGTCCCTCCTCTATGTTGCCCAGCCGGAACACTGGAATCTAA

>VviMAPKKK27    VIT_12s0034g00750

ATGGAGAGGAATTGCACAAGTGATGGATTCTTTTTTGCTGTGAAGGAGGTTTCATTGCTTGATCAAGGGGGCAAGGGCAAA
CAAAGCATTTATCAACTTGAACAGGAGATTTCTCTTTTAAGTCAGTTGGAACATGAGAACATAGTTCGGTATTATGGCACAAA
TAAGGATGACTCTAAACTTTATATCTTCCTTGAGCTTGTGACGAAAGGTTCACTTTTAAGCCTTTATCAAAAGTATCATTTGCA
GGAAAGCCAAGCCTCGGTCTACACAAAGCAGATTTTAAATGGTTTGAAATATCTACATGAGCAAAATGTAGTTCACAGGGAT
ATCAAATGTGCAAATATATTGGTGGATGTACATGGATCTGTGAAAATTGCGGATTTTGGATTGGCAAAGGCAACCAAATTGAA
TGATGTAAAATCTTGTAGAGGAACACCATTCTGGATGGCCCCAGAGGTTGTTAATTGGAAAAATGAGGGCTATGGGCTTGCA
ACTGATATATGGAGCCTTGGATGCACTGTGTTGGAGATGTTAACTCGTCGACCCCCATATTCCCACTTGGAAGGCGCAACCAA
ATTGAATGATGTAAAATCTTTTAAAGGGACACTACGCTGGATGGCCCCAGAGGTATTTAATCAGAAAAATGAGGGCTATGGG
CTTGCAGTTAATATATGGAGCCTTGGATGCACTGTGTTAGAGATGTTGACTTGTCGACCCCCATATTCCAACATGGAAGACGG
GCAAGTGATATCAAAAATTTACAGGAGTGAACCTCCCGATGTTCCTGATTCCTTCTCATCAGATGCCCGAGATTTTATCCTCA
AATGCTTGCAAGTCAACCCTAGTGATCGGCCTACTGCTGGTGAGTTGCTGGACCATCCATTTGTGAAGAGGCCATCTGGCCC
TCAATCTCCTCGTACGAGTGGAATACAACCTTAA

>VviMAPKKK28    VIT_12s0142g00700

ATGGAGAGCAATTGCACAAGTGATAGAATCATTTTTGTTGTGAAGGAGGCTTCATTGCTTGATCAAGGGAGTCAGGGCAAAC
AAAGCATTTATCAACTTGAACAGGAGATTTCTCTTTTAAGTCAGTTTGAACATGAGAACATAGTTCGGTATTATGGCACAAAC
AAGGATGAGACTAAACTTTGTATCTTTCTTGAGCTTGCACCAGAAGGTTCACTGTTAAATCTCTATAGAAAGCATAAACTATT
GGAGCCCCAAGTATCTGAATACACAAGGCAGATTTTGAATGGTTTGAGCTATCTACATGGGAAACATGTAATTCACAGGGAT
GTCAAATGTGCTAACATATTGGTATTTGAAAATCACATTGTGAAACTTGCAGATTTTGGATTGTCAAAGGTTAGTTTCATTAGT
AGAGTTACAATCTCTTTTAAAGGGTCTCCATTCTGGACGGCCCCTGAGGTTGTTAATGCGGTGTATAGGAAGAACGATTGTTA
TGGGCTTGCAGCTGATATATGGAGCCTTGGGTGCACTGTATTAGAGATGTTAACTCAGCAACATCCATACCCTCAATATGAAT
GGATGCAAGCATTATTTAGGATTGGCCATGGTGAACTTCCTTTTGTTCCTGATTCCTTGTCAATAGATGCTCGAGATTTTATCC
TCAAATGCTTGCAAGTCAACCCTAGTGATTGGCCTACTGCTCGTCAGTTACTGGACCATCCATTTGTGAAGAGCCCATTGCAC
CCTTTCATTGGCCCTGCATCTCCTAGGGCTAATGGAATACGACCTTAA

>VviMAPKKK29    VIT_14s0066g00910

ATGGAAACTGGTCTCCTTCTCTCCATCATCTTCCTCGCAACAACATTCTCTTTCATCCATTCCACAAAAGCCAACCCCAGAGC
CGACATCATCGCTAGAATATGCAGCAATGACTACGCCCACAACTTCTCCAATTACCTCGACAGCTACTCCAAAATTATAACCC
AGTTGCGAGATGAACTGCCTAAGACAAAGTTCGCATTCAAGGAAGCTGGAGAGCCTCCTGACAAAATCTATGTGTTGGCTC

AGTGCATGGATGATCTCTCCTTCCAGGATTGTCAAGCCTGCTTCTCTCAGATCAGTTCTCTTTTCCCTGGTTGCTTTCCTGCAA
CTGGCGGTCGTGTTTATCTTGATGGCTGCTTCCTTAGAGGCGACAATTACAGCTTCTTTCAAGATACTCTCACGCCCATGGATT
ATTTGGTAACTGCTTTTGTTTTCTTAGTATTTTTTTTATTTTTTTGGCTGACATCAGCGGTGTCCAGAATTTTGATGATGTAGC
CAAGAGTGTGATTGATGAACTGGTCAGGATGACACCAAGTAGGGACGGGTATGCAGCATATGATGAGAGTGCCAATGGTATA
ACAGTTTACGGTATGGCTAGTTGTTGGAAGACTTTGGACCGTGATCGTTGTGCATCATGCCTTGCTAGTGCAGCCATTTCTGC
CTTTGCTTGCTTCCCATCTGCAGAAGGGCGTGTGCTCAATGCTGGCTGTTTTCTACGTTACTCTGATTACAAATTTTATCAGGG
CTTTGATTTTTCTTTTTATAGTTTTTCATGGGGATCTCTTGCAGATGCTACATTATCATTCATCTCACACGTCGTTGGCGTGGTC
AGTGTCTGCATTTTGGCGATTATTATCGGGTTTTTCATTGGTAAAGCTGCTTACCAAAAACGAAATCAGCAAAATGAAGCAAA
TGGTAACTCTATTATGAGGAAATTTGTTATGATTGAACACATTCCTCAAATCATCTTCTTGATTAATGAAGAAATTGAAGTGGA
CAGCTCGGTTGTAAAGAGAAGCTTGCAGTTCAAGTACACGACTCTTGAAAAAGCAACCGACTACTTCAATGAAGCCAACAA
ACTTGGGCAAGGTGGATTTGGTGAAGTGTTCAAGGGAACCTTAAGAGATGGCAGAGAAATTGCTATAAAGCGTTTATTCATA
ACGGGACAGAGTGGAGCTCAGGAGGTTTATAACGAAATAGACATTATAGGCAGTGCCTGCCACAAAAACTTGGTTCGTTTC
CTTGGTTGCTGCTTCACCAGACATGATAGCTTCCTTGTCTATGAATTCCTGCCCAACAGAAGCCTCGATCGAGTCTTATTTGA
CACAGAGAAGAAGAAAGAACTACCCTGGAAGATCAGGCTTGGAATAATCATGGGAACAGCAGAAGGCTTAGAGTACCTTC
ACAAAGACTGTCACGTTCGAATCATTCATAGAGACATCAAGGCCAGCAACGTCTTATTAGACTTCAGATACAGACCAAAGAT
TGCAGATTTTGGTCTGGCAAGGTTTTATTCCACTGACAGAGCCCTCACCGGCACTGCCATTGCAGGCACACTAGGGTACATG
GCCCCTGAATACCTAGCTCAGGGCCGATTAACCGACAAGGTGGATGTTTATAGCTATGGAGTTCTCATACTTGAGATTGTCAG
TGGTGTGCAGAATAACAAATTTCAGCTTGATGACTCCCTCAACACTCTAGCCACTGCTACATGGAAGCACTTTCAGTCAAAT
ACAATGACTGAGATTATAGACAAGGGCATGGAGATTGAAGATATGGAAGAAGTCACAAGAGTAATTCAGGTGGGTTTGTTGT
GTACTCAAGAATCACCCACTTTACGCCCTGCCATGACTGAAATAATTCAAATGCTTAAACAAAAGGATGTCTCATTGCCTATT
CCATCCAAGCCTCCATTCACTGAAGAAAATTTGACAATTTCTCCTGCATTGGGGTGTCCTAGGAGACCAGCTGTGGATGCATA
TGATCTATGTATATCTTGTGATCATTCTGATACTGAGCTCAGATAA

>VviMAPKKK30 VIT_04s0044g00850

ATGGGCTTGGTTGGTGTTGAGAGTGGTGAAGAGGGTATAGGGCATTCAGAGCCACCTGACCCTGATGTTCTAGAGATTGATC
CTACTTCACGGTACATTCGGTTCAAAGACATTCTCGGGAAAGGCGCATTCAAGACTGTTTACAAGGCATTCGATCAAGTTGA
TGGAATTGAAGTAGCATGGAACCAAGTTCGCATTGATGAGGTATTACAGTCACCAGATGAGCTGGAAAGGCTGTATTCTGAA
GTGCATCTCTTGAAATCATTGAAGCATAAGAACATAATAAAGTTTTATAATTCATGGATTGATGATGGGAACAAGACTGTAAAT
ATCATTACCGAGTTGTTCACCTCGGGTAGCCTTAGACAGTACCGTAAGAAGCATAAGAAGGTTGACATGAAGGCAGTCAAG
GGATGGGCAAGGCAAATTTTGATGGGTTTGAACTACCTTCACAATCACAATCCGCCAATTATACATAGAGACCTGAAATGTGA
TAACATTTTTATTAATGGGAACCAAGGGGAAGTTAAAATAGGAGATCTTGGCCTAGCGACTGTCATGCAACAGGCTAATGCA
AGAACTGTGATAGGAACTCCTGAGTTCATGGCGCCTGAGCTGTATGATGAGAATTACAATGAGTTAGCTGATATATATTCCTTT
GGGATGTGCATGCTGGAGATGGTAACACTTGAGTATCCTTACAGTGAATGCAGGAACTCAGCTCAGATATACAAGAAGGTTT
CAAATGGAATAAAACCTGCCGCCCTGTCTAAAATAAAAGATCTAGAAGTTAAAATGTTTATAGAGAAGTGTCTAGTACCTGCA
TCTCAAAGATTGTCAGCAAAGAAGCTTCTGAATGATCCCTTCTTCCAAGTTGATGGATTGACTAAGAATCATCCTTTGCAACT
TCCTGATATTGTTATTCCAAAGACTGGAGCCTTTGGTGACCGCTGTCTGCTCTCAGAAGGACCTACCAGTTTACAGAATAGAC
CACTTGCAATGGATCTTGATGCTGTTGATGATGATGAGCTACCCATTATCACCTCTATGGATAATTCTGTTGATGGAGGGCCCT
ATTCATTATGCATGGAGGTTCAAAGAGCAAAAGGAGGCAATTTTTTCCTGTTGAAAGGCGAGGGAAATGATGAGAACTCTAT
ATCACTAATATTAAGAATAGCTGATCAGAATGGTCGCTTGAGAAATATCCATTTCATGTTCTACCTTGATAGTGATACAGCCCT
CTCGGTTTCAAGTGAAATGGTGGAACAACTGGAACTAGCAGACCAGAATGTCACGTTCATTGCAGAGTTGATCGATTTGTTA
TTAATTATGCTAATACCAACCTGGAAACCTTGTGTCCCAATTGACCATCTGGTTGCTTTGAATAGAATGCAAACCTCTAATGGT
CATCATGAGGACTTACAATGTCCAGAACATGGAGAGTGCTTGGTGGGATCATTCGAAGGTGTTTGTGAAACAGATAATCTTTT
AAGCCCCCATGTCTACCCCAACTCAACTTCTTTTGAGGGATACATTGAGACAATGCAGGAAAACCCTAAACATTTGAGTCTT
GATGAGATCAAGACTCATGCTGATTTAGGGCTTCCAAGCTCAGCCACAGTAGAGGATCATGGTTCTGATATGTCATATGTTTC
TGCAACCTCAAATGAAGGGAGTGATAAGAAGTACTCTCATAATGCATATCTCTCTGCAGAGTCAGGATGCATGGATTACAATG

AGTATGGATCGAAAAGGGGAGTTAGACAATCCTTGTCGGCAGTGCAAACGAGTTCATGCAACCTTGACAAGGGAAAGGCTA
CAGACATCGGCAGCAATGGTGCAGTGACTTCTTCAGATTATCCAATTGATTCATCTTTGTCTGACCAGGTTGAAAGTGAGAA
CATGATATTGGAGCTGGAAATGATTGAGCTGCAATACCACGAGGCAGTTAAGGAAATAGCCAAGAGAAGACAAGAAGCTAT
CAGGGAGACAAAGAAAAGGTTGTCACAGAAAAGGATAGAGTCAAGTGGGTTTTTATTCTTAATTAAATTGGCTTGGGAAGC
GGATTCCCTCTCAAAGGCAAGGAGATAA

>VviMAPKKK31    VIT_02s0025g02360

ATGAATTCGGACAAGTTCGAGACTGATGATAGTGATTTTGAGGAGAAAGATCCCACAGGTCGATACGTTAGGTATAATGAATT
CTTGGGGAAAGGAGCATTCAAGACTGTATACAAGGCATTTGATGAAGTAGATGGAATAGAAGTCGCATGGGGTCAAGTGGA
AATTGAGGATTTGTTACAGTCCCCTCAACAGCTGGAAAGATTATATTCAGAGGTTCATCTGCTGAAGTCATTGAAACACGATA
ACATCATCAAGTTCTATAACTCTTGGGTTGATGATACGAACAAGACTATTAACTTGATTACAGAGTTATTCACTTCTGGGAGTT
TGAGACAATATCGGAAGAAGCATAAGAATGTTGACCTCAAGGCCATCAAGAACTGGGCGAAGCAGATCCTTCGAGGTTTAC
ACTATCTCCACAGTCACAATCCGCCCATTATTCATAGGGATTTAAAATGTGATAACATTTTTGTTAATGGTAATAATGGAGAAG
TTAAAATTGGAGATCTGGGTTTGGCAATTGTCATGCAGCAACCTACTGCACGAAGTGTTATTGGTACTCCAGAGTTCATGGCA
CCAGAGCTTTATGAAGAGGAATATAATGAACTCGTTGACATATATTCTTTTGGCATGTGCATATTAGAGTTGGTTACATGTGAA
TACCCATATAATGAATGCAAAAATCCTGCACAAATATATAAGAAGGTTTCTTCGGGTATAAAGCCTGCTTCCCTTGGTAAAGT
GAGTGACCCCCAAGTCAAGCAGTTCATAGAGAAGTGTTTGGTTCCAGCATCCTTGAGATTGTCTGCACAAGAGCTCTTAAA
GGACGCATTCTTCGCCACAGAAAATTCAAAGGAGCCTCTTTCAGTGAGCACTCATAAGAGTAGTGTGGATGATTCTCTGCAG
TCATCCAATTTTATGCCCAATCTGATGAACTTGCCTAAGCCTGAATTGCAGCCCATGGACATGGATCCTAACTACAAGAAGCT
TTCAGTGAGCACTCACATGAAAAGCATCAGTGGAACTCCTCATTTTCGTGCTCTACAATTTGAGAGGTTCAACAAAAATAAT
CTCTTCAAGTTAAGAGGGGAGAAAATTGATGATAATTCAATCTCAATGACCTTGCACATAGCTGATCCATGTGGTCGAGCAA
AGAATATCCATTTTGCATTTTATCTTGATTCTGATACTGCACTTTCAATAGCTGGTGAGATGGTTGAACAACTGGATTTGTATAA
TGAAGATGTGGCAGTCATAGCTGAGTTAATTGATGTTATGATATCCGAGCTTGTACCCACATGGAAACCTGCATTTGAGAGCA
TGTTATGTGGAGCAAATAGTTCATGTGAGGATTCTCTTGTACTTCACAATGGTGGAACCTCATTGAGGCATCCTAGTGATTCA
GGTTCAGCTAAGGGTACATCAGATGCAGTTACTGAACATCTTATCTCACTGTCGGCCAATGGGGAAGAACAGAGCACTGTGG
AGTCTGCTCTTTCAGGAATGTCAACTAAAGATGATGCCACAGTTGCTTCGGATGCAAATGACATCAAATCTTTGGAATGCCCT
GATGATGAATGCTATGAAGCTTCGGACAGATGTTGTTTCAATGGAGATCGTCAGGTACTTGATCATGAAAGGCATAAAGAAG
GAAGATACAACGGTAATATTGGTGAACCTGTTGCTATGAATGGATTCACTAAGGACTGGGAGATATCCTGCATTGAATCATGC
TCTGGGATGTCTAACAGTTTGAGCCTGTCAAGCATCTGTTCTCTATCCCTAGCAGACAAAGATCCAAGTGATGAACTAAAGC
TGGAGGTTGACACAATCGATACACAGTATCACCAGTGCTTTCAGGAACTCTTGAGGATGAGGGAGGAAGCAATAGAGAAAG
CCAAGAATAGGTGGATCACAAAAAGTAGATACCAACCTCTTCTTTGA

>VviMAPKKK32    VIT_15s0046g00100

ATGAATTTTAGGCATGGTTTGGTAATGCAAGGGGCTGAGGACGAGGACGAGGCCGCCTATGTCGAGAAAGATCCCACTGGT
CGATATGTTCGGTTCAAAGAAATATTGGGCAAGGGCGCATTCAAGACTGTCTATAAGGCATTTGACGAAGTTGATGGAATAG
AAATCGCTTGGAACCAAGTGAAGATTGACGATGTCTTGCGGTCACCAGAAGATTTGGAAAAACTATATTCTGAAGTTCATCT
CCTGAAATCATTAAAACATGAAAATATTATCAAATTCTATAATTCCTGGGTGGATGATAAGAAGAAGACTGTTAACATGATTAC
TGAGCTCTTCACGTCTGGGAGTCTAAGGCAATATCGAAAGAAGCACAAAAATGTTGATATGAAGGCTATAAAGAATTGGGCA
AGGCAGGTTCTTCGAGGTTTGGTGTATCTTCACAGTCACAACCCACCAATTATTCATAGAGACTTAAAATGTGACAATATATT
TGTCAATGGAAATCATGGGGAAGTTAAAATTGGAGACCTCGGACTGGCAATTGTTATGCAGCAGCCTACTGCTCGAAGTGTT
ATTGGGACTCCCGAGTTCATGGCTCCAGAGCTTTATGAAGAAGAATATAACGAACTTGTCGACATATATTCTTTTGGGATGTG
CATGTTGGAGATGGTTACTTTTGAGTATCCATATAATGAATGCAAAAATCCGGCTCAAATCTATAAGAAGGTTACCTCTGGCAT
CAAACCTGCTTCTCTTTGTAAGGTGACGGATCTCCAAATTAAGGAGTTCATTGTGAAATGTCTGGCTCCAGCATCTGAAAGA
TTACCTGCAAAGGAGCTTCTCAAAGATCCATTCTTTCAATCTGAGAATCCAAAGGAGCCGATCCGTGTCCCTTTGCAGTTAC
CTTCCCGAAGTCCCAAATCAATAATTCTATCCAAATCTGGGCCTTTCTCCATGGACATAGATCCTGACCATCCACAACTTTCTT
CAAGCACAAGTACAGAAAACAATGGAAGTCCAGATTTTCCAGTCTTGGAATTTCAGAGGATGTACAAAAGCAGTGAATTTA

GATTGAGGGCAAAGAAAATTAATGATAACTCAATATCATTAACTTTGCGTACTGTTGACTCGTATGGTCCAGTGAAGAATATT
CATTTTCCCTTTTCACTTGATACTGACACTGTACACTCTGTAGTGGGTGAGATGGTTGAACAACTGGAGTTAGCAGAACATG
AGGTGGCCTTCATAGCTGATTTTATTGATTACGTGATAATGAGACTTCTGCCCGGTTGGAAGCCTCCACGTGATGATCCCTTA
GGTGGAGCAAGGAGTCCAAATGCAGAGCCTCCAGTACTTGGAAATGGTAACAACCATGATTGTACTATTTCCCATGGTGATG
GTAATTCTTCTCCAAATTTGGCTAATGCAGAAGATCAGGATTCATTGGCATCTGCTGGTTTGGTGACCCTGACTGTAGATGCT
TCCAAAAAAAATGATAAAACGGTGGGATTTGGAGATTACAATATTGGTGGCAACTATAAAGGTTCAAATGGTGGGCATGCCT
CTGAACAGGAGTCTAGGGATCCATACCATGAAGACTACAAATTGCAAAGAAATAATAGCAGTATTGAGGAATTTACCCCAAT
GAACAAATTCCAGAAGAGCACGGTATTGTCTTTCGATGATTTAAGTGGACTTTCTAATGTTAGGAGCTTGACTTGTAGTTGCT
CATCTCTGTCTCTAGCAGACATAGATCAGGATCCTGGGCTAAAGCAGGAGCTTGATGCTATTGACTTACAGTATCAGCATTGG
TTTCAAGAGCTTTCTAGGATGAGAGTAGAGGCATTAGAGGCCACTAAGAAGAGATGGATGACAAAGAAGAAGCTGGCTGTT
CAATGA

>VviMAPKKK33    VIT_17s0000g09380

ATGCCCCAGGTTTTGTCCTCCGAGCAAGACCCTGATGATCCCGATACTGAATTCGTAGAGATTGATCCAACTGGGAGATATGG
TCGGTATAAGGAGGTTCTAGGAAAAGGGGCTTTCAAGAAAGTATATAGAGCATTTGATGAATTGGAAGGAATTGAAGTAGCT
TGGAATCAGGTTAAGGTGGCAGATCTATTACGGAACTCAGAAGAGTTCGAGCGTCTGTATTCAGAAGTTCATTTGCTCAAGA
CTCTGAAGCATAAGAACATAATTAAGTTTTATATTTCGTGGGTCGATACAAGGAATGAGAATATCAATTTTATTACAGAGATTT
TCACCTCAGGCACATTGAGGCAGTATCGAAAGAAACATAAGCATGTTGATTTGAGGGCTCTGAAGAAATGGTCTAGGCAGAT
TCTGGAGGGTCTTCTCTATCTTCACAGTCATGACCCTCCTGTTATTCATCGGGACCTGAAGTGTGACAACATTTTTGTTAATGG
AAACCAAGGAGAGGTGAAAATTGGGGACTTAGGACTGGCTGCCATTCTTCGCCAGGCTCGATCAGCTCATAGTGTCATTGGT
ACTCCTGAGTTCATGGCACCTGAGCTTTACGAGGAGGAATACAATGAGCTTGTAGACATTTATGCCTTTGGGATGTGCTTGCT
GGAGTTGGTGACCTTTGAGTACCCATATGTTGAATGTGCCAATGCTGCTCAAATATACAAGAAAGTGACATCAGGAATCAAA
CCAGCATCATTAGCAAAAGTGAAAGACCCTAGAGTTAGAGCATTTATAGATAAATGTATTGCAAATGTCTCTGATCGGTTGTC
TGCCAAGGAACTCTTGAGGGATCCTTTTCTCCAATCAGATGAGGAAAATGGAAGTGTAGGCCGTTCTTTACAACCCCATCCT
CATCATTCAGGTAGTCATGACCACTTCAATACTGGTACAAGTTCTAAGGTCTCTCTGCCTGAGTCAAGTCGAGACTTCAAGG
TGCAAGGTCAAAGGAGAGATGTCAACACAATATTTCTAAAATTACGAATTGCAGATTCTAAAGGTCATATTCGCAACATCCAC
TTCCCATTTGATATTGGGGCTGATACAGCAATTTCTGTTGCTGGTGAAATGGTTGAAGAGTTGGACTTGACAGATCAAGATGT
TTCAACCATTGCTGCAATGATTGACTCAGAAATTCGGTCCATCATTTCAGATTGGCCCCCATCAAGAGAAGTTTTTGGGGATA
ATTTGAGCACAGAGGTTGCAATTTCTGATATCTGTCCCCCTGAAAGTGAGGGTGATGCTTTGCCCTTGATGAATGAGTCTGCT
ACTTCTTCTTGTGGCCTTGTCCTGGAACGGTTGCCTTCAGGTCGGAGGTACTGGTCCGACTCACCCAAGGCAGTTGGTGGA
AACTCTCCTATTAGACCTGCCTTTTCAAACTTGTCTTCTCAGGTTGATTCGGTAACTACTGAAGGTAGGTTTAGTGAACTTAA
TGAGCAATCTCTTGCTAGTCCTAGAGATGGAGATAAGTTAAATACTGCTGCTTCACTTGATAAGCGGGAGGATGAACGTGTAT
GTGGTGATGATGACGTTGAAGAGAAAGAAGCTAGTATATCTGCTGAAACCCAGTTTAGTGATCAAAACGATGTGGCTGTAGA
ATTACTTGGTGGCTATAGAGCACCTTCATGGGGAGGGAACTGTAAAATATTGAGGGAAACTGAATTAGGAGACGCCAAAGTT
ATTGTGGAGAAACTTAAGCATTTGTTTGTAAAACAACAAAAGGAGTTAGACGAACTAAAGAGGAAGCATGAATTGGCCATA
TTAGATCTTGTGAAGGAACTTCCTCCAGACATCCGTAACAAAGTTTCTAGCCTTTGTAACCTGAAGATCTCTGGCTCCTTTTC
AAAAATGGAACCAATGTCATCAGAAGTTTCCAACTTGGCCGTGTATAACAATCTAGATACTTATTCCATTGGACCAACTAATG
TCAAACTTAGTCTCCCAAAAAAAAGGTTAA

>VviMAPKKK34    VIT_19s0090g01690

ATGCCAGCGGTGAGCCCCGATCAATCTGATCGGGATTCAGAGCCGTTTGTGGAGGTAGATCCGACTAGAAGATATGGTCGGT
ACAATGAACTGCTTGGCTGTGGTGCAGTGAAGAGGGTGTACCGTGCTTTTGATCAGGAGGAGGGCATAGAGGTGGCTTGGA
ACCAGGTGAAATTAAGGGCTTTTAGTGATGACAAGCCAATGATTGATCGGCTTTTCTCTGAAGTTCGATTGCTCAAGACTCTT
AAGGACAAGAACATCATTGCTCTGTACAATGTGTGGCGAAATGAGGATCATAACACCTTGAATTTCATAACTGAAGTGTGTA
CATCTGGTAATTTGAGGGAGTATCGGAAGAAGCATAGACATGTTTCCATGAAGGCACTGAAGAAATGGTCAAAGCAGATTTT
GAAGGGTTTAGACTATTTGCATAGGCATGAACCCTGTATTATTCATAGGGATCTCAACTGCAGTAATGTCTTCATCAATGGCAA

TGTGGGTAAGGTGAAGATCGGTGATTTTGGATTGGCAGCAACAGTGGGGAAGAGCCATGTAGCACATTCAGTGCTTGGAAC
ACCAGAATTCATGGCTCCAGAGCTATATGAAGAGGACTACACAGAACTAGTAGACATATACTCATTTGGTATGTGCTTTCTAG
AGATGGTCACATTAGAAATCCCATACAGCGAATGTGACAACATCGCCAAAATATACAAAAAGGTGATATCTGGGGCTAGACC
TCGGGCCATGGACAAGGTCAGGGACCCTGAGGTGAAGGCATTCATCGAAAAGTGCCTGGCAAAGCCAAGGGCAAGACCCT
CAGCTTCAGAGCTCCTCAATGACCCATTTTTCCATGGAATTGATGATGACGAAATTGACAACAGTGATAGTTAG

>VviMAPKKK35    VIT_06s0004g07920

ATGTATAGAACAAAGCTTGGAGAGCTCACCAACGAAGGAAAAGATGAGTCTGGATATGCTGAAACCGATCCAACTGGTCGC
TATGGTAGATTGGACGAAGTCTTGGGCAAAGGAGCAATGAAGACAGTTTACAAGGCAATAGATGAGGTCCTTGGAATGGAA
GTGGCATGGAACCAAGTGAAACTCAATGAAGTTCTCCGATCACCCGATGAGTTACAGCGGCTTTACTCTGAAGTTCATCTCC
TCAGCGCCCTCAATCACGATTCCATCATCCAATTCTACACTTCTTGGATTGATGTGGAACGCAAAACCTTCAACTTCATCACT
GAATTCTTCACCTCAGGCACCCTCAGAGAATACAGGAAGAAGTACAAAAGGGTGGACATCCGAGCTATTAAATGCTGGGCA
CGGCAAATCCTCAGGGGGCTTGTTTATCTGCATGGCCATGATCCTCCAGTAATTCACAGAGACCTCAAGTGTGATAACATCTT
TGTCAATGGGCATCTTGGAGAAGTTAAGATTGGCGATCTAGGCTTGGCAGCCATTCTCCGGGGCTCCCAATCAGCTCACAGT
GTTATAGGAACACCGGAGTTCATGGCACCAGAGTTATATGAGGAAAACTACAATGAACTGGTTGATGTCTACTCATTTGGCAT
GTGTGTATTAGAAATGCTTACATCTGAGTACCCCTATAGTGAATGCTCCAACCCTGCACAAATTTACAAGAAAGTGACATCGG
GAAAGCTTCCTGGAGCATTCTACCGGATTCAAGACTTGGAAGCACAGAGGTTCATTGGGAAATGCTTAGTAACTGCTTCCAA
GAGACTACCTGCAAAAGAACTATTGCTTGATCCTTTTCTGGCCTCTGATGAGGCCAAACGGCTTCCTAAACCAAAGCTTGGA
AGTCAGAAGCCATTTCTGAATGACATAAGAATAGAGAAACTGCGCTTGAGCGATGATAGAGTAAGGACCAACATGACAATC
ACAGGGACACTGAATCCTGATGATGATACCATTTTCCTTAAAGTGCAGACTGCTGATAAAGATGGGTCTGCAAGGAACATATA
TTTTCCCTTTGACATTGTGACTGACACTCCAATTGATGTGGCAATGGAGATGGTGAAGGAGTTGGAGATCACAGATTGGGAG
CCATTTGAAATTGCAGATATGATTGATGGGGAGATATCTGCTCTAGTACCACAATGGAAGAAATGGGACATGCCACAGCAACA
TCACTATGCATTTGATTATCAAGAAGAAGATGAGGGACATAACCATCCATTCCGCTCTTTCTCCTCCTGTTCATCATCCCAAGC
TTCGTTTCCGTGTTTGAGCACTTCTCACAGGTTGGACACAATGGCTCAAGGTGGTGATTGGCTCAAAGATGATTTGTTTGAC
GATACCAGCTCTGAAAGTTCTGCACACTCTGGAAAATATTCCAACTTGAATTACTTTTCTGGCAATGAACACTGTTCCGAAAC
AAGTACTTTAAGAAGAGAACAGCATCCTGGGGCGAAAACTCAGAAGTCTACAAGGTTTTGCCCTGAAGAGAACTCTAGCAC
GCGGAAGGCCCTGCCTGGAAAAAGCTACAAGCAAGGCAAGGTTTTGCAGGAGTCACAGAGAGCTCCTGGTTCTAAGGATA
AGTTCGCAATGGAGACCATTAGATTAACAAGAAACCGCTCATTAGTAGATGTGCGAAGCCAGCTACTGCATAGAACATTGGT
GGAGGAGGTGCACAAAAGACGGTTGTCCAAGACGGTTGGAGCTGTGGAAAACATTGGGTTTCAGGCACCCTGCAATGTCT
CCGGGAAGGTATCACAGAAACCAACTGGTGCTCATTCCACAAGAACTACAAGAGATGGAAAAGGGCAAGGATCTCAAAGA
AGAAGAGCTTAA

>VviMAPKKK36    VIT_08s0058g01130

ATGAATAATACTCGGTTGGGAGAATGTCGAGGTGGAGCGAGGCAACAATTTGGATACGTTGAGACCGATCCATCTGGTCGGT
ATGGGCGTTTCAGGGAAATTCTTGGCAAAGGAGCAATGAAGACAGTGTACAAGGCATTCGACGAGTTCCTTGGCATGGAGG
TGGCTTGGAATCAGGTGAAGCTGAACGATGTCTTCAATTCACCCGACGATTTGCAGCGACTCTACTCTGAGGTTCACCTCCT
CAAGAACCTCGATCATGACTCCATAATGCGATTCCACACATCTTGGATCGACCTTGATGGAGGAACCTTCAACTTCATCTCTG
AAATGTTTACCTCTGGCACCCTCAGAGAGTATAGACAAAAGTACAAGCGGGTTGACATTGGAGCTGTTAAGAACTGGGCAC
GCCAGATTCTACATGGGCTTGCTTATCTGCACGGCCATGACCCTCCGGTGATCCACAGAGACCTCAAGTGCGATAACATCTTT
GTGAATGGTCATCTTGGACAGGTCAAGATTGGTGATCTAGGCTTAGCAGCTATTCTTCGGGGTTCCCAACACGCCCACAGCG
TCATAGGTACACCGGAGTTCATGGCACCGGAATTATATGAAGAAGAGTACGACGAGCTTGTAGATGTATACTCCTTTGGCATG
TGTGTGTTAGAGATGCTTACATCAGAGTACCCATATTGCGAATGCTCCAATCCTGCTCAGATATACAAGAAAGTGACTTCGGG
AAAGCTACCAGAAGCATTCTACAGGATTGAAGATGTTGAGGCAAGGGAATTTGTGGGAAGATGTTTAGAGCATGTTGCAAA
GAGGTTGCCTGCCAAAGAGCTCTTGATGGACCCTTTTCTAGCAGTGGACCATGGAGAGCAGATGTTGCCCATGCTAAAGATA
TCGAGTCAGAAGCCAAGTCCTAATGGAACAGTGGAGAAGATACCATCTTTCCAGACTAATCCTAGGAAAAGGAGTACAGAT
ATGACGATCACAGGCACCATAAATCCGGATGACTATACAATTTTTCTTAAAGTGGCAATCTCTGATAAAGATGGTCTTTCAAG

GAACATTTACTTTCCCTTTGACATTGGAAGTGACACTCCAATAGACGTTGCAGCTGAGATGGTGAGGGAATTGGAAATCACA

GATTGGGAGCCATTTGAGATAGCCAAGATGATTGAGGAGGAGATTTTTGCTTTGGTTCCAAGTTGGAAGCAATGTACCTCAC

CTGAAAATCATCAGCATAGCTTCGAATATGAAGAAGAAGAAGAAGAAGATGATGACGACGAAACTTACCATCCTTTCTA

CTGTTATGCCTCTGAGTCCTCTCGGGTTGCTCTCCAAGATCTCAGCATTTCATGTGAGATCCAATCTCAACATAGGAACCATAT

GATTTCCGGCGAGGACTGGTTTCAAGAAGGTTTGCTCATCAATGACGATGCAAGTTCTCAAAGCTCCTTAAACTCCGACAAG

TACTCCACTTTAGTTTACTGCTCAGTCACTGAAAATGATATTGATCACCTAGCTCCCAAAAGAGTAGAGCCCATCTTCACTGC

AAGTACCCACAAGTCCACTAGGTTCTGCCCTGAAGAAGGCACGAGTAGCTGGAACCAATGCAATGGTTCGCGGAGACCATA

TGACTCAAATTGTCATCGGAAATTAAGCAGGATAAAATCATTTGTGGATGTGCGCAGCCAGCTGTTGCGGCGGTCTCTAATG

GAGATGATAAACAAACGGCGATTGTTCAAAACCATCGGAGCTGTTGAGAACATCGGGTATCAAGAACCAGGCAAGTTTCCC

AAAGAGATGTCAATGACTGGTGGTTTGTCTGGGAACTCTCCCAGAAACAGTGAAAAGCAGAGATTTAAATGCTGA

>VviMAPKKK37 VIT_05s0020g03380

ATGAATTGCGTTACAGAGCTTGAACCAGATTATTCTGAGTTTGTTGAAGTTGATCCTACCGGTAGATATGGAAGGTACAATGA

AATTCTTGGCAAAGGAGCTTCCAAGACAGTATACAGAGCCTTCGATGAGTATGAAGGGATTGAAGTAGCCTGGAACCAGGT

GAAGCTTAATGATTTCCTGCAGAGTCCTGAAGAGCTGGAGAGGCTGTACTGTGAAATTCATCTGCTCAAGACCTTGAAACAC

AACAACATCATGAAATTCTACACTTCCTGGGTTGATCCTGCAAACAGGAACATTAATTTTGTGACAGAGATGTTCACCTCCG

GTACTCTTAGACAGTATAGGCTAAAACACAGGAGAGTTAACATCAGAGCTGTGAAGCATTGGTGCAGACAGATCTTAAGGG

GGCTGCTCTACCTCCACAACCATAAACCCCCTGTTATCCACAGAGATCTCAAGTGTGACAACATTTTTGTCAATGGCAACCA

AGGGGAAGTCAAGATTGGTGATCTGGGGCTGGCTGCAATTCTTCGGAAATCCCATGCAGCTCACTGTGTTGGAACACCAGA

GTTCATGGCCCCAGAAGTGTATGAAGAGGAGTACAATGAATTAGTTGACATCTATTCTTTTGGAATGTGCATCTTGGAAATGG

TTACCTTTGAATATCCATACAGTGAATGCACCCACCCCGCTCAGATCTACAAGAAAGTTATCTCCGGTAAAAAACCAGATGCC

CTTTACAAAGTGAAGGATCCTGAGGTGCGGCAATTTGTCGAAAAATGCTTAGCCACGGTGACTCTTAGGCTTTCTGCCAGGG

AGCTTCTGAAGGACCCTTTTCTCCAAAGTGATGACTATGGGTCTGATTTGAGGCCAATAGAGTACCAGAGGGATTTGGGTGA

AGTTGGCCCTCTCCCGAGACTACCTCACTATGGCATTCATCATAGTTACAGCTCTTTGAGGAACGAGTATTCGGGCTATCCCG

ACTTTGAACCAGAGAATGGCTTAGACTGCCACCCAGTTGAGTTTGAAAGAAATGAAATTGATCTCTTCACTTATCAAGAGGA

TGAGCATTTGGAAAATGTTGATATCTCCATCGAAGGGAGAAAGAGAGATGATCATGGAATCTTTCTAAGGCTCAGAATTTCA

GATAAAGAAGGTCGGGTTCGGAACATTTACTTCCCTTTTGATATGGAGACTGACACGGCACTGAGTGTTGCAATGGAAATGG

TTTCTGAGCTGGATATTACCGACCAAGATGTGACTAAGATAGCAGATATGATAGATGATGAGATTGCTAGCCTGGTACCGGGT

TGGAAGATGGGGCTTGGCATAGAGGAGAGCCAGAATTATGGCCATGATGCAAGTTTCTGTCACCATTGTGCTTCAAACGGTT

CCCCTTTAGATTACGTGTCACCAAATAACCCGGGCACCAAAAATTTGCAAGTTCTTCAATGTTCAAGACAGGGATGTGCTGC

TGTGCATGGCCGCTTTGAAGAGATCACTTACCGAGTTGAAGGGTCTGAACAATGTGTTACAGAAGGTGCCCCAGTTGTTTCT

AGCCAATCTGATGGAATGCAGTATGCTGATATTTGGGCTCAACGAGAGGGACCTGAATTAAGTTCAGAGGGGTCCAGGGAGA

TCCAATCTGATGAAGAGCATGAATCATTAGACAAATCAATCTATGGAAAGGATGAGAGAGTCATAAATATCGATAACCAAAGT

GAATCCAATGCAAAGAACTCTTTTGCTCCCCTGGACGATTATGAGAATGAGATCAGGCAGGAATTGAGATGGCTGAAAGCA

AAGTACCAAATGCAGCTGAGGGAGCTTAGAGATCAACAGTTAGGAGCCAAACCAAAATGGCTGAGTCTAACCCCAAATTCA

GATAGCATGGAACACAGTAGAGATAACAAGGTTTCACCTTCTTCACTCTCAACCCCACTGGAGGGAGAGGACAATGATCCTC

CCTTGAAATCTTTTCCCTGTGGGAAGCATTTTAACTCATTTTTTCCTGTTGATACTGAAAGGGGATGTGCCAATTTAGCATATC

GAAGGCCCCATAATCGTGAGCCAGTGAGCGAATCCTGCAGCCCTGAGGACATGGTAACTGCCAAGAGTTTCTTTACAGGGA

CTTTGCTACCACAATCTCTTCACAGGGCAACTTCTCTTCCAGTTGATGCTGTAGACTTTTGA

>VviMAPKKK38 VIT_05s0094g01080

ATGGAGATTGCGGCTCAGCTGAAGCGAGGCATCTCGCGCCAGTTCTCCACTGGATCGCTGCTGAGGACCCTGAGCCGGCAG

TTCTCGCGGCAGTCGTCGCTGGATCCGCGGAGGACCAATCTGAGGTTCAGCCTCGGGAGGCAATCGTCGCTGGATCCGATTC

GGCGGAGCCCGGTGAATGAGGAGCTATCGGTGCCGGAGAATCTGGATTCCACCATGCAGATGCTGTTTATGGCGTGTAGAGG

GGATGTGAAGGGAGTGGAGGATTTGTTGAACGAAGGCACTGATGTGAACAGCATCGATTTGGATGGCCGCACTGCTCTGCA

TATCGCGGCCTGTGAAGGTCAGATTGAGGTAGTTAAGCTCCTGCTCAGTCGGAAGGCCAACATCGATGCTCGTGACCGTTGG

GGGAGTACGGCAGCTGCTGATGCTAAATACTATGGAAATGTAGAAATTTATAATATTTTGAAGGCTCGGGGAGCCAAAACTCC
GAAAATCAGGAAGACGCCAATGGCTGTTGCAAATCCTCGAGAAGTTCCAGAGTATGAGCTTAATCCATTAGAGCTTCAGGTC
CGCAAAAGTGATGGTATCACAAAGGGATCATACCAAGTAGCTAAATGGAATGGTACAAAGGTTTCTGTAAAGATACTTGATA
AGGACAGCTATTCAGACCCCGATAGCATAAATGCTTTCAAATATGAGCTAACCTTATTAGAAAAGGTCCGGCATCCAAATGTG
GTTCAGTTTGTTGGAGCTGTTACCCAAAATATACCCATGATGATTGTTTCAGAGTATCATCCGAAAGGTGACCTGGGAAGCTA
TCTTCAAAAGAAAGGGCGTCTATCTCTATCTAAAGCTCTAAGATATGCTCTTGATATTGCCAGGGGCATGAATTATCTACATGA
ATGCAAACCAGACCCAGTTATCCACTGTGATTTAAAGCCAAAAAATATTTTGCTGGATAGTGGAGGTCAGTTGAAGGTTGCC
GGATTTGGTTTATTAAGGCTGTCAAAGATGTCACCTGACAAAGTAAAATTAGCGCAATCAGGGAGTCACATTGATGCTTCAA
ATGTATATATGGCACCTGAGGTTTATAGAGATGAACTATTTGACAGAAGTGTGGATTCATTCTCTTTCGGTCTCATACTTTATGA
GATGATTGAAGGAGTACAGCCCTTTCACCCAAAACCTCCAGAAGAAGCCATCAAAATGATCTGTTTAGAAGGAAAGAGACC
ACCTTTCAAGAGCAAATCAAGAAGTTATCCTCCAGATTTAAAAGAGTTGATCGAGGAATGTTGGAATCCGGAACCTGTTGTT
CGGCCAATCTTTTCTGAGGTCATAGTACGGTTGGATAAAATTGTTGGACATTGCTCTAGACAAGCATGGTGGAAAGACACTT
TCAAGCTTCCTTGGAAATAA
>VviMAPKKK39 VIT_15s0046g02850
ATGGCACCTGAGGTTTATCGCCGAGAATCATATGGGAAGAGTATTGATGTCTTCTCCTTTGCTGTAATAGTACATGAGATGTTC
CATGGGAAAACATCAAAGAGAGCAGAAAACCCAGAGTATGTTGCAGACAAGCAAGCATATGAAGATTCCCGGCCTCCTCTC
TCCTCATATGTATACCCTAGACCCATCAAGACGCTTCTCAGAAACTGCTGGCACAAGAATCCAGAAGTGCGGCCTACATTTG
AAGCCATAATTTTGGAGCTAGAGGAAATACAAGTGAGTATGCTAGATAAGAAAGCAGCTTGCCATGATTATCAATCACATGAA
TACCATGTAAATGGTTATGTATTCTTCATAATGGTTACACTATTATATCTCTACGAGCCTGCTTGGCGTCTCTTGAAACCGAACA
TAGCACAAATGAAGATGTATGACATCAAATTAGTAGTTATGCTAGCTAAGACAGAAACCTTGGGTGCCAGCTATAAACTTCAT
GTGCTAGGTATGAAAGTTAGGATGCAACCACAAACCATGGATTCAGAAGGACCATATCAGCTTCTCCACTGCTCTAGTAAGG
GTGACAAAGAAGGTGTAATACAAGAATTGGAGAAAGGAGTTGATGCCAATCTAGCTGACTATGATAAAAGAACAGCACTCC
ATTTAGCAGCCTGTGAAGGCTGCGAAGAAATTGTTGTTCTCCTTCTTGAGAAAGGAGCTGATGTGAACTCCATCGATCGCTG
GGGACGCACTCCACTTTCAGATGCTCGCAGCTTTGGTCATGAGAAGATATGCAAGATACTGGAGGCGCAGGGTTTTCATGTC
TTACAAACATACAAAACACACATATCTGAGGCCTCTTTTCACTATATGCAACGGACTCCATGCTACGAAATTGATCACACCGA
GGTGGACATGGATGAAGCAACTCTTATCGGAGAAGGAGCATATGGTGAAGTTTATTTAGTGAAGTGGCGTGGAACAGAAGT
TGCTGCAAAGACAATCCGTTCCTCCATTGCATCGGATCCAAGGGTGAAGAATACCTTTTTGAGGGAACTAGGTTTATGGCAG
AAACTGCGCCACCCTAATATAGTACAGTTCCTTGGTGTTCTAAAGCACTCTGAGCGCCTTATTTTCCTCACTGAGTATCTTCG
AAATGGAAGTTTGTATGATATTTTGAGAAAGAAAGGAAGACTTGATCCACCAGTAGCTGTTGCCTATGCTTTAGATATTGCAA
GAGGGATGAATTATCTTCACCAGCATAAACCACATGCTATAATTCACCGGGATTTAACTCCAAGAAATGTGTTACAAGATGAA
GCAGGACGCCTTAAGGTTACAGACTTCGGTTTAAGCAAAATTGCTCAGGAAAAAGATGCAGTTGGTTACAAAATGACTGGA
GGAACAGGTTCATATCGTTACATGGCACCTGAGGTTTATCGCCGAGAATCATATGGGAAGAGTATTGATGTCTTCTCCTTTGC
ACTAATAGTACATGAGATGTTCCAAGGGGGGCCATCAAATAGAGCAGAGAACGCAGAATATGTTGCAGATAAGCGAGCATAT
GAAGACTCACGGCCACCTCTCTCCTCATTTGTATACCCCGAACCTATCAAGACGTGA
>VviMAPKKK40 VIT_10s0003g02060
ATGGCTGTGGAATCGAAGACGGCGGTGAGGTTCACCTTGGGGAAGCAGTCGTCCCTGGCGCCGGAAAGGGCCCGGGATGA
AGCTCTGACCGAAGGTGAGCAGGGAGATGTTGAGGGGATTGATCCCAGGGTCAGGCTGATGTATCTGGCAAATGAGGGCGA
CTTGGAGGGGCTGCGCGAGCTTTTGGATTCGGGAATGGATGTCAATTTCAGGGATATTGATAATCGGACGGCTCTCCATGTGG
CCGCGTGTCAGGGCTTCAGCGATGTGGTTGAGTTTTTGTTGAAGAATGGGGCTGAAATTGACCTCGAAGATCGCTGGGGAA
GCACACCCCTTGCAGATGCAATACATTACAAAAACCATGATGTAATCAAACTTCTGGAGAAACATGGTGCACAACATCTGAT
GGCTCCCATGCATGTAAATAATGCCCGTGAAGTCCCAGAGTATGAAATTGATCCTAAGGAGCTTGATTTTACAAACAGTGTTG
ACATAACTAAGGGAACCTACCGCATTGCATCATGGCGTGGAATTCAAGTTGCTGTAAAAAGGCTTGGGGACGAAGTGATAAT
TGATGAGGATAAAGTGAAGGCATTTAGAGATGAGCTTGCATTGCTTCAGAAGATACGACATCCCAATGTAGTCCAGTTTCTG
GGTGCTGTAACTCAAAGTAGTCCAATGATGATTGTGACAGAATATTTACCCAAGGGAGATCTTCATGCATTCTTGAAAAGAA

AAGGAGCATTAAAAACGGCCACAGCTGTGAAATTTGCGCTTGATATTGCAAGGGGAATGAATTATTTGCATGAGCATAGACC
AGAAGCAATAATTCATCGTGATCTAGAGCCTTCAAATATATTGCGGGATGATTCGGGACATCTGAAAGTTGCAGACTTTGGAG
TTAGCAAGCTGCTGAAAGTTGCTAATACAGTTAAAGAAGATTACCCTCTAATCTGTCAAGAAACTTCTTGTCGATATTTGGCT
CCAGAGGTTTTTAAAAATGAAGCTTATGATACCAAGGTGGATGTTTTTTCATTTGCTCTAATTCTACAAGAGATGATTGAGGG
CTGTCCACCTTTCAGTGCAAAGCCAGAAAATGAGGTTCCTAAAGTATATGCTGCACAAGAGCGACCGCCTTTCAGAGCTCCA
TCCAAGCTTTATTCTCATGGACTTAAAGAGTTGATTGAGGAATGCTGGAATGAGAATCCTACGAAGAGACCAACATTTGGGC
AAATACTTACTAGGCTGGACCGCATTTATAACCATCTTGGTCAAAAGAGGCGTTGGAAGGTTAGGCCATTGAAATGTTTTCAG
AACCTGGAAGCAATGTGGAAGAAAGATCATTCTGATCTAAGCACCTCCAGCCGTTCATCTCGTCCAAACCGGTAG
>VviMAPKKK41    VIT_01s0182g00020
ATGAGTTGCAGAGACGACAATTCCGGTGGCCACTCCTCGACGTCTGCCGCCGGGAAAGACAAGGCCTCTGATAAGCAGAA
GGAGAAGGCCCGGGTGAGCAGGACTTCTCTCATACTCTGGCACGCTCACCAAAACGACGCCGCTGCCGTCCGCAAGCTTCT
CGAGGAGGATCAGTCTCTTGTTCACGCCAGGGATTATGATAGTAGGACTCCGCTTCATGTTGCTTCTCTCCATGGTTGGATCG
ATGTAGCCAAGTGTTTGATTGAGTTCGGCGCTGACGTCAACGCCCAAGATCGATGGAAGAACACGCCTCTTGCTGATGCAG
AAGGAGCTAAAAAACACAGCATGATAGAGCTGCTAAAATCATATGGCGGCTTGTCTTATGGACAAAATGGAAGCCATTTTGA
ACCAAAGCCTGTTCCACCTCCTCTACCAAACAAGTGTGACTGGGAGATTGACCCTTCCGAGTTGGACTTCTCAAACTCTAGT
ATTATTGGAAAGGGCTCTTTTGGTGAGATTTTAAAGGCCTGTTGGCGTGGAACACCAGTAGCTGTGAAACGCATTCTTCCAT
CTCTTTCAGACGATAGATTGGTGATTCAGGATTTCAGGCATGAAGTTAATTTGCTCGTGAAGCTTCGTCACCCTAATATAGTCC
AATTTCTTGGAGCTGTCACTGATAAGAAGCCGTTGATGTTAATTACAGAGTATTTGCGAGGGGGTGATCTTCACCAGTACCTC
AAAGAAAAGGGCTCACTTAGCCCATCAACAGCTATTACCTTTGCCATGGATATTGCCAGAGGCATGGCTTATCTTCACAACG
AGCCAAATGTCATAATTCACCGAGACCTAAAGCCAAGGAATGTTCTTTTGGTCAACACCGGCGCCGACCATTTAAAAGTTGG
AGACTTTGGACTGAGCAAGCTCATCAAGGTTCAGAATTCTCATGATGTGTACAAAATGACTGGTGAAACAGGAAGTTACCG
ATACATGGCTCCTGAAGTTTTCAAGCACCGGAAATATGACAAGAAAGTTGATGTTTTCTCTTTTGCAATGATACTGTATGAGA
TGCTTGAAGGAGACCCACCATTGTCTAATTATGAACCTTATGAAGCAGCTAAATATGTGGCAGAAGGACAGCGGCCTATGTT
CCGTGCGAAAGGATATATTACTGAACTGAAAGAGTTGACAGAGCAATGCTGGGCTGCTGACATGAACCACAGACCATCTTTC
TTGGAAATTCTTAAGAGGCTTGAAAAGATCAAGGAAATTCTACCACCGGATCATCACTGGAACATATTTACTGCATAA
>VviMAPKKK42    VIT_17s0000g08140
ATGAAGGAGAGCAGTGATGGGTTTGTGAGGGCAGATCAGATTGATCTCAAGAGCTTGGATGAGCAACTGGAGAAGCATCTT
AACAGAGTATGGACCATGGACAAGAACAAGAAGAAAGAGGATGACTCCTCCTCCGCCGCCGCCGCCATCCCCACCCTCGCC
CCCTCCACCACCGCCTCCACCACCGCCCCCACCACCGCCCGACAAGACTGGGAGATCGACCCCTCTAAGCTCATCATCAAG
ACCGTCATTGCTCGTGGCACTTTCGGCACTGTCCACCGTGGCGTTTACGACGGCCAAGATGTTGCCGTTAAACTGCTTGATT
GGGGGGAAGAGGGTCACAGGACAGAAGCTGAAATTGCTTCACTGAGGGCGGCTTTTACGCAAGAGGTAGCCGTGTGGCAT
AAGCTTGACCATCCTAATGTAACAAAGTTTATAGGGGCGACGATGGGCTCAGCGGAGCTAAACATACAAACGGAGAATGGT
CATATAGGCATGCCAAGTAATATTTGTTGTGTTGTTGTGGAGTATCTTCCAGGGGGTGCCCTGAAATCTTATCTCATAAAGAAC
CGAAGAAGAAAGTTGGCTTTCAAAGTTGTCATTCAGCTGGCACTTGATCTTGCAAGAGGGTTGAGTTACCTTCACTCTCAGA
AGATTGTTCACAGAGATGTAAAGACAGAGAACATGCTATTGGACAAGACTCGAACTGTAAAGATAGCTGATTTTGGAGTTGC
TCGGGTTGAGGCTTCAAATCCTAATGACATGACTGGGGAGACTGGGACCCTTGGTTACATGGCTCCAGAGGTCTTAAATGGA
AGCCCATATAACAGGAAATGTGATGTGTATAGTTTTGGTATCTGTTTGTGGGAGATATATTGCTGTGACATGCCATATCCTGAC
CTCAGTTTCTCAGAAGTGACTTCAGCTGTGGTTCGCCAGAATCTGAGGCCAGAGATACCAAGGTGTTGCCCTAATTCTTTGG
CAAATGTAATGAAGCGATGCTGGGATGCTAACCCTGACAAACGACCAGAGATGGATGAGGTGGTTGCTATGATAGAAGCCAT
TGATACATCAAGAGGAGGAGGTATGCTTCCTGTTGATCAGCCTCAGGGCTGTTTTTGTTTCCGCAAGTATCGAGGTCCTTGA
>VviMAPKKK43    VIT_14s0066g01400
ATGAAGGAGAAGAGTGACGGGTTTGTGAGGGCAGATCAGATAGATCTGAAGAGCTTGGATGAGCAGCTTCAGAGGCATCTC
AGCAGAGCATGGACTATGGAGCAGAAGAAGGAGCAGCAGGAAGAGAGGCCCAATACTCGAGAAGAGTGGGAGATTGATCC
TTCCAAGCTCGTCATCAAGAGTGTCATCGCTCGTGGCACTTTTGGGACGGTTCATCGTGGGGTTTACGATGGCCAAGATGTT

GCAGTTAAACTTCTTGATTGGGGAGAGGAGGGCCACAGAACAGAGGCTGAAATAGCTTCACTTAGAGCAGCTTTTACACAA
GAAGTTGTTGTATGGCACAAGCTTGATCATCCTAATGTAACTAAGTTCATTGGGGCTACAATTGGCTCATCAGAGCTAAACAT
ACAAACAGAAAATGGTCACATTGGCATGCCAAGTACTGTATGTTGTGTTGTTGTTGAATATCTTCCTGGGGGAGCACTGAAA
TCTTACCTCATAAAGAACCATAGAAGGAAGCTGGCTTTCAAAGTAGTTGTCCAACTGGCACTAGATCTTGCACGAGGGTTGA
GCTATCTTCATTCTAAGAAGATTGTCCACAGAGATGTGAAAACAGAGAACATGCTTCTGGACAAGACTCGTACTTTAAAAAT
TGCTGACTTTGGGGTTGCTCGCATGGAGGCTTCAAATCCTAATGACATGACAGGCGAGACTGGAACCCTTGGTTACATGGCA
CCAGAGGTTCTCAATGGCAACCCGTATAATAGGAAATGCGATGTGTACAGTTTTGGTATCTGCTTGTGGGAAATTTATTGCTG
TGACATGCCATATCCAGATCTCAGCTTCTCAGAAGTGACCTCAGCTGTTGTCCGCCAGAATCTGAGGCCTGAGATACCTCGTT
GTTGCCCGAGCTCTCTTGCGAATGTAATGAAGCGATGCTGGGATGCTAACCCTGACAAGCGCCCAGAGATGGACGAGGTGG
TATCGATGCTGGAGGGCATTGACACATCCAAGGGTGGAGGTATGATCCCTCTAGATCAACCTCAAGGCTGTTTCTGTTTTGGC
AAGTACCGAGGGCCTTGA

>VviMAPKKK44    VIT_07s0151g00500

ATGGATTTGAGAAGTGATGATGAGGTTTTAGTGACTGCAAAAGCTGAGAAATCCAGGACTGGAGAGGGAGAAACTGTCTCA
GACAAGTCTAGTATTTCGAGTAGTGATTTGGGTTCAAAGAAGGATGGGGGGAATACTAGTAACAAGGATTTATTTCTTAGAG
CAGATAAAATCGATTTCAAAAGCTGGGATATCCAGCTGGATAAGCACTTGAGTCGAGTTATTTCAAGGGATAGGGAAGTTAAT
ACCAATACAAAGAAGGAAGACTGGGAAATTGAGTTGTCTAAATTGGATATAAGAAGTGTTATAGCTCATGGGACATATGGTA
CTGTGTACCGAGGCGTTTATGATGGCCAAGATGTTGCAGTGAAGCTGTTGGATTGGGGGGAGGATGGTCTTGCCACAGCTGC
TGAAACTGCTGCTCTTCGGACCTCGTTTCGGCAAGAGGTTGCTGTCTGGCATAAGCTTGACCATCCAAATGTTACAAAGTTT
ATTGGAGCTTCGATGGGAACATCTGATCTTAGGATCCCTTCAAATAGCATTTCAAGTGATGGTCGTAATCCTGTTCCTTCCAG
AGCATGTTGTGTTGTAGTCGAGTACCTTCCTGGTGGGACGCTAAAGAAATTTTTAATCAGGAATAGGAGAAAGAAACTTGCC
TTTAAGATTGTAATTCAACTGGCTTTGGACCTCTCTCGAGGTTTGAGCTATCTTCACTCAAAGAAGATCGTACACCGTGATGT
TAAAACAGAAAATATGTTGCTTGATGCTCATAGAACATTGAAAATTGCTGATTTTGGTGTTGCTCGAGTTGAAGCTCAAAATC
CAAGAGACATGACTGGGGAAACCGGAACCCTTGGTTACATGGCCCCAGAGGTTCTTGATGGTAAACCATATAATAGGAAGT
GTGACGTCTACAGTTTTGGCATATGCTTATGGGAAACGTATTGCTGTGACATGCCTTACCCTGATCTTAGCTTCGCCGATATAT
CATCTGCAGTTGTTCGACAGAATTTACGACCAGATATCCCTAGATGTTGCCCAAGTTCATTGGCAAGCATCATGCGGAAATGT
TGGGATGGGAACCCTGACAAACGTCCTGACATGGATGAGGTGGTGAGGTTGTTAGAAGCCATTGATACGAGCAAAGGAGGC
GGCATGATACCAGAAGACCAGGTTAGTAGCTGTTGCTTTTTCTCTGTGGCTCGTGGTCCCTGA

>VviMAPKKK45    VIT_08s0058g01180

ATGGCCCCCCAGTTAACAATTGATGAGAATCTTCTTGTTGATCCAAAATTAATTTTTATCGGATCGAAAGTTGGTGAAGGAGC
ACATGGGAAAGTTTATGAAGGAAGGTATGGTGATCAAATTGTTGCTATCAAAGTTCTCCACCGTGGAAGCACTTCAGAAGAA
AGAGCTGCACTTGAGGGACGTTTTGCCCGGGAAGTTAATATGATGTCAAGAGTAAAACATGAGAATCTTGTCAAGTTTATTG
GAGCTTGTAAGGATCCTTTAATGGTGATAGTTACAGAGCTATTACCAGGTATGTCACTTCGGAAGTATTTAACCAGTATTCGTC
CAAAACGGATGGACATTCATGTTGCATTAAGTTTTGCTCTTGACATTGCTCGAGCCATGGAGTGTTTGCATGCCAATGGGATT
ATACATAGAGATCTTAAACCTGACAATTTGTTGCTTACGGCCAATCAGAAATCTGTAAAGCTTGCAGATTTTGGTCTTGCAAG
GGAAGAATCTGTGACTGAGATGATGACCGCAGAAACTGGGACATACCGTTGGATGGCTCCCGAGCTGTACAGCACTGTGAC
ATTGCGTCAGGGTGAGAAGAAGCATTATAATAACAAGGTTGATGTCTACAGCTTTGGGATTGTCTTCTGGGAATTATTGACCA
ATCGCATGCCATTTGAAGGCATGTCAAATTTGCAGGCCGCTTATGCTGCTGCATTTAAGCAAGAGCGCCCTAGTCTTCCAGAA
GACATATCCCCTGATCTTGCATTTATCATACAATCTTGTTGGGTTGAGGACCCTAACATGCGACCCAGCTTCAGCCAGATCATC
CGCATGCTGAACACATTTCATTTCAAAGTCACACCACCTCCATCATCATTACTAGAAGAATCTGACACCAATGGAGCAGCAAT
GTCTAGCAATGGTACCATGACCGAGTTGTCCGCACGTACGAGAGGGAAGTTTTCTTTCCTTCGCCAACTTTTTGCTGCAAAG
AGGACAAGGAATTCACAGTGA

>VviMAPKKK46    VIT_04s0023g01350

ATGCCTGTTGCCATAAAGATGATACAACCGAACAAAACGTCAGCTGTGAGTCCTGATCGAAAGGAAAAATTTCAGAGGGAG
GTTACAATACTATCTAGGGTTAAACATGAAAATATTGTGAAGTTTATTGGGGCATCTATAGAACCGACTATGATGATAATTACA

GAGCTCATGAAAGGTGGTACACTTCAGCAGTACTTGTGGAGCATTCGTCCAAATTCTCCAGATCTGAAACTGTCTTTAAGTT
TTGCTTTGGATATTTCTCGGGTTATGGAATACTTGCATGCAAACGGCATCATTCATCGTGACTTGAAACCTAGCAATCTGCTCC
TCACAGAGGACAAGAAGCAAATTAAGGTTTGTGACTTTGGTCTAGCAAGAGAGGAGACAGCTGGTGATATGACGACTGAG
GCTGGTACTTATCGGTGGATGGCTCCTGAGTTGTTCAGCACAGTTCCACTTCCAAGGGGTGCAAAGATACACTATGATCACA
AGGTGGATGTGTACAGCTTCGCCATTATTCTGTGGGAGTTGCTAACAAACAGAACTCCATTCAAGGGGGTACAAAGCATACT
AATAGCATATGCTGCAGCTAATAGAACTTGTACTACAACACCAAAAGGAGACAAACCCTAG

>VviMAPKKK47    VIT_04s0008g03020

ATGGTGGAGGGTCCGAAATTCACTGGAATTATTGGCGGAGGGGGAAATCACAACCATGATAACAATTACTTTGATTTCACTC
AAGGCTTCTATCAGAAACTTGGGGAGGACTCCAACATGTCTATTGACAGTTTGCAGACCAGTAATGCTGGCTTGTCCGTGTC
GATGTCTGTGGACAACAGTAGCGTTGGGTCCAATGATTCTCTTACCCATATCCTAAACCACCCCGGTCTGAAGCCTGTGGCTA
CCCACAATTATTCTGTGGGGCACAGCGTCCTTCGTCCCGGGAAAGGTAAGGTCACCCATGCTCTGAATGAAGACGCACTTGC
TCGAGCTTTGATGGATACTAGGTATCCAACTGAGGGTTTGGAGAATTATGATGAATGGACCATTGATTTGCGCAAGCTCAACA
TGGGGACGGCTTTTGCTCAAGGGGCTTTTGGGAAGTTATACAGGGGAGAGTATAATGGGGACGATGTGGCGATTAAGATTTT
GGAAAGGCCGGAGAATAGTCCTGAGAGGGCACAGGTGATGGAGCAACAATTCCAGCAAGAGGTGATGATGCTTGCGACAC
TGAAACACCCCAATATTGTGAGGTTTATTGGGGCATGCCGTAAGCCCCTTGCTTGGTGCATTGTGACTGAATATGCCAAGGGA
GGGTCAGTTCGGCAGTTCTTGATGAGGAGGCAGAATAGGTCTGTGCCGCTGAAATTGGCTGTCAAGCAGGCATTGGATGTT
GCAAGGGGGATGGCATATGTGCATGGACTCGGGTTCATCCACAGGGACCTGAAGTCAGATAACCTCTTGATTGCTGCTGATA
AATCCATTAAGATTGCTGATTTTGGTGTTGCCCGGATTGAGGTGCAAACCGAAGGAATGACACCAGAGACAGGGACGTATCG
CTGGATGGCCCCAGAGATGATCCAGCACAGGCCATACACTCAGAAGGTAGATGTCTACAGCTTTGGAATTGTCCTGTGGGAG
CTCATCACAGGCTTGCTTCCCTTCCAGAACATGACTGCAGTGCAGGCAGCCTTTGCTGTGGTGAACAAGGGAGTGAGGCCA
ATTATCCCAAGCGACTGTCTTCCTGTTCTAAGCGATATCATGACCCGCTGCTGGGACGCCAATCCTGAAGTGCGTCCTCCCTT
CACTGAGGTTGTCAGGATGCTTGAGAATGCAGAAATTGAGATCATGACAACTGTGCGGAAGGCACGCTTTAGGTGCTGCAT
GACCCAGCCTATGACAACCGACTGA

>VviMAPKKK48    VIT_12s0028g02130

ATGGAGGAGACACGAGATGATGCAGGGCCAGCAGAGCAAGGGTCTCCTGTTACAACATGGTGGCCATCAGATTTCATTGAA
AGATTTGGATCTGTTTCTCTGGTTTCTCAAGAAGAAATTTTGAGTAATAAAAATTCTAATAGCAACACTGAACAAGATGAGCT
GTCATCTCAGACAGCCTCACAAATTCTCTGGAGCACTGGAATGCTTTCTGAACCGATTCCTAATGGTTTCTACAGTGTCATTC
CGGACAAAAAGCTTAAGGAGATTTTTGATGATATTCCTACTCTGGATGAGCTTTATGCTTTGGGAAGCGAGGGTGTCAGAGC
TGATATCATTCTTGTAGATGCTGTGAGAGATAAAAAATTATCTATGCTGAAGCAACTAATTGTGGCATTGGTGAAGGGACTAA
ACTCAAATCCAGCTGCAGTGATTAAAAAGATTGCTGGATTGGTTTCAGATTTCTATAAGCGGCCAAATTTGGAACTAAGTCCA
GCAAAAGCTGCACTAGAGGAAACCTCTCATGTGTCTGAGAATCGAGTAGCCCAGTTACTGGGCCAAATAAAACATGGTTCTT
GCCGTCCTCGGGCAATTCTATTCAAAGTTCTTGCAGATACTGTCGGTCTTGAAAGCAGGCTCATGGTGGGTTTACCTAATGAT
GGGGCTATTGGGTGCGTAGACTCATATAAACATATGTCTGTGATAGTCATGTTGAATTCTGGGGAACTACTGGTCGATCTGATG
CGATTTCCTGGCCAGCTGATACCTAGATCAACCAGGGCAATTTTTATGACCCATATTTCTGCAGCAGGGGAAAGTGACTCTGC
AGAGAATGATTCTTGTGATTCTCCCCTAGAACCAAACAGTCCTTTGTATGGTTTTTCAGATAGAGTTGACCCTGACAGTACTG
AGAAAGATGAGGGCCTTCAGTTCCAACGGAGATTAGAAGCATCATCAAATGTATCAGGCCCTTCTTTACGGAATGTAATGCT
GCGATCTACCCCCTCTATTGATAGAAAATTGAGTTTATCGCATAGTGAACCCAATATTGCTACTACTTTTTGGCGACGGAGCCG
GAGAAAGGTCATTGCTGAACAACGAACTGCCAGTTCAAGTCCAGAGCACCCTTCATTTCGAGCACGTGGGCGGTCAATGCT
TAGCGGTGATAGGAAATCATTTAGAGATTATGCTGATGACATAGCTGCCTCAAGCTATAGGTCAGATGGTGCATCAACATCAA
CGTCAGAAACTCGAAGAATAAGAAGAAGAAGCATTAGTATTACTCCAGAGATTGGCGATGATATCGTAAGGGCTGTACGTGC
AATGAATGAAACACTGAAGGCTAACCGTCTTATGAGAGACCAAGGCGATGACAGGGCATTTTCCAGTAATCCAGATATTCAA
AAAAATGTATCTGATTTCCATCTTGATGGTCATGGGGAAATATCTCATGGAAGCTCTTCTATGTATACCCTGCCTAGGGAGCAA
ATAAGTTCCCAAAAGGCAATATCGTTGCCTTCATCTCCTCATGAATTTAGGAGTCAGACTTCTGGGAGAAGTGGAACTTCAG
ACATAGTGAATGATGAAATGGTTTCAATCTGGAACAGGGTTCTTGAAAAACCAATGTTCCATAGCAAGCCTCTATTACCTTTC

CAAGAATGGAATATTGATTTCTCAGAATTAACTGTTGGAACTCGTGTTGGGATTGGGTTCTTTGGAGAAGTTTTTCGTGGCAT
TTGGAATGGGACAGATGTTGCCATCAAGGTTTTTCTGGAGCAAGATCTTACTGCTGAGAACATGGAAGATTTCTGCAATGAA
ATATCCATCCTTAGCCGCCTTCGACACCCAAATGTTATTTTATTTTTGGGTGCATGCACAAAGCCTCCACGCTTGTCAATGATA
ACTGAATACATGGAGATAGGATCTTTGTATTACTTGATCCATTTGAGTGGTCAGAAGAAGAAACTCAGCTGGCGTCGGAGAA
TTAAAATGCTGCGTGATATATGCAGGGGTTTGATGTGCATACACCGGATGAAGATTGTCCATCGTGACATAAAGAGTGCAAAT
TGTCTCGTCAATAAGCATTGGACTGTCAAGATTTGCGATTTCGGGCTCTCAAGAGTAATGACAGACACACCCTTGAGAGATT
CCTCATCTGCAGGAACTCCAGAATGGATGGCTCCTGAACTCATCCGCAATGAACCCTTCACAGAGAAATGTGATATTTTTAGC
TTTGGGATGATTATGTGGGAGCTCTGCACTCTTAATAGACCATGGGAAGGCGTGCCACCAGAACGGGTGGTATATGCTGTTG
CTCATGAAGGATCACGGCTGGATATTCCTGAAGGCCCCCTGGGCATGCTTATAGCAGATTGCTGGGCAGAACCGCATCAAAG
GCCAAGCTGTGAGGATATCCTCTCCCGCTTGCAGGACTGCGAGTATACCCTCTGCTAA

>VviMAPKKK49    VIT_11s0052g01480

ATGTGTAATAAAGGAATTGCACGCGTGAGCGACTCTGTTGATCAGAAACAGCACCAGGCTGTGTATTTGATGGATAGCCCTT
CTGCGACTCCTAGTTCAGCACATGGGTCGAACGATGAGAACCCTCGTGTGAAGTTTTTGTGTAGTTTTTCTGGTAGTATATTG
CCCAGGCCACAGGATGGGAAGCTGAGGTATGTGGGTGGGGAGACGAGAATTGTAAGCGTGCCCCGGGATATTGGTTATGAG
GAGCTGATGGGGAAGATGAAGGAGCTTTTTGATATGGCGGCAGTTTTGAAATACCAGCAACCTGATGAGGATCTTGATGCTC
TTGTCTCGGTTGTGAACGATGATGACGTGACCAATATGATGGAGGAATATGACAAGTTGGGTTCTGGGGATGGGTTCACTAG
GCTTAGGATTTTTCTGTTTTCGCATCCCGACCAAGATGGTGGTTCTTCGCATTTTGTTGATGTTGATGATACTGAGAGGAGGTA
TGTGGATGCTTTGAATAATTTAAACGATGCTTCGGATTTTAGGAAGCAGCAGGTGGGTGAGTCGCCGACTATGAGTGCAATT
GATGATATTCATTTGGCCGAACAGTTCTTTAACTCCATAAGTCTTGAGGGTGGCCTCCATAACCAGAGAAACTGTGAGATGCC
TATGTCTCAGTTCAACTTGCATCACCTTACAATTCCTCATATGGGGTCGGGGCAACACCAACCTGTTGCTCAGAGGTATAATG
AAATGGAGTCTCAGTGGAACCCTGCCTACTTTTCCCCTAGGCATCACGGACACCATGATGCAAGGCCGCTGGCAGAGTATCC
ATCTTCACCTTCATCTGCGAGGTTCCGCATGCCATTTGGGGAGTTACCAGATAAATGCATTGATAGACTGCCTGAAGAATACA
GTCGGCAGCCAGTGAATCCCCAAGCTCCATATGATCATCAACCACAAGCTTCAGATAATGTTGTATGGCTGCCAACAGGAGC
TATATCCAGTGAGAAGGCAGGTTTTCCGGGTAGCATGCTTCATGGCCCCAATGTTTTTGAAGGGAATAGTATCTGTGAGCATT
GCCGGATGACTTTCCATAGACATTTAGAGCAACCCAACATGGGGAATGGATTGCCTCCAGTTGCTAATCCATGTGCAGAGTG
CCCCCCAGGTAGGGAGTCTTTTTTGTTGAATACAGATGCAAAAATGCAGCATGGAATATATCCAAAAGAGCACAATGATCCT
CGATCTCTGTATAATGAAACTCATAATCATGAACGGGGATGGATTCTACAGCATCAGTTGAATCCTCGGGCTGAGGATGCAAG
AGCTCAAATCTCTGGAGCTGGAAGGTTGAATGATCCGTACATTGTAGATGGTTCTGGCGTGAATTTTCCAGTTGCACATGGTA
ATTTGCTGGATAACCATCATGTGTCTTCAAATTATGTCCATCATGAAGACCCACGGTACATTCGAACTGGACCTGAATTGGGG
AATGGAGTATTCCATGACCAAGCTGCAGCTGCTGGCCCTGCTATCAATGTTCCTCCTCTGGAAGAGCGTGCTGTTCGCTATGG
CAATTTGCCTTATCCTTATGGAGCTGATAATCTTTACCAAGTCTCACACGGACATGTACCTGCACATGCTTTATGGAGAAATGT
TCAGAACCCAATGCATGGTGCTCCCTCTTATGAAGCATCCACTTCTACCTGTCAAGCAAGTGGTTCAGTTAATCCAGGACCCA
TTAGGGGTACACGGGAGGGTAGTCCTAGGTTCTGTGTTGGGTTAGATAATCAAAATCCTTGGGGTGAATCATCACAGAAAAT
ACTAGGTTTTGATGGCTCTGCTCTCCCTGATTATTCTTATGGCCATGCTACAAAATTGAACCCAAACACTCATGGTCAGGAAG
GCCAACACCCATTTACTCCAGGTCCTGTTCCATCCCCATCTGACATGTTAAAGTTTGCTGCTCCTATGGAACCATTGCATTTCA
CAAATTCATCTCCAACTTTAATGGATGATAAGTTTGTTGCTTCAGCCAATTTGAGTTACAATCCAGAATCAAGGAATGATAATA
ATGTAAATCAAACAGTTATAATGGAGGCTAAACAAGCTTTTAGAGAAGGAAAAGAAGAGATTCACATGGAGAAGGTTGAAG
ATAATGATATGCCAGTTACCTCCCTTCCAGAGAAAAACAATAATGCAGATAAGAAATGTGAGGTGGCATCTCTGGAGCCTGT
CAATTTACCAGCTGAAGATAATGTTTTTAAACCTGTTGTCAACGATTGTGCTCCTCTTGAAGAAGATGCTAAACTGGATGTTA
GCAATTTGTCTTTCTTACCAGAGTTGATTGCTTCTGTCAAAAGGGCTGCATTAGAAAGTGCTGAGGAGGTGAAAGCTAAAGT
TCAAGAAAATGCTGATGCAGTGCATGCTTCATCAACTAAAGAGGCCTCAAACGAATTGGAGACAGCCAATGCCCTGGGGGA
TTTGGAGTTGGATTCTGACAATGATAATGTGAATACATTCAAGATTGAGCCAACAAAGGCTGAAGAAGAAGCTCTTTCAAGG
GGATTACAGACAATAAAAAAATGATGATCTGGAGGAGATTCGGGAATTAGGTTCTGGTACGTATGGAGCTGTTTACCATGGAA
AATGGAAGGGGTCTGATGTAGCAATAAAGAGAATCAAAGCCAGCTGCTTTGCTGGGAGACCATCTGAAAGAGAACGTTTGA

TTGCGGATTTCTGGAAGGAGGCTTTGATATTGAGTTCATTACATCATCCAAATGTCGTTTCCTTTTATGGTATAGTTCGTGATG
GCCCTGGTGGCTCTTTAGCAACTGTGACAGAATTCATGGTTAATGGTTCTTTGAAACAATTTTTGCAGAAAAAGGACAGGAC
AATAGATCGTCGGAAGAGACGCATCATAGCTATGGATGCTTCATTTGGAATGGAGTATTTACATGGCAAGAATATTGTACATTT
TGACTTAAAATGTGAGAATTTATTAGTAAATATGAGAGATCCACATCGGCCTGTTTGCAAGATTGGTGACTTGGGTTTATCAA
AGGTTAAACAGCATACGTTAGTATCAGGAGGTGTTCGTGGAACACTTCCTTGGATGGCACCAGAGCTTCTTAGTGGGAAAAC
CAACATGGTAACAGAAAAGATTGATGTATACTCATTTGGAATTGTGATGTGGGAATTGCTTACTGGGGACGAACCTTATGCAG
ATATGCATTGTGCTTCTATAATTGGAGGAATCGTGAACAACACATTGCGTCCACAAATTCCAAGATGGTGCGAACCCGAATGG
AAATATTTGATGGAGAGTTGCTGGGCTTCCGATCCAGCAGAGAGGCCATCATTCTCAGAAATCTCTCAAAAGCTGAGGAATA
TGGCTGATGCCCCAATTACAAAATAA

>VviMAPKKK50    VIT_11s0016g04880

ATGAAAGAACCCTCTATGTTGCGCCGGAGGAGACCCAATTCGTCGAAGAAACTGAAACTCGTTTGCAGCTTCAATGGCCGA
TTCCAGACGCGTCCGCCCTCTGGTAAGCTTGGTTACGTCGGCGGCGATACTCGTATCATCTCCGTCGACCGAGGCATCGGATT
CATGAAACTTCGGTCTAAGATCTCAGAGCTCTGCCCCGATATCCGTTCCTTCTCTCTCAAATACCGCCTTCCCGAGTCGGATC
CTGTTCACGGTGACACCACCAATCTCGTTTTGATAGCTTCCGACGACGACGTTCGTTGCATGGTCGATGAGTACGATAAGATG
GACTTCTACGGACAGCAAACAAGATTGAGGATTTTCGTTTTCCGTGATAATGGGTATGTTAACGTGAATCTCCCCATGAATTG
TATTGAAAACATAAATGATTATGTGTGTGGGAAAAAGGGGTTTGGTGTTGAGGGGAAAGAGACTTGTGTGAAGGGTGTGAG
TGATTTTGGTGATTATATTAGTAATGTGTTTCAAAGTCGAAATAGTGGGAGCTATTTGTTTGGACTCCAGTTTGATACTAAGGA
AATCAACAATCCGGCGACTGTTGTGGCCGGAGGCCGGTATAGTGATCGTTCACTGAGAAAAGTGATCTTGAAACAACGGTT
TTCGGCAAAGAAACCGGCTCCAATTAGCAGTTTTTGCAGTGGGGAAAGGGAATTTAGGCGTTCTGACGAGCAAAAATATTG
TTACCCACTTATAGATTTAGCCCCTGAAGCATTAGTCCCAAAGAGTAAACAAACTGCTAATCTGAATTTTGAACACCCGCGTG
AAGTGAATATTTTGATTTGCAAAACTGAGGATGCCCTTTCTCCAGGAAATCAAAATTTTGAAAATTTGGTAAGGAATGGAAAT
ACAAGGGTGGATGGGAGTAGTCCCAGGCAGTGTTTGTTGAGGCTGTCTGGGTGTAATGGAGGTGGAATGAATCAAGGCATT
TCAAATACATCCAGTGAGGTGGTTCAGTTTCCACAGCTGTCATGTATTTCAAGTATCATTTCGTTGTCAAGTGGTTCTAATGCT
AAGCAGGACTTGAGAAACATGGACCCTATGTCATGGACTAATTTCAACAGGAAAAACATGCCATGTCCTGCAAATTATGATT
CAGGGAAGATTAGTTTATTGCCGCTTTCTTGCAGCAATGAGATGGTGGGAAGTGCTTCTCCAATGAAAATCCTGTCTGCTAG
AGATAGGGCGTTGGGGGGTGATCTTCAGAGTGGCATCCGCAAGCATAGGTTTGGCATGTGTGATACCAGAAATCATCGGATG
TGTTTATATCACATACGTAACCATCAGAGTAATCTCTCTGAGATGGGTAGCAATCAAAATTTGAGGTTGGACGGAAGGTCTTG
GTCAGGGCGATGCTGTCCTGGGCTCAGACCAAACCCAAACATAGCAAAACAGGGGCAATCTATGAGATCATACCATCCAAAT
TATTTGAAGCCATTGTCTTGCACACACACCCATACACTGCAAGGATTGATGAGAATGATGGACTCTAGCTTAAATAGTCACTC
TTGCTCCCATGATCTCCAATATGCCAATGAGAACATAAGAGACCAGGGTATCCTTGCATCTGAATATGGAAGCCCAAATATTG
AGGAATGTAAATTTGCCTACCATGGTGCATATGCTGGCATGGGCAACCCACCCCTCTTGTTTCGTAATGCTGTTGAGAACCCA
TTGAAGGACGGTTTCTTAACTGATTCTGGTAGTGGGATGCATGAAGTTCCACATCAGAACCCTTACCAGAATTGTCACAGAG
TGCTCACAAATTGTGAATCTGGTTGTTATGATTCAAGGCAACCATTCTCATTGAGCCCTCAGAAAGTTGACAATATTTCTGCC
TTTCTGAATTATCCAGGTTATAGCCAAGGAACAGAACTGAGATGCAACAGTAAGCTGTCAGATAGGGAAGCAGGTATTGAGT
CATTAAGCACTCATCGTGATGGTGCCCATACTTTACAAGGTGGAGTTGCATCTCCAGTTGATCTTTCATTGGGTAATCTTTCAT
TGTCATCATCCAAAGAAGTGGAACCTCTTGCACTTTCTTCTCATGTTGACATTGATGTTTCTGAAGCCCTACTAAAGTCTCAA
TCAAAGCATTTGGATCTCATAGATGGACACTCAAGCCCAGAAGCTTACAACTCAAATGGAATGGAATCCGGTTCTCTCACTG
GGAATGCAACCAAATTGGGAAATGATTATGTGCATAAAGAAGAAATCCAACTGGACCCTTCATCTGATTTAAGCATTGATGA
AAAGAGGGATTTAAGCTTCTTATATTTTTTGCAGACTATAAAAAATACTGACCTGGAATACATAAAAGAACTTGGTTCGGGTA
CTTATGGAACTGTATCTTATGGAAAATGGAAGGGGTCTGATGTTGCCATTAAGAGGATAAAACCCAGTTGCTTCACAGAAGA
CACATTGGAGGAGGACCGATTGGTTGCAGAGTTCTGGAAGGAGGCCCACATACTAGGGCAGCTTCACCATCCAAATATTGTG
GCATTCTATGGTGTAGTTACAGATGGACCTGTGACAAATTTGGCAACGGTAACAGAATACATGGTGAATGGCTCTCTTAAACA
AGTGTTGCAAAAAAAAGATCGAACAATTGATCATCGGAAGAGATTAATTATCGCTATGGATGCAGCCTTTGGTATGGAATATC
TACATGGGAAGAACATTGTTCATTTCGATTTAAAGTCTCATAACTTGTTCATGAATATGAGGGATCCTCAGCGACCAGTTTGC

AAGATTGGTGATCTGGGCTTGTCAAAAATTAAGCAGAGGACCCTCATCTCTGGTGGGTTACGAGGAACTATACCATGGATGG
CTCCAGAACTTTTTAACAGTAAGAATGACTTGGTAACAGAAAAGGTTGATGTATACTCATTTGGAATAGCCATGTGGGAACT
CTTGACTGGGGAGGAACCTTATGGAAATAAGATTTGGGTTTTCTTTTTCAGCTGGTATAATCAAAGGCAATCTGCGACCCAA
AATTCCAACCTGTGA

>VviMAPKKK51    VIT_11s0118g00790

ATGGCTTTTGATCAGAACTCGATTCCCTTAGATCTGAGGCCATTGAATGTTCCGCGAACGATGGTCGAGGACCCTCGCATTGC
CCCCGCCACAACCACGGGCCGGACCACAGAGGGGGTTTTCCCCAACCCAGCCCGTGATGCTGGTAGCCCAGGGTCGGTTCA
GATGTTCTATCCGGCCACTGTGTCGGATGCTGGGTTGGTAGGTTTAGGGTTTGGAAATGCGGTGCCAGGTGTTGCCGCTTGG
TGCCCTCATGTGCCTGTGGCCATTGGGCGTGCTGGAATTAGTCCAGGAGCGATTGGATTGGGCTATAATCCCAATTTGGGGAC
TCGGGTTGCTGGCAATGCCTCTGATCAGGCAAGCGATGAAGGTACAGATGACTCGAATTCAGGGAAGAAAGTTAAGTTCTTA
TGTAGTTTTGGGGGAAAGATTTTGCCTAGGCCAAGTGATGGAATGTTGAGATATGTCGGAGGGCATACAAGAATTATTTGTCT
GAGAAGGGACGTGAGCTTTAATGAGTTGGTGCAAAAGATGGTGGATACATATGGGCAACCGGTGGTAATTAAGTATCAGCTA
CCCGAAGAGGATCTTGATGCATTAGTGTCAGTTTCTTGCCCAGATGATCTTGAAAACATGATGGATGAGTATGAGAAATTAGT
TGAGCGATCATCTGATGGATCAGCTAAGTTACGAGTGTTCTTGTTCTCAGCTTCAGAGCTTGATCCCTCTGATATGGTGCAATT
TGGAAATTTTAATGATAGTGGGCAAAGATATTTTGATGCTGTGAATGGGATTATGGATGGAATTGGGGGTGGTATTGCTAGAA
AGGAGAGTATAGCAAGTGCAACTTCCACACAGAATTCTGATGTGAGTGGAAATGATGCCACTGATAACTTGGTTCAGCATCA
AGGGGATGTTAGTGGGCCACCGTTTAGCAGTGCATTATCTCCCAAAGGAAATTCGGCTACATCTAATGAACCTGCTACAAGAT
TGATGTGTGTGGATCCCAACCCAGCAATCTATGCAGATGTCTCTGCCATTCCATTGGGAATTCCAGTGGGTAATACTGGTCCT
CCCCAGACTTCATCTTCTAAGCCTGATGTTGAGTTTGAGAGATCTGTACCCCTTACTGTACAGCCACAGCAAGTGGGGTTTGA
TTTGCAGCAATGCCGGATGGATATTCCAGCAACCACAGCTTACTTGCAGTCTTATGTGCATCCTCATCGAGAGGTTACTAACC
ATGCTGATTATGTTCAAGTTCCTCACCAGATGGGGTTCCCAAATCAGCTGTTGGCAACTTCTGGTTCTGTACTGACCCACCAG
CAGATCCGTGACAATGCTAGTGGTGTTAGCTCTCATCAATTTATTCCTGCAGTGCACATGACAATGACCCCTACAGCTTCTCA
TGTCAGTATCAGACCAAGTGTGATTCAGCCATTGGTTCAGCCCCAACAGGCCAGGATAGATTGTTACACTGATGAAAGTACA
TTTGGGCCAAGGGTTGTCCAGCTTCCACTAGACCAAAGCTATAATCCATATCAAGCCCAGGTCCCACTCCCTCCTGCAGTGG
TGGGAGGCTATGGCTGGCATCAAGTCCCAGCACAGGACCATGTAGTCTTATCTGATGGATGGGCTCATCAACAAGTAATTCTT
CCAGAGACAACCACAAGGTTGGAGGACTGTTTTATGTGTCAGAAAGAATTGCCTCATGCACACTCTGATCCTTTGGTACAGG
GACTGAGAGACAGCAGTGCAAGCTCTGTATCTGATTCAAACTCAGCGTATCATAGCCTCCGATTGGAGGACAATGTGAGAGC
CCGTCAAATAAACAGGGTTGTGGTAACTGGAGCCTTGGGGGAAGGCATTATTGAACAAGGAGTTGGTGCTCAGCCGAGGGT
TCTTGGTCATATGGATCATCAAGCTGGGACACTTCAATCAGAGGTAGTTGGGATCTGTCAGAACCTTGATGCGCAGCATGAA
AATGAGAAAATTATTCTCCAAAAAATGGACAACCCTGATCAACCCAGAGTCCCAATTCCCCAGGGTGTGGTGGGGTTGGCA
GGTGCTGTGCAGTCATCTTATGGTGTATTCACGGGCACTATTCCTCAGACTTCCCAAGAAGAGGCTGTCCAGCAGTATGCAG
TGCCAACCCAGTACCAGGTTAAACCGGACACCTTAGTGAATAGACCAATTAATAGTGATGTCCCTCTATTTGGAGGTGTGCCT
TTACAAACATCAGAACGCCTGGTTCAGGAATCTCCAAGAGACTATTCTGGTAAACTTCCTGGTGTTGTTCCTAAGGAAGATA
CTGCAGAGTCTTGCATTTCATTTGATCATATGCGTCCAATTGATGAGAGGATGGAAAATCTAAGGGTAGGCCCTGCTGAAAAT
TTTGTTAATAGTGAGCAGAGTAAATCATCTGCTGATAAACCTAGAAAGGAGGACATCTTGGAACACAGACTGCAGCAAATTG
CAGGGAAAGAGGTGCTTCTGGACAGTACATTCAGCAAAGCCAAAATTGTTGTTGAGTCGAATCACAATAAAGCAACTGAGG
TGTTGCCTTGCTCTGCTGCTGAAGTTCCTTACCTGCATAATGTTTGGCCAGTGGAGACATATGAAGTAACAAAACTGCCTATT
TTGGGAACTCTGGCGACATATACACATTCTAAGACTGGGATTCATAATGTGACTTCTGGTGAAGTTTCTTATGGCAGCCCTGC
ATTTTCCGATGTTGAATCGGCTTATCTAACAGATAAAGCTCCACCCATATCTGAATGGAATGATGACACCTCGCAGTTTCAGC
CAAAGATGGTTCCTACAGATATCAGAGTTGTCTCATCAAATGGTAATACACCTTATTTATCCCCGTCTAACAGAATTGGAGATG
TTCAAGATTCCTCAAACTCACTCTTCAGCAGCCAGGATCCTTGGAATTTACGGCATGATATTCATTTCCCCCCTCCTAGACCTA
ACAAAATTACAATAAAAAATGAAGCCTTTAGTATTAGGGAACCATTTGGTGAAAATGGTACGAGCGATAGTGGGGATATAAAT
ACAGATGTGCAATTGGAGGATGGAGCCCACCAGCCATTTTCCAATTTGGATAAGGATTTCAATTCAGAGCATAGTTGGTCTGC
GAAAGGCTCAGGAGAGGAAGTGATCAAACAAGAACTTCAGGCGATTGCTGAGGGTGTTGCTGCTTCTGTTCTCCACTCGAC

TACATCTAATCCTGAAATTTCTATACACGAGAAAAATGAGCCTCTTTCTTTGTCCAATAAAGATATAGAGCTTCAAGATAGCGA
TTTGGAAATGCAGCATAAAAGTAAAGTTGAGGACAATATAAACAAAGTGCCAGAAAAAATTAATATGGGCTTCCCAGTGTCA
GATGGCATAGGTCGCTTGCAGATCATAAAAAACAGTGACCTTGAAGAGCTCCGAGAATTGGGTTCTGGCACCTTTGGTACTG
TTTATCATGGAAAATGGAGGGGCACTGACGTTGCAATCAAACGAATCAATGACAGGTGCTTTGCTGGGAAGCCTTCAGAAC
AAGAACGTATGAGAGATGACTTCTGGAATGAGGCAATCAAGCTTGCTGATTTGCATCATCCAAATGTGGTAGCTTTCTATGGT
GTTGTTCTTGATGGCCCTGGAGGCTCAGTTGCAACTGTTACAGAGTATATGGTTAATGGTTCTTTAAGAAATTCTCTGCAGAA
GAATGAAAAGAATCTTGATAAGCGTAAGCGTCTCTTGATTGCCATGGATGTAGCCTTTGGAATGGAGTACTTGCATGGTAAG
AATATAGTGCACTTCGACTTGAAAAGTGATAACTTACTTGTCAATCTTCGAGATCCTCACCGCCCAATATGCAAGGTTGGTGA
TTTGGGCTTATCAAAGGTGAAATGCCAGACACTGATCTCTGGTGGTGTGCGAGGGACACTTCCTTGGATGGCTCCAGAGCTA
CTGAATGGCAGCAGTAGCCTTGTGTCTGAGAAGGTTGATGTGTTTTCATTCGGTATTGTGATGTGGGAACTTCTTACTGGAGA
AGAACCATATGCAGATCTGCATTATGGGGCAATCATTGGTGGTATTGTGAGCAACACCTTGCGGCCATCTGTACCTGAGTTTT
GTGACCCAGAATGGAGAGCTCTGATGGAGAGATGTTGGTCTTCAGAACCATCAGAGAGGCCAAGCTTCACCGAAATTGCAA
ACCAGTTGCGATCTATGGCGGCTAAGATTCCTCCAAAAGGACAAATATCACAGCCCCAGGTTCAAAAATGA

>VviMAPKKK52    VIT_19s0085g00550

ATGGAACCTGGAAATGGGAAATTTTACCCTGAATCTCAGGGCTTTATGCTGGACCCAACAACTGCCATAAATACTGATACAA
GACCTCCAGAGTTTAACAATTTGGAAGTTAAACCTGTGCGTAATTACTCCATACAGACAGGTGAGGAGTTTGCCCTGGAATT
TATGCTTGATCGGGTGAATCCAAGGAATCAATTCATTCCAGATACTGCTGGTGATCCCCACTATGTACCAAAGTATACAGAATT
GAAAGGGATTTTAGGCATCAATCATACAGGGTCTGAAAGTGGGTCAGATATCTCAATGCTTACAATTGTAGAAAGAGGCCCA
AAAGAGTTTGAGAGAAAGAACTCTGCTTTATATGAAGACAGAAGTTACTATGGTTCTGTGCAATTGGTGCCACGGACTTCAT
CAGGGCATGATAGCAGTCGAGGAGTAATTCATGGGTATGCCTCTTCTGGAGCATCTGATAGCTCATCTACAAAGATGAAAGTT
CTATGCAGCTTTGGTGGTAAAATCCTTCCCCGGCCTAGTGATGGAAAACTCAGATATGTTGGAGGTGAAACACGTATTATTCG
TATAAGAAAGGACATTTCTTGGCAGGAGCTTGTGCAGAAAACGTTAATGGTCTTTAACCAAGCTCATATTATTAAGTATCAGC
TTCCTGGGGAGGACCTTGATGCCTTAGTTTCTGTTTCTTGTGACGAGGATTTGCAGAACATGATGGAGGAATGTAATGAACTA
GAAGATGGAGAAGGATCAAAGAAACTGAGGATGTTTCTGTTTTCTACAAGTGACTTGGATGATGCTTATTTTGGTCTGGACA
GCACAGATGGTGATTCAGAGATTCAGTATGTTGTTGCTGTCAATGGCATGGACATGGGATCGAGAAAAAACTCAACTCTGCA
TGGTTTGGTAGGCTCTTCCTCAAATAATTTGGCTGATTTAGATGGGCAAAATATTGAGAGGAATGCCACTAGAGTTGCAACGG
ACTCTGTTGGCATCAGCACTCTGCCTTTGACAGGCACTATCGTCCCACCATCCACAATTCAATCTTCTCAACCAATTCTACCA
AATTCTTCCAGTGCTTATGAAGCTGACCCACCGTTTTACCATGGCCAGATGATCTATCATGGAGAAACTAGTCAGCACATGCT
GCATTATGGTTATCCTTCTCATCAATCTAATTGCACACCATATCAAGAAAGTACTAATCTGATGCCAGTTCATGGGCTGATGAC
TCAACAAGAAGGCTATGCTGAAGGGCAGCCATACATTGGCTTGCAAGTTCAGGATCCAAGTGTGCTAGTGAAGGAGGTAAC
TCTAAAAAATGATGCTTCAATTCAGCAAGAGAATATTCCTGAAAATATCTCTCCTTCAAAAAATGATTGTCTCATACCATCACA
GCCATCTGATGGTGAGGTGATGGATCGCATTCCAGTTGAAGAAGCATTAGTCTCCATTTCTTCACTGGATCAATTTCCTTCAG
AAAATAAGGGAAAGCACCACAAACCTGTGGAGATCTCTTCTTCTGTTGATGCTATGAATCAGGCACAGGTTCCTAAATCTGA
TTATGATCATCATCCCGCATCAAGTAGTCCATTTGCTCCTGTATATGCTGACCCTGGGTCTGGTCTAATGGATTTGAGCTACCT
GGAGCCACCTGTGCTTCCTCAGAGGGTCTATTATTCTGAAAGAGTTCCCCGGGAGCAGGCAGAGCTGCTCAATCGGTTATCA
AAGTCTGACGATTCACTTGGTTCTCAGTTTCTTATATCCCACTCACGTTCTGATATTGAAAAGCAGGATTCAGTCGCCGAATC
CACTGACAAACTGCGGAATGGAAATCTGGCTCCTCAAACTGAACAGTCCATCTCAACTGGAGAAGCTATGGTTGAGGATAT
GGCTGTTAAGGCTGACCATGGAACAACCGGCACCAAAGATATCCCTAGGAAACTCCTGCTTCATGGAACAACTGAACCTGG
ATCAGAACTGCCTGCAATGAATCAAGTTGCTTCTGTTAAGCACTGGAGCTCTGTTGGTGTTTCCACGCCTGAGCAGGGAGAT
ATCCTTATTGATATCAATGACCGGTTCCCTCGTGATTTCCTTTCCGATATATTCTCCAAAGCTGTACATTTTGCAGATTCCCCTG
ACATTAGCAAACCACAAAAAGATGGAGCTGGCTTGAGCTTAAACATGGAAAATCGTGAACCTAAGCATTGGTCATACTTTCA
GAAGCTAGCACAGGGTGGATTTGTTCAAAATGATGTTTCTCTTATGGATCAAGATCATCTTGGATTTTCATCGGTCCTTACAA
AGGTCGAAGAAGAAGTGTCTAAACCTTATCAATTTACGCCTTTAATGGCAGATGAAGTTTTGATAGGCCAGTTGGAGTCACG
AATAAGTTTTGGTGAAGAAAATCAGAAAGAATCACCACCTGGTAGGATTGCTGCTGACAGTACTGATTTGCATTCAGATTAC

AGTCCTTCTGAAATAAAGGAGAGTGACAGTGTACAATTTGATCGTATGATCGAGAACCTTAGAACACCAGATTCAGAGGGCG
AGGATGGGAAAATGGAAACCAAGAATATTGGTCGACCTCCTCTAGATCCTTCTATTGGAGATTTTGATATCAATACTTTGCAG
ATCATAAAGAATGAAGATCTTGAAGAGCTGAAGGAACTGGGTTCTGGTACTTTTGGGACCGTCTATCATGGAAAATGGAGGG
GATCAGATGTTGCCATTAAGCGAATAAAGAAGATCTGCTTCACAAGTCGTTCATCGGAGCAAGAAAGATTGACCATAGAATT
CTGGCGAGAAGCTGATATTCTCTCAAAGCTTCACCATCCAAATGTGGTGGCATTTTACGGTGTAGTGCACGATGGACCGGGA
GCAACGTTAGCCACTGTCACTGAGTACATGGTTGATGGTTCTCTTAGGCATGTTCTACTTCGCAAAGATAGGTATCTTGATCG
CCGCAAGCGGCTCTTAATTGCTATGGATGCTGCATTTGGAATGGAATATTTGCACTCAAAGAATATTGTGCATTTTGATTTGAA
ATGTGACAACTTGCTTGTGAACTTGAAAGATCCCCTACGACCCATTTGCAAGGTTGGTGACTTTGGCCTGTCAAAAATAAAG
CGAAATACTTTGGTTTCTGGTGGCGTAAGGGGGACCTTACCATGGATGGCACCAGAGCTGCTTAATGGTAGCAGCAATAAAG
TTTCTGAAAAGGTTGATGTGTTCTCCTTTGGTATCGTGTTATGGGAGATTCTCACTGGCGAGGAACCATATGCCAACATGCAC
TATGGTGCAATCATAGGAGGCATTGTGAGCAACACATTGAGGCCAACCGTTCCAAGCTCCTGTGATCCCGAATGGAGAACCC
TAATGGAGCAGTGCTGGGCTCCTAACCCTGCAGTCCGGCCATCTTTCACGGAAATCACCGGTCGCTTAAGGGTGATGTCTGC
AGCAGCCCAAACTAAAACACCAGGTCATAAGGCTTCTAAATAA

>VviMAPKKK53    VIT_01s0011g01490

ATGGAGAGAAATCTCGGAAGAGAAATGGAGCAACAGAAAAACTACGAACAAGTTCGATACAACATTGTGGAAGCCAGAAA
TGAGGGACTTGGGTCTGCAAATCAAAGGTTTCTTCATGATCCATCGAGCACCATTAATACCAATATGAGACCTCCTGATTTCA
ATATAACAGTTGCAGCTAGGCCTGTGCTGAATTACTCCATTCAGACTGGTGAAGAGTTTGCTCTCGAGTTTATGAATCCCCGG
CAACACTTTGTTCCAAGTGCTTCTGGTGATCCTAACAGTGCAACTAACTATGCAGTTCTGAAGGGCTTTCTGGGAGCATCTCA
TACAGGGTCAGAAAGTGGGCCAGATATCCCAATGCTTACCTCTGTTGAAAAAAGTCGAGTCCAGGAGTTTGAGAGGAAGAG
CTCTTCTGTGCACGAAGATAAAGGTTACTATGATTCTGTAAGATCAGTGCCTCGAATCTCATCTAGAAATGACAGTAGTCGGG
GACTTCATGGCTATACCTCTTCAGGAGCATCTGAGAGATCATCGACAAAGTTCAAGTTCCTCTGCAGTTTTGGTGGTAAAATC
CTCCCCCGTCCAAGTGATGGAAAGCTTAGGTATGTAGGAGGTGAAACACGTATTATTCGCATGAACAAGGACATATCTTGGC
AGGATCTTATGCAGAAAACTATGACAATTTATAATCAATCTCATACGATCAAATATCAGCTTCCTGGTGAGGACCTTGATGCAT
TGGTTTCTGTATCTTGTGATGAGGATCTGCAAAACATGATGGAGGAGTGTAATGTACTAGAAGATGGGGGATCGCAGAAACT
TAGGCTTTTTCTGTTTTCTAGTAGTGATTTTGATGATGGTCAGTTTGGCTTGGGAAGCATGGAGGGTGACTCTGAGATTCAAT
ATGTGGTTGCTGTGAATGGAATGGACCTAGAATCAAGAAAGAACTCAATTGGGTTGGCAAGCACTTCAGATAACAATTTGGA
TGAGTTACTCAATCTAAATGTTGAAAGGGAGACTGGTCGAGTTGCAACAGAATTACCTGGACCTAGTACTGCACCTTCGACT
GTCAATGTACATTCATCAGCAGTCCAATCTTCTCAACCACTGGTGCCGAATTTTTCTGGTGCTTATGAATCCAATTCAAAGCC
TTACCAAGGTCAGAAAATGCGTCACGGAGAAGCTGAACAACATCAGGTAAAATCTGGTTCATATGCCTCTCCATGGAAAATG
AATGAACCTGAAAAGAATAGGTCACTAGAAAAGGAAGCGTCTGTGAAGGAGGCAAAAATAAAAACTGATAGCTCAGTCCA
AAAAATGAACGAGCTGGAAAAGATCCGGTCTTTAGAAAGTGAGCACAATGTTTCTTCACATCCACATGATGGTTCCGTTCCA
AATTATATTCCTAGGGACGAAGCATCAGTTGTCAATTCTACTGCAGACATAGGAGTGCCCATGTTGCTTCCAAAAACAAGTAA
AAAACACCTAGAATCTGTACAGATCTCAAAGCCCCCTGAAGCTGTAAGTGATGGGAAAATAAATACATTCAATGGAGATGGC
CATTTTCATACCTCTGGTGGAGCATTTTCTCCCGGATATGGTGACTCTGAGGCTGATCCAACTGAAGTTAGCTACCCTGAACA
AACACTGATTCCTCCACGGGTTTTTCATTCTGAACGTATTCCCAGGGAGCAGGCAGAATTGAACCGCTTGTCAAAGTCTGAT
GATTCCTTTGGTTCTCAGTTTCTGATGAGTCACACACGATCTGATGTTTCACAACAGGTTGCAGAATCAATTGATAAACTGCA
TGGTGGGAATGTGACTTCCCAGAGTGAGCAGGCCGCCTCATCTACAACAGCACTGTATACAAATCCTAAAACTGTTGAGGAT
GGACTGACACAATTTGAAAAGTATAAAGATGTTGCTGATGATATTAAAAAATTGAATTCCAATATTTCTGAAGATGGGTTGGG
GCCAAAGTTATTGAAATCTGAATCAAAATGGCCAGCACCAACTTCCGTGGATGATCATGAAATAGCCGGGGTTAGGGATGGT
AATAAAGACCCTGCTGTCAGTGATAGAGAAGCAGCTGGGTTGAATAATTTAACAGCAAGTCAAGGAACTTCAAGCAAACCC
CATGATGACTCTCCTTCTAAGCCAACAGGATTTCATTGGGATGAGATGGCTAACCCACTCAGATCTGTTCCTGGAGGAGAATC
CTCCGTTGGCGTTGGTGCTCCAGAGGGGGGAGACATCCTTATTGATATCAATGACCGCTTCCCTCGTGACTTCCTCTCTGATA
TATTCTCCAAGGCAAGAACCTCTGAGGGCCCCCCTGGAATCAGTCCACTGCATGGTGATGGAACTGGCTTGAGCTTGAATTT
GGAGAATCATGAACCTAAGCATTGGTCATTCTTTCAAAAGTTGGCTCAAGAGGAGTTTATTAGGAAAGGTGTTTCCCTTATG

GACCAGGATCATCTTGGGTATCCATCCTCACTTATGAATATTGAGGAAGGGACTCCTATAGATTACAGTTTTCCTCCTTTGAAG
AGTGATGGAGTTGCCTTGGGTCCTATGGATTCCCGTATCAATTTTGAGGAGGAAATTCAGCAAGAGTCTTCCAGCATGGTTAG
ACCTAACACCATTGATATGCATGAAGATTATGACCCTTCTCCGGTCAAGAGAGATGAAAGCGTGCAGATGGATGGAATGGCT
AACCCAAGAACGCCAGATTCGGATTATGAGGAGGTAAAATTCGAAATTCAGAACACAGGCGCACCTTTTGTTGATCCCTCTT
TGGGAGATATCGATATTAGCACCTTGCAGATCATAAAGAATGAAGATCTTGAAGAGTTGAGGGAATTGGGTTCTGGCACATTT
GGGACTGTTTATCATGGAAAATGGAGGGGAACAGACGTTGCTATTAAAAGAATAAAGAAGAGCTGTTTCACTGGTCGTTCAT
CTGAGCAAGAAAGATTGACTGTGGAGTTTTGGCGTGAAGCTGACATTCTGTCAAAGCTTCACCATCCCAATGTGGTGGCATT
TTATGGCGTGGTGCAGGATGGACCAGGGGGAACACTAGCCACTGTGACAGAATTTATGGTTAATGGTTCGCTTAGGCATGTT
TTGGTTTCCAAGGACAGGCATCTTGATCGTCGTAAGCGGCTCATTATTGCAATGGATGCGGCATTTGGAATGGAATACTTGCA
TTCAAAGAATATTGTGCATTTTGATCTGAAATGCGATAACTTGCTTGTGAACTTGAAAGATCCTCTACGACCCATTTGCAAGG
TTGGTGACTTTGGCTTGTCAAAAATTAAACGAAATACCTTGGTTACTGGTGGTGTGCGGGGAACCCTTCCATGGATGGCACC
AGAATTATTGAATGGTAGCAGTAGTAGGGTTTCTGAAAAGGTTGATGTTTTCTCCTTTGGTATTGTCCTATGGGAGATCCTCAC
TGGTGAGGAGCCTTATGCCCATATGCATTATGGAGCAATCATAGGAGGCATTGTGAACAACACATTGAGACCACCTGTCCCA
AGCTACTGTGATTCTGAGTGGAAATTGCTAATGGAGCAGTGTTGGGCCCCAGATCCCATTGGCCGCCCATCATTCACTGAAAT
TGCTAGACGATTACGGGCGATGTCTGCAGCATGCCAAACAAAACCCCAGGGTTATTCAGCAGGACCAGCACACAACCCAGT
CACCAAGTAA

>VviMAPKKK54    VIT_05s0051g00660

ATGACAGGTGAGACTTCTGGTTTTTCAGGCCAACACTTCTGTAATAACCCAGATAATGCTGTGTCAAGTGGTCAGTTGGCCG
CAGACAGAAATGCACATGATATCTGTGCACAGACAGGTGAGGAATTTTCTGCTGAGTTCCTTCGTGATCGTGTTGCTCCAAG
AAGGGCGTCTGCCATGATTGATACAGATCAGAGACAGCCAAAGGGATGGTGTAAAGGTTTTAATGAGAATCATCAGATGGTT
TATGAGCCCCTCAATGGCATTCTTGGGCTAAGGAGAGGGGATTCTGAATGCAGCTCTGACATCTTGGATTTTGTTCCTGGAAA
AGGATATGCAGCAGAAGTGGAGAATAGGGTTTATCTTGATAAAGCAAGCAGAATCCACAGGGAATATAGTGCCCCCAGACTA
GGATCAGGTCAGCTTTTTGAGGATTTCAATTGCGATCAAGCTGTCCCAGGACATGCTACTCCATCCTTTAATATACCAGAGTC
CCCTCAACCACATCATTGTCAAGGGTCAGGAGTTTCAGATGCTTCTTTCTCTGACAAGATGAAATTCCTTTGCAGCTTTGGG
GGGAGGATATTACCAAGGCCAAATGATGGGAAGCTTAGATATGTGGGTGGAGAGACAAAGATTATATCCATCAGGAAAAACC
TCTCTTGGGTTGAACTTGTGAAGAAAACTTCTGCTATTTGCAACCAACTTCATACAATCAAGTATCAGCTCCCAGGTGAGGAT
CTTGATGCACTCATATCTGTTTCTTCAGATGAAGATCTTCATCATATGATCGAGGAGTATCATGAGCTGGAAAGAATTGAGGGT
TCTCAAAGACTAAGGATATTTCTTGTACCGGTGGGTGAACCTGAGAGCCCTAGTTCTTTCGAAACAAGGGCCACACAACAG
AATGAAGCTGATTATCAGTATGTTGTTGCTGTGAATGGCATGCTTGACCCAAGTCCACGGAAGAACTCTAGTGGGCAGAGTG
TATCAAGCCAGACGGGAAATACCTGTGATTATAGAGACCCTCCTTTTTTTCATCCTTTGGAGATGAAGGATGGAGCTAGTTCC
TCAAATTTGGTGGGGATGTTCACAAATCCTGCTGCTCAGTTTTTGACTTCACTCCAGATACCAACCAAGTCATTTCAGCAATC
TCCTCCTGTATCTCCATTGCCAGTTCAGAATAGAGATCCTCAGAATTCTGCAATGCATTTCTTCGAGGATCATGCATACCATGA
TGGTCATGAAAGCGCCAGTCAATTTGTCACAGATCAATGGCCATGTGACAACGCCTATTGTGTTGATTCTCCCAGCTATTATC
ATAATAATCCGTATGGGCCTGTGCCATTGATGAATTACCATCATCATAACAAACATTTTCTAGAAACTGACCAAATAAACAAAC
TTCCTAGTTTGCATGTCCAGAACCGTCCCAGTAGAGATTTTGTTTTTTCTCCTGTACTTGGTCAAAGTGAAGTGGATTTTGAG
AGGCCTGTGCTTAAGGAGAGAGCATTCCACTCTCATCCCAAAGATCCACTGGGTCTTCTGTCAGGATCAACTAATGATTTGGT
AGGTTCTCATCACCGGATGTTGCATGTGCTTTCTGATTCACAGTTGCGGGGACATGAAGGGAGGCCTGATTACCATTTGGAG
GAAGGAATTATTCCATTGTCCCCATGGACTTTTGAAGTTCAAAAATCACCTTCGTTGGCTTTGTCTAATTCTCCGCAAGAATG
GTCTTTTCAACCGCAAGAAATAAGCAATGAGAAATACCAAGAAGCTTATCAGAATCAGCCCACTCTCATAGTGGATGACCAC
AAGGGAAATAATGGACTGGGTCAAGATACATGGAACTGGGAGGATGAGATAGATACTCAGGTAGGTCAGGAAAGGAAACAT
GATAAAGTAATCACTGACCTTACATCACAAGATAATTCCACACTACCTAATACAAAGTTGCAAAATGTTTGTTACAACCCAAA
TTCAGTACCCAGTATTCATATTTCCCCACTTGAGTTTCAAGACCATGGAGACACAACTATGAATTCTGCTTCAACACTCATGAT
TCCAGAAAATTCTGCTGATATTGTGAGAGAGCAGCCTCATGATTACTCATTGGGTGCAAGTACACCCAAGTTTCTCGTCAAA
AGCCAAAATGCTACCAAGGACATGCAGCATGCTATGACTGAGGTAATAAGTAGTGAATCAGTTCCCAATGAATCTTCTCGAC

CTCTATCTGTTGCAATTCAAGGAACAGGTGACCAAGAAGCAGCAGTCCCAAGCTCTGCAAGTTTAACTCCATCTGCAGGAA
ATAAATCTGATCCAAGCTTAAACTTGCAGAAGAATTACCCACTCAGTACAGAGTCTTCATTTGAAAATCCTGACAAGAAAGC
TGTTATGAGTGGAGTATCTACTCTTAAAGATGAAGACCCTCTGAATTTCCCTTGTCATGAGGTTGATGGCCCAGAGGGACACT
TTTATGAGAGATTAAATCCTGGAGATGCTATCTTTGTCCAATCACAGCCCTCAGATAATCATCATAACGGCAACACACCAGGT
GCTGCAGTTATTGTTGAAGATGTAACTGATATTTTGCCTCCTGGCATTCCCTCATCTTCCCCACTTATCCCACAAGTAGAAGAT
GAAGCGAGTGATGTAATTACATCATCTGGAGAAGCAGAGGCAGAGAGTGATATTCAAGAATCTGAAGGGGAGGAGGGCAG
AGATCTGGGTGAATCTATTAGCGATGCTGCTATGGCTGAAATGGAGGCAAGCATCTATGGTTTGCAGATAATAAAGAATGCTG
ATCTTGAAGAGCTGAAAGAGTTGGGATCTGGTACATTTGGAACTGTTTATCATGGAAAGTGGAGGGGAACAGATGTTGCAAT
TAAGAGAATTAAAAAGAGCTGCTTTGCAGGGAGATCATCTGAGCAAGAGCGGTTGACTAAAGACTTCTGGAGAGAGGCTCG
GATCCTATCAAATCTTCACCATCCAAATGTGGTAGCATTTTATGGGGTGGTTCCTGATGGGCCTGGAGGAACATTGGCAACTG
TAACTGAATATATGGTAAATGGATCATTGAGGCATGTCCTACTAAGGAAGGATAGATCTCTTGATCGCCGGAAAAGGCTTATA
ATTGCAATGGATGCAGCTTTTGGCATGGAATACTTGCATTTGAAAAACATTGTTCACTTTGATTTGAAATGCGACAATTTGCT
CGTCAATATGAGGGACACTCAACGGCCAATATGCAAGGTTGGAGATTTTGGATTATCGAGAATTAAACGCAATACACTTGTAT
CGGGTGGTGTTCGAGGAACCCTTCCATGGATGGCACCAGAATTATTGAATGGTAGCAGTAACCGGGTTTCGGAGAAGGTTG
ACGTTTTCTCATTTGGCGTTGCAATGTGGGAGATCCTAACTGGAGAAGAGCCCTATGCAAACATGCACTGTGGTGCTATCATA
GGGGGAATTGTGAGTAACACACTTAGGCCTCCTATTCCGGAGCGATGTGATCCTGACTGGAGAAAGTTAATGGAAGAGTGCT
GGTCACCTGATCCGGCAGCTAGGCCATCATTCACTGAGATAACAAACAGGCTGCGGGTTATGTCCATGGCGATCCAGACAAA
GCGGCATAACCAGGCAAACAGATGA

>VviMAPKKK55    VIT_18s0001g07700

ATGCCTCACAGAACCACTTACTTCTTCCCCAGGCAGTTTCCCGACCGCAGATTCGATGCATCTTCCAAGGAGCTGCTGGCTC
ACGAGAAGAAGATTGGAGGCGAAAGTAACAGAAAAGGTACGAGAACAACCAAGGATGTGACTGCTGATAGGACTTACAAT
GCTTCTGATCTATTCACGGGAAGCGATAAGTTTCGGAGCAAGAAGCAACTCGCCGCTTTTTGTGACTGGTTGGTGGAGAAA
AAGGGTGACAGATCGGGTCACGTGAGGTTGAGGTCGCGGAACGATGAAGGTGATCGCGACGTGTTGTTGCCTCCACCGCC
AGCGCCGGTGCCGGAGGTGGTGGCAGGAAAGGACCAGCAGTTTGATCGGCAGGTGTCGTTGCCGAGGGTGTCGAGCGGTA
GTAGCTATGCTGGGAGCCTGTTCTCTGGAACGACGGTGGAAGGGAACGTGTCGAGCGGTCTGAAGGATTCGCATACGAATT
CGCATTCGCAGGAGTCGACGAGGCGAGAGGTGGATGAGGAGAAGGAGAGTGCGGCGCAGAAGTCAAGGGAGAGCTACTA
TTTGCAGCTCACGCTTGCTAAAAGACTCGCTTCTCAAGCGTCGTTGGCTTGTGAGCCTGTGCTCTTTTTACAAGAGAGTGGA
GCGGAGGGAAATGCCGTCTCCTTCGATCCCGACGTTGTTTCCTATCGTCTTTGGGTTAGTGGGTGCTTGTCATATACTGACAA
AATATCAGACGGGTTCTATAACATTCTGGGAATGAATCCGTATGTGTGGGTGATGTGCAATGAATTGGAGGAAGGTAGAAGG
CTGCCACCTTTAATGGCGCTTAAAGCCGTAGAACCCAATGATACATCCATGGAGGTGGTTCTTGTTGATAGACGTGGAGACT
CGCGCCTCAAGGAGCTTGAAGATAAAGCACATCAATTATACTGCGCTTCAGAGAATACCTTGGTGTTGGTGGAGCAACTTGG
CAAGCTGGTTGCAATCTATATGGGGGGTAGTTTTCCGGTGGAGCAAGGTGATCTCCATAAGCAATGGAAATTGGTTAGCAAG
AGATTGAGAGATTTTCAGAAGTGTATTGTGCTACCGATCGGCAGCCTATCAATGGGGCTCTGTAGGCATAGAGCAATTCTTTT
CAAGAAATTGGCGGACTACATAGGTTTGCCTTGCCGGATAGCTCGAGGTTGCAAGTATTGTGTTGCCGATCATCGCTCTTCAT
GTCTTGTGAAAATTGATGACAAGCAGTCCTCAAGGGAATATGTAGTTGACCTAGTTGGGGAACCAGGAAATGTCCATGGTCC
AGATTCCTCAATCACTGGAGGTTTACTTTCATCAATGCCTTCACCACTTCAAATTTCTCATCTTAAAGAATATCAACAGCCTTA
CATGGATAATGAATCATGTTGTCAAATTCAAAACTCAAAGAACACATGTATTTATCCCGAAGATCCTCTATATTTAGGCAATGA
GAAAAACACCTTGTACACTCCAACTGATCAAATTTGTGAAAGAATGGAATCATCTGTGTTGCCTTTGGAGTTCAATGGGAAC
ACTGATCGGTGCATAATTCAAAGTGCAATGTTGCAATCTGTCCAAAGCAATGTTTCTGAAGCTGTGGATGCTTCTGCTTCTGG
GGTGTCTATACATGAATGTTTTAGAATTGCTGGAGAAAAGATTGTTATACAACAAGCTCACAAAAAAGAGATTGCTCTATCTG
GAAGTCCGATTACGAGTAAGGCTCTCAAGCAGCCTAAAGTAAGCTTGTCTAGTAAATCAAATATCAAGGAGGTTGAAGGCA
GACTTGAGAATCGAGGTAGATTCCATACTGTAACCATTCCAAGATACTTGAATCTTGAACCTTCTCTTGCAATGGACTGGCTA
GAGATTTCATGGGATGAATTACATATCAAAGAGCGTGTTGGTGCTGGTTCATTTGGAACAGTGCATCGTGCTGAATGGCATGG
ATCGGATGTTGCTGTTAAAGTTCTAACTGTCCAGAATTTTCAGGATGATCAGTTGAAGGAGTTCCTAAGAGAGGTTGCAATAA

TGAAACGGGTCCGCCATCCGAATGTGGTACTCTTCATGGGTGCAGTAACAAAGCGTCCACATCTTTCAATTGTGACTGAATAT
CTGCCTAGGGGTAGTTTATATCGTCTAATTCATAGACCAACTTCAGCGGAAATTTTGGATCAAAGGAGGAGATTACGAATGGC
CTTGGATGTGGCCAAGGGAATCAATTATCTACATTGCCTTAAACCTCCTATAGTTCATTGGGATCTTAAATCTCCCAATCTATTG
GTGGATAAAAATTGGACAGTGAAGGTATGTGATTTTGGGTTATCCAGGTTCAAGGCAAACACTTTTCTCTCATCAAAATCTGT
TGCTGGGACACCTGAGTGGATGGCCCCCGAGTTCCTACGTGGAGAGCCTTCAAATGAGAAGTCTGATGTTTACAGTTTCGG
AGTGATCTTATGGGAGCTTGTGACCATGCAACAGCCATGGAATGGACTTAGCCCTGCCCAGGTGGTTGGAGCTGTGGCTTTC
CAAAACAGAAGGCTTTCTATCCCACAGAATACCTCTCCAGTGTTGGCTTCTCTTATGGAATCTTGTTGGGCTGATGACCCTGC
TCAGCGCCCCTCTTTCTCTAGTATAGTAGAGACACTGAAGAAGCTGCTAAAGTCTCCACTGCAGTTAATACAGATGGGGGGT
ACATAA

>VviMAPKKK56    VIT_13s0074g00430

ATGGAACCTCATAATAGGCATGTACAGGAACAGATTCACTCCACCTCCAACCGCCAGCTCGCTTATGGAAGGGAACACTGGT
CCAGCCCCAAAGACACGACGTGCTCCAAGAGCTGGGCTCAGCAGACCGAGGAGAGCTACCAATTGCAGCTCGCTCTCGCA
CTTCGCCTCTCCTCCGATTCTTCTTCTGCCGCCGATCCTTATTTCTTGGATTCTGCTACCGGTGATCGCCCAATTGGGTCTGCC
AGGGACCTCTCTCATCGGTTCTGGGTCAATGGCTGCCTGTCTTACATTGATAGGGTTCCAGATGGATTTTACCTGATACATGG
GATGGACCCATATGTATGGACTATAAGCACTGATTTGAAAGAGACTGGTCGGATCCCATCTTTTGAATCATTGAAAGCTGTTG
ATCCTCGTGATGATTTCTCAATTGAAGTGGTTTTGGTTGATAAACATCGGGATCCTTCTTTAAAGGAGCTACAGAATAGGGCA
CTAAGTCATTCTAGTAGTTGGATTAAGGCAAAACAAGTGGTTGATGAGCTTGCAAACCTTGTCTGTAATCACATGGGGGGTG
CAGCTTCTTCTGGAGAAGATGGTTTTGCCAACCACTGGAAGGAGTTTTCTGGCATGTTGAAGAACAGCCTTGGCTCTGTTGT
ACTTCCAATTGGAAGCTTATCTGTTGGCCTGTGTGTTCATCGTGCACTTCTATTTAAAGTGCTAGCAGATGTAGTCAACTTACC
ATGCCGGATTGCAAAGGGTTGCAAATATTGTAGAAGCAATGTTGCTTCCTCCTGCCTTGTGCGATTTGGCCCTGAAAGGGAG
TATTTGGTTGATTTGATGTGTAATCCGGGTGCTCTGTGCTCACCTGATTCCTTGCTCAATGGTACATCTTCCATCTTAGTTTCTT
CGCCCTTATGCCATCCAAGATTTAAATTGGTTGAAACGGCTGAGGACTTCAGGATATTGGCCAGACTGTATTTCTTTGACTGT
CAATCACTTAATATTGCATTTGATGATCCTTCCTCAGGTGCTGCTGTTGGACAAGATGATAACTCAGATTCACGGTTCCCTAAA
CCTTTTGATAGGAGTTACACTGAGAGCAAGAACCTGGTATCAACTTCAAATAACCACCATGAACTTTTTCTGCCCCAAAGGA
CTGCACGACTTGTATCCCATGATAGAGATCCCCAGATGCAAAACTCGTTCAATCCTTTGCCAAATGTCATAAACTCAAAACAC
TTGGTCAAAGGTGCAGTTCGACCAAGTCACATCCTACCAATGGGTAATAGAGATGTTCAACCTATTTTGCCCTTTCCTAGGCC
AAGGCCTGGTACAAATAAGAATCTGGGGTTCATGGAAAAGAATCACTCAGTAACAAGTAGATCAAGTCTAAAATATTCTCTT
GTAGAGGAGGATCTGGAGATTCCGTGGAGTGAACTTGTTTTGAAGGAGAACATTGGAGCAGGTTCGTTTGGAACTGTTCAT
CGTGCTAAGTGGCGTGATTCAGATGTTGCTGTCAAGATTCTCATGGAGCAAGATTTCCATGCAGAGCGCTTCGAGGAATTTCT
AAGAGAGGTTGCAATCATGAAACGCTTGAGGCATCCAAACATTGTTCTCTTTATGGGTGCTGTTACTCAGCCTCCACATCTGT
CCATAGTTACAGAATACTTATCAAGAGGCAGCTTATATAAACTCTTGCGCATGCCTGATGCAGGCATGGTACTGGATGAGAGG
CGTCGTTTAAATATGGCTTATGATGTGGCAATGGGGATGAATTATCTTCATCAACTCAAACCTCCTATTGTTCATCGAGATTTG
AAGTCTCCAAATCTTTTGGTGGATGGTAATTATACGGTAAAGGTTTGTGATTTTGGTCTTTCCCGTTCAAAGGCAAACACATT
TCTTTCATCTAAAACGGCCGCAGGGACACCTGAGTGGATGGCACCAGAAGTTCTCCGTGATGAGCCATCAAATGAAAAGTC
AGATGTTTACAGCTTTGGTGTAATCTTGTGGGAACTTGTGACCTTGCAAAGGCCTTGGAAACATTTAAATCCAGCTCAGGTT
GTAGCAGCTGTTGCTTTCAAGGGAAAAAGGCTTGAGATTCCAGCTGAAGTAAATCATCAAGTCGCCTACTTGATTGAGGCCT
GCTGGGCTAATGAGCCCTCGAAGCGTCCTCCTTTCTCCTTTATCAAGGAATACTTGCAGCCACTGATCTCATCCTCACCTCAA
CCATTTCAAGGGTCACATTCCAGAAATTACCCGAAGGGTGGTTAA

>VviMAPKKK57    VIT_08s0007g03910

ATGCCTGGTAAAAGATCGAACTACTCTCTCCTCAGTCAGTTTCCCGACGACCAGTTCGTCGGCGGAGCGGCCGGAAATCAG
CCTCCCTTATACGAGTCCCTTTCTGGGGAGAAGAGCAAAGGGAAAGGCTTTGATTGGGATGGTGGTGATCTTAGGAACCGGA
TCGGGAACTTGTTCACTACGTCGATCGGGTTGCAGCGGCAGTCCAGCGGCAGTAGCTTCGGCGAAAGCACTCTGTCGGGGG
AGTATTACGTGCCGACGATGTCGATGGCGGCGTCGAGCGACTTCGATGCGTTTGGGGATGTGTTTAAGGTTGGTGGTGGCGG
AGGCGCAGAATTGAGGGCCAAGGCAGTGACCGGGACTGGGGACTCGTCGTCGTCCAAGAGTTGGGCACAGCAGACGGAG

GAGAGTTACCAATTGCAGTTGGCGTTGGCGCTTCGGCTTTCGTCGGAGGCCACCTGTGCTGATGATCCCAATTTCTTGGATCC
AGTGCCAGACGACTCGGCGTCCAGGTCGTTATCGTCGTCCGGGAGTTCGGTCGAGGCTATGTCTCATCGATTCTGGGTGAGT
GGCTGCCTATCATATTTCGACAAAGTTCCTGATGGATTTTACCTAATTCACGGGATGGATCCATATGTGTGGACTGTATGCAAT
GATTTGAGAGAAAATGGTCGTATTCCTTCCATTGAATCACTAAAGCATGCTGAGCCTAGTGCTGATTCTCCAATTGAAGTTGT
TTTGATTGATCGACGTACTGATCCCACTTTGAAGGAATTACAAAATAAGGTTCATGGTATTTCTTGTAGTTGCATGACCACAA
AAGAGGTGGTTGATCAGCTTGCAAAGCTTGTCTGCAATTGCATGGGGGGTGCAGCTTCCACTGGAGAAGATGACTTTGTTTC
CATCTGGCGGGAGTGCAGTGATGATCAAAAGGATTGTTTAGGATCTATTGTGGTTCCGATTGGTAGCCTGTCCTTTGGTCTCT
GCAGACATCGTGCTTTACTGTTCAAAGTGCTGGCTGACACAATAGATTTGCGATGTCGGATAGCCAAGGGCTGCAAATATTGT
ACAAGAGATGATGCTTCCTCCTGTCTTGTTCGGGTTGGGCCTGACAGGGAGTTTCTAGTTGATTTGGTTGGCAAGCCAGGGT
GCTTATGCGAGCCTGATTCTTTGCTCAATGGTCCAGCTTCCATCTCAATTTCTTCCCCATTGCGCTTTCCACGTTCCAAACCAG
TTGAAACTAACATTGATTTCAGGTCACTGGCTAAGCAGTATTTCTCAGAATGCCAATCCCTTAATCTTGTTTTTGAAGACACT
TCAGTGGGTGTCATTGTTGATGAAGCTGATGGAGGAGATTCCATGTATCCTAAAAAATTTGATAGGAAGTGCACAGATAGAA
CCCACCTTGTGCCCATTTCAAGAAACAGAGGTGAAACTCCACAGTTGCCTATGCCCCCAAAAGTTGCTTGGCCAAGTGCCC
ATGATCAAGATTCCCAACTGTTTAAATCATGCAACCCTTATCAGAGCAGTATAAGTCCAACAGATGCAGTCAAAGATCCTATC
CCACCAAAGCGTATCCCACTAACTGGGCATGGGGATGTTCAACCATCTTTAGCCTTGTCTGATTTGAGAGGGGATACAATAA
AAGATATGAGGTTCACTGATGGAGGCCAATTATATCCAAATAAACCATGCAAGGAACTTTCCCTTGATGTGGAGGATTTGGAT
ATTCCATGGAGTGACCTTGTTTTAAAAGAGAGAATTGGAGCAGGTTCTTTTGGGACTGTTCATCGTGCTGATTGGAATGGATC
GGATGTTGCTGTAAAGGTTCTAATGGAACAAGATTTCCATGCAGAACGTTTCAAGGAATTTTTGAGGGAGGTTTCTATAATGA
AACGCCTGCGGCATCCGAATATCGTTCTTTTTATGGGTGCAGTTACGCAGCCCCCAAATTTGTCCATAGTTACAGAATATTTAT
CAAGAGGTAGCTTATATAGGCTTTTGCACAAACCTGGTGCAAGAGAAATGTTAGATGAGAGGCGTCGATTGAGTATGGCTTA
TGATGTGGCCAAGGGCATGAATTATCTTCATAAACGCAATCCTCCCATTGTTCATCGAGATTTGAAGTCTCCAAACCTTTTGG
TTGACAAAAAATACACCGTGAAGGTTTGTGATTTTGGTCTTTCACGGTTCAAGGCAAATACATTTCTTTCATCCAAATCGGCG
GCAGGGACGCCTGAGTGGATGGCACCAGAAGTTCTTCGGGATGAGGCATCAAATGAAAAGTCAGACATATATAGCTTTGGTA
TAATCTTGTGGGAACTTGCAACATTGCAACAACCTTGGAGTAATTTAAATCCAGCACAGGTTGTAGCAGCTGTTGGTTTTAA
GGGCAAAAGGCTTGAGATCCCACGTGATTTAAATCCTCAAGTGGCTTCCATAATTGAAGCATGTTGGGCAAATGAGCCTTGG
AAGCGCCCTTCCTTTTTCAACATCATGGAATCCTTGAAGCCACTGATTAAACCTCCCACGCCTCAGCCAGTTCGTGCAGATA
GACCACTGCTTACCTGA
>VviMAPKKK58    VIT_17s0000g02540
ATGAAGAACATTCTGAAGAAGCTCCATATTGTGTCGAATCAAACCGAAGACGTTGAAGGGTCTACTTCATCAAGGGGAAGC
AAGACTCATGATGGGTCATCCCCAGATAGGCTTTTGCATTCTCGGCCCCATCATAATTCCGAGCATAAACCCTTTTCGGGTCTT
TCGAATTGGTTGAATTCAGTTGCTAATAGACATAGCCCCAGTCCCCCATTATCTTCAAATGTCACAAGAGTAGAGAGATCAGA
ACCATCTGATTCCATGAGCAGTTGCGGTTTGGACGTCGTTTCAGATGCAGTGAGGCGTGACTCAGGGTCTAGCAATTCAAGG
GATCCGGATATAGAGGAGGAGTATCAGATACAATTGGCCTTGGAGTTGAGTGCGAGGGAGGATCCTGAAGCAGTTCAGATTG
AAGCCGTTAAGCAGATCAGTTTGGGCTCTTGTGCTCCAGAAAATACTCCAGCAGAAATTGTTGCTTATCGATACTGGAATTAC
AATGCTCTTAGCTATGATGACAAGATCTTGGATGGTTTTTACGACCTGTATGGAATTTTGATGGAGTCCACCTCACAAAAGAT
GCCTTCCCTTGTTGATCTACAAGGAACACCATTATCAGATTGTGTCACCTGGGAAGCAGTTTTGGTCAATAGAGCTGCTGATG
CTAACCTGTTAAAACTTGAACAGGAGGCCCTGGTCATGGCCGTCAAGTCAAGGTCGGAATCTCCAGTTTTTGTAGGTAGTGA
TTTGGTGCAAAGGCTTGCTGCTTTAGTTGCTGCGAATATGGGTGGACCAGTTGGGGATCCAGTCAACATGTCAAGAGCGTGG
CAAAGTCTCAGTTACAGTTTGAAAGCAACCCTTGGGAGCATGGTTTTGCCTCTTGGTTCTCTGACAATTGGATTGGCTCGCC
ATCGGGCATTGTTGTTCAAGGTTTTGGCTGATAGTGTGGGCATCCCATGCCGATTAGTGAAAGGACAGCAATATACAGGTTCT
GATGATGTGGCAATGAACTTCGTGAAGATTGAAGATGGAAGGGAGTACATTGTTGATCTAATGGCAGATCCTGGAACACTTA
TTCCATCTGATGCAGCAGGATCGCATATAGAATATGATGATTCTATCTTTTCGGCCAGTACTTTGTCTAGAGAGATAGACTCCT
CATATATAGCCTCTTCTAGCAGTGGGGTTGTGAGGCCATATCTGTCAGCTGTTGGAAATGAATCTGATGACAGAGGGGAACTT
ACGGCTTGTGCAAATCTACCCAGGCCAAGTAAAGATAGTTTGAATGCAGAGCAGACTCTGCTTCGGGCACTTCCCAGCAGG

CCTAGCCATCCGTACATGCATGGAAGATCTCCCTCCTGGACAGAAGGTGTAAGCTCTCCAGCGGTGCGTAGAATGAAAGTGA
AGGATGTTTCACAATATATGATTGATGCTGCCAAAGAAAATCCACAGTTGGCCCAGAAACTTCATGATGTGTTACTTGAAAGT
GGTGTTGTTGCTCCTCCAAACCTATTCACTGAAATTTATCCCGAGCATATAGATGTGTCAATAGTTGAGGCCAAGTCCCCAAC
TGAAGATAAAGATGAAAATGAAAAGAGACCAGTAATCCGGAAAATTAAAGATCAAGATGACCTTGGTCCGATTGGCTTTTTG
CCACCTCTGCCTTATCATGGGATGCAACCTAGAGTAAGTCCTTGTGTACAGCCAGATCTTAAGCCTGTGGAAGGTTTAGGGTT
TAACAATCTGCTAGATTTTAAAGAAGTAACTGGGCAGTCTGTTTCATCACAATCAGAGGTGAATCCTGTGAAATATGTGAAG
AATGTTCCTGTTGCTGCTGCTGCAGCGGCAGCAGCAGTTGTTGCATCTTCAATGGTTGTTGCTGCAGCAAAGTCAACTGCTG
ACCCAAACCTTGAACTTCCTGTGGCGGCTGCTGCCACTGCTGCTGCAGCAGTCGTAGCAACAACTGCAGCTGTTGGTAAGC
AGTATGAGAACTTGGAAACTGGTGTTCATTCTCCAAGTGGTGCTGCTGAGTGCTTTAACCAAACGGATGGCATGCAAAGTG
GGGGAGATGCAGATGGTGCTGGTTATGAGCCTCATGGTAGTGGTAACCGGGAGCATGATGCTTCTGGGACAAATCCTGAAGG
AGAGAGAACATCGGATAGATCTGCGGATAGCACAAAATCAGATGTGGCACTTGATGATGTTGCAGACTGTGAGATCCCATGG
GATGAAATCGCCTTGGGTGAGCGTATCGGACTTGGATCGTATGGGGAGGTATATCGTGGAGACTGGCATGGCACTGAAGTTG
CTGTTAAGAAGTTCCTGGACCAAGATATTTCTGGTGAATCACTTGACGAATTCAGAAGTGAGGTGCGGATCATGAAAAGACT
AAGGCATCCCAATGTTGTTCTCTTCATGGGAGCGGTAACTCGTGTCCCAAATCTTTCAATTGTTACAGAATTTCTTCCCAGAG
GTAGTTTGTATAGACTAATTCACCGGCCTAACAATCAATTAGATGAAAGGAGGCGTTTGAGGATGGCCCTTGATGCTGCTCGG
GGAATGAATTATTTGCATAATTGCACTCCAGTAATAGTTCATCGTGATTTGAAGTCTCCAAACCTTCTTGTCGATAAGAATTGG
GTTGTGAAGGTATGCGACTTTGGCTTATCACGAATGAAGCATAGTACTTTTCTTTCTTCCAGGTCAACTGCAGGGACGGCTG
AGTGGATGGCTCCGGAAGTACTAAGAAATGAGCCTTCAGATGAAAAGTGTGATGTTTTCAGCTTTGGGGTCATATTATGGGA
ACTCTCTACATTACAACAACCATGGGGAGGAATGAACCCAATGCAAGTTGTTGGTGCTGTTGGATTCCAGCATCGCCGTCTT
GACATTCCAGATGATATGGATCCTGTTGTGGCAGATATTATTAGGAGATGCTGGCACACAAATCCAAAAATGAGGCCTACATT
TGCTGAAATCATGGCTACTCTGAAACCATTGCAAAAGCCTATAACCAGTTCACAAGTGCCTAGACCTAGTGCAGCAATAAGC
AGTGGTCAGGAGAGGGTTCAACCATCACGAGCTGCAGAAGAACCAGCAGAATAA

>VviMAPKKK59    VIT_04s0008g01310

ATGTCTCGAATGAAACATCTGCTCCGAAAACTCCATATCGGAGGAAGCCTCAATGAGCACCAACGGATACCCGAGACCCGA
CCCGTGATCAATCCGAGTCCGAGTCCGAATCAGTCATCCCCCGTGGCCGCGGCGGCTCCATCGTCGGCATTGGGGAGCGTGG
GAGGCGGTGATGCGGTGGATCGGGCGGCGGTGGATTCACAAGATGCTGCAGTCGATTTCAGTTTCCTGGAGGAGGAGTTTC
AGGTACAGCTGGCTCTGGCCATATCCGCCTCGGATCCCGATGCGCGAGATGACCGAGAGACGGCTCAGATCAAAGTAGCCA
AGCGGATTAGCTTGGGGTGTTCGCCGTCCACGACCGACACTGAAACCCTAGTTGAGTTGCTCTCGCTTCGATACTGGAACTA
TAATGCTGTAAACTACGATGAAAAAGTGATGGATGGGTTTTATGATGTATATGGTATTACTGCAAATTCAGTTGTGCAAGGAA
AGATGCCATTGCTCGTTGATCTACAAGCAATTTCTGTTCTGGATAATGTTGATTATGAAGTGATTTTGGTAGACCGCATGATTG
ATCCTGATCTGCGAGAACTTGAGGATAAAGCTTATTCTTTGTCCATGGAGTACCAAGTTTCTGATCAGTTGACTATTTTAGATG
GGTTGGTCCAGAAAATTGCAGATATGGTTGTTGAGAGAATGGGTGGTCCTGTTGGTGATGCTGATGAAATGTTGAAAAGATG
GACTATCAGAAGTTATGAGTTGCGGAGTTCTCTGAACACTATCATCCTTCCCCTTGGACGGCTGGATATTGGGCTTTCACGCC
ATAGGGCCTTGCTCTTTAAGGTGCTAGCTGATAGGATTAATCTTCCATGTCTGCTGGTTAAAGGGAGCTACTACACGGGTACT
GATGACGGGGCCATAAACCTGATTAAAATTGATAATGGAAGTGAATATATAATTGATCTAATGGGTGCCCCGGGTGCTCTAATT
CCTGCTGAGGTACCCAGTAGTCATCATCAAAATTTTGGACTAGATGTAAGGAGCTGTACAGATGTTATAGAAGCTGCCCGAG
AATCACTTCTAGTGCCTGAAAAAGGAACTGGATTTTCACCTAATCTTGATGTAGTTTCCAAGCCTGGCAGTTCAAAGTCAGA
AGAAGCACCATTTATAGGCATTCGATCGAAAGGGGATGACAGGAGTCCTGTTGAAAAATTTGAAACTGAGAGATTTGAAAA
TGAATTTGGGAACCTTCTTCCTTCACTACGTAAATTATGTGAAGGCTCATCGGGAACCTGTGGGAAAGCATCACCTGCACAA
AAGATGAAAGTTAAAGATGTTTCCAAATATGTCATCAGTGCAGCAAAAAACCCAGAATTCGCACAGAAACTACATGCTGTTT
TATTAGAGAGTGGTGCATCGCCTCCACCAGATTTGTTTTCGGATATAAATTCCCGGGGTCAGGTAGAGCAGAAAGTCCTTGA
GCAAATCCATATGGCAAAAGGGAAACAAGTGGATCATGGGGTTTGGTATTCTCCTGGTGAGTTTTTGTTGAACAGTGAGCAA
CCTCTCATGCCCTCTCACCAGGTGGAAACAAATGTCACCAACTCAGACTTCTCTTTGCCTTCTGATACTACAAGTGAGGGAT
TTATACTTATTGGTGCTGGAGCTAATGGAATGATCCGCACTAATGCTACAGGAGAAACATGCCAAAGACAACCTGAAAATGC

CTTAGTTAGTGATGGTGGACCCTGCTTTCAGGATAATATAGGGAGAATTCTTAGTAACATTGGAACAGAGAAGGAATCTGCTT
TGGGATTAATGGAAACAGCCAACGGTGCCTTGCATATTCCTTCCAATGCTCACAGTGAGCAGATCAACCCAATGCTGGCTGA
GGTTGCGGAATGGGAAATCCCATGGGAGGATCTTCAGATTGGTGAACGTATTGGTATTGGCTCATATGGTGAGGTTTACCGAG
CAGATTGGAATGGCACTGAGGTTGCTGTGAAGAAGTTCCTAGCCCAAGATTTCTCTGGTGATGCATTGGTTCAGTTTAGATAC
GAGGTTGAAATCATGTTGAGATTGAGGCATCCTAATGTTGTTCTTTTTATGGGAGCTGTTACTCGCCCCCCAAATCTCTCTATA
CTCACAGAGTTTCTCCCAAGGGGAAGTTTGTATAGGTTATTGCACAGATCAAATATTCAACTTGATGAAAAGAGGAGATTGC
GAATGGCTCTTGATGTGGCCAAGGGAATGAATTACTTGCACACAAGCCATCCTACTATTGTACATAGAGATCTGAAATCTCCA
AATCTCCTTGTTGATAAAAACTGGGTTGTTAAGGTTTGTGATTTTGGGTTGTCACGTCTGAAGCACCATACGTTTCTGTCTTC
AAAGTCTACTGCAGGAACGCCTGAGTGGATGGCACCAGAAGTTCTAAGGAATGAGCCATCGAATGAAAAGTGTGATGTGTA
TAGCTTCGGTGTGATACTATGGGAGTTGGCTACCTTGCGCATCCCATGGAGTGGGATGAATCCGATGCAGGTTGTTGGAGCTG
TTGGATTTCAAGACAGACGCCTAGAAATCCCAGAAGAGGTTGATCCAATGGTTGCACAGATAATAAATGATTGTTGGGAAGT
TGAGCCACGCAAGCGGCCGTCGTTCTCACAGCTCATGTCTCGTCTCAAGCATCTTCAGCATCTGGTGTTTGAAAGAGCAAGC
TCTTCAAGACAAGCACAAGTGCAATAG
>VviMAPKKK60    VIT_14s0030g01440
ATGAAGCACATTTTCAAGAAGCTTCATATAGGGAGTAGCAGTCACGATCCCAATCGATCCAACGAAACCCTAAGCTCCGCCA
CTACATCATCGCCCGCCTGTGCTTCCGATCATCGGACATCTTCCGCGCAGTCTTCGGTGAGCCCTCCCTCCTCCTATCCTTCTC
CGACTACTGTATCCTCCACCGCCGCCTCCACCCTCCCCACCGCCACCTCTCCGGCGGCATCGAATCGGTCGGACTATTTCTTG
TCCGAAGAGGAGTTTCAGGTCCAACTTGCTCTTGCTATCAGCGCTTCCAATTCGGACTTCCGTGATGATTCGGAGAAGGATC
AGATCCGCGCCGCGACGTTGCTCAGCTTGGGTCGCCACCGAACTGATTCGGTCCGCGATAAGGATGAATCGGCGGAGTCTAT
GTCGCGCCGATATTGGGATTACAATGTGCTTGACTATGAAGAGAAAGTGGTAGATGGGTTTTATGATGTGTATGGGCTCTCCA
CAGATCCAGTAATCCAAGGGAAAATGCCATCTCTCACAGATCTTGAAACAAACCTTGGAAACTCTGGATTTGAAGTGATTGT
AGTTAACCGCAGAATTGACCCTGCCCTGGAAGAGTTGGTGCAAGTCGCACACTGTATTGCCTTAGACTGCCCTGCTGCTGAG
GTTGGTGTTTTGGTGCAGAGGCTTGCTGAGATTGTTACAGATCACATGGGTGGGCCAGTAAGGGATGCTAATATTATGTTGGT
GAAGTGGATGGAAAGCAGAAAAGATTTGAGGACATCTCTTCACACAAGCATTTTGCCTGTTGGGTCCTTAAGTATTGGCCTT
TCACGTCACCGTGCTTTGCTTTTCAAGATATTAGCTGACAATGTTGGTGTACCATGTCGACTGGTAAAAGGTAGTCATTACAC
TGGCGTAGAGGATGATGCTGTCAACATAATAAAATTGGATAATGAAAGGGAGTTTTTGGTTGATCTCATGGGAGCCCCTGGG
ACACTTATTCCAGCTGACATTCTTAGTGCAAAGGATAGTTCTTTAAAGTCATATAATCCAAAATTAAGTAAAATCCCAACTCTT
CAGGCATCTAAGGACCCTGGAGGTGTTTACTCAAGACCTAAGCCATTGCTTGGTGATTATGAAGGCAGTAGTCAAACCTCCA
CAATTGAAAATAGTTTGCCTCAGGATAGGAAAGCAAGTTCTGAAAAGATAGAATCCTTGGATTCATTTTCTAGTTCAAGTGGT
GACACTGGTGTTGGTACTTCCAGAATTTCTAAAAGAGTGACTCCTGTTAATCAGTCAGATCTTCGGCCTTCATTGGCAATTGG
GGCCTCTGTGTATAAAGGGAGTCGTGGTGCCAATGCAGTTGGTGATGGTTCGAGGATGAATGTTAACATAGTTCCATATAATC
AAAACAGCACAGAAGACCCTAAAAACCTTTTTGCAGATCTTAATCCTTTCCAGATGATAGGATCCAGCAAGGCTTCTGCACA
GAGCAAACCTATGGAGAATAAAGTTGATGAGTTTCAAAGAGAGAAAAATAGCGCTGCCCCTGGTAGACCCCCTTTGCCATT
GATGTGGAAGAATCGTTATGCTAACAATGAGGTCCCAAGAAAAAAGGAAAATGATTTTGTGGAGGGTCTCTTTCCAAAAATC
AACCGTGAAACCAATGACTACAATCTGCCATCATTAACTTCCAACAATGCTACTACATCTGAAAAAGTTTATTCCGGTGTTTT
CAAGTTATCTGGTAATGCCTATATGAATAACAAAGTTAATGATGACCAAAATTCTTCTTGTAATACCACTTCAATGTTGGCACC
AAGCACAAGTCAGTTCAATAGGTTATCTTTGGATGAGGATGTAAATGCTAATTACAATGAAAAATATCACAAGGATGGGAAA
GTTTTTCAAAGTGACATGGTAGATGCTGCAAAAGAACATGACAAGAATGAAACTGGTCTGCATGATCACAGAAAGTTCAGA
CATGATAGCTTTATGGAGAACAATTTGAGGGAAGCTGAAAGTCCTTGCTCGTCTGTTGACAGTGATGCTGGTAAAGTTGATC
AAATGTTTGAAGATGTAGGTGAATGCGAAATTCCTTGGGAAGACCTGGTTCTTGGGGAAAGGATTGGACTAGGCTCGTATGG
AGAGGTCTACCATGGTGATTGGAATGGCACAGAGGTTGCTGTGAAGAAGTTCTTGGACCAGGATTTCTCAGGTGCTGCTTTG
GCCGAGTTCAAAAGAGAAGTGCGAATAATGCGTAGATTACGTCATCCAAATGTTGTTCTTTTTATGGGTGCTGTTACTCGCCC
TCCAAACCTCTCTATCATCACTGAGTTTCTTCCAAGAGGAAGCTTATATCGGATTCTTCATCGTCCTTCTTGTCAAATTGATGA
GAAGCGTAGAATTAAAATGGCTCTTGATGTGGCAAAGGGTATGAATTGTTTGCACACCAGTTTACCAACAATTGTTCACCGG

GATTTGAAGTCACCAAATCTGCTGGTTGATAAGAACTGGAATGTCAAGGTATGTGATTTTGGGTTGTCACGCCTGAAGCACA
ACACATTTTTGTCATCCAAATCAACTGCTGGAACGCCTGAGTGGATGGCCCCAGAAGTTCTCCGCAATGAAAATTCAAATGA
GAAGTGTGATGTCTATAGCTTTGGAATCATTCTGTGGGAGCTTGCAACTTTAAGGTTGCCTTGGAGTGGGATGAATCCAATGC
AAGTTGTGGGAGCAGTAGGTTTCCAGAACCGTCGTCTTGACATACCAAAGGAAGTTGATCCCTTGGTTGCAAGGATAATCTG
GGAATGCTGGCAGACGGATCCGAACTTGCGCCCATCGTTTGCACAGCTGACAGTAGCTCTGAAGCCCTTACAGCGACTGGT
GATCCCTCAACATTTGGACCAGCAAAGCCTGACCCTCCAGCAAGAGATCTCAGATGAAGTTAGATTGTGCGATTAA
>VviMAPKKK61    VIT_05s0077g00920
ATGGACACGCCTCCAGCTGAAGAGCTGCTGAGGAAGATCCAAGAGCTGGAAGCGGGTCATGCGCATCTCAAGGAAGAGAT
GTCGAAGCTGATGGTTTCTGGACCCAAATCGGACCATGGGCATCAGAGGTCGCACTCGACATCGCCACAGAGGTCGAGGTT
TTCGTCGCCGGCGACATCGTCATGGAGAAAGGGAAGCGGTGGGTTCGACGGACCGGCGGGGTGGAGGAGGGGGTCGGCGT
CGTTCCGGCATTCGTCGCCGTTGCAGAGGGAGAGCCGAAGTCGTGATCCTTCAAGCACTGCCGGCGGTGGTGGTGGCCCGG
CGGCTTACAAATTTAATGATAAGCAGTATTCGAATATTTTGCAGAGCATGGGGCAGTCAATTCATATATTTGATCTCAATGGTC
GCGTAATCTATTGGAATAGAACTGCTGAAAAACTTTATGGTTATTCAGCTGAAGAAGCCCTCGGTCAGCAGGCCATTGAGCT
CCTATCGGATGTCCAGGACTACGCTATTGCTAATAATATAGTGGATCGAGTATCAAGGGGGGAGAGCTGGACTGGGCAGTTCC
CTGTGAAGAATAAGATGGGGGAGAGGTTTCTAGCCGTTGCTACCAACACTCCATTCTATGATGATGATGGTACATTAATTGGG
ATTATTTGCGTATCTAGTGATTCGCAGCCCTTTCAAGAAATAAGAGTTGCAATGTCCAATGAGAGGCAATCAGAAGCTAATGC
GAGCTATAATCGTTCCCGAAGCAGTAGTGCTTCGGCAAAGCTTGGTCTTGATCCTCAGCAGCCTATTCAAGCTGCAATTGCCT
CCAAAATATCAAATTTGGCTTCCAAGGTGAGCAACAAAGTTCGGAAAATTAAGGCTGGGGAGAACAATGTGGTTCGCGAGG
GTGGGAGTGGAGATAGTCATCATTCTGATCATGGTTTCTCAGATGCAGCTTTCTCTGACCATAGGGAGGATGCAACTTCAAGT
GGAGCTAGCACCCCCAGAGGAGATGTGGCACCATCTCCTTTTGGCATATTTTCTCAGGCAACTGCTGATGAGAAGTCCCCAG
GAAAGAACTTAAGAGATTCTGGTGATGAGAATGAAGGGAAACCTGGGATCCACAGGGTTATTACCTCCAAGGCAGAGGCAT
GGATTGGCAAGAAAGTTATGTCATGGCCATGGAAAGGGAATGAACGGGAAGGGTCGGAGGTAAAGACAAACCGGTTTGGT
TGGCCCTGGTTGCAGAATGATCATGAGAATGACATGGTCCAACCAAAGAATCCCAATTTTGGTGCAAAAACCGAAAACCTG
GTGAGTGAAAGTAATCGGCACGGGAATAATGATGCTTCAGGGTCCTGGTCTTCATTTAATGTTAACAGCACGAGCAGTGTAA
GTAGCTGTGGCAGTACCAGCAGTAGTGCTGTTAATAAAGTGGACATGGAAACTGACTGCTTGGATTATGAAATTTTGTGGGA
AGACTTGACTATTGGAGAACAGATTGGGCAAGGATCTTGTGGAACCGTATATCATGGTCTTTGGTATGGATCAGATGTTGCTA
TCAAGGTGTTCTCCAAGCAGGAATATTCAGATGACGTGATACTTTCATTCAGACAAGAGGTATCTCTCATGAAAAGGCTTAG
ACATCCAAATGTTCTGCTCTTCATGGGTGCAGTAACTTCACCTCAGCGTCTATGCATTGTCACTGAGTTCCTTCCACGTGGAA
GTTTATTTCGGTTACTCCAAAGGAACACATCCAGACTAGATTGGAGACGACGTGTACACATGGCTTTGGATATAGCACAAGG
CATGAACTATCTTCATCATTTCAACCCACCTATCATCCATCGTGATTTGAAGTCTTCAAATCTTCTAGTTGATAGAAACTGGAC
TGTGAAGGTTGGTGATTTTGGTCTATCACGTCTCAAACATGAAACATATCTCACAACAAAGACTGGGAAAGGAACGCCTCAA
TGGATGGCTCCAGAGGTTCTCCGTAATGAACCCTCAGATGAGAAGTCTGATGTATATAGTTATGGAGTAATATTATGGGAGCTT
GCCACTGAGAAGATCCCTTGGGATAATCTCAACACAATGCAGGTGATTGGAGCTGTTGGTTTTATGAACCAACGGCTTGACA
TACCAAAAGAAGTGGATTTACGATGGGCTTCTATAATTGAGAGTTGCTGGCACAGTGATCCCCGGAGTAGGCCAACATTCCA
GGAATTGCTGGGAAAGTTCAAAGATATACTGAGACAGCAAACTATGCAATTTCAGGCAGCCCGTGCTGCAGCTGGAGATAA
CACCCAAAAGGAGTTGTAG
>VviMAPKKK62    VIT_18s0166g00290
ATGGATTTGATTGAAGGTGTGGGGGAGAGTTCGTCTCCTCCGCGGAGCTTCGGGTGCTTCGGTGTGTATGATGTGAGGAACG
ATGTGTATAATCGGTTGATGGAGAGTGGGAACGAGGAGGCAGTGAGTAATCCCGAGTTTCGCGAACAGTTGGATGCTCACTT
CAATCGCTTGCCTCCTAGTTATGGACTAGATGTCAATATTGATAGAGTGGATGATGTTCTGTTACATCAAAAACTGCTTGCTTT
GGCGAAGGAACCAGATAAACGACCTGTTTATCATGTTCGTTTCTTGGAGAATTTATCAACTAAAGTAGATGGTAATGATGATC
AACAATCTATGAGTGTTCTTTCTACCGCAAGGCCATGTTGTAATGCAGATAATGAAGGAGTTGTGCCATCACATAACAGGAAT
GAGATTGATTTTGAACCTTGCTCTAAGCTTGAGGACCTAAATTTGGATGTTAAGAAGGATTCCACAGACATGGAGAGAAGAT
GTCTTATGGAGAATTCTTCTAGAAGACAGGAAACTTCAAATGTTCCAATTCATGAAGTAATATTTTCTACCATTGACAAGCCC

AAGCTTCTTAGTCAGCTTTCTGCTTTGCTTTCCGACATAGGACTTAACATCCGTGAAGCGCATGTCTTCTCGACAATTGATGG
CTACTCCTTGGATGTCTTTGTAGTGGATGGATGGCCTGTTGAGGATACAGATGGTTTGAGTGAAGCTATGGAAAAAGCAATT
GCTAGAAGTGAGGGTTCGTGGTCTGGTTCTTCACATTCTCATTCAGCAGTGGAAAAAGCATTAGCAGCACAAGTCAAATCTG
GAGATTGGGAAATAGATAGAAGATTATTGAAGATAGGAGAAAGAATTGCATCTGGATCTTGTGGAGATTTGTATCGTGGAGTT
TATCTTGGTCAGGATGTTGCTGTTAAGATTCTTAGGTCTGAGCATTTGAATGAATCTCTGGAGGATGAATTTGAACAAGAAGT
GGCAATTCTGAGGGAGGTTCAACATAGAAATGTTGTTCGATTTATTGGTGCCTGTACAAGGTCTCCACATTTGTGCATAGTAA
CTGAGTATATGCCTGGAGGAAGCCTGTATGATTATTTGCATAAGAATCACAATGTCTTGAAGCTCCCTCAATTGCTGAAGTTT
GCAATTGATGTCTGTAAAGGAATGGGGTATTTGCATCAAAACAACATAATTCACAGGGATCTGAAGACAGCAAATTTGCTAA
TGGATACTCATAATGTTGTTAAGGTGGCAGATTTTGGTGTTGCTCGGTTCCAAAATCAAGAGGGAGTGATGACTGCAGAGAC
TGGAACTTACAGATGGATGGCACCTGAGGTTATAAATCACCTACCATATGATCAAAAGGCAGATGTATTCAGTTTTGCCATTG
TACTGTGGGAGCTAACAACGGCTAAGATTCCATATGATAACATGACTCCATTACAAGCTGCCCTAGGAGTGAGACAGGGACT
GCGACCAGATCTTCCTGAGAATACACATCCCAAACTGGTGGACATGATGCAGAGATGTTGGGAGGCAGTTCCTGGAAATCG
GCCTTCCTTCTCTGAGATAACAGTTGAACTTGAAGAATTGCTACAAGAAGTTCAGGGAACTTCAAGAGCATCAAATGGCAA
CTGA
>VviMAPKKK63    VIT_03s0038g03040
ATGGTGATGGAAGACAACGAGAGTTGCAGTAGCAGAGTTCACGATTCGTCGTCGCCGGCGCAGTCGCGGCAACAGCGCCA
GAAGCTGGAGGTATACAATGAGGTTCTGCGTCGGCTCAAGGATTCCGACAACGAGGAGGCGTTTGAGCCTGGTTTCGACGA
GGAACTGTGGGCTCACTTCGTTCGACTCCCCACTCGGTATGCTCTGGATGTGAATGTGGAGAGGGCTGAAGACGTTCTAACG
CACAAGAGATTGCTGCATTTGGCGCATGATCCTACCAATAGGCCTGCAATTGAAGTTCGCCTTGTGCAGGTTCATCCTATATC
TGATGGGATTCATGGGAACATAGCTGATTCTATTCATTCAAACTCTCCTACTATTGGGCCAGCCCATGGTTCTCCAAAGTACTC
TAGCAAACAGAGCATTCTCCCACCACCTGCCTTTGGATCATCGCCTAATCTTGAGGCCCTTGCAATTGAAGCAAACAATTCTC
ATGTCCAAGATGGGGATGGGGATGATTCTGTACATGCTAGTTCACAGTATTCTCGGCCCATGCATGAAATCACATTTTCATCAG
ATGACAAGCCAAAACTCCTGAGTCAGTTGACTTGCTTGCTTTCTGAGCTTGAGCTCAACATCCAAGAAGCACATGCCTTTTC
CACAGTGGATGGTTACTCCTTAGATGTCTTTGTTGTTGATGGATGGCCATATGAGGAAACAGAGCAGCTGAGAACTGCACTG
GAAAAGGAAGTTTTTAAGATTGAGAAGCAATCTTGGCCAAATCATCATTCCTTATCTCCTACAGGCGAGCAAGAAGAAACAG
GAATCAAATGCGAATCTGATTTTGTGACAATACCTAATGATGGGACTGATGTCTGGGAAATCGATGTTAGGCAGTTGAAATTT
GAGAACAAAGTTGCATCGGGTTCATATGGTGATCTGTACAAAGGTACATACTGTAGTCAGGAAGTGGCAATTAAAGTCCTCA
AGCCTGAGCGTTTAAATTCAGACATGCAGAAAGAGTTTGCACAGGAAGTCTTTATTATGAGGAAAGTTAGGCACAAGAATG
TGGTACAGTTCATTGGTGCATGTACAAGGCCTCCAAGCTTATACATTGTGACAGAATTTATGTCTGGTGGAAGTGTGTATGAC
TATCTACACAAGCAAAAGGGTGTTTTTAAGCTTCCTGCATTGCTCAAAGTATCAATTGATGTTTCCAAGGGAATGAACTACTT
GCACCAAAATAATATAATCCACAGGGATTTGAAAGCTGCCAATCTTCTGATGGATGAGAATGAAGTTGTTAAGGTAGCAGATT
TTGGAGTTGCCAGAGTGAAAGCTCAATCTGGAGTTATGACTGCAGAAACTGGGACATATCGATGGATGGCTCCAGAGGTCAT
AGAACACAAGCCATATGATCACAAAGCTGATGTTTTTAGTTTTGGAATTGTGTTATGGGAGTTGCTAACTGGAAAGCTTCCAT
ATGAGTACTTGACCCCATTACAAGCAGCCGTTGGAGTCGTCCAAAAGGGTCTCCGGCCAACCATGCCAAAGAACACTCATC
CAAAGCTTGCAGAGTTGCTCGAGAGGTGCTGGCAACAAGATCCAACATTAAGACCCGACTTCTCTGAAATTATAGAGATTCT
TCAGCAAATAGCCAAAGAGGTTGGAGATGAAGAAGACCGGCGCAAGGAGAAATCTTCTAGTGGATTCCTATCAGTTCTCAG
ACGTGGCCATCACTGA
>VviMAPKKK64    VIT_18s0001g00720
ATGCGTCCGGGTTTTGATGATGAACTTTGGGCACACTTCTCTCGACTTCCGACTAGGTATGCGCTTGATGTAAATGTAGAGAG
GGCCGAAGATGTTCTTACACACAAGAGACTACTACAATTGGCACATGATCCAGCCACTAGACCAGCACTTGAAGTCCGCCTT
GTGCAGGTCCATCCCATTTCTGGTGGAATTCATGGTGATTCTGATCCTTCAAACCCTTTAAAAAAGGTGGATGCTCAAAGTCA
TTATCACACCAGTCAGAGGAGCACTCATCCACCATCTACCTTTGGATTGTTGCCTAATATGAAAGTTTTGGTTGAAGCCAAAA
ATTCACATGTTCAAGATGGAGAAGGTGATGTGGATGCCAATCTACGCTTTTGGAGGCTGATGCATGAAGTTATAATTTCAACA
AATGACAAGCCCAAGCTCCTCAGTCAGTTGACCTCATTACTTTCTGATATTGGATTGAACATTCAAGAAGCACATGCTTTTTC

CACGACAGATGGCTACTCCTTGGATGTTTTTGTTGTTGAGGGCTGGGCACATGAGGAAACTGAGCAGCTTAGAAATGTACTG

CTAAAGGAAATTCAAATGATTGAGAAGCAACCTTGGTCAGAATTCCAGCTCATATCTCCTGGGAGGGAGCAAGGACACCCT

GAAAAACAGCTTATCCCTAGTCACATAAACTTAACCATTGATGGGGCTGATGTCTGGGAAATTGATGCCACGCTGTTGAAATT

TGAAAACAAAATTGCATCTGGATCGTATGGTGATTTGTATAAAGGTACTTTTTGTAGCCAGGATGTGGCTATTAAAGTTCTCA

AGACTCAGCATTTAAATGAAGATATGTGGAGGGAATTTTCTCAAGAAGTCTATATTATGAGAAAAGTTCGGCACAAGAATATT

GTGCAATTTATTGGTGCATGTACCAGACCTCCAAGCCTGTGCATTGTGACTGAGTTTATGTTTGGTGGAAGTGTCTATGACTT

TCTGCATAAGCAAAAGGGCAGTTTTAAGCTTCCATCCTTGCTCAAAGTAGCAATTGATGTGTCCAAGGGAATGAATTACTTG

CATCAAAATGACATAATCCACAGGGACCTGAAAGCTGCCAATATTCTGATGGATGAAAACAAAGTTGTTAAGGTTGCTGATT

TTGGTGTTGCTAGAGTACAAGCTCAATCTGGAGTCATGACTGCTGAAACTGGAACTTATCGTTGGATGGCTCCAGAGGTCAT

TGAACATAAACCATATGATCACAAGGCTGATGTTTTCAGCTTCGGAATTGTATTGTGGGAGCTTCTAACAGGAAAGCTTCCTT

ATGAGCACTTAACCCCATTACAAGCTGCAGTTGGTGTGGTCCAGAAGGGTCTAAGGCCTACTATTCCGAGTCACACTTATCCA

TCACTTGTAAAATTGATCAAGAGATGCTGGCACCAGGAACCATCTTTAAGACCTGAATTCACTGAAATTATGGAGATATTGCA

GCAGATAGCTAGCAAGGTTGTGGAAAAAAAGAGAGAGATCTTAGAGGGAAGAGGGAAAAGGCAGAAGGAGAAATCTATCT

AG

>AtMEKK1 (AT1G09000)

ATGCAAGATTTCTTCGGCTCCGTTCGTCGATCGCTTGTTTTCCGTCCTTCTTCCGACGACGATAACCAGGAGAACCAGCCTCC

GTTTCCCGGTGTTCTCGCCGATAAGATCACCTCTTGCATCCGCAAATCGAAGATTTTTATCAAACCCTCCTTCTCGCCTCCTCC

TCCTGCTAACACTGTAGACATGGCACCTCCGATTTCGTGGAGGAAAGGTCAGTTAATTGGTCGCGGCGCGTTTGGTACGGTG

TACATGGGTATGAATCTTGACTCCGGGGAGCTTCTCGCCGTCAAACAGGTTCTGATTGCAGCCAATTTTGCTTCCAAGGAAA

AGACTCAGGCTCATATTCAGGAGCTTGAAGAAGAAGTTAAGCTTCTTAAAAATCTCTCCCATCCTAATATAGTTAGATATTTG

GGTACAGTGAGGGAAGATGATACCCTGAATATCCTTCTCGAGTTTGTTCCCGGTGGATCGATATCATCGCTCTTGGAGAAATT

TGGACCTTTTCCTGAATCAGTTGTCCGGACATACACAAGGCAACTGCTTTTAGGGTTGGAGTACCTGCACAATCATGCAATTA

TGCACAGAGACATTAAGGGGGCTAATATCCTTGTGGATAATAAAGGATGCATTAAGCTTGCTGATTTTGGTGCATCCAAACAA

GTAGCTGAGTTGGCTACGATGACTGGTGCAAAATCTATGAAAGGGACACCATATTGGATGGCTCCGGAAGTTATTCTTCAAA

CTGGACATAGCTTCTCTGCTGACATATGGAGCGTCGGCTGTACAGTTATTGAAATGGTGACTGGGAAGGCTCCTTGGAGTCA

GCAGTATAAAGAGGTTGCTGCTATCTTCTTCATAGGAACAACAAAATCACATCCTCCAATACCTGATACTCTCTCCTCTGATGC

AAAAGATTTTCTGCTCAAGTGTCTGCAGGAGGTACCAAATCTGCGGCCAACCGCATCTGAGCTACTAAAGCATCCTTTTGTT

ATGGGGAAACACAAGGAGTCTGCTTCTACTGATCTTGGTTCTGTCCTGAACAATCTTAGCACTCCACTACCGTTACAGATAAA

TAACACCAAGAGCACTCCAGATTCTACTTGCGACGATGTAGGTGACATGTGTAACTTTGGCAGTTTGAATTATTCACTTGTAG

ATCCTGTGAAATCAATCCAAAACAAAAATTTATGGCAACAAAATGATAATGGAGGTGATGAAGACGATATGTGTTTGATAGAT

GATGAGAATTTCTTGACATTTGACGGAGAAATGAGTTCTACCCTTGAAAAAGATTGTCATCTGAAGAAGAGCTGTGATGACA

TAAGTGATATGTCCATTGCTTTGAAGTCCAAATTTGACGAAAGTCCTGGTAATGGAGAGAAAGAGTCTACAATGAGCATGGA

ATGTGACCAACCTTCATACTCAGAGGATGATGATGAGCTGACCGAGTCAAAAATTAAAGCTTTCTTAGATGAGAAGGCTGCA

GATCTAAAGAAGTTACAGACTCCTCTCTATGAAGAATTCTACAATAGTTTGATCACATTCTCTCCCAGTTGTATGGAGAGTAAT

TTAAGTAACAGTAAAAGAGAGGACACTGCTCGTGGTTTCCTGAAACTGCCTCCAAAAAGCAGGTCACCGAGTCGGGGCCCT

CTTGGTGGTTCACCTTCAAGAGCAACAGACGCAACTAGTTGTTCCAAGAGCCCAGGAAGTGGAGGTAGTCGTGAATTGAAT

ATTAACAATGGAGGTGATGAAGCTTCACAGGATGGTGTATCAGCACGGGTCACAGACTGGAGGGGTCTCGTTGTTGACACT

AAGCAGGAATTAAGCCAGTGTGTTGCTTTGTCAGAGATAGAGAAGAAGTGGAAGGAAGAGCTTGATCAAGAACTGGAAAG

AAAGCGACAAGAAATCATGCGCCAAGCAGGGTTGGGATCATCCCCAAGAGACAGAGGCATGAGCCGACAGAGAGAGAAG

TCGAGGTTTGCATCACCAGGAAAATGA

>AtMEKK2 (AT1G54960)

ATGGTTTTCGCCAAATCCCAGTCACCTCCGAATAACTCCACCGTACAAATCAAACCTCCGATTCGGTGGCGGAAAGGTCAGT

TAATTGGCCGTGGCGCTTTTGGTACTGTGTATATGGGAATGAATCTCGATTCCGGTGAGCTTCTCGCCGTTAAACAGGTTCTG

ATTACATCTAATTGTGCATCCAAGGAAAAAACTCAGGCTCATATTCAGGAGCTTGAAGAGGAAGTGAAGCTACTCAAGAATC

TCTCTCATCCAAATATAGTTAGATATTTGGGTACGGTGAGGGAAGATGAAACTTTGAATATCTTGCTTGAATTTGTTCCTGGTG
GATCTATATCTTCACTCTTGGAGAAATTTGGAGCCTTTCCTGAATCTGTTGTTCGGACATACACGAACCAACTGCTTTTGGGA
TTGGAGTACCTTCATAATCATGCCATTATGCACCGTGACATTAAGGGTGCTAATATCCTTGTGGATAATCAAGGATGCATTAAA
CTTGCTGATTTTGGTGCGTCCAAACAGGTAGCGGAGTTGGCTACTATTTCGGGTGCCAAATCTATGAAAGGAACTCCCTATTG
GATGGCTCCAGAAGTTATTCTTCAAACCGGGCATAGCTTTTCTGCTGATATTTGGAGTGTAGGATGCACAGTGATTGAAATGG
TGACTGGAAAAGCTCCTTGGAGCCAGCAATATAAAGAGATTGCTGCTATTTTCCACATTGGAACGACGAAATCGCATCCTCC
AATCCCTGACAATATCTCCTCTGACGCAAATGATTTTTTGCTCAAGTGTCTGCAGCAGGAACCAAATCTGCGGCCAACCGCT
TCTGAGCTGCTAAAGCATCCATTTGTTACGGGCAAACAGAAGGAATCTGCGTCTAAAGATCTTACTTCATTTATGGACAATTC
ATGCAGTCCTTTACCATCAGAGTTGACTAACATTACGAGCTATCAAACATCTACGAGTGACGATGTAGGAGACATCTGTAACT
TGGGTAGTCTGACTTGTACACTTGCTTTCCCTGAGAAATCAATCCAAAATAACAGTTTGTGTCTGAAAAGTAATAACGGGTAT
GATGACGATGATGATAATGATATGTGTTTGATTGACGATGAGAATTTCTTGACATATAATGGAGAGACTGGCCCTAGTCTTGAC
AATAATACTGATGCCAAGAAGAGCTGTGATACCATGAGTGAGATCTCTGATATTTTGAAGTGCAAATTTGACGAAAATTCTGG
AAACGGAGAAACAGAGACGAAAGTTAGTATGGAAGTTGACCATCCATCATACTCGGAGGATGAAAATGAGCTGACTGAGTC
GAAAATCAAAGCTTTCTTAGATGACAAGGCTGCAGAGTTAAAGAAGTTACAGACGCCTCTGTACGAAGAATTCTACAACGG
TATGATCACATGCTCCCCCATCTGCATGGAGAGTAACATCAATAACAATAAACGAGAGGAGGCACCTCGTGGTTTCTTGAAA
CTGCCTCCAAAAAGTCGGTCTCCGAGTCAGGGCCATATTGGTCGATCACCTTCTAGAGCAACAGATGCAGCCTGTTGTTCCA
AGAGTCCAGAAAGTGGTAATAGCTCTGGTGCCCCGAAGAATAGCAATGCAAGTGCTGGTGCTGAACAAGAATCAAACAGTC
AAAGTGTCGCGCTGTCGGAGATAGAGAGGAAGTGGAAGGAAGAGCTTGATCAAGAACTTGAAAGAAAGCGAAGAGAGAT
TACACGGCAAGCAGGGATGGGATCATCCCCGAGAGATAGAAGCTTGAGCCGACATAGAGAGAAGTCAAGATTTGCATCTCC
AGGCAAATGA

>AtMEKK3 (AT1G53570)

ATGCCTACTTGGTGGGGAAGAAAGTCCTGCAAGAACAAGGACGATAATCACAGAGGAATCATCTCCACAGATAGAGATATC
AAGAGCTCTGCTGTTGTTGTTGATCCTCCTCTCACTCCTACTCGTGGTGGTACACCTCGTTGCAGTCGTGAATTCGCTGGAGC
TTCTTCTGCTTTCTCTGGTTTTGACTCTGATTCTACCGAGAAGAAAGGTCACCCTCTTCCTCGTCCTTTGCTCTCTCCTGTTTC
GATCCATCATCAAGATCATGTAAGCGGATCCACTTCTGGATCTACATCTGTTTCTAGCGTTAGCTCATCTGGATCAGCTGATGA
TCAGAGTCAACTTGTTGCTTCTAGGGGTCGTGGTGATGTGAAATTCAATGTAGCAGCAGCACCACGTAGTCCTGAGAGAGTT
TCTCCAAAGGCAGCTACTATTACTACTAGGCCCACGTCTCCACGGCATCAGCGTTTGTCTGGAGTTGTGAGCTTAGAGTCTTC
TACAGGGAGGAATGATGATGGAAGGTCCTCCTCTGAGTGTCATCCTTTGCCTCGACCACCTACTTCTCCTACAAGCCCTTCTG
CTGTGCATGGTTCCAGGATTGGAGGAGGCTACGAGACCTCTCCTTCGGGGTTTTCCACGTGGAAAAAAGGGAAATTTCTTG
GGAGTGGCACCTTTGGTCAAGTCTATCTTGGTTTCAACAGTGAGAAAGGGAAAATGTGTGCTATTAAAGAGGTCAAGGTCAT
TTCTGACGATCAAACATCAAAAGAATGTCTGAAGCAACTAAATCAGGAGATAAATTTGCTTAACCAGCTTTGTCATCCGAATA
TTGTCCAGTATTACGGAAGTGAACTGAGTGAAGAAACCTTGTCCGTCTACTTGGAGTACGTGTCAGGTGGTTCAATCCATAA
ACTACTTAAGGATTATGGTTCTTTCACTGAACCCGTTATCCAAAACTACACGCGGCAGATTCTTGCTGGGCTTGCCTATTTACA
TGGACGAAATACAGTACATAGGGACATCAAAGGAGCAAATATATTAGTGGATCCGAATGGTGAAATCAAGTTGGCAGACTTT
GGGATGGCCAAACATGTAACAGCCTTTTCTACTATGCTTTCTTTTAAAGGGAGTCCTTACTGGATGGCACCCGAGGTTGTGAT
GAGCCAAAATGGCTACACTCATGCAGTCGATATCTGGAGTTTGGGTTGTACTATTCTGGAAATGGCAACATCAAAGCCACCT
TGGAGCCAGTTTGAAGGGGTTGCTGCGATTTTCAAAATCGGAAACAGTAAAGACACCCCGGAAATACCTGATCACCTTTCA
AATGATGCAAAGAATTTTATAAGGCTTTGTCTGCAACGAAATCCGACAGTACGTCCTACAGCTTCTCAGCTTCTAGAACACC
CTTTTCTACGTAACACAACAAGAGTGGCTAGTACTAGTTTGCCCAAAGACTTCCCCCCACGTTCCTATGATGGAAACTTCTCA
CTGCAGCCTACAAGGGAACCCTATCCAGGGAGACTGAGCCATGATAATTATGCAAAACAGCCATTGTCTAGAACTATAAAGA
GCCCGAGCAGAGAAAACGTAAGAGCTATCACATCCTTACCAGTATCTCCATGTTCAAGCCCTTTACGCCAACTTGGGCCAGC
ATACAAAAGTTGTTTCCTGTCACCTCCTCACCCGTCTTACGCATTTCCTGGGCAAGACAGTGGGTACAACCTAGCAGAGTTT
GCTGCAAGCCCTTTCAGGATGAAGAAAGACGCAATGATGGAACCATCTAGTTTCAGGACTCAAACACCGAATTCACCATTG
AGATCAAGACTGGTGTAG

>AtMEKK4 (AT1G63700)

ATGCCTTGGTGGAGTAAATCAAAAGATGAAAAAAAGAAAACTAATAAGGAGAGTATCATTGATGCGTTTAATCGGAAACTG
GGATTCGCATCTGAGGATAGGTCTAGTGGAAGATCAAGAAAATCAAGACGACGACGTGATGAGATTGTGTCTGAAAGAGGA
GCTATATCTCGATTACCATCAAGATCTCCCTCTCCTTCTACTCGGGTTTCACGCTGTCAGAGTTTTGCAGAAAGATCTCCTGCT
GTACCTCTTCCTCGTCCTATTGTCCGTCCTCATGTAACCAGTACTGATTCAGGAATGAATGGATCACAGAGACCAGGTTTAGA
TGCAAATTTGAAGCCGTCATGGTTGCCACTTCCAAAGCCCCATGGTGCTACAAGCATACCTGATAATACCGGTGCTGAGCCT
GATTTTGCCACTGCTTCTGTGTCTAGTGGAAGTTCTGTGGGTGACATTCCATCTGATTCTCTTCTCAGTCCATTGGCGTCTGAT
TGTGAAAATGGGAACCGAACACCAGTAAACATATCTTCGAGGGATCAGTCAATGCATAGTAACAAAAACTCAGCTGAGATG
TTTAAGCCAGTCCCTAATAAAAATAGGATTCTGTCTGCATCTCCTAGGCGGAGACCTCTGGGAACTCATGTGAAGAATCTACA
AATCCCCCAACGAGATTTAGTGCTATGCAGTGCTCCAGATAGTTTGTTGTCTAGTCCTTCCAGGAGTCCAATGAGATCCTTTA
TTCCAGATCAAGTCTCAAACCATGGGTTGTTGATTAGTAAACCATATTCAGATGTTTCCTTGCTTGGATCTGGACAGTGCTCA
AGCCCCGGTTCAGGTTACAACTCAGGTAACAATTCCATTGGTGGAGATATGGCTACTCAGCTGTTTTGGCCTCAAAGCAGGT
GTAGCCCTGAATGTTCCCCTGTGCCTAGTCCAAGAATGACAAGCCCTGGTCCTAGCTCTAGAATACAGAGTGGTGCTGTTAC
ACCTCTTCATCCTCGAGCTGGAGGGTCAACTACTGGGTCTCCTACTAGAAGACTTGATGATAACAGACAGCAAAGCCATCGT
CTGCCTCTCCCGCCGTTATTAATCTCTAATACTTGTCCGTTTTCACCCACATATTCAGCAGCGACATCTCCGTCTGTCCCCCGA
AGTCCGGCAAGGGCAGAGGCTACGGTTAGCCCTGGATCGCGATGGAAAAAAGGGAGATTGCTGGGGATGGGAAGTTTTGG
ACATGTGTATCTTGGCTTTAACAGTGAAAGTGGGGAGATGTGTGCCATGAAAGAGGTTACTCTATGCTCAGATGATCCTAAGT
CAAGGGAGAGTGCACAACAATTGGGGCAAGAAATTTCAGTTCTAAGCCGTTTACGACACCAAAATATAGTGCAGTATTATGG
CTCTGAAACCGTCGATGACAAGCTGTATATATATCTGGAGTATGTCTCCGGTGGTTCGATCTATAAACTTCTTCAAGAGTATGG
ACAATTTGGTGAGAATGCCATTCGTAACTACACACAACAAATTTTATCAGGGCTCGCATATTTGCACGCCAAAAATACTGTTC
ATAGGGACATCAAAGGAGCAAATATATTGGTGGATCCTCATGGACGAGTAAAAGTTGCTGATTTTGGGATGGCAAAACATATT
ACTGCTCAATCTGGTCCTTTATCATTCAAGGGGAGCCCATATTGGATGGCACCTGAGGTGATAAAGAATTCAAATGGCAGTAA
CCTTGCGGTCGACATATGGAGTCTTGGATGTACTGTTTTAGAAATGGCTACAACGAAACCTCCATGGAGCCAGTATGAAGGG
GTTCCTGCTATGTTCAAGATTGGAAACAGCAAGGAGCTTCCAGATATCCCTGATCATTTATCTGAAGAGGGGAAGGATTTTGT
AAGAAAATGCCTACAAAGAAACCCCGCAAATCGTCCTACAGCTGCTCAGCTTTTGGATCATGCTTTTGTAAGAAATGTGATG
CCGATGGAAAGGCCTATTGTGAGTGGCGAGCCTGCAGAAGCCATGAATGTAGCTTCGAGCACCATGAGATCACTGGACATT
GGACATGCAAGGAGTCTTCCGTGCTTAGACTCGGAAGATGCAACCAATTACCAGCAGAAAGGATTAAAACATGGCTCGGGA
TTCAGTATATCCCAATCTCCTAGGAACATGTCATGCCCGATTTCACCAGTCGGTAGTCCAATCTTTCACTCGCATTCACCACAC
ATTAGCGGAAGAAGATCTCCATCCCCAATATCTAGTCCCCACGCTCTCTCTGGTTCATCAACACCTTTAACTGGGTGTGGTGG
AGCCATCCCGTTCCATCACCAAAGACAAACTACAGTTAACTTCTTGCATGAAGGCATAGGATCAAGCAGAAGCCCGGGAAG
TGGCGGAAATTTCTACACCAACAGTTTCTTTCAGGAGCCTAGTAGGCAGCAAGATCGGTCGCGGAGTAGTCCAAGGACTCC
TCCTCATGTATTTTGGGACAACAACGGATCGATCCAGCCAGGCTATAATTGGAACAAGGACAACCAGCCAGTCCTATCTGAT
CATGTGTCCCAACAGCTCTTAAGTGAGCATCTGAAACTGAAGTCCCTCGACCTGAGACCCGGTTTTTCAACTCCCGGATCAA
CAAACAGAGGACCCTAA

>AtMEKK5 (AT5G66850)

ATGCGTTGGCTTCCGCAAATCTCGTTCTCGTCTCCTTCTTCTTCTCCATCTTCTTCTCTAAAACCCGTGGCTTCTTACTCTGAA
TCTCCGGATCCAGATCGTAATCAGGATCGGGATCGGTTTCATCGCCGCTTGTTTCGTTTTAACCGTGGCAGGCTCACCCGTCA
GCGGAAGCTTCGTCACTTGACGGATGACGATGTTTTGTTGGGAGAACGTCGTGCTTCTACCTCTTCCTCCACCTTCGATTCCG
GTTTAACTCGCTCTCCCAGCGCTTTCACCGCCGTTCCTCGCTCTCCTTCTGCCGTTCCCTTACCCTTACCTCTCCCCTTACCGG
AGGTCGCCGGGATTCGAAATGCCGCAAACGCTAGAGGATTGGATGACAGAGATCGAGATCCCGAGAGACTTATTTCTGATCG
CACCTCTTCTGGTCCTCCTCTCACCAGCGTCAATGGCGGCTTTGCCCGTGACTCGAGGAAAGCTACGGAGAATTCGTCATAT
CAAGATTTTAGTCCGAGAAACAGAAATGGTTATTGGGTGAATATTCCAACCATGAGTGCACCAACGAGTCCATACATGAGTC
CTGTGCCTAGTCCACAAAGGAAGAGTACTGGCCACGATTTGCCTTTTTTTATTTGCCTCCTAAAAGCAATCAAGCTTGGTCT
GCTCCAGATATGCCACTTGATACCTCTGGTCTTCCTCCTCCTGCATTTTATGATATTACTGCCTTTAGTACCGATAATTCTCCCAT

CCATAGTCCACAACCTCGGAGTCCACGGAAACAGATCAGAAGCCCACAACCTAGCAGACCATCTTCACCGTTGCATTCGGT

TGATAGCTCAGCTCCACCGCGAGATAGTGTTTCCTCACCTTTGCATCCGAGGTTGTCTACTGATGTTACGAATGGGCGACGTG

ATTGCTGCAATGTTCATCCTTTGCCTCTCCCTCCTGGAGCTACTTGTTCCTCTTCATCAGCTGCTTCTGTTCCGTCCCCGCAGG

CTCCTCTCAAACTGGATTCATTCCCAATGAATTCCCAGTGGAAGAAAGGGAAGCTAATAGGTCGTGGTACTTTTGGAAGTGT

TTACGTTGCAAGCAACAGCGAAACTGGAGCATTGTGTGCGATGAAAGAAGTTGAGCTATTTCCTGATGACCCCAAATCCGC

AGAGTGTATAAAGCAATTAGAGCAGGAGATCAAACTTCTAAGTAACCTTCAACATCCAAACATTGTGCAGTATTTTGGTAGC

GAGACAGTAGAAGATCGTTTCTTTATATACCTGGAATATGTTCACCCGGGTTCAATAAACAAATATATCCGTGATCATTGCGGC

ACCATGACAGAATCTGTTGTTCGCAATTTTACTCGTCACATTTTGTCTGGGCTGGCTTATTTGCACAATAAAAAGACTGTACAT

AGGGATATCAAAGGTGCTAATCTCCTTGTTGATGCTTCTGGGGTTGTCAAGCTTGCTGATTTCGGCATGGCTAAACACCTTAC

TGGACAAAGAGCTGATCTCTCGTTAAAGGGAAGCCCGTACTGGATGGCACCAGAGCTCATGCAAGCTGTGATGCAAAAAGA

TAGCAACCCAGATCTGGCTTTTGCTGTTGATATATGGAGTTTAGGATGTACAATCATTGAGATGTTCACTGGGAAGCCTCCTT

GGAGTGAGTTTGAAGGGGCTGCAGCTATGTTCAAGGTCATGAGAGATAGCCCACCGATACCTGAATCAATGTCACCTGAGG

GTAAAGACTTCCTGAGATTATGCTTCCAGAGAAACCCAGCTGAGCGACCAACCGCATCTATGTTGCTAGAACACCGGTTCCT

AAAGAACTCTTTGCAACCAACCTCACCAAGCAACAGTGATGTCTCTCAATTATTTAATGGGATGAACATAACGGAACCAAGC

AGTAGAAGGGAGAAGCCAAATTTCAAACTAGACCAGGTCCCGCGAGCTAGAAACATGACATCCTCAGAGAGTGAAAGTGG

GCAACAGCAGCAGCAACAACAGTACCGGTCTCCCGATCTAACAGGAACCGTGAACCGTCTGTCTCCTCGTTCCACTCTGGA

GGCTATCCCAAGCCCGTGTCCTTCCCAACGACCTAAGCCCAGCAGCAGTGACAGGAGAAGAACGGGCGTCACTTCAGATCA

CCTTTGA

>AtMEKK6 (AT3G07980)

ATGGCGCGACAGATGACGTCATCCCAGTTCCACAAATCAAAGACTCTCGACAACAAATACATGCTAGGAGATGAAATTGGTA

AAGGAGCTTATGGTCGAGTTTATATAGGATTAGACTTGGAGAATGGAGACTTTGTTGCCATTAAACAAGTCTCTTTGGAAAAC

ATTGGTCAAGAGGATCTTAACACCATAATGCAAGAAATCGATCTCCTAAAGAACTTGAACCATAAAAACATTGTCAAGTATCT

CGGATCGTTGAAGACAAAGACTCACCTTCACATTATTCTGGAGTACGTTGAGAATGGCTCTCTTGCAAACATTATTAAACCTA

ATAAATTTGGACCTTTCCCAGAGTCGTTGGTGACTGTTTACATTGCACAGGTCTTGGAAGGTTTAGTATATCTTCATGAGCAG

GGTGTCATACATCGTGATATCAAGGGTGCAAATATTTTGACAACCAAGGAGGGCCTTGTTAAGCTTGCTGATTTTGGAGTTGC

CACAAAACTCAACGAGGCTGATTTTAACACTCACTCAGTGGTCGGAACTCCTTACTGGATGGCTCCTGAGGTTATTGAATTG

TCAGGAGTTTGTGCTGCTTCTGACATTTGGAGTGTTGGATGCACTATTATTGAACTTTTGACTTGTGTACCTCCTTACTATGAT

CTGCAACCCATGCCAGCCCTCTATCGCATTGTTCAGGATGATACCCCTCCTATTCCTGATAGTCTTTCTCCTGATATTACAGACT

TCCTAAGACTGTGCTTCAAGAAGGATTCCAGGCAGAGGCCTGATGCGAAGACACTTCTCTCTCACCCTTGGATACGTAACTC

GAGACGAGCATTGCGGTCATCGCTTCGGCATAGTGGAACCATCAGATATATGAAGGAAACCGATTCAAGTTCAGAGAAGGAT

GCTGAAGGTAGTCAAGAAGTAGTTGAAAGCGTTTCAGCAGAAAAAGTAGAGGTGACAAAAACTAACTCGAAAAGCAAATT

ACCTGTTATAGGTGGGGCGAGTTTTAGGTCTGAGAAAGATCAATCTTCACCCAGTGATCTTGGTGAAGAAGGAACGGATTCA

GAAGATGATATCAATTCAGATCAAGGTCCTACATTGTCTATGCACGATAAGTCTTCTCGTCAATCCGGTACTTGCAGTATTTCT

TCGGATGCAAAGGGGACATCTCAAGATGTATTAGAGAACCATGAGAAATATGATCGTGATGAAATACCTGGAAATCTTGAAA

CGGAAGCTTCTGAAGGTAGAAGGAATACTTTAGCAACAAAGCTGGTTGGAAAAGAATATTCTATCCAGTCATCACATAGTTT

TAGCCAAAAAGGTGAAGATGGGCTTAGAAAGGCTGTGAAGACTCCATCTAGTTTTGGTGGGAATGAACTGACCAGATTCAG

TGATCCTCCTGGGGATGCTTCTTTGCATGATTTATTTCATCCACTGGATAAAGTTCCTGAGGGAAAAACAAATGAAGCCTCAA

CATCGACGCCTACTGCAAATGTAAATCAGGGTGATTCTCCTGTTGCAGATGGTGGAAAGAATGATCTGGCAACGAAATTGAG

GGCTAGAATTGCTCAGAAGCAAATGGAGGGTGAAACGGGGCACTCACAAGATGGTGGTGATCTTTTCCGCTTAATGATGGG

TGTTTTGAAAGATGATGTCCTTAACATCGATGACTTGGTATTTGATGAAAAAGTTCCCCCGGAGAATCTTTTTCCTCTGCAGG

CAGTCGAGTTCAGCAGATTGGTGAGCTCCTTAAGGCCAGATGAATCAGAAGATGCAATAGTAACTTCCTCTTTGAAACTTGT

TGCCATGTTTCGTCAGAGACCTGGGCAGAAAGCAGTATTTGTGACACAGAATGGTTTCCTCCCTCTAATGGATCTACTCGATA

TTCCTAAATCTCGAGTAATATGTGCCGTGCTGCAGCTGATAAATGAAATTGTAAAAGATAATACCGACTTCCTGGAAAATGCT

TGTCTTGTTGGTCTTATTCCTTTGGTAATGAGTTTTGCTGGTTTTGAGAGGGATCGATCACGAGAAATTCGTAAGGAAGCAGC

CTACTTCTTGCAGCAGCTTTGTCAGTCAAGCCCCTTGACGTTGCAAATGTTCATATCTTGCCGTGGAATACCCGTTTTGGTGG

GATTTCTTGAAGCAGACTATGCCAAACACAGGGAGATGGTTCACTTGGCTATTGATGGGATGTGGCAGGTATTTAAACTCAA

AAAATCCACCTCGAGAAATGATTTTTGCCGTATTGCTGCAAAGAATGGAATTCTTCTTAGGCTAGTCAACACTCTTTATAGCT

TGAGTGAGGCAACTCGACTGGCTTCTATATCGGGGGATGCATTGATTTTGGATGGTCAAACTCCACGAGCACGCTCTGGTCA

ACTTGACCCTAACAATCCTATCTTTAGTCAGCGTGAGACTTCACCTAGTGTGATTGATCATCCTGATGGGTTGAAAACTAGGA

ATGGGGGTGGTGAAGAGCCTTCTCATGCTTTAACTTCAAATTCTCAAAGTTCAGATGTCCATCAACCCGATGCCTTGCACCC

AGATGGTGATAGGCCTAGATTAAGCAGTGTTGTAGCAGATGCCACAGAGGATGTTATACAACAACATAGGATATCTCTTTCTG

CCAATAGAACATCAACAGATAAGCTTCAGAAACTGGCGGAGGGTGCGTCTAATGGTTTTCCTGTTACTCAGCCAGATCAAGT

TCGACCTTTGCTTAGCTTATTGGAGAAAGAACCTCCTTCAAGAAAAATTTCTGGTCAACTGGATTACGTAAAGCATATCGCTG

GCATAGAGAGGCATGAAAGTAGACTTCCTCTTTTGTATGCATCAGACGAAAAGAAAACCAATGGAGACCTAGAATTTATAAT

GGCTGAATTTGCAGAAGTCTCTGGACGTGGAAAGGAAAACGGAAATCTTGATACTGCGCCAAGATATTCCAGCAAGACGAT

GACTAAGAAGGTTATGGCTATTGAGAGAGTCGCATCTACGTGTGGGATTGCATCTCAGACAGCATCTGGTGTTCTGTCAGGT

TCGGGTGTTTTAAATGCTAGACCTGGAAGTACCACATCATCTGGTTTACTTGCCCATGCGCTGAGTGCAGATGTCTCAATGGA

TTACTTGGAGAAAGTGGCTGATCTGCTTCTTGAATTTGCCCGAGCTGAAACGACAGTAAAATCATACATGTGCAGCCAAAGT

TTACTCAGTCGTCTTTTCCAGATGTTCAACCGTGTGGAACCTCCTATTCTGTTAAAGATACTGGAATGCACCAATCATTTATCC

ACGGATCCAAATTGCTTGGAAAATCTTCAGCGTGCAGATGCAATCAAGCAATTGATCCCCAACCTTGAGCTTAAGGAAGGGC

CTCTTGTTTATCAGATCCATCATGAGGTACTTAGTGCACTATTCAACCTCTGCAAGATAAACAAGAGGAGGCAGGAACAAGC

TGCTGAAAATGGAATCATTCCGCACCTGATGCTTTTCGTCATGTCGGATTCTCCTCTGAAACAATATGCATTGCCACTACTTTG

TGATATGGCTCATGCATCTCGGAATTCAAGAGAGCAGTTAAGGGCCCACGGCGGTCTGGATGTTTACCTGAGTTTACTCGATG

ATGAATATTGGTCCGTGATAGCCTTGGATTCAATTGCTGTTTGCTTGGCGCAAGACGTTGACCAAAAGGTGGAGCAGGCGTT

TCTCAAGAAAGATGCAATTCAGAAATTAGTTAACTTTTTCCAGAACTGTCCAGAGAGACATTTTGTGCACATTTTGGAGCCA

TTCTTGAAGATCATCACGAAATCATCTTCAATCAATAAGACATTAGCGTTAAATGGACTGACTCCGTTACTCATTGCAAGACT

AGACCACCAAGATGCTATTGCTCGACTTAACCTCCTGAAACTCATCAAGGCCGTTTACGAGAAGCATCCGAAGCCAAAACA

GCTGATCGTAGAGAACGATCTTCCACAAAAACTGCAGAATCTGATAGAAGAACGACGTGATGGACAACGTTCAGGAGGCCA

AGTTCTGGTGAAGCAAATGGCAACATCTCTCCTCAAAGCACTTCACATCAACACCATCTTGTGA

>AtMEKK7 (AT3G13530)

ATGGCGCGGCAAATGACGTCATCTCAGTTTCACAAATCAAAGACTCTCGACAACAAATATATGCTGGGAGATGAAATTGGTA

AAGGAGCTTATGGTCGAGTTTATAAAGGATTAGACTTGGAGAATGGTGACTTTGTGGCCATTAAACAAGTCTCTTTGGAAAA

TATTGTTCAAGAGGATCTTAACACCATTATGCAAGAAATTGATCTTTTGAAGAACTTGAACCATAAAAACATTGTGAAGTATC

TTGGGTCGTCGAAGACAAAGACTCACCTTCACATTATTTTGGAGTACGTTGAGAATGGCTCTCTTGCAAACATTATTAAACCA

AATAAATTTGGACCTTTCCCAGAATCTTTGGTGGCTGTTTACATTGCTCAGGTCTTGGAAGGTTTGGTATATCTGCATGAGCA

GGGTGTTATACACCGTGATATTAAGGGTGCAAATATTTTGACGACAAAGGAGGGTCTTGTGAAGCTTGCTGACTTTGGAGTT

GCCACTAAACTTAACGAGGCTGATGTTAATACTCACTCGGTGGTTGGAACTCCTTACTGGATGGCTCCTGAGGTTATTGAAAT

GTCAGGAGTTTGTGCTGCTTCTGACATTTGGAGCGTTGGATGCACTGTTATCGAACTTCTGACATGTGTACCTCCTTACTATG

ATCTGCAACCCATGCCAGCCCTCTTTCGTATTGTTCAGGATGACAACCCTCCTATTCCTGATAGTCTTTCTCCAGATATTACGG

ACTTTCTACGACAGTGCTTCAAGAAGGATTCCAGGCAGAGGCCTGATGCAAAGACACTGCTCTCTCACCCTTGGATACGAA

ACTCTAGACGAGCTTTGCAGTCATCACTTCGGCATAGTGGCACCATCAAATATATGAAGGAAGCCACTGCAAGTTCAGAGAA

GGATGATGAAGGTAGTCAAGATGCAGCAGAAAGCCTTTCAGGAGAAAATGTGGGAATATCAAAAACTGACTCGAAAAGCA

AATTACCTCTGGTAGGGGTGTCGAGTTTTAGGTCTGAGAAAGATCAGTCGACACCCAGTGATCTTGGTGAAGAAGGAACAG

ATAATTCAGAAGATGATATTATGTCAGATCAAGTTCCTACACTGTCGATCCATGAGAAATCTTCGGATGCTAAGGGGACACCT

CAAGATGTATCAGACTTTCATGGGAAATCTGAACGTGGTGAAACACCTGAAAATCTTGTAACGGAAACTTCTGAAGCTAGA

AAAAATACTTCAGCAATAAAGCACGTTGGCAAAGAATTATCCATCCCAGTGGACCAGACATCACATAGTTTTGGCCGCAAAG

GTGAAGAACGTGGGATTCGAAAGGCTGTGAAGACTCCATCTAGTGTTAGTGGGAATGAACTGGCTAGGTTTAGTGATCCTCC

TGGGGATGCTTCTTTGCATGATTTATTTCACCCATTGGATAAAGTGTCTGAGGGAAAACCAAATGAAGCCTCAACATCCATGC

CTACGTCAAATGTCAACCAAGGTGATTCTCCTGTTGCAGATGGTGGAAAGAATGATCTGGCTACAAAATTGAGGGCTACAAT
TGCTCAGAAGCAAATGGAGGGGGAAACAGGGCACTCAAATGATGGTGGTGATCTTTTTCGCTTGATGATGGGTGTTTTGAA
AGATGATGTTATTGACATTGATGGCTTGGTATTTGATGAAAAAGTGCCCGCGGAGAATCTTTTTCCTCTGCAGGCAGTCGAGT
TCAGCAGACTGGTCAGCTCCTTGAGGCCAGATGAGTCAGAAGATGCAATAGTATCTTCTTGTCAGAAACTTGTTGCCATGTT
TCGTCAGAGACCTGAGCAGAAAGTAGTATTTGTGACACAGCATGGTTTCCTGCCTCTAATGGATCTACTCGATATTCCTAAAT
CTCGAGTAATATGTGCTGTGCTGCAGCTGATAAACGAAATTATTAAAGATAACACTGACTTCCAGGAAAATGCTTGTCTTGTT
GGCCTTATTCCTGTTGTAATGAGTTTTGCTGGTCCTGAGAGGGATCGTTCTCGCGAAATTCGTAAAGAAGCAGCTTACTTCTT
GCAGCAGCTTTGTCAGTCAAGCCCCTTGACGTTGCAAATGTTCATAGCTTGCCGTGGAATACCCGTTTTGGTGGGATTTCTTG
AAGCAGATTATGCCAAATACAGGGAGATGGTTCACTTGGCTATTGATGGGATGTGGCAGGTATTTAAACTCAAAAGATCCAC
CCCAAGAAATGATTTCTGCCGTATAGCTGCAAAGAATGGAATTCTTCTGAGGCTAATCAACACTCTTTATAGCTTGAATGAGG
CAACCCGGCTGGCTTCTATATCAGGGGGCCTGGATGGTCAAGCTCCACGAGTGCGCTCTGGTCAACTTGACCCTAACAATCC
TATTTTTGGTCAAAATGAGACTTCTTCACTCAGTATGATTGATCAGCCTGATGTATTGAAAACTAGGCATGGGGGTGGTGAAG
AGCCTTCTCATGCTTCAACCTCAAATTCTCAAAGATCAGATGTCCATCAACCAGATGCTTTGCACCCAGATGGTGATAAGCCT
AGAGTAAGCAGTGTTGCACCAGATGCCTCAACCTCTGGCACAGAAGATGTTAGACAACAGCATCGAATATCTCTTTCCGCCA
ATAGGACATCGACAGATAAGCTTCAGAAGCTGGCAGAGGGCGCCTCTAATGGTTTTCCTGTTACTCAGACAGAACAAGTTCG
ACCTTTGCTTAGCTTGTTGGATAAAGAACCCCCTTCAAGACATTATTCTGGTCAACTGGATTATGTTAAGCATATCACTGGCAT
AGAGAGACATGAAAGTAGACTTCCTCTTTTGCATGGATCAAATGAAAAGAAAAACAACGGAGACCTAGATTTTCTGATGGC
TGAATTTGCAGAGGTCTCTGGACGTGGAAAGGAAAATGGAAGTCTTGATACTACGACTAGATATCCCAGTAAAACAATGACA
AAGAAGGTTCTGGCTATTGAAGGAGTTGCTTCAACATCTGGGATTGCATCTCAGACAGCATCTGGAGTACTGTCAGGTTCGG
GTGTTCTAAACGCTAGACCTGGAAGTGCCACATCATCTGGTTTACTTGCCCATATGGTCTCTACGCTGAGTGCAGATGTTGCA
AGGGAATACTTGGAGAAAGTTGCTGACCTGCTTCTTGAATTTGCTCGAGCTGATACGACAGTAAAGTCATATATGTGCAGCC
AAAGCTTACTCAGTCGTCTTTTTCAGATGTTCAACCGTGTAGAACCTCCTATTCTGTTAAAGATACTGGAGTGCACAAATCAT
TTATCAACTGATCCAAATTGCTTGGAGAATCTGCAGCGTGCAGATGCAATTAAGCATTTGATCCCCAACCTTGAGCTTAAGGA
TGGGCATCTTGTTTATCAGATCCATCATGAGGTGCTTAGTGCACTATTCAACCTGTGCAAGATAAACAAGAGGAGGCAGGAA
CAAGCGGCTGAAAACGGAATCATTCCGCATCTGATGCTTTTCATTATGTCAGATTCTCCTCTGAAACAGTATGCATTGCCACT
ACTCTGCGATATGGCTCATGCATCTCGAAATTCAAGAGAACAGTTAAGGGCCCACGGTGGTCTTGATGTTTACCTGAGTTTAC
TCGATGATGAATACTGGTCCGTGATAGCCTTAGATTCAATCGCTGTTTGCTTGGCGCAAGACAATGACAACCGCAAGGTAGA
GCAGGCATTGCTCAAGCAAGATGCAATTCAGAAATTAGTTGACTTCTTCCAGAGCTGCCCAGAGAGACACTTCGTGCACATA
TTGGAGCCATTCTTAAAGATTATCACGAAATCATATCGGATCAATAAGACATTAGCTGTAAATGGATTGACTCCACTGCTTATT
TCAAGGCTAGACCATCAAGATGCGATTGCTCGACTTAACCTCCTAAAACTCATCAAGGCTGTTTATGAACATCATCCACGGCC
GAAACAGCTGATCGTAGAGAACGACCTTCCTCAAAAACTGCAGAATCTAATAGAAGAACGACGAGATGGACAACGTTCAG
GAGGCCAAGTTCTGGTGAAACAAATGGCAACATCTCTCCTCAAAGCACTTCACATCAACACAATATTGTGA

>AtMEKK8 (AT4G08500)

ATGGACAGAATTCTAGCTCGTATGAAGAAATCAACTGGACGAAGAGGAGGAGATAAGAATATTACTCCGGTACGGCGGTTAG
AGCGTCGCGATGCGGCGAGGAATATCAATTACGACGCAGCTTCATGTTCTAGTTCGTCAGCTGAAGATCTTTCCGTTTCGACT
TCTTCGTTGATGACTCGCTCTTTGGAGTTTCCGGAGCCTACTAGTTTCCGAATCGGTGGTGGTGTTGGAGAGATGGATCGGAT
TTATCGGTCTCTTGGTGTTTCTGGTCCTGATGATTTGGCTATTTCTTTTGATGCTTGGGAAGCTTGTAAGAAACGTTCTTCTTC
AGATGTTGTTAATAGGTTTAAGTCTTTTGATCTTGATAAGGTTCGTGATCAGGATTTGAGTGAAGAAGGTCCTAGTGGTGTTG
TTGTTGGTTCTGATTCAATGAATCATAAGGTTCAGGGTCAGGATTTGAGTGAAGCAGGTCCTAGTGGTGGAATTGTTACTGA
GTTGAGTGAGATTGGGAATTTAATCACTCCTGTGGATAGGTTAGTAGCTGATGGTGTGGTAGAGAATAGGCGTGTTATGGAA
AGAACACCAACTATTGTGAAGTCGAAAGGGTATCTTGTACCAAATAATGTAGTGGCTGTTGGTGTTGGTGTTGGTGGTGGTA
TTAAGGGGCTAAGACCACCAGTACTTAAGCCTCCTCCGGCTATGAAACGACCCCCTATTGATCATCGGGGATCGTCTTGGGAT
TTCCTGACGCATTTCGCTCCAAGTGAAACAGTTAAGCGGCCGAGTTCCTCTTCTTCTTCTTCCGAGGATGGATGCGATGAAG
AGGAAGGCAAGGAAGAGGAAGCGGAAGCGGAAGAGATGGGAGCTAGGTTTATCCAGTTGGGGGATACGGCTGACGAGAC

GTGCTCATTCACTACAAATGAGGGTGACTCCTCAAGCACAGTATCCAATACTTCGCCTATCTATCCAGATGGAGGAGCTATCA
TAACGTCTTGGCAAAAGGGTCAACTTTTGGGACGAGGATCATTTGGTTCTGTGTATGAAGGCATTTCTGGAGATGGGGACTT
CTTTGCTGTCAAGGAAGTTTCACTTCTTGATCAGGGAAGTCAGGCACAAGAATGCATACAACAACTTGAGGGGGAGATTAA
ACTACTTAGTCAGCTTCAGCATCAGAATATTGTGAGATATCGTGGCACAGCCAAGGATGGGTCAAACTTGTACATTTTTCTTG
AGCTTGTAACCCAAGGGTCCCTTCTAAAACTCTACCAAAGATACCAGCTTCGGGACTCTGTAGTCTCCTTGTACACTAGACA
GATTCTTGACGGTTTGAAATATCTCCACGATAAAGGTTTTATTCACAGGGACATCAAATGTGCAAATATATTGGTGGACGCTAA
TGGCGCCGTCAAACTTGCAGATTTTGGATTGGCAAAGGTTTCAAAGTTTAACGACATTAAGTCCTGCAAGGGAACTCCATTT
TGGATGGCTCCAGAGGTTATTAACCGAAAGGATAGTGATGGCTATGGAAGTCCAGCTGATATATGGAGCCTCGGGTGCACTG
TGCTGGAAATGTGTACTGGTCAGATCCCCTACTCTGATCTCGAACCCGTTCAAGCCCTGTTTAGGATCGGAAGGGGTACTCTT
CCGGAAGTACCTGATACGTTATCACTAGATGCTCGGCTTTTCATACTTAAGTGTCTTAAAGTGAACCCGGAAGAGCGGCCAA
CTGCAGCTGAACTGCTGAACCATCCATTTGTGAGAAGACCCTTACCATCCGTGGGTTCAGGAGGCTCGGGATCAGCATCTCC
CCTTCTCCGTAGATGA

>AtMEKK9 (AT4G08480)

ATGAAGAAGTCGTCGGATAAGTCACCGGTGCGGCAACATGACACGGCGACGCAAATCAACTCTGACGCAGTGTCTTCTTCT
ACTTCCTTCACTGATTCCGACTCTACTTGTTCCTTTTTGACTCCCTCCATGGAGTTCCCGGATCGAATCAGTTTCCGGAGAATC
GATTTTAGTGAAGCAGCTCCTACTGGTGTCGTTCTTCCTTCTACTTCCAGTGAGTTAACTCGTTCCAATTCCAGTGAGAATAA
GATTCCCAATGAAGATATTTCCGTTTCAACTTCTTCTCGCTATTTGGTGTTTGATAAAATTCTAGCTCTTATGAAGAAATCACC
AGGACGAAGAGGAGATAAGACTAGTCCGGCGCGGCGGTTAGACCGTAGTGATGCGGTGAGGAGGAATATCGATTATGACGC
AGGTGAAGATTCTTCTTCGTTGTTGATAACTCGTTCTTTGGATTTCCCGAATCGTACTAGTTTTCGAGTCGATGGAGTTGATGA
TGGAGAAATAGATCGGATTTATCAGTATATTGGTGTTTCTGGTCCTGAAGATTTTGCTATTTCTTCTGATGCTTGGAAAGCTCG
TATGGAGCATGAGCGTTCTTCTTCAGATGTTGTTAATAAGTTAAAGTCTCTTGATCTTGATAGTCGTGAAGCAGGTCCTAGTG
GTGGTGTTGTTGCTTCTAGTTCAATGAATCATAAGTTTCAGGGTCATGATTTGAGTGAAGCAGGTTCTATTGGTGTTGTTGTT
GCTTCTAATTTTACGCTTTCTGAGTCGAATAAGATTGAGAACTTAAATAGTTTGAGGGATAAGGAAATTGTTGATGGTGATAT
GGTAGAGAATAGGTGCGGTATTGAGCGAAAACCGACTATTTTGGTGAAGTCGAGAGGGTATCTTGTTCACAATGATGATGTG
GGTGTTGGTGGTGGAATTAAGGGTGTAAGACCACCAGTACTAAACGTTCCTCGTGCGGATAAGGAAGTTGTTGATGGTGGTA
CGGTGGAGAGCAAGAGTGGTATTGAATGGAAACCAACTATTTTGGTGAAGTCAAAAGGGTATCTTGTTTCCAATGATGGTGG
AATAAAGGGGGTAACATCACCGGTACTAAATCTTCGCCCGACAGATAAGGAAGTTGTTGATAGTGGTACGGTAGAGAATAGA
CGTGGTATTAAGGGCGTAAGACCATCGGTACTTAAGCCTCCTCCGGTGATGAAACTACCTCCAGTTGATCTTCCGGGATCATC
TTGGGATATCCTTACTCATTTTGCTCCTGATAGTGAAATAGTTAGGCGGCCGAGTTCCTCTTCTTCTTCCGAAAATGGTTGTGA
TGAAGAAGAAGCCGAGGATGACAAAGTGGAAAAGGAAGAGACGGGAGATATGTTTATCCAGTTGGAGGATACGACTGATG
AGGCATGCTCATTCACTACAAACGAGGGTGACTCCTCAAGTACAGTATCCAATACCTCACCGATTTGTGTCTCTGGGGGATCT
ATCAATACTTCTTGGCAAAAGGGTCAACTTCTGCGACAAGGATCGTTTGGCTCTGTGTATGAAGCCATTTCAGAAGATGGGG
ACTTCTTTGCTGTCAAGGAAGTTTCACTTCTTGATCAGGGAAGTCAGGCACAAGAATGCATACAACAACTTGAGGGGGAAA
TTGCACTACTTAGTCAGCTTGAGCATCAGAATATTCTGCGATATCGTGGCACAGACAAGGACGGGTCAAATTTGTATATTTTT
CTTGAGCTTGTAACACAAGGATCCCTTCTAGAACTCTACCGAAGATACCAGATTCGGGACTCGCTAATCTCCTTGTACACAA
AACAAATCCTTGACGGTTTGAAATATCTTCACCATAAAGGTTTCATTCACAGGGACATTAAATGTGCAACTATATTGGTGGAC
GCCAATGGCACCGTCAAACTTGCAGATTTTGGATTGGCAAAGGTGTCAAAGTTAAACGACATTAAGTCCCGCAAGGAAACT
CTATTTTGGATGGCTCCAGAGGTTATTAACCGGAAGGATAATGATGGTTACAGAAGTCCAGCTGATATATGGAGCCTCGGGTG
CACTGTGCTGGAAATGTGTACTGGTCAGATCCCATACTCTGATCTAGAACCCGTTGAAGCCCTGTTTAGGATCAGAAGGGGT
ACGCTTCCGGAAGTACCTGATACTTTATCACTAGACGCTCGGCATTTCATACTTAAGTGTCTCAAATTGAACCCGGAAGAGCG
TCCAACTGCAACTGAGCTACTGAACCATCCATTTGTGAGAAGGCCCTTACCATCCTCAGGCTCAGGATCAACATCTCCGCTA
ATCCGTAGATGA

>AtMEKK10 (AT4G08470)

ATGGACGTAACTGCTATCTTCGCCGGTGATATTTTGGTGCAATCCAGAGAGTATCTTATCCCTAATGATGTTGTGGATGTTGAT

GGTGGTATTAAAGCGGTAAGACCACCAATAATTCAGCCCCCTCCGGGAAGAAAACTACCTCTTATTGACTTTCCAGGATCATC
GTGGGATTTCCTTACGTATTTTGCTCCAAGTAAAACAGTTAAGCGGCAGAGTTCCTCCTCTTCTGATAATACCTCTGATAAAG
AGGAAGTTGAAACGGAAGAGACGCGAGGTATGTTTGTCCAGTTGGGGGATACGGCTCATGAGGCATGCCCATTCGCTACAA
ACGAGGCTGACTCCTCCAGTACAGTATCCATTATTTCGCCGAGTTATGCCTCTAGAGGTTCTATCGTCCCTTCTTGGCTGAAG
AGGAAATTTCTGGGACGAGTATCATTAGGTTTTGTGTATGAAGGCAGTTCAGGTTCCTCCGTTGGTTCCGAGTCGACTTGTTC
CTTGATGACGCCTTCTCTGGAGTTCCCGGATCGCATCAGTTTCCGGAAAAAGGATTTCAGTGAAAAAGGTCCTAGTCGACAC
GTTTGGGAAAAGCGTAAATTGACGCGTGCCAAGTTGATTGAAAATTTTTGCAATCCGGAGGACATTGAACCAGTCACATCTT
GGCTGAAGGGTCAACTTCTAGGAGAAGAATCATTTGCCTCTGTGTATGAAGCCATTTCAGATTCCTCTGTTGGTTCCGAGTC
GACTTGTTCCTTGATGACGCCTTCTATGGAGTTCCCGGATCGCATCAGTTTCCGAAAAAGGGATTTCAGCGAAGAAGGTCCT
AGTGGACGTGTTAAGGAAAAGCGTAAATTGATGCGTAACAAGTTGATTGAGAATTTTCGTAAGCCTGAGGATATCACGTCTT
GGCTGAAGGGTCAACTTCTAGGACGAGGATCATATGCCTCTGTGTATGAAGCCATTTCAGAAGATGGGGACTTCTTTGCTGT
CAAGGAAGTTTCACTTCTTGATAAGGGAATTCAGGCACAAGAATGCATTCAACAACTTGAGGGGGAGATTGCACTACTTAGT
CAGCTTCAGCATCAGAATATTGTGCGATATCGTGGCACAGCCAAGGACGTGTCAAAATTGTACATCTTTCTTGAGCTTGTAAC
CCAAGGATCCGTTCAAAAACTCTACGAAAGATACCAACTTAGCTACACAGTAGTCTCCTTGTATACAAGACAAATTCTTGCT
GGGTTGAATTATCTCCACGATAAAGGCTTTGTTCACAGGGACATTAAATGTGCAAATATGTTGGTGGACGCTAATGGCACCGT
CAAACTTGCAGATTTTGGATTGGCAGAGGCGTCAAAGTTCAACGACATTATGTCTTGCAAGGGAACATTATTTTGGATGGCT
CCGGAGGTTATTAACCGAAAGGATAGTGATGGCAATGGAAGTCCAGCTGATATATGGAGCCTTGGGTGCACTGTGCTGGAAA
TGTGTACTGGTCAGATCCCCTACTCCGATCTAAAACCCATTCAAGCCGCGTTTAAGATTGGAAGGGGTACGCTTCCAGACGT
ACCTGATACTTTATCACTAGACGCTCGGCATTTCATACTTACGTGTCTCAAAGTGAACCCGGAAGAGCGGCCAACTGCAGCT
GAACTGCTGCACCATCCATTTGTTATAAATCTTTGA

>AtMEKK11 (AT4G12020)

ATGTCGGAGAAGGAAGAACTTCCGTTGACATTGACGTCCATCGGAGCGGCCACCGCGACTAGTGATTATCATCAGAGAGTA
GGAAGTTCCGGTGAAGGGATTAGTAGCTCGAGTAGTGATGTTGACCCGAGGTTCATGCAGAATAGCCCCACGGGTTTGATGA
TTTCCCAATCGTCGTCGATGTGCACCGTACCGCCTGGCATGGCAGCAACACCACCAATAAGCTCAGGTTCCGGTTTATCTCA
GCAGCTTAATAATTCTTCTAGTTCCAAGTTATGTCAAGTGGAAGGATGTCAAAAAGGAGCAAGAGATGCATCTGGTCGTTGC
ATTTCCCATGGCGGTGGACGTAGATGCCAGAAACCTGATTGCCAGAAGGGAGCTGAAGGTAAAACAGTGTACTGTAAAGCC
CACGGAGGTGGTCGCAGATGTGAATATCTTGGATGCACCAAAGGCGCAGAAGGCAGTACTGATTTTTGTATAGCTCATGGAG
GTGGTCGAAGATGCAACCATGAAGATTGCACACGATCTGCTTGGGGAAGAACAGAATTCTGTGTCAAGCACGGTGGAGGA
GCGAGATGCAAAACATACGGCTGCGGAAAAAGCGCTAGTGGTCCTTTGCCATTCTGCCGAGCCCATGGTGGTGGTAAAAAA
TGCAGCCATGAAGATTGCACAGGATTTGCTAGGGGAAGATCAGGACTCTGTCTCATGCACGGTGGGGGAAAGAGATGCCAA
AGAGAGAACTGCACTAAAAGCGCTGAAGGTCTTTCGGGACTCTGCATATCCCATGGTGGTGGTCGGCGATGTCAATCTATTG
GATGCACAAAAGGAGCGAAAGGGAGCAAAATGTTCTGCAAAGCATGCATAACTAAAAGGCCTCTAACGATTGATGGAGGA
GGAAATATGGGAGGGGTAACAACAGGTGATGCCTTGAACTATCTCAAAGCTGTGAAGGACAAGTTTGAAGACAGTGAGAAA
TATGACACTTTCCTTGAAGTCTTGAATGACTGTAAACATCAGGGAGTTGACACTAGTGGCGTCATAGCCAGATTAAAAGATTT
GTTCAAGGGCCATGACGACTTACTTTTGGGTTTTAATACCTACTTGTCAAAGGAGTACCAAATAACCATTCTGCCCGAGGATG
ATTTCCCTATCGATTTTCTTGACAAGGTTGAGGGACCTTATGAAATGACATATCAGCAAGCTCAAACAGTTCAAGCCAATGCC
AATATGCAACCTCAAACTGAGTACCCTTCTTCCTCTGCGGTTCAATCATTTTCATCGGGTCAACCTCAGATCCCCACCTCAGC
TCCGGATTCTTCACTACTAGCTAAAAGTAATACCTCAGGTATAACTATCATCGAGCACATGTCACAACAGCCTCTAAATGTTG
ACAAACAAGTTAATGATGGCTATAACTGGCAAAAGTATGGGCAAAAGAAAGTTAAAGGCAGCAAGTTTCCTCTAAGCTATTA
CAAGTGCACATATCTAGGATGTCCTTCCAAGAGGAAGGTTGAGAGATCTCTTGATGGACAAGTAGCAGAAATCGTCTACAAA
GATCGACACAATCACGAACCTCCTAACCAAGGAAAAGATGGTAGCACCACATATCTAAGTGGGAGTTCGACACACATCAATT
GCATGAGCTCTGAATTGACAGCATCACAGTTTAGCTCCAACAAGACTAAGATAGAGCAACAGGAAGCAGCAAGTCTAGCTA
CGACAATAGAGTACATGTCTGAGGCAAGTGACAATGAAGAAGACAGTAATGGAGAAACTAGTGAGGGAGAGAAAGATGAA
GACGAGCCTGAACCAAAGAGAAGAATTACAGAAGTTCAGGTTTCGGAACTAGCTGATGCTTCAGATAGAACCGTGAGAGA

GCCTAGGGTTATTTTCCAAACAACGAGTGAAGTTGATAATTTAGATGATGGATATAGGTGGCGGAAATATGGACAGAAAGTT
GTTAAAGGGAATCCTTATCCAAGGTTTTCCTCCTCTAAAGATTATGATGTCGTAATCAGATACGGAAGAGCAGATATAAGCAA
TGAGGATTTCATTAGCCATCTTCGTGCTTCCCTCTGCCGGAGAGGGATTTCTGTCTATGAAAAATTTAATGAAGTGGATGCAC
TTCCAAAATGTAGGGTTTTGATTATAGTATTAACAAGCACATATGTCCCTTCGAACCTCTTAAACATTCTTGAACACCAACATA
CAGAGGATCGAGTGGTTTATCCAATTTTCTACAGACTATCACCATATGATTTTGTCTGTAACAGCAAGAATTATGAGAGATTTT
ATCTCCAAGATGAGCCAAAAAAATGGCAAGCTGCTTTGAAGGAAATAACTCAGATGCCTGGCTACACATTGACAGATAAGTC
TGAATCTGAACTTATAGATGAGATTGTAAGAGATGCTTTAAAGGTGCTATGTTCTGCTGATAAGGTGAACATGATTGGGATGG
ATATGCAAGTAGAGGAGATTTTGTCACTGCTATGCATTGAGTCCCTTGATGTTCGCAGCATTGGTATATGGGGTACAGTTGGTA
TAGGAAAAACAACCATTGCTGAAGAGATCTTTCGCAAAATCTCTGTCCAATATGAGACCTGTGTCGTCCTTAAGGACCTCCA
CAAAGAAGTTGAGGTAAAAGGTCACGATGCTGTGAGAGAGAATTTTCTGTCTGAAGTTTTAGAGGTAGAACCTCATGTTATC
CGGATATCTGACATTAAAACAAGCTTCTTGAGAAGTCGGCTTCAGCGTAAAAGGATCCTTGTTATTCTTGACGATGTGAATGA
TTACAGAGATGTTGACACCTTTTTGGGGACGCTTAACTATTTTGGTCCAGGAAGCAGAATAATCATGACCTCTAGAAATAGAC
GTGTTTTCGTACTATGTAAAATCGATCATGTCTATGAGGTTAAGCCATTAGATATTCCTAAGTCTCTACTACTTCTTGATCGTGG
GACATGTCAAATTGTTTTGTCACCTGAGGTTTACAAGACATTGTCACTTGAGCTGGTCAAATTTTCAAATGGAAATCCCCAG
GTTCTTCAGTTCTTGAGCAGTATTGACAGAGAATGGAATAAGTTATCACAAGAAGTTAAGACAACATCTCCCATTTACATCCC
AGGTATATTTGAAAAGAGCTGTTGTGGGCTTGATGACAACGAGAGGGGTATATTTTTGGACATTGCATGTTTCTTTAATAGGA
TTGATAAAGACAATGTCGCAATGTTGCTGGATGGTTGTGGTTTCTCTGCACATGTCGGATTTAGAGGCCTTGTTGACAAATCA
CTGTTGACAATATCACAACACAACTTGGTGGACATGCTCAGTTTTATCCAGGCAACTGGTCGAGAAATTGTTCGCCAAGAAT
CAGCTGACAGACCAGGAGACCGCAGCAGGTTGTGGAATGCCGACTATATCAGACACGTATTCATAAATGACACTGGCACATC
AGCTATTGAGGGCATTTTCCTAGACATGTTGAATCTTAAATTTGATGCAAATCCCAACGTGTTCGAGAAAATGTGTAACCTTA
GACTGTTGAAATTGTATTGCTCCAAAGCGGAAGAGAAGCATGGAGTATCTTTTCCACAAGGTCTTGAATATTTGCCGAGCAA
GCTAAGGCTTCTCCATTGGGAATATTATCCTCTAAGTTCTTTGCCGAAAAGTTTTAATCCAGAGAACCTTGTCGAGCTTAACT
TGCCAAGTAGCTGTGCAAAGAAACTTTGGAAAGGAAAAAAGGCAAGGTTTTGTACAACCAATTCAAGTCTGGAAAAGCTT
AAAAAGATGAGACTTAGCTACTCCGACCAGTTAACTAAAATCCCAAGACTTTCAAGCGCAACAAATCTTGAGCATATTGATC
TTGAAGGTTGCAACAGTTTGTTGAGCCTTAGCCAGTCCATTTCTTATCTTAAGAAGCTTGTTTTTCTGAATTTAAAGGGCTGC
TCGAAGCTGGAGAATATTCCATCTATGGTTGATTTAGAATCGCTTGAGGTTCTAAATCTTTCGGGTTGTTCAAAGCTAGGGAA
CTTCCCGGAGATCTCACCAAATGTGAAAGAACTGTACATGGGTGGGACTATGATACAAGAAATCCCGTCATCGATTAAGAAC
TTGGTATTGCTTGAGAAACTGGACCTGGAAAACAGTAGACATCTCAAGAATCTTCCAACAAGCATCTACAAGTTGAAGCATC
TTGAAACTCTAAATCTTTCAGGCTGCATAAGCCTGGAGCGATTTCCAGACTCGTCGAGAAGGATGAAATGCTTAAGGTTTTT
GGATTTAAGCAGGACAGACATTAAAGAGCTGCCCTCTTCCATATCGTATCTGACTGCTCTTGACGAACTATTATTCGTAGACT
CCAGGAGAAACTCGCCAGTTGTAACCAATCCCAATGCCAATTCAACTGAGTTGATGCCTTCTGAGTCAAGTAAGCTTGAGAT
CTTAGGTACTCCGGCAGATAACGAAGTAGTTGTTGGTGGTACGGTAGAGAAAACCCGTGGTATTGAACGAACGCCGACTATT
TTGGTGAAGTCGAGAGAGTATCTAATTCCCGATGATGTTGTGGCGGTTGGTGGTGATATTAAGGGGCTAAGACCACCAGTAC
TTCAGCTCCAACCAGCAATGAAACTATCTCATATTCCTCGAGGATCAACTTGGGATTTCGTTACGCATTTCGCTCCACCTGAA
ACAGTTGCGCCGCCGAGTTCCTCTTCAGAAGCCAGGGAAGAGGAAGTGGAAACGGAAGAGACGGGAGCTATGTTTATCCC
ATTGGGGGATAAGGAGACATGCTCATTCACTGTAAACAAGGGTGACTCCTCAAGGACAATATCTAATACGTCGCCGATTTATG
CCTCCGAAGGATCTTTCATCACGTGTTGGCAGAAGGGTCAACTTCTGGGACGAGGATCATTAGGGTCCGTATATGAAGGCAT
TTCAGCAGACGGGGACTTCTTTGCTTTCAAGGAAGTTTCACTACTTGATCAGGGAAGTCAGGCACATGAATGGATACAACA
AGTCGAGGGGGGGATTGCGCTACTTAGTCAGCTTCAGCATCAGAATATCGTGCGATATCGTGGCACAACTAAGGACGAGTCG
AATTTGTACATTTTTCTTGAACTTGTAACCCAAGGGTCCCTTCGAAAACTCTACCAAAGAAACCAGCTTGGGGACTCTGTAG
TCTCCTTATACACAAGACAGATTCTTGATGGATTGAAATATCTCCACGATAAAGGTTTTATACACAGGAACATTAAATGTGCA
AATGTATTGGTGGACGCTAATGGAACAGTTAAACTTGCAGATTTTGGATTGGCTAAGGTAATGTCCCTCTGGCGAACTCCGTA
TTGGAATTGGATGGCTCCAGAGGTTATTCTTAACCCGAAGGATTATGATGGTTATGGAACTCCAGCTGATATATGGAGCCTTG
GGTGTACTGTGCTAGAAATGTTGACTGGTCAGATTCCCTACTCCGATCTGGAAATCGGTACAGCCTTGTATAACATTGGAACG

GGTAAGCTTCCGAAAATACCTGATATTCTATCGCTAGACGCCCGGGATTTCATACTTACGTGTCTCAAAGTGAACCCGGAAGA
GCGGCCAACTGCAGCTGAGCTGCTTAACCATCCATTTGTGAATATGCCATTACCATCCTCGGGCTCAGGTTCAGTATCTTCGC
TCCTCCGTGGATGA

>AtMEKK12 (AT3G06030)

ATGCAGGATATTCTCGGATCGGTTCGCCGATCCTTGGTTTTCCGGTCGTCTTTGGCCGGAGACGATGGTACTAGCGGCGGAG
GTCTTAGCGGATTCGTCGGGAAGATTAACTCTAGTATCCGTAGCTCTCGAATTGGGCTCTTTTCTAAGCCGCCTCCAGGCCTT
CCTGCTCCTAGAAAAGAAGAAGCGCCGTCGATTCGGTGGAGGAAAGGGGAATTAATCGGTTGCGGTGCTTTTGGAAGAGTT
TACATGGGAATGAACCTCGATTCCGGCGAGCTTCTTGCAATTAAACAGGTTTTAATCGCTCCAAGCAGTGCTTCAAAGGAGA
AGACTCAGGGTCACATCCGAGAGCTTGAGGAAGAAGTACAACTTCTTAAGAATCTTTCACATCCGAACATCGTTAGATACTT
GGGTACTGTAAGAGAGAGTGATTCGTTGAATATTTTGATGGAGTTTGTTCCTGGTGGATCAATATCATCTTTGTTGGAGAAGT
TTGGATCTTTTCCTGAGCCTGTGATTATTATGTACACAAAGCAACTTCTGCTTGGTCTGGAATATCTTCACAACAATGGGATCA
TGCATCGAGATATTAAGGGGGCAAATATTTTGGTCGATAACAAAGGTTGCATCAGACTCGCAGATTTTGGTGCTTCCAAGAA
AGTTGTAGAGCTAGCTACTGTAAATGGTGCCAAATCTATGAAGGGGACGCCTTATTGGATGGCTCCTGAAGTCATTCTCCAGA
CTGGTCATAGCTTCTCTGCTGATATATGGAGTGTTGGGTGCACTGTGATTGAGATGGCTACGGGGAAGCCTCCCTGGAGCGA
GCAGTATCAGCAGTTTGCTGCTGTCCTTCATATTGGTAGAACAAAAGCTCATCCTCCAATTCCAGAAGACCTCTCACCAGAG
GCTAAAGACTTTCTAATGAAATGCTTACACAAAGAACCAAGCTTGAGACTCTCTGCAACCGAATTGCTTCAGCACCCGTTTG
TCACTGGAAAGCGCCAGGAACCTTATCCAGCTTACCGTAATTCTCTTACGGAATGTGGAAACCCAATAACTACTCAAGGAAT
GAATGTTCGGAGTTCAATAAATTCGTTGATCAGGAGGTCGACATGTTCAGGCTTGAAGGATGTCTGTGAACTGGGAAGCTTG
AGGAGTTCCATTATATACCCACAGAAGTCAAATAACTCAGGATTTGGTTGGCGAGATGGAGACTCTGATGACCTTTGTCAGA
CCGATATGGATGATCTCTGCAACATTGAATCAGTCAGAAACAATGTTTTGTCACAGTCCACCGATTTAAACAAGAGTTTTAAT
CCCATGTGTGATTCCACGGATAACTGGTCTTGCAAGTTTGATGAAAGCCCAAAAGTGATGAAAAGCAAATCTAACCTGCTTT
CTTACCAAGCTTCTCAACTCCAAACTGGAGTTCCATGTGATGAGGAAACCAGCTTAACATTTGCTGGTGGCTCTTCCGTTGC
AGAGGATGATTATAAAGGCACAGAGTTGAAAATAAAATCATTTTTGGATGAGAAGGCTCAGGATTTGAAAAGGTTGCAGAC
CCCTCTGCTTGAAGAATTCCACAATGCTATGAATCCAGGAATACCCCAAGGTGCACTTGGAGACACCAATATCTACAATTTAC
CAAACTTACCAAGTATAAGCAAGACACCTAAACGACTTCCGAGTAGACGACTCTCAGCAATCAGTGATGCTATGCCCAGCCC
ACTCAAAAGCTCCAAACGTACACTGAACACAAGCAGAGTGATGCAGTCAGGAACTGAACCAACTCAAGTCAACGAGTCGA
CCAAGAAGGGAGTAAATAATAGCCGTTGTTTCTCAGAGATACGTCGGAAGTGGGAAGAAGAACTCTATGAAGAGCTTGAGA
GGCATCGAGAGAATCTGCGACACGCTGGTGCAGGAGGGAAGACTCCATTATCAGGCCACAAAGGATAG

>AtMEKK13 (AT1G07150)

ATGGAGAAACAGAGCATCAGAAACACTTGTTCTTCTTCTTTAATGTTATCCTCACCATCTTCTTTTTGGGTTCGTGGTGCGTGT
ATCGGCAGAGGTTGTTTCGGTGCAGTTAGTACGGCGATCAGCAAAACCAACGGTGAAGTTTTCGCCGTGAAATCCGTGGAT
CTCGCCACGAGTCTCCCCACTCAATCAGAGTCTCTCGAGAACGAAATATCTGTCTTCCGCTCGCTAAAGCCTCACCCTTACAT
CGTGAAGTTTCTCGGCGACGGAGTCTCGAAAGAAGGAACGACGACGTTTAGGAATCTCTACTTAGAGTATCTCCCAAACGG
TGACGTGGCTAGTCACAGAGCCGGAGGAAAAATCGAAGACGAGACTCTGCTCCAGCGTTACACGGCGTGTCTTGTCTCTGC
TCTCCGCCACGTACACTCTCAAGGATTCGTTCACTGCGACGTCAAGGCGAGGAACATCCTCGTCAGCCAAAGCTCCATGGTC
AAGTTAGCCGATTTTGGGTCGGCGTTCAGAATCCACACACCGAGGGCTCTGATCACGCCACGTGGAAGTCCGCTTTGGATG
GCTCCGGAGGTGATCAGACGAGAGTACCAAGGTCCGGAGAGTGACGTCTGGTCTCTCGGCTGCACGATCATCGAGATGTTC
ACTGGGAAACCCGCTTGGGAAGACCACGGAATTGACTCGCTGAGTCGAATCAGTTTCTCTGACGAGTTACCGGTTTTCCCCT
CGAAGTTGTCGGAAATCGGCCGTGATTTCCTAGAAAAGTGTCTGAAGCGAGACCCGAATCAGAGGTGGAGTTGCGATCAGC
TTTTGCAGCATCCATTTCTCTCTCAGTGTCACAACTCATCTCCGACTGAGTCATCTCCGCGTTGCGTACTTGACTGGGTCAAC
TCGGGGTTTGACTTAGAAGAAGAAGAAGAGGAAGTAGGGAGATCAGAATTCGAAGACGCGGCGAAGGCAATAATTTGTAA
TTTGGCGACGACCGGAGGAGTAATTTGGGAGTCAGATGGTTGGGTGGAAGTTAGATGCCACGCTTCGGAAGAGGAAGGGA
CAACAATGGAATATTCGGGATCCACAAGGGTAGAATCGGAATATAACACATCATCGGATCCAAACGATGACGTGGCTGGTGA
CTCGGCGATAATTGACGTTTCTATGTCTCAGAATTTACCGCCTGGAAACGGGGGATCGGCGGCGGCGCTGCCATATGAATTTG

TGGTGGTTTTACATTTATTAATGGAAATTATGGTTTATACTACATGTATCTTTAGGGAAATTGTTCTCACTATGTATTTGTTGTAT
CAGTATAATCAAAGCAATAAACTCGAAACGTTGTCTTTTAATCACAGCCTCAAGTTTTGTTTGTTTGCACATGTGATTCGGAT
CGGACAAAACTATCTTTTACGCGGTGAGATGAGATCGAGTGTCTTAATTACGAGCCATTGCCTTATATTGATAACTCTCGTTTA
CAGCCAAATAGTTGTATTTTAA

>AtMEKK14 (AT2G30040)

ATGGAGAAACAGAACATTATCTCAAACACTTCTTCTTCTTCGTCTTCTTGGATTCGTGGTTCTTGTGTTGGAAGAGGTTGTTT
TGGTACAGTAAGCAAAGCTTTGAGTAAAATCGACGGTGGACTTTTCGCCGTGAAGTCGATAGATCTCGCCACGTGTCTTCCT
TCTCAAGCAGAGTCGTTAGAGAACGAAATCGTTATTCTACGGTCGATGAAGTCTCATCCAAACATAGTGAGGTTTCTCGGTG
ATGATGTGTCTAAAGAAGGAACGGCGTCGTTTCGAAATCTTCATTTAGAGTATTCACCGGAAGGTGACGTTGCTAACGGTGG
AATCGTTAACGAAACGCTTCTCAGGCGTTACGTGTGGTGTCTTGTCTCTGCTTTGAGTCACGTTCATTCTAACGGAATTGTTC
ACTGCGACGTTAAATCTAAGAACGTTCTTGTTTTTAACGGTGGTAGCTCCGTTAAGCTAGCGGATTTTGGATCGGCGGTTGAG
TTTGAGAAATCTACGATTCATGTTTCGCCACGTGGAAGTCCGCTTTGGATGGCTCCGGAGGTGGTTAGAAGAGAGTACCAAG
GACCGGAGAGTGACGTGTGGTCTTTAGGGTGTACCGTCATCGAGATGCTCACCGGAAAACCAGCTTGGGAAGATCACGGTT
TCGACTCGCTGAGTCGGATCGGGTTCTCTAACGATTTGCCTTTTATTCCGGTGGGGTTATCGGAACTCGGGAGAGATTTCTTA
GAGAAATGCTTGAAACGTGATCGGAGTCAGCGTTGGAGCTGTGATCAGCTCTTGCAGCATCCGTTTCTGTGTCAAGATCATC
ATGACTCGTTCTTCACTGAGTCGTCTCCGCGTTGTGTTCTTGACTGGGTCAACTCGGAGTTTGACGAAGAAGAAGAGAGCG
ACGAATGGAGACCAGAATCCATGGTTTCGGCGATGGCAAGGATAAGTAAATTAGCTATAACCGGAGGAGCAAATTGGGAATC
TAATGGTTGGACTGAGGTTAGAGACACTTCCGAAGAGTCAGAGGCAAAAAAGGAAGTTCTTGTTTCTCCAAGGGTAGAATT
GGAATCTTACATATCATTGGAGTCGTCAAGCGATGATTCCGTAAGACAACCGAGGAATGAAGAGTCGGCGACGGAGTTGGC
GTCGGCAGTGACGTGTGAAGCAATATTGTTGGTGATGATATTGGTGGTAGAGAATATTCAAATATATGCCACGTTTTACACCA
GTAGTATTATTATGCATATTCTATATTGTTGTAGTTGTTGTTGTTATTATCATTATCAAAACAATAATAAGAAGAATAATTTTTCGA
AATCTACATCTTTCATTCTCAGCCTTAACTTTTTGTTTGGTATTGCATGTGATTCGGATCGGTCTATCTATTAA

>AtMEKK15 (AT5G55090)

ATGGAGGAACAAAACTGGATAAGAGGACCAATCATAGGTCGAGGCTCAACAGCTACTGTCTCACTTGGAATCACAAATTCC
GGTGACTTCTTCGCCGTCAAATCCGCCGAGTTTTCTTCATCGGCGTTTTTGCAGAGAGAACAATCGATTTTGTCGAAGTTGA
GCTCTCCTTACATAGTCAAGTACATTGGTTCTAATGTAACGAAGGAGAACGACAAGTTGATGTATAATCTCTTAATGGAGTAT
GTTTCCGGTGGGAGTCTTCACGATTTGATCAAGAATTCCGGCGGGAAGTTACCGGAGCCGTTGATTAGATCCTACACACGTC
AGATATTGAAAGGTTTGATGTATCTTCATGATCAAGGAATCGTTCATTGCGATGTGAAGAGCCAGAATGTGATGATCGGAGGA
GAGATTGCGAAGATCGTCGATTTGGGTTGTGCTAAAACGGTGGAAGAGAATGAGAACTTGGAATTTTCCGGTACACCGGCG
TTTATGTCACCGGAGGTAGCACGTGGTGAAGAACAGAGTTTTCCAGCTGATGTTTGGGCTTTGGGGTGTACGGTGATAGAGA
TGGCTACAGGGTCAAGTCCTTGGCCGGAGCTAAACGACGTCGTTGCTGCTATTTATAAGATTGGTTTCACCGGCGAGTCGCC
GGTGATTCCGGTTTGGTTATCGGAGAAAGGTCAAGACTTTTTGAGGAAGTGTTTGAGAAAAGATCCGAAACAGAGATGGAC
TGTTGAAGAATTGCTTCAACATCCGTTTCTCGACGAAGAAGACAACGACAGTGACCAAACTGGTAACTGTCTGAATTCTTCA
TCTCCTAGTACTGTGTTGGATCAACGGTTTTGGGATTTATGTGAAACTTCGAGAAGTAGATTCATTAAAGAGGATCACGAAGA
CCCATTTGCAAATTCTACAAATTTCTTATGGGATGACGATTCTTTACCGGGTGATCGGATCAAGAAACTCGCCGGAGACGAGA
GTTCCGGTGAACCAGACTGGGAAACGAACGGTTGGATTGAAGTAAGGGGAGAGATAGAGAAACGTAACGAGGAAGAAGAT
GAGAATTGCGTTGAAGCAACGTCATTGGAGGAGGACGAAGAAGAAGTGGGAGGATTTGAGAATTGGATCTGGGATCAACA
AGACAGCTTGTTCTTGGAATATTCCCCTGAGGACAACATTTATTATTTTTACTCTTATTATAACATCTTTGATGAAGATATAATT
CTATATTATGATCATCTTGAAGATTGTTTTGTACTAAAATTTGATGATAATAATAAGAAGATTTTTTTCTTTAGCCACATTACAAA
TTCTTGTTTTGAGATTACTAATTACAATTGA

>AtMEKK16 (AT4G26890)

ATGGAGATTAATTGGACAAGAGGACCAATCATCGGTCGAGGCTCTACCGCCACTGTCTCTATAGCAATCTCAAGCTCCGGTG
AGCTCTTCGCCGTCAAATCTGCCGACTTATCTTCTTCGTCCTTGTTGCAGAAAGAGCAATCAATCTTGTCTACATTGAGCTCT
CCTCACATGGTCAAATACATAGGCACTGGTTTAACGCGCGAGAGCAACGGATTGGTGTACAATATACTGATGGAATACGTTTC

TGGTGGAAATCTTCACGATTTAATCAAAAATTCCGGCGGTAAGTTGCCGGAGCCGGAGATAAGATCATACACTCGTCAGATT
CTTAACGGTTTGGTGTATCTTCACGAGAGAGGGATTGTTCACTGCGACTTGAAGAGCCATAACGTTCTGGTTGAGGAAAATG
GCGTTTTGAAAATCGCCGACATGGGTTGTGCTAAATCGGTTGACAAGTCGGAATTTTCCGGTACACCGGCGTTTATGGCCCC
AGAAGTTGCTCGTGGTGAAGAACAGAGGTTTCCGGCTGATGTGTGGGCTTTGGGATGTACGATGATCGAGATGATGACTGGA
TCAAGCCCTTGGCCGGAGTTAAACGACGTCGTTGCTGCAATGTATAAGATTGGATTTTCAGGCGAGTCGCCGGCGATTCCCG
CGTGGATATCGGATAAAGCTAAAGACTTTCTGAAGAATTGTTTAAAGGAAGATCAAAAACAGAGATGGACTGTGGAAGAAT
TACTTAAACATCCATTTCTTGATGACGACGAAGAATCTCAGACAAGTGATTGTTTGAAGAACAAGACTTCTTCTCCAAGCAC
TGTGTTGGATCAACGATTTTGGGATTCATGCGAAAGCTCCAAAAGTCACTTAGTTTCGATTGATCATGAAGACCCTTTTGCTG
AATACTCGGAATCTTTGGATTCACCGGCTGATCGGATCGAGAAACTCGCCGGAGATGAGTTTTCAAGCTTACTTGATTGGGAT
ACGGAAGACGATGGCGGATGGATTCAAGTGAGAGGCGAGAAACATAAAGAAACCGAGAAACGCGACGGCGACGAAGACG
TGATTTGCGTTGAAGCAACGTCATCGTCGCAAACGATTGAAGAAGTTGAAGATTGGATTTCGAATCAAGACAGCTTGTTCTC
GGAATATTCCTCTGACGACATCATTAATAATTTCTACTCTAATGTTGCTATTCAAGGAAATTTAATTGCATTTCATTATTGTACTG
ATGAAGATGAAAATGTATCCATAAAGAATATGTTTCGTACTAATAACAAACATTTGTTTTTCAAATTGCAAGCTCATGTTAAAT
TCACTAACCAAGTCTGA

>AtMEKK17 (AT2G32510)

ATGGAATGGACTAGAGGAAGAATCCTAGGCCGAGGCTCTACAGCCACCGTATACGCCGCCGCAGGTCACAATTCAGATGAA
ATCCTCGCCGTTAAATCATCTGAGGTTCACCGCTCAGAGTTCTTGCAAAGAGAAGCCAAGATTCTCTCGTCGTTAAGTTCTC
CTTACGTGATCGGATACAGAGGATCAGAAACCAAGAGAGAGTCAAACGGTGTCGTTATGTATAATCTTTTGATGGAGTACGC
ACCGTATGGAACGTTGACCGATGCGGCGGCGAAAGACGGGGGAAGAGTCGACGAGACTCGGGTAGTGAAGTACACGCGCG
ATATACTAAAAGGATTGGAGTATATACACTCGAAAGGAATCGTGCATTGTGATGTAAAGGGTAGTAACGTGGTTATTTCGGAG
AAAGGCGAGGCCAAGATTGCGGATTTTGGTTGTGCGAAACGGGTTGACCCGGTGTTCGAGTCGCCGGTTATGGGAACGCCG
GCTTTTATGGCTCCAGAGGTGGCGCGTGGAGAGAAACAGGGGAAGGAGAGTGATATATGGGCGGTTGGTTGTACGATGATA
GAGATGGTTACGGGTTCTCCACCGTGGACGAAGGCAGATTCGAGGGAGGATCCGGTTTCGGTTCTTTATCGGGTCGGGTATT
CGAGTGAAACTCCGGAGCTTCCTTGTTTGCTTGCGGAAGAAGCAAAAGATTTTTTGGAAAAGTGTTTGAAGAGGGAAGCA
AATGAAAGATGGACAGCGACACAACTCCTAAACCATCCTTTTTTGACAACTAAACCAGACATTGAACCAGTTCTGGTTCCCG
GTTTGATTTCAAACTCACCGACAAGTGTGACAGATCAAACGTTTTGGAGATCAGTAGAGGAAGAGGAAGAAGAAGAAACA
GAGGAAATACAGAAGGATTCGAGAGATCTTGATCGTTTAAGCTTGTGGGGTTGTTACTCGGAGAGGATCGGACGGCTGAAG
TGTGTTGGTGGTTTGGATGGGACCAGATGTGATATGGAGGGTGGGGATTGGATTATGGTGAGAGCGAGGTGTGAAGGAACA
ATGATTAGTGGGTCACAAAAGGAATTGATTATTAGTGAAAATGTATTGGTGGGGGAATTGTAA

>AtMEKK18 (AT1G05100)

ATGAATTGGACTAGAGGAAAAACTTTAGGCCGTGGCTCAACGGCCACCGTTTCCGCCGCCACTTGTCACGAATCCGGCGAA
ACACTCGCCGTAAAATCCGCCGAGTTTCACCGGTCGGAGTTCTTGCAAAGAGAAGCCAAGATTCTCTCCTCATTAAACTCTC
CGTACGTCATCGGATACAGAGGATGCGAAATTACGAGAGAGCCCTTCCACAACAACGGAGAAGCTACGACTTACAGTCTTC
TTATGGAGTACGCGCCGTACGGAACGTTGACCGACGTAGCGACCAAGAACGGCGGCTTCATTGACGAGGCTCGGGTCGTGA
AGTACACGCGCCAGATACTTCTTGGGTTGGAGTACATTCATAACTCAAAGGGAATTGCGCATTGCGACATTAAGGGAAGCAA
CGTGTTGGTCGGAGAGAACGGAGAAGCCAAGATCGCTGATTTCGGGTGTGCGAAATGGGTTGAACCGGAAATAACCGAAC
CGGTTAGAGGAACGCCGGCGTTCATGGCTCCAGAAGCGGCGCGTGGAGAGAGACAAGGGAAAGAGAGTGATATATGGGCG
GTGGGTTGTACGGTGATAGAGATGGTTACCGGGTCTCAGCCGTGGATTGGGGCGGATTTTACCGACCCGGTTTCGGTTCTTT
ACCGGGTCGGATATTTAGGTGAGTTACCAGAGCTGCCTTGCTCGCTTACGGAACAAGCAAAGGATTTTTTAGGAAAGTGTTT
GAAAAAAGAAGCGACGGAGAGATGGACAGCGAGTCAACTACTAAACCATCCGTTCTTGGTTAATAAAGAACCGGAATTGGT
AACCGGTTTGGTTACGAACTCACCGACAAGTGTGACGGATCAAATGTTCTGGAGATCAGTGGAAGAGGAGGTGTCAGAGG
ACCGTTCAAGCTGGTGGGAATGTCACGAGGATGAGAGAATTGGAGTGCTAAGCTGGATTGGTCATGTTGTGGTGGAGTCCA
CATGGGACTTGGACGGTGAGGATTGGATCACGGTTCGACGGAATTAG

>AtMEKK19 (AT5G67080)

ATGGAGTGGATTCGAGGAGAAACTATCGGATACGGAACTTTTTCTACAGTAAGTCTAGCGACGCGGTCTAATAACGATTCCG
GCGAGTTTCCTCCGTTAATGGCTGTGAAATCTGCAGACTCATACGGCGCTGCTTCTCTGGCAAACGAGAAATCAGTTCTAGA
TAATCTCGGAGACGATTGCAACGAGATCGTACGGTGTTTCGGCGAGGATCGGACGGTCGAAAACGGTGAAGAGATGCATAA
TTTGTTCTTGGAATACGCTTCTAGAGGAAGCTTAGAGAGTTATCTTAAGAAATTAGCCGGTGAAGGTGTACCGGAATCCACC
GTGCGTCGCCACACAGGATCGGTGCTTAGAGGTCTACGACACATCCACGCTAACGGATTCGCTCACTGTGATTTAAAACTCG
GGAATATTCTGTTGTTCGGTGACGGCGCCGTTAAGATTGCGGATTTTGGATTGGCGAAGAGAATTGGGGATTTAACGGCGTT
AAATTACGGTGTGCAGATTAGAGGTACGCCGTTGTACATGGCGCCGGAATCTGTTAACGATAACGAGTACGGATCAGAAGGT
GACGTGTGGGCTTTAGGATGCGTAGTAGTTGAGATGTTTAGTGGTAAAACGGCATGGAGTTTAAAAGAAGGGTCGAACTTCA
TGTCGTTGTTGTTACGCATCGGTGTTGGTGACGAGGTTCCGATGATTCCCGAGGAGTTGTCGGAACAAGGAAGAGATTTTTT
GTCAAAGTGTTTCGTTAAAGATCCCAAAAAGAGATGGACGGCTGAGATGCTTCTAAACCATCCATTTGTAACCGTCGATGTT
GATCACGACGTTTTAGTCAAAGAAGAAGATTTCGTTGTTAATATGAAAACAGAGGACGTCTCGACATCGCCGAGATGCCCAT
TCGAATTTCCCGATTGGGTTTCGGTTTCTTCCGGTTCACAAACGATCGATTCGCCGGATGAGAGAGTTGCTAGTTTGGTGACT
GATATGATCCCTGATTGGTCTGTTACCAATAGCTGGGTCACCGTACGGTGA

>AtMEKK20 (AT3G50310)

ATGGAGTGGGTTCGAGGAGAAACAATTGGGTTCGGAACCTTCTCTACTGTCAGTACAGCGACAAAGTCTAGAAACTCCGGC
GACTTTCCTGCACTTATCGCTGTGAAGTCGACGGATGCTTATGGCGCCGCTTCACTCTCCAACGAGAAATCGGTGTTGGATTC
ACTCGGTGATTGTCCTGAGATCATACGGTGTTACGGCGAGGATTCAACGGTGGAGAACGGAGAAGAGATGCATAACTTGTTA
TTAGAGTACGCTTCGAGAGGAAGCTTAGCGAGTTACATGAAGAAACTTGGCGGTGAGGGTTTACCGGAGTCCACCGTGCGT
CGCCACACAGGATCAGTGCTTCGAGGGTTACGTCACATCCACGCTAAAGGGTTTGCACACTGCGATATAAAACTCGCGAATA
TTCTGTTATTTAACGACGGCTCTGTTAAAATTGCCGATTTCGGTTTGGCGATGAGAGTTGACGGAGATTTAACGGCGTTAAGA
AAAAGTGTGGAGATTAGAGGGACGCCGTTATACATGGCACCGGAATGTGTTAATGATAACGAGTATGGATCAGCGGCTGACG
TGTGGGCTTTAGGATGTGCTGTAGTTGAGATGTTTAGTGGCAAAACGGCATGGAGTGTTAAAGAAGGATCACACTTCATGTC
GCTTTTGATACGTATAGGTGTTGGTGATGAGTTACCGAAGATTCCAGAGATGTTGTCCGAAGAAGGCAAGGATTTTTTGTCG
AAGTGTTTTGTTAAAGATCCAGCGAAAAGATGGACGGCTGAGATGCTTTTGAACCATTCGTTTGTAACCATTGATCTTGAAG
ATGATCACCGAGAAAACTTCGTTGTGAAGGTTAAGGATGAGGATAAAGTATTGATGTCTCCAAAGTGTCCTTTTGAGTTTGAT
GATTGGGATTCTTTTACATTGGATTCAAACCCCTCGTTTGATTCTCCGGTGGAGAGACTTGGGAGTTTGGTTAGTGGTTCGAT
CCCTGATTGGTCTGTCGGTGGGAGTTGGCTTACAGTCCGGTGA

>AtMEKK21 (AT4G36950)

ATGGAGTGGATTCGTAGAGAAACGATTGGTCACGGAAGCTTCTCCACCGTTAGTTTAGCAACTACCTCTGGAAGTTCTTCTA
AAGCGTTTCCATCGTTAATGGCTGTGAAATCTTCCGGAGTTGTTTGCTCCGCCGCGTTACGAAACGAGAGAGATGTTCTTGA
TGATCTCGGTGACTGTTCGGAGATCGTGCGGTGTTTTGGTGAAGGAAGGACGGTGGAGAACGGAGAAGAGATCTATAATCT
GTTTCTTGAGTATGCTTCCGGTGGTAGTTTAGCTGATCGGATTAAAAGCTCCGGTGAAGCGTTGCCGGAGTTTGAAGTGAGG
AGATTCACGAGATCGATTGTTAAAGGATTGTGTCATATTCATGGTAATGGTTTCACTCATTGTGATATAAAGCTTGAGAATGTT
TTGGTTTTTGGTGATGGAGATGTGAAGATATCTGATTTCGGGTTAGCGAAACGGAGGAGTGGTGAGGTTTGTGTTGAGATTA
GAGGAACACCGTTGTATATGGCACCGGAGTCTGTGAATCACGGCGAGTTTGAATCTCCGGCGGATATTTGGGCTTTGGGATG
CTCTGTTGTTGAGATGTCTAGTGGTAAAACGGCATGGTGTTTGGAGGATGGTGTTATGAATAATGTTATGTCTTTATTGGTTCG
TATTGGTTCCGGCGATGAAGTTCCGAGGATTCCGGTGGAATTGTCGGAGGAAGGTAAAGATTTCGTGAGCAAGTGTTTTGTT
AAAAACGCCGCGGAGAGATGGACGGCTGAGATGCTTTTGGATCATCCATTCTTAGCCGTTGATGATGAGAGTGGCGAAGAA
GACGAAGCTTGTTCGGTTTCACCGAGGAATCCATTTGATTTTCCCGGTTGGAATTCGGTTCAATCTCCGGTTAATGATTCGGT
TATGTTTGGTTCATTGGTAGGTTCGCCGGAGGAAAGGATTAGTGGTTTGGTGAGTGAGAAGGTACCTGATTGGTCGGTGTCG
TGTGATTGGGTCAACGTCAGGTGA

>AtZIK1 (AT3G51630)

ATGTATATGGAGATTTCTTCTGCTTCCGATGATTCGATCGCTTACGTTGAGACAGATCCTTCTGGTCGCTACGGACGTTTCAGA
GAAGTACTTGGCAAAGGGGCGATGAAGACGGTTTATAAAGCATTTGACCAGGTTTTAGGAATGGAAGTTGCTTGGAATCAA

GTTAAGCTCAATGAGGTTTTCCGATCACCTGAGCCTTTACAACGCCTCTACTCTGAGGTTCATCTCCTCAAGAATCTCAATCA
CGAATCCATTATTCGTTACTGCACGTCTTGGATCGATGTCAACCGCAGAACATTCAACTTCATTACTGAGCTGTTTACATCAG
GCACCCTTAGAGAGTATAGAAGGAAGTATCAAAAGGTTGATATCCGGGCAATCAAGAGCTGGGCACGTCAGATCTTGAATG
GTCTTGCTTATCTCCATGGACATGATCCTCCTGTCATTCACAGAGACCTCAAATGCGATAATATCTTCGTTAATGGACACCTTG
GGCAAGTTAAGATTGGTGACCTGGGCTTAGCTGCAATTCTTCGTGGATCACAGAATGCGCACAGCGTCATAGGAACGCCTGA
ATTTATGGCACCAGAGCTATATGAAGAAGATTACAACGAGCTTGTAGACATTTATTCGTTTGGTATGTGTGTCCTAGAGATGCT
TACCGGTGAGTATCCTTATAGTGAATGCACCAACCCTGCCCAAATATACAAGAAAGTTACATCGGGGAAGTTGCCTGACTCAT
TCCATCTAATCCAACACACGGAGGCCCAACGTTTTGTTGGTAAATGCTTGGAAACTGTGTCAAGAAGATTGCCTGCAAAGG
AGCTCTTAGCGGACCCATTCCTTGCCGCAACTGATGAGAGAGATTTGGCGCCTCTTTTTAGATTGCCGCAACAATTGGCAATC
CAGAATTTGGCTGCAAATGGAACGGTGGTAGAGCATCTGCCGTCAACGACTGATCCAACTAGAACAACAGATATGTCAATAA
CAGGAAAGATGAACTCAGAAGACCACACCATCTTCCTTCAAGTACAGATTCTAGATGGCGATGGTCACATGAGGAACATTCA
GTTCCCGTTCAACATTTTAAGTGACACACCTCTAGAAGTAGCTTTGGAGATGGTGAAGGAGCTTGAGATCACTGATTGGGAT
CCTTTGGAGATCGCTGCAATGATAGAAAATGAGATATCTTTGCTCGTCCCCAACTGGAGGGCCAATGACTCTTCTATCCGGCA
CGAAAGCTTTGGTCATGAAGATGATGAGGACAATGGCGACACAGAGGGAAGAACGCGCCTTTTCTCCTCTGCTTCTTCCTCT
CACGACTCTCCTGTAGCAGTTAGAGAGAACAATGACGATTCTAGTAACGATGTGATCCCAGATATGGATGATGGCAATAGAA
GCTCTAACAGGTTGCTGAATTCTTCAACATACCACTACTCACCTGCCATTGATGATGATCAAAATCAGCAACAACGGAGACG
AGTAAGGCTACAACAGAAAATGAGATCGCTGGTGGACACGAGAACACAGGTGCTACACCGATCGCTCATGGAGTTGATCAA
CAAGCGTCGTGGGCGTGGCTTTGACCCGAACACAAACGAGCTACAACCTCAACCGTCGTCCACTGATTTCATTCGACGATG
TTGA
>AtZIK2 (AT5G58350)
ATGAATATGAATCAAGTTGCAGAGTATGTGGAAACTGATCCAACTGGTCGCTATGGACGTTTTGCAGAAATTCTTGGAAGGG
GAGCGATGAAGACAGTGTACAAAGCAATCGACGAGAAGCTGGGAATAGAAGTAGCATGGAGCCAGGTGAAGCTTAAGGAG
GTTCTGCGTTCCTCTGTTGATCTACAGAGGCTTTACTCCGAGGTTCATCTCCTCAGCACTCTCAACCACAAATCCATCATTCG
TTTCTACACTTCCTGGATCGATGTTCATAACCACACTCTCAACTTCATCACCGAGTTGTTCACTTCAGGGACCCTCCGACAAT
ACAAAAATAAGTACTTGCGGATAGATATCCGAGCAATCAAGTCCTGGGCTCGGCAAATCTTGGAAGGGCTAGTTTATCTCCA
CGAGCACGACCCTCCTGTTATCCATAGAGACCTTAAGTGTGATAATATCTTTGTGAATGGGCATCTTGGACAAGTCAAAATTG
GTGATCTTGGTCTTGCAAGAATGCTGCGGGACTGCCACTCGGCCCATAGCATCATTGGCACTCCTGAGTTCATGGCACCAGA
ATTATATGAGGAGAACTATAATGAACTCATTGACGTATATTCCTTTGGTATGTGCTTTTTGGAGATGATCACCTCTGAGTTCCC
GTATAGTGAGTGCAACCATCCGGCACAGATTTACAAGAAAGTTGTTGGGGGAAAGCTACCAGGAGCATTTTACAGAGTTGG
AGACATCGAGGCACAGAGGTTCATCGGGAAATGCCTCGTGTCTGCCTCAAAGAGAGTATCAGCAAAAGAACTATTGCAAGA
TCCATTTCTTGCCTCCGATGAGTCCTGGATGGTATACACGAGCGGTGCTGGGAATCCAAAGCCCTTCTTGAACGAGAATGAA
ATGGACACACTGAAGTTGGAAGATGATGAGTTAAGGACCGAGATGTCCATCGCTGGGAAGCTGGGCGCTGAAGATAACAAA
ATCGATCTGGAAGTACAAATCGCATATGATAATGGTCTGGCCAATAATGTATTCTTTCCCTTTGACATCATGAATGATACTTCCA
TTGATGTTGCAAAGGAGATGGTAAAAGAACTCGAAATTATAGATTGGGAGCCAGTCGAGATTGCTAAAATGATTGATGGAGC
GATTTCTTCTTTGGTGTCTGATTGGAAGTATGAGGAAGATGATGAAACCCCTCATGATCATCATCGTCATCGTACCGATTCTTT
TCACTCTTCTTCCTCGCACGCGTCATCTTCCCAAGCTTCACTCTCGAACTATATGGCTCGAGGCCTTCAAGATTGGGTACAAG
ATGATTTACACGATGAGACGTATTCTCAGAGTTCTTCCCATTCAGGGTCTTACTCCAACCTCAACTACATAGCTGTTGATGAGT
ACAGCTCCCAATCTCCTGTTATGAGCAGAACTCACAACATGACGAGGTTTTGCCCCGAAGAAAGCTCTCATCTACAGTCAGG
ACAAGCCAACGCGTATGCAGCTTCCAGTTCAACTAACAGAAGCTTGGCTTCAGACAACCGCACACTTACAAGGAACCGATC
ACTTGTAGACGTGCAGAGACAGTTACTGCACCGGTCGCCAGGGGAAGAGGCTAGAAAAAGAAGATTGTTCAAAACTGTTG
GAGACGTAGAAACAGTTGGGTTTCAGTCACCTTATGCGGTTTCACGGAAGCCACCAAGCTCAAGGCGCTAG
>AtZIK3 (AT3G22420)
ATGAATGGTGAAGAAAGCTTTGTAGAAGATTGCTCTGTTTTTGTTGAGATTGATCCTTCTGGAAGATATGGAAGATACGATGA
AATACTTGGCAAAGGAGCTTCAAAGACAGTATACAGAGCATTTGATGAGTATGAAGGTATAGAAGTAGCATGGAACCAAGTA

AAGCTTCGAAATTTCACAAGGAATCCTGAGGAATTAGAGAAGTTTTTCAGAGAGATTCATCTTCTCAAGACTTTGAATCATC

AAAACATTATGAAATTCTACACTTCTTGGGTTGATACCAACAATTTATCAATCAATTTTGTCACTGAACTCTTCACCTCTGGTA

CTCTCAGACAGTATAGGTTGAGACATAGAAGAGTGAATATTAGAGCAGTGAAGCAATGGTGCAAGCAGATTTTAAAAGGGC

TTCTTTATTTACATAGTCGTTCTCCACCAATTATACATAGAGATCTCAAATGTGATAACATTTTCATCAATGGAAACCAAGGTG

AAGTCAAGATCGGTGACCTTGGACTCGCTGCGATTCTTCGTAAATCACATGCCGTTCGTTGCGTTGGTACTTCAAAACCTTC

ACATCATTGGAACTTTATTGCTTTAATTATGTTTTTTACAACTTTGGACCTACCTTTACTATGTTTATGTGTTGTTAAAGGAACC

CCTGAGTTTATGGCTCCAGAAGTGTATGATGAGGAATATAATGAGTTGGTTGATGTATATGCTTTTGGCATGTGTGTGTTGGAG

ATGGTTACTTTTGATTATCCTTACAGTGAATGTACTCACCCGGCACAAATCTACAAGAAAGTTACCTCGGGGAAAAAGCCTG

AAGCTTTTTACTTAGTGAAGGATCCTGAGGTTCGTGAGTTTGTTGAGAAGTGTTTAGCTAACGTGACGTGTAGGCTAACGGC

ATTGGAGCTTTTACAAGACCCTTTTCTACAAGATGATAATATGGATGGATTTGTTATGAGACCTATTGATTACTACAATGGTTAT

GATGAAACTGGTGTGTTCCTTAGACATCCTTTGATTGATGATCCTCTTTACCATGATCAGTTTGAGTCGTCACAGATATGTGAG

ATCGATCTTTTCGCTAACGATGATGAAGATCATGTCGACATTTCGATTAAAGGGAAGAGAAACGGTGATGATGGGATATTCTT

GAGACTTAGAATATCTGATGCTGAAGGTATAGTTTCAATCTTTTTCGATAGTTTTAAAATCATCGGTCTTAAAAACGTAACACG

CTTTCGAGTTTTGGGTAATGTAGGACGGATAAGGAACATTTACTTCCCGTTTGAGACGGCTATTGATACTGCATGGAGTGTAG

CGGTTGAGATGGTGTCAGAGCTCGACATAACGAATCAAGATGTTGCGAAAATCGCGGAGATGATCGATGCAGAGATTGCTGC

ATTGGTGCCTGATTGGAAAAATGATACAGAAAGTTCCCAAAATGTAAACAACAACAAGAACAACAACACTGCAGGATTCTG

TGGAGAGTGTGCTTCAAACGGGTATATACAAGAGACTGTATCATCAGGAGAAAAATCTCATCATAATCATCATGAGTTCGATA

GTTCTGAAGACAAGAGCTGTTCTTCGGTTCACGGTAGGTTTGCGGATATGTGGGGTTTGCGAGAATCATATTCTGATGATGGA

GAAAAACAGAGCTCAAGGAAGGTTAGAAGTGGACGGTGGTCGGAGAATGAGATGAGACGAGAACTGAGATGGCTTAAGG

CAAGGCACAAGATTCAACTTATGAAAATGAGAGGTCAAACGATCTGCGAGACACCGATAGAGATCTCTCTTACACCGGGAA

CTTCAGTTTCGTTACCTCTTCTTTACAGGGCTATATCACTTCCTGTGGATGCCGTGGATATGTGA

>AtZIK4 (AT3G04910)

ATGAACAATCTGAGTTATCTTGAACCAGATTACTCTGAGTTTGTTGAAGTTGATCCTACTGGAAGATATGGAAGATACAATGA

AGTTCTTGGTAAAGGAGCTTCAAAGACTGTTTATAGAGCATTTGATGAATATGAAGGCATAGAAGTAGCATGGAACCAAGTT

AAGCTATATGATTTCCTACAAAGCCCTGAGGATCTTGAGAGGCTTTACTGTGAGATTCATCTTCTCAAGACTCTAAAACATAA

GAACATCATGAAATTCTACACTTCTTGGGTCGATACCGCCAATCGAAATATCAATTTCGTCACTGAATTGTTCACTTCTGGCAC

CTTAAGACAATATAGACTCAGACATAAGAGAGTGAACATAAGAGCTATGAAGCATTGGTGCAGACAAATCTTGAGAGGCTTA

CATTATCTTCACAGCCATGATCCTCCTGTGATCCACAGGGATCTCAAATGTGACAACATTTTCGTTAACGGGAATCAAGGAGA

GGTCAAGATTGGAGATCTTGGCCTTGCTGCTATTTTAAGAAAGTCCCATGCTGCTCACTGCGTCGGGACTCCTGAGTTCATG

GCACCTGAAGTTTACGAAGAAGCATATAACGAATTGGTTGATATATACTCGTTCGGTATGTGTATTTTGGAGATGGTTACGTTT

GATTACCCGTACAGTGAGTGTACTCATCCTGCTCAGATCTACAAGAAAGTTATGTCGGGCAAGAAACCGGATGCATTGTACA

AGGTGAAAGACCCCGAGGTTAAATGTTTCATTGAGAAATGCTTGGCCACCGTATCGCTTAGAGTCTCTGCTCGTGAGTTACT

AGATGACCCTTTTCTCCGTATAGACGATGGTGAGTTTGATTTAAGATCAGTTGATATGGAAGATTCGGTCGGGCCACTCTATA

GGCAGCCGCACCATCTTCCTGACTACTACAATTACCCGTCGAATAGTAGCTCTTTGAATCGTCAGTACTCAAATGGCAACTAC

CCGTCGAATAGTAGCTCATTGAATCGTCAGTACTCAAATGGTTATAATAGTCATCACGAGTATCAGAATGGATGGGCGTATAAT

CCAGCTGAGACAGAGGAGACTCACGGCATCGAGCTCTTTGAATCTCGAAACAATGATGATCAAGAAGAAGAAAAGAAATC

TGGTAATGTTGACATAACCATCAAAGGGAAGAGGAGAGATGATGGTGGCTTGTTCTTGCGTCTTAGGATCGCTGACAAAGAA

GGACGTGTCCGAAACATTTACTTCCCATTTGACATTGAGACGGACACCGCATTGAGCGTTGCAACAGAGATGGTAGCGGAA

CTGGATATGGACGATCATGGAGTCACAAAAATAGCCAACATGATTGACGGTGAGATATCTTCTCTTGTACCTAGTTGGAGACC

GGGACCTGAATTCGAAGAATGTCTTGCGGCTGCGGCGGCGGCAAATGCTGCGAGCATTTGCAACAACTGCGTATCAAACCG

CACCTCAATGGGCTCGGTGATGGATTTCCTGAGAACCAATCCTGGGGCAAATGTGATACAATGTTGCAGAAACGGGTGCGGT

GAGACTCATGGTCGGTTTGAAGAGATCACGATCAGAGAAACCGAGGTTCGTCTTAGAGAGCTATGGAAGCTGCAGCAACAG

CAAGAAAGCCGCGAGCTAAGCTCGATAGATTCAGGCCATAACCATTCCGAAGAAGAGGAGGAAGAAGAGGTGCTATACGA

AGACCCCGAAAACATGTTTTCTTGCGAGGCAGGTAACGAGATAAACCATATATCGGGTTCTGGATCGTTCTCGTTTATGCCAT

CTAAATACTGCGATGAGCCATCCGAAAAAACCGAAAATCAGGTCCAACAAGAGTTGAGATGGCTTAAAGCCAAATGCCAAA

TTGAGCTTAGAGATATTCAGGATGAACAACTAAAAACCCGGTGGCCGGAATCCGGAGAAGAGGTGGAAATTTCTCCGAAAG

ACGGGTTCTTGGGTTCGGTTTCCGGGTTAGGAAGAGAAGAAGATACGGTGAAAGAGATGTTTGGAGAAAGATTGGTACCAA

AGTGTCTGAAAAGAACAACTTCACTTCCTGTTGATGCCATTGATTCTTGA

>AtZIK5 (AT3G18750)

ATGGAAGGTACAGATGATGCTTCTGCACTTCAAGAGCCTCCTGACCCCGAAGTGCTCGAAGTTGATCCAACTTTTCGATATAT

ACGGTATAAAGAGGTCATTGGAAAAGGGGCATTCAAGACTGTTTATAAGGCATTTGATGAAGTAGATGGAATTGAAGTCGCA

TGGAACCAAGTACGGATAGATGATGTTTTGCAGTCACCGAATTGCCTGGAGAGGCTCTATTCTGAAGTGCGTCTGTTGAAAT

CATTGAAGCACAATAATATTATTAGGTTCTATAATTCATGGATCGATGATAAGAACAAGACTGTTAACATAATAACTGAGCTATT

CACTTCAGGGAGCCTCCGGCACTACCGCAAGAAACACAGGAAGGTGAACATGAAGGCCGTCAAGAATTGGGCGAGGCAGA

TTTTAATGGGTTTGAGATATCTTCACGGTCAAGAGCCACCAATTATACATCGGGATCTCAAATGCGACAACATTTTTATCAATG

GAAACCATGGAGAAGTGAAAATAGGAGATCTTGGTCTAGCCACTGTCATGGAGCAAGCTAATGCCAAAAGTGTGATTGGAA

CCCCGGAGTTTATGGCACCCGAGCTTTACGACGAGAACTACAACGAATTGGCTGATATTTATTCATTTGGGATGTGTATGTTG

GAGATGGTGACATTTGATTATCCCTACTGTGAATGCAAAAACTCTGCTCAGATATACAAGAAGGTTTCATCGGGAATAAAACC

TGCTTCACTCTCCAGGGTTAAAGACCCAGAAGTAAAACAGTTTATTGAGAAATGCTTACTTCCTGCATCCGAAAGGCTGTCA

GCAAAAGAGCTTTTATTGGACCCTTTTCTGCAACTAAATGGTTTAACAATGAACAATCCTTTGCCGCTTCCTGACATTGTAAT

GCCCAAAGAAGGCGCATTTGGAGACCGTTGCCTTATGTCTGAAGGTCCTCCTACAACACGGCCTAGTAAAACCTTGTCGATA

GATCTTGATGAAGACAGTAACCTGCCTATTGTTACTTTTAGCGACAATTCAGGATCCAGGTGTATTGAGGTTAGACGCGCAAA

GAGAGGGAATTTCTTTGTCCTCAAGGGTGAAGAAAACGATGAGCAGTCTGTGTCCTTAATTCTTCGTATAGTAGATGAGAAT

GGGCGTGTGAGGAACATTCATTTCCTGTTCTATCAAGAAGGGGACACTGCTTCTAAGGTGTCGAGTGAGATGGTTGAGCAA

CTCGAGTTAACTGACCAGAACGTCACGTTTATAGCCGAGCTGATTGATATACTACTTGTCAACATGATACCCACCTGGAAAAC

CGATGTCACAGTCGATCATCTCATCCATTCGCAACTGAATCAGAACTCAAGAAGTCATCATAACGAAGCAAAGCCACAGAAA

CAAGAGGAGACTGTTTTTCATGATACTTGTGAGTTGGTTAGTCACTCGTGTAACTCAGATTGCCCACGATCAGATGAGGAAG

ATAAGCAATGCGTCGATGCCACCAAAGGAGAAGACAAGAGCTCCATTCAAGAAGTAGAAGAAGCAACCGAACCAGTAAGT

TTGGAGGAAGAAGAAAGGTTAAGACAAGAGCTGGAGGAGATAGAAGCTAAGTATCAGGAAGATATGAAAGAGATAGCAAC

GAAAAGAGAAGAGGCCATTATGGAGACGAAGAAAAAGTTGTCTCTGATGAAGTTAAAGTAA

>AtZIK6 (AT5G41990)

ATGGCTTCTGGTTCTGGATTTTTAGGTCAGATATCGTCCATGGAAGAGGCTGATTTTGCCGAGAAAGATCCTTCTGGCCGTTA

CATCAGGTATGATGATGTTCTGGGGAGAGGAGCTTTCAAGACTGTATATAAGGCGTTTGACGAAGTAGACGGGATTGAAGTT

GCTTGGAACCTAGTTAGCATTGAAGATGTAATGCAGATGCCTGGTCAACTTGAAAGGCTATATTCTGAAGTTCATCTACTAAA

GGCTCTAAAACATGAAAACATCATCAAACTCTTTTACTCTTGGGTTGACGAAAAGAACAAGACTATTAACATGATCACTGAG

CTTTTCACCTCTGGTAGTCTCAGGGTATATCGTAAGAAGCATAGGAAGGTTGATCCCAAGGCCATCAAGAACTGGGCAAGGC

AGATTCTCAAAGGCCTGAACTATTTGCATTCTCAGAACCCTCCGGTAATTCACCGGGATCTGAAGTGTGACAATATATTTGTC

AATGGAAATACTGGAGAGGTCAAAATCGGAGATCTCGGGCTCGCAACTGTACTGCAGCAGCCCACTGCTCGAAGTGTGATA

GGTACTCCTGAATTCATGGCACCTGAACTGTATGAAGAGGAATACAATGAGCTTGTGGACATCTATTCTTTTGGCATGTGTAT

GTTGGAGATGGTAACCTGTGAATATCCATACAACGAGTGCAGAAACCAGGCTCAAATTTATAAAAAGGTCACCTCGAACATA

AAGCCTCAGTCTCTTGGCAAAGTTGACGATCCTCAAGTTAGGCAATTTATAGAGAAATGTCTTCTTCCTGCCTCTTCCAGACC

AACTGCCCTCGAGCTCTCGAAGGACCCATTCCTTGCAAGGGATGGAGGCAAGGACTCTGCTCTGCTTGCTTCATCAAGCAC

ATCATCTAAATATGTGAGACCACCACAGCTTGAACATCTTCCCATGGATGTGGATCATAACGAGAATAAAAGTGTTTCAAGCA

ACGAAGACTACCCATGGTCCCAAACCATTGAACTTCAGAGGATTGCAGAGAATAAAGAGTTCAGGTTGAGAGGTGAGAGG

AGCGATGATGTCACAGCGTCTATGGTTCTCCGTATTGCTGACCCGTCTGGTAAATGTAGAATCGTACACTTTGCATTCTATCTG

GAGTCAGACACAGCAACAGCAATCGCTGAGGAAATGGTTGAGGAACTGCATTTAACTAGCCAAGAGGTCGTTGTGATAGCT

GATATGATCGATGACTTCATAATGCAACTTCTCTCTGACCGGACCTCGTCGCATCATAACCAAAACTCTCCACGTCTAACCCA

TGAGGATCATGAAGCAGCAAATCAGCAAACTGTGAACTCGAAAGATGAAGAAGCTGCAGGACAATCAATGAAGTCAGACA

TATCAGCAGACTATTACTTTCCCTACTCAGCAAACGACGGAAATGCTGCCATGGAAGCTGGTCGAGATGCAGAGTCGATGAG
CTCGTATCTGGATTCTTGCTCAATGATGTCAACCATTTACAATCTTTCCATTTCAGACAATGATTACCCAGAAGATCTCAAGAC
AGAACTAAACCTGATCGAGTCACAGTTTAACCAGTCCTTCCAAGATCTGCTGAAACTGAAAGAGGATGCAATAGAGAACGC
TAAGCGAAAATGGATTACGAAAAAGCAAAAAGCAGTTAACATCTCTTGA

>AtZIK7 (AT1G49160)

ATGGAAGGTTCAGAAGATGCTTCTGCAATTGTTGAACCACCTGACCCAGAAGTTCTTGAAATCGACCCAACTTGTCGATATA
TTCGGTACAAAGAAGTAATAGGCAAAGGCGCATCCAAGACAGTTTTTAAGGGATTCGACGAAGTAGATGGGATTGAAGTAG
CGTGGAATCAAGTAAGGATCGACGATCTTTTGCAATCACCAGATTGTCTAGAGAGGCTATATTCCGAAGTGCGGCTTTTGAAA
TCTCTGAAGCATAAAAACATCATAAGGTTTTATAACTCGTGGATCGATGACAAGAACAAGACGGTTAACATAATTACCGAGTT
GTTCACTTCAGGAAGTCTCCGGCAGTACCGTAAGAAACATAGAAAGGTGAATATGAAGGCTGTCAAGTGTTGGGCGAGACA
GATTTTAACGGGATTGAAATATCTACATAGTCAAGATCCGCCGATAATACACCGAGATATCAAATGTGACAACATTTTTATCAA
CGGTAATCATGGAGAAGTGAAAATAGGAGATCTTGGTTTAGCTACTGTCATGGAACAAGCTAATGCCAAAAGTGTGATTGGA
ACACCGGAGTTTATGGCGCCTGAGCTTTACGATGAGAACTACAACGAGCTCGCTGATATATATTCGTTCGGGATGTGTATGTT
GGAAATGGTGACTTTTGAATATCCTTATTGTGAGTGTAGAAACTCTGCTCAAATCTACAAGAAAGTTTCATCGGGGATCAAAC
CAGCTTCACTATCAAAGGTTAAAGATCCAGAGGTAATGAAATTTATCGAGAAATGTTTATTGCCAGCTTCCGAAAGGTTATCG
GCAGAGGAGCTTTTATTGGATTCTTTTCTCAATGTGAACGGTTTAGTTATGAACAATCCATTACCGCTTCCCGACATTGTAATG
CCGAAAGAAGGATCATTCGGTGAACGTTGTCTTATGTCTGAAGGACCACCTAATGCTCGGAATAGAACGATGTCAATGAATC
TTGACGAAGACAATAACCTTCCTATTGTTATTTCAAGCAACAATTCAGGAACAAACTGTATTGAGGTTAGACGGGCAAAGCG
AGGAAACTTTTTTGTCCTTAAAGGTGAAGAGAATGATGAGAACTCTGTCTCATTAATTCTTCGTATAGTCGATGAAAATGGGC
GTGTGAGGAACATTCATTTTCTATTCTTTCAAGAAGGTGACACTGCTTCTAATGTATCTAGTGAGATGGTTGAACAGCTTGAA
TTGACGGATAAAAACGTAAAGTTTATAGCGGAATTGATTGACGTGTTACTCGTCAATTTGATACCGAATTGGAAAACCGATGT
AGCTGTCGATCATCTAATTCATCCGCAACAGAACCAGAGCTCCAAGGATAATCATCAAAACGGTGCAAGTTCTCAAGCTGGT
GAATCCATTAGCCACTCTTTGTCCTCTGATTATTGCCCACGATCCGATGATGAGGCAAACCCGACCGTAGCTGCAACAACAG
AAGATCAAGAAGCAGAGAAACCAGGAAGCTTGGAGGAAGAAGAAGAAGATGAGAGGTTAAAAGAGGAGTTAGAAAAGAT
AGAAGAACGGTTCAGAGAAGAGATGAAAGAGATAACGAGGAAAAGAGAAGAAGCAACAATGGAGACCAAAAATAGATTT
TTCGAGAAGAAGATGCAACAAGTTGAGTAA

>AtZIK8 (AT5G55560)

ATGATGACTTGTGCGAGCTCCGATGATAATGAGAGTGAAAAGGACAAGGACTCGGAGTCCTTCGTGGAGGTAGATCCGACA
GGTAGATACGGTCGGTACGGCGAACTTCTTGGCTCAGGCGCCGTAAAAAAAGTCTACAGAGCCTTTGACCAAGAAGAAGGC
ATCGAGGTGGCTTGGAACCAAGTCAAACTGAGATGTTTTTCGGATGATCCAGCCATGACCGAGAGGCTTTACTCGGAGGTC
AGGTTGCTTAAGAACCTTAAGAACAGTAACATCATCACCCTGTATAAAGTGTGGAGAGACGAGAGAAACAACACCTTGAAC
TTCATCACCGAGATCTGTACCTCCGGAAACCTGAGAGAGTACCGGAAGAAGCACAGACACGTCTCTATGAGGGCTTTGAAG
AAGTGGTCCAAACAGATTCTCAAGGGCTTGGATTATCTCCACACTCACGACCCTTGCATCATCCACAGAGACCTCAACTGTA
GCAATATCTTCGTCAATGGCAACATCGGCCAGGTCAAGATTGGTGATCTAGGTTTAGCTGCAATTGTGGGGAAGAACCATTTA
GCTCACTCGATCCTCGGGACACCAGAGTTCATGGCTCCTGAGCTGTACGAGGAGAACTACACTGAGATGGTTGACATATACT
CGTATGGAATGTGCGTTTTGGAGCTTGTGTCACTCGAGATTCCTTATAGCGAATGTGACAGCGTCGCCAAAATATACAAAAG
GGTGAGCAAAGGCCTCAAACCAGAGGCTCTCAACAAAGTTAATGATCCAGAAGCTAAGGCATTTATTGAAAAATGCATCGC
GCAGCCGAGAGCTAGACCTTCTGCAGCTGAGCTCCTCTGCGACCCGTTCTTTGATGGGATATTAGATGATGATGACGAAGAC
GGTGAAAACAATGACAACAATGGAGCTGGTCGTATTGTTGTTTCTTGA

>AtZIK9 (AT5G28080)

ATGATGAACAATCTCAGCCATCTTGAATCAGATTACTCTGAGTATGTTGAAGTTGATCCTACTGGAAGATATGGAAGATACAA
TGAAGTTCTGGGTAAAGGATCTTCAAAGACTGTTTACAGAGGATTTGATGAGTATCAAGGTATAGAAGTAGCATGGAACCAA
GTAAAGCTCTACGATTTCTTGCAGAGTCCTCAAGAGCTAGAGAGGCTCTACTGTGAGATCCATCTCCTCAAAACCTTAAAAC
ACAAAAGCATCATGAAGTTCTACGCTTCTTGGGTCGATACCGATAATCGAAACATCAACTTTGTCACTGAAATGTTCACTTCT

GGCACCTTGAGACAGTATAGGCTAAAACATAAGAGAGTGAACATAAGAGCGGTAAAGAATTGGTGTAGACAGATTTTAAGA
GGATTAAACTATCTTCATACACATGACCCTCCTGTGATTCATAGAGATCTCAAGTGTGACAACATTTTTATAAATGGGAACCAA
GGAGAAGTCAAGATTGGAGATCTTGGTCTTGCTGCTTGTTTACAACACTCTCACGCTGCTCATTGTGTTGGCACACCAGAGT
TCATGGCTCCTGAAGTTTATAAAGAAGAATATAACCAATTAGTCGATATTTACTCGTTCGGTATGTGTGTTTTGGAAATGGTAA
CGTTTGATTACCCGTATAGTGAGTGTTCTCACCCTGCTCAGATTTACAAGAGAGTTATATCGGGCAAGAAACCAGATGGATTA
GACAAAGTGAAGGATCCAGAGGTTAGAGGGTTTATCGAGAAGTGTTTGGCCACAGTGTCCCTTAGACTCTCTGCTTGTGAA
CTACTAGATGACCATTTTCTTTGTATCGATGAATCGGATATGAGACGCGTAGAGAGCGAAAAGGGCTTGATAGATGAAGCAG
GGACTCCTCTTAGACATTCTTATCATATACCTCACTACTCAAACGGTTACTACAGCCTCTATAATCAAAACCAATGGGATTACA
ATGGAGATGAGACAGTGGAGTCACACGAGATAGATCTCTTGGAGTTTCAAAATGATGATGATGAAGAAGAAGAAGATAAGA
GGTTTGGTAGTGTAGATATAAGCATTAAAGGGAAGAGAAGAGACAATGGAGATGGACTATTTCTGCGACTCAAAACCGTCA
ACAAGGAAGGTTGTGTGAGAAACATATACTTCCCGTTCGACATCGAGACTGACACAGCAATAAGCGTTGCAAGAGAGATGG
TGGAAGAGCTGGAGATGGATGACCGTGATGTCACAAAGATAGCTAATATGATCGATGGAGAGATTGCTTCTTTGGTTCCTAAT
TGGAGTATCTTCTGCAGCTCCGAATCGAACCGTTCCTCGGTGGGTTCAGTCATGGATTTCAATGAGATGCAGTGTGGAAGAG
ACGGGTGTGAAGAGAAGCATGGACGGTTTGAGGAGATTACGTTTGAGATAACAGTAAACGACTCTGACGAAGAAGACTAG

>AtZIK10 (AT1G64630)

ATGGAAGAGGCTGACTTCGTCCAGAAAGATCCCACTGGTCGCTACATTAGGTATAATGACGTTCTCGGGAGAGGAGCCTTCA
AAACTGTATATAAGGCATTTGATGAAGTCGAAGGTATTGAAGTTGCTTGGAACCTCATGAGCATTGAAGATGTCTTGCAGATG
CCTGGCCAACTTGATAGGCTATATTCCGAAGTCCATCTCCTCAATTCCCTTAAACATGATAACATCATCAAACTCTTCTACTCT
TGGGTTGATGATCATAACAAGTCCATCAACATGATCACTGAGCTTTTCACCTCTGGTAGTCTCACCCTTTATCGCAAGAAGCA
CCGCAAAGTTGATCCCAAGGCCATCATGAACTGGGCAAGGCAGATTCTTAAAGGCCTGCACTATTTGCATTCTCAGACCCCT
CCTGTAATTCACCGTGACCTTAAGTGCGACAATATTTTTGTCAATGGAAATACCGGAAAGGTCAAAATTGGAGATCTTGGGCT
CGCTGCTGTGATGCAGCAACCCACTGCTCGAAGTGTGATTGGCACTCCTGAATTCATGGCGCCTGAGCTTTATGAAGAAGAA
TATAATGAACTTGTCGACATCTATTCTTTTGGCATGTGCATGTTGGAGATGGTAACATGTGAATATCCATACAGAGAATGCAGA
AACCAGGCTCAAATATACAAGAAGGTTACCTCGGGTATAAAACCTCAATCTCTCAGCAAAGTTGATGATCCTCAAGTTAAGC
AATTCATAGAGAAGTGTCTTCTCCCAGCTCCTTCTAGACCAACTGCTCTAGAGCTCCTGAAGGACCAACTCCTTGCAGTAGA
TGGCGCCAAGGACTCCACTCTTACTGCTTCATCAAACACAACATTCAAACCTGCAATGCCACCACAGTGTGAATATCGTCCT
ATGGATGTTGAATATAAGAAGAATACAAGTGTTTCCATCTGCTCTTCTGCTAAAAGCAGCCAAGAATGCGCATTGCTCCAGAC
CATGGAAGTCCAGAGGGTTGCTGAAAGTACCGAATTCAAGCTGAGTGGAGAGAGGAGAGATGATGTGGCAGCATCAATGG
CTCTGCGGATCGCGGGCTCATCTGGTCAAGCGAGAAAAGTTGATTTTGACTTCAATCTGAAGACAGACACAGCAAGAGCAG
TTACAGGGGAGATGGTTGAAGAGCTTGATCTGTCAAGCCATGAGGTTACTGTGATAGCTGAGATGATAGATGAACTGATAAT
GAAGCTGAAGGCTAATCGAAGCCTGCCTAATGCAAACAGTGTATATCAGTCCAAGGATGAAGAAGCTGGAGAATCAATGAA
GTCAGAGATATCAGCGGACTACTACCATCGGGTCTCCTCAAACGAGGGGTCACGGCTCGGCTGCTGCTGCGAGGCAGTAGA
GTCATTGTTGTCCTCGTTTCTGGATTCATGCTCAATGGTGTCAAACAAGCAGTCGGAGGATCTGAAGACGGAGCTGAATGTG
ATAGAGTCACAATATAACCAATCGTGTCAACGACTGCTTAGAATGAAAGAGGAAGCTATAGAGAAGGCGAAACGGAAGTGG
ATGAAGCTGAGTTGA

>AtZIK11 (AT3G48260)

ATGCGACAAGACGAGAATAACTCCGAGGAAGAATTCGTTGAGATTGATCCCACTGGTCGCTATGGCCGGTATAAAGAAGTTC
TAGGCAAAGGAGCTTTCAAAGAAGTATACAGAGCATTTGACCAACTTGAAGGAATCGAAGTGGCTTGGAACCAAGTTAAGT
TAGATGATAAATTCTGTAGCTCAGAGGATTTAGATCGTCTTTACTCTGAAGTTCACTTACTCAAAACCCTTAAACACAAAAGC
ATCATCAAATTCTACACTTCTTGGATCGATCACCAACACATGACTATCAATCTCATCACCGAAGTCTTCACTTCCGGAAATCTT
AGACAGTACCGGAAGAAACATAAGTGTGTTGATCTAAGAGCATTGAAGAAATGGTCAAGGCAGATCTTAGAAGGGCTTGTT
TATCTACATAGCCATGATCCTCCTGTGATCCATAGAGATCTTAAATGTGATAACATTTTCATTAATGGGAACCAAGGTGAGGTC
AAAATTGGAGATCTAGGGCTAGCCGCGATTCTGCACCGCGCTCGCTCTGCTCATAGCGTCATCGGAACTCCTGAATTTATGGC
GCCGGAACTCTATGAAGAGGACTATAATGTACTTGTCGATATCTACGCGTTTGGAATGTGTTTGCTTGAGCTGGTAACTTTTG

AATACCCGTATTCCGAATGCACAAATGCTGCTCAGATTTATAGGAAAGTTACATCGGGAATCAAACCAGCCGCTCTTTTAAAT
GTGACTGATCCGCAAGTGAGAGCATTCATAGAGAAGTGTATCGCAAAGGTCTCGCAACGCTTGTCAGCCAAGGAACTACTT
GATGATCCTTTTCTGAAATGCTACAAGGAAAATACCGAAAACGTCAGCTCTCACAAAGAAAATGGTTACAACGGAAACGGA
ATCGTGGATAAACTCTCGGATTCTGAGGTAGGCTTATTAACCGTAGAAGGCCAACGTAAAGACCTCAACACAATCTTCCTAA
AACTACGTATCACCGATTCCAAAGGTCAAATCCGCAATATCCACTTCCCATTTAACATAGAGACAGACACATCTTTCTCAGTC
GCCATCGAAATGGTCGAAGAACTAGACTTAACCGATGATCAAGACATCTCAACGATAGCTAAAATGATCGACACAGAGATAC
ATTCACACATCCCCGATTGGACCCCTTCTCGACTTATCGGTGATGATTCAGCGGTACAAAAATGTTTGTCTTCTCCAGAGACG
CTCCATTTAGATAGATTTCCTTCAGGGAGAAAATTCTGGTCCTCTCCTAAAGCTGGAGCTGGAGACTCACGTTCACCGTTTGC
GCCACGGTCTAATTCTAAGTTGTCGTCAGCACAAGGACCGATCAATCAAGAAGTTGGGGTTATAGTAGAGAAACTTGAGTCT
TTGTTGAGGAAACAGAGAGAGGAGATTGAAGAGATGCAGAGAGATCAAGAACGTATTGTTACTGAGTTCTTAAAAGAGTTT
CCTCCTGAGATTTGTGAAGAGGCTTTGGTGAGATTGCAAGTTAAAGATTCTGATAACTTATTGTGTTAG

>AtRAF1 (AT5G03730)

ATGGAAATGCCCGGTAGAAGATCTAATTACACTTTGCTTAGTCAATTTTCTGACGATCAGGTGTCAGTTTCCGTCACCGGAGC
TCCTCCGCCTCACTATGATTCCTTGTCGAGCGAAAACAGGAGCAACCATAACAGCGGGAACACCGGGAAAGCTAAGGCGGA
GAGAGGCGGATTTGATTGGGATCCTAGCGGTGGTGGTGGTGGTGATCATAGGTTGAATAATCAACCGAATCGGGTTGGGAAT
AATATGTATGCTTCGTCTCTAGGGTTGCAAAGGCAATCCAGTGGGAGTAGTTTCGGTGAGAGCTCTTTGTCTGGGGATTATTA
CATGCCTACGCTTTCTGCGGCGGCTAACGAGATCGAATCTGTTGGATTTCCTCAAGATGATGGGTTTAGGCTTGGATTTGGTG
GTGGTGGAGGAGATTTGAGGATACAGATGGCGGCGGACTCCGCTGGAGGGTCTTCATCTGGGAAGAGCTGGGCGCAGCAG
ACGGAGGAGAGTTATCAGCTGCAGCTTGCATTGGCGTTAAGGCTTTCGTCGGAGGCTACTTGTGCCGACGATCCGAACTTTC
TGGATCCTGTACCGGACGAGTCTGCTTTACGGACTTCGCCAAGTTCAGCCGAAACCGTTTCACATCGTTTCTGGGTTAATGG
CTGCTTATCGTACTATGATAAAGTTCCTGATGGGTTTTATATGATGAATGGTCTGGATCCCTATATTTGGACCTTATGCATCGAC
CTGCATGAAAGTGGTCGCATCCCTTCAATTGAATCATTAAGAGCTGTTGATTCTGGTGTTGATTCTTCGCTTGAAGCGATCAT
AGTTGATAGGCGTAGTGATCCAGCCTTCAAGGAACTTCACAATAGAGTCCACGACATATCTTGTAGCTGCATTACCACAAAA
GAGGTTGTTGATCAGCTGGCAAAGCTTATCTGCAATCGTATGGGGGGTCCAGTTATCATGGGGGAAGATGAGTTGGTTCCCA
TGTGGAAGGAGTGCATTGATGGTCTAAAAGAAATCTTTAAAGTGGTGGTTCCCATAGGTAGCCTCTCTGTTGGACTCTGCAG
ACATCGAGCTTTACTCTTCAAAGTACTGGCTGACATAATTGATTTACCCTGTCGAATTGCCAAAGGATGTAAATATTGTAATAG
AGACGATGCCGCTTCGTGCCTTGTCAGGTTTGGGCTTGATAGGGAGTACCTGGTTGATTTAGTAGGAAAGCCAGGTCACTTA
TGGGAGCCTGATTCCTTGCTAAATGGTCCTTCATCTATCTCAATTTCTTCTCCTCTGCGGTTTCCACGACCAAAGCCAGTTGA
ACCCGCAGTCGATTTTAGGTTACTAGCCAAACAATATTTCTCCGATAGCCAGTCTCTTAATCTTGTTTTCGATCCTGCATCAGA
TGATATGGGATTCTCAATGTTTCATAGGCAATATGATAATCCGGGTGGAGAGAATGACGCATTGGCAGAAAATGGTGGTGGGT
CTTTGCCACCCAGTGCTAATATGCCTCCACAGAACATGATGCGTGCGTCAAATCAAATTGAAGCAGCACCTATGAATGCCCC
ACCAATCAGTCAGCCAGTTCCAAACAGGGCAAATAGGGAACTTGGACTTGATGGTGATGATATGGACATCCCGTGGTGTGAT
CTTAATATAAAAGAAAAGATTGGAGCAGGTTCCTTTGGCACTGTCCACCGTGCTGAGTGGCATGGCTCGGATGTTGCTGTGA
AAATTCTCATGGAGCAAGACTTCCATGCTGAGCGTGTTAATGAGTTCTTAAGAGAGGTTGCGATAATGAAACGCCTTCGCCA
CCCTAACATTGTTCTCTTCATGGGTGCGGTCACTCAACCTCCAAATTTGTCAATAGTGACAGAATATTTGTCAAGAGGTAGTT
TATACAGACTTTTGCATAAAAGTGGAGCAAGGGAGCAATTAGATGAGAGACGTCGCCTGAGTATGGCTTATGATGTGGCTAA
GGGAATGAATTATCTTCACAATCGCAATCCTCCAATTGTGCATAGAGATCTAAAATCTCCAAACTTATTGGTTGACAAAAAAT
ATACAGTCAAGGTTTGTGATTTTGGTCTCTCGCGATTGAAGGCCAGCACGTTTCTTTCCTCGAAGTCAGCAGCTGGAACCCC
CGAGTGGATGGCACCAGAAGTCCTGCGAGATGAGCCGTCTAATGAAAAGTCAGATGTGTACAGCTTCGGGGTCATCTTGTG
GGAGCTTGCTACATTGCAACAACCATGGGGTAACTTAAATCCGGCTCAGGTTGTAGCTGCGGTTGGTTTCAAGTGTAAACGG
CTGGAGATCCCGCGTAATCTGAATCCTCAGGTTGCAGCCATAATCGAGGGTTGTTGGACCAATGAGCCATGGAAGCGTCCAT
CATTTGCAACTATAATGGACTTGCTAAGACCATTGATCAAATCAGCGGTTCCTCCGCCCAACCGCTCGGATTTGTAA

>AtRAF2 (AT1G08720)

ATGAAGCATATTTTCAAGAAGCTACACAGAGGTGGGAATCAAGAGCAGCAGAATCGAACCAACGATGCAGCTCCTCCATCG

GATCAAAATCGGATTCACGTTTCTGCTAATCCTCCTCAAGCAACCCCTTCGTCAGTCACTGAGACGCTTCCGGTGGCCGGAG

CTACTTCTTCTATGGCCTCTCCTGCTCCAACCGCTGCTTCGAACCGTGCGGATTACATGTCTTCTGAGGAGGAGTATCAAGTG

CAGTTAGCCCTAGCGATCAGTGCTTCGAATTCGCAGTCCAGTGAGGATCCGGAGAAGCATCAGATCCGAGCGGCGACGCTT

CTGAGCTTAGGAAGCCATCAACGGATGGATTCAAGGAGGGATTCATCGGAGGTGGTAGCCCAGAGGTTATCGAGACAGTAC

TGGGAATATGGCGTGCTTGACTATGAGGAGAAAGTTGTCGATAGTTTCTACGACGTATACAGTCTATCCACAGACTCCGCAA

AGCAGGGAGAAATGCCATCGCTGGAAGATCTTGAAAGCAATCATGGCACACCTGGCTTTGAAGCTGTAGTTGTAAATCGAC

CTATTGATTCTTCCCTGCATGAGTTGCTAGAAATCGCAGAGTGTATTGCACTGGGTTGTTCTACTACCAGTGTTAGTGTGTTGG

TACAGAGGCTGGCTGAGCTTGTCACCGAGCATATGGGTGGATCTGCGGAAGATTCCAGTATAGTATTGGCAAGGTGGACTGA

AAAAAGCAGCGAGTTCAAGGCAGCATTGAATACTTGCGTATTCCCTATTGGATTTGTAAAGATTGGTATCTCAAGGCATCGTG

CTCTGCTTTTCAAGGTTTTGGCAGATAGTGTCAGGTTACCTTGTAGGTTGGTAAAAGGTAGCCACTACACAGGCAATGAGGA

TGATGCTGTGAACACGATAAGACTGGAAGATGAAAGAGAGTACTTGGTTGATCTTATGACAGATCCTGGGACGCTTATACCC

GCTGATTTTGCAAGTGCTAGTAATAACACCGTTGAGCCATGTAACTCAAATGGAAACAAATTTCCTACAGCTCAGTTTTCAAA

TGACGTGCCAAAGCTCTCAGAAGGTGAAGGAAGTAGTCACAGTTCTATGGCCAACTATAGTTCTTCTTTGGATAGAAGGACA

GAGGCTGAAAGGACAGATTCTTCATACCCAAAGGTGGGACCACTTCGGAACATAGACTATAGTTCTCCTTCTAGCGTAACTA

GTTCTACTCAGTTGGAGAACAATTCCTCAACAGCAATTGGAAAGGGGAGTCGAGGAGCCATAATTGAATGTTCAAGAACAA

ACATGAATATAGTTCCTTACAATCAGAACAGTGAGGAAGACCCAAAAAACCTTTTCGCAGACCTTAATCCATTTCAAAATAA

GGGAGCTGACAAGCTGTATATGCCCACTAAATCAGGTTTGAATAACGTTGATGATTTTCATCAACAGAAAAATAATCCTCTGG

TTGGTAGATCACCTGCGCCAATGATGTGGAAGAATTACAGTTGCAATGAAGCCCCAAAGAGAAAGGAGAATAGTTATATAGA

AAATCTTCTCCCGAAACTCCACCGTGATCCTCGTTATGGAAACACTCAATCCTCATATGCTACCTCAAGCTCCAATGGAGCTA

TTTCCTCAAATGTGCATGGCAGAGACAATGTGACATTTGTGTCACCGGTTGCTGTACCATCATCCTTCACATCCACTGAAAAT

CAGTTTAGACCAAGTATAGTGGAGGATATGAACAGAAACACCAACAATGAACTAGATCTTCAGCCTCATACTGCTGCTGTGG

TACATGGACAACAGAATGATGAATCTCACATCCATGATCACAGAAAGTACACAAGTGATGACATATCCACTGGCTGTGATCC

GAGGCTTAAGGATCACGAAAGTACAAGTTCATCTCTTGATTCTACATCCTACCGGAATGATCCTCAAGTTCTTGATGATGCAG

ATGTTGGTGAATGTGAAATTCCTTGGAATGATCTCGTTATTGCGGAGAGAATAGGATTAGGGTCCTATGGAGAGGTCTATCAT

GCTGACTGGCATGGCACGGAAGTTGCTGTCAAGAAATTTTTGGACCAGGACTTCTCAGGTGCTGCTTTAGCCGAGTTCAGA

AGCGAAGTACGGATTATGCGAAGATTGCGTCATCCAAATGTTGTATTCTTCCTTGGGGCTGTTACTCGTCCTCCAAATCTTTCC

ATCGTCACAGAGTTTCTGCCTAGAGGAAGCTTGTATCGAATCCTTCATCGGCCCAAATCTCACATTGACGAGCGGCGCCGGA

TTAAAATGGCCCTTGACGTGGCAATGGGGATGAACTGCTTACACACCAGTACACCGACAATTGTTCATCGTGATCTCAAAAC

ACCAAACCTTTTGGTTGATAACAACTGGAATGTTAAGGTCGGTGATTTTGGGTTGTCTCGCTTAAAGCACAATACTTTTTTAT

CCTCCAAATCAACTGCTGGAACGCCTGAATGGATGGCTCCAGAAGTTCTACGCAATGAGCCCTCAAATGAAAAGTGTGATGT

GTACAGTTTCGGGGTAATACTTTGGGAACTAGCAACATTGAGATTACCATGGAGAGGAATGAACCCAATGCAAGTAGTTGGA

GCAGTTGGGTTCCAGAATCGGCGGCTTGAGATCCCCAAGGAACTTGATCCTGTGGTGGGAAGGATCATCCTGGAATGTTGG

CAAACGGATCCGAATCTGCGGCCGTCATTTGCTCAGCTGACGGAAGTGCTGAAGCCTTTGAACCGGCTTGTACTTCCTACAC

CACAATAG

>AtRAF3 (AT5G11850)

ATGTCGAAGATGAAGCATCTTCTACGGAAGCTCCACATCGGCGGAAGTAGTGGCGTCGGCGGCGGATTTGCTGACCATCAC

AGGTTAGACGACTCGACTAGACCTATGATCGATCCTAGCCCTATTCTTAGTACTAGCCCTAGCCCTGCATCGACTTCTTCCGTC

TCTTCCTCTGGTTTTGGTAACGCTTCCACGACAATGCCGAGACTGGATACATTTGAGCCTGTTGGCCGTGATCTGACGGCTGT

TGATGGTGTTGATTTCAATTTGATGGAGGAGGAGTACCAAGTCCAGTTAGCTATGGCGATCAGCGTCTCTGATCCTGATCCGA

GAGAGAATGCAGATACAGCTCAGCTTGATGCCGCTAAGAGGATTAGCCTTGGGGTTTCTGCTCCGGTCACCGACGCTGATTC

CGCCGTTGACTTTCTCTCGCTTCGTTATTGGGGACATAAGGTCATTAATTATGACCAGAAAGTCAGGGATGGATTTTACGATG

TGTATGGGATTACATCTAATTCTCTTTCACAGGGGAAGATGCCACTTCTTGTTGATCTTCAAGCGATCTCTATTTCAGATAATG

TTGATTATGAGGTCATTCTAGTTAACAGATTGATTGATCCTGAACTACAAGAGCTAGAGAGGAGAGTATTCGCTTTGGCTTCG

GAATGTCCAGACTTTGCTCCTGGTCAGGTGTCAAGTGATTTGACTCAGAAAATTGCAAATATAGTTGTAGAGCAAATGGGTG

GCCCCGTTGAAAATGCTGATGAAGCATTGAGAAGGTGGATGCTTCGGAGCTATGAACTAAGAAATTCTTTGAACACTACTAT
TCTTCCACTTGGTCGAGTTAATGTTGGTCTTGCACGACACAGGGCTTTGCTTTTCAAGGTCCTTGCTGATAGGATTAATCTCC
CATGTATGCTGGTAAAAGGCAGTTACTACACTGGAACTGATGATGGGGCTGTGAACTTGATTAAACTAGATGACAAAAGTGA
ATACATTATTGATTTAATGGGTGCTCCGGGTGCTCTGATCCCTTCTGAGGTTCCAAGCAGTTTTCTTCCAGTTTCTTGCACAGA
TACAAGAGTATTTCCTGAGAATTTGGACTCTTTGCAACATTCATCCCCCGTACTTGAGAAAGAAATTGAAACGCCAGCATTTT
CAGTTTCGAAGGAAGCAGATTCTAGATCTGGTATGGTAGCAAACTTCTTCACTGGAAACCAGGAAGAAAACAGTGACAGAT
GTGCTGTTGAAAAACATCAAACAGAGAGATTTGAGCATGATTTTGGGAAGTTAATGCACTCACAGCAGATATCTGGTGAAAA
TATGCCACCATTTTCTGGGAAACCGACTTGTGCACAGAAAGTTAAAGTTAAAAATGTCTCAAAGTATGTCATAAGTGCAGCA
AAGAACCCTGAATTTGCGCAGAAATTACATGCTGTGTTGTTAGAAAGTGGTGCATCACCTCCCCCAGATTTGTTTATGGATAT
TAACCCACATAACTTGAGGGGGAAGAATTTGCTTCAAGAGCTCCGCCAAGAAAGTAGCAATTCTATGGTTTCTGGTATTCCA
TGCTACCCAGAAAAGGTAGCTGAACAACTGAGAGAATCTGAAAGGAACCCCACAGCCGAGAGTTACCAACAATCAGTGGA
GGTCGATTTGTCAATGAAGAGGAACTTTGATTTGGATAATACTGGTAAAGCTTCTTCATCCGAAAATATGGAGGTTGGCACTG
CTGATGGGGAGTCTGCTGTTTGTGATAGTCATGACCAAGGGATTAATCCATTGCTCGGAGAAGCTGCAAAGTGGGAAATTAT
GTGGGAAGATCTTCAGATTGGCGAGCGCATTGGTATTGGTTCATATGGAGAAGTTTATCGTGCAGAGTGGAATGGAACTGAA
GTGGCTGTTAAGAAGTTTCTGGACCAAGATTTCTCTGGTGATGCATTGACACAGTTCAAATCTGAAATTGAAATAATGTTGA
GGTTACGGCATCCAAACGTTGTTCTTTTCATGGGAGCAGTTACTCGTCCCCCAAATTTCTCCATCCTGACAGAGTTCCTACCC
AGGGGAAGTTTGTATAGATTACTCCATCGGCCGAACCATCAGCTTGATGAGAAGAGGAGAATGCGGATGGCTCTTGATGTGG
CAAAGGGAATGAACTACTTACACACCAGCCACCCGACTGTTGTACATAGGGATTTAAAATCTCCAAACCTTCTTGTTGATAA
AAATTGGGTTGTGAAGGTTTGTGATTTTGGATTGTCCCGCATGAAACACCACACATATTTGTCCTCGAAATCAACTGCAGGA
ACGCCTGAGTGGATGGCTCCAGAAGTGTTGAGGAATGAACCGGCTAATGAGAAATGTGACGTGTACAGCTTTGGTGTCATAT
TGTGGGAATTAGCTACTTCACGCGTCCCCTGGAAAGGTTTGAACCCGATGCAAGTCGTTGGAGCTGTGGGATTCCAGAATCG
ACGCCTTGAAATCCCAGATGATATCGATCTAACTGTGGCACAGATAATCCGTGAATGTTGGCAAACGGAACCGCATTTACGG
CCATCGTTTACACAGCTGATGCAAAGTTTGAAGCGGCTTCAGGGTCTAAACATAAGCAACAGAGCGAATACGAGTGAAAGT
TTGATGTAA

>AtRAF4 (AT1G18160)

ATGAAGATGAACATGAAGAAATTTCTCAAAAAGCTTCGTATCACGCCCAATCAACGAGACGATGGTGAAGGTTCAGTCTCTA
ACAGAAGCAATAAGTCTAGTGATGCAGAACCATCTCCTTCTGATTCATTGAGGTCTCAAGATAACTCTGAATTCAAACCCTTT
TTGGGTTTATCAAATTGGTTAAGCTCTGTTACTCACAGGAAAAGCCCTTCTTCTTCCAATGCCACCAATAGCAAAGAGGATG
ACACTACCATGGAACATGGTGGTCCCGTTGGGTCGGAATCTGGGATGCAGGGTTTGGGTTCTAGTAGTAATTCCAAGGATCC
AGAAGTTGAAGAAGAATATCAGATACAGTTGGCTCTAGAGTTAAGTGCTAGGGAGGATCCTGAAGCTGCTCAGATTGAGGCT
ATGAAGCAGTTCAGCTTAGGTTCTCGTCCTTCTGCTCCCGAGAATACTCCAGCTGAACTCATGGCATATAGATATTGGAATTAT
AACTGTCTTGGCTATGATGACAAGATTGTGGATGGTTTCTATGACCTGTGTGGAGTAATGAATGAATCTTCCTTAAAAAGAAT
ACCTCCCTTAGTTGATCTTCAGGGGACACTTGTGTCAGATGGTGTAACTTGGGATGCTGTTCTAGTGAACAGCAGCAAAGAT
TCTAATCTGTTGAGACTTGAACAAATGGCTCTTGATATTGCAGCAAAATCAAAGTCAGCGTCTTCTTCAGGGTTTGTGAACA
GTGAATTGGTAAGGCAACTGGCAGTTTTAGTTGCTGATTACATGGGTGGACCAGTTTTGGATCCAGATAGCACGTTAAGAGC
TTGGTGGAGTCTGAGCTACAGCTTGAAAGCAACCCTCCGTAGCATGGTTCTGCCCCTGGGTTCTCTAACTATTGGATTGGCTC
GTCATCGTGCCTTGTTATTCAAAGTTTTGTGTGATAGTGTCGGCGTTCCTTGTCGAATAGTCAAAGGACAGCAATATACCGGT
TCAGATGATGTGGCAATGAACTCCATTAAGACTGATGATGGCAGGGAGTACATTGTTGATCTCATGGGAGATCCAGGCACGC
TTATCCCCGCTGATGCAGCTGGACTACAAATGGACTTTGATGATTCTGTCTACTCTGCCAGTCCCAGAGATGTTGATTCATCTC
ATGTAGCTTCTTCCAGCAGTGGGGTCGAGAGCTCAATTGAAGAACACACAGAATCTTGGTCAGCGGAACATCGTTCTAGGA
CCAAGGGTTCCCGGGAGGAAAACCAATCTGCAGGTGGAGGGGATCTCATGATTCCAAATATTAGGGAAGCTGTGGGAAGTC
AAAAGGCTCCTGTTCAACATCTTTCCAGCAAGCCTACTCATTCTTTCACTCATGCCAGATCACCTTCGTGGACTGAAGGTGTT
AGCTCTCCAGCTGGACGCAGGATGAAAGTCAAAGATGTTTCACAATATATGATTGATGCTGCCAAAGAGAATCCACAACTAG
CTCAGAAGCTTCATGATGTGTTACTTGAAAGTGGAGTTGTAGCTCCTCGAAATTTATTTTCGGAAGTCTATTCAGAGTCAATG

GAGGCAACAGGTGAAATCAAATCTGTGGCTGAATCCAATGATGAGAAAGGGAAAGATTTTGGAACAATCCAGCAAGGAAG
AAACCAGAGCAACCTTGGCCCTGTGAGGTTTTTGCCTCCTCTTCCAAGACCGCAATCTAAAGCAATTACCCATGACCTACGT
GAACATTCAGGATCTGGACTCGGTCATTTGTCTGAACATTGCAATATTGATGGTCACTCTGATTCCTCACATTCTGAAACATCT
ACTGACTATCCCAGAAATGTTCCTGTTGCCGTAGCTGCAGCGGCTGTTGTTGCGTCTTCCATGGTTGTTGCTGCCGCCAAGTC
AGCAAACTCGGATTCCTCCACTTTAGAACTTTCTGCTGCAGCTGCTGCTGCAGTTATGGCGACAGCTGCAGCAGTGAGCAG
GCAGTTTGAACTAGATTCACTTAGCAACGGGGATGCTGGTTCTGGTGGGCTTCATGGGGTAGATTCAGGAGGAGAACGAATA
TCCGACAGATCTATTGGCAATGAAAGTTCAAAATCTGATGCTGCAATTGATGATGTAGCTGAATGCGAGATTTTGTGGGAGG
AAATTACTGTGGCTGAGCGTATCGGACTGGGATCATACGGAGAGGTATATAGGGGAGATTGGCATGGAACTGCAGTGGCTGT
CAAGAAGTTCATTGACCAAGATATTACTGGAGAGGCACTGGAGGAATTCAGAAGTGAGGTCCGGATGATGAGAAGGCTCAG
ACACCCTAATATTGTTCTCTTCATGGGAGCTGTAACCCGCCCACCAAATCTCTCAATTGTTACAGAATTTCTTCCTCGAGGTA
GCTTGTATAGGTTAATTCACAGGCCTAATAACCAATTAGATGAGCGAAAGCGTCTGAGGATGGCTCTTGATGCTGCTCGTGGA
ATGAATTATTTACACAGCTGTAATCCGGTAATTGTCCATCGTGATCTTAAATCTCCAAACCTTCTAGTTGACAAAAATTGGGTT
GTCAAGGTGTGCGATTTTGGGTTGTCTAGAATGAAAGTAAGCACATACCTCTCTTCAAAGTCAACAGCAGGCACGGCTGAAT
GGATGGCTCCAGAAGTGCTTAGAAACGAACCCGCAGATGAGAAGTGTGATGTGTACAGCTACGGGGTCATTCTCTGGGAAC
TCTTTACGTTACAGCAACCATGGGGAAAAATGAACCCAATGCAAGTGGTTGGCGCGGTTGGTTTTCAACATCGACGACTTGA
TATCCCTGAATTTGTGGATCCTGGAATTGCAGATATCATCAGGAAATGTTGGCAGACGGATCCAAGGTTAAGACCAAGTTTTG
GAGAGATCATGGATTCTTTAAAGCAGCTACAGAAACCTATCCAGAGAGCAGCAGTTCCCAGTTCTTCTGCATTGACCACTGA
TGAGCAGGAACAATAA

>AtRAF5 (AT1G73660)

ATGAAAGTAAAAGAAGAAACTTTGAAGAATTTGGGAGATGGAGTGGTTTTAAGACCTGTTGATCATTGTTCTAGCATTTGGA
GTATGAAGATGAACATGAAGAACTTTCTTAAGAAACTTCATATCTCGCCCAATCAATCAGATGAAGCTGAAGGATCAATTTCA
ACAACTAAGAGCAATCATCATAAGTCTATCGATGTATCATCATCATCATCACCGAGGTCTCATCACAGCAATAGCCCTGAAATC
AAACCTTTTTCTGGTTTATCTAATTGGTTAAGTTCTGTTGGTCATAGAAAAATCCCTAGTCCTCCTAATTCTTTCAATGCCAAG
AACAGAGCCGCCACGGTTGATGACACTGTTGTTGTTAATGGGTCAGAACATGTGGATTTAGGTTCCAAAGATCCAGCTGTTG
AAGAAGAGAATCAGATACAGTTGGCTTTAGAGTTAAGTGCTAGAGAAGATCCTGAGGCTACTCAGATTGAGGCTATTAAGCA
ATTCAGTTTAGGCTCTTGTGCTCCTGAGAACTCTCCAGCTGAACTCATCGCTTATCGCTACTGGAATTACAATTGTCTTGGCTA
TGATGACAAGATCTTGGATGGTTTTTATGACTTGTATGGAGTGTTGAATGCATCCTCAGCAGAAAGAATACCTCCTTTGCTCG
ATCTTCAAGGGACACCTGTTTCAGACGGTGTGACATGGGAAGCTGTTCTTGTGAACAGAAGTGGGGATTCTAATCTGTTGAG
ACTTGAACAGATGGCTCTTGATATTGCTGCTAAATCAAGATCAGTTTCTTCCTCTGGTTTTGTGAATAGTGAATTGGTAAGGA
AACTGGCTATTTTAGTGGGAGATTACATGGGTGGACCAGTCGTGCACCCAGAGAGCATGTTGAGAGCTTGGAGGAGTCTTA
GCTATAGTTTGAAAGCAACTCTTGGAAGCATGGTTTTGCCACTTGGTTCTCTGACTATTGGTTTGGCTCGTCACCGAGCCTTG
TTATTCAAAGTATTGTGTGATAGTGTTGGTGTTCCTTGTCGAATAGTCAAAGGTCAGCAATATACCGGTTCTGAAGATGTGGC
AATGAACTTTATTAAGGCTGATGATGGCAGGGAGTACATTGTTGATCTTATGGGAGATCCCGGCACGCTTATTCCAGCTGATG
CAGCTGGACTACAAATAGACTATGATGAATCTGCCTATTCCGCTAGTCCTGGAGACAATGATTCAATTCATGTAGCTTCTTCCA
GCAATGGTATTGAAAGCTCATATGAAGAGAATACAGAGTTTCGAACAGGGGAACATCGTTCTAGTACCAAGAGTTCTGGGG
AGAGAAACCAATCCGGAGGTGGAGGCGATCTCATTGTTCATCCAAATATTTCTAGAGAAGATGTGAAAAATCAGAAGAAAG
TTGAAAAGGCTCCATTTCAAAATCTGTCTAGCAGGCCTATTCATTCTTTCACCCATATGAGATCACCTTCTTGGACTGAAGGG
GTTAGCTCCCCAGCTGCACAAAGGATGAAAGTCAAAGATGTTTCACAATATATGATTGATGCTGCTAAAGAGAATCCACGGT
TAGCTCAGAAGCTTCATGATGTATTACTTGAAAGCGGAGTTGTAGCTCCTCCCAATTTATTTTCCGAAGTCTATCCCCAGCAAT
TGGAGGCAACTGTTGAAAGCAAAAACTCGACTGAAGCCAAGAAAGAGAGAGGAAAAGATTTAGAGACAACTCAGGAAGG
AAGACACCAAAACGGTTTTGGTCCAGTGAGGTTTTTGCCTCCATTACCAAGAGTGCAATCTAAAACAAATGCACATGATCAA
CGTGATAATGGCAAAGTTGTAAGTCAGTCTGATTCTTCACATTCTGAAGCATCTTCGACAGAATATGCCAGAACCGTCCCTGC
TGCTGTAGCTGCAGCTGCTGTTGTTGCATCTTCCATGGTTGCTGCTGCTGCTGCCAAGTCTGCAAACTCAGACTCCTCCCCCA
TAGAACTTCCTGCTGCAGCTGCTGCCACGGCCACTGCTGCTGCAGTTGTGGCAACAGCTGCAGCCGTGTCCAGGCAACTTG

AATTAGGCTCGAATAGCGACGGGGATGATGGTTCTGGTGGGCATGAGCCTCAAGGTAGTGGGGACTCTAATCATGGGCCAAA
TTCAGGAGGGGAAAGAATATCTGACAAATCTATTGGCAATGAAAGTTCTAAGTCAGACTGTGATGATGTATCTGACTGTGAG
ATTTTGTGGGAAGAAATTACTGTGGGAGAACGTATTGGACTTGGATCTTATGGAGAAGTGTATCGGGGAGATTGGCACGGGA
CTGAAGTGGCTGTCAAGAAGTTCCTTGATCAAGATTTAACAGGAGAAGCATTGGAGGAATTCAGAAGTGAGGTCCGAATCA
TGAAAAAGCTAAGACATCCCAACATTGTTCTCTTCATGGGAGCTGTGACTCGCCCACCGAATCTTTCAATTGTTACAGAGTTT
CTTCCTAGAGGTAGCTTGTATAGGTTAATCCACCGGCCAAATAACCAATTAGACGAGAGGAGGCGCCTGAGAATGGCCCTTG
ATGCTGCTCGTGGAATGAACTATTTGCACAGCTGTAATCCGATGATTGTCCATCGCGATCTTAAGTCCCCAAACCTTCTAGTT
GACAAAAACTGGGTCGTGAAGGTGTGTGATTTTGGATTGTCTAGGATGAAACACAGTACATACCTCTCTTCAAAGTCAACAG
CAGGGACGGCTGAATGGATGGCTCCAGAAGTGCTAAGAAACGAACCTGCTGATGAGAAGTGCGATGTTTACAGCTACGGTG
TGATCTTATGGGAACTCTTTACGTTACAACAACCGTGGGGAAAGATGAACCCGATGCAAGTAGTTGGGGCAGTTGGGTTTCA
GCATCGACGTCTTGACATTCCCGACTTTGTAGATCCAGCAATTGCAGATCTCATCAGTAAATGCTGGCAGACGGATTCAAAGT
TAAGGCCAAGTTTTGCAGAGATAATGGCGTCTCTAAAGCGGCTACAGAAACCTGTAACAGGTTCCAACATCCCAAGACCAG
TCCCCAGTTCCTCTTCATTACCAACTGAACATGAACAAAAGGATTGA

>AtRAF6 (AT4G24480)

ATGCCTCACCGGACTACTTACTTCTTTCCGCGGCAGTTTCCCGATCGTGGATTCGATTCGTTTTCGCTTAAGAATGATCACGA
CAAGAAGAAATCGTCGTCTAACGTTGGAGAGAGTTTTGGATTTCAAAGAGATAATAAATCTAATGGAGTCGGAGAAGATTCT
AACAAGGAGAAGGAGAGTACTGTGTTCTCCTCTAATCCTCTGCTTTCTAAAAGCTCAGCCGTTTCAGATCTCTTTAGCGATGA
TCGCAAGTCGGAGAAGAAGCATCAGCAGCAACTAGCCGCTTTCTATGAGTGGTTGGCGGAGAAGAAGGCTAATCTCTCTAG
GTCATCATCTACTACTACTACTCATGGACGCGGGGTGAAGCCCACGCGCTTCTCTATGTCTAGCGACGCCGATGAGGAGAGA
GAGCTTCTGTTGTCTTCTCCGGCTGATCCCGCTCCGCTTCCTGCTACTTCTTCGCCGGATTCTATCATTGATTCGGCTAGGACG
GTTAACATACATGAACGAAACATTGATCGGAGCTTTGACAGAGAGGTTTCGCTTCCCCGAATGTCGAGTGAGAGCAGCTTCG
CTGGAAGCTTCTTCTCCGGGACGACGGTGGACGGGAATTTCTCCAATTTCTCAAGTCATACAGACGCAAGGGAAACTTCGA
CCACCACGCTTGTCTCAGTGAACAAAGAGGAAGAAGAAGTTGAGGTTAGGGAACAGGGCAAAGAGCAAAGCTTAGCGCA
GAAGTCAAGAGAAGGCTATTACTTGCAGGTCACGCTTGCTAAGTGGCTAAGTTCTCAAGCAAACCTAGCATGTGAGTCTGTT
CATATACAGAGCACAGAATCTATCTCCTACCGTTTTTGGGTAAGTGGTTGTTTATCATACAGTGACAAGATATCAGATGGATTT
TACAGTATATTAGGAATGGATCCGTATCTTTGGTTGATGTGTAACAATTCTGAGGATGGCAAACGAATTCCATCTCTTTTGTTA
CTCAAGGAGACTGAGCCGAATGATACATCAATGGAAGTGGTTTTGATAGATAGACGTGAGGACTCACGTCTTAAAGAACTG
GAGGATAAGGCACATGAGCTGTATTGTTCTTCAGACAACATGTTAGTGCTCGTCGAGAAACTTGGCAGACTTGTTGCGGTCT
ATATGGGGGGGAATTTCCAGGTGGAGCAAGGTGATCTCCAGAAACGATGGAAACTGGTCAGCAATAGACTCAAGGAATTTC
GGAAATGTATCATTCTTCCTATAGGTAGTCTAACAATGGGCCTTTGCCGGCATCGTGCCATCCTATTTAAGAAATTGGCTGATT
ACATAGGTTTACCATGTCGGATAGCTCGAGGTTGCAGATACTGTAAAGAGAGCCACCAATCTTCTTGCCTTGTCAAGATTGAC
GATGACAGGAAGCTTTCAAGGGAATATGTAGTTGACCTCATCGGGGAACCAGGAAATGTCCATGATCCGGATTCCTCTATCA
ACGGTGAAACACAGTGTCAGATTCCTTCACCTCTTCAAATGAGTCATCTTACAGATTTTTCCAGGCCTTGCGTGCATAGTACA
TCTCCTTGTCAGACTGTAGAGTCAAAGACTTCACGCACTCTTTCTGAAAACATTCAACGTTCAGGGAGTCAAGGCCAAGTA
CACAAAGAATTTGAGTTGCCTGATAACGCAGGGACAGTATGTTGTGCTCATATTGATCAAACTTGCTGTGCAAAAGTATCATC
AATGGTTTTGACAGAATCTGTTCTTCGAGCTCTACCACTTGATATACCAAACCTTAGTGAAGAAAAGATTGCCCCACAAGAA
ACCTGCAAAGAAGAAACCGTTCTATTAGAAGATCCAACAGCTATGAAGCAGCCAAACTTATCAGTTGAACCAGAGATAGTA
GAGGCTGACACTCGAAAAGATAAAAAAGGAAGGTTACCTGTTGACGCCATCTCACCTTACTTGACTATTGAACCTTCTTTGG
CATCAGATTGGCTGGAGGTCTCATGGAATGAATTACATATCAAAGAGCGTGTGGGTGCTGGATCATTTGGAACTGTTCATCGT
GCTGAGTGGCATGGATCAGATGTTGCTGTCAAGATTTTGTCTATTCAAGATTTCCATGATGACCAATTCAGAGAATTTCTCAG
AGAGGTATGTAAGCAAGCGGTTGCTATAATGAAACGTGTTCGTCACCCAAATGTTGTTCTCTTCATGGGTGCTGTGACAGAG
CGACCCCGGTTATCAATAATAACAGAATATTTGCCAAGAGGCAGTCTTTTTCGCCTTATCCATAGGCCAGCTTCTGGGGAGTT
GCTAGATCAGAGGAGGAGGCTACGTATGGCATTGGATGTGGCCAAGGGGCTCAACTACCTACACTGTCTTAATCCTCCTGTA
GTGCATTGGGACCTGAAATCTCCAAATCTACTGGTTGATAAGAACTGGACAGTGAAGGTTTGCGATTTTGGACTTTCAAGAT

TCAAGGCAAACACTTTCATACCATCAAAATCTGTTGCAGGAACACCTGAGTGGATGGCTCCAGAGTTTCTTAGAGGGGAAC
CGACAAACGAGAAATCAGATGTTTACAGTTTCGGAGTAGTCTTATGGGAGTTGATTACTTTGCAACAGCCTTGGAATGGACT
CAGTCCTGCTCAGGTGGTTGGAGCAGTTGCATTCCAGAACAGGCGGCTTATAATTCCTCCCAACACCTCTCCGGTTTTGGTAT
CTCTAATGGAAGCTTGCTGGGCAGATGAGCCGTCTCAGCGGCCAGCATTTGGTAGTATAGTGGACACATTGAAGAAGCTACT
AAAGTCTCCGGTGCAGCTGATCCAAATGGGTGGAGACAAAGGGGTAATTCCAACTAAATCAGCTCCCATACTATAG

>AtRAF7 (AT3G06620)

ATGGAGAATCCACCTGCGGAGGAGCTGCTGAAGAAGATACTTGAGCTAGAGGAGAGTCAGGAGCATCTCAAGCAGGAGAT
GTCTAGGCTTAAGGTATCTACGGAGCTTAGACAGCGATCGCATTCGGTGTCTCCTCATCGTCCGGCGAGGAGAAACATCGGA
GAGGGAGCTCCGTCTTGGAGGAAAAGCGGTGCCGCCTCGTTTCGTAACGCGTCGCCGTTGCGGAAGGAGAGCCGTATCCA
GAATTCGATGAGATTGAGGTCTGAAGTTGGTGGCGGGGGACCATCTGCTGGGAAATTCACTGATAAACAGTATTTGAATATTT
TGCAGTCGATGGCACAAGCTGTTCATGCCTTCGATCTTAATATGCGGATTATCTTTTGGAATGCTATGGCGGAAAAGGTTTATG
GGTACTCTGCAGCAGAAGCACTTGGGGAGAACCCTATTAATGTTATTGCAGATGATCGGGACGCTGCCTTTGCAATGAATATT
GCTCGACGTTGTGTCCGTGGAGAGAGCTGGACTGGTGAGTTTCCTGTTAAGAGCAAATCAGGGGATAGATTTTCAGCTGTC
ACTACCTGTTCCCCTTTCTATGATGATGATGGTGCTCTTATGGGGATCATTTGTATCACCAGTAACACGGCACCGTATCTGAAC
CCAAGAATCTCTTTGGCTAAATTGAAGGCGCAAGAAGAAGGTGAAACGAGCTCTATCCCTGCAAGGAATAGTTTTGCCTCTA
AACTTGGCTTGGATTCCAGAGGAGCTGTTATATCGAAACTTGGCCTTGACTCTGATCAGCCTATACAAGTTGCTATAGCATCA
AAGATCTCAGATTTGGCATCCAAGGTCAGCAACAAGGTCAGGTCGAAAATGCGAGCAGGTGATAATAGTGCCACACTCTCG
GAGGGTGGCAGTGGGGATAGTCATCAAAAAGATCATAATGTCTTCGGTGCTACTCTTGTTGACCACAGGGACGATGCAGCAT
CAAGTGGTGCCAGCACACCAAGAGGGGATTTTATCCAGTCTCCTTTTGGTGTATTCACATGTAATGATGAGAAGTTTGTTTCG
AAACCCTTCAAAGATTCCAGTGATGAGAGTGATGGAAAGCCTGCAATCCATAAGGTTCTCACCTCAAAAGCTGAAGAGTGG
ATGGTAAAGAAAGGTTTGTCATGGCCATGGAAAGGGAATGAGCAGGAAGGTTCAAAAGGAAGACCTACAAATTCTGTTTGG
CCTTGGGTACAGAATGAACAAAAGAAAGAGAGGTGTCACCAGATTAATCCCTCTGCTGGTGTTCAGTATGAAAGCCATGCCT
TTGAAAGTAATAAGCCTATCAATAATGAGGCTTCTAGTTTGTGGTCTTCCCCTATAAATGCAAACAGTACAAGCAGCGCTAGT
AGCTGCGGAAGTACCAGCAGCAGTGTTATGAACAAGGTTGATACTGATAGCGAAGGCTTGGAATATGAAATTTTATGGGATG
ATTTGACAATTGGAGAACAAGTCGGACAAGGTTCATGTGGAACTGTTTATCACGGTCTCTGGTTTGGATCTGATGTAGCTGTA
AAAGTGTTCTCCAAACAAGAATATTCAGCAGAGGTCATAGAATCTTTTAAACAAGAGGTATTGTTGATGAAAAGACTTAGAC
ACCCTAACGTCCTGCTGTTTATGGGAGCTGTGACTTCACCCCAGCGTCTCTGTATAGTGTCAGAGTTCCTTCCACGTGGAAGT
CTCTTCCGTCTACTACAGAAGAGCACGTCAAAACTGGATTGGAGGCGGCGTATCCATATGGCCTTGGACATTGCTCGCGGTA
TGAATTATCTTCACCATTGTAGTCCACCCATTATCCATCGTGATCTGAAGTCATCAAATCTGCTGGTAGACAAGAACTGGACC
GTTAAGGTAGCTGACTTTGGTCTCTCGCGTATCAAGCATGAAACATACCTAACCAGCAAGTCTGGGAAGGGAACGCCTCAAT
GGATGGCACCAGAAGTTCTTCGAAATGAGTCTGCTGATGAGAAATCTGACATTTACAGCTTTGGAGTAGTATTATGGGAGCT
TGCCACCGAGAAGATCCCATGGGAAACTCTCAACTCTATGCAGGTGATTGGAGCTGTGGGGTTCATGGACCAGAGGCTGGA
AATCCCAAAAGACATTGATCCTCGTTGGATCTCATTAATGGAGAGCTGTTGGCACAGTGATACAAAGCTGAGACCCACATTC
CAAGAACTGATGGATAAATTAAGAGACCTGCAAAGAAAGTATATGATACAGTTCCAAGCGACTCGTGCTGCGTTATCTGACA
ATTCTCTTCTCAAGGACAACTAA

>AtRAF8 (AT3G06630)

ATGGAGGAGGAGCTGCTCAAGAAGATGCTTGAGCTAGAGCAAAGTCAGGAGCTTCTAAAGCAGGAGATGTCTAGGCTCAA
GCTCTCCACGGAGCTTAGGCAGCCGTCGCATCCGGTGTTGCCGCGTCGGCCGTTGAGACGTATCCAGGGATCGATGAATTCG
AATCCATCGCCCGGGAAGTTCACTGATAAACAGTATTTGAATATTTTGCAGTCGTTGGCTCAGTCTGTTCATGTCTTAGATCTC
AATACGCGAATTATCTTTTGGAATGCCATGTCGGAAAAGCTTTATGGGTACTCTGCTGCAGAAGTAGTTGGGCGGAACCCAG
TACATGTTATTGTAGATGATCAGAATGCTGCCTTTGCCTTGAATGTTGCTCGACGTTGTGCCAATGGAGAAAGCTGGACAGGG
GAGTTTCCTGTCAAGACCAAATCAGGAAAGATCTTTTCAGCTGTCACTACGTGTTCTCCCTTTTATGATGATAACGGCACTGT
TGTTGGTATCATTTCTATCACAAGTGACATTGCACCCTATCTCAACCCGAGACTCTCTTTGCCTAGATTGAAGCCGCAAGAAC
CTGAGAGGAAACTTGGTTTGGACTCCAAAGGAGCTGTTATATCGAAACCTGGCCTCGACTCTGATCAGCCTATACAAGTTTC

TATAGCATCAAAGATATCAAGTTTGGCATCCAAGTTGAGCAACAAAGTCAGGTCAAAAATGCGAGCAGGTGATAACAGTGC
CTGTGGGGATAGTCATCATTCAGATCATGATGTATTCGGTGATACTCTCTCTGACCACAGGGACGACGCAGCGTCAAGTGGTG
CTAGCACACCAAGAGGGGATTTTATCCAATCTCCTTTTGGTGTTTTCACATGTTACGATGACAAGTTTCCTTCAAAACCCTCC
AAAGATTCCAGTGATAGAAAGCCTGCAATCCATAAGGTTCCCACCTCAAAAGCTGAAGAATGGATGGTAAAGAAAGGTTTG
TCACGTCCATGGAAAGGGAATGAGCAAGAAGGTTCAAGAGTAAGGCCTACTCATTCTGTATGGTCTTGGGTAGAAAATGAA
CAAGAGAAAGATAAGTACCACCAGATTTATCCCTCTGCTGGTGTTAAGTCTGAAAGCCATGGCTCTGAAAGTAATAAGCCTA
CTGATGACGAAGCTTCTAATATGTGGTCTTCCTCTATAAATGCAAACAGCACAAACAGCGCTAGTAGTTGCGGCAGTACCAGT
AGGAGTGTTATGGACAAGGTTGATATAGATAGCGATCCCTTGGAACATGAAATTTTATGGGATGATTTGACAATCGGGGAACA
AATTGGACGAGGTTCATGTGGAACTGTCTATCACGGTATCTGGTTTGGATCTGATGTAGCTGTGAAAGTGTTCTCCAAACAA
GAATATTCAGAATCAGTCATAAAGTCTTTTGAAAAAGAGGTATCGCTGATGAAAAGACTTCGACATCCTAACGTTCTGCTGTT
TATGGGAGCTGTGACTTCACCCCAGCGTCTCTGTATAGTGTCAGAGTTCGTTCCACGTGGAAGTCTCTTCCGTCTACTGCAG
AGAAGTATGTCGAAACTTGATTGGAGGCGACGTATCAACATGGCCTTGGACATTGCTCGCGGTATGAATTATCTTCACTGTTG
TAGTCCACCCATCATCCACCGTGATCTGAAGTCGTCAAATTTGCTGGTAGACAGGAACTGGACCGTTAAGGTAGCTGACTTT
GGTCTTTCGCGTATTAAGCATCAGACATACCTAACTAGCAAGTCGGGAAAGGGAACGCCTCAATGGATGGCACCTGAAGTTC
TTCGAAATGAGTCTGCTGATGAGAAGTCTGACATTTATAGCTTTGGAGTAGTATTATGGGAGCTTGCCACAGAGAAGATCCCA
TGGGAAAATCTCAACTCGATGCAGGTGATCGGAGCTGTGGGGTTCATGAACCAGAGGCTGGAAATTCCAAAGGACACTGAT
CCTGATTGGATCTCATTAATAGAGAGCTGTTGGCACAGGTAA

>AtRAF9 (AT3G06640)

ATGGTTAAGCTTCTTCAAGACCCGATTACACCTAACAAGGAGCTGCTGAAGAAGATGATAGAGCTAGAGAAAAGTCAGGAG
CATCTAATGCAGGAGATGTCTCGGCTCAAGGTCTCCACGGAGCTTCGGAAGGAGAGCCGTATCCAGTGTTCCATGAATCTGA
GACCATCGCCCTGGAAGTTCACTCATAAACAGTATCTCAATATTTTGCAGTCCATGGCACAATCTGTTCATGCCTTCGATCTCA
ATATGCGAATTATCTTTTGGAATGCTATGGCGGAAAAGGTTTATGGGTACTCTGCTGCAGAAGCAGTTGGACAGAACCCTATT
GACGTTATGGTAGATGATCGGGATGCTCCCTTTGCCATGACTATTGCTCAACTTTGTTCCAATGGAGAGAGCTGGACTGGCAA
GTTTCCTGTCAAGCGCAGAACAGGAGAGAAATTCTCAGCTGTCACTACGTGTTCCCCCTTCTATGCTGATGACGGTTCTCTTA
TTGGGATCGTTTCTATCACAAGTGACGTAGCACCATATCTGAACCCAACAATCTCTTTGGCTAAATTAAAGGCTTCAGAAGTC
GAAACCAGCTCTACCCCTGCAAGGAATAGTTTTGCCTTTAAACTTGGGTTGGACACCAAAGGAGCTGTTGTATCGAAACTTG
GCCTTGATTCTGATCAGCCTATACAAGTTGCTATAGCATCAAAGATATCTGATTTGGCATCCAAGGTGAGAAACAAGGTCAGG
TCGAAAATGCCAGCAGGTGATAGTAGTGTCACAGTATTTGAGGGTGAAACTGGGGATAGTCATCATTCAGATCATGGAGTCT
TCGGTGCTACTCTCTCTGACCACATGGATGATGCAGCATCAAGTGGTGCTAGCACACCAAGAGGGGACTTTATCCAATCTCC
TTTTGGTGTATTCACATGTAACGATGACAAGTTTTCTTCCGAACCCTTCATAGATTCCAGTGATGGATATCCTATAACCCTTTTC
ACCTCAAAAGCTGAAGAATGGATGGTAAAGAAAGGTTTGTCTTGGCCATGGAAAGGGAATGAGCAGGAAGGTTCAAGAGT
AAAGCCTACTTATTCTGTATGGCCTTGTGTACAGAATGAACAAAAGAAAGATAAGTCTCACCAGATCAACCGCTATTCTGGT
GTTAAGTCTAAAAGCCATGCCTCTGAAAGTAATAAGCCTACCAATAACAAGGCTTCTGGTTTGCGGTCTTCTTGTATAAATGC
GAACAGCGCTATTAGCCGTGGAATTATCAGTCACAGTACTATGAACAAGGTGGATACAAATAGTAATTGCTTGGAGTATGAAA
TTTTGTGGGATGATTTGACAATCGGAGAACAAATCGGACAAGGCTCATGTGGAACTGTCTATCACGGTCTTTGGTTTGGATCT
GATGTAGCTGTAAAATTGATCTCCAAACAAGAATATTCAGAAGAGGTCATACAATCTTTTAGACAAGAGGTATCGTTGATGCA
AAGACTTAGACATCCTAACGTTCTGCTGTTTATGGGAGCTGTGACTTTACCTCAGGGTCTCTGTATAGTGTCAGAGTTCCTTC
CACGTGGAAGTCTATTTCGTCTACTGCAGAGAAATATGTCAAAACTTGATTGGAGGCGGCGTATCAATATGGCCTTGGACATT
GCTCGCGGCATGAATTATCTTCACCGCTGTAGTCCCCCCATCATCCATCGTGATTTGAAGTCATCAAATCTGCTGGTAGACAA
GAACTTGACCGTTAAGGTAGCTGACTTTGGTCTATCGCGTATTAAGCATCATACATACCTAACCAGCAAGTCGGGAAAGGGA
ATGCCTCAATGGATGGCACCAGAAGTTCTTCGAAATGAGTCTGCTGATGAGAAGTCTGACATTTACAGCTTTGGAGTAGTAC
TATGGGAGCTTGCCACAGAGAAGATCCCATGGGAAAATCTCAACTCGATGCAGGTGATCGGAGCTGTGGGGTTCATGAACC
AGAGGCTGGAAATTCCAAAGGACATTGATCCTGATTGGATCTCACTAATAGAGAGCTGTTGGCACAGGGATGCAAAGCTGA
GACCCACATTCCAAGAACTGATGGAGAGACTAAGAGACCTGCAAAGAAAGTATACAATACAGTTCCAAGCGACTCGTTGGT

TGACTATGGTTCGAACGGAGTCTTCCCAGGTACTGAAACAAAATCATTGA

>AtRAF10 (AT5G49470)

ATGGAGAAGACGACTCCACCAGCGGAGGAGCTTCTAAAGAAGATACGAGAGTTAGAAGAAAGCCAAGAGCATCTAAAACG

AGAGATGTCGAGACTTAAAGTATCGGCGGAGATGAAGCAGCGTTCGCATTCAGCATCGCCGCAACGTCCTGTGAGGAGAAA

CAGTAACGACGGAACTCCGATGTGGAGAAAAACCGGTGCGGCTTCGTTTCGTCACGCTTCTCCGTTGCGTAAGGAGAGCCA

TGCGAAAGTTGCCGGCGGCGGAGGCGAGGGGCAATCGGCAGGCAAGTTCACCGATAAACAGTATTTGAATATTTTGCAGTC

TATGGCTCAAGCTGTTCATGTCTTCGATCTAAATGGCCAAATTATCTTTTGGAACTCAATGGCGGAGAAGCTTTATGGATTCTC

AGCTTCAGAAGCACTGGGGAAGGATCCGATTGATATTCTTGTAGATGTTCAGGATGCTTCGGTTGCACAGAATATCACTCGGC

GTTGCAGTAGTGGAGAGAGTTGGACTGGTGAGTTTCCTGTAAAGAACAAAGCTGGAGAGAGGTTTTCAGTTGTGACTACGA

TGTCTCCTTCTTATGATGATGATGGTTGTCTTATTGGGATCATATGTATCACAAATGATTCGGCACTCTTTCAAGACCCGAGAG

GCTCTCCGGCTAAGACAAGGAGGGGGCAAGAAGGTGAAACGAGCTTTAGTCGGGTGACTAGTAGTGTTGCATCTAAGCTTG

GTCTTGACTCGAAAGAGGCGGTTGTATCAAAACTTGGCCTTGATTCTCAGCAGCCTATACAAGTTGCTATAGCTTCAAAGATA

TCAGATTTGGTGGGCAACAAGGTCAAGTCGAAAATGCGAGCAGGTGATAATAACGCTGCAAACCTTGAGGGTGGAAGTGG

GGACAGTCATCAATCTGATCAGGGTTTCTTTGATGCTGCTTTCGCTGACCGGAGGGAGGATGCAGCAACAAGTGGTGCTGAT

ACACCTAGGGGGGATTTTATCCAATCTCCTTTTGGTGTATTCTTACGTAGCGATGAGAAAGCTTCTACAAAGCCCTTCAGAGA

TTCCAGTGATGAAAGTGATGGAAACTCTGTTGTACCTAAAACACTGACCTCAAAAGCTGAAGAGTGGATGGTAAAGAAAGG

ATTATCATGGCCATGGAAAGGGAATGAAAGGGAAGGTTTGGAAGGAAGGCGTAGTCATTCTGTGTGGCCTTGGGTGCGGAA

TGAACAACAAAAACAACAGGCTTATCAAAGCAATTCTAATCACAGTGTTAAGTCTGAAAGCCAGGCATGTGAAAGTATTAA

GGCTTCCAGTAACGAGCCTATGGGGTATTGGTCTTCCTCTGTTAATGTGAACAGCACAAGCAGTAGTAGCAGCTGTGGAAGT

ACCAGCAGCAGTGTTATGAACAAGGTCGATATGGATAGTGACTGCTTGGATTATGAAATCTTATGGGAAGATTTGACAATTGG

AGAACAAATCGGGCAAGGTTCATGTGGAACTGTCTATCACGGTCTATGGTTTGGATCCGATGTGGCTGTAAAGGTGTTTTCC

AAACAAGAATATTCAGAAGAGATCATAACATCTTTTAGACAAGAGGTATCGTTGATGAAAAGACTTAGACATCCTAACGTTTT

ACTGTTTATGGGAGCCGTGACATCACCTCAGCGTCTCTGTATAGTGACAGAGTTCCTTCCACGGTTTGGTCTTATTACTTTGG

CTAATATTACACTTCCTTTTGTGCTATTCGAACTCCGGGGCCAAAACTCTGACTTACCCTATCAAATAACATTTGCAGTGGAA

GTCTCTTCCGTTTGCTGCAGAGGAACACGTCAAAACTGGATTGGAGGCGACGTATCCATATGGCCTCGGATATTCTATCTTGC

GTATTATTCTTTGGACTCTTTAATTCAGTTACTGTATCTTGTCTACAACATGTTACACATTTTTCTTACATACTTCTTCGCACAG

GCTCGTGGCATGAATTATCTTCACCATTGTACTCCACCGATCATTCACCGAGATTTGAAGTCATCAAATCTACTGGTTGATAAA

AACTGGACCGTGAAGGTTGCTGACTTTGGTCTTTCGCGTATCAAGCATGAGACGTACCTCACTACAAAGACAGGAAGGGGA

ACGCCTCAATGGATGGCACCGGAAGTTCTCCGAAATGAGGCTGCTGATGAAAAGTCTGACGTTTACAGCTTTGGAGTAATAC

TATGGGAGCTGGTGACTGAGAAGATCCCGTGGGAAAGTCTAAATGCTATGCAGGTGATTGGAGCTGTAGGGTTCATGAACCA

GAGACTGGAAGTCCCGAAAAATGTTGATCCTCAGTGGATTTCTTTAATGGAAAGCTGCTGGCATAGTGAACCGCAGGACAG

ACCATCGTTCCAAGAAATTATGGAGAAACTAAGAGAGCTTCAAAGAAAGTACACTATACAGTTTCAGGCGGCTCGTGCTGCA

TCAATAGAAAACTCTGCCCTCAAGGAAAAATAA

>AtRAF11 (AT1G67890)

ATGGAGAATCCGAATCCACCAGCAGAGAAGCTATTGAAGAAGATACGAGAGCTAGAAGAAAGCCAAGAAGATCTAAAGCG

AGAGATGTCAAAACTCAAAGTATCAGCAGAGATTAAAAGGCGTTCGCATTCTTCATCACCTAAACGACCTTCAAGGAGAAA

CAGTGGAGAAGGAACTCCGTTATGGAGAAAAACCGGCGCCGCATCGTTCCGTCACGCTTCTCCGTTACGGAAAGAGAGTCA

TTCTAAAGACGGAGTAGCTGGTGGTGGTGATGGACCATCGGCAGGCAAGTTCACCGATAAACAGTATTTGAATATTTTGCAG

TCTATGGCTCAAGCTGTTCATGTCTTCGATCTTAATGGCCAAATTATCTTTTGGAACTCGATGGCGGAGAAGCTTTATGGATTC

TCAGCTGCAGAAGCGTTAGGGAAGGACTCGATTAATATACTCGTAGATGGTCAAGATGCTGCTGTTGCAAAAAATATCTTTCA

GCGTTGCAGCAGTGGAGAGAGTTGGACTGGAGAGTTTCCTGTTAAGAATAAAATGGGAGAGAGGTTTTCGGTTGTGACTAC

GATTTCTCCTTTTTATGATGATGATGGTTTGCTTATTGGGATTATATGTATCACGAATGATTCGGCACTTTTTCAACGGCCGAGA

GTTCCTCCCGCTAAAAACAGGTGGCAAGAAGGGGATTCAAGCTTTTGCCGGGGGACTAATGGTGTTGCTTCTAGGCTTGGTT

TTGATTCCAAAGAAGCTGTTGTGTCGAAACTTGGCCTTGATTCTCAGCAGCCTATTCAAGCTGCTATAGCATCTAAGATCTCA

GATTTGGCATCCAAGGTGGGCAACAAGGTCAGGTCGAAAATGCGAGCAGGTGATAATAACGCCTCACACCCTGAGGGTGGA
AATGGGGGCAGTCATCAGTCTGATCAGGGTTTCTTCGATGCTGCTTTCTCTGACCAGAGGGAAGATGCAGAAACAAATGATG
CTAGCACACCTAGGGGGAATTTGATCCAGTCTCCTTTTGGTGTGTTCTTATGTAACGATGATAAGTCTTCTTCAAAAGCCTCC
GGAGAATCCAATGATGAAAATGATAGAAACTCTGTTGTCCCTAAGAAACTTACCTCAAAGACTGAAGAGTGGATGGTAAAG
AAAGGACTGTCATGGCCATGGAAAGGAAATGAGCGGGAAGGTTTGGAAAGAAGAAATGCTCATTCTGTGTGGCCTTGGGT
GCATAATGAACAACAAAAAGAAGAGGCTCATCATAGTAATTCCTATAACAGTGTTAAGTCTGAAAGCCTGGCAAGTGAAAGT
AATAAACCTGCCAATAATGAGAATATGGGTTCTGTTAATGTGAATAGCGCAAGCAGTGCTAGCAGCTGTGGAAGTACCAGCA
GCAGTGTTATGAACAAGGTTGACATGGATAGCGACTGCTTAGACTATGAAATCTTATGGGAGGATTTGACAATTGGAGAACA
AATCGGGCAAGGTTCATGTGGAACTGTCTATCATGGTCTATGGTTTGGATCTGATGTGGCTGTAAAGGTGTTTTCCAAACAAG
AATATTCAGAAGAGATCATAACATCTTTCAAACAAGAGGTATCATTGATGAAAAGGCTTAGACATCCAAACGTTCTGCTGTTT
ATGGGAGCTGTGGCATCACCTCAGCGTCTTTGTATAGTGACAGAGTTTCTACCACGTGGAAGTCTCTTTCGTTTGCTGCAGA
GGAACAAGTCAAAACTAGATTTGAGGCGACGTATCCATATGGCATCAGACATTGCTCGTGGCATGAATTATCTTCACCACTGT
AGTCCTCCCATCATTCACCGTGATCTGAAGTCGTCAAATCTACTGGTTGATCGGAACTGGACTGTGAAGGTTGCTGATTTTGG
TCTTTCTCGCATCAAGCATGAGACGTACCTCACTACAAACGGAAGGGGAACGCCTCAATGGATGGCACCAGAAGTTCTTCG
AAATGAGGCTGCTGATGAAAAGTCTGACGTTTACAGTTTTGGAGTAGTACTATGGGAGCTTGTGACAGAGAAGATCCCATGG
GAAAATCTAAATGCTATGCAGGTGATTGGAGCCGTGGGGTTCATGAACCAGAGGCTGGAAGTCCCAAAAGACGTTGACCCT
CAGTGGATTGCTTTAATGGAAAGCTGCTGGCACAGCGAACCGCAATGTAGACCATCGTTCCAAGAATTGATGGACAAATTAA
GAGAGCTTCAAAGAAAGTATACTATACAGTTCCAAGCAGCTCGTGCTGCATCGATAGACAACTCTTCTCTCAAGGAAAAATA
A

>AtRAF12 (AT4G23050)

ATGGCCGGAAACAACTCGGAATCGAGTCTCTACCAGGTTTTGGTGGAGTGGTGTCAGAGGATGGAGACGAGTCAAGCGAG
ACTAAGGGAGGATGTTGATGACTTATTGCTTCAGGAGGAGAGTCGAACCGGTAAGGAATCGGCGACGGGATTGGAGACGGA
CACGGAGAAAGAGGCTGAGGTAGAGGTGGAAGCGGAGGCGGCGGATTCCTGGGATAATCCGACGGCGACTTGGGAGAGA
GCTGTTTCAGGATTCTATTTCGCTGATAGTGCGTATCGGACTTTGATGGATTCAATGGGACACGCGATTCATGTAACATCAGCT
GCGTCTGGAGAAATTACATTCTGGAGTCGTTCTGCTGAGAATCTGTACCATTGGTATGCTGAGGAAGTTGTTGGCTACAGAA
CTATTGATGTGCTTGTAACCGAAGAATACCGTAATTCCCTTACTGGTATCAGGAATAGGGTGTGCCGTGGAGAGACTTGGACT
GGTCAGTTTCCGTTTCAGAAGAAAACTGGTGAACTATTTATGGCTTTGGTGACTAAAAGCCCTGTGTATGAAAACGGTGAGC
TGGTTGGTGTTGTTACTGTCTCGAGCGATGCAACCCTTTTCAATAGGATGCATCCACTAAGTAATGAGCATCAACAACAAGCT
CGTAGTAATAATAGACATGAATCAAACTTGAGAAAACATCAGTGGCACCTCCCGAGGCCTCAGATCGCTGCAGCATCACAGG
TTCCAGTTGTCCCACAATATTCATCAGCTGTTGCTTCAAACCTGAAGGCGTCGAAGCTTCTACCACAGAGAAATGGAGATGA
TTCCTTCAATGGAAATCATAATTCGAGGAGCCGAGATGAAAATGTTCCGGTTGTTGCCTCCACAACTTTCGAGAAGTATGGC
TCACTGGCGGATAAGTTTTTGGGGAAGCTGCAAAGGAAGATTACTGGCAGCCAAGGAACCGAAGATAATGAGCCTATCTTA
AGAAACGGTATCAATAAGTCAGCATGTGGCAGTGGAGGTAGTTCTAAGGCTTCTAATGCAGTTACCTGCACGGCTTTCAGAG
ACAATGGCAATGGAAAACCAAAAAGGGCTGAAGTTAGAATTTCAGATGTCTATGGAAATGGAGCTGAGGGTTTGATACATAA
TGGAGATCGCTTTCAGTATATTGGGAACCTGGGACAAAGTAAACCTCCCAGAGGACTAGAGAGTGGTTTGGTGTCTGGCATG
CGGGGAACAAAAATGTCGGACCTAAATGGTGAGATAGAAGATGCTTGGAATACTCGTCTTAGTGTTGATCCCTTGCCAATTC
TAGGAGTCAACAGCGGTAGGCAACAAAGTCCTGTCAACCAAAGGAATAATAGATTAGTTACTGATTCGTCATGTGAGATACG
ATGGGAAGATCTACAACTTGGGGAGGAGGTCGGAAGAGGTTCATTTGCTGCGGTTCATCGTGGAGTTTGGAATGGATCGGAT
GTTGCTATTAAGGTTTACTTCGATGGTGATTACAATGCGATGACTTTGACGGAGTGCAAAAAGGAGATCAACATTATGAAGA
AACTGAGACATCCGAATGTGCTACTATTTATGGGAGCAGTATGTACAGAAGAAAAATCTGCCATAATCATGGAATATATGCCA
AGAGGGAGTCTCTTCAAAATACTTCATAATACGAATCAGCCATTGGACAAGAAACGCCGTTTAAGAATGGCCCTTGATGTTG
CTAGGGGAATGAATTACTTACACCGCAGAAATCCGCCAATTGTACATAGAGACTTGAAATCTTCCAATCTACTCGTGGACAA
GAACTGGAATGTCAAGGTTGGAGACTTTGGGTTATCAAAGTGGAAGAACGCAACCTTCTTGAGTACTAAATCCGGGAAAGG
AACTCCGCAGTGGATGGCTCCTGAAGTTCTCAGAAGTGAACCTTCGAATGAGAAGTGTGATGTGTTCAGCTTTGGAGTCATC

TTATGGGAGCTAATGACTACGTTAGTACCATGGGACCGTTTGAACTCTATTCAGGTTGTTGGAGTTGTTGGTTTCATGGATCG
ACGATTAGACTTACCTGAAGGATTAAATCCCCGGATCGCATCCATAATACAGGATTGTTGGCAAACTGATCCAGCAAAACGA
CCGTCGTTCGAGGAATTAATTAGTCAGATGATGAGCCTGTTCCGCAAACCAGGGTCAGGTGCGCAAGAAGAAGACGATTGA

>AtRAF13 (AT2G31010)

ATGGAAGAGAGACGAGATGATGAATCAAGCCCTACACACCAGGGATCAGAACTTGCGGAAAGGGTGAAGTTGCTTTCTTTT
GAATCTCAAGGTGAAGCTTTGAGTAAGGACTCCCCTAGAAGTGTTGAACAGGATTGCTCTCCAGGTCAAAGAGCATCACAG
CATCTTTGGGATACTGGAATACTCTCTGAACCTATTCCTAATGGTTTCTACTCAGTTGTTCCGGATAAGAGAGTAAAGGAGCTA
TATAATAGACTACCTACTCCGAGTGAGCTTCATGCTTTGGGAGAGGAAGGTGTCAGAATTGAAGTTATTCTTGTTGATTTTCA
AAAAGATAAGAAGCTCGCAATGCTTAAACAGTTGATCACTACACTTGTCAGCGGCAGCGGCACAAATCCTGCTTTGGTGATT
AAGAAAATAGCAGGAACGGTCTCTGACTTTTACAAACGTCCAACACTAGAAAGCCCTTCAAAACTTGCTCTAGAGGAAAAT
GCGTTCTTGTTTGAAAACCATGGTGCTCAGTTGCTTGGCCAGATTAAGCGTGGTTGCTGTCGAGCTCGGGCAATTTTGTTCA
AAGTTCTAGCTGATACTGTAGGACTTGAAAGTCGGCTGGTGGTGGGTTTGCCTAGTGATGGGACTGTGAATTGCATGGACTC
TAACAAACACATGTCTGTTATAGTTGTGCTGAATTCCGTTGAACTACTAGTCGATCTTATTCGGTTTCCTGGTCAGTTGGTACC
GCGATCAGCCAAGGCAATTTTCATGTCACACATCTCGCCTGCTGGAGAGAGTGATTCTGCAGAAAACGACTCTTGTGACTCA
CCTTTGGAGCCAAATAGTCCTTTATATGAGAGAAGAGATCCTGAGAGTACAGAAAAAGATGAAAATCTTCAATTTTATCGGA
AGTTAGAAGGATATCCAAATGCATCTGGTTCATCACTGCGTAGTTTGATGCTCAGACCTTCTACAGCTATTGAAAGGAAATTG
AGCAACACCTCGCATAGTGAACCAAATGTTGCTACTGTATTCTGGAGGCGTAGCCGCAGAAAAGTAATTGCTGAACAGAGA
ACAGCTAGCTCAAGCCCAGAACACCCATCTATGCGACGTGGACGATCAATGTTGAGCACTGGTAGGAATTCTTTTAGGGATT
ACACTGGTGAAGCATCATCTCCTTCAAGTTCATCCACATCAGAAATTCGTAAAACTAGACGGCGAAGTTTTAGGATCACACC
AGAAATTGGCGATGACATTGCAAGCGCTGTACGAGAAATGTATGAGAAATCAAAGCAAAACCGTCTCTTGCAGGGTCGAGA
GGATGAAAACAGCTCAGTTATCGATAACAACGTGTCTGGTCTCCATCTTGATGATGAGTTAAATTCTAAGAAGACAATGTCAT
TGCCCTCATCTCCCCATGCTTACAGGTGTCAAACATTTGGACGAAGAGGGCCTTCAGAATTTGCCGTGAAGGATACATGGAA
TAAAGTGGTTGAGTCTTCCACATTGCAGAATCAGCCTTTATTACCCTATCAAGAATGGGACATTGACTTCTCAGAGCTGACTG
TTGGAACTCGGGTGGGGATTGGTTTTTTTGGTGAAGTTTTTCGTGGAGTATGGAATGGGACAGATGTTGCAATCAAATTGTTT
CTGGAGCAAGATCTTACTGCCGAAAACATGGAAGATTTCTGCAATGAGATATCAATTCTCAGCCGTGTTCGCCACCCAAATG
TTGTACTATTTTTGGGTGCATGCACAAAACCTCCACGCTTATCCATGATCACGGAGTACATGGAGCTGGGATCGTTGTATTATT
TGATCCACATGAGTGGTCAGAAGAAGAAACTTAGCTGGCACAGAAGGCTCAGGATGCTTAGAGACATCTGCAGGGGTTTGA
TGTGCATACACCGGATGAAGATAGTCCACCGCGACCTAAAGAGTGCCAACTGTCTAGTGGACAAACATTGGACAGTCAAGA
TCTGTGATTTTGGGCTGTCAAGAATAATGACTGATGAAAACATGAAGGACACTTCATCTGCTGGGACACCAGAGTGGATGGC
TCCAGAGCTCATTCGCAACAGGCCTTTTACAGAGAAATGTGATATCTTTAGTCTTGGGGTCATAATGTGGGAACTTTCAACTT
TACGTAAACCATGGGAAGGAGTTCCACCTGAGAAGGTTGTCTTTGCTGTTGCACATGAAGGGTCACGTTTGGAGATTCCTGA
TGGTCCACTCAGCAAACTAATTGCAGATTGTTGGGCAGAGCCTGAAGAACGCCCAAATTGTGAAGAGATACTTAGAGGCTT
ACTCGATTGTGAGTACACACTGTGCTAA

>AtRAF14 (AT2G42630)

ATGATGCAGAGTGACCTCTTGAAAGAGCGAGGTGTTGATGATTCATCTCCTTACTCTCCAGATGAAAAGAATGTCTCTGGTTT
CCAGCTTGATTCCCATGACCTAGTGTCTGGTGAATGTTCGACAGTATATCCGCGGAAGTCAATATCATTGCCGTCATCTCCGC
GAAGCTATCAAATTCAATTATCTGAAAGAAGTGAACATTCACCTCAAGAAATAAGCCACATTTGGAATGAAGTGCTGGAGTC
TCCAATGTTCCAGAATAAACCTTTGTTACCATTTGAAGAATGGAACATAGATTTCTCCAAGTTGAAAGTTGGGGCTTCTGTTG
GGAGTGGAACTTCTGGAGTAGTATGTCGTGGCGTCTGGAATAAAACTGAAGTTGCGATCAAGATATTTCTTGGACAACAGCT
TACAGCTGAGAACATGAAAGTTTTCTGCAATGAGATATCTATTCTTAGCCGTCTTCAACATCCCAATGTTATTCTGCTCCTTGG
AGCATGCACAAAGCCTCCTCAGTTATCACTGGTCACAGAGTATATGAGTACGGGGTCCTTGTATGATGTGATCCGTACCAGAA
AAAAGGAGTTGAGCTGGCAAAGGAAACTAAAGATTCTGGCTGAAATTTGCAGGGGTTTGATGTACATACACAAGATGGGGA
TAGTCCACCGTGATCTAACCAGTGCGAACTGCCTGTTAAACAAGTCGATAGTCAAGATCTGTGATTTTGGGCTCTCGAGAAG
GATGACAGGTACGGCAGTGAAAGATACTGAAGCTGCAGGAACTCCTGAGTGGATGGCTCCTGAACTTATCCGCAATGAACC

CGTCACAGAAAAAAGCGACATCTTTAGCTTTGGTGTTATAATGTGGGAACTTAGCACTCTATCCAAGCCCTGGAAAGGAGTC
CCAAAAGAAAAAGTCATTCATATCGTTGCAAACGAAGGAGCCCGGCTCAAAATACCTGAAGGACCACTACAGAAGCTTATT
GCAGATTGTTGGTCGGAACCAGAGCAAAGGCCGAGCTGTAAAGAGATACTGCACCGTCTTAAGACCTGCGAGATTCCGATT
TGCTGA

>AtRAF15 (AT3G58640)

ATGGGAGAGACGGGAGATGATGCTGGGCCATCAGAGCAAGGACCTTCTAATCAAACCTGGTGGCCTTCAGAATTTGTGGAG
AAATTTGGTTCTGTTTATCTGGGATCGCAAGAAGAGACATCTAGTACTAAAGACTCACCTAGAAATCTTGGGCAAGATGGGC
TGCCATCTAGTACTGCTTCGAATATTCTTTGGAGTACTGGTTCACTTTCTGAGCCCATCCCAAATGGCTTCTACTCTGTGATCC
CGGATAATAGATTGAAGCAGCTATTTAATAACATTCCTACATTGGAAGATCTTCATGCTCTGGGTGACGAAGGTCTTAAGGCT
GATGTGATTCTAGTTGATTTTCAGAAAGATAAAAAGCTTTTCAGGCAGAAGCAGTTGATTACCAAACTTGTCAGTGGATTGA
ACTCAAAGCCAGCTACTATTATTAAGAAAATTGCTGGACTGGTAGCAGATGTTTATAAGCAATCAACTCTGCAGAGTCCAGC
AAAAAGTACGCAGTCATTTGAAAACTGTGGTATTCAACTGCTAGGACAGATCAAGCATGGTTCTTGTCGTCCTCGAGCAATC
TTGTTCAAAGTCTTAGCTGATACTGTTGGCCTGCAAAGTCGACTTGTAGTGGGTTTGCCAAGTGATGGAGCTGCTGAGAGTG
TGGACTCATATAGTCATATTTCTGTCACTGTTTTGCTGAATTCTGTGGAAATGCTGGTTGACCTAATGCGATTTCCTGGTCAGC
TTATTCCTCTATCAACCAAAGCAATTTTTATGAGTCATATCTCAGCGGCAGGGGAAAGTGATTCTGCAGAGAATGACTCTTGT
GATTCACCTTTGGAGCCAAACAGTCCCATGTTTGGTTATCCAGAGAAATTTGACCATGAAAATGCAGAAAAAGATGAAAACC
TTTCGTTGCACAGGAAACTGGATGGGTCTCCAAATACGTCTGGTCCACCATCAAGGAATATGCTGTTACGATCTGCCTCTGCG
CTAGAGAGGAAATTAAGTTTCTCACAGAGCGAGTCAAACATGGCTAACGAGTTTTGGCGGCAGAGCCGGAGGAAGGTCATT
GCTGATCAGCGGACTGCTAGTTCCAGTCCCGAACATCTTTCCTTCCGAGCCCGTACAAAGTCCATGCTAAGTGGCGACAAGA
ACTTGGCACGAGATTTCACCGGGGATGTTGCCACATCAAGCTGCAAATCTGTTGGAGGAGCAAAAATGGAAACTAAGAGAA
TAAGGAGGAGAAGCATCAGTATAACTCCTGAGATTGGCGATGATATTGTCAGGGCAGTACGAGCGATGAATGAAGCCTTGAA
GCAGAATCGCCTCTCAAAAGAGCAAGGGGATGATGATTCATCTCCTAACTCTCCAAATGACAGAACCGAGAGCTCTCATCTC
CAGAAAAATGTCTCTGGTTTTCATCTTGATGCCCATGACCAAGTTTCTGGTGGAAGGTCGACACTTTCAAGGGAACCACTAG
ATCCGCAGAAGGCAATCTCACTACCTTCTTCTCCCCAGAACTACCGAAGCCAATATGAACAGAGTGGATCGTCACATCGCAA
TATAAGCCACATCTGGGATAAAGTATTGGGATCTCCCATGTTCCAGAATAAGCCATTATTACCTTATGAAGAATGGAACATAGA
CTTCTCTGAGCTGACTGTTGGGACACGTGTCGGAATTGGATTTTTCGGAGAAGTATTCCGTGGGATCTGGAACGGGACAGAC
GTTGCTATCAAAGTGTTCCTTGAGCAAGACCTTACAGCTGAGAACATGGAGGATTTCTGCAATGAGATATCAATTCTTAGCCG
ACTTAGACATCCCAATGTTATACTGTTCCTTGGAGCATGCACAAAGCCTCCACGATTATCACTGATCACTGAGTACATGGAAA
TGGGATCTTTGTATTACTTGCTCCACTTGAGTGGACAAAAAAAGAGGTTAAGCTGGCGGAGGAAGCTAAAGATGCTGCGGG
ACATTTGCAGGGGATTGATGTGTATACACCGGATGGGGATAGTACACCGTGATATAAAGAGTGCAAACTGTCTTTTGAGCAA
CAAATGGACAGTCAAGATCTGTGATTTTGGGCTGTCGAGAATAATGACAGGGACAACAATGAGAGACACAGTCTCTGCAGG
AACTCCAGAGTGGATGGCTCCTGAACTTATCCGCAATGAGCCCTTCTCAGAAAAGTGTGATATCTTCAGTTTAGGTGTAATAA
TGTGGGAGCTATGCACTTTAACCAGACCTTGGGAAGGAGTACCGCCTGAACGGGTTGTTTATGCTATTGCTTACGAGGGAGC
TCGGCTTGAGATTCCTGAAGGACCACTGGGAAAGCTTATTGCAGATTGTTGGACAGAACCGGAACAAAGACCGAGTTGCAA
TGAGATACTCTCTCGTTTGCTGGATTGTGAGTATTCGCTTTGCTAA

>AtRAF16 (AT1G04700)

ATGAGAATGGAGTTTCCTGGCAGCTCCAATCAGCATTTAGGTAGGGATAGGTTCAATGGAGAAGTTGGTTGCGGTAATAACT
GTTCTCAGACAGGAGAGGAATTTTCAAATGAGTTTTTGAGGGATTTTGGTGCTCAACGAAGACTACAACATGGTGGTGTTAA
CAGAAATGTTGAGGGTAATTATAATAATCGCCATCTTGTTTACGAGGATTTCAACAGAATTCTTGGCCTTCAAAGAGTTGATT
CCAACATGTCAGAAGGTATTAATTCCTCGAATGGTTACTTTGCTGAGTCAAATGTTGCAGACTCACCTCGGAAGATGTTTCAA
ACTGCAATATCAGATGTTTATTTGCCTGAAGTATTGAAGCTTCTTTGTAGCTTCGGGGGAAGGATCTTACAGCGGCCTGGTGA
TGGCAAGCTTAGATACATTGGAGGGGAGACTCGGATTATCTCAATACGCAAACATGTTGGATTAAACGAATTGATGCACAAG
ACTTATGCTTTGTGCAACCATCCGCATACAATCAAGTATCAATTACCAGGAGAAGATCTTGATGCCCTTATATCGGTTTGCTCA
GATGAGGATCTTCTTCATATGATTGAAGAGTATCAAGAAGCTGAAACAAAGGCTGGATCCCAGAGGATTAGGGTCTTTCTTG

TACCTTCCACAGAATCATCAGAGAGTCCTAAAATTTTCCATGAAAGGAATATGAACATAAACCGAAACACGAACCAGCAGAC
GGATATAGATCATTATCAGTATGTTTCAGCTCTTAATGGTATTGTAGATGTGAGTCCTCAAAAGAGTTCCAGCGGCCAAAGTG
GAACAAGTCAGACCACACAATTTGGGAATGCTTCAGAATTTAGCCCTACATTTCATCTTCGAGACTCGCCTACTTCTGTTCAT
ACATGGGAACACAAAGACAGCAACAGTCCAACCTTCATGAAACCATATGGGAACACGAATGCTGTTCATTTTATGCCGAAAA
TGCAGATTCCCAGGAACTCGTTTGGTCAACAATCTCCTCCAACATCTCCTTTTTCAGTCCACAAGAGAGCAAACACCGACGT
TCCTTATTTTGCCGATCAAAATGGTTTCTTTGACCCTTACTTGGCTGCTCCAAACTTCCCACAGCAAAATAGGTTCTTTTTTGA
GACTACTACACAAAAGCAGAAGCATCCAGAAGTGAATCTTCATGACCGGAGGCCAAGCGATGATATTTACCCCCATGGCCAA
GCTTATATCGGAGCCGAGAAAATGACGCTTAAGAAAAACGCTCTTTCTGATCCGCAGCTGCATGATGAGAGCCAAATCAACA
ATGGGTTAGAAGCATTTACCAAACAACCATGGAAAATATTGAGAAAGAATCTACGTGTTGTGGCTACATCAAAGTGGGAAGA
TAGTGATGATATCTATTTCAATAATCCAGAAGGCAAGAGATGCAAGGAGCTTGAACTCACCAAGGAAGTTCCTAATAGTTGG
ATCAATCGCGATAATAATCCCGATTCTTTTGATCAGGCAACAAAGAAACAGGATGGTAGTAACAGTAATAGCTCCTTCAGTCC
GAATTATTTCAGTCCGAATCATCAACCAGCTGCTCAGATTACTTCAAGTGATTCACAAGATTCAGGAAGCTCAGTGTTCTCTT
TATCAGTTAACACCAACGAAAATTATCTTGATTGTTCGAGGGAAAAATTTAATGGTTTCCAGCATGACATGTCACTAGATATTC
TCATCAGAAGCCACACTTCAGCTACAGATCAACTATGTAGTACAACCAAGTCAAGTGATAAAGCAGATTACTCCTCACCCAA
TACAAATTTCCCCGTGGTATTTCTGAGACAGGAACCTATGATTCCGAGACATGATTTGGAGACAAATAGCGATGATTCAGATA
CTCAAAAATCTCTTCCTAGAGAAGAGAGTATTCACTATAGTGGATTGCCACTTAGGAAGGTGGGAAGTAGAGAAACTACTTT
TATGCACACGCAGGGGTCAGATGATTTCTTCAAAAGCAAGCTGCTTGGTCCACAGTTGATAGTTGAGGATGTGACGAACGA
AGTAATTTCAGATAATCTCTTATCAGCTACTATTGTCCCTCAAGTAAATAGGGAGTCCGATGATGATCACAAATCCTACACAAG
GGAGAAAGAGATTACAAATGCGGATCATGAATCAGAAATGGAGGAAAAGTACAAGAAAAGCAGAAACACCGATGATTCATT
CAGCGAAGCTGCAATGGTTGAAATCGAAGCTGGAATATATGGCTTACAGATAATTAAAAACACTGATCTTGAAGACTTACAC
GAGCTTGGATCTGGTACATTTGGAACTGTTTACTATGGGAAGTGGAGAGGAACTGATGTTGCTATCAAGAGGATAAAGAATA
GTTGTTTCTCTGGTGGATCATCCGAGCAAGCACGGCAGACTAAAGATTTCTGGAGAGAAGCTCGGATATTGGCGAATCTTCA
CCATCCTAACGTAGTGGCCTTTTATGGAGTTGTACCAGATGGACCTGGTGGAACAATGGCAACAGTGACTGAGTATATGGTG
AACGGATCTTTGAGGCATGTCTTGCAGAGAAAAGACAGATTACTTGATAGGCGCAAAAAACTGATGATAACTCTTGATTCTG
CATTTGGCATGGAGTATCTACATATGAAAAATATTGTTCATTTTGATCTTAAATGCGATAACTTGCTTGTAAACTTGAGGGATC
CACAACGACCCATTTGCAAGGTTGGAGATTTCGGATTGTCGAGAATAAAACGAAACACGCTTGTCTCTGGTGGAGTAAGAG
GAACCCTTCCATGGATGGCACCAGAGCTGCTAAACGGAAGCAGCAATCGGGTCTCAGAGAAAGTCGATGTTTTCTCATTTG
GTATCGTGATGTGGGAGATCTTGACAGGCGAAGAACCCTATGCGAATCTGCATTGCGGTGCCATCATTGGTGGAATTGTGAA
CAATACGTTAAGACCTCCGGTGCCTGAACGGTGTGAAGCTGAGTGGAGGAAGTTGATGGAGCAATGCTGGTCATTCGATCC
AGGAGTACGGCCATCGTTTACGGAGATTGTTGAACGACTTCGATCAATGACTGTTGCCTTGCAACCCAAGCGAAGAACCTAA
>AtRAF17 (AT1G14000)
ATGAGCTCCGATTCACCGGCGGCTGGAGACGGTGGCGAACAAGCCGCTGCGGGAACAAGCGTGCCTTCACCTTCATACGAC
AAACAAAAGGAGAAGGCACGTGTGAGTAGAACATCTCTGATACTATGGCACGCGCACCAAAACGACGCAGCTGCAGTTCG
GAAACTCTTGGAGGAAGATCCGACTTTGGTTCACGCTAGAGACTATGACAAACGAACGCCTCTCCACGTGGCGTCGCTTCA
TGGATGGATCGATGTTGTTAAATGTCTGCTTGAGTTTGGTGCTGATGTCAATGCTCAGGATAGGTGGAAGAACACGCCACTG
GCTGATGCAGAAGGAGCTAGGAAGCAGAAAATGATTGAACTGCTGAAATCTCATGGTGGTTTGTCTTATGGTCAAAACGGA
AGTCACTTTGAACCAAAGCCTGTGCCACCACCAATTCCAAAGAAATGTGACTGGGAAATTGAACCTGCTGAGCTGGATTTT
TCAAATGCTGCAATGATTGGAAAGGGTTCATTTGGTGAGATTGTGAAAGCCTATTGGAGAGGAACACCGGTAGCTGTGAAA
CGTATTCTTCCTTCTCTTTCAGATGATAGACTGGTTATTCAGGACTTCAGGCATGAGGTTGATTTGCTAGTTAAGCTTCGCCAT
CCAAATATAGTCCAATTCTTAGGAGCGGTAACCGAAAGAAAGCCCCTTATGTTAATCACTGAGTACTTACGCGGGGGAGATC
TTCATCAATACCTCAAGGAGAAGGGCGGTCTTACTCCAACAACTGCTGTCAACTTTGCTTTGGATATCGCGAGAGGAATGAC
ATATCTTCACAATGAACCTAATGTTATCATTCACCGAGATCTTAAACCAAGGAACGTTCTTCTGGTTAATTCTAGTGCAGACCA
CTTGAAAGTTGGAGACTTTGGACTTAGTAAGCTCATAAAAGTTCAAAACTCTCATGATGTCTACAAAATGACTGGAGAAACA
GGAAGCTATCGTTATATGGCTCCTGAAGTATTCAAGCATAGAAGATATGACAAGAAAGTCGATGTCTTCTCCTTTGCAATGAT

ACTTTACGAGATGCTTGAAGGAGAGCCACCATTTGCGAACCATGAGCCATATGAAGCAGCCAAACATGTATCTGATGGACAT

AGACCTACATTCCGTTCCAAAGGATGCACACCAGATCTAAGAGAGTTAATTGTGAAATGCTGGGACGCAGACATGAACCAG

AGACCGTCGTTTCTGGACATTCTCAAGAGACTTGAAAAGATCAAGGAAACTCTACCATCAGATCACCATTGGGGCTTATTCA

CTTCATAA

>AtRAF18 (AT1G16270)

ATGGATAGAAACAGACCACCACATCCGTTCCAGCAACATGCTATGGAACCTGGATATGTGAATGACTCTGTCCCACAAGGAT

TTACGCCTGATCAAACGGGTCTCTCGAATGCAAATGTCCGACCTAATCCTGCAGATGTTAAGCCGGGGCTTCATTACTCCATA

CAAACAGGAGAGGAATTTTCTCTTGAGTTTCTGCGTGATCGTGTCATTTCTCAAAGGTCTGCAAATCCCATTGCAGCTGGAG

ATATCAATTATCCCACAGGTTATAACGGGCACGCAGGGTCTGAATTTGGTTCAGATGTTTCCAGGATGAGCATGGTGGGAAAT

GGCATCAGGCAGTATGAGAGAACAAACCCCCCGGTTCACGAGTTTGGAAATAAACTCGGGCATATCCATTCAGCACCAGAA

GCTTCATTATGTCAAGATAGAAGTTTAGGAAATTTCCATGGATATGCATCTTCTTCAGCCTCAGGTAGTTTAACAGCAAAGGTT

AAAGTCCTTTGTAGCTTTGGTGGGAAAATACTTCCACGTCCAGGTGATTCAAAGCTTAGATATGTTGGAGGTGAAACACACA

TTATTTCCATAAGAAAGGACATTTCTTGGCAGGAGCTCAGGCAAAAAGTTCTTGAAATCTACTATCGGACTCATGTTGTCAAG

TATCAACTTCCTGGTGAAGATCTTGATGCCTTGGTGTCTGTATCATGTGACGAAGATCTACTGAATATGATGGAGGAGTATAAT

GAGATGGAAAACCGTGGGGGTTCGCAAAAGCTTAGAATGTTTCTGTTCTCTGTGAGTGATTTGGATGGTGCTCTTTTGGGGG

TTAACAAAAGTGATGTTGACTCTGAGTTCCAGTATGTTGTAGCTGTAAATGACATGGACCTTGGATCAAGAAGCAACTCAAC

CCTTAATGGACTGGACAGCTCTTCTGCAAACAATTTAGCTGAGCTGGATGTCAGGAACACCGAGGGAATCAATGGTGTTGGC

CCTTCGCAGTTAACAGGGATAGATTTTCAACAATCCTCTATGCAGTATTCTGAGTCTGCTCCACCAACCTCCTTTGCTCAGTAT

CCTCAATCTATCCCACACAATGGTGCATTTCAGTTTCAGCAAGCTGTTCCCCCAAATGCTACTCTTCAGTATGCCCCGTCCAA

TCCACCAAGTTCGTCTGTCCACTATCCTCAATCTATTCTACCAAATTCCACTCTCCAGTATCCACAATCGATCTCATCCAGCTC

GTATGGATTATACCCACAATATTATGGAGAAACCGAGCAATTTCCAATGCAGTATCATGATCACAATTCTTCTAATTACTCTATT

CCCATACCTTTCCCAGGGCAGCCATACCCTCACCCTGGCATCACACAGCAAAACGCACCTGTTCAGGTAGAGGAGCCAAAC

ATAAAACCTGAGACGAAAGTTCGTGATTATGTGGAGCCTGAAAATCGTCATATTCTGGCAACTAATCACCAGAATCCCCCTC

AAGCTGATGATACTGAGGTTAAGAATCGGGAGCCATCAGTTGCAACAACTGTACCCAGCCAGGATGCTGCACATATGTTGCC

CCCAAGAAGAGACACACGGCAGAACACTCCTGTGAAGCCTTCTACTTACCGTGATGCTGTTATAACTGAGCAGGTCCCAGTA

TCCGGTGAAGATGATCAGCTTTCAACATCAAGTGGTACCTGTGGTCTTGTTCATACCGACTCTGAGTCGAATCTAATTGATCT

TGATTATCCAGAACCTTTACAGCCCACCCGGAGAGTATATCGTTCGGAGAGGATACCTCGTGAACAGTTAGAAATGCTAAATC

GCTTGTCCAAGTCTGATGACTCACTTGGTTCTCAGTTCTTAATGTCTCATCCACAAGCTAGCACTGGACAGCAAGAACCAGC

AAAAGAAGCAGCAGGTATATCACATGAAGATTCACATATTGTAAATGATGTGGAAAACATCTCTGGAAATGTAGTGGCATCA

AATGAAACCTTGGACAAAAGAACGGTCTCTGGTGGAGGTATTGAGACGGAAGCTCGTAACTTGAGCCATGTAGACACAGAA

AGGAGTCATGATATCCCTGAAAAGCAAACTTCTTCAGGTGTTCTTATTGATATCAATGACAGGTTCCCTCAGGACTTCCTTTC

TGAAATATTCGCAAAGGCACTCTCTGATGATATGCCGTCAGGTGCCAATCCATATCAGCATGATGGAGCTGGTGTTAGCTTGA

ATGTAGAGAATCATGATCCTAAAAATTGGTCTTATTTTCGGAATCTGGCCGATGAACAGTTTAGCGATAGGGATGTTGCTTATA

TTGACCGAACCCCTGGCTTTCCATCTGACATGGAAGATGGTGGAGAAATTGCCAGATTGCATCAGGTTGCTCCGTTGACAGA

AAACCGTGTGGATCCTCAGATGAAGGTCACAGAGAGTGAGGAATTTGATGCTATGGTTGAGAATTTAAGGACCTCAGACTG

TGAACAAGAGGATGAAAAGTCAGAAACAAGGAACGCTGGACTTCCCCCAGTTGGCCCGTCTCTGGCAGATTACGACACGA

GTGGCTTGCAGATCATTATGAATGACGATCTCGAGGAGCTAAAGGAGCTTGGTTCTGGCACATTCGGTACAGTGTATCATGGT

AAATGGAGAGGATCAGATGTTGCCATCAAAAGGATAAAGAAGAGTTGCTTTGCTGGCCGATCATCTGAGCAAGAAAGATTA

ACTGGTGAATTCTGGGGAGAAGCTGAAATTCTTTCAAAGCTTCATCATCCAAATGTGGTTGCATTTTATGGTGTTGTAAAAGA

TGGACCTGGTGCAACATTGGCTACTGTAACAGAGTACATGGTTGATGGTTCTCTGAGACATGTTCTAGTCAGGAAAGATAGA

CACCTGGATCGTCGTAAGAGACTAATCATTGCCATGGATGCTGCCTTTGGAATGGAATACTTGCACGCCAAAAACATTGTTCA

CTTCGATTTGAAATGTGACAATTTACTTGTGAACCTCAAAGATCCTTCTCGCCCAATCTGCAAGGTTGGTGATTTCGGTTTGT

CGAAAATCAAAAGAAATACATTGGTATCTGGTGGTGTACGCGGAACCTTACCATGGATGGCACCAGAGCTTCTCAATGGTAG

CAGCAGCAAAGTTTCAGAGAAGGTCGATGTCTTCTCTTTTGGTATAGTCTTGTGGGAGATTCTAACCGGCGAGGAACCATAT

GCTAATATGCACTACGGTGCTATAATAGGTGGGATAGTGAACAACACACTGAGGCCGACCATACCGAGCTACTGTGACTCGG
ACTGGCGAATATTAATGGAGGAGTGTTGGGCGCCTAACCCAACAGCAAGGCCATCCTTCACGGAGATAGCTGGTCGGTTACG
TGTTATGTCAACTGCAGCTACTTCGAACCAATCCAAACCACCAGCTCACAAGGCTTCAAAGTGA

>AtRAF19 (AT1G62400)

ATGGAGAAGAAGAGATTTGACAGTATGGAATCTTGGTCGATGATCCTCGAGTCCGAGAACGTGGAGACATGGGAAGCTTCT
AAAGGCGAAAGAGAGGAATGGACCGCAGATCTTTCGCAGCTATTTATCGGAAACAAATTTGCTTCCGGAGCACACTCTAGA
ATATACAGAGGGATCTACAAACAAAGAGCCGTTGCCGTGAAGATGGTGAGGATCCCAACACACAAGGAAGAGACAAGGGC
TAAGCTTGAACAACAGTTTAAGTCCGAGGTTGCTCTGCTTTCTCGTCTCTTTCACCCTAACATCGTTCAGTTCATTGCGGCGT
GCAAGAAACCGCCGGTGTACTGCATTATAACAGAGTACATGTCACAAGGAAACCTCCGAATGTACCTAAACAAGAAAGAGC
CTTACTCGCTTTCGATCGAGACCGTACTAAGGCTGGCTCTAGACATCTCAAGAGGAATGGAGTATCTTCACTCGCAAGGTGTT
ATCCACAGAGACCTAAAGTCCAACAATTTGCTTTTGAACGACGAGATGAGAGTTAAAGTTGCAGACTTTGGGACATCTTGTC
TTGAGACGCAATGCAGAGAGGCCAAGGGTAACATGGGAACTTATAGATGGATGGCTCCAGAGATGATCAAGGAGAAGCCTT
ATACCAGAAAAGTCGATGTTTATAGCTTTGGCATCGTTCTATGGGAACTCACCACCGCGTTGCTTCCGTTCCAAGGCATGACT
CCTGTGCAAGCCGCATTCGCAGTCGCTGAAAAGAACGAAAGACCACCATTGCCGGCGAGCTGTCAACCAGCGCTAGCTCAT
CTAATCAAGAGGTGTTGGTCAGAGAATCCGTCGAAGCGGCCAGACTTCTCAAACATAGTGGCGGTGCTAGAGAAGTACGAC
GAGTGTGTCAAAGAAGGACTGCCTTTGACGTCACACGCAAGCCTAACGAAGACAAAGAAAGCTATTCTTGATCACCTTAAA
GGCTGTGTCACATCCATTAGCTCACCATTTTCTTCCTCCTCTGTTCCTGTAAATGCCTAG

>AtRAF20 (AT1G79570)

ATGGATAAAGCTAGACATCAACAGCTTTTTCAGCATTCCATGGAACCTGGTTATAGAAACGAGACTGTTCCTCAACCATTTAT
GCCTGATCAAACAGGGAGCGCGAGTGCTAATATGCGACCTCCCAATTCTAATGGTTCAGATGTTAAGGCGGTGCATAACTTC
TCCATACAAACAGGTGAGGAATTCTCTCTTGAGTTTATGCGTGATCGGGTGATTCCTCAGAGGTCTTCCAATCCCAATGGAGC
TGGCGACATGAACTATAACACTGGTTATATGGAGCTTCGAGGCTTGATAGGGATAAGTCACACAGGATCTGAGTGTGCTTCA
GATGTCTCAAGGTTCAGCACTGTGGAAAATGGCACCAGTGATATTGAGAGGACTAACTCTTCGCTTCACGAGTTTGGAAATA
AACTAAATCATGTCCAGTCAGCACCACAAGCTTTACTAAGTAAAGATAGCAGTGTAGGAAATTTACATGGATACAAGAACAC
GTCTTCTTCAGCCTCAGGTAGTGTAACAGCAAAGGTGAAGATCCTTTGCAGTTTCGGTGGGAAAATACTTCCACGTCCAGGT
GATTCAAAGCTTCGATACGTTGGAGGTGAAACACACATTATATCCATAAGAAAGGATATATCTTGGCAGGAGCTTAGGCAAA
AAATCCTTGAAATCTATTACCAAACACGTGTTGTAAAGTATCAGCTTCCTGGTGAAGATCTTGACGCGTTGGTGTCTGTATCA
TCTGAAGAAGATCTCCAGAATATGCTGGAAGAATATAATGAGATGGAAAACCGTGGGGGATCTCAGAAGCTTAGGATGTTTC
TTTTCTCCATCAGTGATATGGATGATGCTCTTCTGGGGGTTAACAAAAATGACGGCGACTCTGAGTTTCAATATGTTGTAGCT
GTAAATGGCATGGACATTGGATCAGGAAAGAACTCGACCCTTCTTGGGCTTGACAGCTCTTCAGCAAACAACTTAGCTGAG
CTTGATGTGCGGAACACTGAGGGGATCAATACCATTGCTGGAGATGTTGTTGGAGTCGGCGCATCACAGTTAATGGTGAATG
GTTTCCAACAAACGTCTGCTCAACAGTCTGAGTCTATTCCACCAAGCTCATCGCTTCATTATTCTCAATCTATTCCACTGAATG
CTGCATATCAGTTGCAGCAATCTGTTCCGCCAAGCTCTGCTCTTCATTATCCCCAGTCCATTACCCCAGGTTCCTCTCTTCAGT
ATCCCCAATCTATTACGCCAGGCTCCTCTTATCAGTATCCACAATCCATCATACCTGGTTCTGCCAGCTCCTATGGAATATACCC
ACAATATTATGGGCATGTGGTTCAACATGGAGAAAGAGAACGGTTTCCTCTCTATCCTGATCACAGTTCTAACTACTCCGCTA
TCGGGGAAACTACCAGTTCCATACCGATCCAAGGGCATGTCAGTCAGCAAGGTGGCTGGGCTGAAGGGTATCCGTACCCTG
GCAGCACACCCAAAAGCACACAAGCGCTAGCAGAGGAGCAAAAAGTATCATCTGATATGAAAATTCGTGAGGAAGTTGAG
CCTGAAAACCGTAAAACTCCGGGGAATGATCACCAGAATCCTCCCCAAATAGATGATGTTGAGGTGAGGAATCACAATCAG
GTCCGGGAGATGGCAGTTGCAACAACTCCACCTAGCCAGGATGCACATTTACTACCCCCTAGTAGAGATCCACGGCAGAAC
ACTACTGCAAAACCTGCTACCTACCGTGATGCTGTTATTACTGGGCAGGTTCCTCTATCTGGTATTGAAGATCAGCTTTCAAC
TTCAAGCAGTACCTATGCCCCTGTTCATAGCGACTCTGAGTCAAATTTAATTGATCTTAACTACCCGGAGCCTGAGCAGTCTT
CCCAAAGGGTATATTGCTCAGAGAGAATACCTCGTGAACAGCTAGAATTGCTAAATCGTTTGTCGAAGTCCGATAACTCATTA
AGCTCTCAGTTCGTAACGTCTGAGTCACCAGCAAACACTGCACAGCAAGATTCAGGAAAAGAAGCGGTTGGAAAGTCACA
TGACGAATTCAAGACTGTTAATGATGATGCCAATCACCATACGCACAAAGATGTGGAAACAATCTTTGAAAAAGTAGGAGTA

TCAGATGAAACTTTGGAATCCGAGCCCCTGCACAAGATTGTAAATCCTGACGATGCAAACAAGAACAGAGTAGTCAATGGG
GCAGATACTGAAATTGGTGTTTCCAACTTGAGCCATGTAAACGCAGCCATGAGTCATGTTATTCCTGAAGAGCAAGCTTCGC
TGCAGGGAGATATTCTTATTGATATCAATGACCGGTTTCCTCGTGACTTCCTCTCTGAAATATTCTCACAGGCAATCTCAGAGG
ATACATCCACTGTCCGTCCATATCCTCACGATGGAGCTGCTGTTAGCATGAATGTGCAAAATCACGATCGTAAAAACTGGTCA
TATTTTCAGCAGCTGGCTGAAGATCAGTTTATCCAAAGAGATGTTGTACTTGACCAAGCTGATTCTCGCATTCCATCTGACCG
TAAAGATGGTGGAGAGAGCTCCAGATTGCCTTATGTCTCGCCATTGAGTAGAGATGGTATTTCAACAAACCTTGCTAATCCTC
AGTTGACTTTAGGTCAAGATTATGGGGGTAATTTTTCTGAAAAGGATGGGGGTGGCACTGGCAGCATTCCCCCTGCTCTAGA
GAATGAACAGATGAAGGTCACTGAGAGTGAGGAATTTGGTGCCATGGTGGAGAATCTTAGGACGCCGGATTCTGAACCAAA
GGATGAAAAGACAGAAACAAGGCATGCTGCGCTTCCTCCACTTGGCTCAGAGTTTGACTACAGTGGCTTGCAGATCATTAA
GAATGAAGATCTTGAGGAGTTGAGGGAGCTTGGTTCTGGTACTTTTGGAACGGTGTATCATGGGAAGTGGAGAGGATCAGA
TGTCGCTATCAAGAGGATAAAGAAGAGTTGCTTTGCTGGGCGGTCATCAGAGCAAGAGAGATTGACTGGTGAATTTTGGGG
GGAAGCTGAAATTCTTTCAAAGCTTCATCATCCGAACGTGGTAGCATTTTATGGTGTTGTAAAAGACGGGCCTGGAGGGACC
TTGGCGACTGTGACAGAGTACATGGTTGATGGTTCTCTGAGGCATGTTCTGGTCCGGAAGGACAGGCATCTTGATCGTCGTA
AGAGACTAATCATTGCCATGGATGCTGCATTTGGAATGGAATATTTACACTCCAAAAACACTGTTCACTTTGATTTGAAATGT
GACAATTTACTTGTGAACTTGAAAGATCCTTCTCGCCCAATCTGCAAGGTTGGTGACTTTGGTTTGTCAAAAATCAAGAGAA
ATACGTTGGTATCTGGTGGTGTACGTGGAACCTTACCGTGGATGGCACCAGAGCTTCTAAACGGGAGCAGTAGCAAAGTTTC
TGAAAAGGTTGATGTTTTCTCTTTCGGTATCGTTCTGTGGGAGATTCTAACCGGAGAGGAACCATATGCCAATATGCACTATG
GGGCAATAATAGGTGGGATAGTGAACAACACACTGAGACCAACCATACCAGGCTTCTGTGACGATGAATGGAGAACACTAA
TGGAGGAATGTTGGGCACCAAACCCAATGGCAAGACCATCTTTCACAGAGATAGCTGGTCGCTTACGAGTAATGTCATCAGC
AGCAACTTCAACTCAATCCAAGCCATCAGCTCACAGGGCTTCCAAATGA

>AtRAF21 (AT2G17700)

ATGACGATCAAAGATGAGTCGGAGAGTTGCGGTAGCAGAGCCGTCGTTGCTTCGCCGTCACAAGAAAACCCTAGACATTAC
CGGATGAAACTTGATGTCTATAGTGAGGTTTTACAGCGACTTCAAGAATCTAATTACGAAGAAGCCACTCTTCCTGATTTCGA
GGATCAACTTTGGCTCCATTTCAATCGTCTTCCTGCTCGATATGCTCTTGATGTTAAAGTCGAGAGAGCGGAAGATGTTCTCA
CACATCAGAGATTGCTAAAATTGGCTGCAGATCCTGCTACTAGGCCTGTCTTTGAAGTTCGAAGTGTACAGGTTTCTCCCAG
AATCTCTGCTGACTCTGACCCTGCGGTGGAGGAAGATGCTCAAAGCTCTCACCAACCAAGCGGACCGGGGGTTCTTGCTCC
TCCAACTTTTGGTTCTTCTCCAAATTTTGAGGCTATTACTCAGGGAAGTAAAATTGTTGAAGATGTTGATAGTGTTGTGAATG
CAACATTGTCTACACGACCGATGCACGAGATCACTTTTTCAACCATTGATAAACCGAAACTCCTTAGCCAGCTAACTTCCCTG
CTTGGTGAGCTTGGACTGAATATACAAGAGGCTCATGCTTTTTCCACTGTAGATGGTTTCTCTTTAGATGTCTTTGTAGTTGAC
GGTTGGTCTCAGGAGGAAACTGATGGCCTAAGAGATGCATTGAGCAAAGAAATACTGAAGCTTAAGGATCAACCTGGTTCA
AAACAGAAATCTATTTCTTTCTTTGAGCATGACAAATCAAGCAATGAGCTTATACCCGCCTGCATTGAAATACCCACGGATGG
AACTGATGAGTGGGAAATCGACGTGACTCAGCTCAAAATTGAAAAGAAAGTGGCATCTGGTTCATATGGGGATCTGCATAG
AGGCACTTATTGCAGTCAGGAAGTAGCTATCAAATTTCTCAAGCCTGATCGTGTAAACAATGAGATGCTGAGAGAATTTTCTC
AAGAAGTTTTTATAATGAGGAAAGTTCGACACAAAAACGTCGTTCAATTTTTGGGTGCATGCACAAGATCTCCAACCCTCTG
TATAGTGACTGAGTTTATGGCTCGAGGGAGCATATATGATTTTCTTCACAAACAGAAATGCGCTTTCAAACTTCAAACTTTAC
TCAAAGTTGCACTTGATGTCGCAAAAGGAATGAGCTATTTGCATCAAAACAACATTATTCACAGGGACCTTAAGACTGCGAA
TCTTCTTATGGATGAACATGGACTTGTCAAGGTTGCTGATTTCGGAGTTGCCAGAGTGCAGATTGAATCAGGGGTCATGACT
GCTGAAACTGGGACATACCGGTGGATGGCTCCAGAGGTCATTGAGCACAAACCTTACAATCACAAGGCAGATGTGTTCAGT
TATGCGATAGTGCTATGGGAACTTCTGACTGGTGACATCCCATATGCTTTCTTGACTCCACTACAAGCAGCTGTTGGCGTTGT
CCAAAAGGGGCTTCGACCCAAAATCCCAAAGAAAACACACCCAAAAGTGAAGGGGCTTCTAGAGAGATGCTGGCATCAAG
ACCCAGAACAGAGACCACTGTTTGAGGAAATCATAGAAATGCTACAACAGATAATGAAAGAGGTAAACGTCGTAGTGTGA

>AtRAF22 (AT2G24360)

ATGCTAGAAGGAGCAAAGTTCAACGTGCTTGCTGTTGGGAATCATCACAACAACGACAACAATTACTATGCTTTTACGCAAG
AGTTTTATCAAAAACTTAATGAAGGTTCAAACATGTCCATGGAGAGTATGCAGACGAGTAACGCTGGAGGATCTGTCTCAAT

GTCTGTGGATAACAGTAGCGTTGGTTCCAGCGATGCTCTTATTGGCCACCCGGGTTTGAAGCCTGTACGCCATTACTCACTCT
CGGTTGGTCAAAGCGTGTTTCGCCCGGGAAGAGTTACCCATGCGTTGAATGATGATGCTTTGGCTCAAGCACTGATGGATAC
CAGGTATCCAACTGAAGGGCTGACGAACTATGATGAGTGGACGATTGATCTGAGGAAACTCAACATGGGTCCTGCCTTTGCT
CAAGGGGCTTTTGGTAAATTATACAAAGGGACATACAACGGTGAAGATGTAGCTATCAAAATACTTGAGCGGCCAGAGAAC
AGCCCAGAAAAGGCACAGTTCATGGAACAACAGTTTCAGCAAGAGGTGTCTATGCTTGCTAATTTGAAGCACCCAAACATT
GTGAGGTTCATTGGTGCATGTCGCAAGCCAATGGTGTGGTGTATAGTGACTGAATACGCCAAAGGAGGTTCAGTGAGGCAG
TTTTTGACTAGGAGACAGAACCGAGCCGTCCCTTTGAAGTTAGCTGTTAAACAGGCTTTGGATGTTGCTAGGGGTATGGCTT
ATGTCCATGGACGCAACTTCATACACAGAGATCTCAAGTCAGATAACCTTCTCATCTCAGCAGATAAGTCCATCAAGATTGCA
GATTTTGGTGTTGCAAGAATTGAAGTTCAAACCGAAGGAATGACACCAGAAACTGGAACTTACAGATGGATGGCTCCAGAG
ATGATACAGCATAGAGCCTACAATCAAAAAGTGGATGTGTATAGTTTCGGGATTGTGCTGTGGGAGTTAATCACAGGACTCTT
ACCGTTCCAGAACATGACAGCTGTACAGGCAGCGTTTGCGGTTGTAAACAGAGGAGTGCGTCCAACAGTCCCAAACGATTG
TCTCCCGGTGCTGAGTGACATTATGACTCGATGTTGGGATGCTAATCCAGAAGTCCGTCCATGTTTTGTGGAGGTTGTGAAGC
TGCTTGAAGCTGCAGAAACAGAGATAATGACGACAGCGAGAAAAGCCCGTTTCAGATGTTGCTTGAGCCAGCCGATGACGA
TTGACTAA

>AtRAF23 (AT2G31800)

ATGGCGAATGTAGTGGGACAGCTAAAACGAGGAATCTCGAGACAATTCTCAACTGGATCGCTTCGTCGTACGCTTAGTCGTC
AATTCACGCGTCAAGCTTCACATGATCCTCGCCGGAACAATATGCGATTTAGTTTTGGTCGACAATCGTCTTTAGATCCGATTC
GTCGGAGTCCTGATGGTTCTAACGGTCCGCAGCTTGCTGTACCGGATAATCTCGACGCTACTATGCAGCTTCTATTTGTGGCT
TGTAGAGGTGATGTTGAAGGTGTTCAGGATCTGCTTGATGAAGGTATTGATGTTAATAGTATCGATCTTGATGGTCGTACGGC
TCTTCATATCGCTGCTTGTGAAGGTCATGTCGATGTTGTCAAGCTTCTTCTTACTAGGAAGGCTAATATTGATGCTCGTGATCG
TTGGGGAAGCACGGCAGCTGCTGATGCTAAGTATTATGGTAACATGGATGTTTTCAACATTTTGAAAGCCCGTGGAGCTAAA
GTTCCGAAAACCAAAAGGACACCCATGGTTGTGGCGAATCCTCGTGAAGTTCCTGAGTACGAGTTAAATCCGCAGGAACTT
CAAGTTCGGAAAGCTGATGGTATCTCAAAGGGAATATATCAAGTGGCTAAATGGAATGGGACTAAGGTTTCTGTAAAGATAC
TTGATAAGGATCTCTACAAGGATTCTGACACTATAAATGCCTTCAAACACGAACTTACTTTATTCGAGAAGGTACGTCATCCT
AATGTTGTGCAATTTGTTGGAGCTGTTACTCAAAATGTCCCCATGATGATTGTGTCCGAGTATCATCCTAAAGGGGACCTTGG
GAGCTATCTTCAAAAAAAAGGTCGTCTTTCTCCAGCCAAAGTTCTAAGATTTGCCCTCGATATAGCCAGGGGAATGAATTATC
TTCACGAGTGTAAACCAGAACCAGTAATCCACTGTGATCTAAAACCCAAAAATATTATGCTCGATAGTGGAGGACATCTGAA
GGTGGCGGGATTTGGTTTGATAAGTTTTGCAAAGTTATCATCTGATAAATCAAAAATCCTTAATCACGGGGCCCATATAGATCC
TTCAAATTACTGTATGGCACCTGAGGTTTACAAAGATGAAATATTTGACAGGAGTGTGGATTCTTACTCTTTTGGTGTCGTATT
ATATGAGATGATTGAAGGAGTACAACCTTTCCATCCTAAACCCCCAGAAGAGGCAGTGAAGCTAATGTGTTTAGAAGGAAG
AAGACCTTCGTTTAAAGCCAAGTCCAAAAGTTGTCCCCAAGAGATGAGAGAATTGATTGAGGAATGCTGGGATACGGAAAC
TTTTGTTAGACCAACATTTTCTGAGATCATAGTTCGATTGGACAAGATCTTTGTACACTGCTCAAAACAGGGATGGTGGAAA
GATACATTCAAGTTCCCTTGGAAATAG

>AtRAF24 (AT2G35050)

ATGGATCAAGCAAAAGGTTATGAACATGTTCGGTATACTGCCCCTGACCCTAGAGATGAGGGACTTGGCTCCATTAATCAAA
GGTTTTCCCACGACTCTTCAACTAATGTTAACACTTATGTACGACCTCCAGATTATGGTGTTTCAACCCCTGCTCGGCCAGTG
CTAAACTACTCAATACAGACCGGTGAAGAATTTGCTTTTGAGTTTATGAGAGATAGGGTTATTATGAAACCGCAGTTCATCCC
AAATGTGTATGGTGAGCACAGTGGTATGCCTGTTTCTGTTAACTTAAGTGCTCTGGGAATGGTTCATCCAATGTCAGAGAGTG
GCCCTAACGCTACAGTGCTTAACATAGAAGAAAAACGTCAGAGCTTTGAGCACGAGAGGAAACCCCCTTCTAGAATTGAAG
ATAAGACCTATCATGAACTGGTCCAGTCAGCCCCAGTTATCTCTTCGAAAAATGATACTGGTCAAAGGCGTCATAGTTTGGTT
TCTTCTAGAGCTTCTGATAGCTCTTTGAACCGTGCGAAGTTCTTGTGTAGTTTTGGTGGTAAAGTTATACCCCGCCCCAGAGA
TCAGAAACTTAGGTATGTAGGTGGTGAAACGCGTATCATACGGATTAGCAAGACTATTTCTTTCCAAGAACTCATGCATAAAA
TGAAAGAAATATTTCCTGAAGCACGCACCATAAAATATCAGCTGCCAGGAGAGGATCTTGATGCCCTAGTCTCTGTATCTTCT
GACGAGGATTTACAAAACATGATGGAAGAATGTATCGTGTTTGGTAATGGAGGATCTGAGAAGCCCAGGATGTTCTTGTTTT

CAAGCAGTGATATAGAGGAGGCTCAGTTTGTTATGGAACATGCAGAGGGTGATTCTGAGGTTCAGTATGTTGTTGCTGTCAA

TGGGATGGATCTAAGTTCACGGAGAAGTTCCCTTGGATTAAGTCCTCCCGGGAACAATTTGGATGAACTACTTCATGGGAAT

TTTGATAGGAAGATCGATCGGGCTGCTACAGAACCAGCAGTGGCTTCGCTTACTCCCTTAGCAGGTAATGAATCTTTACCAGC

GAGCCAAACTTCTCAACCTGTAACAGGATTTTCTACTGGAAATGAGCCATTTTCACAGCCTTATCTAGGACAACAATTGCAG

TTCCCCGGACTTGGTAACCACCAAATTTACACGTCAGGTCACATGGCAAGCATAGGCTATATAGATGAGAAGAGGTCTGCTC

CTTTACATGTTCAACCACAACCTCATTATATCCCGTATTCTGTGAATCCTGAAACACCTCTTGAAAGCCTGGTGCCCCACTATC

CACAAAAACCTGAGCAAGGATTTTTGCGTGAGGAGCAGATCTTTCATGTACAAGATCCAGAAACTTCATCAAAAGAGGCCA

AAATGAGAAGAGATGACTCATTTCAGAAGGTAAATGATCATCCTATATCTACTGTCGAGAGCAATCTTTCAGCAAAGGAGCC

AAAGATGAGGAGAGAATCCTCAACCCCAAGGGTCAATGAGTATCCTGTTTCTTCTATGCCTAGTGATTTAATAGTCCCAGATG

ACCTCCCGAAGGAAGAAGCTCCAATTGTCACACAAACATCTAGTTCAACACCAGATCCAAGTTCTTCAACTCTCTCAGAGA

AAAGTCTTAGGAAATCCGAGGACCATGTTGAGAACAATCTGTCAGCAAAGGAGCCAAAGATGAGAAAAGAACACTCCACC

ACAAGGGTCAATGAATATTCCGTTTCCTCTGTATCTAGTGATTCTATGGTCCCAGATCAAGCCCTCAAGGAAGAAGCTCCTAT

TTCCATGAAGATATCCAATTCAACACCAGATCCAAAATCCTTGGTTTATCCAGAAAAAAGTCTTAGAACATCCCAGGAGAAA

ACGGGTGCCTTCGATACAACAAATGAAGGCATGAAAAAGAATCAGGACAATCAATTTTGTCTGCTTGGAGGATTCTCAGTAT

CTGGACATGGTACTTCAAATAATAGTTCATCTAATGTGAGCAATTTCGACCAGCCTGTGACTCAGCAAAGAGTCTTTCATTCT

GAGCGAACTGTACGAGATCCAACAGAAACTAACCGTTTGTCTAAATCTGATGATTCCCTTGCTTCTCAATTTGTAATGGCTCA

AACAACATCAGATGCTTTCCTGCCTATCAGCGAATCATCTGAAACTTCTCATGAAGCAAATATGGAGTCCCAGAATGTTCATC

CTACTGCGCCAGTAATACCAGCTCCTGATAGCATCTGGACAGCCGAGGGTAGTATGTCACAGTCTGAAAAAAAAAACGTGG

AAACTAACACCCCGGAGCATGTAAGTCAGACAGAGACTTCAGCAAAGGCTGTTCCACAAGGACACAATGAGAAGGGGGAT

ATAGTTGTTGATATAAATGATAGGTTTCCTCGTGAGTTTCTTGCTGATATATTAAAAACGAAAGAGTCTCTGAACTTCCCTGGA

TTAGGGCCATTGCATGCCGATGGAGCTGGTGTGAGTTTAAATATTCAGAATAATGACCCTAAAACTTGGTCGTATTTTCGAAA

TTTGGCGCAGGATGAGTTTGAGAGGAAGGATCTATCCCTTATGGATCAGGACCACCCTGGATTTCCCACTTCCATGACTAACA

CCAACGGAGTTCCTATTGATTATAGCTACCCACCATTGCAGTCTGAGAAAGTTGCCTCAAGTCAGATACATCCACAAATCCAC

TTTGATGGAAATATCAAGCCAGATGTGTCTACCATTACCATACCTGATTTGAACACAGTAGACACACAAGAAGATTACAGTCA

GTCACAAATCAAAGGTGCTGAAAGCACGGATGCAACTCTGAATGCTGGAGTTCCTCTTATTGACTTTATGGCTGCGGATAGT

GGCATGAGGTCTCTGCAGGTCATTAAAAATGACGACTTGGAAGAACTGAAGGAATTAGGTTCTGGTACTTTTGGAACTGTTT

ATCACGGAAAATGGAGGGGTACAGATGTTGCTATCAAGCGAATAAAAAGGAGCTGTTTTATTGGTCGTTCATCTGAACAAGA

GAGATTGACCTCGGAGTTCTGGCATGAAGCAGAAATTCTTTCAAAGCTTCATCATCCAAATGTTATGGCATTTTACGGCGTAG

TGAAAGATGGACCAGGAGGAACTTTAGCTACAGTGACAGAGTACATGGTCAATGGATCGCTCAGGCATGTTCTGCTCAGCA

ACAGGCACCTTGATCGACGTAAGCGACTTATCATTGCAATGGACGCAGCTTTTGGGATGGAATATTTGCACTCAAAGAGCAT

AGTGCATTTCGATTTGAAGTGTGATAACTTGCTTGTCAACTTAAAGGATCCCGCCCGTCCCATATGCAAGGTTGGTGATTTTG

GTCTGTCAAAGATAAAAAGAAACACTTTGGTCACTGGCGGTGTAAGGGGAACCCTCCCTTGGATGGCTCCCGAGCTACTTA

GTGGAAGCAGCAGCAAAGTTTCTGAAAAGGTTGATGTGTTCTCTTTCGGAATTGTCTTATGGGAAATTCTTACCGGTGAGGA

ACCCTACGCCAATATGCATTATGGGGCAATAATCGGAGGCATAGTGAACAATACATTGAGACCAACCGTGCCAAACTACTGT

GACCCGGAGTGGAGAATGCTGATGGAGCAGTGTTGGGCTCCTGACCCATTTGTTCGACCTGCGTTCCCGGAAATAGCCAGA

CGTCTCCGCACCATGTCCTCCTCTGCGGTCCACACAAAACCACACGCTGTCAACCACCAAATCCACAAGTAA

>AtRAF25 (AT2G43850)

ATGGAGAACATAACCGCGCAGCTCAAACGAGGTATCTCAAGACAATTCTCAACTGGTTCGATTCGTCGAACATTGAGCCGAC

AGTTCACGCGTCAGTCATCTCTTGACCCTCGTAGGACCAACATGAGGTTTAGTTTCGGTCGTCAGTCTTCTCTCGATCCGATC

AGGAGGAGTCCTGATTCTTCCAAGAGTGATGACGAGCCACATATGTCGGTACCGGAGAATCTCGATTCGACGATGCAGCTTC

TCTTTATGGCTAGCAAAGGTGATGTCAGAGGAATCGAGGAGCTTCTTGACGAAGGAATTGATGTTAATAGCATCGATCTTGAT

GGCCGGACGGCGCTTCACATCGCCGCTTGTGAGGGACATCTTGGTGTCGTTAAGGCTCTTCTTAGCCGGAGAGCTAACATTG

ATGCTCGTGATCGATGGGGAAGTACGGCGGCTGCTGATGCAAAGTACTATGGGAATTTAGATGTTTACAATCTCTTGAAGGCT

CGAGGAGCTAAAGTTCCGAAAACTAGGAAGACTCCGATGACTGTGTCGAATCCACGAGAAGTTCCTGAGTATGAGCTTAAT

CCACTTGAGGTTCAAGTCAGGAAATCTGATGGCATCTCAAAGGGAGCATATCAAGTAGCTAAATGGAATGGCACGCGGGTTT
CAGTCAAAATACTTGATAAAGATAGTTACTCAGACCCGGAACGCATAAACGCATTTAGACATGAATTGACGTTGCTAGAAAA
AGTCCGGCATCCAAATGTTATCCAGTTTGTTGGAGCTGTCACTCAGAATATACCGATGATGATTGTAGTCGAGTATAATCCGA
AAGGAGATTTAAGTGTATATCTCCAAAAGAAAGGACGTCTTTCTCCATCCAAGGCACTTAGATTTGCTCTTGATATTGCCAGA
GGCATGAACTACCTCCATGAATGTAAACCAGATCCAATCATTCACTGTGATCTAAAGCCAAAAAATATTTTGCTGGATAGAGG
AGGGCAATTAAAGATCTCAGGATTTGGTATGATAAGATTGTCGAAAATTTCACAAGACAAGGCGAAAGTAGCAAACCACAA
AGCACATATAGATCTCTCTAATTACTACATTGCACCAGAGGTTTATAAAGACGAAATATTCGACCTAAGGGTTGATGCACACT
CCTTTGGTGTCATTTTATACGAGATAACAGAGGGAGTACCAGTTTTCCATCCGAGACCTCCTGAAGAAGTTGCGAGAATGAT
GTGCTTAGAAGGAAAGAGACCTGTATTCAAAACTAAGTCAAGAAGTTACCCTCCAGATATAAAAGAGTTGATCGAGAAATG
TTGGCATCCAGAAGCGGGTATTAGACCGACATTCTCTGAAATTATTATTCGACTTGACAAAATAGTAGCAAATTGCTCTAAAC
AAGGATGGTGGAAAGACACATTCAAGTTCCCTTGGAAATAA

>AtRAF26 (AT4G14780)

ATGGAGAAGAAATCAGAAGAAGATGGGAACAACACGACGAAAGAAAAGATCTTTAGAGCGGATAAGATTGATTTGAAGAG
TTTAGATAGACAGCTTGAGAAACATCTGAGTAGGGTTTGGTCGAGGAACCTTGAGGTGAATCCTAAAGCTAAGGAAGAATG
GGAGATTGATTTGGCTAAGCTTGAAACAAGTAATGTTATTGCTCGTGGTACTTATGGTACTGTCTACAAAGGCATTTATGATGG
ACAAGATGTTGCAGTGAAGGTGCTTGATTGGGAAGATGATGGGAATGAAACAACGGCCAAGACCGCTACAAATCGAGCTTT
GTTCCGTCAAGAGGTCACTGTTTGGCACAAACTCAACCATCCAAATGTCACAAAGTTTGTTGGAGCGTCGATGGGAACAAC
GAATCTTAATATACGATCAGCTGATTCGAAAGGCTCGTTGCCTCAACAAGCATGTTGTGTGGTTGTGGAATATCTTCCTGGTG
GAACATTGAAACAACACTTGATTCGTCATAAGAGCAAGAAACTCGCTTTTAAAGCCGTTATCAAACTCGCTCTTGATCTCGC
CAGAGGGCTAAGCTATTTGCACTCAGAGAAGATTGTCCACCGCGATGTGAAAACAGAGAACATGCTTTTGGATGCTCAGAA
GAATTTGAAAATAGCGGATTTTGGAGTAGCACGAGTGGAAGCTCTTAATCCAAAAGACATGACAGGAGAAACCGGTACTCT
TGGATACATGGCTCCTGAGGTCATTGATGGTAAGCCATACAACAGAAGGTGCGATGTTTACAGCTTTGGGATATGTTTATGGG
AAATCTACTGCTGCGATATGCCTTATCCTGATCTTAGCTTTGTCGATGTTTCTTCCGCGGTTGTCTTACATAATCTGAGACCGG
AGATACCGAGATGCTGTCCGACTGCATTGGCGGGCATAATGAAGACATGTTGGGATGGGAATCCGCAGAAACGGCCGGAGA
TGAAGGAGGTGGTGAAAATGCTTGAAGGTGTTGATACCAGTAAAGGTGGCGGAATGATACCGGAAGATCAAAGTCGGGGCT
GTTTCTGCTTTGCTCCTGCTCGTGGACCTTAA

>AtRAF27 (AT4G18950)

ATGGAAGAGGATTATCAACAGCCGAGGTTTACGATTGGTAGGCAATCATCAATGGCGCCGGAGAAGATTCCGGAGCCGTCG
GTTCACTCAGAAGAAGAGGTGTTTGAGGATGGAGAAGAGATCGATGGTGGTGTGAGACTAATGTATTTGGCTAATGAAGGT
GACATTGAAGGGATTAAGGAGCTTATTGATTCAGGGATTGATGCTAATTACAGAGACATTGATGATCGGACTGCTTTACACGT
GGCGGCTTGTCAAGGATTGAAAGATGTTGTTGAGCTTCTTCTTGATCGGAAAGCTGAGGTTGATCCTAAAGATCGTTGGGGA
AGCACTCCATTTGCAGATGCGATATTTTACAAGAACATTGATGTTATCAAGATTCTTGAGATACATGGAGCTAAACATCCGATG
GCTCCAATGCACGTTAAGACTGCTCGTGAAGTCCCTGAGTATGAAATAAATCCTAGTGAGCTTGATTTCACTCAAAGCAAAG
AGATAACAAAGGGAACTTACTGTATGGCAATGTGGCGTGGTATTCAAGTTGCGGTGAAAAAGCTGGATGATGAAGTTTTGAG
CGATGACGATCAAGTGAGGAAGTTCCATGATGAGCTTGCATTGCTCCAAAGGCTTAGGCATCCAAACATTGTGCAGTTTCTT
GGTGCTGTAACCCAAAGTAACCCAATGATGATTGTGACTGAATATTTGCCCAGGGGGGATTTGCGTGAATTGCTCAAACGAA
AAGGACAATTGAAACCAGCTACTGCTGTTAGATATGCCCTTGATATTGCTAGGGGAATGAGCTATCTTCATGAGATCAAAGGA
GACCCTATAATCCACCGCGATCTTGAACCTTCAAACATTCTGCGGGATGATTCAGGGCATCTGAAAGTTGCAGACTTTGGAGT
AAGCAAGCTTGTTACTGTTAAAGAAGACAAGCCTTTTACATGTCAAGACATTTCTTGTCGATATATAGCTCCCGAGGTTTTCA
CTAGTGAAGAATACGATACAAAAGCTGATGTTTTCTCATTTGCATTGATCGTTCAGGAGATGATCGAAGGCCGAATGCCGTTT
GCTGAAAAGGAAGACAGTGAAGCTTCTGAAGCTTATGCTGGCAAACATCGGCCATTATTCAAAGCTCCATCAAAGAATTACC
CACATGGTCTTAAAACGTTGATAGAAGAATGCTGGCATGAGAAACCTGCAAAGCGACCAACTTTCAGAGAGATCATTAAAC
GACTTGAGTCCATTCTTCACCACATGGGTCACAAGCGACAATGGAGGATGAGGCCATTGACATGCTTTCAGAATTTCGAGCA
CAAGAAGAAACATAATTGGGATTTGAGCAGCCATGACGGCTCATCATCCGGTTCACATTTGTGA

>AtRAF28 (AT4G31170)

ATGCTTGAGAATCCAAAGTTCGATTTGCACGCTGTTGGCAATCACAACAACGACAACAATTACTATGCCTTCACCCAAGACT
TTTATCAAAAGCTCGGGGAAGAAGGTACAAACATGTCTGTTGACAGTATGCAGACAAGTAATGCTGGAGGGTCTGTGTCAAT
GTCTGTCGATAACAGTAGCGTTGGTTCGAGTGATGCTCTTATTGGCCATCCTGGTTTGAAGCCTATGCGCCATCCCTACTCTCT
CTCGGATGGCCAAAGCGTATTTCGGCCAGGAAAAGTTACTCATGCACTTAACGATGATGCCTTAGCACAAGCGTTGATGGAT
AGTAAGTATCCAACCGAGGGACTGGTGAACTATGAAGAGTGGACAATAGATCTGAGGAAACTACATATGGGTCCTGCTTTTG
CTCAAGGGGCTTTTGGAAAGTTATACAGAGGGACTTACAACGGAGAAGATGTAGCCATTAAGCTACTCGAGAGGTCAGATA
GCAACCCTGAAAAGGCACAAGCCCTCGAACAGCAGTTTCAGCAGGAAGTTTCTATGCTTGCATTTTTGAAGCATCCTAACAT
CGTTAGGTTTATTGGTGCGTGCATTAAACCGATGGTGTGGTGCATCGTGACTGAATATGCAAAAGGAGGGTCTGTCAGACAG
TTTCTGACTAAGAGACAAAACCGAGCTGTGCCTTTGAAGTTAGCTGTTATGCAGGCGTTGGATGTTGCCAGGGGTATGGCTT
ACGTCCATGAGCGCAACTTTATACACCGGGATCTAAAGTCAGATAACCTCCTCATATCAGCTGATCGGTCCATCAAGATTGCT
GATTTTGGTGTTGCAAGAATTGAAGTTCAAACCGAAGGGATGACACCAGAGACTGGAACTTACAGATGGATGGCACCAGAG
ATGATCCAGCACAGACCCTACACTCAAAAAGTGGACGTGTATAGTTTTGGAATCGTGCTGTGGGAGTTGATTACAGGTCTGT
TACCGTTCCAGAACATGACGGCGGTTCAGGCTGCATTTGCAGTGGTGAACAGAGGAGTCCGTCCAACAGTCCCAGCAGATT
GTCTTCCTGTGCTTGGAGAGATCATGACACGTTGCTGGGATGCGGACCCTGAAGTCCGTCCTTGTTTTGCAGAGATTGTCAA
TCTTCTGGAGGCGGCGGAAACTGAGATAATGACGAATGTGAGAAAAGCCCGTTTCAGATGTTGCATGACGCAACCAATGAC
AGTCGACTAA

>AtRAF29 (AT4G35780)

ATGGCGATCAAAGAGGAGACGGAGGAGAGTTGCGGAAGCAGAGCCGTGGTAGCTTCAATAACGAAAGAAAGCCCTAGACA
GCACCGTATGAAACTGGAGGTGTACGGTGAGGTTCTTCAACGAATCCAGGAATCCAATTACGAAGAAGCTAATTTCCCTGGT
TTTGATGATCTCCTCTGGCTTCACTTCAATCGTCTTCCTGCTCGATATGCTTTGGATGTAAATGTTGAGAGAGCAGAAGATGTA
CTTACTCATCAGAGATTGTTGAAATTGGCTGAAGATCCTGCTACTAGACCTGTTTTCGAAGTTCGTTGTGTGCAGGTTTCTCC
CACATTGAATGGAAATTCTGGTGACGTTGATCCTTCGGATCCTGCGGTCAATGAAGATGCTCAAAGCTCCTATAACTCGAGGT
CTCTTGCACCTCCAACTTTTGGTTCTTCTCCGAATTTCGAAGCTCTTACTCAAGCTTACAAAGATCATGCTCAAGACGATGAT
AGTGCTGTCAATGCACAGTTGCCTAATTCTCGACCGATGCACGAAATCACCTTTTCTACAATCGACAGGCCGAAACTCCTTA
GTCAGCTAACTTCCATGCTTGGTGAACTTGGATTGAATATTCAAGAGGCTCATGCTTTCTCTACCGCCGATGGTTTCTCTCTCG
ATGTATTTGTTGTTGATGGCTGGTCGCAGGAGGAAACAGAAGGTTTAAAAGATGCATTGAAGAAGGAGATAAGAAAGTTTA
AGGATCAACCTTGTTCAAAACAGAAATCTATCACTTTCTTTGAGCATGACAAATCAACCAACGAGCTGCTACCTGCGTGTGT
TGAAATACCTACGGATGGAACTGATGAGTGGGAAATTGACATGAAGCAGCTCAAAATTGAAAAAAAGGTGGCATGTGGATC
ATACGGGGAACTATTTAGAGGAACCTATTGTAGTCAGGAAGTAGCTATCAAAATTCTCAAGCCTGAGCGTGTTAATGCGGAA
ATGCTACGAGAGTTTTCTCAGGAAGTATATATAATGAGGAAAGTTCGGCATAAAAATGTTGTCCAGTTCATTGGTGCATGTAC
ACGATCACCAAACCTCTGCATTGTGACAGAGTTCATGACTCGGGGGAGCATTTATGATTTCCTTCACAAACACAAAGGGGTT
TTTAAAATTCAATCTTTGCTCAAAGTGGCACTCGACGTCTCGAAAGGAATGAATTATCTGCATCAAAACAATATTATTCATAG
AGACCTTAAGACTGCTAATCTTCTTATGGACGAACATGAAGTTGTCAAAGTTGCCGATTTTGGTGTTGCCAGAGTGCAGACT
GAGTCAGGGGTTATGACAGCGGAAACAGGGACATACCGATGGATGGCTCCAGAGGTCATTGAGCACAAACCTTATGATCAC
AGGGCAGATGTCTTCAGCTACGCGATTGTGCTGTGGGAACTTTTGACTGGGGAACTCCCATATTCTTACTTGACTCCACTGCA
AGCTGCTGTTGGCGTTGTCCAAAAGGGACTTAGACCAAAAATTCCAAAGGAAACACACCCAAAACTGACTGAACTTCTTGA
GAAATGCTGGCAGCAAGACCCAGCTCTAAGACCCAATTTTGCAGAAATCATAGAAATGCTTAACCAACTAATCCGCGAGGTT
GGAGATGATGAGCGCCACAAGGATAAACATGGTGGTTACTTTTCAGGCCTAAAAAAAGGCCATCGTTGA

>AtRAF30 (AT4G38470)

ATGGTGATGGAGGACAACGAGAGTTGCGCTAGTAGAGTTATTTTCGACGCCTTACCTACCTCCCAGGCCACTATGGACCGCC
GTGAGCGCATCAAAATGGAGGTCTTCGATGAGGTTCTCCGCCGTCTCCGTCAATCGGACATTGAAGACGCCCATCTCCCTGG
TTTCGAAGACGACCTCTGGAATCACTTCAATCGCCTCCCTGCCAGGTATGCTTTGGATGTGAATGTGGAGAGGGCTGAAGAC
GTCTTGATGCACAAGCGATTGCTGCATTCTGCTTACGATCCCCAGAATCGGCCTGCTATCGAAGTTCATCTCGTCCAGGTTCA

ACCTGCCGGGATCTCTGCTGACTTGGACTCTACTTCTAATGATGCTGGTCATTCTTCTCCTACCCGAAAAAGCATTCATCCGC
CGCCTGCCTTTGGTTCATCCCCTAATCTTGAAGCACTTGCACTTGCAGCTAGTTTATCCCAAGACGAGGATGCAGACAACTCT
GTTCATAACAATTCACTCTATTCACGGCCCTTGCATGAGATAACCTTTTCCACAGAAGACAAGCCTAAACTCCTTTTTCAGTT
AACTGCCTTGCTTGCTGAGCTTGGGCTGAACATTCAAGAGGCACATGCTTTCTCTACAACTGATGGCTACTCACTAGATGTTT
TTGTTGTTGATGGCTGGCCGTACGAGGAAACAGAGAGACTTAGGATATCATTGGAGAAAGAAGCAGCAAAGATCGAGTTGC
AGAGCCAAAGCTGGCCTATGCAGCAATCCTTCTCTCCGGAAAAGGAAAATGGGCAAACAGGTGCCAGAACGCATGTTCCAA
TACCCAATGACGGAACTGATGTTTGGGAAATCAACCTTAAACACCTAAAATTTGGGCATAAAATAGCGTCTGGTTCTTATGGA
GATCTGTATAAAGGTACATACTGTAGCCAGGAAGTTGCTATCAAAGTCCTAAAGCCAGAGCGTCTAGACTCAGATCTAGAGA
AAGAGTTTGCCCAAGAAGTCTTTATTATGAGGAAAGTTAGACACAAAAATGTTGTTCAGTTCATTGGTGCTTGCACCAAGCC
TCCACATCTGTGTATCGTTACAGAATTCATGCCCGGTGGAAGTGTATATGACTATCTACACAAGCAAAAGGGCGTCTTTAAGC
TTCCAACTTTGTTTAAAGTAGCTATAGATATTTGCAAAGGGATGAGCTACTTACACCAAAATAACATAATTCACAGAGATTTG
AAGGCTGCCAACCTCTTAATGGACGAAAATGAGGTGGTTAAGGTTGCAGACTTTGGGGTGGCTAGAGTGAAAGCACAAACT
GGAGTTATGACAGCTGAAACTGGAACATATCGCTGGATGGCTCCAGAGGTGATAGAACACAAGCCATATGATCACAAGGCTG
ACGTATTCAGCTACGGGATTGTGCTATGGGAGTTGTTGACTGGGAAGCTTCCATATGAATACATGACGCCGTTGCAGGCAGC
AGTAGGGGTTGTCCAAAAGGGATTAAGGCCAACAATACCAAAGAACACGCATCCGAAATTGGCAGAGCTATTGGAGAGATT
GTGGGAGCATGATTCGACGCAGAGACCAGACTTCTCAGAGATCATAGAGCAGCTTCAAGAGATAGCCAAGGAGGTAGGAG
AAGAGGGAGAGGAGAAGAAAAAGTCGTCAACAGGACTAGGAGGGGGTATATTTGCAGCCCTGAGGAGAAGCACCACACAT
CATTGA

>AtRAF31 (AT5G01850)

ATGAGTAGCGATGATACGATTGAGGAGAGTTTGCTTGTGGATCCCAAATTGTTGTTCATCGGCTCCAAGATTGGTGAAGGCG
CTCACGGCAAAGTCTACCAAGGAAGGTATGGTCGTCAGATTGTTGCAATCAAAGTTGTCAACCGGGGCTCCAAACCTGACC
AGCAATCTTCTCTCGAGAGCCGTTTCGTCCGTGAGGTCAATATGATGTCCCGCGTTCAACACCATAACCTTGTCAAGTTTATT
GGAGCGTGCAAAGATCCTTTAATGGTAATAGTAACAGAGCTTCTCCCAGGGATGTCTCTCCGTAAATATCTCACCAGCATCCG
TCCTCAGTTGCTCCATCTCCCTCTTGCTCTCTCCTTTGCCCTTGACATCGCCCGTGCCTTGCACTGCTTACACGCCAATGGTAT
CATTCACAGAGACCTCAAACCTGACAACTTATTGCTCACGGAGAATCACAAATCCGTCAAGCTTGCTGATTTCGGGCTTGCT
AGGGAAGAATCCGTGACTGAGATGATGACTGCTGAGACTGGGACTTACCGTTGGATGGCTCCTGAGCTCTACAGTACAGTG
ACCCTGCGTCAAGGAGAGAAGAAGCATTACAACAACAAAGTTGATGTCTACAGCTTTGGAATCGTGCTTTGGGAGCTTCTC
ACTAATCGTATGCCATTCGAGGGCATGTCCAATCTGCAAGCCGCCTACGCAGCAGCATTCAAGCAGGAGAGGCCCGTAATGC
CAGAGGGGATATCTCCGAGTCTGGCGTTCATAGTGCAGTCTTGTTGGGTGGAGGACCCAAACATGAGGCCAAGCTTCAGTC
AAATTATCAGACTGCTCAATGAGTTCCTCCTTACCCTGACTCCTCCGCCTCCTCAGCCTCTGCCTGAGACTGCCACCAACAG
GACCAATGGCCGAGCCATCACTGAGTTCTCCATCCGTCCAAAAGGGAAATTTGCCTTCATTCGCCAGCTTTTCGCTGCCAAG
AGGAACATAAACTCTTAG

>AtRAF32 (AT5G40540)

ATGGGATCTGTTACTGGGTTTTACTCAAATGAAGTGTTTGAATTAGATCCTAAATGGGTTGTTGATCCTCAACATCTCTTTGTT
GGTCCCAAGATTGGTGAAGGTGCTCATGCCAAAATCTATGAAGGAAAGTATAAGAACAAAACAGTTGCTATTAAGATTGTTA
AAAGAGGAGAATCTCCCGAAGAGATTGCGAAAAGAGAGAGCAGATTTGCAAGAGAGGTCTCTATGTTGTCTAGAGTTCAAC
ACAAAAATTTGGTCAAGTTCATTGGAGCTTGCAAAGAACCAATCATGGTTATAGTCACCGAGCTTTTACTCGGTGGTACATTG
CGTAAATACCTTGTTAGCTTGCGTCCAGGGAGTTTGGACATACGTGTAGCTGTTGGATATGCCCTTGACATTGCTCGGGCGAT
GGAATGCTTACACTCTCATGGAGTCATCCATCGCGATCTCAAACCAGAGAGCTTGATCTTAACTGCGGATTACAAGACTGTTA
AACTAGCAGATTTTGGTTTAGCTAGAGAAGAATCATTAACCGAGATGATGACCGCAGAAACTGGTACATATCGTTGGATGGC
TCCCGAGCTTTACAGTACGGTCACGTTGAGGCATGGTGAGAAAAAACACTATAACCATAAGGTAGATGCATACAGCTTCGCC
ATTGTCTTGTGGGAGCTTATCCACAACAAATTACCTTTTGAAGGCATGTCAAATCTCCAAGCCGCTTACGCTGCTGCATTCAA
GAACGTGAGGCCAAGCGCAGACGATTTACCAAAGGATTTAGCAATGATTGTAACATCTTGCTGGAAAGAGGATCCAAACGA
TCGACCAAACTTCACAGAGATTATTCAAATGCTTCTACGTTGCCTCTCCACAATCTCATCAACTGAGCTTGTTCCTCCGGCCA

TTAAACGTGTTTTCTCGTCGGAAAACACGGTCTTGCCACCAGAATCGCCGGGAACTTGCTCATTGATGACTGTTAGAGACAA
AGATCAGATTCCTACCGATGCAAACTCGGCGCAGAATGAAGTTAGAGGAAGCTTCTTCTTCTTCTGCTGCTAA

>AtRAF33 (AT5G50000)

ATGAAAGAAGGAAAGGATGGGTTTGTTCGAGCAGATCAGATTGATCTAAAAAGCTTAGACGAGCAACTAGAGAGACACTTA
AGCAGAGCTTTGACTTTAGAGAAGAACAAGAAGAAAGATGAAGAAGACACAACCGCCGTCGCCATCGGTGGCTCTGCTTC
TTCTTCCCCTGTCACTCTAAACGGCGGCGGATTTGTTGGCAAGAGGAAACAGAGACTTGAGTGGGAGATCGATCCTTCTAA
ACTTATCATCAAGACTGTTCTTGCTCGTGGCACTTTTGGTACCGTTCATCGTGGTATCTACGACGGTCAAGATGTTGCCGTGA
AGTTGCTTGATTGGGGTGAAGAAGGTCATAGATCAGAAGCTGAGATTGTGTCTTTGAGAGCTGATTTTGCTCAGGAGGTTGC
TGTTTGGCATAAATTGGATCATCCTAATGTCACCAAGTTTATAGGAGCAACAATGGGTGCATCAGGGTTACAGTTGCAAACAG
AGAGTGGTCCATTAGCTATGCCAAACAATATATGTTGTGTTGTTGTTGAATATCTTCCAGGTGGTGCTTTAAAATCTTACCTGA
TCAAGAACAGAAGAAGAAAACTAACATTTAAAATTGTTGTTCAGCTCGCTCTTGATCTCGCTAGAGGGTTGAGTTACCTTCA
TTCGCAGAAGATTGTTCACCGAGATGTAAAGACAGAGAATATGTTGTTAGACAAAACCCGGACTGTTAAAATCGCAGATTTT
GGAGTTGCGAGAGTTGAAGCATCTAACCCTAATGACATGACAGGAGAGACTGGCACACTTGGCTACATGGCGCCTGAGGTT
CTCAATGGAAACCCGTATAATCGAAAATGCGATGTATACAGTTTCGGGATCTGCTTATGGGAGATATATTGCTGTGACATGCCA
TACCCTGACCTTACTTTCTCTGAGGTCACTTCAGCCGTCGTCCGCCAGAACCTGAGACCCGATATACCGAGGTGTTGCCCGA
GCGCACTTGCAGCTGTGATGAAGCGATGTTGGGATGCAAATCCGGACAAGAGACCGGAGATGGATGAGGTTGTACCAATGT
TGGAGAGTATTGACACAACCAAGGGAGGAGGGATGATTCCTAACGACCAGCAACAGGGTTGCCTATGCTTCCGAAGGAAAC
GTGGTCCTTAA

>AtRAF34 (AT5G50180)

ATGGATTCTTTGACTGGATTTAGAATGGAACCCAAATGGCAAATTGATCCTCAGCTTCTCTTTGTTGGTCCAAAGATTGGTGA
AGGAGCTCATGCTAAAGTCTATGAGGGAAAATACAAGAATCAGACAGTTGCTATAAAGATAGTTCACAGAGGAGAAACACC
AGAAGAGATTGCTAAAAGAGATTCAAGATTCCTTAGAGAAGTAGAAATGCTCTCACGTGTTCAACACAAGAATTTGGTCAA
GTTCATTGGTGCTTGCAAGGAGCCTGTAATGGTGATAGTTACAGAACTTCTTCAAGGCGGTACATTGCGTAAATATCTATTAA
ACTTGAGACCCGCATGTTTGGAGACTCGTGTGGCTATCGGTTTTGCGCTTGATATTGCTCGTGGTATGGAATGCTTGCATTCC
CATGGGATCATTCACCGTGATCTCAAACCCGAGAACTTGCTTTTAACTGCAGACCATAAAACAGTAAAACTAGCAGATTTTG
GATTAGCAAGAGAAGAGTCACTGACTGAGATGATGACGGCTGAGACAGGAACATACCGATGGATGGCACCTGAGTTGTACA
GCACGGTAACTCTTCGATTGGGAGAGAAGAAGCACTACAATCATAAAGTCGACGCCTATAGCTTTGCAATAGTTCTTTGGGA
GCTTTTACACAATAAATTGCCTTTTGAGGGAATGTCAAATCTCCAAGCAGCATATGCAGCAGCCTTCAAAAATGTGAGACCA
AGCGCGGAGAGCTTACCGGAGGAGTTGGGCGACATTGTAACATCATGTTGGAATGAGGATCCGAATGCTCGGCCCAACTTC
ACGCATATCATTGAGTTACTTCTTAACTATCTCTCTAAAGTTGGATCTCCGATATCCGCTATTCCACAACGGATTCTTGCTTCGA
AGAACACGCTATTGCCACCGGATTCTCCTGGAACTAGCTCTTTGATGGCTAAGCTTGATGAATGTGGGGAAACTCCAAAGGC
CAAAAGTGAAGACAAACGAAAAGGTTTGTTTTTCTGCTTCAATCAATGTTATTAA

>AtRAF35 (AT5G57610)

ATGGATTCAGGTTCTGTGAACTCATCTGTCACTAGTCTAGTGTCGAGTTTGAACGATGAGCCGCATCGTGTTAAATTCCTGTG
TAGCTTCTTAGGGAGTATATTGCCTCGTCCTCAGGATGGGAAATTGAGGTATGTTGGTGGAGAGACGAGGATTGTGAGTGTG
AATAGAGATATTAGGTATGAGGAGTTGATGAGTAAGATGAGAGAGCTTTATGATGGTGCAGCAGTGTTGAAGTATCAGCAGC
CTGATGAGGATCTTGATGCTTTGGTCTCGGTTGTGAATGATGATGATGTGACGAATATGATGGAGGAGTATGATAAGTTAGGT
TCAGGAGATGGGTTTACTAGGCTTAGGATCTTTCTGTTTTCTACTCCTGAGCAGGATGGTTCTTTGCATTATGTGGAGCGTGAT
GATCAGAGGGAATCTGAGAGGAGATATGTTGATGCTCTTAATAATTTGATCGAAGGGACAGACTTTAGAAAGTTGCAGCAGT
ATCCTGATTCACCTCGTTTCAATCTCGTTGATGATTTTTCGATGGTGGAACCGATGCTGAATCAGCTGAGCATTGAGACTGGT
GGTGGTAGCCAGAGGGGCAACGAGATACCTACTGCGCAGTATAGTAATCTTCACCAGCTCAGGATTCCTCGTGTGGGTTCGG
GGCAGATGCTTGCACAGAGGTATGGTGAAGTAGAAGGTACATGGAGTCCTTTCTATTCTCCTCGGCACCATGGACATCATGAT
CCCAGAACTTTTCAGGAATTTCCATCTTCGCCTTCTTCTGCTCGTTATCGAATGCCATATGGGGAAATTCCTGATAAGGGATTG
GATAGAATGCCTGAGGAGTATGTTAGGCCGCAAGCAAGTCACCATCCTTTTTACGAGCACCAGGCACACATTCCTGACAGTG

TGGTGTGGGTCCCAGCTGGAGCAATGCCACCTGAGAGTAAAGGAGGGTTTCCTGGGAATGTTCTTCACGGTGGTCCAGGTG

GCTATGAAGGTGGTAATGGATGCGAGAACTGTCGTGTACCGTATCATAGGAACCATCAGCTTTTAGAGCAATCTAATATAGGC

AACAATGGATTTCCACCGGTTCATTGTGCACATTGTCCACCAAACAGGGAAAGTTTCCTGCTTAACACAGACCCAAAGCCTA

CTCATCATGGAGCTTATCCAAATGAGACTTTTGGACCTGATAGAGGATGGATGGTTCAACAACAGGTGAATCCTAACCCTCC

AAGGATTGAGGAAGGAAGGTCACATATTTCTAATGTTGGAAGACCGAACGATCATTACACTCCCGATTATCCTGTGTCGAACT

ATCCTCTTGGTCAACGAGCTGGACATGAAATTTCCAATGAAGGGTTTCATGATAAACCACTTGGTGGCATTCCCCTGAATTCG

GCCAACCGTTCTGCTGAGGAACGTGGGTTTCATTATGGGAATAATCTCTATCCTCCAGGACCTGATAGTATCCACTCAGCAGG

TCATAGCCATATGCACCACCCTCAACCAAATATTTGGCAGAACGTTTCAAATCCTATAGCTGGCCCTCCAGGCTTGCCCATGC

AAATTAATGGCACAGTCAATCAGACCGTTATCAGGAATCCGATAGAAACTGCTCCTAGGTATTCTACTGGAATGGAGAATCAA

GGTGTTTTGGTTGGTTCTCCGCAAAGGATTTCAGGCTTTGATGGAATGTCCTCCCTTGGCCAGCCTTCCTACCCTAATCCCCA

TTTGCAAGATAGAGCCTTCCCTTTAGACCCAAATTGGGTGCCATCTGAAAACCCAACTGTACATAATGAACATCTACAGGTG

CGTGAGCCTCTGCCTGGGCCATTATTACAGACAAACCTCACTGCTGCACCAATCATGCAGACACCAGTTATGCAGACATCTG

TGGAAAGCAAGCTTGCTCAGGGAGGTGAACAATTTAACTATGTTAATACTGGTATTTCAAACGGTGTTCCTTACCAAGATAA

GCCTCAACCCTTAGCTGGAGGAAAGAAAGATATGGGGAACCTGGTAGAAGTTAATCCTTCTGCTGCTACTCTTGAAGGGGC

AGAGCTATCAGTTGAGCGTTTGAGTTTCTTGCCTGAGTTAATGGAATCTGTGAAGAGGGCGGCACTTGAAGGAGCTGCTGA

GGTTAAAGCTCATCCAGAAGAGGCCAAAGATCAAGTGAGGCCGGAGTTGGTGGAAAATGAATCAGAGCATATGAATGCTCA

AGACGAACCTGAGATAGATTCTGATAGTGATAACCCAAACAATTTCAAGATTGAACAAACAAAGGCTGAGGCTGAAGCTAA

ATCCAGGGGACTACAGTCTATAAGAAACGATGATTTGGAGGAAATCCGAGAGTTAGGCCATGGAACATATGGATCTGTTTAC

CATGGAAAATGGAAAGGCTCAGATGTTGCAATAAAGAGAATAAAAGCCAGCTGCTTTGCAGGCAAACCTTCGGAAAGGGA

ACGTTTGATAGAAGATTTTTGGAAAGAAGCCCTGTTATTGAGCTCATTGCATCATCCAAATGTTGTGTCGTTCTATGGGATAGT

TCGTGATGGTCCTGATGGTTCTCTAGCAACTGTCGCGGAGTTCATGGTTAACGGATCTTTGAAACAGTTCTTGCAGAAGAAG

GATAGAACTATTGATCGTCGCAAAAGACTCATCATAGCCATGGATACTGCATTTGGGATGGAGTATTTGCACGGAAAGAACAT

TGTTCATTTCGATTTGAAGTGTGAGAATCTTCTTGTGAACATGAGAGATCCTCAGCGTCCTATATGCAAGATTGGTGATTTGG

GGTTATCGAAGGTGAAACAGAAGACTCTAGTATCAGGAGGTGTTCGTGGGACTTTGCCATGGATGGCACCTGAGCTCTTGA

GTGGGAAAAGCAACATGGTCTCTGAAAAGATCGATGTTTACTCGTTTGGGATTGTAATGTGGGAACTACTCACTGGTGAAGA

ACCATACGCAGACATGCATTGTGCTTCCATAATTGGAGGTATAGTGAACAACGCATTGCGTCCAAAAATCCCACAGTGGTGT

GATCCAGAGTGGAAGGGATTGATGGAAAGTTGCTGGACGTCTGAGCCAACAGAGAGACCATCCTTTACTGAAATATCACAG

AAGCTCAGGACAATGGCTGCTGCTATGAATTTGAAATAA

>AtRAF36 (AT5G58950)

ATGGATGAAGAGGCTACTTCGTGGATTAGGAGGGCTAAGTTTTCTCAAACGGTTTCTTATCGTTTGAATTCTTCAAAGTTAGC

TTCTCTTCCTTTTATGATAAACCAAGATAAGTTTTCTGGATTAAAAGCTATACCTCAGAGATCATCTTCTTCGTCGTCGGCATC

ATCATCGGATCCTAAGTTGGTTTCGAGTAACTCACAGACTACGGGAGATACTTCGAGTTTGGAAGCTGCTGATGTATATGTTG

TTGATTCGGAAATACAGACAAACCCGGTTACTCATAAGCAGAGATCTGTTTCTCCTTCGCCTCAGATGGCTGTTCCCGATGTG

TTTAAGGAAGCGAGGTCTGAGCGTAAGAGATTTTCTACTCCACATCCAAGAAGAGTGGAATCGGAAAAGGGAATGAAGCCT

AAGTTATCTCATAAAAACTCCTTTGACAAGAGATCATTTAACTTGCGGTCTCCTTCAGGTCCTATCAGAGATCTTGGCACTTT

GAGAATTCAAGAGAGGGTCAAGAGCAAGAAGGACACTGGATGGTCTAAGCTTTTTGATAATACTGGTCGTAGAGTGAGTGC

TGTGGAAGCTTCTGAAGAGTTCCGTGTTGATATGTCAAAGCTGTTCTTTGGGCTTAAGTTTGCTCATGGTTTATACAGCCGGC

TATATCATGGTAAGTATGAAGATAAAGCTGTTGCCGTGAAGCTTATCACTGTACCTGATGATGACGATAATGGATGCTTGGGA

GCTCGTTTAGAGAAACAGTTTACCAAGGAAGTGACCCTTTTGTCTCGATTGACCCATCCAAATGTTATAAAGTTTGTTGGAG

CGTACAAAGATCCGCCTGTATATTGTGTCCTCACGCAGTATTTACCTGAAGGATCTTTAAGATCTTTTCTGCACAAGCCTGAG

AATAGGTCTCTTCCTTTGAAAAAGCTTATAGAGTTTGCTATAGATATTGCTAGAGGGATGGAATATATTCACTCACGACGCATA

ATTCATCGGGATCTTAAGCCAGAAAATGTATTGATCGACGAAGAATTCCATTTGAAGATCGCTGACTTTGGCATAGCGTGCGA

GGAGGAGTACTGTGACATGTTGGCTGATGACCCCGGAACATATAGGTGGATGGCACCTGAAATGATAAAACGGAAACCACA

TGGACGTAAGGCCGATGTATACAGCTTTGGACTCGTTTTATGGGAAATGGTAGCTGGAGCAATCCCATATGAGGACATGAATC

CTATTCAAGCTGCTTTCGCAGTCGTACATAAGAACATTAGGCCAGCTATCCCGGGAGATTGTCCAGTAGCCATGAAAGCTCTG
ATAGAGCAGTGTTGGTCGGTTGCACCAGATAAGAGACCCGAGTTCTGGCAGATAGTGAAAGTGCTTGAACAGTTTGCGATAT
CACTGGAACGTGAAGGGAACCTGAATCTGTCTTCGAGCAAGATCTGTAAGGACCCAAGGAAAGGTCTGAAACACTGGATC
CAGAAGCTTGGACCAGTCCACGCAGGAGGAGGCGGTGGCAGCAGCAGCAGCGGCCTTGGTGGCTCAGCCTTGCCGAAGCC
TAAATTCGCTTGA
>AtRAF37 (AT5G66710)
ATGAGACCTCGCGGGTATCAACGAGCTCCGTCAATGCAGAAACCAACAGATTATCCGACGGATAAGACGCTTCATCCGAATT
ACCCATTTCTCATGTCCTCTCATGGTCTCAAGTCCTTTGAATCGGACGACGAAGACGACGACTCCGACTCCTCCAACGATCA
ATTCGCTTTCACTATCAACACAGAGTTGCTTGTCGATGTCAAAGACATATCTATAGGGGACTTCATCGGCGAAGGTTCATCAT
CTACTGTCTACAGAGGATTGTTCAGGAGAGTTGTCCCTGTGTCTGTGAAGATATTCCAGCCTAAAAGAACATCTGCTTTAAG
CATTGAGCAGAGGAAGAAGTTTCAGAGAGAGGTTTTGCTGCTTTCCAAGTTTAGACATGAAAACATTGTGCGGTTCATTGG
GGCGTGCATAGAACCAAAGTTGATGATAATTACTGAACTCATGGAAGGCAATACTCTTCAGAAGTTTATGTTGAGTGTTCGTC
CAAAGCCTCTTGATCTGAAGCTCTCCATTAGCTTTGCGTTGGATATTGCTCGGGGAATGGAGTTCTTGAATGCAAATGGCATT
ATTCACCGAGATTTGAAACCAAGTAATATGCTCTTAACAGGTGATCAGAAACATGTAAAGTTGGCTGACTTTGGACTTGCTA
GAGAGGAGACTAAAGGTTTCATGACCTTTGAGGCTGGTACATACAGATGGATGGCTCCAGAGTTATTCAGCTATGACACGCT
TGAAATTGGCGAAAAGAAACACTACGATCATAAGGTGGACGTTTACAGTTTCGCCATTGTTTTCTGGGAGTTACTTACCAAC
AAAACCCCATTCAAAGGAAAGAACAACATCTTTGTAGCTTATGCTGCCAGCAAAAACCAGAGACCAAGTGTGGAGAATCTT
CCAGAAGGCGTTGTTTCTATCCTGCAATCATGCTGGGCAGAGAATCCTGACGCTCGTCCTGAGTTTAAAGAAATTACATATTC
ACTAACAAACTTGCTCAGAAGCTTAAGCTCAGACACTGATGCTACTTCATCCAATAGCAAAGCCAATATAGCTACCGAGGAT
TCAACAAGCAGTCTGGTTCAAGAACGTGTTGTCTGTGATTGTCCCGGACTAAAGATGAGCAAGACGAAGAAGCTGAAGAA
GAAGACGAATAAACTGATGAATATGATCGTCCCTTTTCTCAAGATCTTCAAAAGTTGTATGTCCAAGTGA
>AtRAF38 (AT3G01490)
ATGAAGGAGAAGGCGGAGAGTGGTGGAGGAGTAGGATACGTGAGAGCAGATCAGATAGATTTAAAGAGTCTGGACGAGCA
ATTGCAGAGACACTTAAGTAAAGCATGGACGATGGAGAAGAGGAAGAGTTTGAGTGATGGTGAAGATAACGTCAATAACAC
CCGACATAACCAGAACAACTTCGGACATCGACAGCTTGTGTTTCAGAGGCCGCTTCTTGGTGGTGGATATAGCAACAACAAC
AACAGCAGCAAGAACGACATAATTAGGTCGACCGAGGTTGAGAAGTCGAGGAGAGAGTGGGAGATTGATCCTTCTAAGCTT
ATAATCAAAAGTGTGATTGCTAGAGGTACTTTTGGTACGGTTCACCGTGGAATCTACGATGGTCAAGATGTCGCCGTAAAACT
ACTAGACTGGGGAGAAGAGGGGCACAGGTCAGACGCAGAGATAGCTTCGCTTAGAGCTGCTTTCACTCAAGAAGTTGCTGT
GTGGCATAAGCTCGACCATCCCAATGTCACCAAGTTCATAGGAGCGGCGATGGGGACATCGGAGATGAGCATACAAACGGA
AAACGGGCAAATGGGAATGCCGAGTAACGTTTGCTGTGTCGTGGTTGAGTATTGCCCCGGCGGCGCTCTCAAGTCATTCCTC
ATCAAAACTCGCCGCCGCAAACTCGCCTTCAAAGTCGTTATCCAACTCTCCCTTGATCTTGCTCGCGGGTTGAGTTACTTGC
ACTCTCAGAAGATTGTGCATAGAGACGTGAAAACGGAGAACATGCTTTTAGATAAGTCACGCACCTTAAAGATTGCAGATTT
TGGTGTGGCTCGTCTAGAGGCTTCAAACCCTAACGACATGACCGGTGAGACCGGAACCTTGGGCTATATGGCTCCTGAGGTG
CTAAACGGGAGTCCCTACAACAGAAAGTGCGATGTGTATAGCTTTGGGATATGTCTTTGGGAGATTTACTGTTGCGACATGCC
TTATCCTGACCTCAGCTTCTCCGAAGTAACCTCCGCCGTCGTCCGCCAGAATTTGAGACCAGAGATACCTAGATGTTGTCCA
AGCTCGCTGGCGAACGTGATGAAGAGGTGTTGGGATGCGAATCCGGAGAAGCGGCCGGAGATGGAGGAAGTTGTGGCTAT
GTTGGAGGCTATTGATACTTCCAAAGGCGGAGGAATGATTCCTCCTGACCAACAACAAGGCTGTTTCTGTTTCCGCCGACAC
CGAGGCCCATGA
>AtRAF39 (AT3G22750)
ATGGAAACAAGAAACGAGACAAAAGCTTCACCGGAAAACAACCTCAGAAACAGAGGAGCTGATGGAAACAATAGCAAGA
AAGACATGATTTTCCGAGCTGATAAGATTGATTTGAAGAATTTGGATATCCAGCTAGAGAAACATCTGAGTAGGGTTTGGTCA
AGAAGCATTGAGAAGCACCCTAAGCCTAAGGAGGAATGGGAAATTGAATTGGCTAAATTAGAGATGAGGAATGTCATTGCT
CGTGGTGCTTATGGTATTGTCTACAAAGGCATATATGATGGTCAAGATGTTGCTGTGAAAGTACTTGATTGGGGAGAAGATGG
TTACGCGACAACGGCTGAGACATCTGCTCTACGTGCTTCATTTCGTCAAGAAGTTGCGGTTTGGCATAAGCTTGACCATCCTA

ATGTCACAAGGTTTGTGGGAGCATCAATGGGAACAGCGAATTTGAAGATACCTTCATCAGCTGAGACGGAGAACTCGTTGC
CTCAACGAGCTTGTTGTGTCGTTGTGGAGTATATCCCTGGAGGAACTCTTAAGCAGTATTTGTTCCGAAACAGGCGAAAGAA
ACTCGCTTTTAAAGTCGTGGTTCAACTCGCTCTCGATCTCTCCCGAGGGTTAAGTTATTTGCATTCAGAGAGGATTGTTCATC
GCGATGTGAAAACAGAGAATATGCTTTTGGATTATCAGAGGAATCTAAAGATTGCTGATTTTGGGGTTGCTAGAGTTGAAGC
TCAGAATCCAAAAGACATGACTGGAGAAACTGGAACTCTTGGATACATGGCTCCAGAGGTTCTTGATGGCAAGCCATACAA
CAGAAGATGTGATGTTTACAGCTTTGGGATATGCTTGTGGGAGATTTATTGTTGTGATATGCCTTATCCTGATCTCAGCTTTGC
TGATGTTTCTTCTGCTGTTGTTCGTCAGAATCTGAGACCGGATATTCCAAGATGTTGTCCAACAGCATTGGCGACCATAATGA
AGAGATGTTGGGAAGCTAATCCGGAGAAACGACCAGAGATGGAGGAAGTTGTGAGTTTGCTTGAAGCTGTCGACACCACC
AAAGGCGGTGGAATGATCCCGGAAGATCAGAGACCTGGCTGTTTCTGCTTTGTCTCTGGTCGTGGTCCCTAA
>AtRAF40 (AT3G24720)
ATGGAAGCCAGTGTTTATGGCTTGCAGATAATAAAGAACGCTGATCTTGAAGACCTGACAGAACTGGGATCCGGGACATATG
GAACTGTCTATCATGGAACATGGCGGGGAACGGATGTTGCTATAAAGAGAATACGGAACAGTTGCTTTGCAGGGAGATCATC
TGAACAAGAACGCTTGACGAAAGATTTCTGGAGGGAAGCACAAATACTCTCAAATCTTCATCACCCAAACGTTGTTGCATTC
TACGGTATAGTGCCTGATGGAACTGGAGGAACCTTGGCAACTGTTACAGAGTTCATGGTGAATGGGTCACTCAGGCATGCTC
TTCTCAAAAAGGACAGATTGCTTGACACTCGGAAAAAGATAATAATTGCAATGGATGCTGCTTTTGGCATGGAGTATTTGCA
CTCGAAGAACATCGTCCACTTTGATCTCAAATGCGAAAACTTACTTGTCAATTTAAGGGATCCACAACGGCCTATATGCAAG
GTGGGAGATCTTGGCTTATCTAGAATCAAACGTAACACTTTAGTATCTGGGGGAGTGAGAGGAACTCTTCCATGGATGGCCC
CAGAACTCTTGAACGGAAGCAGCACCCGAGTTTCTGAGAAAGTTGATGTTTTTTCATATGGAATCTCGCTGTGGGAGATTCT
AACCGGAGAGGAACCCTATGCAGATATGCATTGTGGAGCAATCATTGGCGGGATCGTGAAGAACACACTTCGACCTCCTATA
CCAAAAAAGCTGTTCTCCGGAATGGAAAAAGCTGATGGAACAATGCTGGTCTGTCGACCCGGATTCTCGTCCTCCATTCACCG
AAATAACATGCCGACTTCGGTCCATGTCAATGGAAGTCGTCACTAAAAGCAAAAGAAGAGAAAATAAACCCTGA
>AtRAF41 (AT3G27560)
ATGGGATCTGCAAGTGGGTTTTACTCGAACGAAGAGTTTGAATTGGATCCTAAATGGCTTGTTGATCCTCGTCATCTCTTTGT
TGGTCCTAAGATCGGTGAAGGTGCTCATGCCAAAGTTTATGAGGGAAAGTATAGAAACCAAACAGTGGCGATTAAGATTATT
AAAAGAGGAGAGTCCCCTGAAGAGATTGCTAAACGAGATAACCGGTTTGCGAGAGAGAGATTGCTATGTTGTCTAAAGTCCAG
CACAAGAATTTAGTCAAGTTCATTGGAGCGTGCAAAGAACCCATGATGGTTATAGTTACTGAGCTTCTACTAGGCGGTACATT
GCGTAAATATCTAGTCAGCTTACGGCCAAAGCGTTTAGACATTCGTTTGGCTGTTGGTTTTGCTCTTGACATTGCTCGTGCAA
TGGAATGTTTACATTCCCACGGTATCATTCACCGCGATCTTAAACCAGAGAATTTGATCTTATCAGCGGATCATAAGACAGTG
AAACTCGCTGATTTCGGTTTAGCTAGAGAAGAATCATTAACCGAGATGATGACCGCAGAGACTGGTACATACCGATGGATGG
CCCCCGAGCTCTACAGTACGGTTACGTTAAGGCAAGGAGAGAAGAAACACTATAATCATAAAGTAGATGCTTACAGCTTTGC
CATTGTCTTGTGGGAACTTATTCTCAACAAGTTACCATTTGAAGGCATGTCAAATCTACAAGCAGCATATGCTGCTGCATTCA
AGAACTTGAGGCCAAGTGCAGAGGATTTACCAGGAGACTTAGAGATGATTGTAACTTCATGCTGGAAAGAAGATCCTAACG
AACGGCCAAATTTCACAGAGATAATTCAAATGCTCCTCCGTTACCTCACCACTGTTTCGGCACCACAGATCATTCCTCCACCG
AATCGACGAGTCTTCTCATCAGAAAACATAGTTTTATCACCGGAATCTCCCGGAACTTGCTCTCTGATGTCTGTCAGAGACG
GAGACGTTTCTCGTCAGACCGTTAACACTGCTGATTCATCGGAGAAACAAACAAAAGGAAGCTTCTTCTCCTGCTGCTCATA
G
>AtRAF42 (AT3G46920)
ATGGCACATGAACCCAGTAGCCCAAGTTCGAATCTTGTTTCTAATCCGGCCAATCTTTCAGCTTCTGGGTTAGATTATAGCTCT
GATTTGAGTAATAAGCGTGTCTCTGATGGAATTATTTCTGGGTTTGGGAGTGAGCAGGTAAGTATTGATGCTACGAATAGAAA
CAATCCAAATTTAGGCAATAAAAGAAGTGATATGGATGATGAAGAATTGGAAAAGGTGAAGTTTTTATGTAGCTACAATGGA
AAGATAATCCCTAGACCAAGTGATGGCATGTTACGTTACGTTGGTGGTCAAACAAGGATCGTTAGTGTTAAAAAGAATGTGA
GATTTGATGAATTTGAGCAAAAGATGATTCAGGTTTATGGTCACCCTGTTGTTGTTAAGTATCAGCTTCCTGATGAGGATCTTG
ATGCTTTGGTTTCTGTTTCATCTAGTGAAGATATTGATAATATGATGGAGGAGTTTGAGAAGTTGGTAGAGAGATCTTCTGATG
GGTCAGGGAAGTTGAGAGTTTTTTTGTTTGATGCTTCTTCGTCTGAGGTGGATGATTCTTTCGGAATATTGGAGTATGGTGAT

GGAGTTGATATTGGGCAGAGATATGTAGAGGCTGTGAATGGTGTTGTTGTGAGTAAGGAAAGTGTAGCTAGTGGTAGTTCTA
ATCCGAATTCTGATTTTAGTGGCGTGGATGTTGTTGATAGCTTGGGCGTTGGTCAGAGTGATTTTGTCGCAACTACTTGGACC
TCGAGTAATTTTTCACCTCAGACTTATCATAGTAATGTATCAAGACTTGTTCCTCCTGATCCACGCTCTTCTGCTTATGTTGTGC
CAATGACTGTCCACGCCGATCCGCCTCATTCTTTTCAGCTTGAGACTGTTTCAGAGAAACCAATAGTTGGTAAGATGCAGCA
GCAACAACAGGGGTATACTACACCATCTGAACATCATCCACCGGCCTATGTTGAATCTCGCCAAGAAGCTTTAAGACAACCA
GATATTGTTCATTCACCTATCCAGCTTTTGCCAAGTAGTACTTCTTTGTTCTCCCAACAACCTTTTCAAGATAGTCCTTTAAGT
GTCTCATCCCACCAGTTCCTTCCTGCTGCTCACATGTCAATGGCTCCCTTGAATTCTCAGATTAGTTCCACACCAGTTTTGATT
AACCCGGTAATGCAAACCCAAGAAAATCTGTTGGGGAACTATCATGCTGCTCAAAAGTTAGTACCGCTTCCTACAGAGCCAA
GAAACACTGCATATCAAGGTACGATTTCTCCTGGTATACCATTTGATGGTTATGGCGGGTCTCAAGTTCCACCATCTAACCATG
TTGTTCTCCCCGATGGAAGTTTCTATCAACAAGTAACTATGGCTGAAAGTTTTCAAAGGGTAAATGATTGTCATATGTGCCAG
ACATCTTTTCCTCATATGCATTCTGATCCGATTATGCGGGAGGGAAATGATGGTTCTACAATGTATGTGCCATATGTGAGCTCA
GCTTTCTATGCTTCTCGTCCAGATGATATCATGAGAATCCAACAAACGGATAAGTTTACGGGTCAACAAAGCTTCCTCAATCA
TTCAAATCATCAAGAAAGAGATACTTTACATAACGCCAATCTTGCCACTGCTCAGGTCGAGACTACTGAACCTTTTGTCAATG
AGATAGTAAGAGATGTTCCGATAAAAGTTCAAGTAACTAGGCAGCAGCAACATCCCGTAGACCCTTCAGTAGCATATGCACA
ATGCCGAGAACTAAGTGGATTGGTGGATAATGTTAACATACATGCTCCTGAAATATATAGCAATTGTCAGAATTTCATCTCACC
AGTTGACAAAATTGGGAAAGAAGATATTATGGGTACCAGTTCTCAACAAATGGCAAGGAAGAATATGTTCTTACATGACACC
TCTGGTCAGTCTCCAGTAAGTCCAAACATAGATCACACTGATTCAGCTAAGAGGCTAACAAGAGTAGTTCTTCCAGGCCATG
AATCTCAGCCTAAGGAATCATGTGTGCCAACACAGTCTCCCTTATTAGGCAATCCTGGTTTGTATCTACAATCATTGGTTGGA
GGTCAGCAGTTCGATTCAGCGGAAGCGCAGTCCAGCAATCCCGCTTATGACGTTGTTGAATCTACATTTGATGCTGCAAACTT
ACCATCATCCTTATCATCAAATCCTGATGCTGCTAATTTACCATCATCCTTGTCATCTAGTGTTGGTGGTGCAGACCACAAAGA
ATCATCAAAATCACTTTTTAGTAACCAGGACCCATGGAATTTGCAAACTAACTCAAACGAGGATGTCAAACCTGATCTACTTA
ATTCTTCGAAAGTTATCCTTGAGAATGATCTGTTGATTGGTCTTTGGTTCTCTCTGAAAGGCTCCGAGGAGGAGCACATCAAA
CAAGAACTTCAGAATGTTGCTGAAGGAGTCGCCGCTTCTGTGTTACAGTCAAGCACACCTTCATATCATGAACCTCCAATTA
AGGTGGATGAGTATGCTTTTAATTCGAAAGGAGAAGTTTCACGTAATGATGAAATGAAACAGCAGTCTACACACTTCAAGGA
TATCAGGAACCAACTTCTAGAAAGACTGAATTTCGGATATTCTGGTTCTGATTCGCTGGATCAGTTGCAGATTATAAAGGACA
GCGACCTTGAGGAATTGCGAGAACTAGGATCTGGCACATTTGGCACAGTTTATCATGGGAAATGGCGAGGCACTGATGTTGC
AATCAAAAGGATCAATGATCGATGTTTTGCTGGCAAACCATCTGAGCAAGAACGAATGATCGATGACTTTTGGAATGAGGCA
CAGAACCTAGCTGGCTTGCACCATCCTAACGTCGTTGCTTTTTATGGGGTTGTGCTGGATTCACCTGGAGGTTCTGTGGCAAC
AGTTACAGAGTATATGGTCAATGGTTCTTTACGAAATGCGTTGCAGAAGAATGTTAGGAATTTTGATAGGTGCAAGCGTCAAC
TGATCGCTATGGACATTGCTTTTGGGATGGAGTATTTGCATGGAAAGAAGATAGTACACTTTGATCTTAAAAGTGACAACCTG
CTCGTCAATCTCAGGGATCCTCATCGCCCAATTTGCAAGGTTGGGGATTTGGGTCTCTCAAAGGTGAAATGTCAGACATTAAT
ATCAGGTGGTGTTCGGGGAACACTTCCATGGATGGCACCCGAGCTTCTAAATGGAACCAGTAGCCTTGTTTCTGAGAAGGTC
GATGTGTTTTCATTCGGAATTGTCCTCTGGGAACTTTTCACCGGTGAAGAACCTTACGCCGACTTGCACTATGGAGCGATCAT
AGGTGGTATAGTTAGTAATACGCTACGGCCGCAGATTCCCGACTTTTGTGACATGGACTGGAAATTGCTGATGGAGAGATGTT
GGTCTGCAGAACCATCAGAGAGGCCAAGCTTCACTGAGATCGTGAACGAACTACGGACCATGGCTACAAAGCTTCCTTCCA
AAGAACAAGGCTCAACACAGGGACCACAATCTTAA

>AtRAF43 (AT3G46930)

ATGGATGGAGAGGTTACTTCTTGGATTAGGAGAGCAAATTTCTCTCATACTGTTTGTTATCGTATGATTACGCCGAGTTTAGAA
TCTATGCCTTTTACTGTAAACCAAGAGAAGATGCAGAGGAATCCAGTTACTAATAAGAAGAGATCTGTTTCTCCGTTGCCTCA
TATGGCTCTTTCTGATGCTTTTATCGAAGCAAAGTCTGATATTAAGAGGTTTTCTACTCCACATCCTAGAAGAGTGGAACCAG
AGAAGGGAATGAAGGCCAAGTCGTCGTCCCGCAAAGATTCTTCTGAGAAAAAATCAGTTAATCTACGGTCCCTGTCTCACT
CGGGTCCTATTAGGGATCTTAGTACGCAGAAAGTTAAAGAGAGGGGAAAGAGCAAGATAGATAAGAAGTCTTCAAAGTCTG
TTGACTATAGAGGTTCTAAAGTAAGTTCTGCAGGAGTGCTTGAAGAATGCCTCATTGATGTGTCTAAGTTGTCTTATGGGGAT
AGGTTTGCTCACGGGAAATATAGCCAGATTTATCATGGTGAATATGAAGGCAAAGCTGTTGCTCTGAAGATTATCACAGCGCC

TGAGGATAGTGACGACATATTCTTGGGAGCTCGTTTAGAAAAAGAGTTTATCGTGGAAGCCACTCTTTTATCTCGACTAAGCC
ATCCAAATGTCGTTAAGTTTGTTGGAGTGAATACTGGAAACTGTATCATCACAGAGTATGTACCTCGAGGGTCTTTAAGATCA
TATCTGCACAAGCTCGAGCAGAAATCCCTTCCTTTGGAACAGCTAATCGATTTTGGTCTGGATATTGCTAAAGGAATGGAATA
TATTCACTCAAGAGAGATAGTTCATCAGGATCTGAAGCCAGAAAACGTGTTGATCGACAATGACTTTCACTTGAAGATTGCT
GACTTTGGCATAGCGTGCGAGGAGGAGTACTGTGATGTTTTGGGGGATAACATAGGAACTTATAGGTGGATGGCACCTGAAG
TTTTAAAACGGATACCACATGGACGGAAGTGCGATGTTTATAGTTTTGGACTTCTTTTATGGGAAATGGTAGCTGGAGCACTT
CCATATGAGGAGATGAAATTTGCTGAACAAATTGCCTACGCAGTTATATACAAGAAAATTAGGCCAGTTATACCGACGGATTG
TCCAGCGGCCATGAAAGAGCTGATCGAGCGATGTTGGTCATCGCAAACAGACAAGAGACCGGAATTCTGGCAGATTGTCAA
AGTGTTGGAACATTTCAAGAAGTCTCTAACAAGCGAAGGAAAACTTAATCTTTTACCTAGCCAGATCTGTCCAGAACTAAAG
AAATGTCCTAAATTCTGGATTCATATATTCGGATCATTCCACCACCACAGCAGCGGTGGCGGCAGCAGCAGCAACAACTCAG
CCTTACCTAAGCCTAAATTCGCTTGA

>AtRAF44 (AT3G50720)

ATGGCCATTTCTCCGACGATGATGCTTAATGCGAACTACCCTTTTTTCATGTCAGCGTTTGGCTCAGATGACAATAATGATGAG
TCTGATAACCAGTTCGATTTCAATATCAGCAGAGAGTTGCTTCTCAACCCTAAAGACATCATGAGGGGAGAGATGATTGGAG
AAGGAGGCAACTCCATCGTTTACAAAGGCCGACTCAAAAACATTGTTCCTGTGGCGGTGAAGATAGTGCAGCCGGGTAAAA
CATCTGCTGTAAGCATTCAGGACAAACAACAGTTTCAAAAGGAGGTTCTGGTACTATCCTCGATGAAACATGAAAACATTGT
CAGGTTTGTTGGAGCTTGCATAGAGCCACAATTGATGATAGTTACCGAACTCGTAAGAGGTGGTACTCTTCAGAGGTTCATG
TTGAACTCTCGCCCGAGTCCTCTTGATCTCAAGGTGTCACTAAGCTTTGCTTTGGACATTTCTCGAGCCATGGAGTATTTGCA
CTCAAAAGGCATCATTCACCGTGACCTAAATCCAAGGAATGTTTTGGTAACTGGTGATATGAAACATGTGAAGTTGGCTGATT
TTGGACTTGCAAGAGAAAAGACTCTTGGTGGCATGACTTGTGAGGCGGGCACGTACCGTTGGATGGCTCCAGAGGTATGCA
GCCGTGAGCCACTCCGAATTGGGGAGAAAAAACACTACGACCAGAAAATCGATGTGTATAGCTTCGCACTGATTTTCTGGTC
CTTGCTCACAAACAAAACACCTTTCAGTGAGATACCTAGCATTTCTATTCCCTATTTTGTGAACCAGGGCAAGAGACCAAGC
CTATCGAATATTCCTGATGAGGTCGTCCCCATATTAGAATGTTGTTGGGCAGCGGACTCAAAGACTCGTCTCGAATTCAAAGA
TATTACAATCTCACTGGAAAGCTTGCTAAAAAGATTCTGCTCAGAGAGAAGTAATAATGAGATAACGATCACCGAAGATGAA
GCTTATGATGATGAGATAGAGGAATTGGAGACCACTTGGCTGCTTCCAAAGCGCTATATCAAGCTGAAGAAACCTAAGAAGA
TCAAGCAGAACGTGATGAAGAAGATACTTCCTTTCTTCAAGAAGTTCATTTCTTCAAAGTGGTGA

>AtRAF45 (AT3G50730)

ATGATATCTCGAATGATTTTCAGAAACTATCCTTCTCACAATGAGTCCGACGACGAGCCATTCCATTTCAGTATCAGCAGAGA
ATTGCTTCTCGACCGAAACGATGTCGTAGTGGGAGAGATGATTGGAGAAGGAGCTTACTCCATCGTCTACAAAGGATTGCTA
AGAAACCAATTTCCCGTGGCGGTGAAGATCATGGATCCGAGTACAACTTCCGCTGTAACCAAAGCGCACAAAAAAACGTTT
CAAAAGGAAGTTCTGTTACTATCCAAGATGAAACATGACAACATTGTGAAGTTTGTTGGAGCTTGCATAGAACCACAATTGA
TTATAGTTACCGAACTCGTTGAAGGCGGCACTCTACAGAGGTTCATGCACTCTCGTCCGGGTCCTCTTGACCTAAAGATGTC
ACTAAGCTTTGCTTTGGATATTTCACGAGCCATGGAGTTTGTGCACTCAAATGGCATCATTCACCGTGATTTAAATCCAAGGA
ATTTGTTGGTAACCGGTGATCTGAAACATGTGAAATTGGCTGACTTTGGAATTGCAAGAGAAGAAACTAGAGGTGGCATGA
CCTGTGAGGCTGGCACCTCTAAATGGATGGCTCCTGAGGTAGTGTATAGCCCTGAGCCGCTGAGAGTTGGGGAGAAGAAGG
AATATGACCATAAGGCTGATATTTACAGCTTTGCCATAGTATTATGGCAGCTGGTTACGAACGAAGAACCATTCCCTGACGTG
CCTAATAGCCTCTTTGTTCCCTATCTTGTTAGCCAAGGTCGGAGACCAATACTTACGAAGACTCCCGACGTGTTTGTCCCAAT
CGTTGAATCATGTTGGGCACAAGACCCGGATGCGCGTCCGGAATTTAAAGAGATTTCGGTTATGCTGACAAACTTGCTAAGA
AGAATGAGCTCAGACAGTAGCATTGGCACAACACTACCGGATGGGGAAGCTTATGAAGGTGAGATGGAGGAATCAGAGAA
CTCTCCTCTTCTTCAAGAGCATTTTTGCAAGGTGAAGAAACCTAAGGAGAAGAAGAAGAAGAAGAAACTGGTGAAGATGA
GATTTCCTTTCTTTAAGAAGTTCAAGGTTTGGTTGTACAACTACAAGCCATGA

>AtRAF46 (AT3G59830)

ATGGACAACATTGCCGCGCAGTTGAAGCGTGGGATATCGAGACAATTCTCGACAGGATCAATGAGACGAACATTAAGCCGA
CAATTCACGCGCCAAAATTCTCTCGATCCACGGCGTAACAACATGAGATTCAGCTTCGGTCGTCAATCTTCGCTTGATCCGAT

CAGACGTAGTCCTGAGTCTCTTAGCTGCGAGCCGCATATGTCTGTCCCCGAGAATCTTGATTCGACGATGCAGCTTCTCTTTA

TGGCGAGTAAAGGTGATGTTAATGGCGTTGAGGAGCTGCTTAATGAAGGAATAGATGTGAATAGCATTGATCTTGATGGTCGT

ACTGCTCTTCACATTGCTTCCTGTGAAGGTCATTATGATGTTGTTAAGGTTCTTCTTAGCCGGAGAGCTAATATCGATGCTCGT

GATCGTTGGGGTAGTACGGCGGCTGTTGATGCAAAGTATTATGGGAATGTTGAAGTTTATAATCTCTTGAAAGCTCGAGGCGC

TAAAGCTCCGAAAACTAGAAAGACTCCAATGACGGTAGGGAATCCAAAAGAAGTTCCTGAGTATGAGCTTAATCCACTCGA

GCTGCAAGTCCGAAAAGTTGATGGCATTTCAAAGGGAACTTATCAAGTTGCTAAATGGAATGGCACGCGAGTCTCAGTAAA

AATATTCGATAAAGATAGCTATTCAGATCCTGAACGAGTAAATGCTTTCACAAACGAATTGACTTTGCTAGCAAAAGCTCGGC

ATCCAAATATTGTTCAATTTGTTGGAGCCGTCACTCAAAATCTACCGATGATGATTGTAGTTGAGTGTAATCCAAAAGGTGAT

CTAAGTGTCTATCTTCAAAAGAAAGGTCGTCTTTCTCCTTCAAAAGCTCTGAGATTTGCTCTTGATATTGCTAGAGGAATGAA

CTACCTCCATGAATGTAAACCAGATCCGATCATCCACTGCGAGTTAATGCCAAAAAATATTTTGCTAGATAGAGGAGGGCAAT

TGAAGATCTCTGGTTTTGGTTTGATAAAGTTGTCAAAGATTGGAGAAGACAGTGCAAAAGTAGTGAACCACGAAGCTCAAA

TCGATAAGTCAAATTACTATATAGCACCCGAAATTTACAAAGATGAAGTCTTCGATAAAAGGGCTGATGTGCATTCGTTTGGT

GTCATTTTGTACGAGTTGACCGAGGGAGTATCTTTATTCCATCCTAAACCACCTGAAGAGGTTGCAGAATCGATTTGTATAGA

AGGTAAAAGACCAACAATCAGAACAAAGTCAAAGAGTTACCCTCCGGAATTGAAAGAGTTGATTGAGGAATGCTGGCATCC

AGAAATCAGTGTGAGGCCAATATTCTCTGAGATTATTATTCGGCTCGACAAGATAGTTACAAACTGTTCCAAGCAGGGTTGGT

GGAAAGACACATTTAAGTTCCCTTGGAAATGA

>AtRAF47 (AT3G58760)

ATGACGATTAAGCCTAAATCGCCGGCGAGATTCAAGCTAGGTAGGCAGTCTTCGCTTGCGCCGGAATCAAGAACCCCGATTG

ATACGTTAACGGAAGATGAGGACGACGACTTGGCTGCGGCGGCTACAGCTGGGATCGGGGATCCGACGATTCGTTTAATGTA

TTTAGCCAACGAAGGTGATATCGATGGCATCAATAAGATGCTTGATTCGGGTACTAATGTTGATTACCGTGACATCGATGCCC

GTACTGCTCTTCACGTCGCCGCTTGTCAAGGACGAACCGATGTCGTTGAGCTGCTGCTTAGCCGTGGTGCTAAGGTTGATAC

GAAGGACCGATGGGGTAGTACGCCTCTTGCAGATGCAGTGTATTACAAAAATCATGATGTGATTAAACTTTTGGAGAAACAT

GGTGCTAAGCCTACGATTGCTCCTATGCATGTCCTAACTGATAAAGAAGTTCCAGAGTACGAGATTCATCCTACAGAGCTTGA

TTTTTCCAACTCTGTCAAAATTTCAAAGGGTACCTTTAACAAGGCTTCATGGCGTGGAATTGACGTGGCTGTTAAAACCTTTG

GAGAGGAAATGTTCACTGATGAAGACAAAGTGAATGCATTTAGGGACGAACTTGCACTGCTTCAAAAGATACGCCATCCGA

ATGTTGTTCAGTTTCTAGGGGCAGTTACTCAGAGTACCCCCATGATGATTGTCACCGAGTATTTACCAAAGGGAGATCTTCGA

CAATATCTAGACAGGAAAGGACCTCTAATGCCAGCACACGCAGTGAAGTTCGCCCTTGAAATTGCTAGGGGAATGAATTATT

TGCACGAACATAAACCTGAAGCAATAATCCATTGCGACCTAGAACCTCCAAACATACTGCGGGATGATTCGGGACATCTGAA

AGTTGCAGACTTTGGAGTCAGCAAGCTGCTGGTAGTTAAGAAGACAGTTAAAAAAGACAGACCTGTTGTTACATGCTTGGA

CAGTTCATGGCGATATATGGCTCCAGAGGTTTATAGGAATGAAGAGTATGATACAAAAGTAGATGTATTCTCTTTCGCTTTAAT

CTTACAAGAGATGATAGAAGGTTGTGAACCATTTCATGAGATAGAAGACCGCGAAGTTCCTAAAGCATATATTGAAGATGAA

CGTCCACCATTCAATGCTCCAACAAAATCATATCCTTTCGGGTTACAAGAGCTAATCCAGGATTGTTGGGACAAAGAAGCAT

CAAAAAGACCAACATTTAGAGTAATCATCTCTACTTTGGAGTTGATAAGTGATCGAATTGCACGCAAAAGGAGCTGGAAGGT

GATGCTAGGAAGATGCCTTCCAAGATTCAGATTGTTTACAAAGCGAGATTATGTGAATCCCGGTGGTAGTAACCGCTCATCAG

GCTCATTCAACAGATGA

>AtRAF48 (AT3G63260)

ATGGCCTCTGGCGGCGGAGAGGCGGATAAATCACTTGAAATCGGGTCCGGGACCGCGGATCCCAAAATAGGCGGTACTGGG

AGCAGGAGCGCCGGAGAAGAACGATACTTCAGGGCAGATACACTGGATTTCAGTAAATGGGATTTGCATATGGGTCAAACC

TCTACTAGCAGCGTCCTCACCAATTCCGCTTCCACGAGCGCTCCCGCACCGGCGATGCAGGAATGGGAGATTGACCTCTCCA

AACTCGATATGAAGCACGTCCTCGCTCACGGTACTTACGGCACTGTCTACCGCGGTGTCTACGCCGGCCAAGAAGTCGCAGT

GAAAGTGTTAGATTGGGGAGAAGATGGTTACGCCACACCAGCTGAAACTACAGCTCTCCGTGCTTCCTTCGAGCAAGAGGT

CGCCGTCTGGCAGAAGCTCGATCATCCCAACGTTACCAAGTTTATAGGAGCATCCATGGGAACCTCTGATCTGCGGATCCCT

CCTGCTGGTGATACTGGCGGACGTGGTAACGGTGCACATCCTGCGAGGGCCTGTTGTGTTGTGGTTGAATATGTTGCCGGAG

GCACGCTTAAGAAGTTCCTCATCAAGAAATATAGGGCCAAACTACCCATCAAGGATGTCATTCAGCTCGCTTTGGATCTCGCT

AGAGGGCTTAGTTACCTCCACTCCAAGGCGATTGTACATAGGGACGTGAAGTCAGAGAACATGCTGTTACAGCCTAACAAG
ACGCTGAAGATCGCTGATTTCGGGGTAGCTAGAGTTGAAGCTCAGAACCCTCAAGACATGACGGGTGAAACTGGAACACTT
GGATACATGGCACCAGAGGTTCTTGAAGGAAAGCCTTACAACAGGAAATGCGATGTCTATAGCTTTGGGGTATGCCTCTGGG
AAATATACTGCTGTGACATGCCCTATGCTGACTGTAGTTTTGCTGAGATCTCTCACGCCGTTGTTCATAGGAATCTGAGACCA
GAGATTCCGAAATGCTGCCCGCATGCGGTGGCAAACATCATGAAGAGATGCTGGGACCCGAATCCAGACAGGCGTCCGGAG
ATGGAGGAGGTGGTGAAGCTGCTTGAAGCCATAGACACAAGCAAAGGTGGTGGAATGATAGCTCCGGACCAGTTTCAGGG
GTGCCTCTGTTTCTTCAAACCTCGAGGCCCCTGA
