# Supplementary material for: Genome-wide identification and analysis of mitogen activated protein kinase kinase kinase gene family in grapevine (Vitis vinifera)
Source: BMC Plant Biol. 2014 Aug 27;14:219. doi: 10.1186/s12870-014-0219-1 (PMC4243721; doi:10.1186/s12870-014-0219-1)
Supplement: Additional file 6: Figure S2. — Alignment of RAF subfamily from grapevine and Arabidopsis. The highlighted part shows the conserved signature motif. [file 12870_2014_219_MOESM6_ESM.pdf]

>VviMAPKKK4

MPSWWGKSSSKEVKKKENRESFIDSIHRKFRTVSEEKCNNRSGASQRHCGDTVSEKESRSRAQSRSPSPSTKVSRCQSFAERPHA

QPLPLPGPHLTSVVRTDSGINASKKQGLVEGSKTQMVLPLPRPGYVANRLDPTDAEGDLATASVFSYSSIDSEDPSESRLLSPQASD

YENGNRTTMNSPSSVMHKDQSPVLTPRKPREALRPANLLLNNQIHSTSPKWVPLSTHVPNFPVPQNGAFCSAPDSSMSSPSRSPM

RLFSPEQVMNSSFWTGKPYADIALLGSGHCSSPGSGHNSGHNSIGGDMSGQLFWPHSRCSPECSPIPSPRMTSPGPSSRIQSGAVTP

LHPRAGAAAAESPTNRPDDGKQQSHRLPLPPITISNSCPFSPTYSTSTTPSVPRSPGRAENPISPGSRWKKGRLLGRGTFGHVYLGF

NSESGEMCAMKEVTLFSDDAKSKESAQQLGQEISLLSRLRHPNIVQYYGSETVDDKLYIYLEYVSGGSIYKLLQEYGQLGEIAIRS

YTQQILSGLAYLHAKNTVHRDIKGANILVDPNGRVKLADFGMAKHITGQSCPLSLKGSPYWMAPEVIKNSNGCNLAVDLWSLGC

TVLEMATTKPPWSQYEGVAAMFKIGNSKELPTIPDHLSEEGKDFVRQCLQRNPLHRPTAAWLLEHPFVRNAAPLERPSLSSELEP

PPAVTNAVRSMAIGHTRNVLESEGVAIHQSRCSKTGSGSSDTHTPRNLSSPVSPIGSPLLHSRSPQHMSGRMSPSPISSPRTTSGSSTP

LSGGSGAIPFHHPKPINYMHEGIGIIPRSQSSLYANGSSSYQDPQPDLFRGMPQVSHVFREMISSESGSFGNQFGRPVHGDPRDLCD

AQSVLSDRVAQQLLRDHTNLHLSLDLNPGSPMLTRTNGI

>VviMAPKKK5

MPWWQNIAFSSPSSSSSTSSSRRRLTRARKLRHVRGNHIDALVRSRSSAEPKDLLPLPSSSSSFDHSSERLSSVVPHPLPRPELALLF

RRNGGSNSNLNCDRPLPSPKEGLSRGFEDRDKGNSFVGDGNGEVAVSSTATGSKVGYNEARKSMEQFDTLSSRYLPQGQNSVKN

NRVNFKLDVPTRSAPTSSLSSPAVSPQRTSPGNLFLSQNVSPQVFQGWSAPEMPVFDMVTGFTPQMSPEQTMFSTDNSPLHSPTV

KSPHVNPRSPSGPASPLHPKISLETSTARRENNSHANVHRLPLPPGVVAPPQASSIHPVIAKTESFPMTTQWQKGKLIGRGTFGSVY

VATNRETGALCAMKEVELLPDDPKSAESIKQLEQEIKILSQLKHPNIVQYFGSETVEDRLYIYLEYVHPGSINKYVREHCGAITESV

VRNFTRHILSGLAYLHSTKTIHRDIKGANLLVDASGVVKLADFGMSKHLTGAAADLSLKGSPYWMAPELMQAVMQKDHSSDLA

FAVDIWSLGCTIIEMLNGKPPWSEYEGAAAMFKVMRESPPIPKTLSSEGKDFLRCCFRRNPAERPPAIKLLEHRFLKNSTQLDVPLL

TQAFSGMKLPDKANKSREKSNDRVDPVPISPRKKTSKGKKASGTGQQSHRETSDLTVASHHSPRSTLEALPSLSPPHSGQRAYHL

SPPANVPSPINYGAKKKRTWG

>VviMAPKKK22

MQDIFGSVRRSLVLRSPDGDDTSPGTLVDKINSCIRKSRVFSRASPPPLIPKDATAPSIRCRKGELIGCGAFGRVYMGMNLDSGELIA

VKQVLITTSNATKEKAQAHIRELEEEVKLLKNLSHPNIVRYLGIVREEETLNILLEFVPGGSISSLLGKFGSFPEAVIRMYTKQLLLG

LDYLHNNGIMHRDIKGANILVDNKGCIKLADFGASKQVVELATISGAKSMKGTPYWMAPEVILQTGHSFSADIWSVGCTVIEMA

TGKPPWSQKYQEVAALFYIGTTKSHPPIPAHLSAEAKDFLLKCLQKEPDLRPAAYELLKHPFVTGEYNEAQLALQTSVMENSETP

VSTSEENLPNPQMTNDSSLLDVCEMGNLSCSTGYPENSESKPNWGTSSSIDMCQIDDKDDVMMVGEVKFNSILLPDNFNKSFNPI

SEPSHDWSCEIDGNLRPEHSGMDMDTNPSLDMPAGSSGISNKGGNDFSFPCGPSVSEDDDEVTESKIRDFLDEKAIELKKLQTPLY

EEFYNTLNAACSPSVAERTQDENVINYLKLPPKSRSPSRAPIGTPSAAVDSTSTASPGSCGRRMSNVGNAGDQTAQDTPSPPHND

WKGLLVDSQQEPNSPSVSFYEIQRKWKEELDQELERKREMMRQAGKTSSPKDRALNRPRERSRFASPGK

>VviMAPKKK23

MQDIFGSVRRSLVFRPASGEDGEYGGYGGPVEKIWSSIRKSGIGLFSRQSVRALPPIPKDEAPSIRWRKGELIGCGAFGRVYMGMN

LDSGELLAIKQVSIAANSASKEKTQAHIRELEEEVKLLKNLSHPNIVRYLGTAREDESLNILLEFVPGGSISSLLGKFGSFPESVIRM

YTKQLLLGLEYLHKNGIMHRDIKGANILVDNKGCIKLADFGASKKVVELATMTGAKSMKGTPYWMAPEVILQTGHSFSADIWS

VGCTVIEMATGKPPWSQQYQEVAALFHIGTTKSHPPIPEHLTAEAKDFLLKCLQKEPNLRPAASELLQHPFVSGEYQEPHPVFQTS

VMENSGNMMATSETDLKSFPNPVIRRSNCTSSNNVCNMGSVRCSTVYPEKFSGTGPLWGANSCDDDMCQIDDKDDFIVHQSVK

FGSAILSDHLNKSFNPMCEPTDDWPCKFDESPELTKSGANLSSHQTISKPAGSPRASNERENDFTFPCGPLVGDDDDEVTESKIRA

FLDEKALDLKKLQTPLYEEFYNSTLNAAGPPSAVGKSHDNVTNFLNLPPKSRSPNQTPTRRLSAAVDAACISNSANHTKRGLNVG

TISDRTLKEIQSPQLSEWKELLLDAQQEPVSLSTNFSERRRKWKEELDQELEKKRELLRQAGVGGKTASPKDQILNRPRERLRFAF

PGK

>VviMAPKKK24

MKLLWCSVYARGSTWKNTDHLATKSANSPRGFSQDLNDEACPYNLRVNIPARSAPTSGFSSPALSPQRFSPGERLPSSYAAIQDFQ

SPGFDRLPGCSSQMSPLKTPHTPDYSPLHSPTVQSPCISPCLSPKSPTGIAFSLYPKLLPGSHVTWPEKNGHVTVHPLPLPPIALMPS

ELPLPPKALTPSESAISHHTAEKPNVPSMKSQWQKGKLIGRGTFGSVYVATNRETGALCAMKEVDIIPDDPKSSECIKQLEQEIKVL
HHLKHPNIVQYYGSEIVDDHFYIYLEYVHPGSINKYVDHFGAMTENVVRNFTRHILSGLAYLHSTKTIHRDIKGANLLVDSFGVV
KLADFGLAKFLTGQACDLSLKGSPHWMAPEVMQAVLRKDANPDLAFAVDIWSLGCTIIEMLNGRPPWSEFAAPAAMFKVLHES
PPLPETLSSEGKDFLQHCFRRNPAERPSAAMLLDHSFVRSSQDQNVSGFSQAFSGMQLVDKPRSPGDAMKHKIHSMPLSSGTQT
MNRNVLR

>VviMAPKKK25

MPAWWGRKSSKCKEEVQQQNPESTLYNISKISIRNDKKNGKDKPKSFDEGLFSRNSPRSSKDYGALTVSGGGGSSGFSGFDSDCG
DKIRGHPLPLPSAGIEHGVGSGSGSVSSVSSSGSSDDHPSPHDHAPFGVYRGQGETKSNTRSRSPGPGSRSATSPLHPRFSTSNIDSL
TGKQEEGRSCHRLPLPPGSPTSPSTLSSTRTCVVTESTTCNMSKWKKGRLLGRGTFGHVYVGFNSENGQMCAIKEVKVVSDDHT
SKECLKQLNQEINLLSQLSHPNIVQYYGSEMGEETLSVYLEYVSGGSIHKLLQEYGPFKEPVIQNYARQIISGLAYLHGRSTVHRDI
KGANILVGPNGEIKLADFGMAKHINSSSSMLSFKGSPYWMAPEVVMNTNGYSLAVDIWSLGCTILEMATSKPPWSQYEGVAAIF
KIGNSRDVPEIPDHLSNDAKSFVRLCLQRDPSARPTALQLLDHSFVRDQATTRIANIAITKDAFPSTFDGSRTPTALELHSNRTSLTL
FDGDYVTKPVGTVSRAAKNSRDSVRTITSLPVSPCSSPLRNYGPAHKSCFLSPPHPSYPIVGQSSYNSNDYSLYPTRAITKYTHDP
WSDNPPFRSLTPNGSPRTRPI

>VviMAPKKK26

MPSWWGKSSSKEAKKKTNKESFIDTLHRKFKIPSEGKVSNRSGGSHRRCSDTISEKGSQSRAESRSPSPSKLVSRCQSFVERPNAQ
PLPLPGRHPASVGRTDSGISISTKQRLEKGSKSSFLPLPRPRCIGGRPDPTDLDGDFVASVYSEGSTDSEDAADSHHRSPQATDYDN
GTRTAASIFSSVMLKDQSPVAHVNAREAQKPANLLFSNHISPTSPKRRPLSSHVPNLQVPYHGAFGSAPDSSMSSPSRSPLRAFGT
DQGLNSAFWAGKPYSDVTLLGSGQCSSPGSGQNSGHNSMGGDMSGQLFWQPSRGSPEYSPIPSPRMTSPGPSSRIHSGAVTPLHP
RAGGAASESQTSWPDEGKQQSHRLPLPPVAVSSSSPFSHSNSPAASPSVPRSPGRAEAPTSPGSRWKKGKLLGRGTFGHVYVGFN
SESGEMCAMKEVTLFSDDAKSKESAKQLGQEIVLLSRLCHPNIVQYYGSETVGDKLYIYLEYVSGGSIYKLLQEYGQLGELAIRS
YTQQILSGLAYLHAKNTVHRDIKGANILVDPSGRVKLADFGMAKHITGQSCPLSFKGSPYWMAPEVIRNSNGCNLAVDIWSLGC
TVLEMATTKPPWSQFEGVAAMFKIGNSKDLPAIPDHLSDEGKDFVRQCLQRNPLHRPTAAQLLEHPFVKNAAPLERPILSPETSDP
PPGVTNGVKSLGIGHAKNLSSLDSERLAVHSFRVLKTGSHSSDPHIARNISCPVSPIGSPLLHSRSPQHLNGRMSPSPISSPRTTSGPS
TPLTGGSGAIPFPHLKPSVYLQEGFGNVSKPLNNPYSNGPSYHDPNADIFRGMQLGSHIFPESDALGKQFGRTAHVELYDGQSVL
ADRVSRQLLRDQVKMNPSLDLSPSSMLPSRNTGI

>VviMAPKKK27

MERNCTSDGFFFAVKEVSLLDQGGKGKQSIYQLEQEISLLSQLEHENIVRYYGTNKDDSKLYIFLELVTKGSLLSLYQKYHLQESQ
ASVYTKQILNGLKYLHEQNVVHRDIKCANILVDVHGSVKIADFGLAKATKLNDVKSCRGTPFWMAPEVVNWKNEGYGLATDI
WSLGCTVLEMLTRRPPYSHLEGATKLNDVKSFKGTLRWMAPEVFNQKNEGYGLAVNIWSLGCTVLEMLTCRPPYSNMEDGQVI
SKIYRSEPPDVPDSFSSDARDFILKCLQVNPSDRPTAGELLDHPFVKRPSGPQSPRTSGIQP

>VviMAPKKK28

MESNCTSDRIIFVVKEASLLDQGSQGKQSIYQLEQEISLLSQFEHENIVRYYGTNKDETKLCIFLELAPEGSLLNLYRKHKLLEPQV
SEYTRQILNGLSYLHGKHVIHRDVKCANILVFENHIVKLADFGLSKVSFISRVTISFKGSPFWTAPEVVNAVYRKNDCYGLAADIW
SLGCTVLEMLTQQHPYPQYEWMQALFRIGHGELPFVPDSLSIDARDFILKCLQVNPSDWPTARQLLDHPFVKSPLHPFIGPASPRA
NGIRP

>VviMAPKKK29

METGLLLSIIFLATTFSFIHSTKANPRADIIARICSNDYAHNFSNYLDSYSKIITQLRDELPKTKFAFKEAGEPPDKIYVLAQCMDDL
SFQDCQACFSQISSLFPGCFPATGGRVYLDGCFLRGDNYSFFQDTLTPMDYLVTAFVFLVFFFIFLADISGVQNFDDVAKSVIDELV
RMTPSRDGYAAYDESANGITVYGMASCWKTLDRDRCASCLASAAISAFACFPSAEGRVLNAGCFLRYSDYKFYQGFDFSFYSFS
WGSLADATLSFISHVVGVVSVCILAIIIGFFIGKAAYQKRNQQNEANGNSIMRKFVMIEHIPQIIFLINEEIEVDSSVVKRSLQFKYT
TLEKATDYFNEANKLGQGGFGEVFKGTLRDGREIAIKRLFITGQSGAQEVYNEIDIIGSACHKNLVRFLGCCFTRHDSFLVYEFLP
NRSLDRVLFDTEKKKELPWKIRLGIIMGTAEGLEYLHKDCHVRIIHRDIKASNVLLDFRYRPKIADFGLARFYSTDRALTGTAIAG
TLGYMAPEYLAQGRLTDKVDVYSYGVLILEIVSGVQNNKFQLDDSLNTLATATWKHFQSNTMTEIIDKGMEIEDMEEVTRVIQV

GLLCTQESPTLRPAMTEIIQMLKQKDVSLPIPSKPPFTEENLTISPALGCPRRPAVDAYDLCISCDHSDTELR

>VviMAPKKK30

MGLVGVESGEEGIGHSEPPDPDVLEIDPTSRYIRFKDILGKGAFKTVYKAFDQVDGIEVAWNQVRIDEVLQSPDELERLYSEVHLL
KSLKHKNIIKFYNSWIDDGNKTVNIITELFTSGSLRQYRKKHKKVDMKAVKGWARQILMGLNYLHNHNPPIIHRDLKCDNIFING
NQGEVKIGDLGLATVMQQANARTVIGTPEFMAPELYDENYNELADIYSFGMCMLEMVTLEYPYSECRNSAQIYKKVSNGIKPAA
LSKIKDLEVKMFIEKCLVPASQRLSAKKLLNDPFFQVDGLTKNHPLQLPDIVIPKTGAFGDRCLLSEGPTSLQNRPLAMDLDAVDD
DELPIITSMDNSVDGGPYSLCMEVQRAKGGNFFLLKGEGNDENSISLILRIADQNGRLRNIHFMFYLDSDTALSVSSEMVEQLELA
DQNVTFIAELIDLLLIMLIPTWKPCVPIDHLVALNRMQTSNGHHEDLQCPEHGECLVGSFEGVCETDNLLSPHVYPNSTSFEGYIET
MQENPKHLSLDEIKTHADLGLPSSATVEDHGSDMSYVSATSNEGSDKKYSHNAYLSAESGCMDYNEYGSKRGVRQSLSAVQTS
SCNLDKGKATDIGSNGAVTSSDYPIDSSLSDQVESENMILELEMIELQYHEAVKEIAKRRQEAIRETKKRLSQKRIESVI

>VviMAPKKK31

MNSDKFETDDSDFEEKDPTGRYVRYNEFLGKGAFKTVYKAFDEVDGIEVAWGQVEIEDLLQSPQQLERLYSEVHLLKSLKHDNII
KFYNSWVDDTNKTINLITELFTSGSLRQYRKKHKNVDLKAIKNWAKQILRGLHYLHSHNPPIIHRDLKCDNIFVNGNNGEVKIGD
LGLAIVMQQPTARSVIGTPEFMAPELYEEEYNELVDIYSFGMCILELVTCEYPYNECKNPAQIYKKVSSGIKPASLGKVSDPQVKQ
FIEKCLVPASLRLSAQELLKDAFFATENSKEPVYNHMHVDDSLQSSNFMPNLMNLPKPELQPMDMDPNYKKLSVSTHMKSISGT
PHFRALQFERFNKNNLFKLRGEKIDDNSISMTLHIADPCGRAKNIHFAFYLDSDTALSIAGEMVEQLDLYNEDVAVIAELIDVMISE
LVPTWKPAFESMLCGANSSCEDSLVLHNGGTSLRHPSDSGSAKGTSDAVTEHLISLSANGEEQSTVESALSGMSTKDDATVASDA
NDIKSLECPDDECYEASDRCCFNGDRQVLDHERHKEGRYNGNIGEPVAMNGFTKDWEISCIESCSGMSNSLSLSSICSLSLADKD
PSDELKLEVDTIDTQYHQCFQELLRMREEAIEKAKNRWITKSRYQPLL

>VviMAPKKK32

MQGAEDEDEAAYVEKDPTGRYVRFKEILGKGAFKTVYKAFDEVDGIEIAWNQVKIDDVLRSPEDLEKLYSEVHLLKSLKHENII
KFYNSWVDDKKKTVNMITELFTSGSLRQYRKKHKNVDMKAIKNWARQVLRGLVYLHSHNPPIIHRDLKCDNIFVNGNHGEVKI
GDLGLAIVMQQPTARSVIGTPEFMAPELYEEEYNELVDIYSFGMCMLEMVTFEYPYNECKNPAQIYKKVTSGIKPASLCKVTDLQ
IKEFIVKCLAPASERLPAKELLKDPFFQSENPKEPIRVPLQLPSRSPKSIILSKSGPFSMDIDPDHPQLSSSTSTENNGSPDFPVLEFQR
MYKSSEFRLRAKKINDNSISLTLRTVDSYGPVKNIHFPFSLDTDTVHSVVGEMVEQLELAEHEVAFIADFIDYVIMRLLPGWKPPR
DDPLGGARSPNAEPPVLGNGNNHDCTISHGDGNSSPNLANAEDQDSLASAGLVTLTVDASKKNDKTVGFGDYNIGGNYKGSNG
GHASEQESRDPYHEDYKLQRNNSSIEEFTPMNKFQKSTVLSFDDLSGLSNVRSLTCSCSSLSLADIDQDPGLKQELDAIDLQYQH
WFQELSRMRVEALEATKKRWMTKKKLAVQ

>VviMAPKKK33

MPQVLSSEQDPDDPDTEFVEIDPTGRYGRYKEVLGKGAFKKVYRAFDELEGIEVAWNQVKVADLLRNSEEFERLYSEVHLLKTL
KHKNIIKFYISWVDTRNENINFITEIFTSGTLRQYRKKHKHVDLRALKKWSRQILEGLLYLHSHDPPVIHRDLKCDNIFVNGNQGE
VKIGDLGLAAILRQARSAHSVIGTPEFMAPELYEEEYNELVDIYAFGMCLLELVTFEYPYVECANAAQIYKKVTSGIKPASLAKVK
DPRVRAFIDKCIANVSDRLSAKELLRDPFLQSDEENGSVGRSLQPHPHHSGSHDHFNTGTSSKVSLPESSRDFKVQGQRRDVNTIF
LKLRIADSKGHIRNIHFPFDIGADTAISVAGEMVEELDLTDQDVSTIAAMIDSEIRSIISDWPPSREVFGDNLSTEVAISDICPPESEGD
ALPLMNESATSSCGLVLERLPSGRRYWSDSPKAVGGNSPIRPAFSNLSSQVDSVTTEGRFSELNEQSLASPRDGDKLNTAASLDKR
EDERVCGDDDVEEKEASISAETQFSDQNDVAVELLGGYRAPSWGGNCKILRETELGDAKVIVEKLKHLFVKQQKELDELKRKHE
LAILDLVKELPPDIRNKVSSLCNLKISGSFSKMEPMSSEVSNLAVYNNLDTYSIGPTNVKLSLPKKG

>VviMAPKKK34

MPAVSPDQSDRDSEPFVEVDPTRRYGRYNELLGCGAVKRVYRAFDQEEGIEVAWNQVKLRAFSDDKPMIDRLFSEVRLLKTLKD
KNIIALYNVWRNEDHNTLNFITEVCTSGNLREYRKKHRHVSMKALKKWSKQILKGLDYLHRHEPCIIHRDLNCSNVFINGNVGK
VKIGDFGLAATVGKSHVAHSVLGTPEFMAPELYEEDYTELVDIYSFGMCFLEMVTLEIPYSECDNIAKIYKKVISGARPRAMDKV
RDPEVKAFIEKCLAKPRARPSASELLNDPFFHGIDDDEIDNSDS

>VviMAPKKK35

MYRTKLGELTNEGKDESGYAETDPTGRYGRLDEVLGKGAMKTVYKAIDEVLGMEVAWNQVKLNEVLRSPDELQRLYSEVHLL

SALNHDSIIQFYTSWIDVERKTFNFITEFFTSGTLREYRKKYKRVDIRAIKCWARQILRGLVYLHGHDPPVIHRDLKCDNIFVNGHL
GEVKIGDLGLAAILRGSQSAHSVIGTPEFMAPELYEENYNELVDVYSFGMCVLEMLTSEYPYSECSNPAQIYKKVTSGKLPGAFY
RIQDLEAQRFIGKCLVTASKRLPAKELLLDPFLASDEAKRLPKPKLGSQKPFLNDIRIEKLRLSDDRVRTNMTITGTLNPDDDTIFL
KVQTADKDGSARNIYFPFDIVTDTPIDVAMEMVKELEITDWEPFEIADMIDGEISALVPQWKKWDMPQQHHYAFDYQEEDEGHN
HPFRSFSSCSSSQASFPCLSTSHRLDTMAQGGDWLKDDLFDDTSSESSAHSGKYSNLNYFSGNEHCSETSTLRREQHPGAKTQKS
TRFCPEENSSTRKALPGKSYKQGKVLQESQRAPGSKDKFAMETIRLTRNRSLVDVRSQLLHRTLVEEVHKRRLSKTVGAVENIGF
QAPCNVSGKVSQKPTGAHSTRTTRDGKGQGSQRRRA

>VviMAPKKK36

MNNTRLGECRGGARQQFGYVETDPSGRYGRFREILGKGAMKTVYKAFDEFLGMEVAWNQVKLNDVFNSPDDLQRLYSEVHLL
KNLDHDSIMRFHTSWIDLDGGTFNFISEMFTSGTLREYRQKYKRVDIGAVKNWARQILHGLAYLHGHDPPVIHRDLKCDNIFVNG
HLGQVKIGDLGLAAILRGSQHAHSVIGTPEFMAPELYEEEYDELVDVYSFGMCVLEMLTSEYPYCECSNPAQIYKKVTSGKLPEA
FYRIEDVEAREFVGRCLEHVAKRLPAKELLMDPFLAVDHGEQMLPMLKISSQKPSPNGTVEKIPSFQTNPRKRSTDMTITGTINPD
DYTIFLKVAISDKDGLSRNIYFPFDIGSDTPIDVAAEMVRELEITDWEPFEIAKMIEEEIFALVPSWKQCTSPENHQHSFEYEEEEEEE
DDDDETYHPFYCYASESSRVALQDLSISCEIQSQHRNHMISGEDWFQEGLLINDDASSQSSLNSDKYSTLVYCSVTENDIDHLAPK
RVEPIFTASTHKSTRFCPEEGTSSWNQCNGSRRPYDSNCHRKLSRIKSFVDVRSQLLRRSLMEMINKRRLFKTIGAVENIGYQEPG
KFPKEMSMTGGLSGNSPRNSEKQRFKC

>VviMAPKKK37

MFGELEPDYSEFVEVDPTGRYGRYNEILGKGASKTVYRAFDEYEGIEVAWNQVKLNDFLQSPEELERLYCEIHLLKTLKHNNIMK
FYTSWVDPANRNINFVTEMFTSGTLRQYRLKHRRVNIRAVKHWCRQILRGLLYLHNHKPPVIHRDLKCDNIFVNGNQGEVKIGD
LGLAAILRKSHAAHCVGTPEFMAPEVYEEEYNELVDIYSFGMCILEMVTFEYPYSECTHPAQIYKKVISGKKPDALYKVKDPEVR
QFVEKCLATVTLRLSARELLKDPFLQSDDYGSDLRPIEYQRDLGEVGPLPRLPHYGIHHSYSSLRNEYSGYPDFEPENGLDCHPVE
FERNEIDLFTYQEDEHLENVDISIEGRKRDDHGIFLRLRISDKEGRVRNIYFPFDMETDTALSVAMEMVSELDITDQDVTKIADMID
DEIASLVPGWKMGLGIEESQNYGHDASFCHHCASNGSPLDYVSPNNPGTKNLQVLQCSRQGCAAVHGRFEEITYRVEGSEQCVT
EGAPVVSSQSDGMQYADIWAQREGPELSSEGSREIQSDEEHESLDKSIYGKDERVINIDNQSESNAKNSFAPLDDYENEIRQELRW
LKAKYQMQLRELRDQQLGAKPKWLSLTPNSDSMEHSRDNKVSPSSLSTPLEGEDNDPPLKSFPCGKHFNSFFPVDTERGCANLA
YRRPHNREPVSESCSPEDMVTAKSFFTGTLLPQSLHRATSLPVDAVDF

>VviMAPKKK38

MEIAAQLKRGISRQFSTGSLLRTLSRQFSRQSSLDPRRTNLRFSLGRQSSLDPIRRSPVNEELSVPENLDSTMQMLFMACRGDVKG
VEDLLNEGTDVNSIDLDGRTALHIAACEGQIEVVKLLLSRKANIDARDRWGSTAAADAKYYGNVEIYNILKARGAKTPKIRKTP
MAVANPREVPEYELNPLELQVRKSDGITKGSYQVAKWNGTKVSVKILDKDSYSDPDSINAFKYELTLLEKVRHPNVVQFVGAVT
QNIPMMIVSEYHPKGDLGSYLQKKGRLSLSKALRYALDIARGMNYLHECKPDPVIHCDLKPKNILLDSGGQLKVAGFGLLRLSK
MSPDKVKLAQSGSHIDASNVYMAPEVYRDELFDRSVDSFSFGLILYEMIEGVQPFHPKPPEEAIKMICLEGKRPPFKSKSRSYPPD
LKELIEECWNPEPVVRPIFSEVIVRLDKIVGHCSRQAWWKDTFKLPWK

>VviMAPKKK39

MAPEVYRRESYGKSIDVFSFAVIVHEMFHGKTSKRAENPEYVADKQAYEDSRPPLSSYVYPRPIKTLLRNCWHKNPEVRPTFEAII
LELEEIQVSMLDKKAACHDYQSHEYHVNGYVFFIMVTLLYLYEPAWRLLKPNIAQMKMYDIKLVVMLAKTETLGASYKLHVLG
MKVRMQPQTMDSEGPYQLLHCSSKGDKEGVIQELEKGVDANLADYDKRTALHLAACEGCEEIVVLLLEKGADVNSIDRWGRT
PLSDARSFGHEKICKILEAQGFHVLQTYKTHISEASFHYMQRTPCYEIDHTEVDMDEATLIGEGAYGEVYLVKWRGTEVAAKTIR
SSIASDPRVKNTFLRELGLWQKLRHPNIVQFLGVLKHSERLIFLTEYLRNGSLYDILRKKGRLDPPVAVAYALDIARGMNYLHQHK
PHAIIHRDLTPRNVLQDEAGRLKVTDFGLSKIAQEKDAVGYKMTGGTGSYRYMAPEVYRRESYGKSIDVFSFALIVHEMFQGGP
SNRAENAEYVADKRAYEDSRPPLSSFVYPEPIKT

>VviMAPKKK40

MAVESKTAVRFTLGKQSSLAPERARDEALTEGEQGDVEGIDPRVRLMYLANEGDLEGLRELLDSGMDVNFRDIDNRTALHVAAC
QGFSDVVEFLLKNGAEIDLEDRWGSTPLADAIHYKNHDVIKLLEKHGAQHLMAPMHVNNAREVPEYEIDPKELDFTNSVDITKG

TYRIASWRGIQVAVKRLGDEVIIDEDKVKAFRDELALLQKIRHPNVVQFLGAVTQSSPMMIVTEYLPKGDLHAFLKRKGALKTAT
AVKFALDIARGMNYLHEHRPEAIIHRDLEPSNILRDDSGHLKVADFGVSKLLKVANTVKEDYPLICQETSCRYLAPEVFKNEAYD
TKVDVFSFALILQEMIEGCPPFSAKPENEVPKVYAAQERPPFRAPSKLYSHGLKELIEECWNENPTKRPTFGQILTRLDRIYNHLGQ
KRRWKVRPLKCFQNLEAMWKKDHSDLSTSSRSSRPNR

>VviMAPKKK41

MSCRDDNSGGHSSTSAAGKDKASDKQKEKARVSRTSLILWHAHQNDAAAVRKLLEEDQSLVHARDYDSRTPLHVASLHGWIDV
AKCLIEFGADVNAQDRWKNTPLADAEGAKKHSMIELLKSYGGLSYGQNGSHFEPKPVPPPLPNKCDWEIDPSELDFSNSSIIGKG
SFGEILKACWRGTPVAVKRILPSLSDDRLVIQDFRHEVNLLVKLRHPNIVQFLGAVTDKKPLMLITEYLRGGDLHQYLKEKGSLSP
STAITFAMDIARGMAYLHNEPNVIIHRDLKPRNVLLVNTGADHLKVGDFGLSKLIKVQNSHDVYKMTGETGSYRYMAPEVFKH
RKYDKKVDVFSFAMILYEMLEGDPPLSNYEPYEAAKYVAEGQRPMFRAKGYITELKELTEQCWAADMNHRPSFLEILKRLEKIK
EILPPDHHWNIFTA

>VviMAPKKK42

MKESSDGFVRADQIDLKSLDEQLEKHLNRVWTMDKNKKKEDDSSSAAAAIPTLAPSTTASTTAPTTARQDWEIDPSKLIIKTVIA
RGTFGTVHRGVYDGQDVAVKLLDWGEEGHRTEAEIASLRAAFTQEVAVWHKLDHPNVTKFIGATMGSAELNIQTENGHIGMPS
NICCVVVEYLPGGALKSYLIKNRRRKLAFKVVIQLALDLARGLSYLHSQKIVHRDVKTENMLLDKTRTVKIADFGVARVEASNP
NDMTGETGTLGYMAPEVLNGSPYNRKCDVYSFGICLWEIYCCDMPYPDLSFSEVTSAVVRQNLRPEIPRCCPNSLANVMKRCW
DANPDKRPEMDEVVAMIEAIDTSRGGGMLPVDQPQGCFCFRKYRGP

>VviMAPKKK43

MKEKSDGFVRADQIDLKSLDEQLQRHLSRAWTMEQKKEQQEERPNTREEWEIDPSKLVIKSVIARGTFGTVHRGVYDGQDVAV
KLLDWGEEGHRTEAEIASLRAAFTQEVVVWHKLDHPNVTKFIGATIGSSELNIQTENGHIGMPSTVCCVVVEYLPGGALKSYLIK
NHRRKLAFKVVVQLALDLARGLSYLHSKKIVHRDVKTENMLLDKTRTLKIADFGVARMEASNPNDMTGETGTLGYMAPEVLN
GNPYNRKCDVYSFGICLWEIYCCDMPYPDLSFSEVTSAVVRQNLRPEIPRCCPSSLANVMKRCWDANPDKRPEMDEVVSMLEGI
DTSKGGGMIPLDQPQGCFCFGKYRGP

>VviMAPKKK44

MDLRSDDEVLVTAKAEKSRTGEGETVSDKSSISSSDLGSKKDGGNTSNKDLFLRADKIDFKSWDIQLDKHLSRVISRDREVNTNT
KKEDWEIELSKLDIRSVIAHGTYGTVYRGVYDGQDVAVKLLDWGEDGLATAAETAALRTSFRQEVAVWHKLDHPNVTKFIGAS
MGTSDLRIPSNSISSDGRNPVPSRACCVVVEYLPGGTLKKFLIRNRRKKLAFKIVIQLALDLSRGLSYLHSKKIVHRDVKTENMLL
DAHRTLKIADFGVARVEAQNPRDMTGETGTLGYMAPEVLDGKPYNRKCDVYSFGICLWETYCCDMPYPDLSFADISSAVVRQN
LRPDIPRCCPSSLASIMRKCWDGNPDKRPDMDEVVRLLEAIDTSKGGGMIPEDQVSSCCFFSVARGP

>VviMAPKKK45

MAPQLTIDENLLVDPKLIFIGSKVGEGAHGKVYEGRYGDQIVAIKVLHRGSTSEERAALEGRFAREVNMMSRVKHENLVKFIGAC
KDPLMVIVTELLPGMSLRKYLTSIRPKRMDIHVALSFALDIARAMECLHANGIIHRDLKPDNLLLTANQKSVKLADFGLAREESVT
EMMTAETGTYRWMAPELYSTVTLRQGEKKHYNNKVDVYSFGIVFWELLTNRMPFEGMSNLQAAYAAAFKQERPSLPEDISPDL
AFIIQSCWVEDPNMRPSFSQIIRMLNTFHFKVTPPPSSLLEESDTNGAAMSSNGTMTELSARTRGKFSFLRQLFAAKRTRNSQ

>VviMAPKKK46

MPVAIKMIQPNKTSAVSPDRKEKFQREVTILSRVKHENIVKFIGASIEPTMMIITELMKGGTLQQYLWSIRPNSPDLKLSLSFALDIS
RVMEYLHANGIIHRDLKPSNLLLTEDKKQIKVCDFGLAREETAGDMTTEAGTYRWMAPELFSTVPLPRGAKIHYDHKVDVYSFA
IILWELLTNRTPFKGVQSILIAYAAANRTCTTTPKGDKP

>VviMAPKKK47

MVEGPKFTGIIGGGGNHNHDNNYFDFTQGFYQKLGEDSNMSIDSLQTSNAGLSVSMSVDNSSVGSNDSLTHILNHPGLKPVATH
NYSVGHSVLRPGKGKVTHALNEDALARALMDTRYPTEGLENYDEWTIDLRKLNMGTAFAQGAFGKLYRGEYNGDDVAIKILER
PENSPERAQVMEQQFQQEVMMLATLKHPNIVRFIGACRKPLAWCIVTEYAKGGSVRQFLMRRQNRSVPLKLAVKQALDVARGM
AYVHGLGFIHRDLKSDNLLIAADKSIKIADFGVARIEVQTEGMTPETGTYRWMAPEMIQHRPYTQKVDVYSFGIVLWELITGLLP
FQNMTAVQAAFAVVNKGVRPIIPSDCLPVLSDIMTRCWDANPEVRPPFTEVVRMLENAEIEIMTTVRKARFRCCMTQPMTTD

>VviMAPKKK48

MEETRDDAGPAEQGSPVTTWWPSDFIERFGSVSLVSQEEILSNKNSNSNTEQDELSSQTASQILWSTGMLSEPIPNGFYSVIPDKKL
KEIFDDIPTLDELYALGSEGVRADIILVDAVRDKKLSMLKQLIVALVKGLNSNPAAVIKKIAGLVSDFYKRPNLELSPAKAALEETS
HVSENRVAQLLGQIKHGSCRPRAILFKVLADTVGLESRLMVGLPNDGAIGCVDSYKHMSVIVMLNSGELLVDLMRFPGQLIPRST
RAIFMTHISAAGESDSAENDSCDSPLEPNSPLYGFSDRVDPDSTEKDEGLQFQRRLEASSNVSGPSLRNVMLRSTPSIDRKLSLSHS
EPNIATTFWRRSRRKVIAEQRTASSSPEHPSFRARGRSMLSGDRKSFRDYADDIAASSYRSDGASTSTSETRRIRRRSISITPEIGDDI
VRAVRAMNETLKANRLMRDQGDDRAFSSNPDIQKNVSDFHLDGHGEISHGSSSMYTLPREQISSQKAISLPSSPHEFRSQTSGRS
GTSDIVNDEMVSIWNRVLEKPMFHSKPLLPFQEWNIDFSELTVGTRVGIGFFGEVFRGIWNGTDVAIKVFLEQDLTAENMEDFCN
EISILSRLRHPNVILFLGACTKPPRLSMITEYMEIGSLYYLIHLSGQKKKLSWRRRIKMLRDICRGLMCIHRMKIVHRDIKSANCLV
NKHWTVKICDFGLSRVMTDTPLRDSSSAGTPEWMAPELIRNEPFTEKCDIFSFGMIMWELCTLNRPWEGVPPERVVYAVAHEGS
RLDIPEGPLGMLIADCWAEPHQRPSCEDILSRLQDCEYTLC

>VviMAPKKK49

MCNKGIARVSDSVDQKQHQAVYLMDSPSATPSSAHGSNDENPRVKFLCSFSGSILPRPQDGKLRYVGGETRIVSVPRDIGYEELM
GKMKELFDMAAVLKYQQPDEDLDALVSVVNDDDVTNMMEEYDKLGSGDGFTRLRIFLFSHPDQDGGSSHFVDVDDTERRYVD
ALNNLNDASDFRKQQVGESPTMSAIDDIHLAEQFFNSISLEGGLHNQRNCEMPMSQFNLHHLTIPHMGSGQHQPVAQRYNEMES
QWNPAYFSPRHHGHHDARPLAEYPSSPSSARFRMPFGELPDKCIDRLPEEYSRQPVNPQAPYDHQPQASDNVVWLPTGAISSEK
AGFPGSMLHGPNVFEGNSICEHCRMTFHRHLEQPNMGNGLPPVANPCAECPPGRESFLLNTDAKMQHGIYPKEHNDPRSLYNET
HNHERGWILQHQLNPRAEDARAQISGAGRLNDPYIVDGSGVNFPVAHGNLLDNHHVSSNYVHHEDPRYIRTGPELGNGVFHDQ
AAAAGPAINVPPLEERAVRYGNLPYPYGADNLYQVSHGHVPAHALWRNVQNPMHGAPSYEASTSTCQASGSVNPGPIRGTREGS
PRFCVGLDNQNPWGESSQKILGFDGSALPDYSYGHATKLNPNTHGQEGQHPFTPGPVPSPSDMLKFAAPMEPLHFTNSSPTLMD
DKFVASANLSYNPESRNDNNVNQTVIMEAKQAFREGKEEIHMEKVEDNDMPVTSLPEKNNNADKKCEVASLEPVNLPAEDNVF
KPVVNDCAPLEEDAKLDVSNLSFLPELIASVKRAALESAEEVKAKVQENADAVHASSTKEASNELETANALGDLELDSDNDNV
NTFKIEPTKAEEEALSRGLQTIKNDDLEEIRELGSGTYGAVYHGKWKGSDVAIKRIKASCFAGRPSERERLIADFWKEALILSSLH
HPNVVSFYGIVRDGPGGSLATVTEFMVNGSLKQFLQKKDRTIDRRKRRIIAMDASFGMEYLHGKNIVHFDLKCENLLVNMRDPH
RPVCKIGDLGLSKVKQHTLVSGGVRGTLPWMAPELLSGKTNMVTEKIDVYSFGIVMWELLTGDEPYADMHCASIIGGIVNNTLR
PQIPRWCEPEWKYLMESCWASDPAERPSFSEISQKLRNMADAPITK

>VviMAPKKK50

MKEPSMLRRRRPNSSKKLKLVCSFNGRFQTRPPSGKLGYVGGDTRIISVDRGIGFMKLRSKISELCPDIRSFSLKYRLPESDPVHG
DTTNLVLIASDDDVRCMVDEYDKMDFYGQQTRLRIFVFRDNGYVNVNLPMNCIENINDYVCGKKGFGVEGKETCVKGVSDFG
DYISNVFQSRNSGSYLFGLQFDTKEINNPATVVAGGRYSDRSLRKVILKQRFSAKKPAPISSFCSGEREFRRSDEQKYCYPLIDLAP
EALVPKSKQTANLNFEHPREVNILICKTEDALSPGNQNFENLVRNGNTRVDGSSPRQCLLRLSGCNGGGMNQGISNTSSEVVQFP
QLSCISSIISLSSGSNAKQDLRNMDPMSWTNFNRKNMPCPANYDSGKISLLPLSCSNEMVGSASPMKILSARDRALGGDLQSGIR
KHRFGMCDTRNHRMCLYHIRNHQSNLSEMGSNQNLRLDGRSWSGRCCPGLRPNPNIAKQGQSMRSYHPNYLKPLSCTHTHTL
QGLMRMMDSSLNSHSCSHDLQYANENIRDQGILASEYGSPNIEECKFAYHGAYAGMGNPPLLFRNAVENPLKDGFLTDSGSGMH
EVPHQNPYQNCHRVLTNCESGCYDSRQPFSLSPQKVDNISAFLNYPGYSQGTELRCNSKLSDREAGIESLSTHRDGAHTLQGGVA
SPVDLSLGNLSLSSSKEVEPLALSSHVDIDVSEALLKSQSKHLDLIDGHSSPEAYNSNGMESGSLTGNATKLGNDYVHKEEIQLDP
SSDLSIDEKRDLSFLYFLQTIKNTDLEYIKELGSGTYGTVSYGKWKGSDVAIKRIKPSCFTEDTLEEDRLVAEFWKEAHILGQLHHP
NIVAFYGVVTDGPVTNLATVTEYMVNGSLKQVLQKKDRTIDHRKRLIIAMDAAFGMEYLHGKNIVHFDLKSHNLFMNMRDPQR
PVCKIGDLGLSKIKQRTLISGGLRGTIPWMAPELFNSKNDLVTEKVDVYSFGIAMWELLTGEEPYGNKIWVFFFSWYNQRQSATQ
NSNL

>VviMAPKKK51

MAFDQNSIPLDLRPLNVPRTMVEDPRIAPATTTGRTTEGVFPNPARDAGSPGSVQMFYPATVSDAGLVGLGFGNAVPGVAAWCPH
VPVAIGRAGISPGAIGLGYNPNLGTRVAGNASDQASDEGTDDSNSGKKVKFLCSFGGKILPRPSDGMLRYVGGHTRIICLRRDVSF
NELVQKMVDTYGQPVVIKYQLPEEDLDALVSVSCPDDLENMMDEYEKLVERSSDGSAKLRVFLFSASELDPSDMVQFGNFNDS

GQRYFDAVNGIMDGIGGGIARKESIASATSTQNSDVSGNDATDNLVQHQGDVSGPPFSSALSPKGNSATSNEPATRLMCVDPNPAI
YADVSAIPLGIPVGNTGPPQTSSSKPDVEFERSVPLTVQPQQVGFDLQQCRMDIPATTAYLQSYVHPHREVTNHADYVQVPHQMG
FPNQLLATSGSVLTHQQIRDNASGVSSHQFIPAVHMTMTPTASHVSIRPSVIQPLVQPQQARIDCYTDESTFGPRVVQLPLDQSYNP
YQAQVPLPPAVVGGYGWHQVPAQDHVVLSDGWAHQQVILPETTTRLEDCFMCQKELPHAHSDPLVQGLRDSSASSVSDSNSAY
HSLRLEDNVRARQINRVVVTGALGEGIIEQGVGAQPRVLGHMDHQAGTLQSEVVGICQNLDAQHENEKIILQKMDNPDQPRVPI
PQGVVGLAGAVQSSYGVFTGTIPQTSQEEAVQQYAVPTQYQVKPDTLVNRPINSDVPLFGGVPLQTSERLVQESPRDYSGKLPGV
VPKEDTAESCISFDHMRPIDERMENLRVGPAENFVNSEQSKSSADKPRKEDILEHRLQQIAGKEVLLDSTFSKAKIVVESNHNKAT
EVLPCSAAEVPYLHNVWPVETYEVTKLPILGTLATYTHSKTGIHNVTSGEVSYGSPAFSDVESAYLTDKAPPISEWNDDTSQFQPK
MVPTDIRVVSSNGNTPYLSPSNRIGDVQDSSNSLFSSQDPWNLRHDIHFPPPRPNKITIKNEAFSIREPFGENGTSDSGDINTDVQLE
DGAHQPFSNLDKDFNSEHSWSAKGSGEEVIKQELQAIAEGVAASVLHSTTSNPEISIHEKNEPLSLSNKDIELQDSDLEMQHKSKV
EDNINKVPEKINMGFPVSDGIGRLQIIKNSDLEELRELGSGTFGTVYHGKWRGTDVAIKRINDRCFAGKPSEQERMRDDFWNEAI
KLADLHHPNVVAFYGVVLDGPGGSVATVTEYMVNGSLRNSLQKNEKNLDKRKRLLIAMDVAFGMEYLHGKNIVHFDLKSDNL
LVNLRDPHRPICKVGDLGLSKVKCQTLISGGVRGTLPWMAPELLNGSSSLVSEKVDVFSFGIVMWELLTGEEPYADLHYGAIIGGI
VSNTLRPSVPEFCDPEWRALMERCWSSEPSERPSFTEIANQLRSMAAKIPPKGQISQPQVQK
>VviMAPKKK52
MEPGNGKFYPESQGFMLDPTTAINTDTRPPEFNNLEVKPVRNYSIQTGEEFALEFMLDRVNPRNQFIPDTAGDPHYVPKYTELKGI
LGINHTGSESGSDISMLTIVERGPKEFERKNSALYEDRSYYGSVQLVPRTSSGHDSSRGVIHGYASSGASDSSSTKMKVLCSFGGKI
LPRPSDGKLRYVGGETRIIRIRKDISWQELVQKTLMVFNQAHIIKYQLPGEDLDALVSVSCDEDLQNMMEECNELEDGEGSKKLR
MFLFSTSDLDDAYFGLDSTDGDSEIQYVVAVNGMDMGSRKNSTLHGLVGSSSNNLADLDGQNIERNATRVATDSVGISTLPLTGTI
VPPSTIQSSQPILPNSSSAYEADPPFYHGQMIYHGETSQHMLHYGYPSHQSNCTPYQESTNLMPVHGLMTQQEGYAEGQPYIGLQ
VQDPSVLVKEVTLKNDASIQQENIPENISPSKNDCLIPSQPSDGEVMDRIPVEEALVSISSLDQFPSENKGKHHKPVEISSSVDAMN
QAQVPKSDYDHHPASSSPFAPVYADPGSGLMDLSYLEPPVLPQRVYYSERVPREQAELLNRLSKSDDSLGSQFLISHSRSDIEKQD
SVAESTDKLRNGNLAPQTEQSISTGEAMVEDMAVKADHGTTGTKDIPRKLLLHGTTEPGSELPAMNQVASVKHWSSVGVSTPEQ
GDILIDINDRFPRDFLSDIFSKAVHFADSPDISKPQKDGAGLSLNMENREPKHWSYFQKLAQGGFVQNDVSLMDQDHLGFSSVLT
KVEEEVSKPYQFTPLMADEVLIGQLESRISFGEENQKESPPGRIAADSTDLHSDYSPSEIKESDSVQFDRMIENLRTPDSEGEDGK
METKNIGRPPLDPSIGDFDINTLQIIKNEDLEELKELGSGTFGTVYHGKWRGSDVAIKRIKKICFTSRSSEQERLTIEFWREADILSK
LHHPNVVAFYGVVHDGPGATLATVTEYMVDGSLRHVLLRKDRYLDRRKRLLIAMDAAFGMEYLHSKNIVHFDLKCDNLLVNL
KDPLRPICKVGDFGLSKIKRNTLVSGGVRGTLPWMAPELLNGSSNKVSEKVDVFSFGIVLWEILTGEEPYANMHYGAIIGGIVSNT
LRPTVPSSCDPEWRTLMEQCWAPNPAVRPSFTEITGRLRVMSAAAQTKTPGHKASK
>VviMAPKKK53
MERNLGREMEQQKNYEQVRYNIVEARNEGLGSANQRFLHDPSSTINTNMRPPDFNITVAARPVLNYSIQTGEEFALEFMNPRQH
FVPSASGDPNSATNYAVLKGFLGASHTGSESGPDIPMLTSVEKSRVQEFERKSSSVHEDKGYYDSVRSVPRISSRNDSSRGLHGYT
SSGASERSSTKFKFLCSFGGKILPRPSDGKLRYVGGETRIIRMNKDISWQDLMQKTMTIYNQSHTIKYQLPGEDLDALVSVSCDED
LQNMMEECNVLEDGGSQKLRLFLFSSSDFDDGQFGLGSMEGDSEIQYVVAVNGMDLESRKNSIGLASTSDNNLDELLNLNVERE
TGRVATELPGPSTAPSTVNVHSSAVQSSQPLVPNFSGAYESNSKPYQGQKMRHGEAEQHQVKSGSYASPWKMNEPEKNRSLEKE
ASVKEAKIKTDSSVQKMNELEKIRSLESEHNVSSHPHDGSVPNYIPRDEASVVNSTADIGVPMLLPKTSKKHLESVQISKPPEAVS
DGKINTFNGDGHFHTSGGAFSPGYGDSEADPTEVSYPEQTLIPPRVFHSERIPREQAELNRLSKSDDSFGSQFLMSHTRSDVSQQV
AESIDKLHGGNVTSQSEQAASSTTALYTNPKTVEDGLTQFEKYKDVADDIKKLNSNISEDGLGPKLLKSESKWPAPTSVDDHEIA
GVRDGNKDPAVSDREAAGLNNLTASQGTSSKPHDDSPSKPTGFHWDEMANPLRSVPGGESSVGVGAPEGGDILIDINDRFPRDFL
SDIFSKARTSEGPPGISPLHGDGTGLSLNLENHEPKHWSFFQKLAQEEFIRKGVSLMDQDHLGYPSSLMNIEEGTPIDYSFPPLKSD
GVALGPMDSRINFEEEIQQESSSMVRPNTIDMHEDYDPSPVKRDESVQMDGMANPRTPDSDYEEVKFEIQNTGAPFVDPSLGDID
ISTLQIIKNEDLEELRELGSGTFGTVYHGKWRGTDVAIKRIKKSCFTGRSSEQERLTVEFWREADILSKLHHPNVVAFYGVVQDGP
GGTLATVTEFMVNGSLRHVLVSKDRHLDRRKRLIIAMDAAFGMEYLHSKNIVHFDLKCDNLLVNLKDPLRPICKVGDFGLSKIK
RNTLVTGGVRGTLPWMAPELLNGSSSRVSEKVDVFSFGIVLWEILTGEEPYAHMHYGAIIGGIVNNTLRPPVPSYCDSEWKLLME

QCWAPDPIGRPSFTEIARRLRAMSAACQTKPQGYSAGPAHNPVTK

>VviMAPKKK54

MTGETSGFSGQHFCNNPDNAVSSGQLAADRNAHDICAQTGEEFSAEFLRDRVAPRRASAMIDTDQRQPKGWCKGFNENHQMV
YEPLNGILGLRRGDSECSSDILDFVPGKGYAAEVENRVYLDKASRIHREYSAPRLGSGQLFEDFNCDQAVPGHATPSFNIPESPQP
HHCQGSGVSDASFSDKMKFLCSFGGRILPRPNDGKLRYVGGETKIISIRKNLSWVELVKKTSAICNQLHTIKYQLPGEDLDALISV
SSDEDLHHMIEEYHELERIEGSQRLRIFLVPVGEPESPSSFETRATQQNEADYQYVVAVNGMLDPSPRKNSSGQSVSSQTGNTCDY
RDPPFFHPLEMKDGASSSNLVGMFTNPAAQFLTSLQIPTKSFQQSPPVSPLPVQNRDPQNSAMHFFEDHAYHDGHESASQFVTDQ
WPCDNAYCVDSPSYYHNNPYGPVPLMNYHHHNKHFLETDQINKLPSLHVQNRPSRDFVFSPVLGQSEVDFERPVLKERAFHSHP
KDPLGLLSGSTNDLVGSHHRMLHVLSDSQLRGHEGRPDYHLEEGIIPLSPWTFEVQKSPSLALSNSPQEWSFQPQEISNEKYQEA
YQNQPTLIVDDHKGNNGLGQDTWNWEDEIDTQVGQERKHDKVITDLTSQDNSTLPNTKLQNVCYNPNSVPSIHISPLEFQDHGD
TTMNSASTLMIPENSADIVREQPHDYSLGASTPKFLVKSQNATKDMQHAMTEVISSESVPNESSRPLSVAIQGTGDQEAAVPSSAS
LTPSAGNKSDPSLNLQKNYPLSTESSFENPDKKAVMSGVSTLKDEDPLNFPCHEVDGPEGHFYERLNPGDAIFVQSQPSDNHHNG
NTPGAAVIVEDVTDILPPGIPSSSPLIPQVEDEASDVITSSGEAEAESDIQESEGEEGRDLGESISDAAMAEMEASIYGLQIIKNADLE
ELKELGSGTFGTVYHGKWRGTDVAIKRIKKSCFAGRSSEQERLTKDFWREARILSNLHHPNVVAFYGVVPDGPGGTLATVTEYM
VNGSLRHVLLRKDRSLDRRKRLIIAMDAAFGMEYLHLKNIVHFDLKCDNLLVNMRDTQRPICKVGDFGLSRIKRNTLVSGGVRG
TLPWMAPELLNGSSNRVSEKVDVFSFGVAMWEILTGEEPYANMHCGAIIGGIVSNTLRPPIPERCDPDWRKLMEECWSPDPAARP
SFTEITNRLRVMSMAIQTKRHNQANR

>VviMAPKKK55

MPHRTTYFFPRQFPDRRFDASSKELLAHEKKIGGESNRKGTRTTKDVTADRTYNASDLFTGSDKFRSKKQLAAFCDWLVEKKGD
RSGHVRLRSRNDEGDRDVLLPPPPAPVPEVVAGKDQQFDRQVSLPRVSSGSSYAGSLFSGTTVEGNVSSGLKDSHTNSHSQESTR
REVDEEKESAAQKSRESYYLQLTLAKRLASQASLACEPVLFLQESGAEGNAVSFDPDVVSYRLWVSGCLSYTDKISDGFYNILG
MNPYVWVMCNELEEGRRLPPLMALKAVEPNDTSMEVVLVDRRGDSRLKELEDKAHQLYCASENTLVLVEQLGKLVAIYMGGS
FPVEQGDLHKQWKLVSKRLRDFQKCIVLPIGSLSMGLCRHRAILFKKLADYIGLPCRIARGCKYCVADHRSSCLVKIDDKQSSRE
YVVDLVGEPGNVHGPDSSITGGLLSSMPSPLQISHLKEYQQPYMDNESCCQIQNSKNTCIYPEDPLYLGNEKNTLYTPTDQICERM
ESSVLPLEFNGNTDRCIIQSAMLQSVQSNVSEAVDASASGVSIHECFRIAGEKIVIQQAHKKEIALSGSPITSKALKQPKVSLSSKSN
IKEVEGRLENRGRFHTVTIPRYLNLEPSLAMDWLEISWDELHIKERVGAGSFGTVHRAEWHGSDVAVKVLTVQNFQDDQLKEFL
REVAIMKRVRHPNVVLFMGAVTKRPHLSIVTEYLPRGSLYRLIHRPTSAEILDQRRRLRMALDVAKGINYLHCLKPPIVHWDLKS
PNLLVDKNWTVKVCDFGLSRFKANTFLSSKSVAGTPEWMAPEFLRGEPSNEKSDVYSFGVILWELVTMQQPWNGLSPAQVVGA
VAFQNRRLSIPQNTSPVLASLMESCWADDPAQRPSFSSIVETLKKLLKSPLQLIQMGGT

>VviMAPKKK56

MEPHNRHVQEQIHSTSNRQLAYGREHWSSPKDTTCSKSWAQQTEESYQLQLALALRLSSDSSSAADPYFLDSATGDRPIGSARDL
SHRFWVNGCLSYIDRVPDGFYLIHGMDPYVWTISTDLKETGRIPSFESLKAVDPRDDFSIEVVLVDKHRDPSLKELQNRALSHSSS
WIKAKQVVDELANLVCNHMGGAASSGEDGFANHWKEFSGMLKNSLGSVVLPIGSLSVGLCVHRALLFKVLADVVNLPCRIAK
GCKYCRSNVASSCLVRFGPEREYLVDLMCNPGALCSPDSLLNGTSSILVSSPLCHPRFKLVETAEDFRILARLYFFDCQSLNIAFDD
PSSGAAVGQDDNSDSRFPKPFDRSYTESKNLVSTSNNHHELFLPQRTARLVSHDRDPQMQNSFNPLPNVINSKHLVKGAVRPSHIL
PMGNRDVQPILPFPRPRPGTNKNLGFMEKNHSVTSRSSLKYSLVEEDLEIPWSELVLKENIGAGSFGTVHRAKWRDSDVAVKILM
EQDFHAERFEEFLREVAIMKRLRHPNIVLFMGAVTQPPHLSIVTEYLSRGSLYKLLRMPDAGMVLDERRRLNMAYDVAMGMNY
LHQLKPPIVHRDLKSPNLLVDGNYTVKVCDFGLSRSKANTFLSSKTAAGTPEWMAPEVLRDEPSNEKSDVYSFGVILWELVTLQ
RPWKHLNPAQVVAAVAFKGKRLEIPAEVNHQVAYLIEACWANEPSKRPPFSFIKEYLQPLISSSPQPFQGSHSRNYPKGG

>VviMAPKKK57

MPGKRSNYSLLSQFPDDQFVGGAAGNQPPLYESLSGEKSKGKGFDWDGGDLRNRIGNLFTTSIGLQRQSSGSSFGESTLSGEYYV
PTMSMAASSDFDAFGDVFKVGGGGGAELRAKAVTGTGDSSSSKSWAQQTEESYQLQLALALRLSSEATCADDPNFLDPVPDDS
ASRSLSSSGSSVEAMSHRFWVSGCLSYFDKVPDGFYLIHGMDPYVWTVCNDLRENGRIPSIESLKHAEPSADSPIEVVLIDRRTDP
TLKELQNKVHGISCSCMTTKEVVDQLAKLVCNCMGGAASTGEDDFVSIWRECSDDQKDCLGSIVVPIGSLSFGLCRHRALLFKV

LADTIDLRCRIAKGCKYCTRDDASSCLVRVGPDREFLVDLVGKPGCLCEPDSLLNGPASISISSPLRFPRSKPVETNIDFRSLAKQYF
SECQSLNLVFEDTSVGVIVDEADGGDSMYPKKFDRKCTDRTHLVPISRNRGETPQLPMPPKVAWPSAHDQDSQLFKSCNPYQSSI
SPTDAVKDPIPPKRIPLTGHGDVQPSLALSDLRGDTIKDMRFTDGGQLYPNKPCKELSLDVEDLDIPWSDLVLKERIGAGSFGTVH
RADWNGSDVAVKVLMEQDFHAERFKEFLREVSIMKRLRHPNIVLFMGAVTQPPNLSIVTEYLSRGSLYRLLHKPGAREMLDERR
RLSMAYDVAKGMNYLHKRNPPIVHRDLKSPNLLVDKKYTVKVCDFGLSRFKANTFLSSKSAAGTPEWMAPEVLRDEASNEKSD
IYSFGIILWELATLQQPWSNLNPAQVVAAVGFKGKRLEIPRDLNPQVASIIEACWANEPWKRPSFFNIMESLKPLIKPPTPQPVRAD
RPLLT
>VviMAPKKK58
MKNILKKLHIVSNQTEDVEGSTSSRGSKTHDGSSPDRLLHSRPHHNSEHKPFSGLSNWLNSVANRHSPSPPLSSNVTRVERSEPSD
SMSSCGLDVVSDAVRRDSGSSNSRDPDIEEEYQIQLALELSAREDPEAVQIEAVKQISLGSCAPENTPAEIVAYRYWNYNALSYDD
KILDGFYDLYGILMESTSQKMPSLVDLQGTPLSDCVTWEAVLVNRAADANLLKLEQEALVMAVKSRSESPVFVGSDLVQRLAAL
VAANMGGPVGDPVNMSRAWQSLSYSLKATLGSMVLPLGSLTIGLARHRALLFKVLADSVGIPCRLVKGQQYTGSDDVAMNFVK
IEDGREYIVDLMADPGTLIPSDAAGSHIEYDDSIFSASTLSREIDSSYIASSSSGVVRPYLSAVGNESDDRGELTACANLPRPSKDSL
NAEQTLLRALPSRPSHPYMHGRSPSWTEGVSSPAVRRMKVKDVSQYMIDAAKENPQLAQKLHDVLLESGVVAPPNLFTEIYPEH
IDVSIVEAKSPTEDKDENEKRPVIRKIKDQDDLGPIGFLPPLPYHGMQPRVSPCVQPDLKPVEGLGFNNLLDFKEVTGQSVSSQSE
VNPVKYVKNVPVAAAAAAAAVVASSMVVAAAKSTADPNLELPVAAAATAAAAVVATTAAVGKQYENLETGVHSPSGAAECFN
QTDGMQSGGDADGAGYEPHGSGNREHDASGTNPEGERTSDRSADSTKSDVALDDVADCEIPWDEIALGERIGLGSYGEVYRGD
WHGTEVAVKKFLDQDISGESLDEFRSEVRIMKRLRHPNVVLFMGAVTRVPNLSIVTEFLPRGSLYRLIHRPNNQLDERRRLRMAL
DAARGMNYLHNCTPVIVHRDLKSPNLLVDKNWVVKVCDFGLSRMKHSTFLSSRSTAGTAEWMAPEVLRNEPSDEKCDVFSFG
VILWELSTLQQPWGGMNPMQVVGAVGFQHRRLDIPDDMDPVVADIIRRCWHTNPKMRPTFAEIMATLKPLQKPITSSQVPRPSA
AISSGQERVQPSRAAEEPAE
>VviMAPKKK59
MSRMKHLLRKLHIGGSLNEHQRIPETRPVINPSPSPNQSSPVAAAAPSSALGSVGGGDAVDRAAVDSQDAAVDFSFLEEEFQVQL
ALAISASDPDARDDRETAQIKVAKRISLGCSPSTTDTETLVELLSLRYWNYNAVNYDEKVMDGFYDVYGITANSVVQGKMPLLV
DLQAISVLDNVDYEVILVDRMIDPDLRELEDKAYSLSMEYQVSDQLTILDGLVQKIADMVVERMGGPVGDADEMLKRWTIRSY
ELRSSLNTIILPLGRLDIGLSRHRALLFKVLADRINLPCLLVKGSYYTGTDDGAINLIKIDNGSEYIIDLMGAPGALIPAEVPSSHHQ
NFGLDVRSCTDVIEAARESLLVPEKGTGFSPNLDVVSKPGSSKSEEAPFIGIRSKGDDRSPVEKFETERFENEFGNLLPSLRKLCEG
SSGTCGKASPAQKMKVKDVSKYVISAAKNPEFAQKLHAVLLESGASPPPDLFSDINSRGQVEQKVLEQIHMAKGKQVDHGVWY
SPGEFLLNSEQPLMPSHQVETNVTNSDFSLPSDTTSEGFILIGAGANGMIRTNATGETCQRQPENALVSDGGPCFQDNIGRILSNIG
TEKESALGLMETANGALHIPSNAHSEQINPMLAEVAEWEIPWEDLQIGERIGIGSYGEVYRADWNGTEVAVKKFLAQDFSGDAL
VQFRYEVEIMLRLRHPNVVLFMGAVTRPPNLSILTEFLPRGSLYRLLHRSNIQLDEKRRLRMALDVAKGMNYLHTSHPTIVHRDL
KSPNLLVDKNWVVKVCDFGLSRLKHHTFLSSKSTAGTPEWMAPEVLRNEPSNEKCDVYSFGVILWELATLRIPWSGMNPMQVV
GAVGFQDRRLEIPEEVDPMVAQIINDCWEVEPRKRPSFSQLMSRLKHLQHLVFERASSSRQAQVQ
>VviMAPKKK60
MKHIFKKLHIGSSSHDPNRSNETLSSATTSSPACASDHRTSSAQSSVSPPSSYPSPTTVSSTAASTLPTATSPAASNRSDYFLSEEEFQ
VQLALAISASNSDFRDDSEKDQIRAATLLSLGRHRTDSVRDKDESAESMSRRYWDYNVLDYEEKVVDGFYDVYGLSTDPVIQG
KMPSLTDLETNLGNSGFEVIVVNRRIDPALEELVQVAHCIALDCPAAEVGVLVQRLAEIVTDHMGGPVRDANIMLVKWMESRKD
LRTSLHTSILPVGSLSIGLSRHRALLFKILADNVGVPCRLVKGSHYTGVEDDAVNIIKLDNEREFLVDLMGAPGTLIPADILSAKDSS
LKSYNPKLSKIPTLQASKDPGGVYSRPKPLLGDYEGSSQTSTIENSLPQDRKASSEKIESLDSFSSSSGDTGVGTSRISKRVTPVNQS
DLRPSLAIGASVYKGSRGANAVGDGSRMNVNIVPYNQNSTEDPKNLFADLNPFQMIGSSKASAQSKPMENKVDEFQREKNSAA
PGRPPLPLMWKNRYANNEVPRKKENDFVEGLFPKINRETNDYNLPSLTSNNATTSEKVYSGVFKLSGNAYMNNKVNDDQNSSC
NTTSMLAPSTSQFNRLSLDEDVNANYNEKYHKDGKVFQSDMVDAAKEHDKNETGLHDHRKFRHDSFMENNLREAESPCSSVD
SDAGKVDQMFEDVGECEIPWEDLVLGERIGLGSYGEVYHGDWNGTEVAVKKFLDQDFSGAALAEFKREVRIMRRLRHPNVVLF
MGAVTRPPNLSIITEFLPRGSLYRILHRPSCQIDEKRRIKMALDVAKGMNCLHTSLPTIVHRDLKSPNLLVDKNWNVKVCDFGLSR

LKHNTFLSSKSTAGTPEWMAPEVLRNENSNEKCDVYSFGIILWELATLRLPWSGMNPMQVVGAVGFQNRRLDIPKEVDPLVARII
WECWQTDPNLRPSFAQLTVALKPLQRLVIPQHLDQQSLTLQQEISDEVRLCD
>VviMAPKKK61
MDTPPAEELLRKIQELEAGHAHLKEEMSKLMVSGPKSDHGHQRSHSTSPQRSRFSSPATSSWRKGSGGFDGPAGWRRGSASFRH
SSPLQRESRSRDPSSTAGGGGGPAAYKFNDKQYSNILQSMGQSIHIFDLNGRVIYWNRTAEKLYGYSAEEALGQQAIELLSDVQDY
AIANNIVDRVSRGESWTGQFPVKNKMGERFLAVATNTPFYDDDGTLIGIICVSSDSQPFQEIRVAMSNERQSEANASYNRSRSSSA
SAKLGLDPQQPIQAAIASKISNLASKVSNKVRKIKAGENNVVREGGSGDSHHSDHGFSDAAFSDHREDATSSGASTPRGDVAPSP
FGIFSQATADEKSPGKNLRDSGDENEGKPGIHRVITSKAEAWIGKKVMSWPWKGNEREGSEVKTNRFGWPWLQNDHENDMVQ
PKNPNFGAKTENLVSESNRHGNNDASGSWSSFNVNSTSSVSSCGSTSSSAVNKVDMETDCLDYEILWEDLTIGEQIGQGSCGTVY
HGLWYGSDVAIKVFSKQEYSDDVILSFRQEVSLMKRLRHPNVLLFMGAVTSPQRLCIVTEFLPRGSLFRLLQRNTSRLDWRRRVH
MALDIAQGMNYLHHFNPPIIHRDLKSSNLLVDRNWTVKVGDFGLSRLKHETYLTTKTGKGTPQWMAPEVLRNEPSDEKSDVYS
YGVILWELATEKIPWDNLNTMQVIGAVGFMNQRLDIPKEVDLRWASIIESCWHSDPRSRPTFQELLGKFKDILRQQTMQFQAARA
AAGDNTQKEL
>VviMAPKKK62
MDLIEGVGESSSPPRSFGCFGVYDVRNDVYNRLMESGNEEAVSNPEFREQLDAHFNRLPPSYGLDVNIDRVDDVLLHQKLLALA
KEPDKRPVYHVRFLENLSTKVDGNDDQQSMSVLSTARPCCNADNEGVVPSHNRNEIDFEPCSKLEDLNLDVKKDSTDMERRCL
MENSSRRQETSNVPIHEVIFSTIDKPKLLSQLSALLSDIGLNIREAHVFSTIDGYSLDVFVVDGWPVEDTDGLSEAMEKAIARSEGS
WSGSSHSHSAVEKALAAQVKSGDWEIDRRLLKIGERIASGSCGDLYRGVYLGQDVAVKILRSEHLNESLEDEFEQEVAILREVQH
RNVVRFIGACTRSPHLCIVTEYMPGGSLYDYLHKNHNVLKLPQLLKFAIDVCKGMGYLHQNNIIHRDLKTANLLMDTHNVVKV
ADFGVARFQNQEGVMTAETGTYRWMAPEVINHLPYDQKADVFSFAIVLWELTTAKIPYDNMTPLQAALGVRQGLRPDLPENTH
PKLVDMMQRCWEAVPGNRPSFSEITVELEELLQEVQGTSRASNGN
>VviMAPKKK63
MVMEDNESCSSRVHDSSSPAQSRQQRQKLEVYNEVLRRLKDSDNEEAFEPGFDEELWAHFVRLPTRYALDVNVERAEDVLTHK
RLLHLAHDPTNRPAIEVRLVQVHPISDGIHGNIADSIHSNSPTIGPAHGSPKYSSKQSILPPPAFGSSPNLEALAIEANNSHVQDGDG
DDSVHASSQYSRPMHEITFSSDDKPKLLSQLTCLLSELELNIQEAHAFSTVDGYSLDVFVVDGWPYEETEQLRTALEKEVFKIEKQ
SWPNHHSLSPTGEQEETGIKCESDFVTIPNDGTDVWEIDVRQLKFENKVASGSYGDLYKGTYCSQEVAIKVLKPERLNSDMQKEF
AQEVFIMRKVRHKNVVQFIGACTRPPSLYIVTEFMSGGSVYDYLHKQKGVFKLPALLKVSIDVSKGMNYLHQNNIIHRDLKAAN
LLMDENEVVKVADFGVARVKAQSGVMTAETGTYRWMAPEVIEHKPYDHKADVFSFGIVLWELLTGKLPYEYLTPLQAAVGVV
QKGLRPTMPKNTHPKLAELLERCWQQDPTLRPDFSEIIEILQQIAKEVGDEEDRRKEKSSSGFLSVLRRGHH
>VviMAPKKK64
MRPGFDDELWAHFSRLPTRYALDVNVERAEDVLTHKRLLQLAHDPATRPALEVRLVQVHPISGGIHGDSDPSNPLKKVDAQSHY
HTSQRSTHPPSTFGLLPNMKVLVEAKNSHVQDGEGDVDANLRFWRLMHEVIISTNDKPKLLSQLTSLLSDIGLNIQEAHAFSTTD
GYSLDVFVVEGWAHEETEQLRNVLLKEIQMIEKQPWSEFQLISPGREQGHPEKQLIPSHINLTIDGADVWEIDATLLKFENKIASG
SYGDLYKGTFCSQDVAIKVLKTQHLNEDMWREFSQEVYIMRKVRHKNIVQFIGACTRPPSLCIVTEFMFGGSVYDFLHKQKGSF
KLPSLLKVAIDVSKGMNYLHQNDIIHRDLKAANILMDENKVVKVADFGVARVQAQSGVMTAETGTYRWMAPEVIEHKPYDHK
ADVFSFGIVLWELLTGKLPYEHLTPLQAAVGVVQKGLRPTIPSHTYPSLVKLIKRCWHQEPSLRPEFTEIMEILQQIASKVVEKKRE
ILEGRGKRQKEKSI
>AtMEKK1
MQDFFGSVRRSLVFRPSSDDDNQENQPPFPGVLADKITSCIRKSKIFIKPSFSPPPPANTVDMAPPISWRKGQLIGRGAFGTVYMG
MNLDSGELLAVKQVLIAANFASKEKTQAHIQELEEEVKLLKNLSHPNIVRYLGTVREDDTLNILLEFVPGGSISSLLEKFGPFPESV
VRTYTRQLLLGLEYLHNHAIMHRDIKGANILVDNKGCIKLADFGASKQVAELATMTGAKSMKGTPYWMAPEVILQTGHSFSADI
WSVGCTVIEMVTGKAPWSQQYKEVAAIFFIGTTKSHPPIPDTLSSDAKDFLLKCLQEVPNLRPTASELLKHPFVMGKHKESASTD
LGSVLNNLSTPLPLQINNTKSTPDSTCDDVGDMCNFGSLNYSLVDPVKSIQNKNLWQQNDNGGDEDDMCLIDDENFLTFDGEMS
STLEKDCHLKKSCDDISDMSIALKSKFDESPGNGEKESTMSMECDQPSYSEDDDELTESKIKAFLDEKAADLKKLQTPLYEEFYN

SLITFSPSCMESNLSNSKREDTARGFLKLPPKSRSPSRGPLGGSPSRATDATSCSKSPGSGGSRELNINNGGDEASQDGVSARVTD
WRGLVVDTKQELSQCVALSEIEKKWKEELDQELERKRQEIMRQAGLGSSPRDRGMSRQREKSRFASPGK
>AtMEKK2
MVFAKSQSPPNNSTVQIKPPIRWRKGQLIGRGAFGTVYMGMNLDSGELLAVKQVLITSNCASKEKTQAHIQELEEEVKLLKNLS
HPNIVRYLGTVREDETLNILLEFVPGGSISSLLEKFGAFPESVVRTYTNQLLLGLEYLHNHAIMHRDIKGANILVDNQGCIKLADF
GASKQVAELATISGAKSMKGTPYWMAPEVILQTGHSFSADIWSVGCTVIEMVTGKAPWSQQYKEIAAIFHIGTTKSHPPIPDNISS
DANDFLLKCLQQEPNLRPTASELLKHPFVTGKQKESASKDLTSFMDNSCSPLPSELTNITSYQTSTSDDVGDICNLGSLTCTLAFPE
KSIQNNSLCLKSNNGYDDDDDNDMCLIDDENFLTYNGETGPSLDNNTDAKKSCDTMSEISDILKCKFDENSGNGETETKVSMEV
DHPSYSEDENELTESKIKAFLDDKAAELKKLQTPLYEEFYNGMITCSPICMESNINNNKREEAPRGFLKLPPKSRSPSQGHIGRSPS
RATDAACCSKSPESGNSSGAPKNSNASAGAEQESNSQSVALSEIERKWKEELDQELERKRREITRQAGMGSSPRDRSLSRHREKS
RFASPGK
>AtMEKK3
MPTWWGRKSCKNKDDNHRGIISTDRDIKSSAVVVDPPLTPTRGGTPRCSREFAGASSAFSGFDSDSTEKKGHPLPRPLLSPVSIHH
QDHVSGSTSGSTSVSSVSSSGSADDQSQLVASRGRGDVKFNVAAAPRSPERVSPKAATITTRPTSPRHQRLSGVVSLESSTGRNDD
GRSSSECHPLPRPPTSPTSPSAVHGSRIGGGYETSPSGFSTWKKGKFLGSGTFGQVYLGFNSEKGKMCAIKEVKVISDDQTSKECL
KQLNQEINLLNQLCHPNIVQYYGSELSEETLSVYLEYVSGGSIHKLLKDYGSFTEPVIQNYTRQILAGLAYLHGRNTVHRDIKGA
NILVDPNGEIKLADFGMAKHVTAFSTMLSFKGSPYWMAPEVVMSQNGYTHAVDIWSLGCTILEMATSKPPWSQFEGVAAIFKIG
NSKDTPEIPDHLSNDAKNFIRLCLQRNPTVRPTASQLLEHPFLRNTTRVASTSLPKDFPPRSYDGNFSLQPTREPYPGRLSHDNYAK
QPLSRTIKSPSRENVRAITSLPVSPCSSPLRQLGPAYKSCFLSPPHPSYAFPGQDSGYNLAEFAASPFRMKKDAMMEPSSFRTQTPN
SPLRSRLV
>AtMEKK4
MPWWSKSKDEKKKTNKESIIDAFNRKLGFASEDRSSGRSRKSRRRRDEIVSERGAISRLPSRSPSPSTRVSRCQSFAERSPAVPLPR
PIVRPHVTSTDSGMNGSQRPGLDANLKPSWLPLPKPHGATSIPDNTGAEPDFATASVSSGSSVGDIPSDSLLSPLASDCENGNRTPV
NISSRDQSMHSNKNSAEMFKPVPNKNRILSASPRRRPLGTHVKNLQIPQRDLVLCSAPDSLLSSPSRSPMRSFIPDQVSNHGLLISK
PYSDVSLLGSGQCSSPGSGYNSGNNSIGGDMATQLFWPQSRCSPECSPVPSPRMTSPGPSSRIQSGAVTPLHPRAGGSTTGSPTRR
LDDNRQQSHRLPLPPLLISNTCPFSPTYSAATSPSVPRSPARAEATVSPGSRWKKGRLLGMGSFGHVYLGFNSESGEMCAMKEVT
LCSDDPKSRESAQQLGQEISVLSRLRHQNIVQYYGSETVDDKLYIYLEYVSGGSIYKLLQEYGQFGENAIRNYTQQILSGLAYLHA
KNTVHRDIKGANILVDPHGRVKVADFGMAKHITAQSGPLSFKGSPYWMAPEVIKNSNGSNLAVDIWSLGCTVLEMATTKPPWSQ
YEGVPAMFKIGNSKELPDIPDHLSEEGKDFVRKCLQRNPANRPTAAQLLDHAFVRNVMPMERPIVSGEPAEAMNVASSTMRSLDI
GHARSLPCLDSEDATNYQQKGLKHGSGFSISQSPRNMSCPISPVGSPIFHSHSPHISGRRSPSPISSPHALSGSSTPLTGCGGAIPFHH
QRQTTVNFLHEGIGSSRSPGSGGNFYTNSFFQEPSRQQDRSRSSPRTPPHVFWDNNGSIQPGYNWNKDNQPVLSDHVSQQLLSEH
LKLKSLDLRPGFSTPGSTNRGP
>AtMEKK5
MRWLPQISFSSPSSSPSSSLKPVASYSESPDPDRNQDRDRFHRRLFRFNRGRLTRQRKLRHLTDDDVLLGERRASTSSSTFDSGLTR
SPSAFTAVPRSPSAVPLPLPLPLPEVAGIRNAANARGLDDRDRDPERLISDRTSSGPPLTSVNGGFARDSRKATENSSYQDFSPRNRN
GYWVNIPTMSAPTSPYMSPVPSPQRKSTGHDLPFFYLPPKSNQAWSAPDMPLDTSGLPPPAFYDITAFSTDNSPIHSPQPRSPRKQI
RSPQPSRPSSPLHSVDSSAPPRDSVSSPLHPRLSTDVTNGRRDCCNVHPLPLPPGATCSSSSAASVPSPQAPLKLDSFPMNSQWKK
GKLIGRGTFGSVYVASNSETGALCAMKEVELFPDDPKSAECIKQLEQEIKLLSNLQHPNIVQYFGSETVEDRFFIYLEYVHPGSIN
KYIRDHCGTMTESVVRNFTRHILSGLAYLHNKKTVHRDIKGANLLVDASGVVKLADFGMAKHLTGQRADLSLKGSPYWMAPE
LMQAVMQKDSNPDLAFAVDIWSLGCTIIEMFTGKPPWSEFEGAAAMFKVMRDSPPIPESMSPEGKDFLRLCFQRNPAERPTASML
LEHRFLKNSLQPTSPSNSDVSQLFNGMNITEPSSRREKPNFKLDQVPRARNMTSSESESGQQQQQQQYRSPDLTGTVNRLSPRST
LEAIPSPCPSQRPKPSSSDRRRTGVTSDHL
>AtMEKK6
MARQMTSSQFHKSKTLDNKYMLGDEIGKGAYGRVYIGLDLENGDFVAIKQVSLENIGQEDLNTIMQEIDLLKNLNHKNIVKYLG

SLKTKTHLHIILEYVENGSLANIIKPNKFGPFPESLVTVYIAQVLEGLVYLHEQGVIHRDIKGANILTTKEGLVKLADFGVATKLNE
ADFNTHSVVGTPYWMAPEVIELSGVCAASDIWSVGCTIIELLTCVPPYYDLQPMPALYRIVQDDTPPIPDSLSPDITDFLRLCFKKD
SRQRPDAKTLLSHPWIRNSRRALRSSLRHSGTIRYMKETDSSSEKDAEGSQEVVESVSAEKVEVTKTNSKSKLPVIGGASFRSEK
DQSSPSDLGEEGTDSEDDINSDQGPTLSMHDKSSRQSGTCSISSDAKGTSQDVLENHEKYDRDEIPGNLETEASEGRRNTLATKLV
GKEYSIQSSHSFSQKGEDGLRKAVKTPSSFGGNELTRFSDPPGDASLHDLFHPLDKVPEGKTNEASTSTPTANVNQGDSPVADGG
KNDLATKLRARIAQKQMEGETGHSQDGGDLFRLMMGVLKDDVLNIDDLVFDEKVPPENLFPLQAVEFSRLVSSLRPDESEDAIV
TSSLKLVAMFRQRPGQKAVFVTQNGFLPLMDLLDIPKSRVICAVLQLINEIVKDNTDFLENACLVGLIPLVMSFAGFERDRSREIRK
EAAYFLQQLCQSSPLTLQMFISCRGIPVLVGFLEADYAKHREMVHLAIDGMWQVFKLKKSTSRNDFCRIAAKNGILLRLVNTLYS
LSEATRLASISGDALILDGQTPRARSGQLDPNNPIFSQRETSPSVIDHPDGLKTRNGGGEEPSHALTSNSQSSDVHQPDALHPDGDR
PRLSSVVADATEDVIQQHRISLSANRTSTDKLQKLAEGASNGFPVTQPDQVRPLLSLLEKEPPSRKISGQLDYVKHIAGIERHESRL
PLLYASDEKKTNGDLEFIMAEFAEVSGRGKENGNLDTAPRYSSKTMTKKVMAIERVASTCGIASQTASGVLSGSGVLNARPGSTT
SSGLLAHALSADVSMDYLEKVADLLLEFARAETTVKSYMCSQSLLSRLFQMFNRVEPPILLKILECTNHLSTDPNCLENLQRADA
IKQLIPNLELKEGPLVYQIHHEVLSALFNLCKINKRRQEQAAENGIIPHLMLFVMSDSPLKQYALPLLCDMAHASRNSREQLRAH
GGLDVYLSLLDDEYWSVIALDSIAVCLAQDVDQKVEQAFLKKDAIQKLVNFFQNCPERHFVHILEPFLKIITKSSSINKTLALNGL
TPLLIARLDHQDAIARLNLLKLIKAVYEKHPKPKQLIVENDLPQKLQNLIEERRDGQRSGGQVLVKQMATSLLKALHINTIL

>AtMEKK7

MARQMTSSQFHKSKTLDNKYMLGDEIGKGAYGRVYKGLDLENGDFVAIKQVSLENIVQEDLNTIMQEIDLLKNLNHKNIVKYL
GSSKTKTHLHIILEYVENGSLANIIKPNKFGPFPESLVAVYIAQVLEGLVYLHEQGVIHRDIKGANILTTKEGLVKLADFGVATKLNE
ADVNTHSVVGTPYWMAPEVIEMSGVCAASDIWSVGCTVIELLTCVPPYYDLQPMPALFRIVQDDNPPIPDSLSPDITDFLRQCFK
KDSRQRPDAKTLLSHPWIRNSRRALQSSLRHSGTIKYMKEATASSEKDDEGSQDAAESLSGENVGISKTDSKSKLPLVGVSSFRSE
KDQSTPSDLGEEGTDNSEDDIMSDQVPTLSIHEKSSDAKGTPQDVSDFHGKSERGETPENLVTETSEARKNTSAIKHVGKELSIPV
DQTSHSFGRKGEERGIRKAVKTPSSVSGNELARFSDPPGDASLHDLFHPLDKVSEGKPNEASTSMPTSNVNQGDSPVADGGKND
LATKLRATIAQKQMEGETGHSNDGGDLFRLMMGVLKDDVIDIDGLVFDEKVPAENLFPLQAVEFSRLVSSLRPDESEDAIVSSCQ
KLVAMFRQRPEQKVVFVTQHGFLPLMDLLDIPKSRVICAVLQLINEIIKDNTDFQENACLVGLIPVVMSFAGPERDRSREIRKEAAY
FLQQLCQSSPLTLQMFIACRGIPVLVGFLEADYAKYREMVHLAIDGMWQVFKLKRSTPRNDFCRIAAKNGILLRLINTLYSLNEAT
RLASISGGLDGQAPRVRSGQLDPNNPIFGQNETSSLSMIDQPDVLKTRHGGGEEPSHASTSNSQRSDVHQPDALHPDGDKPRVSS
VAPDASTSGTEDVRQQHRISLSANRTSTDKLQKLAEGASNGFPVTQTEQVRPLLSLLDKEPPSRHYSGQLDYVKHITGIERHESRL
PLLHGSNEKKNNGDLDFLMAEFAEVSGRGKENGSLDTTTRYPSKTMTKKVLAIEGVASTSGIASQTASGVLSGSGVLNARPGSAT
SSGLLAHMVSTLSADVAREYLEKVADLLLEFARADTTVKSYMCSQSLLSRLFQMFNRVEPPILLKILECTNHLSTDPNCLENLQR
ADAIKHLIPNLELKDGHLVYQIHHEVLSALFNLCKINKRRQEQAAENGIIPHLMLFIMSDSPLKQYALPLLCDMAHASRNSREQLR
AHGGLDVYLSLLDDEYWSVIALDSIAVCLAQDNDNRKVEQALLKQDAIQKLVDFFQSCPERHFVHILEPFLKIITKSYRINKTLAV
NGLTPLLISRLDHQDAIARLNLLKLIKAVYEHHPRPKQLIVENDLPQKLQNLIEERRDGQRSGGQVLVKQMATSLLKALHINTIL

>AtMEKK8

MDRILARMKKSTGRRGGDKNITPVRRLERRDAARNINYDAASCSSSSAEDLSVSTSSLMTRSLEFPEPTSFRIGGGVGEMDRIYR
SLGVSGPDDLAISFDAWEACKKRSSSDVVNRFKSFDLDKVRDQDLSEEGPSGVVVGSDSMNHKVQGQDLSEAGPSGGIVTELSE
IGNLITPVDRLVADGVVENRRVMERTPTIVKSKGYLVPNNVVAVGVGVGGGIKGLRPPVLKPPPAMKRPPIDHRGSSWDFLTHFAP
SETVKRPSSSSSSSEDGCDEEEGKEEEAEAEEMGARFIQLGDTADETCSFTTNEGDSSSTVSNTSPIYPDGGAIITSWQKGQLLGRG
SFGSVYEGISGDGDFFAVKEVSLLDQGSQAQECIQQLEGEIKLLSQLQHQNIVRYRGTAKDGSNLYIFLELVTQGSLLKLYQRYQL
RDSVVSLYTRQILDGLKYLHDKGFIHRDIKCANILVDANGAVKLADFGLAKVSKFNDIKSCKGTPFWMAPEVINRKDSDGYGSP
ADIWSLGCTVLEMCTGQIPYSDLEPVQALFRIGRGTLPEVPDTLSLDARLFILKCLKVNPEERPTAAELLNHPFVRRPLPSVGSGG
SGSASPLLRR

>AtMEKK9

MKKSSDKSPVRQHDTATQINSDAVSSSTSFTDSDSTCSFLTPSMEFPDRISFRRIDFSEAAPTGVVLPSTSSELTRSNSSENKIPNEDIS
VSTSSRYLVFDKILALMKKSPGRRGDKTSPARRLDRSDAVRRNIDYDAGEDSSSLLITRSLDFPNRTSFRVDGVDDGEIDRIYQYIG

VSGPEDFAISSDAWKARMEHERSSSDVVNKLKSLDLDSREAGPSGGVVASSSMNHKFQGHDLSEAGSIGVVVASNFTLSESNKIE
NLNSLRDKEIVDGDMVENRCGIERKPTILVKSRGYLVHNDDVGVGGGIKGVRPPVLNVPRADKEVVDGGTVESKSGIEWKPTIL
VKSKGYLVSNDGGIKGVTSPVLNLRPTDKEVVDSGTVENRRGIKGVRPSVLKPPPVMKLPPVDLPGSSWDILTHFAPDSEIVRRPS
SSSSSENGCDEEEAEDDKVEKEETGDMFIQLEDTTDEACSFTTNEGDSSSTVSNTSPICVSGGSINTSWQKGQLLRQGSFGSVYEA
ISEDGDFFAVKEVSLLDQGSQAQECIQQLEGEIALLSQLEHQNILRYRGTDKDGSNLYIFLELVTQGSLLELYRRYQIRDSLISLYTK
QILDGLKYLHHKGFIHRDIKCATILVDANGTVKLADFGLAKVSKLNDIKSRKETLFWMAPEVINRKDNDGYRSPADIWSLGCTVL
EMCTGQIPYSDLEPVEALFRIRRGTLPEVPDTLSLDARHFILKCLKLNPEERPTATELLNHPFVRRPLPSSGSGSTSPLIRR
>AtMEKK10
MDVTAIFAGDILVQSREYLIPNDVVDVDGGIKAVRPPIIQPPPGRKLPLIDFPGSSWDFLTYFAPSKTVKRQSSSSSDNTSDKEEVET
EETRGMFVQLGDTAHEACPFATNEADSSSTVSIISPSYASRGSIVPSWLKRKFLGRVSLGFVYEGSSGSSVGSESTCSLMTPSLEFP
DRISFRKKDFSEKGPSRHVWEKRKLTRAKLIENFCNPEDIEPVTSWLKGQLLGEESFASVYEAISDSSVGSESTCSLMTPSMEFPD
RISFRKRDFSEEGPSGRVKEKRKLMRNKLIENFRKPEDITSWLKGQLLGRGSYASVYEAISEDGDFFAVKEVSLLDKGIQAQECIQ
QLEGEIALLSQLQHQNIVRYRGTAKDVSKLYIFLELVTQGSVQKLYERYQLSYTVVSLYTRQILAGLNYLHDKGFVHRDIKCANM
LVDANGTVKLADFGLAEASKFNDIMSCKGTLFWMAPEVINRKDSDGNGSPADIWSLGCTVLEMCTGQIPYSDLKPIQAAFKIGR
GTLPDVPDTLSLDARHFILTCLKVNPEERPTAAELLHHPFVINL
>AtMEKK11
MSEKEELPLTLTSIGAATATSDYHQRVGSSGEGISSSSSDVDPRFMQNSPTGLMISQSSSMCTVPPGMAATPPISSGSGLSQQLNNSS
SSKLCQVEGCQKGARDASGRCISHGGGRRCQKPDCQKGAEGKTVYCKAHGGGRRCEYLGCTKGAEGSTDFCIAHGGGRRCNH
EDCTRSAWGRTEFCVKHGGGARCKTYGCGKSASGPLPFCRAHGGGKKCSHEDCTGFARGRSGLCLMHGGGKRCQRENCTKSA
EGLSGLCISHGGGRRCQSIGCTKGAKGSKMFCKACITKRPLTIDGGGNMGGVTTGDALNYLKAVKDKFEDSEKYDTFLEVLNDC
KHQGVDTSGVIARLKDLFKGHDDLLLGFNTYLSKEYQITILPEDDFPIDFLDKVEGPYEMTYQQAQTVQANANMQPQTEYPSSS
AVQSFSSGQPQIPTSAPDSSLLAKSNTSGITIIEHMSQQPLNVDKQVNDGYNWQKYGQKKVKGSKFPLSYYKCTYLGCPSKRKV
ERSLDGQVAEIVYKDRHNHEPPNQGKDGSTTYLSGSSTHINCMSSELTASQFSSNKTKIEQQEAASLATTIEYMSEASDNEEDSNG
ETSEGEKDEDEPEPKRRITEVQVSELADASDRTVREPRVIFQTTSEVDNLDDGYRWRKYGQKVVKGNPYPRFSSSKDYDVVIRY
GRADISNEDFISHLRASLCRRGISVYEKFNEVDALPKCRVLIIVLTSTYVPSNLLNILEHQHTEDRVVYPIFYRLSPYDFVCNSKNYE
RFYLQDEPKKWQAALKEITQMPGYTLTDKSESELIDEIVRDALKVLCSADKVNMIGMDMQVEEILSLLCIESLDVRSIGIWGTVG
IGKTTIAEEIFRKISVQYETCVVLKDLHKEVEVKGHDAVRENFLSEVLEVEPHVIRISDIKTSFLRSRLQRKRILVILDDVNDYRDV
DTFLGTLNYFGPGSRIIMTSRNRRVFVLCKIDHVYEVKPLDIPKSLLLLDRGTCQIVLSPEVYKTLSLELVKFSNGNPQVLQFLSSI
DREWNKLSQEVKTTSPIYIPGIFEKSCCGLDDNERGIFLDIACFFNRIDKDNVAMLLDGCGFSAHVGFRGLVDKSLLTISQHNLVD
MLSFIQATGREIVRQESADRPGDRSRLWNADYIRHVFINDTGTSAIEGIFLDMLNLKFDANPNVFEKMCNLRLLKLYCSKAEEKH
GVSFPQGLEYLPSKLRLLHWEYYPLSSLPKSFNPENLVELNLPSSCAKKLWKGKKARFCTTNSSLEKLKKMRLSYSDQLTKIPRL
SSATNLEHIDLEGCNSLLSLSQSISYLKKLVFLNLKGCSKLENIPSMVDLESLEVLNLSGCSKLGNFPEISPNVKELYMGGTMIQEIP
SSIKNLVLLEKLDLENSRHLKNLPTSIYKLKHLETLNLSGCISLERFPDSSRRMKCLRFLDLSRTDIKELPSSISYLTALDELLFVDSR
RNSPVVTNPNANSTELMPSESSKLEILGTPADNEVVVGGTVEKTRGIERTPTILVKSREYLIPDDVVAVGGDIKGLRPPVLQLQPA
MKLSHIPRGSTWDFVTHFAPPETVAPPSSSSEAREEEVETEETGAMFIPLGDKETCSFTVNKGDSSRTISNTSPIYASEGSFITCWQK
GQLLGRGSLGSVYEGISADGDFFAFKEVSLLDQGSQAHEWIQQVEGGIALLSQLQHQNIVRYRGTTKDESNLYIFLELVTQGSLR
KLYQRNQLGDSVVSLYTRQILDGLKYLHDKGFIHRNIKCANVLVDANGTVKLADFGLAKVMSLWRTPYWNWMAPEVILNPKD
YDGYGTPADIWSLGCTVLEMLTGQIPYSDLEIGTALYNIGTGKLPKIPDILSLDARDFILTCLKVNPEERPTAAELLNHPFVNMPLPS
SGSGSVSSLLRG
>AtMEKK12
MQDILGSVRRSLVFRSSLAGDDGTSGGGLSGFVGKINSSIRSSRIGLFSKPPPGLPAPRKEEAPSIRWRKGELIGCGAFGRVYMGM
NLDSGELLAIKQVLIAPSSASKEKTQGHIRELEEEVQLLKNLSHPNIVRYLGTVRESDSLNILMEFVPGGSISSLLEKFGSFPEPVIIM
YTKQLLLGLEYLHNNGIMHRDIKGANILVDNKGCIRLADFGASKKVVELATVNGAKSMKGTPYWMAPEVILQTGHSFSADIWS
VGCTVIEMATGKPPWSEQYQQFAAVLHIGRTKAHPPIPEDLSPEAKDFLMKCLHKEPSLRLSATELLQHPFVTGKRQEPYPAYRNS

LTECGNPITTQGMNVRSSINSLIRRSTCSGLKDVCELGSLRSSIIYPQKSNNSGFGWRDGDSDDLCQTDMDDLCNIESVRNNVLSQ
STDLNKSFNPMCDSTDNWSCKFDESPKVMKSKSNLLSYQASQLQTGVPCDEETSLTFAGGSSVAEDDYKGTELKIKSFLDEKAQ
DLKRLQTPLLEEFHNAMNPGIPQGALGDTNIYNLPNLPSISKTPKRLPSRRLSAISDAMPSPLKSSKRTLNTSRVMQSGTEPTQVNE
STKKGVNNSRCFSEIRRKWEEELYEELERHRENLRHAGAGGKTPLSGHKG

>AtMEKK13

MEKQSIRNTCSSSLMLSSPSSFWVRGACIGRGCFGAVSTAISKTNGEVFAVKSVDLATSLPTQSESLENEISVFRSLKPHPYIVKFLG
DGVSKEGTTTFRNLYLEYLPNGDVASHRAGGKIEDETLLQRYTACLVSALRHVHSQGFVHCDVKARNILVSQSSMVKLADFGSA
FRIHTPRALITPRGSPLWMAPEVIRREYQGPESDVWSLGCTIIEMFTGKPAWEDHGIDSLSRISFSDELPVFPSKLSEIGRDFLEKCL
KRDPNQRWSCDQLLQHPFLSQCHNSSPTESSPRCVLDWVNSGFDLEEEEEEVGRSEFEDAAKAIICNLATTGGVIWESDGWVEV
RCHASEEEGTTMEYSGSTRVESEYNTSSDPNDDVAGDSAIIDVSMSQNLPPGNGGSAAALPYEFVVVLHLLMEIMVYTTCIFREI
VLTMYLLYQYNQSNKLETLSFNHSLKFCLFAHVIRIGQNYLLRGEMRSSVLITSHCLILITLVYSQIVVF

>AtMEKK14

MEKQNIISNTSSSSSSWIRGSCVGRGCFGTVSKALSKIDGGLFAVKSIDLATCLPSQAESLENEIVILRSMKSHPNIVRFLGDDVSKE
GTASFRNLHLEYSPEGDVANGGIVNETLLRRYVWCLVSALSHVHSNGIVHCDVKSKNVLVFNGGSSVKLADFGSAVEFEKSTIHV
SPRGSPLWMAPEVVRREYQGPESDVWSLGCTVIEMLTGKPAWEDHGFDSLSRIGFSNDLPFIPVGLSELGRDFLEKCLKRDRSQR
WSCDQLLQHPFLCQDHHDSFFTESSPRCVLDWVNSEFDEEEESDEWRPESMVSAMARISKLAITGGANWESNGWTEVRDTSEE
SEAKKEVLVSPRVELESYISLESSSDDSVRQPRNEESATELASAVTCEAILLVMILVVENIQIYATFYTSSIIMHILYCCSCCCYYHYQ
NNNKKNNFSKSTSFILSLNFLFGIACDSDRSIY

>AtMEKK15

MEEQNWIRGPIIGRGSTATVSLGITNSGDFFAVKSAEFSSSAFLQREQSILSKLSSPYIVKYIGSNVTKENDKLMYNLLMEYVSGGS
LHDLIKNSGGKLPEPLIRSYTRQILKGLMYLHDQGIVHCDVKSQNVMIGGEIAKIVDLGCAKTVEENENLEFSGTPAFMSPEVAR
GEEQSFPADVWALGCTVIEMATGSSPWPELNDVVAAIYKIGFTGESPVIPVWLSEKGQDFLRKCLRKDPKQRWTVEELLQHPFLD
EEDNDSDQTGNCLNSSSPSTVLDQRFWDLCETSRSRFIKEDHEDPFANSTNFLWDDDSLPGDRIKKLAGDESSGEPDWETNGWIE
VRGEIEKRNEEEDENCVEATSLEEDEEEVGGFENWIWDQQDSLFLEYSPEDNIYYFYSYYNIFDEDIILYYDHLEDCFVLKFDDNN
KKIFFFSHITNSCFEITNYN

>AtMEKK16

MEINWTRGPIIGRGSTATVSIAISSSGELFAVKSADLSSSSLLQKEQSILSTLSSPHMVKYIGTGLTRESNGLVYNILMEYVSGGNLH
DLIKNSGGKLPEPEIRSYTRQILNGLVYLHERGIVHCDLKSHNVLVEENGVLKIADMGCAKSVDKSEFSGTPAFMAPEVARGEEQ
RFPADVWALGCTMIEMMTGSSPWPELNDVVAAMYKIGFSGESPAIPAWISDKAKDFLKNCLKEDQKQRWTVEELLKHPFLDDD
EESQTSDCLKNKTSSPSTVLDQRFWDSCESSKSHLVSIDHEDPFAEYSESLDSPADRIEKLAGDEFSSLLDWDTEDDGGWIQVRGE
KHKETEKRDGDEDVICVEATSSSQTIEEVEDWISNQDSLFSEYSSDDIINNFYSNVAIQGNLIAFHYCTDEDENVSIKNMFRTNNKH
LFFKLQAHVKFTNQV

>AtMEKK17

MEWTRGRILGRGSTATVYAAAGHNSDEILAVKSSEVHRSEFLQREAKILSSLSSPYVIGYRGSETKRESNGVVMYNLLMEYAPYG
TLTDAAAKDGGRVDETRVVKYTRDILKGLEYIHSKGIVHCDVKGSNVVISEKGEAKIADFGCAKRVDPVFESPVMGTPAFMAPE
VARGEKQGKESDIWAVGCTMIEMVTGSPPWTKADSREDPVSVLYRVGYSSETPELPCLLAEEAKDFLEKCLKREANERWTATQL
LNHPFLTTKPDIEPVLVPGLISNSPTSVTDQTFWRSVEEEEEEETEEIQKDSRDLDRLSLWGCYSERIGRLKCVGGLDGTRCDMEG
GDWIMVRARCEGTMISGSQKELIISENVLVGEL

>AtMEKK18

MNWTRGKTLGRGSTATVSAATCHESGETLAVKSAEFHRSEFLQREAKILSSLNSPYVIGYRGCEITREPFHNNGEATTYSLLMEYA
PYGTLTDVATKNGGFIDEARVVKYTRQILLGLEYIHNSKGIAHCDIKGSNVLVGENGEAKIADFGCAKWVEPEITEPVRGTPAFM
APEAARGERQGKESDIWAVGCTVIEMVTGSQPWIGADFTDPVSVLYRVGYLGELPELPCSLTEQAKDFLGKCLKKEATERWTAS
QLLNHPFLVNKEPELVTGLVTNSPTSVTDQMFWRSVEEEVSEDRSSWWECHEDERIGVLSWIGHVVVESTWDLDGEDWITVRR
N

>AtMEKK19
MEWIRGETIGYGTFSTVSLATRSNNDSGEFPPLMAVKSADSYGAASLANEKSVLDNLGDDCNEIVRCFGEDRTVENGEEMHNLF
LEYASRGSLESYLKKLAGEGVPESTVRRHTGSVLRGLRHIHANGFAHCDLKLGNILLFGDGAVKIADFGLAKRIGDLTALNYGVQ
IRGTPLYMAPESVNDNEYGSEGDVWALGCVVVEMFSGKTAWSLKEGSNFMSLLLRIGVGDEVPMIPEELSEQGRDFLSKCFVKD
PKKRWTAEMLLNHPFVTVDVDHDVLVKEEDFVVNMKTEDVSTSPRCPFEFPDWVSVSSGSQTIDSPDERVASLVTDMIPDWSVT
NSWVTVR
>AtMEKK20
MEWVRGETIGFGTFSTVSTATKSRNSGDFPALIAVKSTDAYGAASLSNEKSVLDSLGDCPEIIRCYGEDSTVENGEEMHNLLLEYA
SRGSLASYMKKLGGEGLPESTVRRHTGSVLRGLRHIHAKGFAHCDIKLANILLFNDGSVKIADFGLAMRVDGDLTALRKSVEIRG
TPLYMAPECVNDNEYGSAADVWALGCAVVEMFSGKTAWSVKEGSHFMSLLIRIGVGDELPKIPEMLSEEGKDFLSKCFVKDPAK
RWTAEMLLNHSFVTIDLEDDHRENFVVKVKDEDKVLMSPKCPFEFDDWDSFTLDSNPSFDSPVERLGSLVSGSIPDWSVGGSWL
TVR
>AtMEKK21
MEWIRRETIGHGSFSTVSLATTSGSSSKAFPSLMAVKSSGVVCSAALRNERDVLDDLGDCSEIVRCFGEGRTVENGEEIYNLFLEY
ASGGSLADRIKSSGEALPEFEVRRFTRSIVKGLCHIHGNGFTHCDIKLENVLVFGDGDVKISDFGLAKRRSGEVCVEIRGTPLYMA
PESVNHGEFESPADIWALGCSVVEMSSGKTAWCLEDGVMNNVMSLLVRIGSGDEVPRIPVELSEEGKDFVSKCFVKNAAERWTA
EMLLDHPFLAVDDESGEEDEACSVSPRNPFDFPGWNSVQSPVNDSVMFGSLVGSPEERISGLVSEKVPDWSVSCDWVNVR
>AtZIK1
MYMEISSASDDSIAYVETDPSGRYGRFREVLGKGAMKTVYKAFDQVLGMEVAWNQVKLNEVFRSPEPLQRLYSEVHLLKNLNH
ESIIRYCTSWIDVNRRTFNFITELFTSGTLREYRRKYQKVDIRAIKSWARQILNGLAYLHGHDPPVIHRDLKCDNIFVNGHLGQVKI
GDLGLAAILRGSQNAHSVIGTPEFMAPELYEEDYNELVDIYSFGMCVLEMLTGEYPYSECTNPAQIYKKVTSGKLPDSFHLIQHTE
AQRFVGKCLETVSRRLPAKELLADPFLAATDERDLAPLFRLPQQLAIQNLAANGTVVEHLPSTTDPTRTTDMSITGKMNSEDHTI
FLQVQILDGDGHMRNIQFPFNILSDTPLEVALEMVKELEITDWDPLEIAAMIENEISLLVPNWRANDSSIRHESFGHEDDEDNGDT
EGRTRLFSSASSSHDSPVAVRENNDDSSNDVIPDMDDGNRSSNRLLNSSTYHYSPAIDDDQNQQRRRVRLQQKMRSLVDTRTQ
VLHRSLMELINKRRGRGFDPNTNELQPQPSSTDFIRRC
>AtZIK2
MNMNQVAEYVETDPTGRYGRFAEILGRGAMKTVYKAIDEKLGIEVAWSQVKLKEVLRSSVDLQRLYSEVHLLSTLNHKSIIRFY
TSWIDVHNHTLNFITELFTSGTLRQYKNKYLRIDIRAIKSWARQILEGLVYLHEHDPPVIHRDLKCDNIFVNGHLGQVKIGDLGLA
RMLRDCHSAHSIIGTPEFMAPELYEENYNELIDVYSFGMCFLEMITSEFPYSECNHPAQIYKKVVGGKLPGAFYRVGDIEAQRFIG
KCLVSASKRVSAKELLQDPFLASDESWMVYTSGAGNPKPFLNENEMDTLKLEDDELRTEMSIAGKLGAEDNKIDLEVQIAYDNG
LANNVFFPFDIMNDTSIDVAKEMVKELEIIDWEPVEIAKMIDGAISSLVSDWKYEEDDETPHDHHRHRTDSFHSSSSHASSSQASL
SNYMARGLQDWVQDDLHDETYSQSSSHSGSYSNLNYIAVDEYSSQSPVMSRTHNMTRFCPEESSHLQSGQANAYAASSSTNRSL
ASDNRTLTRNRSLVDVQRQLLHRSPGEEARKRRLFKTVGDVETVGFQSPYAVSRKPPSSRR
>AtZIK3
MNGEESFVEDCSVFVEIDPSGRYGRYDEILGKGASKTVYRAFDEYEGIEVAWNQVKLRNFTRNPEELEKFFREIHLLKTLNHQNI
MKFYTSWVDTNNLSINFVTELFTSGTLRQYRLRHRRVNIRAVKQWCKQILKGLLYLHSRSPPIIHRDLKCDNIFINGNQGEVKIGD
LGLAAILRKSHAVRCVGTSKPSHHWNFIALIMFFTTLDLPLLCLCVVKGTPEFMAPEVYDEEYNELVDVYAFGMCVLEMVTFDY
PYSECTHPAQIYKKVTSGKKPEAFYLVKDPEVREFVEKCLANVTCRLTALELLQDPFLQDDNMDGFVMRPIDYYNGYDETGVFL
RHPLIDDPLYHDQFESSQICEIDLFANDDEDHVDISIKGKRNGDDGIFLRLRISDAEGIVSIFFDSFKIIGLKNVTRFRVLGNVGRIRNI
YFPFETAIDTAWSVAVEMVSELDITNQDVAKIAEMIDAEIAALVPDWKNDTESSQNVNNNKNNNTAGFCGECASNGYIQETVSSG
EKSHHNHHEFDSSEDKSCSSVHGRFADMWGLRESYSDDGEKQSSRKVRSGRWSENEMRRELRWLKARHKIQLMKMRGQTICE
TPIEISLTPGTSVSLPLLYRAISLPVDAVDM
>AtZIK4
MNNLSYLEPDYSEFVEVDPTGRYGRYNEVLGKGASKTVYRAFDEYEGIEVAWNQVKLYDFLQSPEDLERLYCEIHLLKTLKHKN

IMKFYTSWVDTANRNINFVTELFTSGTLRQYRLRHKRVNIRAMKHWCRQILRGLHYLHSHDPPVIHRDLKCDNIFVNGNQGEVK
IGDLGLAAILRKSHAAHCVGTPEFMAPEVYEEAYNELVDIYSFGMCILEMVTFDYPYSECTHPAQIYKKVMSGKKPDALYKVKD
PEVKCFIEKCLATVSLRVSARELLDDPFLRIDDGEFDLRSVDMEDSVGPLYRQPHHLPDYYNYPSNSSSLNRQYSNGNYPSNSSSL
NRQYSNGYNSHHEYQNGWAYNPAETEETHGIELFESRNNDDQEEEKKSGNVDITIKGKRRDDGGLFLRLRIADKEGRVRNIYFPF
DIETDTALSVATEMVAELDMDDHGVTKIANMIDGEISSLVPSWRPGPEFEECLAAAAAANAASICNNCVSNRTSMGSVMDFLRTN
PGANVIQCCRNGCGETHGRFEEITIRETEVRLRELWKLQQQQESRELSSIDSGHNHSEEEEEEEVLYEDPENMFSCEAGNEINHISG
SGSFSFMPSKYCDEPSEKTENQVQQELRWLKAKCQIELRDIQDEQLKTRWPESGEEVEISPKDGFLGSVSGLGREEDTVKEMFGE
RLVPKCLKRTTSLPVDAIDS

>AtZIK5

MEGTDDASALQEPPDPEVLEVDPTFRYIRYKEVIGKGAFKTVYKAFDEVDGIEVAWNQVRIDDVLQSPNCLERLYSEVRLLKSLK
HNNIIRFYNSWIDDKNKTVNIITELFTSGSLRHYRKKHRKVNMKAVKNWARQILMGLRYLHGQEPPIIHRDLKCDNIFINGNHGE
VKIGDLGLATVMEQANAKSVIGTPEFMAPELYDENYNELADIYSFGMCMLEMVTFDYPYCECKNSAQIYKKVSSGIKPASLSRV
KDPEVKQFIEKCLLPASERLSAKELLLDPFLQLNGLTMNNPLPLPDIVMPKEGAFGDRCLMSEGPPTTRPSKTLSIDLDEDSNLPIV
TFSDNSGSRCIEVRRAKRGNFFVLKGEENDEQSVSLILRIVDENGRVRNIHFLFYQEGDTASKVSSEMVEQLELTDQNVTFIAELID
ILLVNMIPTWKTDVTVDHLIHSQLNQNSRSHHNEAKPQKQEETVFHDTCELVSHSCNSDCPRSDEEDKQCVDATKGEDKSSIQEV
EEATEPVSLEEEERLRQELEEIEAKYQEDMKEIATKREEAIMETKKKLSLMKLK

>AtZIK6

MASGSGFLGQISSMEEADFAEKDPSGRYIRYDDVLGRGAFKTVYKAFDEVDGIEVAWNLVSIEDVMQMPGQLERLYSEVHLLKA
LKHENIIKLFYSWVDEKNKTINMITELFTSGSLRVYRKKHRKVDPKAIKNWARQILKGLNYLHSQNPPVIHRDLKCDNIFVNGNT
GEVKIGDLGLATVLQQPTARSVIGTPEFMAPELYEEEYNELVDIYSFGMCMLEMVTCEYPYNECRNQAQIYKKVTSNIKPQSLGK
VDDPQVRQFIEKCLLPASSRPTALELSKDPFLARDGGKDSALLASSSTSSKYVRPPQLEHLPMDVDHNENKSVSSNEDYPWSQTI
ELQRIAENKEFRLRGERSDDVTASMVLRIADPSGKCRIVHFAFYLESDTATAIAEEMVEELHLTSQEVVVIADMIDDFIMQLLSDRT
SSHHNQNSPRLTHEDHEAANQQTVNSKDEEAAGQSMKSDISADYYFPYSANDGNAAMEAGRDAESMSSYLDSCSMMSTIYNL
SISDNDYPEDLKTELNLIESQFNQSFQDLLKLKEDAIENAKRKWITKKQKAVNIS

>AtZIK7

MEGSEDASAIVEPPDPEVLEIDPTCRYIRYKEVIGKGASKTVFKGFDEVDGIEVAWNQVRIDDLLQSPDCLERLYSEVRLLKSLKH
KNIIRFYNSWIDDKNKTVNIITELFTSGSLRQYRKKHRKVNMKAVKCWARQILTGLKYLHSQDPPIIHRDIKCDNIFINGNHGEVKI
GDLGLATVMEQANAKSVIGTPEFMAPELYDENYNELADIYSFGMCMLEMVTFEYPYCECRNSAQIYKKVSSGIKPASLSKVKDP
EVMKFIEKCLLPASERLSAEELLLDSFLNVNGLVMNNPLPLPDIVMPKEGSFGERCLMSEGPPNARNRTMSMNLDEDNNLPIVISS
NNSGTNCIEVRRAKRGNFFVLKGEENDENSVSLILRIVDENGRVRNIHFLFFQEGDTASNVSSEMVEQLELTDKNVKFIAELIDVL
LVNLIPNWKTDVAVDHLIHPQQNQSSKDNHQNGASSQAGESISHSLSSDYCPRSDDEANPTVAATTEDQEAEKPGSLEEEEEDER
LKEELEKIEERFREEMKEITRKREEATMETKNRFFEKKMQQVE

>AtZIK8

MMTCASSDDNESEKDKDSESFVEVDPTGRYGRYGELLGSGAVKKVYRAFDQEEGIEVAWNQVKLRCFSDDPAMTERLYSEVRL
LKNLKNSNIITLYKVWRDERNNTLNFITEICTSGNLREYRKKHRHVSMRALKKWSKQILKGLDYLHTHDPCIIHRDLNCSNIFVN
GNIGQVKIGDLGLAAIVGKNHLAHSILGTPEFMAPELYEENYTEMVDIYSYGMCVLELVSLEIPYSECDSVAKIYKRVSKGLKPEA
LNKVNDPEAKAFIEKCIAQPRARPSAAELLCDPFFDGILDDDDEDGENNDNNGAGRIVVS

>AtZIK9

MMNNLSHLESDYSEYVEVDPTGRYGRYNEVLGKGSSKTVYRGFDEYQGIEVAWNQVKLYDFLQSPQELERLYCEIHLLKTLKH
KSIMKFYASWVDTDNRNINFVTEMFTSGTLRQYRLKHKRVNIRAVKNWCRQILRGLNYLHTHDPPVIHRDLKCDNIFINGNQGE
VKIGDLGLAACLQHSHAAHCVGTPEFMAPEVYKEEYNQLVDIYSFGMCVLEMVTFDYPYSECSHPAQIYKRVISGKKPDGLDKV
KDPEVRGFIEKCLATVSLRLSACELLDDHFLCIDESDMRRVESEKGLIDEAGTPLRHSYHIPHYSNGYYSLYNQNQWDYNGDETV
ESHEIDLLEFQNDDDEEEEDKRFGSVDISIKGKRRDNGDGLFLRLKTVNKEGCVRNIYFPFDIETDTAISVAREMVEELEMDDRDV
TKIANMIDGEIASLVPNWSIFCSSESNRSSVGSVMDFNEMQCGRDGCEEKHGRFEEITFEITVNDSDEED

>AtZIK10

MEEADFVQKDPTGRYIRYNDVLGRGAFKTVYKAFDEVEGIEVAWNLMSIEDVLQMPGQLDRLYSEVHLLNSLKHDNIIKLFYSW
VDDHNKSINMITELFTSGSLTLYRKKHRKVDPKAIMNWARQILKGLHYLHSQTPPVIHRDLKCDNIFVNGNTGKVKIGDLGLAAV
MQQPTARSVIGTPEFMAPELYEEEYNELVDIYSFGMCMLEMVTCEYPYRECRNQAQIYKKVTSGIKPQSLSKVDDPQVKQFIEKC
LLPAPSRPTALELLKDQLLAVDGAKDSTLTASSNTTFKPAMPPQCEYRPMDVEYKKNTSVSICSSAKSSQECALLQTMEVQRVAE
STEFKLSGERRDDVAASMALRIAGSSGQARKVDFDFNLKTDTARAVTGEMVEELDLSSHEVTVIAEMIDELIMKLKANRSLPNA
NSVYQSKDEEAGESMKSEISADYYHRVSSNEGSRLGCCCEAVESLLSSFLDSCSMVSNKQSEDLKTELNVIESQYNQSCQRLLRM
KEEAIEKAKRKWMKLS

>AtZIK11

MRQDENNSEEEFVEIDPTGRYGRYKEVLGKGAFKEVYRAFDQLEGIEVAWNQVKLDDKFCSSEDLDRLYSEVHLLKTLKHKSII
KFYTSWIDHQHMTINLITEVFTSGNLRQYRKKHKCVDLRALKKWSRQILEGLVYLHSHDPPVIHRDLKCDNIFINGNQGEVKIGD
LGLAAILHRARSAHSVIGTPEFMAPELYEEDYNVLVDIYAFGMCLLELVTFEYPYSECTNAAQIYRKVTSGIKPAALLNVTDPQVR
AFIEKCIAKVSQRLSAKELLDDPFLKCYKENTENVSSHKENGYNGNGIVDKLSDSEVGLLTVEGQRKDLNTIFLKLRITDSKGQIR
NIHFPFNIETDTSFSVAIEMVEELDLTDDQDISTIAKMIDTEIHSHIPDWTPSRLIGDDSAVQKCLSSPETLHLDRFPSGRKFWSSPKA
GAGDSRSPFAPRSNSKLSSAQGPINQEVGVIVEKLESLLRKQREEIEEMQRDQERIVTEFLKEFPPEICEEALVRLQVKDSDNLLC

>AtRAF1

MEMPGRRSNYTLLSQFSDDQVSVSVTGAPPPHYDSLSSENRSNHNSGNTGKAKAERGGFDWDPSGGGGGDHRLNNQPNRVGN
NMYASSLGLQRQSSGSSFGESSLSGDYYMPTLSAAANEIESVGFPQDDGFRLGFGGGGGDLRIQMAADSAGGSSSGKSWAQQTE
ESYQLQLALALRLSSEATCADDPNFLDPVPDESALRTSPSSAETVSHRFWVNGCLSYYDKVPDGFYMMNGLDPYIWTLCIDLHE
SGRIPSIESLRAVDSGVDSSLEAIIVDRRSDPAFKELHNRVHDISCSCITTKEVVDQLAKLICNRMGGPVIMGEDELVPMWKECIDG
LKEIFKVVVPIGSLSVGLCRHRALLFKVLADIIDLPCRIAKGCKYCNRDDAASCLVRFGLDREYLVDLVGKPGHLWEPDSLLNGP
SSISISSPLRFPRPKPVEPAVDFRLLAKQYFSDSQSLNLVFDPASDDMGFSMFHRQYDNPGGENDALAENGGGSLPPSANMPPQN
MMRASNQIEAAPMNAPPISQPVPNRANRELGLDGDDMDIPWCDLNIKEKIGAGSFGTVHRAEWHGSDVAVKILMEQDFHAERV
NEFLREVAIMKRLRHPNIVLFMGAVTQPPNLSIVTEYLSRGSLYRLLHKSGAREQLDERRRLSMAYDVAKGMNYLHNRNPPIVHR
DLKSPNLLVDKKYTVKVCDFGLSRLKASTFLSSKSAAGTPEWMAPEVLRDEPSNEKSDVYSFGVILWELATLQQPWGNLNPAQV
VAAVGFKCKRLEIPRNLNPQVAAIIEGCWTNEPWKRPSFATIMDLLRPLIKSAVPPPNRSDL

>AtRAF2

MKHIFKKLHRGGNQEQQNRTNDAAPPSDQNRIHVSANPPQATPSSVTETLPVAGATSSMASPAPTAASNRADYMSSEEEYQVQL
ALAISASNSQSSEDPEKHQIRAATLLSLGSHQRMDSRRDSSEVVAQRLSRQYWEYGVLDYEEKVVDSFYDVYSLSTDSAKQGEM
PSLEDLESNHGTPGFEAVVVNRPIDSSLHELLEIAECIALGCSTTSVSVLVQRLAELVTEHMGGSAEDSSIVLARWTEKSSEFKAAL
NTCVFPIGFVKIGISRHRALLFKVLADSVRLPCRLVKGSHYTGNEDDAVNTIRLEDEREYLVDLMTDPGTLIPADFASASNNTVEP
CNSNGNKFPTAQFSNDVPKLSEGEGSSHSSMANYSSSLDRRTEAERTDSSYPKVGPLRNIDYSSPSSVTSSTQLENNSSTAIGKGSR
GAIIECSRTNMNIVPYNQNSEEDPKNLFADLNPFQNKGADKLYMPTKSGLNNVDDFHQQKNNPLVGRSPAPMMWKNYSCNEAP
KRKENSYIENLLPKLHRDPRYGNTQSSYATSSSNGAISSNVHGRDNVTFVSPVAVPSSFTSTENQFRPSIVEDMNRNTNNELDLQP
HTAAVVHGQQNDESHIHDHRKYTSDDISTGCDPRLKDHESTSSSLDSTSYRNDPQVLDDADVGECEIPWNDLVIAERIGLGSYGE
VYHADWHGTEVAVKKFLDQDFSGAALAEFRSEVRIMRRLRHPNVVFFLGAVTRPPNLSIVTEFLPRGSLYRILHRPKSHIDERRRI
KMALDVAMGMNCLHTSTPTIVHRDLKTPNLLVDNNWNVKVGDFGLSRLKHNTFLSSKSTAGTPEWMAPEVLRNEPSNEKCDV
YSFGVILWELATLRLPWRGMNPMQVVGAVGFQNRRLEIPKELDPVVGRIILECWQTDPNLRPSFAQLTEVLKPLNRLVLPTPQ

>AtRAF3

MSKMKHLLRKLHIGGSSGVGGGFADHHRLDDSTRPMIDPSPILSTSPSPASTSSVSSSGFGNASTTMPRLDTFEPVGRDLTAVDGV
DFNLMEEEYQVQLAMAISVSDPDPRENADTAQLDAAKRISLGVSAPVTDADSAVDFLSLRYWGHKVINYDQKVRDGFYDVYGI
TSNSLSQGKMPLLVDLQAISISDNVDYEVILVNRLIDPELQELERRVFALASECPDFAPGQVSSDLTQKIANIVVEQMGGPVENAD
EALRRWMLRSYELRNSLNTTILPLGRVNVGLARHRALLFKVLADRINLPCMLVKGSYYTGTDDGAVNLIKLDDKSEYIIDLMGA
PGALIPSEVPSSFLPVSCTDTRVFPENLDSLQHSSPVLEKEIETPAFSVSKEADSRSGMVANFFTGNQEENSDRCAVEKHQTERFEH

DFGKLMHSQQISGENMPPFSGKPTCAQKVKVKNVSKYVISAAKNPEFAQKLHAVLLESGASPPPDLFMDINPHNLRGKNLLQEL
RQESSNSMVSGIPCYPEKVAEQLRESERNPTAESYQQSVEVDLSMKRNFDLDNTGKASSSENMEVGTADGESAVCDSHDQGINP
LLGEAAKWEIMWEDLQIGERIGIGSYGEVYRAEWNGTEVAVKKFLDQDFSGDALTQFKSEIEIMLRLRHPNVVLFMGAVTRPPN
FSILTEFLPRGSLYRLLHRPNHQLDEKRRMRMALDVAKGMNYLHTSHPTVVHRDLKSPNLLVDKNWVVKVCDFGLSRMKHHT
YLSSKSTAGTPEWMAPEVLRNEPANEKCDVYSFGVILWELATSRVPWKGLNPMQVVGAVGFQNRRLEIPDDIDLTVAQIIRECWQ
TEPHLRPSFTQLMQSLKRLQGLNISNRANTSESLM
>AtRAF4
MKMNMKKFLKKLRITPNQRDDGEGSVSNRSNKSSDAEPSPSDSLRSQDNSEFKPFLGLSNWLSSVTHRKSPSSSNATNSKEDDTT
MEHGGPVGSESGMQGLGSSSNSKDPEVEEEYQIQLALELSAREDPEAAQIEAMKQFSLGSRPSAPENTPAELMAYRYWNYNCLG
YDDKIVDGFYDLCGVMNESSLKRIPPLVDLQGTLVSDGVTWDAVLVNSSKDSNLLRLEQMALDIAAKSKSASSSGFVNSELVRQ
LAVLVADYMGGPVLDPDSTLRAWWSLSYSLKATLRSMVLPLGSLTIGLARHRALLFKVLCDSVGVPCRIVKGQQYTGSDDVAM
NSIKTDDGREYIVDLMGDPGTLIPADAAGLQMDFDDSVYSASPRDVDSSHVASSSSGVESSIEEHTESWSAEHRSRTKGSREENQ
SAGGGDLMIPNIREAVGSQKAPVQHLSSKPTHSFTHARSPSWTEGVSSPAGRRMKVKDVSQYMIDAAKENPQLAQKLHDVLLES
GVVAPRNLFSEVYSESMEATGEIKSVAESNDEKGKDFGTIQQGRNQSNLGPVRFLPPLPRPQSKAITHDLREHSGSGLGHLSEHCN
IDGHSDSSHSETSTDYPRNVPVAVAAAAVVASSMVVAAAKSANSDSSTLELSAAAAAAVMATAAAVSRQFELDSLSNGDAGSGG
LHGVDSGGERISDRSIGNESSKSDAAIDDVAECEILWEEITVAERIGLGSYGEVYRGDWHGTAVAVKKFIDQDITGEALEEFRSEVR
MMRRLRHPNIVLFMGAVTRPPNLSIVTEFLPRGSLYRLIHRPNNQLDERKRLRMALDAARGMNYLHSCNPVIVHRDLKSPNLLV
DKNWVVKVCDFGLSRMKVSTYLSSKSTAGTAEWMAPEVLRNEPADEKCDVYSYGVILWELFTLQQPWGKMNPMQVVGAVGF
QHRRLDIPEFVDPGIADIIRKCWQTDPRLRPSFGEIMDSLKQLQKPIQRAAVPSSSALTTDEQEQ
>AtRAF5
MKVKEETLKNLGDGVVLRPVDHCSSIWSMKMNMKNFLKKLHISPNQSDEAEGSISTTKSNHHKSIDVSSSSSPRSHHSNSPEIKP
FSGLSNWLSSVGHRKIPSPPNSFNAKNRAATVDDTVVVNGSEHVDLGSKDPAVEEENQIQLALELSAREDPEATQIEAIKQFSLGS
CAPENSPAELIAYRYWNYNCLGYDDKILDGFYDLYGVLNASSAERIPPLLDLQGTPVSDGVTWEAVLVNRSGDSNLLRLEQMAL
DIAAKSRSVSSSGFVNSELVRKLAILVGDYMGGPVVHPESMLRAWRSLSYSLKATLGSMVLPLGSLTIGLARHRALLFKVLCDSV
GVPCRIVKGQQYTGSEDVAMNFIKADDGREYIVDLMGDPGTLIPADAAGLQIDYDESAYSASPGDNDSIHVASSSNGIESSYEENT
EFRTGEHRSSTKSSGERNQSGGGGDLIVHPNISREDVKNQKKVEKAPFQNLSSRPIHSFTHMRSPSWTEGVSSPAAQRMKVKDVS
QYMIDAAKENPRLAQKLHDVLLESGVVAPPNLFSEVYPQQLEATVESKNSTEAKKERGKDLETTQEGRHQNGFGPVRFLPPLPR
VQSKTNAHDQRDNGKVVSQSDSSHSEASSTEYARTVPAAVAAAAVVASSMVAAAAAKSANSDSSPIELPAAAAATATAAAVVATA
AAVSRQLELGSNSDGDDGSGGHEPQGSGDSNHGPNSGGERISDKSIGNESSKSDCDDVSDCEILWEEITVGERIGLGSYGEVYRG
DWHGTEVAVKKFLDQDLTGEALEEFRSEVRIMKKLRHPNIVLFMGAVTRPPNLSIVTEFLPRGSLYRLIHRPNNQLDERRRLRMA
LDAARGMNYLHSCNPMIVHRDLKSPNLLVDKNWVVKVCDFGLSRMKHSTYLSSKSTAGTAEWMAPEVLRNEPADEKCDVYS
YGVILWELFTLQQPWGKMNPMQVVGAVGFQHRRLDIPDFVDPAIADLISKCWQTDSKLRPSFAEIMASLKRLQKPVTGSNIPRPV
PSSSSLPTEHEQKD
>AtRAF6
MPHRTTYFFPRQFPDRGFDSFSLKNDHDKKKSSSNVGESFGFQRDNKSNGVGEDSNKEKESTVFSSNPLLSKSSAVSDLFSDDRK
SEKKHQQQLAAFYEWLAEKKANLSRSSSTTTTHGRGVKPTRFSMSSDADEERELLLSSPADPAPLPATSSPDSIIDSARTVNIHERN
IDRSFDREVSLPRMSSESSFAGSFFSGTTVDGNFSNFSSHTDARETSTTTLVSVNKEEEEVEVREQGKEQSLAQKSREGYYLQVTL
AKWLSSQANLACESVHIQSTESISYRFWVSGCLSYSDKISDGFYSILGMDPYLWLMCNNSEDGKRIPSLLLLKETEPNDTSMEVV
LIDRREDSRLKELEDKAHELYCSSDNMLVLVEKLGRLVAVYMGGNFQVEQGDLQKRWKLVSNRLKEFRKCIILPIGSLTMGLCRH
RAILFKKLADYIGLPCRIARGCRYCKESHQSSCLVKIDDDRKLSREYVVDLIGEPGNVHDPDSSINGETQCQIPSPLQMSHLTDFSR
PCVHSTSPCQTVESKTSRTLSENIQRSGSQGQVHKEFELPDNAGTVCCAHIDQTCCAKVSSMVLTESVLRALPLDIPNLSEEKIAP
QETCKEETVLLEDPTAMKQPNLSVEPEIVEADTRKDKKGRLPVDAISPYLTIEPSLASDWLEVSWNELHIKERVGAGSFGTVHRA
EWHGSDVAVKILSIQDFHDDQFREFLREVCKQAVAIMKRVRHPNVVLFMGAVTERPRLSIITEYLPRGSLFRLIHRPASGELLDQRR
RLRMALDVAKGLNYLHCLNPPVVHWDLKSPNLLVDKNWTVKVCDFGLSRFKANTFIPSKSVAGTPEWMAPEFLRGEPTNEKSD

VYSFGVVLWELITLQQPWNGLSPAQVVGAVAFQNRRLIIPPNTSPVLVSLMEACWADEPSQRPAFGSIVDTLKKLLKSPVQLIQMG
GDKGVIPTKSAPIL

>AtRAF7

MENPPAEELLKKILELEESQEHLKQEMSRLKVSTELRQRSHSVSPHRPARRNIGEGAPSWRKSGAASFRNASPLRKESRIQNSMR
LRSEVGGGGPSAGKFTDKQYLNILQSMAQAVHAFDLNMRIIFWNAMAEKVYGYSAAEALGENPINVIADDRDAAFAMNIARRC
VRGESWTGEFPVKSKSGDRFSAVTTCSPFYDDDGALMGIICITSNTAPYLNPRISLAKLKAQEEGETSSIPARNSFASKLGLDSRGA
VISKLGLDSDQPIQVAIASKISDLASKVSNKVRSKMRAGDNSATLSEGGSGDSHQKDHNVFGATLVDHRDDAASSGASTPRGDFI
QSPFGVFTCNDEKFVSKPFKDSSDESDGKPAIHKVLTSKAEEWMVKKGLSWPWKGNEQEGSKGRPTNSVWPWVQNEQKKERC
HQINPSAGVQYESHAFESNKPINNEASSLWSSPINANSTSSASSCGSTSSSVMNKVDTDSEGLEYEILWDDLTIGEQVGQGSCGTV
YHGLWFGSDVAVKVFSKQEYSAEVIESFKQEVLLMKRLRHPNVLLFMGAVTSPQRLCIVSEFLPRGSLFRLLQKSTSKLDWRRRI
HMALDIARGMNYLHHCSPPIIHRDLKSSNLLVDKNWTVKVADFGLSRIKHETYLTSKSGKGTPQWMAPEVLRNESADEKSDIYS
FGVVLWELATEKIPWETLNSMQVIGAVGFMDQRLEIPKDIDPRWISLMESCWHSDTKLRPTFQELMDKLRDLQRKYMIQFQATR
AALSDNSLLKDN

>AtRAF8

MEEELLKKMLELEQSQELLKQEMSRLKLSTELRQPSHPVLPRRPLRRIQGSMNSNPSPGKFTDKQYLNILQSLAQSVHVLDLNTR
IIFWNAMSEKLYGYSAAEVVGRNPVHVIVDDQNAAFALNVARRCANGESWTGEFPVKTKSGKIFSAVTTCSPFYDDNGTVVGIIS
ITSDIAPYLNPRLSLPRLKPQEPERKLGLDSKGAVISKPGLDSDQPIQVSIASKISSLASKLSNKVRSKMRAGDNSACGDSHHSDHD
VFGDTLSDHRDDAASSGASTPRGDFIQSPFGVFTCYDDKFPSKPSKDSSDRKPAIHKVPTSKAEEWMVKKGLSRPWKGNEQEGS
RVRPTHSVWSWVENEQEKDKYHQIYPSAGVKSESHGSESNKPTDDEASNMWSSSINANSTNSASSCGSTSRSVMDKVDIDSDPL
EHEILWDDLTIGEQIGRGSCGTVYHGIWFGSDVAVKVFSKQEYSESVIKSFEKEVSLMKRLRHPNVLLFMGAVTSPQRLCIVSEFV
PRGSLFRLLQRSMSKLDWRRRINMALDIARGMNYLHCCSPPIIHRDLKSSNLLVDRNWTVKVADFGLSRIKHQTYLTSKSGKGTP
QWMAPEVLRNESADEKSDIYSFGVVLWELATEKIPWENLNSMQVIGAVGFMNQRLEIPKDTDPDWISLIESCWHR

>AtRAF9

MVKLLQDPITPNKELLKKMIELEKSQEHLMQEMSRLKVSTELRKESRIQCSMNLRPSPWKFTHKQYLNILQSMAQSVHAFDLN
MRIIFWNAMAEKVYGYSAAEAVGQNPIDVMVDDRDAPFAMTIAQLCSNGESWTGKFPVKRRTGEKFSAVTTCSPFYADDGSLIG
IVSITSDVAPYLNPTISLAKLKASEVETSSTPARNSFAFKLGLDTKGAVVSKLGLDSDQPIQVAIASKISDLASKVRNKVRSKMPAG
DSSVTVFEGETGDSHHSDHGVFGATLSDHMDDAASSGASTPRGDFIQSPFGVFTCNDDKFSSEPFIDSSDGYPITLFTSKAEEWM
VKKGLSWPWKGNEQEGSRVKPTYSVWPCVQNEQKKDKSHQINRYSGVKSKSHASESNKPTNNKASGLRSSCINANSAISRGIIS
HSTMNKVDTNSNCLEYEILWDDLTIGEQIGQGSCGTVYHGLWFGSDVAVKLISKQEYSEEVIQSFRQEVSLMQRLRHPNVLLFM
GAVTLPQGLCIVSEFLPRGSLFRLLQRNMSKLDWRRRINMALDIARGMNYLHRCSPPIIHRDLKSSNLLVDKNLTVKVADFGLSRI
KHHTYLTSKSGKGMPQWMAPEVLRNESADEKSDIYSFGVVLWELATEKIPWENLNSMQVIGAVGFMNQRLEIPKDIDPDWISLIE
SCWHRDAKLRPTFQELMERLRDLQRKYTIQFQATRWLTMVRTESSQVLKQNH

>AtRAF10

MEKTTPPAEELLKKIRELEESQEHLKREMSRLKVSAEMKQRSHSASPQRPVRRNSNDGTPMWRKTGAASFRHASPLRKESHAKV
AGGGGEGQSAGKFTDKQYLNILQSMAQAVHVFDLNGQIIFWNSMAEKLYGFSASEALGKDPIDILVDVQDASVAQNITRRCSSG
ESWTGEFPVKNKAGERFSVVTTMSPSYDDDGCLIGIICITNDSALFQDPRGSPAKTRRGQEGETSFSRVTSSVASKLGLDSKEAVV
SKLGLDSQQPIQVAIASKISDLVGNKVKSKMRAGDNNAANLEGGSGDSHQSDQGFFDAAFADRREDAATSGADTPRGDFIQSPF
GVFLRSDEKASTKPFRDSSDESDGNSVVPKTLTSKAEEWMVKKGLSWPWKGNEREGLEGRRSHSVWPWVRNEQQKQQAYQS
NSNHSVKSESQACESIKASSNEPMGYWSSSVNVNSTSSSSSCGSTSSSVMNKVDMDSDCLDYEILWEDLTIGEQIGQGSCGTVYH
GLWFGSDVAVKVFSKQEYSEEIITSFRQEVSLMKRLRHPNVLLFMGAVTSPQRLCIVTEFLPRFGLITLANITLPFVLFELRGQNSD
LPYQITFAVEVSSVCCRGTRQNWIGGDVSIWPRIFYLAYYSLDSLIQLLYLVYNMLHIFLTYFFAQARGMNYLHHCTPPIIHRDLKS
SNLLVDKNWTVKVADFGLSRIKHETYLTTKTGRGTPQWMAPEVLRNEAADEKSDVYSFGVILWELVTEKIPWESLNAMQVIGA
VGFMNQRLEVPKNVDPQWISLMESCWHSEPQDRPSFQEIMEKLRELQRKYTIQFQAARAASIENSALKEK

>AtRAF11

MENPNPPAEKLLKKIRELEESQEDLKREMSKLKVSAEIKRRSHSSSPKRPSRRNSGEGTPLWRKTGAASFRHASPLRKESHSKDG

VAGGGDGPSAGKFTDKQYLNILQSMAQAVHVFDLNGQIIFWNSMAEKLYGFSAAEALGKDSINILVDGQDAAVAKNIFQRCSSG

ESWTGEFPVKNKMGERFSVVTTISPFYDDDGLLIGIICITNDSALFQRPRVPPAKNRWQEGDSSFCRGTNGVASRLGFDSKEAVVS

KLGLDSQQPIQAAIASKISDLASKVGNKVRSKMRAGDNNASHPEGGNGGSHQSDQGFFDAAFSDQREDAETNDASTPRGNLIQS

PFGVFLCNDDKSSSKASGESNDENDRNSVVPKKLTSKTEEWMVKKGLSWPWKGNEREGLERRNAHSVWPWVHNEQQKEEAH

HSNSYNSVKSESLASESNKPANNENMGSVNVNSASSASSCGSTSSSVMNKVDMDSDCLDYEILWEDLTIGEQIGQGSCGTVYHG

LWFGSDVAVKVFSKQEYSEEIITSFKQEVSLMKRLRHPNVLLFMGAVASPQRLCIVTEFLPRGSLFRLLQRNKSKLDLRRRIHMAS

DIARGMNYLHHCSPPIIHRDLKSSNLLVDRNWTVKVADFGLSRIKHETYLTTNGRGTPQWMAPEVLRNEAADEKSDVYSFGVVL

WELVTEKIPWENLNAMQVIGAVGFMNQRLEVPKDVDPQWIALMESCWHSEPQCRPSFQELMDKLRELQRKYTIQFQAARAASI

DNSSLKEK

>AtRAF12

MAGNNSESSLYQVLVEWCQRMETSQARLREDVDDLLLQEESRTGKESATGLETDTEKEAEVEVEAEAADSWDNPTATWERAVS

GFYFADSAYRTLMDSMGHAIHVTSAASGEITFWSRSAENLYHWYAEEVVGYRTIDVLVTEEYRNSLTGIRNRVCRGETWTGQFPF

QKKTGELFMALVTKSPVYENGELVGVVTVSSDATLFNRMHPLSNEHQQQARSNNRHESNLRKHQWHLPRPQIAAASQVPVVPQ

YSSAVASNLKASKLLPQRNGDDSFNGNHNSRSRDENVPVVASTTFEKYGSLADKFLGKLQRKITGSQGTEDNEPILRNGINKSAC

GSGGSSKASNAVTCTAFRDNGNGKPKRAEVRISDVYGNGAEGLIHNGDRFQYIGNLGQSKPPRGLESGLVSGMRGTKMSDLNG

EIEDAWNTRLSVDPLPILGVNSGRQQSPVNQRNNRLVTDSSCEIRWEDLQLGEEVGRGSFAAVHRGVWNGSDVAIKVYFDGDYN

AMTLTECKKEINIMKKLRHPNVLLFMGAVCTEEKSAIIMEYMPRGSLFKILHNTNQPLDKKRRLRMALDVARGMNYLHRRNPPI

VHRDLKSSNLLVDKNWNVKVGDFGLSKWKNATFLSTKSGKGTPQWMAPEVLRSEPSNEKCDVFSFGVILWELMTTLVPWDRL

NSIQVVGVVGFMDRRLDLPEGLNPRIASIIQDCWQTDPAKRPSFEELISQMMSLFRKPGSGAQEEDD

>AtRAF13

MEERRDDESSPTHQGSELAERVKLLSFESQGEALSKDSPRSVEQDCSPGQRASQHLWDTGILSEPIPNGFYSVVPDKRVKELYNR

LPTPSELHALGEEGVRIEVILVDFQKDKKLAMLKQLITTLVSGSGTNPALVIKKIAGTVSDFYKRPTLESPSKLALEENAFLFENHG

AQLLGQIKRGCCRARAILFKVLADTVGLESRLVVGLPSDGTVNCMDSNKHMSVIVVLNSVELLVDLIRFPGQLVPRSAKAIFMSH

ISPAGESDSAENDSCDSPLEPNSPLYERRDPESTEKDENLQFYRKLEGYPNASGSSLRSLMLRPSTAIERKLSNTSHSEPNVATVFW

RRSRRKVIAEQRTASSSPEHPSMRRGRSMLSTGRNSFRDYTGEASSPSSSSTSEIRKTRRRSFRITPEIGDDIASAVREMYEKSKQNR

LLQGREDENSSVIDNNVSGLHLDDELNSKKTMSLPSSPHAYRCQTFGRRGPSEFAVKDTWNKVVESSTLQNQPLLPYQEWDIDF

SELTVGTRVGIGFFGEVFRGVWNGTDVAIKLFLEQDLTAENMEDFCNEISILSRVRHPNVVLFLGACTKPPRLSMITEYMELGSLY

YLIHMSGQKKKLSWHRRLRMLRDICRGLMCIHRMKIVHRDLKSANCLVDKHWTVKICDFGLSRIMTDENMKDTSSAGTPEWM

APELIRNRPFTEKCDIFSLGVIMWELSTLRKPWEGVPPEKVVFAVAHEGSRLEIPDGPLSKLIADCWAEPEERPNCEEILRGLLDCE

YTLC

>AtRAF14

MMQSDLLKERGVDDSSPYSPDEKNVSGFQLDSHDLVSGECSTVYPRKSISLPSSPRSYQIQLSERSEHSPQEISHIWNEVLESPMFQ

NKPLLPFEEWNIDFSKLKVGASVGSGTSGVVCRGVWNKTEVAIKIFLGQQLTAENMKVFCNEISILSRLQHPNVILLLGACTKPPQ

LSLVTEYMSTGSLYDVIRTRKKELSWQRKLKILAEICRGLMYIHKMGIVHRDLTSANCLLNKSIVKICDFGLSRRMTGTAVKDTE

AAGTPEWMAPELIRNEPVTEKSDIFSFGVIMWELSTLSKPWKGVPKEKVIHIVANEGARLKIPEGPLQKLIADCWSEPEQRPSCKE

ILHRLKTCEIPIC

>AtRAF15

MGETGDDAGPSEQGPSNQTWWPSEFVEKFGSVYLGSQEETSSTKDSPRNLGQDGLPSSTASNILWSTGSLSEPIPNGFYSVIPDNR

LKQLFNNIPTLEDLHALGDEGLKADVILVDFQKDKKLFRQKQLITKLVSGLNSKPATIIKKIAGLVADVYKQSTLQSPAKSTQSFEN

CGIQLLGQIKHGSCRPRAILFKVLADTVGLQSRLVVGLPSDGAAESVDSYSHISVTVLLNSVEMLVDLMRFPGQLIPLSTKAIFMS

HISAAGESDSAENDSCDSPLEPNSPMFGYPEKFDHENAEKDENLSLHRKLDGSPNTSGPPSRNMLLRSASALERKLSFSQSESNM

ANEFWRQSRRKVIADQRTASSSPEHLSFRARTKSMLSGDKNLARDFTGDVATSSCKSVGGAKMETKRIRRRSISITPEIGDDIVRA

VRAMNEALKQNRLSKEQGDDDSSPNSPNDRTESSHLQKNVSGFHLDAHDQVSGGRSTLSREPLDPQKAISLPSSPQNYRSQYEQ

SGSSHRNISHIWDKVLGSPMFQNKPLLPYEEWNIDFSELTVGTRVGIGFFGEVFRGIWNGTDVAIKVFLEQDLTAENMEDFCNEISI
LSRLRHPNVILFLGACTKPPRLSLITEYMEMGSLYYLLHLSGQKKRLSWRRKLKMLRDICRGLMCIHRMGIVHRDIKSANCLLSN
KWTVKICDFGLSRIMTGTTMRDTVSAGTPEWMAPELIRNEPFSEKCDIFSLGVIMWELCTLTRPWEGVPPERVVYAIAYEGARLEI
PEGPLGKLIADCWTEPEQRPSCNEILSRLLDCEYSLC

>AtRAF16

MRMEFPGSSNQHLGRDRFNGEVGCGNNCSQTGEEFSNEFLRDFGAQRRLQHGGVNRNVEGNYNNNRHLVYEDFNRILGLQRVD
SNMSEGINSSNGYFAESNVADSPRKMFQTAISDVYLPEVLKLLCSFGGRILQRPGDGKLRYIGGETRIISIRKHVGLNELMHKTYA
LCNHPHTIKYQLPGEDLDALISVCSDEDLLHMIEEYQEAETKAGSQRIRVFLVPSTESSESPKIFHERNMNINRNTNQQTDIDHYQ
YVSALNGIVDVSPQKSSSGQSGTSQTTQFGNASEFSPTFHLRDSPTSVHTWEHKDSNSPTFMKPYGNTNAVHFMPKMQIPRNSFG
QQSPPTSPFSVHKRANTDVPYFADQNGFFDPYLAAPNFPQQNRFFFETTTQKQKHPEVNLHDRRPSDDIYPHGQAYIGAEKMTL
KKNALSDPQLHDESQINNGLEAFTKQPWKILRKNLRVVATSKWEDSDDIYFNNPEGKRCKELELTKEVPNSWINRDNNPDSFDQ
ATKKQDGSNSNSSFSPNYFSPNHQPAAQITSSDSQDSGSSVFSLSVNTNENYLDCSREKFNGFQHDMSLDILIRSHTSATDQLCSTT
KSSDKADYSSPNTNFPVVFLRQEPMIPRHDLETNSDDSDTQKSLPREESIHYSGLPLRKVGSRETTFMHTQGSDDFFKSKLLGPQL
IVEDVTNEVISDNLLSATIVPQVNRESDDDHKSYTREKEITNADHESEMEEKYKKSRNTDDSFSEAAMVEIEAGIYGLQIIKNTDL
EDLHELGSGTFGTVYYGKWRGTDVAIKRIKNSCFSGGSSEQARQTKDFWREARILANLHHPNVVAFYGVVPDGPGGTMATVTE
YMVNGSLRHVLQRKDRLLDRRKKLMITLDSAFGMEYLHMKNIVHFDLKCDNLLVNLRDPQRPICKVGDFGLSRIKRNTLVSGG
VRGTLPWMAPELLNGSSNRVSEKVDVFSFGIVMWEILTGEEPYANLHCGAIIGGIVNNTLRPPVPERCEAEWRKLMEQCWSFDP
GVRPSFTEIVERLRSMTVALQPKRRT

>AtRAF17

MSSDSPAAGDGGEQAAAGTSVPSPSYDKQKEKARVSRTSLILWHAHQNDAAAVRKLLEEDPTLVHARDYDKRTPLHVASLHGW
IDVVKCLLEFGADVNAQDRWKNTPLADAEGARKQKMIELLKSHGGLSYGGQNGSHFEPKPVPPPIPKKCDWEIEPAELDFSNAAM
IGKGSFGEIVKAYWRGTPVAVKRILPSLSDDRLVIQDFRHEVDLLVKLRHPNIVQFLGAVTERKPLMLITEYLRGGDLHQYLKEKG
GLTPTTAVNFALDIARGMTYLHNEPNVIIHRDLKPRNVLLVNSSADHLKVGDFGLSKLIKVQNSHDVYKMTGETGSYRYMAPEV
FKHRRYDKKVDVFSFAMILYEMLEGEPPFANHEPYEAAKHVSDGHRPTFRSKGCTPDLRELIVKCWDADMNQRPSFLDILKRLE
KIKETLPSDHHWGLFTS

>AtRAF18

MDRNRPPHPFQQHAMEPGYVNDSVPQGFTPDQTGLSNANVRPNPADVKPGLHYSIQTGEEFSLEFLRDRVISQRSANPIAAGDIN
YPTGYNGHAGSEFGSDVSRMSMVGNGIRQYERTNPPVHEFGNKLGHIHSAPEASLCQDRSLGNFHGYASSSASGSLTAKVKVLC
SFGGKILPRPGDSKLRYVGGETHIISIRKDISWQELRQKVLEIYYRTHVVKYQLPGEDLDALVSVSCDEDLLNMMEEYNEMENRG
GSQKLRMFLFSVSDLDGALLGVNKSDVDSEFQYVVAVNDMDLGSRSNSTLNGLDSSSANNLAELDVRNTEGINGVGPSQLTGID
FQQSSMQYSESAPPTSFAQYPQSIPHNGAFQFQQAVPPNATLQYAPSNPPSSSVHYPQSILPNSTLQYPQSISSSSYGLYPQYYGETE
QFPMQYHDHNSSNYSIPIPFPGQPYPHPGITQQNAPVQVEEPNIKPETKVRDYVEPENRHILATNHQNPPQADDTEVKNREPSVAT
TVPSQDAAHMLPPRRDTRQNTPVKPSTYRDAVITEQVPVSGEDDQLSTSSGTCGLVHTDSESNLIDLDYPEPLQPTRRVYRSERIP
REQLEMLNRLSKSDDSLGSQFLMSHPQASTGQQEPAKEAAGISHEDSHIVNDVENISGNVVASNETLDKRTVSGGGIETEARNLS
HVDTERSHDIPEKQTSSGVLIDINDRFPQDFLSEIFAKALSDDMPSGANPYQHDGAGVSLNVENHDPKNWSYFRNLADEQFSDR
DVAYIDRTPGFPSDMEDGGEIARLHQVAPLTENRVDPQMKVTESEEFDAMVENLRTSDCEQEDEKSETRNAGLPPVGPSLADYDT
SGLQIIMNDDLEELKELGSGTFGTVYHGKWRGSDVAIKRIKKSCFAGRSSEQERLTGEFWGEAEILSKLHHPNVVAFYGVVKDGP
GATLATVTEYMVDGSLRHVLVRKDRHLDRRKRLIIAMDAAFGMEYLHAKNIVHFDLKCDNLLVNLKDPSRPICKVGDFGLSKIK
RNTLVSGGVRGTLPWMAPELLNGSSSKVSEKVDVFSFGIVLWEILTGEEPYANMHYGAIIGGIVNNTLRPTIPSYCDSDWRILMEE
CWAPNPTARPSFTEIAGRLRVMSTAATSNQSKPPAHKASK

>AtRAF19

MEKKRFDSMESWSMILESENVETWEASKGEREEWTADLSQLFIGNKFASGAHSRIYRGIYKQRAVAVKMVRIPTHKEETRAKLE
QQFKSEVALLSRLFHPNIVQFIAACKKPPVYCIITEYMSQGNLRMYLNKKEPYSLSIETVLRLALDISRGMEYLHSQGVIHRDLKS
NNLLLNDEMRVKVADFGTSCLETQCREAKGNMGTYRWMAPEMIKEKPYTRKVDVYSFGIVLWELTTALLPFQGMTPVQAAFAV

AEKNERPPLPASCQPALAHLIKRCWSENPSKRPDFSNIVAVLEKYDECVKEGLPLTSHASLTKTKKAILDHLKGCVTSISSPFSSSSV
PVNA

>AtRAF20

MDKARHQQLFQHSMEPGYRNETVPQPFMPDQTGSASANMRPPNSNGSDVKAVHNFSIQTGEEFSLEFMRDRVIPQRSSNPNGA
GDMNYNTGYMELRGLIGISHTGSECASDVSRFSTVENGTSDIERTNSSLHEFGNKLNHVQSAPQALLSKDSSVGNLHGYKNTSSS
ASGSVTAKVKILCSFGGKILPRPGDSKLRYVGGETHIISIRKDISWQELRQKILEIYYQTRVVKYQLPGEDLDALVSVSSEEDLQNM
LEEYNEMENRGGSQKLRMFLFSISDMDDALLGVNKNDGDSEFQYVVVAVNGMDIGSGKNSTLLGLDSSSANNLAELDVRNTEGI
NTIAGDVVGVGASQLMVNGFQQTSAQQSESIPPSSSLHYSQSIPLNAAYQLQQSVPPSSALHYPQSITPGSSLQYPQSITPGSSYQY
PQSIIPGSASSYGIYPQYYGHVVQHGERERFPLYPDHSSNYSAIGETTSSIPIQGHVSQQGGWAEGYPYPGSTPKSTQALAEEQKVS
SDMKIREEVEPENRKTPGNDHQNPPQIDDVEVRNHNQVREMAVATTPPSQDAHLLPPSRDPRQNTTAKPATYRDAVITGQVPLSG
IEDQLSTSSSTYAPVHSDSESNLIDLNYPEPEQSSQRVYCSERIPREQLELLNRLSKSDNSLSSQFVTSESPANTAQQDSGKEAVGKS
HDEFKTVNDDANHHTHKDVETIFEKVGVSDETLESEPLHKIVNPDDANKNRVVNGADTEIGVSNLSHVNAAMSHVIPEEQASLQ
GDILIDINDRFPRDFLSEIFSQAISEDTSTVRPYPHDGAAVSMNVQNHDRKNWSYFQQLAEDQFIQRDVVLDQADSRIPSDRKDG
GESSRLPYVSPLSRDGISTNLANPQLTLGQDYGGNFSEKDGGGTGSIPPALENEQMKVTESEEFGAMVENLRTPDSEPKDEKTET
RHAALPPLGSEFDYSGLQIIKNEDLEELRELGSGTFGTVYHGKWRGSDVAIKRIKKSCFAGRSSEQERLTGEFWGEAEILSKLHHP
NVVAFYGVVKDGPGGTLATVTEYMVDGSLRHVLVRKDRHLDRRKRLIIAMDAAFGMEYLHSKNTVHFDLKCDNLLVNLKDPS
RPICKVGDFGLSKIKRNTLVSGGVRGTLPWMAPELLNGSSSKVSEKVDVFSFGIVLWEILTGEEPYANMHYGAIIGGIVNNTLRPTI
PGFCDDEWRTLMEECWAPNPMARPSFTEIAGRLRVMSSAATSTQSKPSAHRASK

>AtRAF21

MTIKDESESCGSRAVVASPSQENPRHYRMKLDVYSEVLQRLQESNYEEATLPDFEDQLWLHFNRLPARYALDVKVERAEDVLTH
QRLLKLAADPATRPVFEVRSVQVSPRISADSDPAVEEDAQSSHQPSGPGVLAPPTFGSSPNFEAITQGSKIVEDVDSVVNATLSTRP
MHEITFSTIDKPKLLSQLTSLLGELGLNIQEAHAFSTVDGFSLDVFVVDGWSQEETDGLRDALSKEILKLKDQPGSKQKSISFFEH
DKSSNELIPACIEIPTDGTDEWEIDVTQLKIEKKVASGSYGDLHRGTYCSQEVAIKFLKPDRVNNEMLREFSQEVFIMRKVRHKNV
VQFLGACTRSPTLCIVTEFMARGSIYDFLHKQKCAFKLQTLLKVALDVAKGMSYLHQNNIIHRDLKTANLLMDEHGLVKVADFG
VARVQIESGVMTAETGTYRWMAPEVIEHKPYNHKADVFSYAIVLWELLTGDIPYAFLTPLQAAVGVVQKGLRPKIPKKTHPKVK
GLLERCWHQDPEQRPLFEEIIEMLQQIMKEVNVVV

>AtRAF22

MLEGAKFNVLAVGNHHNNDNNYYAFTQEFYQKLNEGSNMSMESMQTSNAGGSVSMSVDNSSVGSSDALIGHPGLKPVRHYSL
SVGQSVFRPGRVTHALNDDALAQALMDTRYPTEGLTNYDEWTIDLRKLNMGPAFAQGAFGKLYKGTYNGEDVAIKILERPENSP
EKAQFMEQQFQQEVSMLANLKHPNIVRFIGACRKPMVWCIVTEYAKGGSVRQFLTRRQNRAVPLKLAVKQALDVARGMAYVH
GRNFIHRDLKSDNLLISADKSIKIADFGVARIEVQTEGMTPETGTYRWMAPEMIQHRAYNQKVDVYSFGIVLWELITGLLPFQNM
TAVQAAFAVVNRGVRPTVPNDCLPVLSDIMTRCWDANPEVRPCFVEVVKLLEAAETEIMTTARKARFRCCLSQPMTID

>AtRAF23

MANVVGQLKRGISRQFSTGSLRRTLSRQFTRQASHDPRRNNMRFSFGRQSSLDPIRRSPDGSNGPQLAVPDNLDATMQLLFVACR
GDVEGVQDLLDEGIDVNSIDLDGRTALHIAACEGHVDVVKLLLTRKANIDARDRWGSTAAADAKYYGNMDVFNILKARGAKV
PKTKRTPMVVANPREVPEYELNPQELQVRKADGISKGIYQVAKWNGTKVSVKILDKDLYKDSDTINAFKHELTLFEKVRHPNVV
QFVGAVTQNVPMMIVSEYHPKGDLGSYLQKKGRLSPAKVLRFALDIARGMNYLHECKPEPVIHCDLKPKNIMLDSGGHLKVAG
FGLISFAKLSSDKSKILNHGAHIDPSNYCMAPEVYKDEIFDRSVDSYSFGVVLYEMIEGVQPFHPKPPEEAVKLMCLEGRRPSFKA
KSKSCPQEMRELIEECWDTETFVRPTFSEIIVRLDKIFVHCSKQGWWKDTFKFPWK

>AtRAF24

MDQAKGYEHVRYTAPDPRDEGLGSINQRFSHDSSTNVNTYVRPPDYGVSTPARPVLNYSIQTGEEFAFEFMRDRVIMKPQFIPNV
YGEHSGMPVSVNLSALGMVHPMSESGPNATVLNIEEKRQSFEHERKPPSRIEDKTYHELVQSAPVISSKNDTGQRRHSLVSSRAS
DSSLNRAKFLCSFGGKVIPRPRDQKLRYVGGETRIIRISKTISFQELMHKMKEIFPEARTIKYQLPGEDLDALVSVSSDEDLQNMM
EECIVFGNGGSEKPRMFLFSSSDIEEAQFVMEHAEGDSEVQYVVAVNGMDLSSRRSSLGLSPPGNNLDELLHGNFDRKIDRAATE

PAVASLTPLAGNESLPASQTSQPVTGFSTGNEPFSQPYLGQQLQFPGLGNHQIYTSGHMASIGYIDEKRSAPLHVQPQPHYIPYSVN
PETPLESLVPHYPQKPEQGFLREEQIFHVQDPETSSKEAKMRRDDSFQKVNDHPISTVESNLSAKEPKMRRESSTPRVNEYPVSSM
PSDLIVPDDLPKEEAPIVTQTSSSTPDPSSSTLSEKSLRKSEDHVENNLSAKEPKMRKEHSTTRVNEYSVSSVSSDSMVPDQALKEE
APISMKISNSTPDPKSLVYPEKSLRTSQEKTGAFDTTNEGMKKNQDNQFCLLGGFSVSGHGTSNNSSSNVSNFDQPVTQQRVFHS
ERTVRDPTETNRLSKSDDSLASQFVMAQTTSDAFLPISESSETSHEANMESQNVHPTAPVIPAPDSIWTAEGSMSQSEKKNVETNT
PEHVSQTETSAKAVPQGHNEKGDIVVDINDRFPREFLADILKTKESLNFPGLGPLHADGAGVSLNIQNNDPKTWSYFRNLAQDEF
ERKDLSLMDQDHPGFPTSMTNTNGVPIDYSYPPLQSEKVASSQIHPQIHFDGNIKPDVSTITIPDLNTVDTQEDYSQSQIKGAESTD
ATLNAGVPLIDFMAADSGMRSLQVIKNDDLEELKELGSGTFGTVYHGKWRGTDVAIKRIKRSCFIGRSSEQERLTSEFWHEAEILS
KLHHPNVMAFYGVVKDGPGGTLATVTEYMVNGSLRHVLLSNRHLDRRKRLIIAMDAAFGMEYLHSKSIVHFDLKCDNLLVNL
KDPARPICKVGDFGLSKIKRNTLVTGGVRGTLPWMAPELLSGSSSKVSEKVDVFSFGIVLWEILTGEEPYANMHYGAIIGGIVNNT
LRPTVPNYCDPEWRMLMEQCWAPDPFVRPAFPEIARRLRTMSSSAVHTKPHAVNHQIHK

>AtRAF25

MENITAQLKRGISRQFSTGSIRRTLSRQFTRQSSLDPRRTNMRFSFGRQSSLDPIRRSPDSSKSDDEPHMSVPENLDSTMQLLFMAS
KGDVRGIEELLDEGIDVNSIDLDGRTALHIAACEGHLGVVKALLSRRANIDARDRWGSTAADAKYYGNLDVYNLLKARGAKV
PKTRKTPMTVSNPREVPEYELNPLEVQVRKSDGISKGAYQVAKWNGTRVSVKILDKDSYSDPERINAFRHELTLLEKVRHPNVIQ
FVGAVTQNIPMMIVVEYNPKGDLSVYLQKKGRLSPSKALRFALDIARGMNYLHECKPDPIIHCDLKPKNILLDRGGQLKISGFGM
IRLSKISQDKAKVANHKAHIDLSNYYIAPEVYKDEIFDLRVDAHSFGVILYEITEGVPVFHPRPPEEVARMMCLEGKRPVFKTKSR
SYPPDIKELIEKCWHPEAGIRPTFSEIIIRLDKIVANCSKQGWWKDTFKFPWK

>AtRAF26

MEKKSEEDGNNTTKEKIFRADKIDLKSLDRQLEKHLSRVWSRNLEVNPKAKEEWEIDLAKLETSNVIARGTYGTVYKGIYDGQD
VAVKVLDWEDDGNETTAKTATNRALFRQEVTVWHKLNHPNVTKFVGASMGTTNLNIRSADSKGSLPQQACCVVVEYLPGGTL
KQHLIRHKSKKLAFKAVIKLALDLARGLSYLHSEKIVHRDVKTENMLLDAQKNLKIADFGVARVEALNPKDMTGETGTLGYMA
PEVIDGKPYNRRCDVYSFGICLWEIYCCDMPYPDLSFVDVSSAVVLHNLRPEIPRCCPTALAGIMKTCWDGNPQKRPEMKEVVK
MLEGVDTSKGGGMIPEDQSRGCFCFAPARGP

>AtRAF27

MEEDYQQPRFTIGRQSSMAPEKIPEPSVHSEEEVFEDGEEIDGGVRLMYLANEGDIEGIKELIDSGIDANYRDIDDRTALHVAACQ
GLKDVVELLLDRKAEVDPKDRWGSTPFADAIFYKNIDVIKILEIHGAKHPMAPMHVKTAREVPEYEINPSELDFTQSKEITKGTY
CMAMWRGIQVAVKKLDDEVLSDDDQVRKFHDELALLQRLRHPNIVQFLGAVTQSNPMMIVTEYLPRGDLRELLKRKGQLKPAT
AVRYALDIARGMSYLHEIKGDPIIHRDLEPSNILRDDSGHLKVADFGVSKLVTVKEDKPFTCQDISCRYIAPEVFTSEEYDTKADVF
SFALIVQEMIEGRMPFAEKEDSEASEAYAGKHRPLFKAPSKNYPHGLKTLIEECWHEKPAKRPTFREIIKRLESILHHMGHKRQWR
MRPLTCFQNFEHKKKHNWDLSSHDGSSSGSHL

>AtRAF28

MLENPKFDLHAVGNHNNDNNYYAFTQDFYQKLGEEGTNMSVDSMQTSNAGGSVSMSVDNSSVGSSDALIGHPGLKPMRHPYS
LSDGQSVFRPGKVTHALNDDALAQALMDSKYPTEGLVNYEEWTIDLRKLHMGPAFAQGAFGKLYRGTYNGEDVAIKLLERSDS
NPEKAQALEQQFQQEVSMLAFLKHPNIVRFIGACIKPMVWCIVTEYAKGGSVRQFLTKRQNRAVPLKLAVMQALDVARGMAYV
HERNFIHRDLKSDNLLISADRSIKIADFGVARIEVQTEGMTPETGTYRWMAPEMIQHRPYTQKVDVYSFGIVLWELITGLLPFQN
MTAVQAAFAVVNRGVRPTVPADCLPVLGEIMTRCWDADPEVRPCFAEIVNLLEAAETEIMTNVRKARFRCCMTQPMTVD

>AtRAF29

MAIKEETEESCGSRAVVASITKESPRQHRMKLEVYGEVLQRIQESNYEEANFPGFDDLLWLHFNRLPARYALDVNVERAEDVLTH
QRLLKLAEDPATRPVFEVRCVQVSPTLNGNSGDVDPSDPAVNEDAQSSYNSRSLAPPTFGSSPNFEALTQAYKDHAQDDDSAVNA
QLPNSRPMHEITFSTIDRPKLLSQLTSMLGELGLNIQEAHAFSTADGFSLDVFVVDGWSQEETEGLKDALKKEIRKFKDQPCSKQ
KSITFFEHDKSTNELLPACVEIPTDGTDEWEIDMKQLKIEKKVACGSYGELFRGTYCSQEVAIKILKPERVNAEMLREFSQEVYIM
RKVRHKNVVQFIGACTRSPNLCIVTEFMTRGSIYDFLHKHKGVFKIQSLLKVALDVSKGMNYLHQNNIIHRDLKTANLLMDEHE
VVKVADFGVARVQTESGVMTAETGTYRWMAPEVIEHKPYDHRADVFSYAIVLWELLTGELPYSYLTPLQAAVGVVQKGLRPKIP

KETHPKLTELLEKCWQQDPALRPNFAEIIEMLNQLIREVGDDERHKDKHGGYFSGLKKGHR

>AtRAF30

MVMEDNESCASRVIFDALPTSQATMDRRERIKMEVFDEVLRRLRQSDIEDAHLPGFEDDLWNHFNRLPARYALDVNVERAEDVL

MHKRLLHSAYDPQNRPAIEVHLVQVQPAGISADLDSTSNDAGHSSPTRKSIHPPPAFGSSPNLEALALAASLSQDEDADNSVHNN

SLYSRPLHEITFSTEDKPKLLFQLTALLAELGLNIQEAHAFSTTDGYSLDVFVVDGWPYEETERLRISLEKEAAKIELQSQSWPMQ

QSFSPEKENGQTGARTHVPIPNDGTDVWEINLKHLKFGHKIASGSYGDLYKGTYCSQEVAIKVLKPERLDSDLEKEFAQEVFIMR

KVRHKNVVQFIGACTKPPHLCIVTEFMPGGSVYDYLHKQKGVFKLPTLFKVAIDICKGMSYLHQNNIIHRDLKAANLLMDENEV

VKVADFGVARVKAQTGVMTAETGTYRWMAPEVIEHKPYDHKADVFSYGIVLWELLTGKLPYEYMTPLQAAVGVVQKGLRPTIP

KNTHPKLAELLERLWEHDSTQRPDFSEIIEQLQEIAKEVGEEGEEKKKSSTGLGGGIFAALRRSTTHH

>AtRAF31

MSSDDTIEESLLVDPKLLFIGSKIGEGAHGKVYQGRYGRQIVAIKVVNRGSKPDQQSSLESRFVREVNMMSRVQHHNLVKFIGAC

KDPLMVIVTELLPGMSLRKYLTSIRPQLLHLPLALSFALDIARALHCLHANGIIHRDLKPDNLLLTENHKSVKLADFGLAREESVT

EMMTAETGTYRWMAPELYSTVTLRQGEKKHYNNKVDVYSFGIVLWELLTNRMPFEGMSNLQAAYAAAFKQERPVMPEGISPSL

AFIVQSCWVEDPNMRPSFSQIIRLLNEFLLTLTPPPPQPLPETATNRTNGRAITEFSIRPKGKFAFIRQLFAAKRNINS

>AtRAF32

MGSVTGFYSNEVFELDPKWVVDPQHLFVGPKIGEGAHAKIYEGKYKNKTVAIKIVKRGESPEEIAKRESRFAREVSMLSRVQHK

NLVKFIGACKEPIMVIVTELLLGGTLRKYLVSLRPGSLDIRVAVGYALDIARAMECLHSHGVIHRDLKPESLILTADYKTVKLADFG

LAREESLTEMMTAETGTYRWMAPELYSTVTLRHGEKKHYNHKVDAYSFAIVLWELIHNKLPFEGMSNLQAAYAAAFKNVRPSA

DDLPKDLAMIVTSCWKEDPNDRPNFTEIIQMLLRCLSTISSTELVPPAIKRVFSSENTVLPPESPGTCSLMTVRDKDQIPTDANSAQ

NEVRGSFFFFCC

>AtRAF33

MKEGKDGFVRADQIDLKSLDEQLERHLSRALTLEKNKKKDEEDTTAVAIGGSASSSPVTLNGGGFVGKRKQRLEWEIDPSKLIIK

TVLARGTFGTVHRGIYDGQDVAVKLLDWGEEGHRSEAEIVSLRADFAQEVAVWHKLDHPNVTKFIGATMGASGLQLQTESGPL

AMPNNICCVVVEYLPGGALKSYLIKNRRRKLTFKIVVQLALDLARGLSYLHSQKIVHRDVKTENMLLDKTRTVKIADFGVARVE

ASNPNDMTGETGTLGYMAPEVLNGNPYNRKCDVYSFGICLWEIYCCDMPYPDLTFSEVTSAVVRQNLRPDIPRCCPSALAAVMK

RCWDANPDKRPEMDEVVPMLESIDTTKGGGMIPNDQQQGCLCFRRKRGP

>AtRAF34

MDSLTGFRMEPKWQIDPQLLFVGPKIGEGAHAKVYEGKYKNQTVAIKIVHRGETPEEIAKRDSRFLREVEMLSRVQHKNLVKFIG

ACKEPVMVIVTELLQGGTLRKYLLNLRPACLETRVAIGFALDIARGMECLHSHGIIHRDLKPENLLLTADHKTVKLADFGLAREES

LTEMMTAETGTYRWMAPELYSTVTLRLGEKKHYNHKVDAYSFAIVLWELLHNKLPFEGMSNLQAAYAAAFKNVRPSAESLPEE

LGDIVTSCWNEDPNARPNFTHIIELLLNYLSKVGSPISAIPQRILASKNTLLPPDSPGTSSLMAKLDECGETPKAKSEDKRKGLFFC

FNQCY

>AtRAF35

MDSGSVNSSVTSLVSSLNDEPHRVKFLCSFLGSILPRPQDGKLRYVGGETRIVSVNRDIRYEELMSKMRELYDGAAVLKYQQPDE

DLDALVSVVNDDDVTNMMEEYDKLGSGDGFTRLRIFLFSTPEQDGSLHYVERDDQRESERRYVDALNNLIEGTDFRKLQQYPD

SPRFNLVDDFSMVEPMLNQLSIETGGGSQRGNEIPTAQYSNLHQLRIPRVGSGQMLAQRYGEVEGTWSPFYSPRHHGHHDPRTFQ

EFPSSPSSARYRMPYGEIPDKGLDRMPEEYVRPQASHHPFYEHQAHIPDSVVWVPAGAMPPESKGGFPGNVLHGGPGGYEGGN

GCENCRVPYHRNHQLLEQSNIGNNGFPPVHCAHCPPNRESFLLNTDPKPTHHGAYPNETFGPDRGWMVQQQVNPNPPRIEEGRS

HISNVGRPNDHYTPDYPVSNYPLGQRAGHEISNEGFHDKPLGGIPLNSANRSAEERGFHYGNNLYPPGPDSIHSAGHSHMHHPQP

NIWQNVSNPIAGPPGLPMQINGTVNQTVIRNPIETAPRYSTGMENQGVLVGSPQRISGFDGMSSLGQPSYPNPHLQDRAFPLDPN

WVPSENPTVHNEHLQVREPLPGPLLQTNLTAAPIMQTPVMQTSVESKLAQGGEQFNYVNTGISNGVPYQDKPQPLAGGKKDMG

NLVEVNPSAATLEGAELSVERLSFLPELMESVKRAALEGAAEVKAHPEEAKDQVRPELVENESEHMNAQDEPEIDSDSDNPNNF

KIEQTKAEAEAKSRGLQSIRNDDLEEIRELGHGTYGSVYHGKWKGSDVAIKRIKASCFAGKPSERERLIEDFWKEALLLSSLHHP

NVVSFYGIVRDGPDGSLATVAEFMVNGSLKQFLQKKDRTIDRRKRLIIAMDTAFGMEYLHGKNIVHFDLKCENLLVNMRDPQRP

ICKIGDLGLSKVKQKTLVSGGVRGTLPWMAPELLSGKSNMVSEKIDVYSFGIVMWELLTGEEPYADMHCASIIGGIVNNALRPKI
PQWCDPEWKGLMESCWTSEPTERPSFTEISQKLRTMAAAMNLK
>AtRAF36
MDEEATSWIRRAKFSQTVSYRLNSSKLASLPFMINQDKFSGLKAIPQRSSSSSSASSSDPKLVSSNSQTTGDTSSLEAADVYVVDS
EIQTNPVTHKQRSVSPSPQMAVPDVFKEARSERKRFSTPHPRRVESEKGMKPKLSHKNSFDKRSFNLRSPSGPIRDLGTLRIQERV
KSKKDTGWSKLFDNTGRRVSAVEASEEFRVDMSKLFFGLKFAHGLYSRLYHGKYEDKAVAVKLITVPDDDDNGCLGARLEKQFT
KEVTLLSRLTHPNVIKFVGAYKDPPVYCVLTQYLPEGSLRSFLHKPENRSLPLKKLIEFAIDIARGMEYIHSRRIIHRDLKPENVLID
EEFHLKIADFGIACEEEYCDMLADDPGTYRWMAPEMIKRKPHGRKADVYSFGLVLWEMVAGAIPYEDMNPIQAAFAVVHKNIRP
AIPGDCPVAMKALIEQCWSVAPDKRPEFWQIVKVLEQFAISLEREGNLNLSSSKICKDPRKGLKHWIQKLGPVHAGGGGGSSSSG
LGGSALPKPKFA
>AtRAF37
MRPRGYQRAPSMQKPTDYPTDKTLHPNYPFLMSSHGLKSFESDDEDDDSDSSNDQFAFTINTELLVDVKDISIGDFIGEGSSSTVY
RGLFRRVVPVSVKIFQPKRTSALSIEQRKKFQREVLLLSKFRHENIVRFIGACIEPKLMIITELMEGNTLQKFMLSVRPKPLDLKLSI
SFALDIARGMEFLNANGIIHRDLKPSNMLLTGDQKHVKLADFGLAREETKGFMTFEAGTYRWMAPELFSYDTLEIGEKKHYDH
KVDVYSFAIVFWELLTNKTPFKGKNNIFVAYAASKNQRPSVENLPEGVVSILQSCWAENPDARPEFKEITYSLTNLLRSLSSDTDAT
SSNSKANIATEDSTSSLVQERVVCDCPGLKMSKTKKLKKKTNKLMNMIVPFLKIFKSCMSK
>AtRAF38
MKEKAESGGGVGYVRADQIDLKSLDEQLQRHLSKAWTMEKRKSLSDGEDNVNNTRHNQNNFGHRQLVFQRPLLGGGYSNNN
NSSKNDIIRSTEVEKSRREWEIDPSKLIIKSVIARGTFGTVHRGIYDGQDVAVKLLDWGEEGHRSDAEIASLRAAFTQEVAVWHKL
DHPNVTKFIGAAMGTSEMSIQTENGQMGMPSNVCCVVVEYCPGGALKSFLIKTRRRKLAFKVVIQLSLDLARGLSYLHSQKIVH
RDVKTENMLLDKSRTLKIADFGVARLEASNPNDMTGETGTLGYMAPEVLNGSPYNRKCDVYSFGICLWEIYCCDMPYPDLSFSE
VTSAVVRQNLRPEIPRCCPSSLANVMKRCWDANPEKRPEMEEVVAMLEAIDTSKGGGMIPPDQQQGCFCFRRHRGP
>AtRAF39
METRNETKASPENNLRNRGADGNNSKKDMIFRADKIDLKNLDIQLEKHLSRVWSRSIEKHPKPKEEWEIELAKLEMRNVIARGA
YGIVYKGIYDGQDVAVKVLDWGEDGYATTAETSALRASFRQEVAVWHKLDHPNVTRFVGASMGTANLKIPSSAETENSLPQRAC
CVVVEYIPGGTLKQYLFRNRRKKLAFKVVVQLALDLSRGLSYLHSERIVHRDVKTENMLLDYQRNLKIADFGVARVEAQNPKD
MTGETGTLGYMAPEVLDGKPYNRRCDVYSFGICLWEIYCCDMPYPDLSFADVSSAVVRQNLRPDIPRCCPTALATIMKRCWEAN
PEKRPEMEEVVSLLEAVDTTKGGGMIPEDQRPGCFCFVSGRGP
>AtRAF40
MEASVYGLQIIKNADLEDLTELGSGTYGTVYHGTWRGTDVAIKRIRNSCFAGRSSEQERLTKDFWREAQILSNLHHPNVVAFYGI
VPDGTGGTLATVTEFMVNGSLRHALLKKDRLLDTRKKIIIAMDAAFGMEYLHSKNIVHFDLKCENLLVNLRDPQRPICKVGDLG
LSRIKRNTLVSGGVRGTLPWMAPELLNGSSTRVSEKVDVFSYGISLWEILTGEEPYADMHCGAIIGGIVKNTLRPPIPKSCSPEWKK
LMEQCWSVDPDSRPPFTEITCRLRSMSMEVVTKSKRRENKP
>AtRAF41
MGSASGFYSNEEFELDPKWLVDPRHLFVGPKIGEGAHAKVYEGKYRNQTVAIKIIKRGESPEEIAKRDNRFAREIAMLSKVQHKN
LVKFIGACKEPMMVIVTELLLGGTLRKYLVSLRPKRLDIRLAVGFALDIARAMECLHSHGIIHRDLKPENLILSADHKTVKLADFG
LAREESLTEMMTAETGTYRWMAPELYSTVTLRQGEKKHYNHKVDAYSFAIVLWELILNKLPFEGMSNLQAAYAAAFKNLRPSA
EDLPGDLEMIVTSCWKEDPNERPNFTEIIQMLLRYLTTVSAPQIIPPPNRRVFSSENIVLSPESPGTCSLMSVRDGDVSRQTVNTADS
SEKQTKGSFFSCCS
>AtRAF42
MAHEPSSPSSNLVSNPANLSASGLDYSSDLSNKRVSDGIISGFGSEQVSIDATNRNNPNLGNKRSDMDDEELEKVKFLCSYNGKIIP
RPSDGMLRYVGGQTRIVSVKKNVRFDEFEQKMIQVYGHPVVVKYQLPDEDLDALVSVSSSEDIDNMMEEFEKLVERSSDGSGK
LRVFLFDASSSEVDDSFGILEYGDGVDIGQRYVEAVNGVVVSKESVASGSSNPNSDFSGVDVVDSLGVGQSDFVATTWTSSNFSP
QTYHSNVSRLVPPDPRSSAYVVPMTVHADPPHSFQLETVSEKPIVGKMQQQQQGYTTPSEHHPPAYVESRQEALRQPDIVHSPIQ

LLPSSTSLFSQQPFQDSPLSVSSHQFLPAAHMSMAPLNSQISSTPVLINPVMQTQENLLGNYHAAQKLVPLPTEPRNTAYQGTISPG
IPFDGYGGSQVPPSNHVVLPDGSFYQQVTMAESFQRVNDCHMCQTSFPHMHSDPIMREGNDGSTMYVPYVSSAFYASRPDDIM
RIQQTDKFTGQQSFLNHSNHQERDTLHNANLATAQVETTEPFVNEIVRDVPIKVQVTRQQHPVDPSVAYAQCRELSGLVDNVNI
HAPEIYSNCQNFISPVDKIGKEDIMGTSSQQMARKNMFLHDTSGQSPVSPNIDHTDSAKRLTRVVLPGHESQPKESCVPTQSPLLG
NPGLYLQSLVGGQQFDSAEAQSSNPAYDVVESTFDAANLPSSLSSNPDAANLPSSLSSSVGGADHKESSKSLFSNQDPWNLQTNS
NEDVKPDLLNSSKVILENDLLIGLWFSLKGSEEEHIKQELQNVAEGVAASVLQSSTPSYHEPPIKVDEYAFNSKGEVSRNDEMKQ
QSTHFKDIRNQLLERLNFGYSGSDSLDQLQIIKDSDLEELRELGSGTFGTVYHGKWRGTDVAIKRINDRCFAGKPSEQERMIDDF
WNEAQNLAGLHHPNVVAFYGVVLDSPGGSVATVTEYMVNGSLRNALQKNVRNFDRCKRQLIAMDIAFGMEYLHGKKIVHFDL
KSDNLLVNLRDPHRPICKVGDLGLSKVKCQTLISGGVRGTLPWMAPELLNGTSSLVSEKVDVFSFGIVLWELFTGEEPYADLHYG
AIIGGIVSNTLRPQIPDFCDMDWKLLMERCWSAEPSERPSFTEIVNELRTMATKLPSKEQGSTQGPQS

>AtRAF43

MDGEVTSWIRRANFSHTVCYRMITPSLESMPFTVNQEKMQRNPVTNKKRSVSPLPHMALSDAFIEAKSDIKRFSTPHPRRVEPEK
GMKAKSSSRKDSSEKKSVNLRSLSHSGPIRDLSTQKVKERGKSKIDKKSSKSVDYRGSKVSSAGVLEECLIDVSKLSYGDRFAHG
KYSQIYHGEYEGKAVALKIITAPEDSDDIFLGARLEKEFIVEATLLSRLSHPNVVKFVGVNTGNCIITEYVPRGSLRSYLHKLEQKS
LPLEQLIDFGLDIAKGMEYIHSREIVHQDLKPENVLIDNDFHLKIADFGIACEEEYCDVLGDNIGTYRWMAPEVLKRIPHGRKCD
VYSFGLLLWEMVAGALPYEEMKFAEQIAYAVIYKKIRPVIPTDCPAAMKELIERCWSSQTDKRPEFWQIVKVLEHFKKSLTSEGKL
NLLPSQICPELKKCPKFWIHIFGSFHHHSSGGGSSSNNSALPKPKFA

>AtRAF44

MAISPTMMLNANYPFFMSAFGSDDNNDESDNQFDFNISRELLLNPKDIMRGEMIGEGGNSIVYKGRLKNIVPVAVKIVQPGKTSA
VSIQDKQQFQKEVLVLSSMKHENIVRFVGACIEPQLMIVTELVRGGTLQRFMLNSRPSPLDLKVSLSFALDISRAMEYLHSKGIIH
RDLNPRNVLVTGDMKHVKLADFGLAREKTLGGMTCEAGTYRWMAPEVCSREPLRIGEKKHYDQKIDVYSFALIFWSLLTNKTP
FSEIPSISIPYFVNQGKRPSLSNIPDEVVPILECCWAADSKTRLEFKDITISLESLLKRFCSERSNNEITITEDEAYDDEIEELETTWLLP
KRYIKLKKPKKIKQNVMKKILPFFKKFISSKW

>AtRAF45

MISRMIFRNYPSHNESDDEPFHFSISRELLLDRNDVVVGEMIGEGAYSIVYKGLLRNQFPVAVKIMDPSTTSAVTKAHKKTFQKEV
LLLSKMKHDNIVKFVGACIEPQLIIVTELVEGGTLQRFMHSRPGPLDLKMSLSFALDISRAMEFVHSNGIIHRDLNPRNLLVTGDL
KHVKLADFGIAREETRGGMTCEAGTSKWMAPEVVYSPEPLRVGEKKEYDHKADIYSFAIVLWQLVTNEEPFPDVPNSLFVPYLV
SQGRRPILTKTPDVFVPIVESCWAQDPDARPEFKEISVMLTNLLRRMSSDSSIGTTLPDGEAYEGEMEESENSPLLQEHFCKVKKP
KEKKKKKKLVKMRFPFFKKFKVWLYNYKP

>AtRAF46

MDNIAAQLKRGISRQFSTGSMRRTLSRQFTRQNSLDPRRNNMRFSFGRQSSLDPIRRSPESLSCEPHMSVPENLDSTMQLLFMAS
KGDVNGVEELLNEGIDVNSIDLDGRTALHIASCEGHYDVVKVLLSRRANIDARDRWGSTAAVDAKYYGNVEVYNLLKARGAK
APKTRKTPMTVGNPKEVPEYELNPLELQVRKVDGISKGTYQVAKWNGTRVSVKIFDKDSYSDPERVNAFTNELTLLAKARHPNI
VQFVGAVTQNLPMMIVVECNPKGDLSVYLQKKGRLSPSKALRFALDIARGMNYLHECKPDPIIHCELMPKNILLDRGGQLKISGF
GLIKLSKIGEDSAKVVNHEAQIDKSNYYIAPEIYKDEVFDKRADVHSFGVILYELTEGVSLFHPKPPEEVAESICIEGKRPTIRTKSK
SYPPELKELIEECWHPEISVRPIFSEIIIRLDKIVTNCSKQGWWKDTFKFPWK

>AtRAF47

MTIKPKSPARFKLGRQSSLAPESRTPIDTLTEDEDDDLAAAATAGIGDPTIRLMYLANEGDIDGINKMLDSGTNVDYRDIDARTAL
HVAACQGRTDVVELLLSRGAKVDTKDRWGSTPLADAVYYKNHDVIKLLEKHGAKPTIAPMHVLTDKEVPEYEIHPTELDFSNSV
KISKGTFNKASWRGIDVAVKTFGEEMFTDEDKVNAFRDELALLQKIRHPNVVQFLGAVTQSTPMMIVTEYLPKGDLRQYLDRKG
PLMPAHAVKFALEIARGMNYLHEHKPEAIIHCDLEPPNILRDDSGHLKVADFGVSKLLVVKKTVKKDRPVVTCLDSSWRYMAPE
VYRNEEYDTKVDVFSFALILQEMIEGCEPFHEIEDREVPKAYIEDERPPFNAPTKSYPFGLQELIQDCWDKEASKRPTFRVIISTLEL
ISDRIARKRSWKVMLGRCLPRFRLFTKRDYVNPGGSNRSSGSFNR

>AtRAF48

MASGGGEADKSLEIGSGTADPKIGGTGSRSAGEERYFRADTLDFSKWDLHMGQTSTSSVLTNSASTSAPAPAMQEWEIDLSKLD
MKHVLAHGTYGTVYRGVYAGQEVAVKVLDWGEDGYATPAETTALRASFEQEVAVWQKLDHPNVTKFIGASMGTSDLRIPPAGD
TGGRGNGAHPARACCVVVEYVAGGTLKKFLIKKYRAKLPIKDVIQLALDLARGLSYLHSKAIVHRDVKSENMLLQPNKTLKIAD
FGVARVEAQNPQDMTGETGTLGYMAPEVLEGKPYNRKCDVYSFGVCLWEIYCCDMPYADCSFAEISHAVVHRNLRPEIPKCCPH
AVANIMKRCWDPNPDRRPEMEEVVKLLEAIDTSKGGGMIAPDQFQGCLCFFKPRGP
