# Supplementary material for: Genome-wide identification and analysis of mitogen activated protein kinase kinase kinase gene family in grapevine (Vitis vinifera)
Source: BMC Plant Biol. 2014 Aug 27;14:219. doi: 10.1186/s12870-014-0219-1 (PMC4243721; doi:10.1186/s12870-014-0219-1)
Supplement: Additional file 8: — VviMAPKKK genes expression values under different biotic and abiotic stimuli. [file 12870_2014_219_MOESM8_ESM.pdf]

Treatment	Time	VviMAPKK4	VviMAPKK5	VviMAPKK22	VviMAPKK23	VviMAPKK24	VviMAPKK25	VviMAPKK26	VviMAPKK27	VviMAPKK28	VviMAPKK29	VviMAPKK30	VviMAPKK31	VviMAPKK32	VviMAPKK33	VviMAPKK34	VviMAPKK35	VviMAPKK36	VviMAPKK37	VviMAPKK38	VviMAPKK39	VviMAPKK40	VviMAPKK41	VviMAPKK42	VviMAPKK43	VviMAPKK44	VviMAPKK45	VviMAPKK46	VviMAPKK47	VviMAPKK48	VviMAPKK49	VviMAPKK50	VviMAPKK51	VviMAPKK52	VviMAPKK53	VviMAPKK54	VviMAPKK55	VviMAPKK56	VviMAPKK57	VviMAPKK58	VviMAPKK59	VviMAPKK60	VviMAPKK61	VviMAPKK62	VviMAPKK63	VviMAPKK64
**E. necator treatment**	4h	0.36	0.52	0.39	1.16	1.59	0.79	0.90	0.50	0.52	0.57	0.50	2.74	1.20	0.23	1.04	1.14	0.37	1.21	4.86	0.87	0.57	0.65	0.79	0.68	0.96	0.97	2.71	0.68	0.84	1.54	6.07	0.47	1.07	0.98	0.22	0.30	1.27	1.04	0.47	0.76	0.84	0.81	0.52	0.80	0.94
	8h	0.46	1.42	1.07	0.81	0.67	1.01	1.07	0.86	1.07	2.08	0.95	1.87	3.93	0.71	0.46	1.04	0.80	1.50	1.76	3.31	0.66	1.32	0.96	1.27	1.31	0.96	4.05	1.21	1.07	1.35	22.15	0.60	1.20	1.08	0.30	1.44	1.06	1.30	0.87	1.16	1.83	0.97	1.19	1.26	1.75
	12h	1.50	0.82	0.94	1.68	0.59	2.73	3.10	1.28	0.62	1.49	0.76	2.65	7.50	1.02	1.43	1.16	1.05	1.23	2.89	2.21	0.68	1.09	1.15	1.25	2.59	1.41	3.12	1.31	2.07	2.12	9.74	0.96	1.51	1.82	1.01	1.07	1.13	0.84	0.75	1.00	3.02	1.07	1.09	0.92	2.06
	24h	0.46	0.83	0.93	0.86	1.04	0.89	0.89	1.32	2.19	1.95	0.67	1.39	15.86	1.43	6.90	1.46	1.23	1.87	5.68	11.74	1.01	0.87	1.13	0.96	1.60	1.40	2.70	1.01	0.67	0.78	27.55	0.35	0.81	1.14	0.27	1.26	0.85	0.88	1.43	0.90	1.04	1.00	1.51	1.40	2.50
	48h	0.31	1.78	0.29	0.46	2.25	0.66	0.95	1.54	1.82	3.28	1.57	2.59	3.11	1.39	11.91	1.57	1.19	1.43	3.34	0.89	2.35	1.05	1.54	1.53	1.16	1.30	10.97	1.57	0.43	0.85	16.56	0.20	0.66	0.57	0.17	1.23	1.24	1.25	2.79	1.14	1.41	1.78	1.85	0.89	2.67
	72h	0.21	0.34	1.10	0.95	1.26	0.62	0.77	1.46	0.64	0.44	0.82	0.90	2.12	0.34	4.63	0.65	0.87	0.91	0.75	0.46	0.52	0.56	0.75	0.59	1.29	0.88	1.28	0.83	1.00	0.82	9.93	0.44	0.73	0.54	0.31	0.46	0.87	0.56	1.13	0.95	1.13	0.60	0.67	0.36	0.59

Treatment	Time	VviMAPKK4	VviMAPKK5	VviMAPKK22	VviMAPKK23	VviMAPKK24	VviMAPKK25	VviMAPKK26	VviMAPKK27	VviMAPKK28	VviMAPKK29	VviMAPKK30	VviMAPKK31	VviMAPKK32	VviMAPKK33	VviMAPKK34	VviMAPKK35	VviMAPKK36	VviMAPKK37	VviMAPKK38	VviMAPKK39	VviMAPKK40	VviMAPKK41	VviMAPKK42	VviMAPKK43	VviMAPKK44	VviMAPKK45	VviMAPKK46	VviMAPKK47	VviMAPKK48	VviMAPKK49	VviMAPKK50	VviMAPKK51	VviMAPKK52	VviMAPKK53	VviMAPKK54	VviMAPKK55	VviMAPKK56	VviMAPKK57	VviMAPKK58	VviMAPKK59	VviMAPKK60	VviMAPKK61	VviMAPKK62	VviMAPKK63	VviMAPKK64
**SA treatment**	4h	0.66	0.83	1.00	1.26	1.66	2.06	0.63	0.90	2.44	0.60	0.40	3.38	0.40	0.57	0.11	2.21	0.31	0.60	5.35	0.11	0.48	0.53	0.85	0.52	0.63	0.71	0.40	0.63	0.96	1.15	0.48	0.41	3.56	1.44	0.63	0.68	0.77	0.86	0.62	0.93	1.14	0.66	0.78	0.78	1.62
	8h	1.01	1.09	1.40	0.48	0.77	2.17	0.73	0.92	1.65	2.09	0.80	1.28	0.88	1.42	0.07	2.64	0.64	0.56	1.09	3.13	0.63	1.05	0.29	0.34	0.77	1.02	0.09	1.45	1.39	1.18	3.14	0.74	2.15	0.95	0.74	0.62	0.77	0.89	0.96	0.87	2.75	0.73	0.57	1.33	0.80
	12h	0.64	1.60	1.47	1.09	1.56	1.60	0.89	2.99	1.30	2.21	0.76	1.95	1.83	1.15	0.05	2.50	0.44	0.70	1.73	0.37	0.52	1.56	1.34	0.75	1.29	1.05	0.05	0.76	1.00	1.28	1.90	0.37	2.41	1.56	1.34	1.04	1.02	1.37	1.47	1.04	5.15	0.80	1.39	1.44	9.48
	24h	0.62	1.01	1.67	0.70	0.41	2.00	0.74	1.39	2.13	2.83	0.59	1.13	4.40	1.68	2.80	1.75	0.90	1.02	4.01	2.22	0.89	0.87	1.21	0.92	0.95	1.01	0.15	0.64	0.87	1.04	0.71	0.52	1.16	1.05	1.10	1.34	1.13	1.18	1.19	0.59	2.11	0.87	1.02	1.12	1.48
	48h	0.56	1.52	0.58	0.64	2.37	0.92	0.79	1.00	0.96	2.86	1.55	1.99	1.34	1.44	8.53	1.37	0.46	1.45	1.44	2.73	1.24	0.83	1.09	1.09	1.04	1.11	2.58	1.07	0.49	0.94	6.15	0.36	0.76	0.85	0.38	0.62	1.11	0.94	1.67	0.91	1.54	0.96	1.03	0.53	1.91
	72h	0.42	0.95	2.25	1.35	4.39	1.46	1.00	0.86	0.75	1.55	1.13	1.35	0.85	0.62	1.55	0.88	1.03	1.07	0.45	0.55	1.33	0.98	1.26	1.16	0.55	1.76	0.14	1.19	1.53	1.95	0.57	0.96	1.02	1.24	1.50	0.99	1.24	1.00	0.71	1.00	1.14	1.23	1.24	1.23	1.01

Treatment	Time	VviMAPKK4	VviMAPKK5	VviMAPKK22	VviMAPKK23	VviMAPKK24	VviMAPKK25	VviMAPKK26	VviMAPKK27	VviMAPKK28	VviMAPKK29	VviMAPKK30	VviMAPKK31	VviMAPKK32	VviMAPKK33	VviMAPKK34	VviMAPKK35	VviMAPKK36	VviMAPKK37	VviMAPKK38	VviMAPKK39	VviMAPKK40	VviMAPKK41	VviMAPKK42	VviMAPKK43	VviMAPKK44	VviMAPKK45	VviMAPKK46	VviMAPKK47	VviMAPKK48	VviMAPKK49	VviMAPKK50	VviMAPKK51	VviMAPKK52	VviMAPKK53	VviMAPKK54	VviMAPKK55	VviMAPKK56	VviMAPKK57	VviMAPKK58	VviMAPKK59	VviMAPKK60	VviMAPKK61	VviMAPKK62	VviMAPKK63	VviMAPKK64
**ETH treatment**	4h	0.63	0.56	1.77	2.98	2.65	1.54	0.55	0.45	0.58	0.35	0.39	2.69	0.69	0.31	1.18	1.28	0.28	0.70	1.96	0.90	0.54	0.71	0.80	0.67	2.26	0.58	1.09	0.46	0.72	1.00	0.57	0.62	1.71	0.69	0.59	0.42	1.12	1.11	0.40	0.58	1.22	0.62	0.72	0.59	2.92
	8h	1.55	1.54	5.84	3.73	4.98	2.25	1.64	1.06	1.30	1.22	0.51	1.75	1.17	0.53	4.02	2.33	0.35	1.37	0.61	17.39	0.97	1.13	1.07	1.20	5.51	0.93	3.44	0.76	1.60	2.08	2.53	1.96	5.38	0.97	2.31	1.00	1.25	1.57	1.08	0.81	6.32	0.80	1.39	0.88	9.87
	12h	1.26	0.95	4.03	2.89	1.02	1.74	1.19	0.92	1.19	0.41	0.25	0.95	2.31	0.63	1.14	1.43	0.22	0.33	0.34	1.26	0.56	1.28	1.34	1.02	2.28	0.99	0.51	0.51	1.43	1.39	0.98	0.86	4.07	1.61	2.12	0.62	1.32	1.83	0.89	1.07	9.43	0.78	1.20	0.99	6.28
	24h	0.62	0.75	2.72	1.40	1.04	1.76	0.79	0.56	1.53	0.89	0.40	0.69	5.69	0.86	4.24	2.07	0.45	1.04	2.06	12.30	0.98	1.01	0.89	0.69	1.05	1.04	1.44	0.68	0.54	0.84	2.02	0.84	1.70	0.79	1.47	1.38	1.13	1.42	0.90	0.58	0.73	0.67	1.11	1.00	3.68
	48h	0.72	0.82	1.25	1.02	1.52	1.18	0.95	0.76	1.30	1.12	0.68	0.88	0.63	0.91	5.30	1.32	0.26	1.17	0.55	5.67	0.82	0.82	1.01	0.89	1.08	0.72	7.31	0.83	0.42	0.80	1.52	0.59	1.59	0.67	0.54	0.83	1.16	1.59	1.66	0.72	2.19	0.85	1.26	0.56	4.73
	72h	0.71	0.94	4.75	4.06	1.66	1.90	1.51	1.61	1.06	1.25	0.95	0.22	0.89	0.80	7.30	0.83	0.86	0.60	0.22	1.33	1.61	1.12	1.02	1.11	1.32	1.45	1.16	0.56	1.33	1.44	0.60	1.62	2.24	1.20	2.27	1.08	1.38	1.06	1.69	0.80	0.62	0.94	1.46	0.50	4.03

Treatment	Time	VviMAPKK4	VviMAPKK5	VviMAPKK22	VviMAPKK23	VviMAPKK24	VviMAPKK25	VviMAPKK26	VviMAPKK27	VviMAPKK28	VviMAPKK29	VviMAPKK30	VviMAPKK31	VviMAPKK32	VviMAPKK33	VviMAPKK34	VviMAPKK35	VviMAPKK36	VviMAPKK37	VviMAPKK38	VviMAPKK39	VviMAPKK40	VviMAPKK41	VviMAPKK42	VviMAPKK43	VviMAPKK44	VviMAPKK45	VviMAPKK46	VviMAPKK47	VviMAPKK48	VviMAPKK49	VviMAPKK50	VviMAPKK51	VviMAPKK52	VviMAPKK53	VviMAPKK54	VviMAPKK55	VviMAPKK56	VviMAPKK57	VviMAPKK58	VviMAPKK59	VviMAPKK60	VviMAPKK61	VviMAPKK62	VviMAPKK63	VviMAPKK64
**H_2O_2 Treatment**	4h	0.62	1.92	1.33	1.85	2.02	1.23	0.84	0.84	0.67	0.18	0.79	3.83	1.04	0.65	4.76	1.25	0.66	1.84	6.01	4.03	0.99	0.96	0.92	1.04	1.92	1.09	2.95	0.63	1.18	2.11	1.44	0.39	2.93	1.03	0.24	0.52	1.03	1.12	0.71	0.85	0.66	0.94	0.80	0.77	1.68
	8h	0.99	1.50	1.83	2.18	1.47	1.21	1.11	0.84	0.86	0.82	1.20	0.53	0.87	0.77	4.41	1.48	1.22	2.01	1.42	24.93	0.60	1.00	1.37	1.18	2.99	0.98	3.66	1.07	1.13	1.77	1.37	0.65	2.59	1.11	0.32	1.11	0.88	0.99	0.88	0.99	0.61	1.06	1.19	0.83	1.23
	12h	0.85	1.89	1.86	4.24	0.33	1.03	1.09	1.60	0.96	1.49	0.93	1.24	1.77	0.94	2.64	1.40	1.48	1.21	1.99	6.22	0.62	1.32	1.23	1.46	2.53	1.24	1.05	0.81	1.56	2.40	6.74	0.57	2.56	1.30	0.52	1.27	0.97	0.84	1.90	0.98	4.61	1.11	0.92	0.77	2.25
	24h	0.69	1.36	2.93	3.06	0.47	1.47	1.13	0.98	2.32	2.71	0.74	2.00	7.39	1.13	43.93	2.22	2.03	1.65	5.23	53.94	0.91	1.29	1.09	1.29	0.87	1.33	2.35	1.08	0.81	1.86	4.06	0.58	2.34	1.42	0.30	1.52	1.14	1.26	1.81	0.73	2.47	1.21	1.25	1.51	1.51
	48h	0.56	1.62	1.35	3.10	0.74	0.90	0.85	0.87	1.67	1.88	1.06	1.65	0.89	1.10	15.57	0.44	1.92	1.42	0.76	4.68	1.52	1.26	0.84	1.82	1.16	1.38	9.64	0.32	0.91	1.58	3.15	0.48	1.94	1.00	0.20	1.09	0.99	1.09	1.42	0.62	0.55	1.39	1.49	0.78	3.00
	72h	0.35	0.40	1.58	3.04	0.59	0.74	0.49	0.51	0.39	0.30	0.65	0.51	0.56	0.30	6.54	0.44	0.82	0.74	0.30	3.77	0.54	0.59	0.83	0.88	1.02	0.93	1.29	0.49	0.75	0.95	2.70	0.43	0.91	0.71	0.20	0.47	0.72	0.62	0.40	0.63	0.14	0.94	0.75	0.61	0.52

Treatment	Time	VviMAPKK4	VviMAPKK5	VviMAPKK22	VviMAPKK23	VviMAPKK24	VviMAPKK25	VviMAPKK26	VviMAPKK27	VviMAPKK28	VviMAPKK29	VviMAPKK30	VviMAPKK31	VviMAPKK32	VviMAPKK33	VviMAPKK34	VviMAPKK35	VviMAPKK36	VviMAPKK37	VviMAPKK38	VviMAPKK39	VviMAPKK40	VviMAPKK41	VviMAPKK42	VviMAPKK43	VviMAPKK44	VviMAPKK45	VviMAPKK46	VviMAPKK47	VviMAPKK48	VviMAPKK49	VviMAPKK50	VviMAPKK51	VviMAPKK52	VviMAPKK53	VviMAPKK54	VviMAPKK55	VviMAPKK56	VviMAPKK57	VviMAPKK58	VviMAPKK59	VviMAPKK60	VviMAPKK61	VviMAPKK62	VviMAPKK63	VviMAPKK64
**Drought treatment**	4d	1.53	0.80	1.01	0.79	0.25	1.41	1.32	0.64	1.41	0.30	2.06	1.02	0.67	1.86	0.13	2.00	0.34	0.86	1.34	0.52	0.97	1.16	1.23	0.99	1.29	1.52	0.03	1.40	0.82	1.50	0.60	1.85	0.81	1.34	2.05	1.63	0.99	1.18	1.32	1.07	1.07	1.01	1.24	1.37	0.55
	8d	3.43	17.38	40.40	175.43	1.66	5.56	6.86	6.78	19.25	12.87	6.33	1.85	4.17	4.17	2.73	3.72	6.22	5.95	7.01	3.17	5.58	2.86	6.77	3.76	3.51	5.50	0.09	0.33	7.56	7.48	2.73	20.79	18.81	2.10	109.26	4.29	4.52	2.02	8.57	16.77	6.02	3.01	1.21	3.37	14.85
	12d	3.19	2.27	13.36	20.59	0.53	4.27	4.35	1.08	4.40	4.14	0.75	1.49	0.32	2.32	1.32	2.65	0.78	0.63	1.47	0.51	8.08	1.49	3.40	1.66	1.86	2.33	0.05	0.57	3.26	2.85	0.17	28.18	7.82	1.78	60.90	3.94	2.20	2.36	5.54	4.13	2.41	1.08	3.46	3.05	6.11
